# Cell lineage-specific methylome and genome alterations in gout

**DOI:** 10.18632/aging.202353

**Published:** 2021-01-20

**Authors:** Chia-Chun Tseng, Wei-Ting Liao, Man-Chun Wong, Chung-Jen Chen, Su-Chen Lee, Jeng-Hsien Yen, Shun-Jen Chang

**Affiliations:** 1 Graduate Institute of Clinical Medicine, College of Medicine, Kaohsiung Medical University, Kaohsiung, Taiwan; 2 Division of Rheumatology, Department of Internal Medicine, Kaohsiung Medical University Hospital, Kaohsiung, Taiwan; 3 Department of Biotechnology, College of Life Science, Kaohsiung Medical University, Kaohsiung, Taiwan; 4 Department of Medical Research, Kaohsiung Medical University Hospital, Kaohsiung, Taiwan; 5 Department of Internal Medicine, Kaohsiung Municipal Ta-Tung Hospital, Kaohsiung, Taiwan; 6 Division of General Internal Medicine, Department of Internal Medicine, Kaohsiung Medical University Hospital, Kaohsiung, Taiwan; 7 Laboratory Diagnosis of Medicine, College of Medicine, Kaohsiung Medical University, Kaohsiung, Taiwan; 8 Institute of Biomedical Sciences, National Sun Yat-Sen University, Kaohsiung, Taiwan; 9 Department of Biological Science and Technology, National Chiao-Tung University, Hsinchu, Taiwan; 10 Department of Kinesiology, Health and Leisure Studies, National University of Kaohsiung, Kaohsiung, Taiwan

**Keywords:** gout, inflammation, methylation, interleukin-1β

## Abstract

In this study, we examined data from 69 gout patients and 1,455 non-gout controls using a MethylationEPIC BeadChip assay and Illumina HiSeq platform to identify lineage-specific epigenetic alterations and associated genetic factors that contributed to gouty inflammation. Cell lineage-specific differentially methylated sites were identified using CellDMC after adjusting for sex, age, alcohol drinking, smoking status, and smoking history (total pack-years). Different cell lineages displayed distinct differential methylation. Ingenuity Pathway Analysis and NetworkAnalyst indicated that many differential methylated sites were associated with interleukin-1β expression in monocytes. On the UCSC Genome Browser and WashU Epigenome Browser, metabolic trait, cis-methylation quantitative trait loci, genetic, and functional annotation analyses identified nine methylation loci located in interleukin-1β-regulating genes (*PRKCZ, CIDEC, VDAC1, CPT1A, BIRC2, BRCA1, STK11,* and *NLRP12*) that were associated specifically with gouty inflammation. All nine sites mapped to active regulatory elements in monocytes. MoLoTool and ReMap analyses indicated that the nine methylation loci overlapped with binding sites of several transcription factors that regulated interleukin-1β production and gouty inflammation. Decreases in *PRKCZ* and *STK11* methylation were also associated with higher numbers of first-degree relatives who also had gout. The gouty-inflammation specific methylome and genome alterations could potentially aid in the identification of novel therapeutic targets.

## INTRODUCTION

Gout is the most common form of inflammatory arthritis worldwide, and its prevalence increases with age [1]. Gout is associated with numerous comorbidities, such as increased low-density lipoprotein levels, elevated triglyceride levels, and altered glycosylated hemoglobin (HbA_1c_) levels [2, 3]. Despite increases in associated health and economic costs, gout remains a poorly controlled disease, and therapeutic choices are limited due to side effects or contraindications [4]. Novel drug targets and therapeutics that exhibit improved tolerability are therefore urgently needed.

Previous studies have shown that the development of gout consists of two steps: hyperuricemia-elicited monosodium urate crystal deposition and crystal-activated gouty inflammation [5–9]. In the first step, serum uric acid levels increase, resulting in a hyperuricemic state and thereby promoting crystal deposition. In the second step, monosodium urate crystals activate interleukin-1β (IL-1β) production, resulting in a gouty attack [5–9]. The following evidence indicates that distinct pathogenic mechanisms underlie these two steps: (a) most hyperuricemia patients do not develop gout even after decades [10], and (b) colchicine, which effectively reverses gouty inflammation, has no influence whatsoever on urate metabolism.

To date, much effort has been devoted to elucidating the mechanisms of hyperuricemia, and a multitude of associated proteins, such as *ABCG2,* *SLC2A9, GCKR* and *SLC17A1*, have been identified [9]. In contrast, relatively little is known regarding mechanisms underlying gouty inflammation, although increases in monocyte/macrophage-derived IL-1β induced by monosodium urate crystals play an important role [9]. However, anti-IL-1 therapies are associated with infectious complications [4, 11]. Targeting upstream gout-specific molecules in the IL-1 signaling cascade might reduce such side effects. However, the relationship between monosodium urate crystals and IL-1β levels remains poorly understood.

Studies have shown that gout’s heritability is approximately 30%, but sequence variants explain less than 10% of the risk of developing gout [12, 13]. Epigenetic modifications, which are also heritable and can account for parent-of-origin patterns of inheritance [14], may also be implicated in the heritability of gout. Furthermore, although specific environmental factors are known to increase the risk of gout [12], many more environmental factors can impact the epigenome, and these environmentally-induced epigenomic changes can exert long-term effects on disease phenotypes [15].

Interestingly, DNA methylation regulates cytokine expression and inflammation in macrophages, which are crucial in gouty inflammation [16–18]. DNA methylation might therefore act as a potential mechanism that links heritability, environmental factors, immune biology, and gout. Previous epigenome-wide association studies (EWAS) have demonstrated numerous associations between DNA methylation, inflammation, and disease development [19]. However, the extent to which DNA methylation influences gout and related genetic factors is not fully understood. Moreover, previous EWAS analysis methods couldn’t identify disease-associated genes at a cell-type-specific resolution, hindering further translational research [20]. However, computational model that is validated for cell-type-specific EWAS was recently developed [20]. Here, we employed this method to conduct an integrative analysis of cell lineage-specific methylome and genome alterations in gout. Our goal was to identify cell lineage-specific DNA methylation sites associated with gouty inflammation and their relationships to other genetic variants and clinical characteristics.

## RESULTS

Sixty-nine patients with gout and 1,455 non-gout controls underwent concurrent methylation profiling and whole genome sequencing and were included in cell lineage-specific methylation and cis-methylation quantitative trait loci (cis-meQTL)/genetic analyses. Gout patients were predominantly males (60 males, nine females) with a mean age of 52.58 ± 10.98 years and a mean uric acid level of 7.13 ± 1.96 mg/d, which are similar to the characteristics of gout patients in other studies [21] (Table 1).

**Table 1:** Characteristics of study participants.

**Characteristics**	**Participants with gout (n=69)**	**Participants without gout (n=1455)**
Age (years)	52.58 ± 10.98	49.16 ± 11.15
Sex, male/female	60/9	683/772
Uric acid (mg/dl)	7.13 ± 1.96	5.53 ± 1.39
Hyperuricemia (uric acid >7 mg/dl)	NA	207 (14.23%)
Low density lipoprotein (mg/dl)	124.96 ± 34.15	121.69 ± 32.35
Triglyceride (mg/dl)	144.01 ±86.38	114.33 ± 95.45
HbA_1c_ (%)	5.96 ± 0.78	5.71 ± 0.73

The results were expressed as mean ± standard deviation. HbA_1C_: glycosylated hemoglobin. NA: not applicable.

After quality control (Supplementary Figure 1, Step I), CellDMC analysis identified 1,763 cytosine-phosphate-guanine dinucleotide (CpG) sites across promoter (TSS200, TSS1500) [22], 5'UTR, gene body, 3'UTR, and intergenic regions exhibited differential methylation in monocytes, eosinophils, neutrophils, NK cells, B cells, CD4+ T cells, and CD8+ T cells in gout patients after correcting for sex, age, alcohol drinking, smoking status, smoking history (total pack-years), and cell fractions [20] (Supplementary Table 2).

To clarify whether different cell lineages displayed distinct differential methylation patterns, we examined the overlap of differential methylation between different cell lineages at the CpG site and gene levels (Supplementary Figure 1, Step IIa). There was little overlap (overlap rate < 0.5) [23] in differentially methylated CpG sites among different cell lineages (Supplementary Figure 2A). Similarly, little overlap in differential methylation at the gene level was observed (Supplementary Figure 2B). These results suggest that DNA methylome signatures are relatively unique in different cell lineages.

### Epigenetic modifications were associated with IL-1 signaling in multiple cell lineages

**Figure 1:**
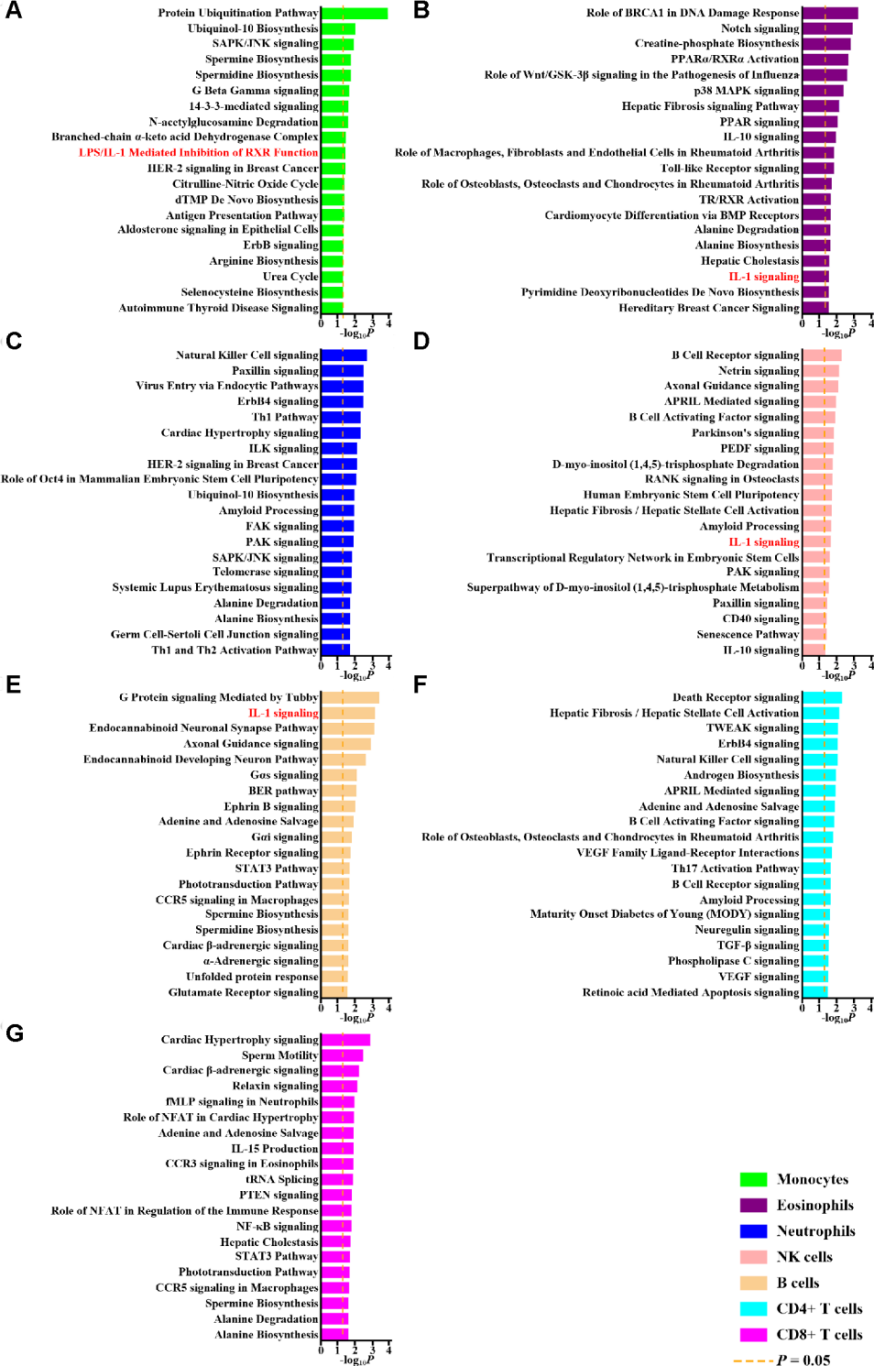
**Pathway analysis of differentially methylated genes.** The top 20 pathways of differentially methylated genes in monocytes (**A**), eosinophils (**B**), neutrophils (**C**), NK cells (**D**), B cells (**E**), CD4+ T cells (**F**) and CD8+ T cells (**G**) revealed by Ingenuity Pathway Analysis and corresponding *P* values are shown. The dashed orange lines represent *P* values of 0.05. Pathways related to interleukin-1 (IL-1) are highlighted with red.

To examine the functions of differentially methylated genes, we performed separate pathway enrichment analyses for monocytes, eosinophils, neutrophils, NK cells, B cells, CD4+ T cells, and CD8+ cells (Supplementary Figure 1, Step IIb; Figure 1). Genes related to IL-1 signaling pathways were enriched among the differentially methylated genes in multiple cellular subsets (Figure 1A, 1B, 1D, 1E). This might help account for the therapeutic efficacy of an IL-1β blocking agent in treating gout [4]. These results indicate that IL-1 regulation is a critical mechanism underlying gout despite differential methylation in different cell types; we therefore examined the impacts of this differential methylation on IL-1β expression.

Although the role of IL-1β in gout has already been established [9], mechanistic links between monosodium urate crystals and IL-1β production in gout have not been identified. We explored whether gout-associated differential methylation might be implicated in regulation of IL-1β. Previous work indicated that IL-1β is primarily produced by monocytes/macrophages in gout [24, 25]. Consistent with these reports, the protein-protein interaction network constructed with genes to which monocyte-specific CpG sites were mapped (Supplementary Figure 1, Step IIc) pinpointed several hub genes (those directly connected to many interacting partners, indicating higher importance) with well-established IL-1β regulatory functions (Supplementary Figure 3). We therefore focused on monocyte-specific differentially methylated CpG sites in genes known to increase or decrease IL-1β production (Supplementary Figure 1, Step IId). Of the 58 monocyte-specific differentially methylated CpG sites, 15 were located in genes previously found to affect IL-1β expression (Table 2, Supplementary Table 3) [26–37]; no evidence was found indicating that genes to which the remaining 43 CpG sites were mapped directly affect IL-1β expression (Supplementary Table 4).

**Table 2:** Surviving monocyte-specific methylation sites.

**CpG site**	**Δβ^a^**	***P***	**FDR**	**Chr**	**Position^b^**	**Gene (Alias)**	**Genomic features^c^**	**CpG features**	**References^d^**
**Increases/decreases interleukin-1β (IL-1β) in monocyte/macrophage lineage cells**									
cg03795507	-1.00	3.42×10^-7^	1.60×10^-3^	5	133340866	*VDAC1*	TSS200	island	↑IL-1β [26]
cg10257063	-0.71	2.59×10^-6^	9.04×10^-3^	11	68587159	*CPT1A*	5'UTR	open sea	↑ IL-1β [27]
cg16975613	1.00	2.66×10^-12^	5.77×10^-8^	11	102217719	*BIRC2* (c*IAP1*) [28]	TSS1500	island	↓IL-1β [29]
cg17151991	-0.52	1.42×10^-6^	5.38×10^-3^	19	1207421	*STK11* (*LKB1*) [30]	Body	island	↓ IL-1β [30]
cg26375855	0.64	4.61×10^-9^	4.30×10^-5^	19	54299976	*NLRP12*	Body	open sea	↑ IL-1β [31]
**Increases IL-1β and expressed in monocyte/macrophage lineage cells**									
cg22626579	-0.69	1.81×10^-7^	9.63×10^-4^	1	2038628	*PRKCZ*	5'UTR	island	↑ IL-1β [Supplementary Table 2 of 32, 33]
cg10314750	-0.74	2.98×10^-6^	1.03×10^-2^	3	9922257	*CIDEC*	TSS1500	open sea	↑ IL-1β [34, Figure 6E of 35]
cg16630982	-0.20	3.31×10^-6^	1.12×10^-2^	17	41277394	*BRCA1*	5'UTR	shore ↑ IL-1β [36, 37]
cg12182452	-0.35	1.14×10^-5^	3.12×10^-2^	17	41277730	*BRCA1*	TSS1500	shore

Chr: chromosome; CpG: cytosine-phosphate-guanine dinucleotide; FDR: false discovery rate.

^a^Methylation levels of gout-methylation levels of non-gout in monocytes after adjusting for age, sex, alcohol drinking, smoking status, smoking history (total pack-years) and cell fractions with CellDMC.

^b^Positions of the CpG sites in hg19.

^c^TSS200 and TSS1500 both belong to promoters [22].

^d^References about genes in relationship to IL-1β production and supporting its expression in monocyte/macrophage lineage cells.

**Figure 2:**
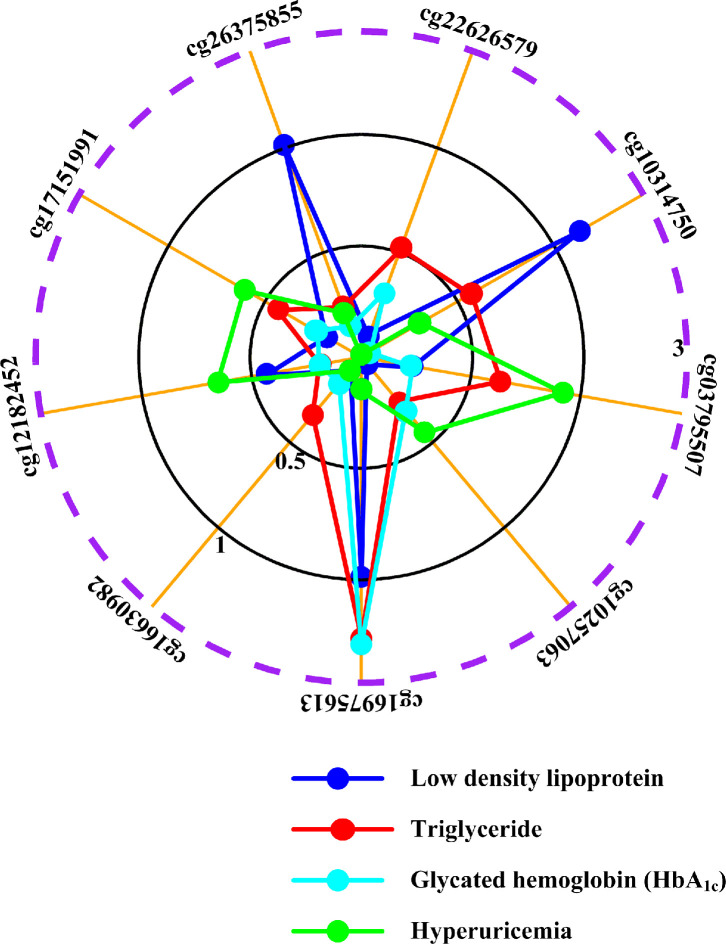
**Associations of surviving methylation sites with metabolic traits and hyperuricemia.** The graphical representation shows monocyte-specific associations between cg22626579, cg10314750, cg03795507, cg10257063, cg16975613, cg16630982, cg12182452, cg17151991, cg26375855 methylation and low density lipoprotein (blue line), triglyceride (red line), HbA_1c_ levels (cyan line) and hyperuricemia (green line). The radii mean minus log_10_*P* of monocyte-specific associations between cytosine-phosphate-guanine dinucleotide (CpG) sites and phenotypes. The dashed purple circle shows threshold value of *P*=0.05. For example, cg17151991 is not monocyte-specifically associated with low density lipoprotein (*P*=0.6650, minus log_10_*P*=0.18), triglyceride (*P*=0.3715, minus log_10_*P*=0.43), HbA_1c_ (*P*=0.5758, minus log_10_*P*=0.24) and hyperuricemia (*P*=0.2481, minus log_10_*P*=0.61). All results are adjusted for sex, age, alcohol drinking, smoking status, smoking history (total pack-years) and cell fractions with CellDMC (see supplementary methods).

Gout was associated with numerous comorbidities, including increased low-density lipoprotein levels, elevated triglyceride levels, and altered HbA_1c_ levels [2, 3]. To confirm that the monocyte-specific differential methylation we observed was specific to gout, we also examined monocyte-specific associations with metabolic traits (Supplementary Figure 1, Step IIe; Supplementary Table 5). Some of the initially identified CpG sites (cg18886702, cg00091098, cg22408430, cg13204333, cg14326053, cg10027934, cg01718853, cg13559233, cg03275949, cg07235456, cg25692425, cg11169286, cg05438708, and cg04810466) were not specific to gout (Supplementary Table 5). Meanwhile, of the CpG sites located in IL-1β-regulating genes, cg18886702 showed monocyte-specific associations with both low-density lipoprotein and HbA_1c_, cg00091098 showed monocyte-specific associations with low-density lipoprotein, cg22408430 manifested monocyte-specific associations with HbA_1c_, cg13204333, cg14326053, and cg10027934 displayed monocyte-specific associations with low-density lipoprotein (Supplementary Figure 4). Only nine CpG sites of the CpG sites located in IL-1β-regulating genes, (cg22626579, cg10314750, cg03795507, cg10257063, cg16975613, cg16630982, cg12182452, cg17151991, and cg26375855) that did not exhibit monocyte-specific associations with low-density lipoprotein, triglyceride, or HbA_1c_ levels survived metabolic trait analysis (Figure 2; Supplementary Figure 1, Step IIe). To augment metabolic trait analysis, we examined previous EWAS results from EWASdb (Supplementary Figure 1, Step IIf) [38]. None of these final nine CpG sites were associated with body mass index (Supplementary Table 6) according to EWASdb. A separate study also found no associations between DNA methylation and hypertension [39].

Furthermore, to differentiate between the hyperuricemia and gouty inflammation stages of gout [5–9], we examined monocyte-specific relationships between the final nine monocyte-specific differentially methylated CpG sites (cg22626579, cg10314750, cg03795507, cg10257063, cg16975613, cg16630982, cg12182452, cg17151991, cg26375855) and hyperuricemia (Supplementary Figure 1, Step IIg).

None of the final nine monocyte-specific differential methylation sites were associated with hyperuricemia (Figure 2), indicating that all nine sites were specifically involved in gouty inflammation alone. Similarly, a literature review did not identify any overlap between these nine loci and previously identified uric acid-associated loci (Supplementary Figure 1, Step IIh; Supplementary Table 7).

### Relationships between the final nine loci and gout were not confounded by genetic variants

Previous studies have demonstrated local correlations between genetic variants and DNA methylation levels (cis-meQTL) [40]. To determine whether genetic variants were involved in the monocyte-specific associations between CpG methylation and gout observed here, we conducted monocyte-specific cis-meQTL and genetic analyses of variants within 20,000 base pairs of the nine surviving monocyte-specific CpG sites; this strategy was similar to previous approaches (Supplementary Figure 1, Step IIIa-IIIb) [40].

**Figure 3:**
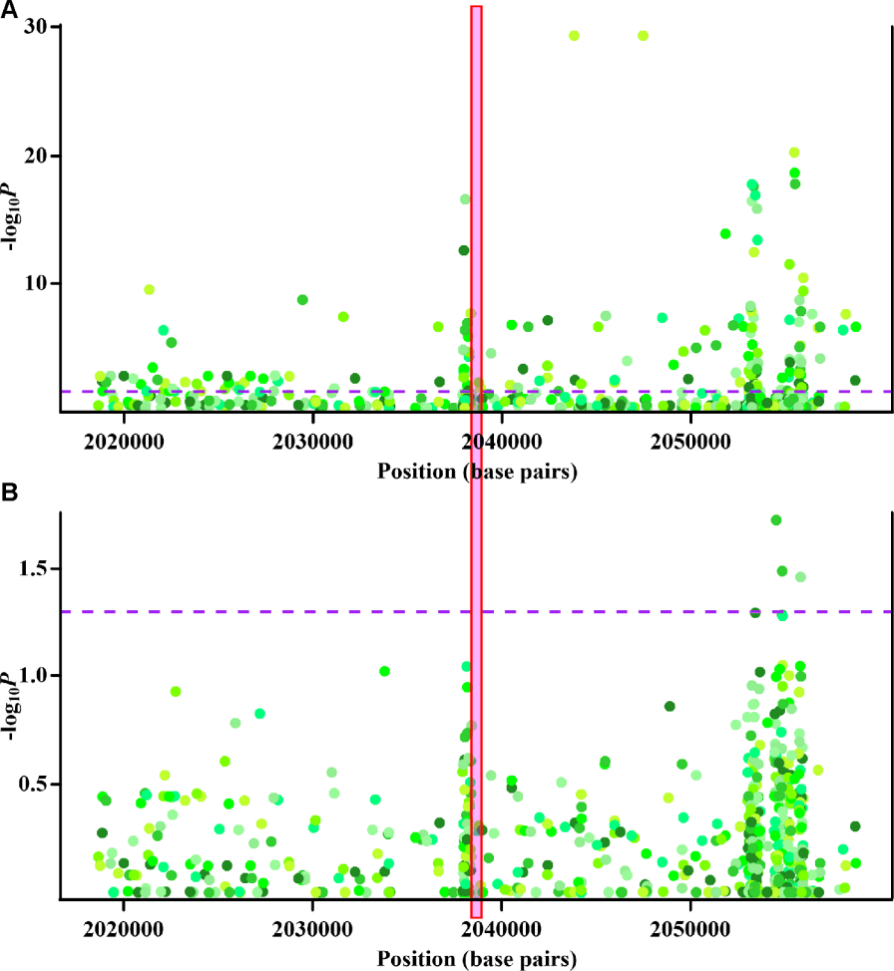
**Cis-methyl-quantitative trait locus (cis-meQTL) and genetic analysis of cg22626579.** (**A**) Regional association plots of monocyte-specific associations between nearby genetic variants and cg22626579 methylation. X-axis represents positions on respective chromosome. Y axis represents minus log_10_*P* of monocyte-specific associations between genetic variants and cg22626579 methylation. (**B**) Regional association plots of monocyte-specific associations between nearby genetic variants and gout. X-axis represents positions on respective chromosome. Y axis represents minus log_10_*P* of monocyte-specific associations between genetic variants and gout. Every point is one genetic variant colored with respective hue, with different colors implying different genetic variants. The dashed purple lines indicate the significance threshold (*P* = 0.05), and the red box highlights the location of cg22626579. Monocyte-specific associations of genetic variants with cg22626579 methylation and gout are analysed using multiple regression analysis, adjusting for sex, age, alcohol drinking, smoking status, smoking history (total pack-years) and cell fractions (see supplementary methods).

Associations between methylation at some of the initially identified the monocyte-specific CpG sites and gout were likely confounded by genetic variants (Supplementary Table 8). However, for cg22626579, as shown in Figure 3, although some nearby genetic variants displayed monocyte-specific associations with cg22626579 methylation (Figure 3A) or gout (Figure 3B), no single genetic variant was associated with both monocyte-specific cg22626579 methylation and gout. Thus, genetic variations did not underlie the observed monocyte-specific epigenetic associations between cg22626579 methylation and gout. Similar results were obtained for cg10314750, cg03795507, cg10257063, cg16975613, cg16630982, cg12182452, cg17151991 and cg26375855 (Supplementary Figures 5–12). Taken together, these results suggest that the relationships between monocyte-specific methylation at the final nine sites and gout were not confounded by genetic mediators, and all final nine CpG sites were therefore included in subsequent analysis.

### Less evidence of associations with gout from local co-methylated CpGs

Due to the high degree of spatial correlation in CpG methylation and the biological relevance of co-methylation [41–43], we examined whether methylation of CpG sites surrounding the final nine monocyte-specific differentially methylated loci were associated with gout (Supplementary Figure 1, Step IVa).

**Figure 4:**
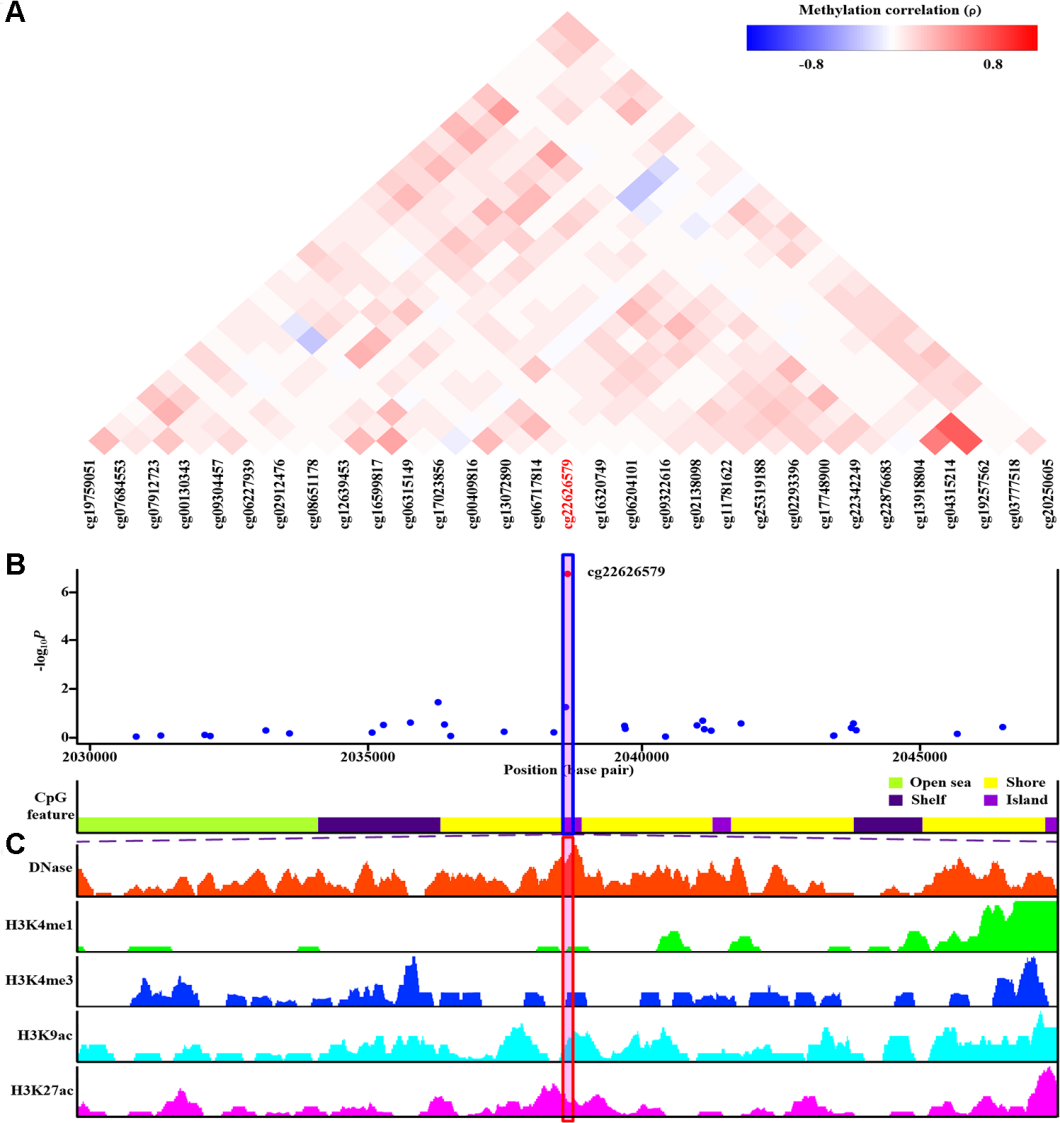
**Co-methylation analysis and functional annotation of cg22626579.** (**A**) Patterns of co-methylation at the cytosine-phosphate-guanine dinucleotide (CpG) sites surrounding cg22626579. (**B**) Monocyte-specific regional association results along with position of nearby CpG island (dark violet), CpG shore (yellow), CpG shelf (indigo) and CpG open sea (green yellow). cg22626579 (highlighted in shaded box) is located in CpG island. (**C**) Functional annotation of cg22626579. DNase hypersensitive sites derived by DNase-seq (DNase Track) and histone marks surrounding cg22626579 (H3K4me1, H3K4me3, H3K9ac and H3K27ac Tracks) in monocyte are shown. DNase hypersensitivity, H3K4me1, H3K4me3, H3K9ac and H3K27ac histone marks are associated with active regulatory elements.

Of the final nine sites that passed monocyte-specific cis-meQTL/genetic analyses (cg22626579, cg10314750, cg03795507, cg10257063, cg16975613, cg16630982, cg12182452, cg17151991 and cg26375855), corresponding MethylationEPIC BeadChip array CpG probes located in the nearby region were examined (Figure 4A, Supplementary Figures 13A–20A). As shown in Figure 4A, none of the nearby CpG sites demonstrated evidence of co-methylation with cg22626579 (ρ≥0.8) [44]. Furthermore, none of the monocyte-specific associations between these nearby CpG sites and gout were nearly as strong as the association for cg22626579 (Figure 4B). Similar results were obtained for cg10314750 (Supplementary Figure 13A, 13B), cg03795507 (Supplementary Figure 14A, 14B), cg10257063 (Supplementary Figure 15A, 15B), cg16975613 (Supplementary Figure 16A, 16B), cg16630982 (Supplementary Figure 17A, 17B), cg12182452 (Supplementary Figure 18A, 18B), cg17151991 (Supplementary Figure 19A, 19B), and cg26375855 (Supplementary Figure 20A, 20B). Furthermore, there was no evidence of co-methylation between cg16630982 and cg12182452 (ρ=0.51) (Supplementary Figure 17A), suggesting that monocyte-specific associations observed for those two CpG sites were independent.

We also completed co-methylation analysis for the six methylation sites located in IL-1β-regulating genes that did not pass metabolic trait analysis (Supplementary Table 3; Supplementary Figure 21A–26B) and for the remaining 43 CpG sites located in genes that did not directly regulate IL-1β (Supplementary Table 4; Supplementary Figure 27A–69B). Although cg00748492 displayed co-methylation with cg01904227 (ρ=0.81) and cg26544725 (ρ=0.81) (Supplementary Figure 51A), neither of the latter two sites had monocyte-specific associations with gout as strong as that of cg00748492 (Supplementary Figure 51B). These results suggest that the 58 monocyte-specific methylation sites identified here were independent signals.

### Functional annotation of CpG loci in monocyte regulatory elements

To assess whether the final nine monocyte-specific differentially methylated CpG sites that passed monocyte-specific cis-meQTL and genetic analyses had functional potential, we examined monocyte epigenetic data for the area surrounding each site in monocytes using the WashU epigenome browser (Supplementary Figure 1, Step IVb). We chose DNase, H3K4me1, H3K4me3, H3K9ac, and H3K27ac as annotation histone marks. DNase-seq is a well-established method for identifying nucleosome-depleted regions that include active regulatory elements [45]. We also evaluated CpG sites with respect to post-translational histone modifications H3K4me1, H3K4me3, H3K9ac, and H3K27ac, which are associated with active regulatory regions [46]. The WashU epigenome browser (hg19) was queried to create separate epigenetic tracks for regions containing differentially methylated CpG sites (Figure 4C, Supplementary Figures13C–20C).

DNase-seq results indicated that cg22626579 was located in a DNase hypersensitivity site in monocytes (Figure 4C). Furthermore, cg22626579 was located in a transcriptional regulatory region containing multiple histone marks characteristic of active regulatory elements (H3K4me1, H3K4me3, H3K9ac and H3K27ac) in monocytes (Figure 4C) [46]. Together, these features suggested that cg22626579 might have regulatory potential in monocytes. Similar results were obtained for the remaining eight monocyte-specific differentially methylated CpG sites (cg10314750, cg03795507, cg10257063, cg16975613, cg16630982, cg12182452, cg17151991, and cg26375855), each of which overlapped with DNase hypersensitivity sites and the histone marks (H3K4me1, H3K4me3, H3K9ac and H3K27ac) indicative of active regulatory elements in monocytes (Supplementary Figures 13C, 20C). Taken together, these findings suggest that the final nine monocyte-specific differentially methylated CpG sites might have regulatory potential in monocytes.

Of the additional six methylation sites located in IL-1β-regulating genes that failed metabolic trait analysis (Supplementary Table 3) as well as the remaining 43 CpG sites located in genes that did not directly regulate IL-1β (Supplementary Table 4), all showed some regulatory potential, as evidenced by overlap with various combinations of DNase hypersensitivity site and histone marks indicative of active regulatory region in monocytes (Supplementary Figure 21C, 69C).

### Transcription factor mapping for the final CpG loci

Although DNA methylation changes may not directly impact cellular processes, differential DNA methylation coordinates transcription factors that exert downstream effects [47]. To explore how cell lineage-specific methylation might affect mechanisms underlying gout, we used MoLoTool and ReMap to identify associated transcription factors (Supplementary Figure 1, Step Va-Vb) [48, 49]. No associated transcription factors were identified for cg10257063 and cg26375855 by ReMap; otherwise, associated transcription factors were identified for all nine sites by both tools (Supplementary Tables 9–24, Supplementary Figure 70).

### Associations between methylation at the final nine CpG loci and clinical characteristics

To evaluate whether the final nine epigenetic loci had clinically relevant associations with phenotypic variations among gout patients in addition to disease susceptibility, we examined associations between methylation at the final nine CpG loci and clinical characteristics in gout patients (Supplementary Figure 1, Step Vc). Interesting, methylation levels of *PRKCZ* and *STK11* were lower for individuals with more extensive family histories of gout in first-degree relatives (Supplementary Figure 71). These results were consistent with the associations between *PRKCZ* and *STK11* hypomethylation and development of gout (Table 2). Family history of gout is associated with other markers of severity such as polyarticular gout and tophi formation [50, 51]. These observations suggest that *PRKCZ* and *STK11* hypomethylation could be associated with more severe gout phenotypes in addition to gout susceptibility.

## DISCUSSION

Different cell lineages displayed distinct methylation alterations in gout. However, the IL-1 signaling pathway was identified as the pathway most affected by altered methylation in multiple cell lineages. Aberrant DNA methylation that exhibited monocyte-specific associations with gout was observed at nine sites (cg22626579, cg10314750, cg03795507, cg10257063, cg16975613, cg16630982, cg12182452, cg17151991, and cg26375855) that mapped to eight genes (*PRKCZ*, *CIDEC*, *VDAC1*, *CPT1A*, *BIRC2*, *BRCA1*, *STK11* and *NLRP12*); however, methylation at these sites was not associated with gout comorbidities or hyperuricemia (Figure 2). These observations suggest that these sites were specifically associated with gouty inflammation, rather than hyperuricemia. Furthermore, all nine CpG sites survived monocyte-specific cis-meQTL and genetic analyses, excluding the possibility of confounding genetic factors (Figure 3, Supplementary Figures 5–12). Functional annotation revealed that each site was located in a region of open chromatin structure containing histone markers indicative of active regulation, supporting their regulatory potential in monocytes (Figure 4C, Supplementary Figure 13C, 20C). Transcription factor mapping also identified several transcription factors affected by the methylation sites that might exert effects in gout (Supplementary Tables 9–24, Supplementary Figure 70). Finally, decreased *PRKCZ* and *STK11* methylation were associated with familial clustering of gout (Supplementary Figure 71).

In this study, there was little overlap in differential methylation patterns between different cell lineages (Supplementary Figure 2). Similarly, previous studies have reported limited overlap of differential methylation between distinct cell lineages [52]. Whether this observation applies to other diseases as well remains unknown. However, this result suggested that different regulatory mechanisms of DNA methylation operated in different cell subsets, resulting in distinct methylation landscapes in different cell lineages. Improved understanding of these cell lineage-specific methylation regulatory mechanisms might help identify cell type-specific therapies for various diseases that might minimize unnecessary side effects.

Multiple pathways besides IL-1 signaling that were affected by methylation, such as IL-10 signaling (Figure 1B, 1D) and ubiquinol-10 biosynthesis (Figure 1A, 1C), were shared by several cell types. Interestingly, most were also related to IL-1β production and gouty inflammation (Supplementary Table 25). The precise roles these pathways play in the development of gout remain unknown and should be examined in future investigations.

**Figure 5:**
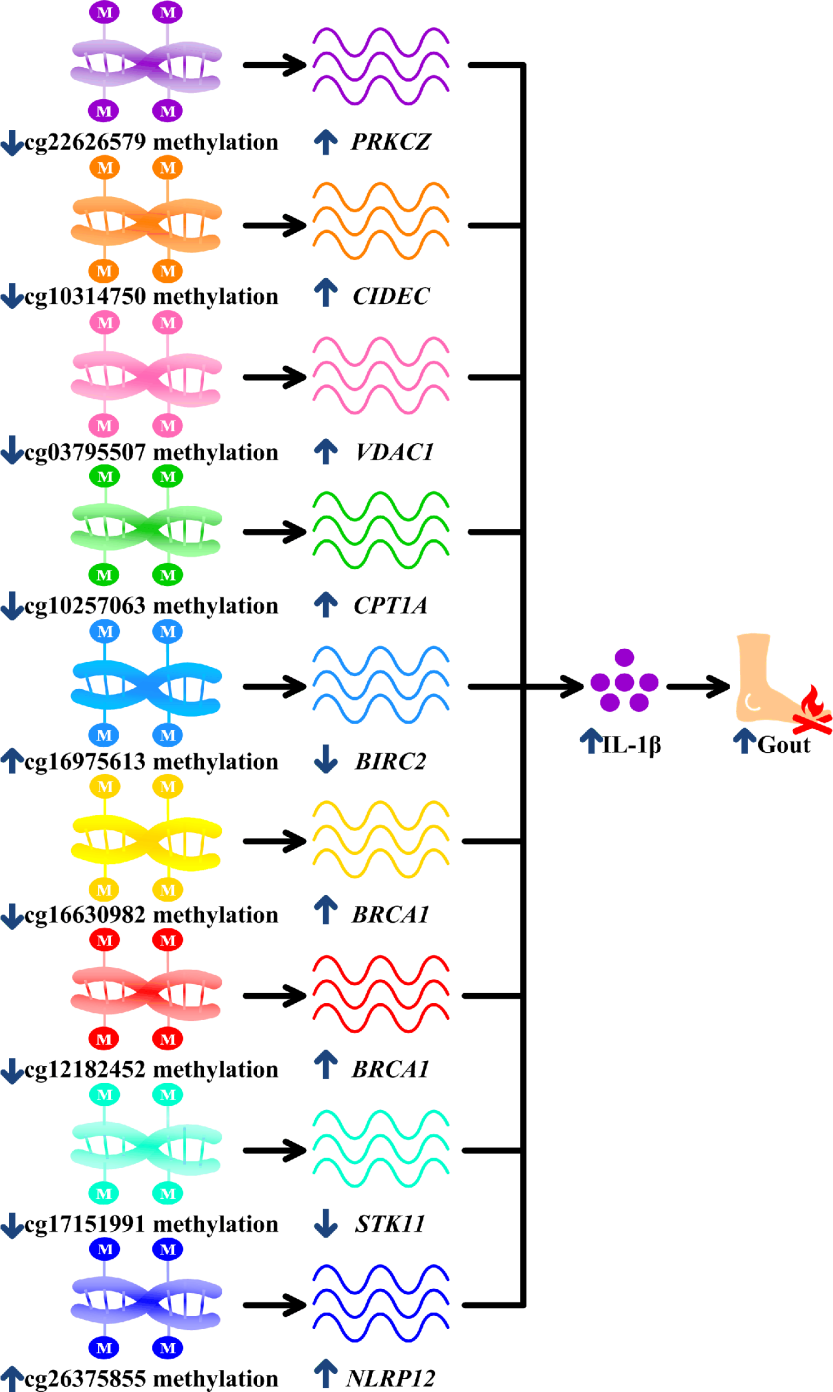
**Potential mechanisms linking surviving methylation sites with gouty inflammation.** cg22626579 hypomethylation in gout increases *PRKCZ,* cg10314750 hypomethylation in gout increases *CIDEC*, cg03795507 hypomethylation in gout increases *VDAC1*, hypomethylated cg10257063 in gout increases *CPT1A*, cg16975613 hypermethylation in gout decreases *BIRC2*, cg16630982 and cg12182452 hypomethylation in gout increases *BRCA1,* cg17151991 hypomethylation in gout decreases *STK11* and hypermethylated cg26375855 in gout increases NL*RP12*. All these culminate in increased interleukin-1β (IL-1β) production, promoting gouty inflammation.

The final nine surviving monocyte-specific differentially methylated CpG sites were mapped to eight genes (*PRKCZ*, *CIDEC*, *VDAC1*, *CPT1A*, *BIRC2*, *BRCA1*, *STK11,* and *NLRP12*) that have not previously been studied in the field of gout genetics. *PRKCZ* is expressed in macrophages and increases IL-1β release [Supplementary Table 2 of 32, 33]. 5'UTR hypomethylation is associated with gene upregulation [53], and hypomethylated *PRKCZ* might therefore increase *PRKCZ* expression, subsequently increasing IL-1β production in macrophages and facilitating gout (Table 2, Figure 5).

*CIDEC* is expressed in macrophages and increases the release of IL-1β [34, Figure 6E of 35]. Promoter hypomethylation is associated with gene upregulation [54, 55]. Therefore, *CIDEC* hypomethylation might increase IL-1β, which is the central driver in gouty inflammation (Table 2, Figure 5) [9]. *VDAC1* also stimulates IL-1β expression in macrophages [26], and the hypomethylated *VDAC1* promoter may promote *VDAC1* expression [54, 55] and subsequent IL-1β expression in macrophages, thus perpetuating gouty inflammation (Table 2, Figure 5). *CPT1A* is also required for IL-1β production in macrophages and may be upregulated in response to 5'UTR hypomethylation [27, 53]. The *CPT1A* hypomethylation observed here therefore likely promotes gout by increasing *CPT1A* expression and IL-1β production in macrophages (Table 2, Figure 5).

Previous studies demonstrated that *BIRC2*, also called *cIAP1* [28], downregulates IL-1β in macrophages [29]. Gene is downregulated in association with promoter hypermethylation [54, 55], Here, hypermethylation of *BIRC2* likely suppressed *BIRC2* expression and increased IL-1β production, exacerbating gout (Table 2, Figure 5). *BRCA1* is also expressed in macrophages and can increase IL-1β expression [36, 37]. Consequently, hypomethylated promoter and 5'UTR of *BRCA1* increased *BRCA1* expression and IL-1β secretion, thus promoting gouty inflammation (Table 2, Figure 5) [36, 37, 53–55]. *STK11* inhibits IL-1β release in macrophages [30]. Hypomethylation of *STK11* gene body likely suppressed *STK11* expression [56, 57], which in turn increased IL-1β levels and magnified gouty inflammation (Table 2, Figure 5) [30, 56, 57]. *NLRP12* also upregulated IL-1β in macrophages [31] and hypermethylation of *NLRP12* gene body probably increased *NLRP12* expression [56, 57] and IL-1β release, thereby promoting gouty inflammation (Table 2, Figure 5) [31, 56, 57].

Fifteen of the monocyte-specific differentially methylated CpG sites identified here were located in genes that affect IL-1β levels according to previous reports (Table 2, Supplementary Table 3) while 43 sites were located in genes with no reported direct effects on IL-1β (Supplementary Table 4). Of these 43 methylation sites, only cg19824059, cg08797047, and cg11169286 were associated with hyperuricemia (Supplementary Table 26). However, most of the 43 sites displayed potential associations with inflammation according to current evidence (Supplementary Table 26). Therefore, although the associations of cg19824059, cg08797047, and cg11169286 methylation with gout could be partly attributed to hyperuricemia, gouty inflammation, rather than hyperuricemia might provide the link between most of the 43 remaining CpG sites and gout. Deeper analysis of these 43 sites might help identify additional mechanisms underlying gout pathogenesis.

Some of the factors identified during transcription factor mapping in this study (*AHR*, *CEBPA*, *CEBPB*, *EGR1*, *HIF1A*, *KLF9*, *MED1*, *NFE2L1*, *PPARG*, *RELA*(*p65*), *SPI1*(*PU.1*), *STAT1*, and *VDR*) have been reported to increase or decrease IL-1β levels in monocytes or macrophages in previous studies (Supplementary Figure 70). Additionally, *AHR*, *BRD4,* and *PPARG* are known to facilitate or inhibit gouty inflammation (Supplementary Figure 70). Together, these findings support potential roles for these transcription factors in gouty inflammation. The results of transcription factor mapping could inform further studies to clarify mechanism through which these CpG sites facilitate gouty inflammation.

We also explored whether the nine epigenetic variations associated with gout development (Table 2) were also associated with clinical phenotypes in gout patients. The results indicated that hypomethylation in *PRKCZ* and *STK11* was associated with familial clustering of gout (Supplementary Figure 71). Familial clustering of gout is associated with polyarticular manifestation and presence of tophi [50, 51], both of which are indicative of severe gout. These findings suggest that *PRKCZ* and *STK11* hypomethylation might impact not only disease susceptibility but might also impact family histories and clinical phenotypes in gout patients.

The etiology of monocyte-specific differential methylation of *PRKCZ*, *CIDEC*, *VDAC1*, *CPT1A*, *BIRC2*, *BRCA1*, *STK11*, and *NLRP12* in gout remains unknown. Given that both genetic and environmental factors play roles in gout [12] and because environmental exposure is crucial in shaping the epigenome landscape [15], environmental factors may underlie this differential methylation. For example, previous studies found that the risk of gout was inversely associated with consumption of coffee and omega-3 fatty acids, both of which decreased DNA methylation [58–61]. Conversely, legumes, which increased methylation, reduced the risk of gout [62, 63]. Additional studies are needed to identify other environmental factors that might impact gout by increasing or decreasing DNA methylation.

Irrespective of the underlying causes, these methylation changes could facilitate gouty inflammation (Figure 5). Several lines of evidence further showed molecules targeting *PRKCZ*, *CIDEC*, *VDAC1*, *CPT1A*, *BIRC2*, *BRCA1* and *STK11* exerted corresponding effects on gouty inflammation (Supplementary Table 27). Thus, findings of this study could represent novel therapeutic targets in gout.

One of the main strengths of this study is the inclusion of large number of subjects from the general population of Taiwan, which increases its applicability to clinical practice. Furthermore, we adjusted for the following covariates using CellDMC: age, sex, alcohol drinking, smoking status, smoking history (total pack-years), and cell fractions [20]. All of these covariates can affect DNA methylation [64–66], and previous epigenetic studies have not adequately controlled for their effects [67]. Furthermore, the cell lineage-specific nature of the methylation studies we examined here allowed us to draw novel conclusion. Because different cell types participate in different aspects of gout [68], identification of a specific cell type showing epigenetic alterations is crucial in understanding the impact of such alterations on diverse biological function, identifying causal relationships between methylation and phenotype, and establishing valuable clinical biomarkers and therapeutic targets. In addition, previous studies demonstrated that genetic factors contributing to disease susceptibility often function in a cell type-specific manner [69]. Cell type-specific functional investigations like ours might therefore ultimately help connect epigenetic variations identified via EWAS to molecular mechanisms and drug discovery. Finally, drugs that act on cell type-specific targets are more likely to gain regulatory approval and enter clinical use [70]. Cell lineage-specific studies might therefore increase drug discovery successful rates.

We used a deconvolution algorithm to infer cell type-specific differential methylation from tissue-level methylation results instead of directly measuring of methylation in various isolated cell lineages. Several advantages are associated with tissue-level bulk profiling compared to cell-type specific profiling, in which low recovery rates per specimen lead to large volume requirements for each individual. It is difficult to obtain samples of sufficient volume that contain various cell lineages in large enough quantities to conduct research on the scale of this study. Additionally, samples for lineage-specific analysis performed in isolated cell lineages must be handled in a manner that preserves cellular membranes and epitopes for cell-sorting. The steps necessary to preserve these structures differ between cell lineages, making such procedures impractical for large-scale cohort investigations like the present study that simultaneously targeting multiple cell lineages. Finally, the high costs of additional collection, sorting, enrichment, and purification also limit available sample sizes. Most recent epigenetic studies have therefore used whole-available tissue samples for EWAS rather than isolated specific cell subsets [71, 72].

In conclusion, this cell lineage-specific epigenetic and genetic study of gout revealed distinct methylation landscapes across various cell lineages. IL-1 signaling emerged as the most important associated molecular mechanism in pathway analysis and network construction. Further cis-meQTL and genetic analyses revealed evidence of monocyte-specific aberrations in methylation of *PRKCZ*, *CIDEC*, *VDAC1*, *CPT1A*, *BIRC2*, *BRCA1*, *STK11,* and *NLRP12* in gout. All of these genes were associated specifically with gouty inflammation rather than hyperuricemia. Moreover, *PRKCZ* and *STK11* methylation was also associated with familial clustering of gout. Regardless of the reasons of differential methylation, these methylation changes promote gouty inflammation. However, the processes by which environmental factors promote gout by increasing or decreasing DNA methylation in each cell type-specific manner remain unknown. Multi-omic studies are required to improve our understanding of how DNA methylation interacts with other biological networks to impact gout. Longitudinal studies might help pinpoint DNA methylation markers that predict severe gout phenotypes. Additional studies and clinical trials are needed to determine whether the molecular markers might serve as effective treatments for gout.

## MATERIALS AND METHODS

### Ethical statement

Informed consent was obtained from all enrollees. All experimental protocols were performed in compliance with the Declaration of Helsinki and according to national and international guidelines and were approved by the Tian-Sheng Memorial Hospital Institutional Review Board (TSMHIRB 17-122-B).

### Study participants

All study participants of this study were recruited from the Taiwan Biobank, a prospective national cohort aiming to promote biomedical research in Taiwan [73]. This cohort was recruited from the general Taiwanese population and has been examined in several epigenetic and genetic studies, including studies on gout [12, 74, 75]. All Taiwan Biobank patients who provided informed consent for peripheral blood leukocyte DNA methylation measurement and DNA sequencing were included in the study. All steps were performed in compliance with relevant guidelines and regulations.

Sixty-nine patients with self-reported gout and 1,455 patients who self-reported the absence of gout underwent methylation array and whole genome sequencing analysis. For patients with gout, the number of first-degree relatives who also had gout were obtained from Taiwan Biobank. All participants self-identified as Han Chinese. Previous genetic studies also utilized self-reported gout [12, 76, 77] and demonstrated that self-reporting of physician-diagnosed gout resulted in high sensitivity and precision [78].

### DNA bisulfite conversion and methylation quantification

Peripheral blood was collected from enrolled participants into sodium citrate tubes. DNA was isolated using the Chemagic™ Prime™ instrument, an automated chemical extraction machine that uses magnetized rods to separate nucleic acids from solutions. A Fragment Analyzer (Agilent) was used to measure DNA length, and the 260/280 optical density (OD) ratio was used to assess DNA purity. Samples with an OD 260/280 ratio of 1.6~2.0 were considered pure. DNA samples were subjected to sodium bisulfite treatment using the EZ DNA Methylation Kit (Zymo Research, CA, USA). DNA methylation at each cytosine-phosphate-guanine dinucleotide (CpG) site was quantified with the Infinium® MethylationEPIC BeadChip array (Illumina Inc) which covered 850,000 methylation sites [79]. Samples were randomized on the MethylationEPIC BeadChip to avoid batch effects. The assay was performed according to the manufacturer’s standard protocol.

### Whole genome sequencing

Genomic DNA was isolated from peripheral blood and purified using standard protocols. Whole genome sequencing was performed using the HiSeq platform (Illumina Inc). On average, 8.6 Gb of mappable sequence data per individual was obtained. Isaac version 01.13.10.21 was employed to map DNA sequence reads to the hg19 reference genome. A minimum coverage of 30× was confirmed for regions of interest. Isaac Variant Caller version 2.0.17, Grouper version 1.4.2 and CNVseg version 2.2.4 were used for variant calling. Alleles were annotated with ANNOVAR version 2014Jul14. Complete assemblies of the regions of interest were obtained for all samples.

### Marker classification

In the MethylationEPIC BeadChip array, CpG markers were categorized based on chromosome location and the feature category of the gene region according to UCSC annotation (TSS200, TSS1500, 5'UTR, first exon, Body, 3'UTR, intergenic). In this classification scheme, the TSS200 category refers to the region 0~200 bases upstream from the transcriptional start site (TSS), TSS1500 category refers to the region 201~1,500 bases upstream of TSS [80], 5'UTR refers to the region between the TSS and the start site (ATG). CpGs within the first exon of a gene are included in the first exon category, CpGs downstream from the first exon and before the stop codon, including intronic regions, are included in the gene body category, and CpGs downstream from the stop codon and before the poly A signal are included in the 3'UTR category. Finally, CpGs that do not fit into any of the previous categories are annotated as intergenic.

### Cell lineage-specific genome-wide methylation analysis

Minfi version 1.18.2 was used to import raw methylation data and generate methylation β-values for subsequent analyses [81]. Methylation results from the MethylationEPIC BeadChip array were processed and analyzed according to previously reported approaches (Supplementary Figure 1, Step I) [64]. Quality control at the probe level was performed by computing a detection *P* value relative to control probes. Probes without significant detection (*P* > 0.05) in more than 5% of the samples were excluded (Supplementary Figure 1, Step Ia) [64]. Sex chromosome CpG sites were also excluded because X chromosomes were inactivated by methylation in females (Supplementary Figure 1, Step Ib) [82]. Probes with single nucleotide polymorphisms (SNPs) at the CpG site (minor allele frequency ≥ 5%), at the single base extension (minor allele frequency ≥ 5%), and at probes (minor allele frequency ≥ 5%) (Supplementary Figure 1, Step Ic) were also eliminated [64]. Previously identified cross-reactive probes in the MethylationEPIC BeadChip were excluded as well (Supplementary Figure 1, Step Id) [83]. Methylation intensity levels were functionally normalized after subdividing by probe type, sub-type, and color channel (Supplementary Figure 1, Step Ie), and beta-normalized methylation levels were then calculated based on these normalized intensity levels [64]. Qualified probes were examined in subsequent analyses.

To estimate cell-type fractions, we leveraged the previously validated EpiDISH procedure (Supplementary Figure 1, Step If) [20]. Briefly, the EpiDISH procedure uses a DNA methylation reference matrix defined by 333 immune-cell-subtype-specific differentially methylated CpG sites and seven immune cell subtypes (monocytes, eosinophils, neutrophils, NK cells, B cells, CD4+ T cells, and CD8+ T cells) to estimate underlying immune cell subtype fractions.

CellDMC was used to identify cell lineage-specific differentially methylated CpG sites in gout (Supplementary Figure 1, Step Ig) [20]. Adjustments were made for sex, age, alcohol drinking, smoking history (total pack-years), smoking status and cell fractions to avoid the confounding effects of these factors [64–66, 84]. Participants were categorized as non-smokers if they had never smoked or have not smoked continuously for at least six months. Former smokers included those who continuously smoked for at least six months but were currently not smoking. Participants who have continuously smoked for at least six months and were currently smoking were classified as current smokers. Participants were considered non-drinkers if they had never drunk or drank less than 150 cc of alcohol per week continuously for six months. Patients who abstained from alcohol for more than six months were regarded as former drinkers while current drinkers were those who drank at least 150 cc of alcohol per week continuously for six months, according to Taiwan Biobank questionnaires [74]. CpG sites showing differential methylation between gout and non-gout patients and the cell lineages that drove those effects were identified. False discovery rates less than 0.05 were considered significant [85].

### Overlap of differential methylation in different cell lineages

To examine whether differential methylation patterns were similar between different cell lineages, we used the definition of Jaccard Index to calculate overlap rates (Supplementary Figure 1, Step IIa) [86]. At the CpG site level, the overlap rate of differential methylation was the ratio of the number of CpG sites with differential methylation in both cell lineages over the number of CpG sites with differential methylation in one or both cell lineages. At the gene level, the overlap rate of differential methylation was the ratio of the number of genes containing differentially methylated CpG sites in both cell lineages over the number of genes with differentially methylated CpG sites in one or both cell lineages. Differential methylation between different cell lineages was considered similar if the overlap rate was ≥ 0.5, the same threshold used in a previous study [23].

### Ingenuity Pathway Analysis (IPA)

To identify the common functional characteristics of differentially methylated genes in monocytes, eosinophils, neutrophils, NK cells, B cells, CD4+ T cells, and CD8+ T cells, we used IPA (Qiagen) to determine the relative enrichment of genes in target pathways using right-tailed Fisher’s exact tests (Supplementary Figure 1, Step IIb). Genes containing differentially methylated CpG sites in each cell lineage were uploaded into IPA to perform enrichment analysis. The ingenuity knowledge base (genes only) with direct and indirect relationships and only molecules and/or relationships that had been experimentally observed or predicted in human were considered. Core analysis of the differentially methylated genes in each cell lineage was performed and results were downloaded. The top 20 pathways with the lowest *P* values in each lineages were plotted.

### Protein-protein interaction (PPI) network construction

To explore functional relationships between differentially methylated genes, regulatory networks were constructed for genes containing differentially methylated CpG sites using NetworkAnalyst (Supplementary Figure 1, Step IIc) [87] and were visualized with Cytoscape [87]. NetworkAnalyst integrates machine learning and Walktrap algorithms and uses protein-protein interaction data from the IMEx Interactome database to perform topology analysis that examines the overall network structure to identify important genes (hubs) that act as critical players in biological networks [87].

### Identify monocyte-specific CpG sites associated with gouty inflammation

IPA identified IL-1 signaling as a key pathway (Figure 1), and interleukin-1β (IL-1β) is synthesized primarily by monocytes and macrophages [24]. Additionally, most of the hub genes in the gout network were IL-1β-regulating genes (Supplementary Figure 3). We therefore focused on monocyte-specific differentially methylated genes that upregulated or downregulated IL-1β expression. CpG sites which also displayed differential methylation in other cell lineages were excluded to prevent those associations from potentially confounding the associations between CpG methylation in monocytes and gout. We also performed a literature review to elucidate the biological functions of these monocyte-specific differentially methylated genes. CpG sites annotated to genes that regulated IL-1β, the most crucial player in gouty inflammation, were included in subsequent analysis (Supplementary Figure 1, Step IId) [9].

Gout is accompanied by numerous comorbidities, including increased low density lipoprotein levels, elevated triglyceride levels, and altered glycated hemoglobin (HbA_1c_) levels [2, 3]. To confirm the specificity of the identified monocyte-specific differential methylation in gouty inflammation, we tested monocyte-specific associations between DNA methylation and low-density lipoprotein, triglyceride, HbA_1c_ and hyperuricemia, adjusting for sex, age, smoking history (total pack-years), smoking status, alcohol consumption and cell fractions using CellDMC [20] (Supplementary Figure 1, Step IIe, Step IIg).

### Comparison with past epigenome-wide association study (EWAS) results

To augment the results of our analyses, we obtained CpG sites associated with hypertension and body mass index from EWASdb on April 14, 2020 (Supplementary Figure 1, Step IIf) [38]. EWASdb is a part of “The EWAS Project” which seeks to store epigenetic association results for DNA methylation from various studies; it currently includes over 1,300 sets of EWAS results associated with more than 300 diseases/phenotypes. CpG sites associated with body mass index were downloaded from EWASdb. No CpG sites were associated with hypertension in EWASdb. Similarly, no evidence of associations between DNA methylation and hypertension was found in another recent study [39].

### Comparison with past genome-wide association study (GWAS) results

To compare methylation results with past uric acid-associated risk variants, we performed a literature search of PubMed on October 1, 2019 and retrieved studies describing relationships between genetic loci and uric acid levels using the search terms “GWAS” and “uric acid”. Studies were included if they were written in English and contained data on loci associated with uric acid levels. No limitations were placed on patient’s ethnicity. Review articles and case reports were excluded. Selected articles were screened for potential uric acid-associated loci. Only genome-wide significant loci (*P* < 5×10^-8^) related to uric acid levels were considered; CpG sites located in uric acid-associated genes were discarded (Supplementary Figure 1, Step IIh).

### Monocyte-specific cis-meQTL and genetic analysis

To exclude monocyte-specific epigenetic associations between gout and CpG site methylation that were confounded by genetic factors, we conducted monocyte-specific cis-meQTL and genetic analyses of variants within 20,000 base pairs of CpG; this was consistent with previous studies (Supplementary Figure 1, Step III) [40]. In short, we first searched for genetic variants within 20,000 base pairs of CpG sites of interest using the UCSC Genome Browser (https://genome.ucsc.edu/). Corresponding genetic variant genotyping results were obtained from whole genome sequencing. To infer the cell type specificity of genetic variants, we applied a statistical model that tested for an interaction effect between the genetic variant dosage and the proportion of various cell types, adjusting for sex, age, smoking history (total pack-years), smoking status, and alcohol consumption. In other words, monocyte-specific associations between genetic variants and CpG methylation and gout were identified by introducing additional variant×cell proportion interaction terms in the regression model between genetic variants and CpG methylation/gout and examine *P* values for every genetic variant for the interaction terms associated with each genetic variant; this was analogous to past approaches [88–90]. Genetic variants displaying concomitant monocyte-specific associations with CpG methylation (Supplementary Figure 1, Step IIIa) and gout (Supplementary Figure 1, Step IIIb) were considered likely to confound monocyte-specific associations between CpG methylation and gout.

### Regional visualization of co-methylation patterns surrounding surviving CpG sites

After identifying CpG sites whose methylation was associated with gouty inflammation in a monocyte-specific manner, we applied coMET to estimate DNA methylation correlation between CpG sites [43]. A regional plot of monocyte-specific epigenetic-phenotype association results, estimated DNA methylation correlations between CpG sites (co-methylation) and genomic context was generated for visualization of the results (Supplementary Figure 1, Step IVa).

### Monocyte-specific functional annotation of surviving CpG sites

To gain further insight about the biological relevance of differentially methylated CpG sites, the WashU epigenome browser was used to visualize chromatin modifications and histone acetylation patterns around CpG sites of interest in monocytes [91]. DNase footprinting patterns in the region identified in our association analysis were examined since DNase hypersensitivity implies an open chromatin structure, which is typical of active regulatory elements [45]. CpG regions were also aligned against ChIP-seq data for acetylated and methylated lysine variants of histone H3, which are also indicative of the histone code of active regulatory regions [46]. The WashU epigenome browser was also used to visualize DNase and histone markers located near monocyte-specific differentially methylated sites in monocytes (Supplementary Figure 1, Step IVb).

### Transcription factor mapping of surviving CpG sites

Differential DNA methylation contributes to transcriptional dysregulation by altering transcription factor binding [47]. To gain insight into which transcription factors might be involved, we used MoLoTool in conjunction with ReMap (Supplementary Figure 1, Step Va-Vb) [48, 49]. For all the monocyte-specific CpG sites surviving the above analysis, we downloaded the DNA sequence of the region around each monocyte-specific CpG site from Ensembl BioMart (GRCh38.p13 assembly) and used MoLoTool to identify transcription factors that bind to the CpG sites (Supplementary Figure 1, Step Va) [48, 92]. The threshold *P*-value was set at the recommended default of 0.0005 [48]. We also used ReMap, which includes high-quality transcription factor binding region data from a large-scale integrative analysis of thousands of ChIP-seq and ChIP-exo datasets [49]. We retrieved transcription factor binding sites in monocytes/macrophages (Supplementary Table 1) from ReMap database and overlapped transcription factor binding sites of monocytes/macrophages with surviving CpG sites from this study (Supplementary Figure 1, Step Vb). Transcription factors obtained from MoLoTool and ReMap were plotted and summarized.

### Associations between monocyte-specific CpG site methylation and clinical characteristics

Because all samples used in this study were obtained from Taiwan Biobank, detailed clinical characteristics were not available for further analysis. However, numbers of first-degree relatives with gout were available for gout patients. We therefore examined monocyte-specific associations between DNA methylation at the surviving CpG sites and the number of first-degree relatives with gout, adjusting for sex, age, smoking history (total pack-years), smoking status, alcohol consumption and cell fractions using CellDMC [20] (Supplementary Figure 1, Step Vc).

## Supplementary Material

Supplementary Figures

Supplementary Tables
