# Supplementary material for: Cell lineage-specific methylome and genome alterations in gout
Source: Aging (Albany NY). 2021 Jan 20;13(3):3843–65. doi: 10.18632/aging.202353 (PMC7906142; doi:10.18632/aging.202353)
Supplement: Supplementary Figures [file aging-13-202353-s001.docx]

Supplementary Figures

**Supplementary Figure 1. Flowchart of the analysis pipeline.** Methylation results first undergo various quality control steps to identify cell lineage-specific differentially methylated cytosine-phosphate-guanine dinucleotide (CpG) sites with CellDMC, adjusting for sex, age, alcohol drinking, smoking status, smoking history (total pack-years) and cell fractions (Step 1). After examining overlap of differential methylation between different cell lineages (Step IIa), Ingenuity Pathway Analysis (Step IIb) and protein-protein interaction network construction (Step IIc), monocyte-specific CpG sites mapping to genes regulating interleukin-1β (IL-1β) are singled out (Step IId). Furthermore, metabolic traits-related CpG sites are eliminated (Step IIe), CpG sites associated with metabolic traits in past epigenome-wide association studies (EWAS) are discarded (Step IIf), hyperuricemia-related CpG sites are removed (Step IIg) and CpG sites located in uric acid-associated genes are excluded (Step IIh). Remaining nine gouty inflammation-specific CpG sites are examined with monocyte-specific cis-methylation quantitative trait loci (cis-meQTL) analysis (Step IIIa) and monocyte-specific genetic analysis (Step IIIb) to exclude confounding by genetic factors. Further co-methylation analysis (Step IVa) and functional annotation (Step IVb) support their regulatory potential. In the final steps, potential transcription factors involved in respective regulation (Step Va-Vb) are found through MoLoTool identification (Step Va) and ReMap retrieval (Step Vb), and monocyte-specific associations of surviving CpG methylation with clinical characteristics are analysed with CellDMC, adjusting for sex, age, alcohol drinking, smoking status, smoking history (total pack-years) and cell fractions (Step Vc).





**Supplementary Figure 2. Overlap of differential methylation in different cell lineages.** The plots show the overlap of differential methylation between monocytes, eosinophils, neutrophils, NK cells, B cells, CD4+ T cells and CD8+ T cells at the cytosine-phosphate-guanine dinucleotide (CpG) site level (A) and at the gene level (B). The darkness of heatmap corresponds to the degree of overlap (0%-100%) of differential methylation between different cell lineages.





**Supplementary Figure 3. Protein-protein interaction network.** The protein-protein interaction network reveals several hub genes regulating interleukin-1β (IL-1β) (Table 1, Suppolementary Table 3). Hub genes regulating IL-1β are highlighted with respective hues.





**Supplementary Figure 4. Associations of failed methylation sites with metabolic traits and hyperuricemia.** The graphical representation shows monocyte-specific associations between cg18886702, cg00091098, cg22408430, cg13204333, cg14326053, cg10027934 methylation and low density lipoprotein (blue line), triglyceride (red line), HbA_1c_ levels (cyan line) and hyperuricemia (green line). The radii mean minus log_10_*P* of monocyte-specific associations between cytosine-phosphate-guanine dinucleotide (CpG) sites and phenotypes. The dashed purple circle shows threshold value of *P*=0.05. For example, cg10027934 is monocyte-specifically associated with low density lipoprotein (*P*=0.0019, minus log_10_*P*=2.72) but is not monocyte-specifically associated with triglyceride (*P*=0.2303, minus log_10_*P*=0.64), HbA_1c_ (*P*=0.9220, minus log_10_*P*=0.04) and hyperuricemia (*P*=0.5481, minus log_10_*P*=0.26). Data are analysed using CellDMC, adjusting for sex, age, alcohol drinking, smoking status, smoking history (total pack-years) and cell fractions (see supplementary methods).





**Supplementary Figure 5.** **Cis-methyl-quantitative trait locus (cis-meQTL) and genetic analysis of cg10314750.** (A) Regional association plots of monocyte-specific associations between nearby genetic variants and cg10314750 methylation. X-axis represents positions on respective chromosome. Y axis represents minus log_10_*P* of monocyte-specific associations between genetic variants and cg10314750 methylation. (B) Regional association plots of monocyte-specific associations between nearby genetic variants and gout. X-axis represents positions on respective chromosome. Y axis represents minus log_10_*P* of monocyte-specific associations between genetic variants and gout. Every point is one genetic variant coloured with respective hue, with different colours implying different genetic variants. The dashed purple lines indicate the significance threshold (*P* = 0.05) and the red box highlights the location of cg10314750. Monocyte-specific associations of genetic variants with cg10314750 methylation and gout are analysed using multiple regression analysis, adjusting for sex, age, alcohol drinking, smoking status, smoking history (total pack-years) and cell fractions (see supplementary methods).





**Supplementary Figure 6.** **Cis-methyl-quantitative trait locus (cis-meQTL) and genetic analysis of cg03795507.** (A) Regional association plots of monocyte-specific associations between nearby genetic variants and cg03795507 methylation. X-axis represents positions on respective chromosome. Y axis represents minus log_10_*P* of monocyte-specific associations between genetic variants and cg03795507 methylation. (B) Regional association plots of monocyte-specific associations between nearby genetic variants and gout. X-axis represents positions on respective chromosome. Y axis represents minus log_10_*P* of monocyte-specific associations between genetic variants and gout. Every point is one genetic variant coloured with respective hue, with different colours implying different genetic variants. The dashed purple lines indicate the significance threshold (*P* = 0.05) and the red box highlights the location of cg03795507. Monocyte-specific associations of genetic variants with cg03795507 methylation and gout are analysed using multiple regression analysis, adjusting for sex, age, alcohol drinking, smoking status, smoking history (total pack-years) and cell fractions (see supplementary methods).





**Supplementary Figure 7.** **Cis-methyl-quantitative trait locus (cis-meQTL) and genetic analysis of cg10257063.** (A) Regional association plots of monocyte-specific associations between nearby genetic variants and cg10257063 methylation. X-axis represents positions on respective chromosome. Y axis represents minus log_10_*P* of monocyte-specific associations between genetic variants and cg10257063 methylation. (B) Regional association plots of monocyte-specific associations between nearby genetic variants and gout. X-axis represents positions on respective chromosome. Y axis represents minus log_10_*P* of monocyte-specific associations between genetic variants and gout. Every point is one genetic variant coloured with respective hue, with different colours implying different genetic variants. The dashed purple lines indicate the significance threshold (*P* = 0.05) and the red box highlights the location of cg10257063. Monocyte-specific associations of genetic variants with cg10257063 methylation and gout are analysed using multiple regression analysis, adjusting for sex, age, alcohol drinking, smoking status, smoking history (total pack-years) and cell fractions (see supplementary methods).





**Supplementary Figure 8.** **Cis-methyl-quantitative trait locus (cis-meQTL) and genetic analysis of cg16975613.** (A) Regional association plots of monocyte-specific associations between nearby genetic variants and cg16975613 methylation. X-axis represents positions on respective chromosome. Y axis represents minus log_10_*P* of monocyte-specific associations between genetic variants and cg16975613 methylation. (B) Regional association plots of monocyte-specific associations between nearby genetic variants and gout. X-axis represents positions on respective chromosome. Y axis represents minus log_10_*P* of monocyte-specific associations between genetic variants and gout. Every point is one genetic variant coloured with respective hue, with different colours implying different genetic variants. The dashed purple lines indicate the significance threshold (*P* = 0.05) and the red box highlights the location of cg16975613. Monocyte-specific associations of genetic variants with cg16975613 methylation and gout are analysed using multiple regression analysis, adjusting for sex, age, alcohol drinking, smoking status, smoking history (total pack-years) and cell fractions (see supplementary methods).





**Supplementary Figure 9.** **Cis-methyl-quantitative trait locus (cis-meQTL) and genetic analysis of cg16630982.** (A) Regional association plots of monocyte-specific associations between nearby genetic variants and cg16630982 methylation. X-axis represents positions on respective chromosome. Y axis represents minus log_10_*P* of monocyte-specific associations between genetic variants and cg16630982 methylation. (B) Regional association plots of monocyte-specific associations between nearby genetic variants and gout. X-axis represents positions on respective chromosome. Y axis represents minus log_10_*P* of monocyte-specific associations between genetic variants and gout. Every point is one genetic variant coloured with respective hue, with different colours implying different genetic variants. The dashed purple lines indicate the significance threshold (*P* = 0.05) and the red box highlights the location of cg16630982. Monocyte-specific associations of genetic variants with cg16630982 methylation and gout are analysed using multiple regression analysis, adjusting for sex, age, alcohol drinking, smoking status, smoking history (total pack-years) and cell fractions (see supplementary methods).





**Supplementary Figure 10.** **Cis-methyl-quantitative trait locus (cis-meQTL) and genetic analysis of cg12182452.** (A) Regional association plots of monocyte-specific associations between nearby genetic variants and cg12182452 methylation. X-axis represents positions on respective chromosome. Y axis represents minus log_10_*P* of monocyte-specific associations between genetic variants and cg12182452 methylation. (B) Regional association plots of monocyte-specific associations between nearby genetic variants and gout. X-axis represents positions on respective chromosome. Y axis represents minus log_10_*P* of monocyte-specific associations between genetic variants and gout. Every point is one genetic variant coloured with respective hue, with different colours implying different genetic variants. The dashed purple lines indicate the significance threshold (*P* = 0.05) and the red box highlights the location of cg12182452. Monocyte-specific associations of genetic variants with cg12182452 methylation and gout are analysed using multiple regression analysis, adjusting for sex, age, alcohol drinking, smoking status, smoking history (total pack-years) and cell fractions (see supplementary methods).





**Supplementary Figure 11.** **Cis-methyl-quantitative trait locus (cis-meQTL) and genetic analysis of cg17151991.** (A) Regional association plots of monocyte-specific associations between nearby genetic variants and cg17151991 methylation. X-axis represents positions on respective chromosome. Y axis represents minus log_10_*P* of monocyte-specific associations between genetic variants and cg17151991 methylation. (B) Regional association plots of monocyte-specific associations between nearby genetic variants and gout. X-axis represents positions on respective chromosome. Y axis represents minus log_10_*P* of monocyte-specific associations between genetic variants and gout. Every point is one genetic variant coloured with respective hue, with different colours implying different genetic variants. The dashed purple lines indicate the significance threshold (*P* = 0.05) and the red box highlights the location of cg17151991. Monocyte-specific associations of genetic variants with cg17151991 methylation and gout are analysed using multiple regression analysis, adjusting for sex, age, alcohol drinking, smoking status, smoking history (total pack-years) and cell fractions (see supplementary methods).





**Supplementary Figure 12.** **Cis-methyl-quantitative trait locus (cis-meQTL) and genetic analysis of cg26375855.** (A) Regional association plots of monocyte-specific associations between nearby genetic variants and cg26375855 methylation. X-axis represents positions on respective chromosome. Y axis represents minus log_10_*P* of monocyte-specific associations between genetic variants and cg26375855 methylation. (B) Regional association plots of monocyte-specific associations between nearby genetic variants and gout. X-axis represents positions on respective chromosome. Y axis represents minus log_10_*P* of monocyte-specific associations between genetic variants and gout. Every point is one genetic variant coloured with respective hue, with different colours implying different genetic variants. The dashed purple lines indicate the significance threshold (*P* = 0.05) and the red box highlights the location of cg26375855. Monocyte-specific associations of genetic variants with cg26375855 methylation and gout are analysed using multiple regression analysis, adjusting for sex, age, alcohol drinking, smoking status, smoking history (total pack-years) and cell fractions (see supplementary methods).

.





**Supplementary Figure 13. Co-methylation analysis and functional annotation of cg10314750.** (A) Patterns of co-methylation at the cytosine-phosphate-guanine dinucleotide (CpG) sites surrounding cg10314750. (B) Monocyte-specific regional association results along with position of nearby CpG island (dark violet), CpG shore (yellow), CpG shelf (indigo) and CpG open sea (green yellow). cg10314750 (highlighted in shaded box) is located in open sea. (C) Functional annotation of cg10314750. DNase hypersensitive sites derived by DNase-seq (DNase Track) and histone marks surrounding cg10314750 (H3K4me1, H3K4me3, H3K9ac and H3K27ac Tracks) in monocytes are shown. DNase hypersensitivity, H3K4me1, H3K4me3, H3K9ac and H3K27ac histone marks are associated with active regulatory elements.


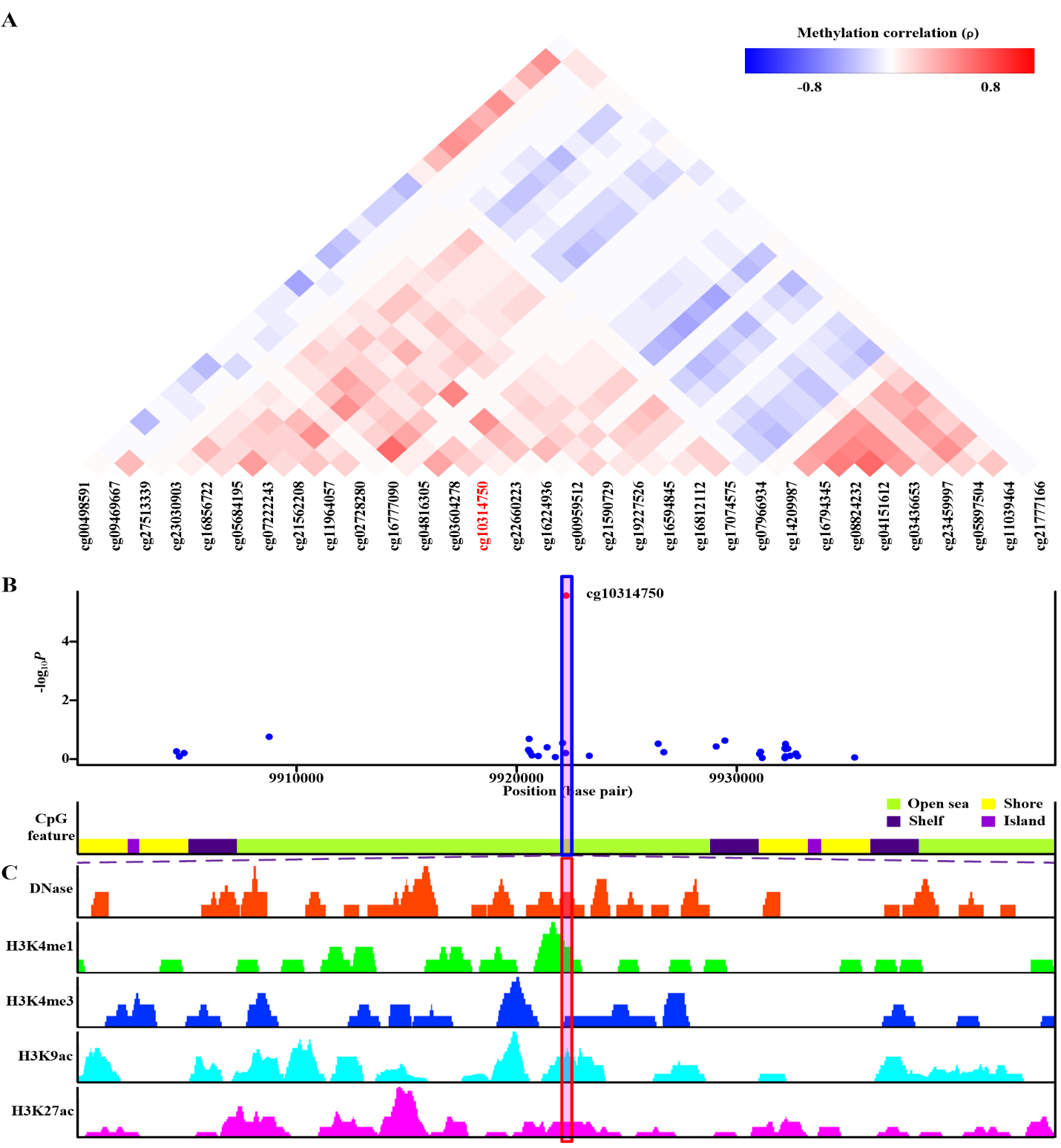


**Supplementary Figure 14. Co-methylation analysis and functional annotation of cg03795507.** (A) Patterns of co-methylation at the cytosine-phosphate-guanine dinucleotide (CpG) sites surrounding cg03795507. (B) Monocyte-specific regional association results along with position of nearby CpG island (dark violet), CpG shore (yellow), CpG shelf (indigo) and CpG open sea (green yellow). cg03795507 (highlighted in shaded box) is located in CpG island. (C) Functional annotation of cg03795507. DNase hypersensitive sites derived by DNase-seq (DNase Track) and histone marks surrounding cg03795507 (H3K4me1, H3K4me3, H3K9ac and H3K27ac Tracks) in monocytes are shown. DNase hypersensitivity, H3K4me1, H3K4me3, H3K9ac and H3K27ac histone marks are associated with active regulatory elements.


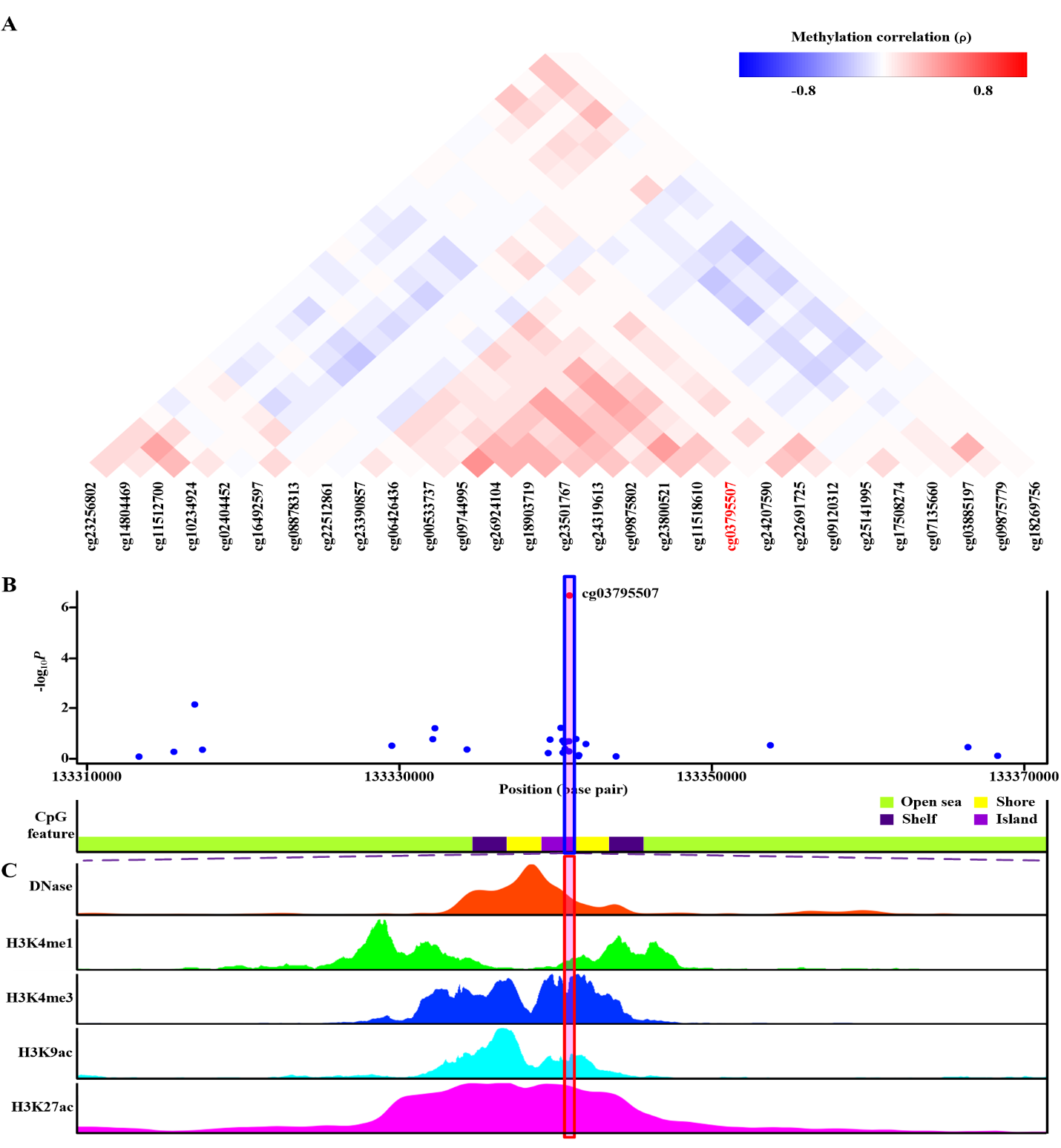


**Supplementary Figure 15. Co-methylation analysis and functional annotation of cg10257063.** (A) Patterns of co-methylation at the cytosine-phosphate-guanine dinucleotide (CpG) sites surrounding cg10257063. (B) Monocyte-specific regional association results along with position of nearby CpG island (dark violet), CpG shore (yellow), CpG shelf (indigo) and CpG open sea (green yellow). cg10257063 (highlighted in shaded box) is located in CpG open sea. (C) Functional annotation of cg10257063. DNase hypersensitive sites derived by DNase-seq (DNase Track) and histone marks surrounding cg10257063 (H3K4me1, H3K4me3, H3K9ac and H3K27ac Tracks) in monocytes are shown. DNase hypersensitivity, H3K4me1, H3K4me3, H3K9ac and H3K27ac histone marks are associated with active regulatory elements.


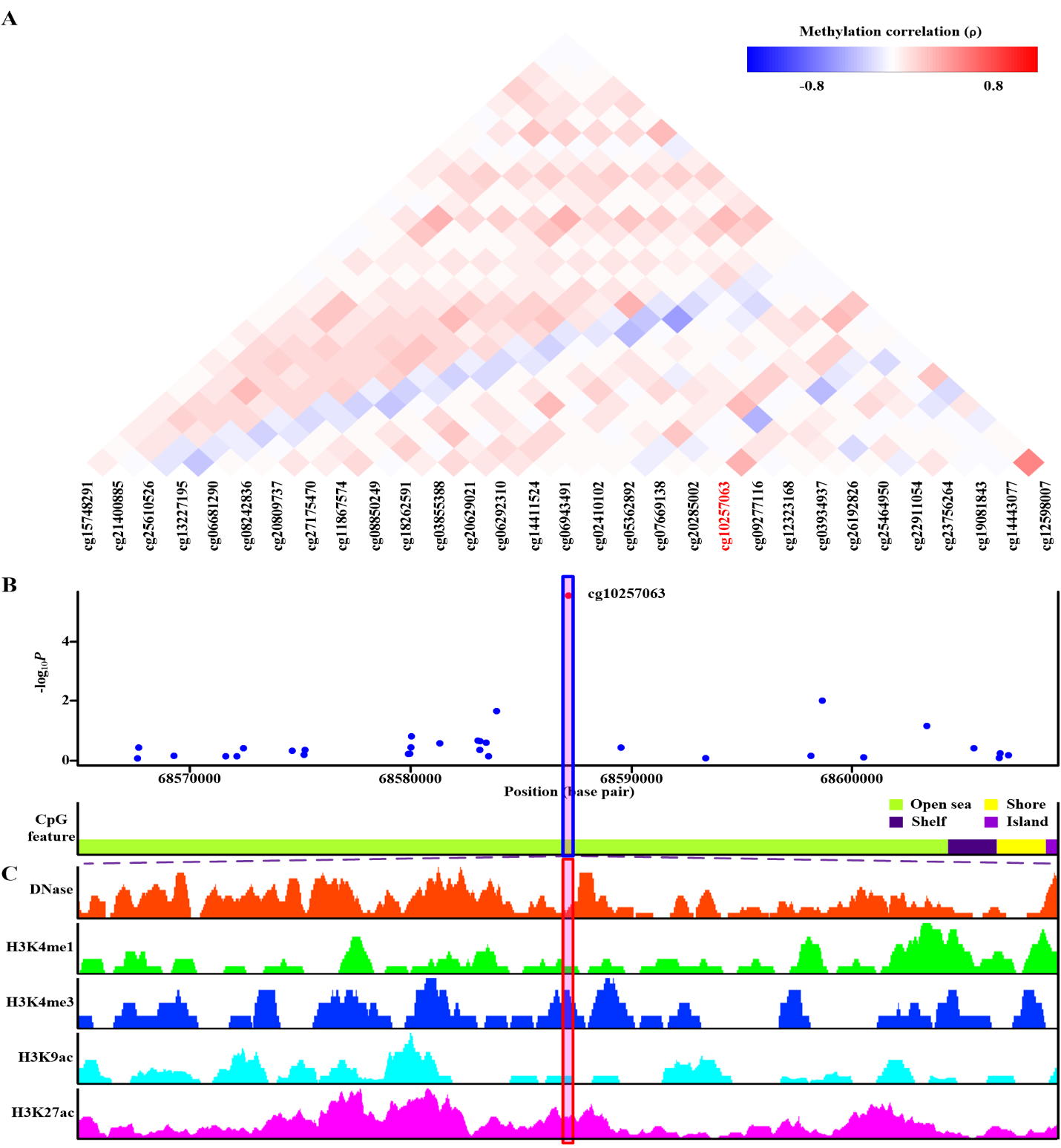


**Supplementary Figure 16. Co-methylation analysis and functional annotation of cg16975613.** (A) Patterns of co-methylation at the cytosine-phosphate-guanine dinucleotide (CpG) sites surrounding cg16975613. (B) Monocyte-specific regional association results along with position of nearby CpG island (dark violet), CpG shore (yellow), CpG shelf (indigo) and CpG open sea (green yellow). cg16975613 (highlighted in shaded box) is located in CpG island. (C) Functional annotation of cg16975613. DNase hypersensitive sites derived by DNase-seq (DNase Track) and histone marks surrounding cg16975613 (H3K4me1, H3K4me3, H3K9ac and H3K27ac Tracks) in monocytes are shown. DNase hypersensitivity, H3K4me1, H3K4me3, H3K9ac and H3K27ac histone marks are associated with active regulatory elements.


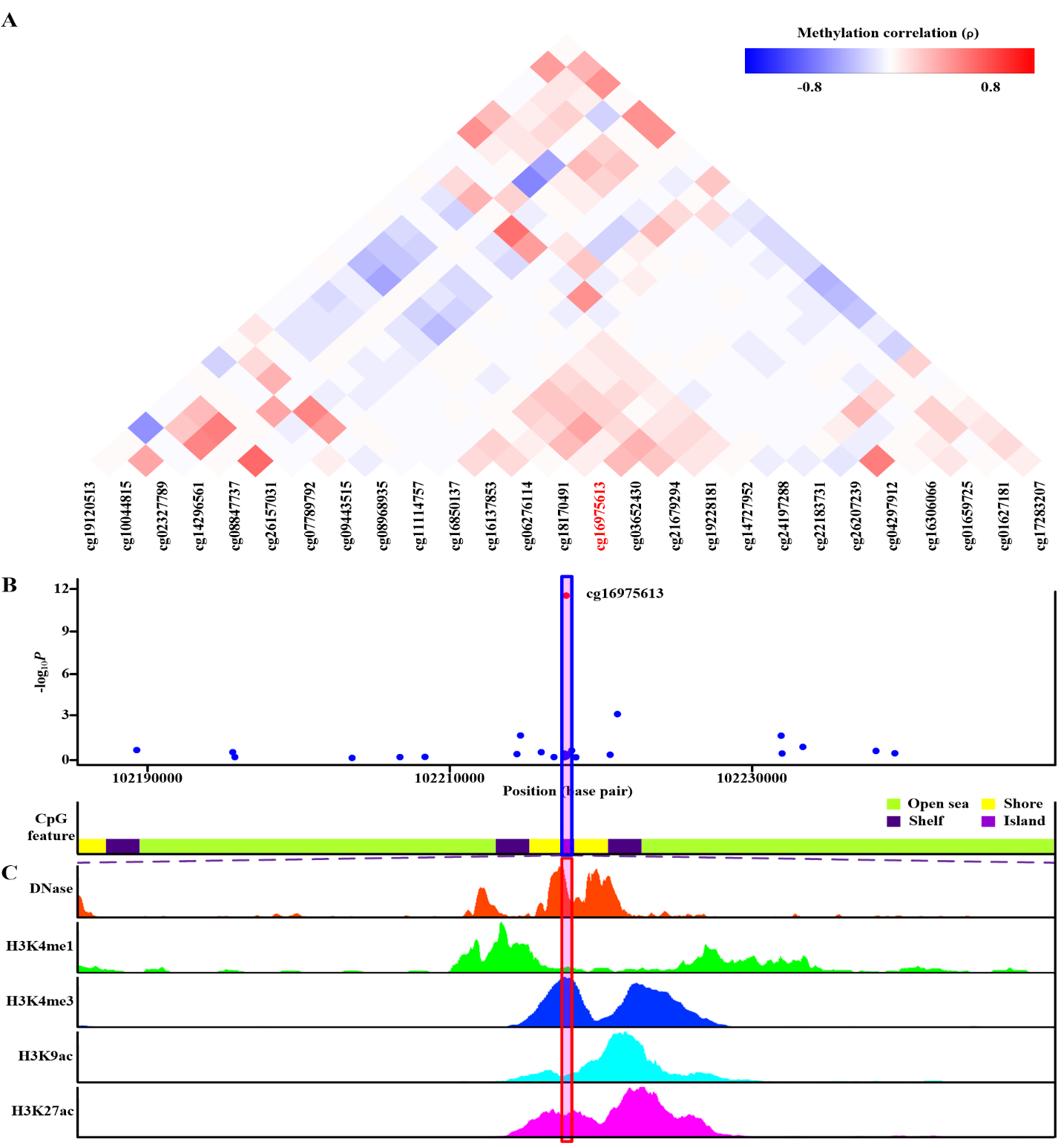


**Supplementary Figure 17. Co-methylation analysis and functional annotation of cg16630982.** (A) Patterns of co-methylation at the cytosine-phosphate-guanine dinucleotide (CpG) sites surrounding cg16630982. cg16630982 is highlighted with red while cg12182452 is highlighted with blue. (B) Monocyte-specific regional association results along with position of nearby CpG island (dark violet), CpG shore (yellow), CpG shelf (indigo) and CpG open sea (green yellow). cg16630982 (highlighted in shaded box) is located in CpG shore. (C) Functional annotation of cg16630982. DNase hypersensitive sites derived by DNase-seq (DNase Track) and histone marks surrounding cg16630982 (H3K4me1, H3K4me3, H3K9ac and H3K27ac Tracks) in monocytes are shown. DNase hypersensitivity, H3K4me1, H3K4me3, H3K9ac and H3K27ac histone marks are associated with active regulatory elements.


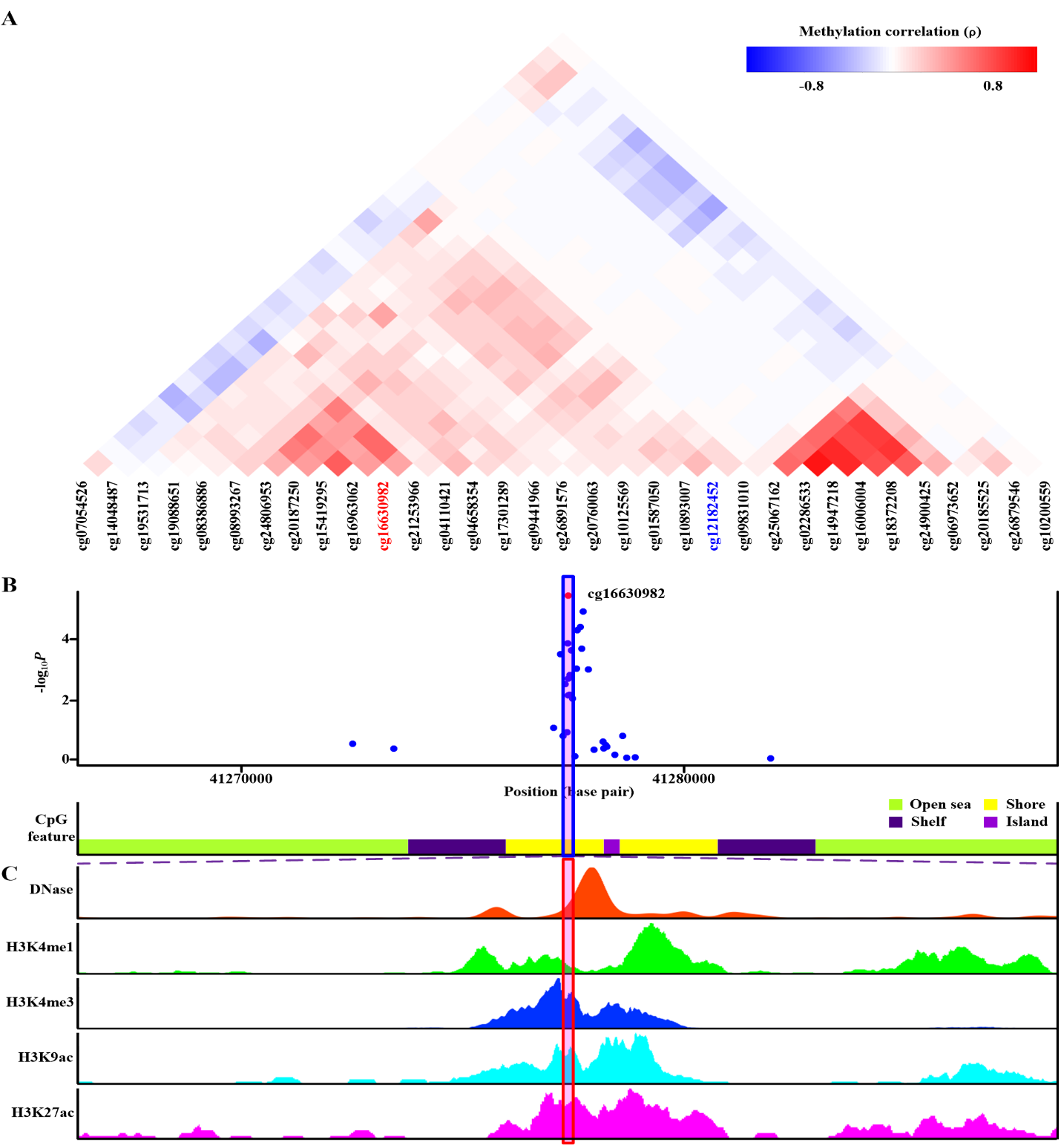


**Supplementary Figure 18. Co-methylation analysis and functional annotation of cg12182452.** (A) Patterns of co-methylation at the cytosine-phosphate-guanine dinucleotide (CpG) sites surrounding cg12182452. (B) Monocyte-specific regional association results along with position of nearby CpG island (dark violet), CpG shore (yellow), CpG shelf (indigo) and CpG open sea (green yellow). cg12182452 (highlighted in shaded box) is located in CpG shore. (C) Functional annotation of cg12182452. DNase hypersensitive sites derived by DNase-seq (DNase Track) and histone marks surrounding cg12182452 (H3K4me1, H3K4me3, H3K9ac and H3K27ac Tracks) in monocytes are shown. DNase hypersensitivity, H3K4me1, H3K4me3, H3K9ac and H3K27ac histone marks are associated with active regulatory elements.


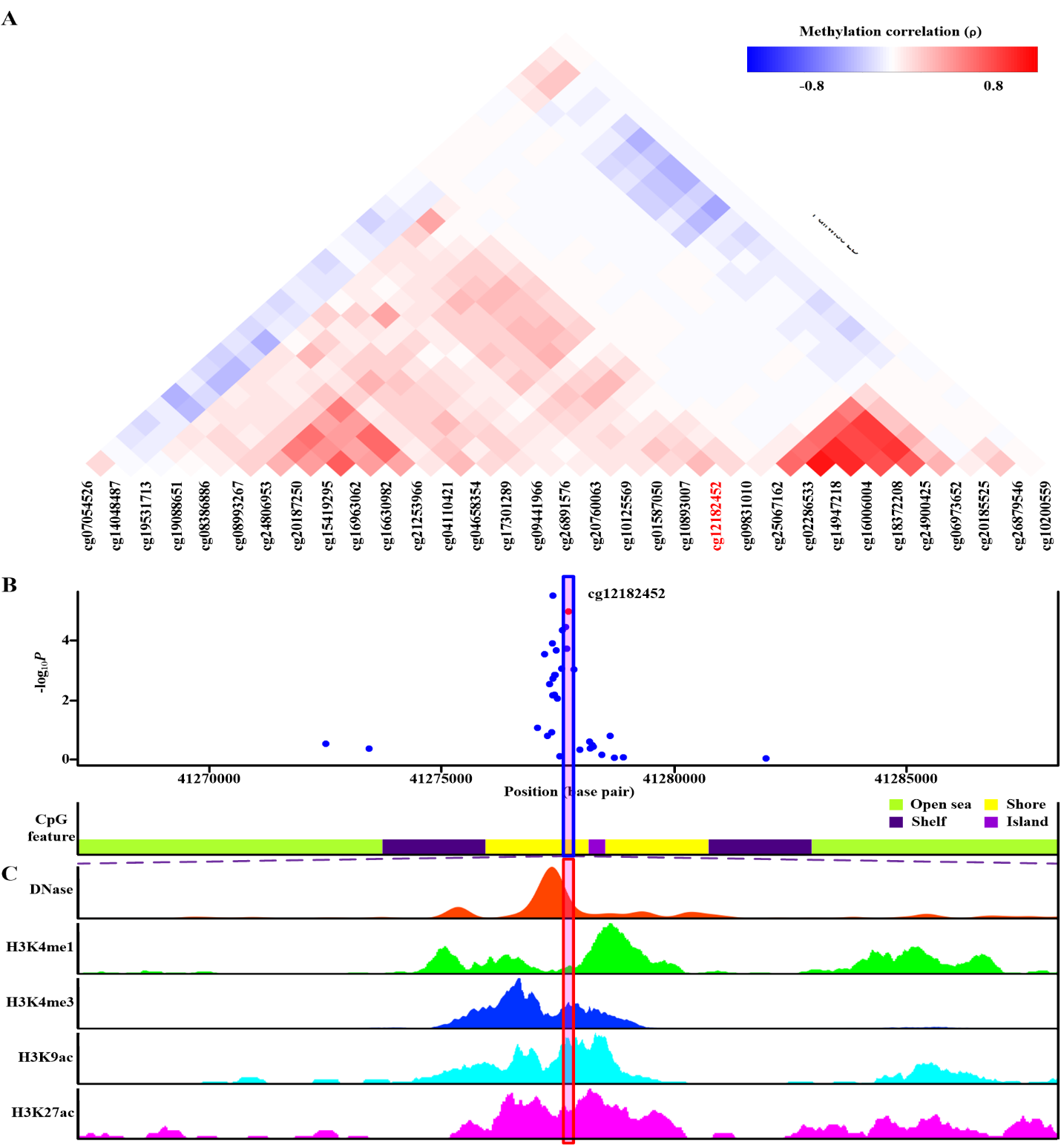


**Supplementary Figure 19. Co-methylation analysis and functional annotation of cg17151991.** (A) Patterns of co-methylation at the cytosine-phosphate-guanine dinucleotide (CpG) sites surrounding cg17151991. (B) Monocyte-specific regional association results along with position of nearby CpG island (dark violet), CpG shore (yellow), CpG shelf (indigo) and CpG open sea (green yellow). cg17151991 (highlighted in shaded box) is located in CpG island. (C) Functional annotation of cg17151991. DNase hypersensitive sites derived by DNase-seq (DNase Track) and histone marks surrounding cg17151991 (H3K4me1, H3K4me3, H3K9ac and H3K27ac Tracks) in monocytes are shown. DNase hypersensitivity, H3K4me1, H3K4me3, H3K9ac and H3K27ac histone marks are associated with active regulatory elements.


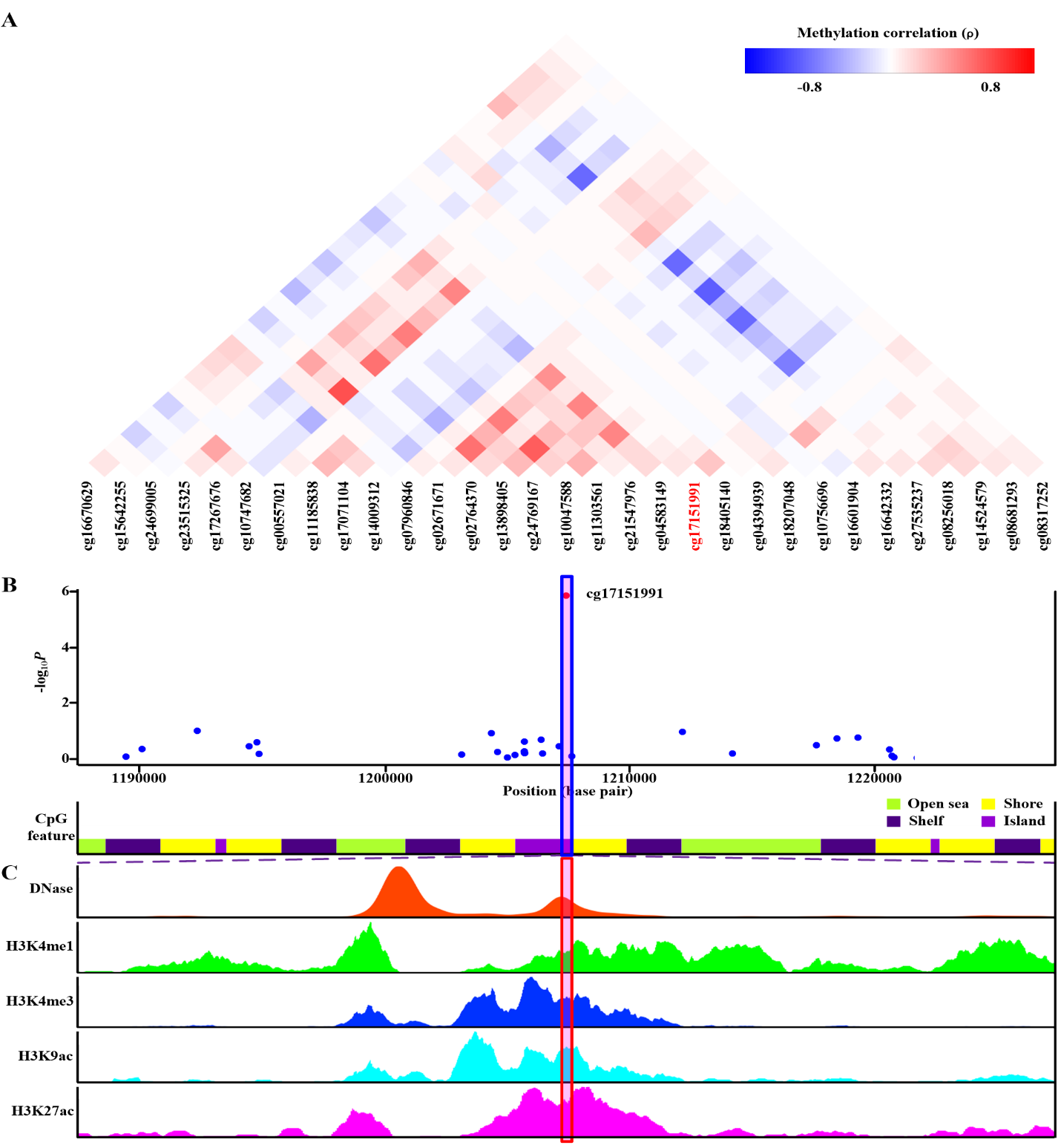


**Supplementary Figure 20. Co-methylation analysis and functional annotation of cg26375855.** (A) Patterns of co-methylation at the cytosine-phosphate-guanine dinucleotide (CpG) sites surrounding cg26375855. (B) Monocyte-specific regional association results along with position of nearby CpG island (dark violet), CpG shore (yellow), CpG shelf (indigo) and CpG open sea (green yellow). cg26375855 (highlighted in shaded box) is located in CpG open sea. (C) Functional annotation of cg26375855. DNase hypersensitive sites derived by DNase-seq (DNase Track) and histone marks surrounding cg26375855 (H3K4me1, H3K4me3, H3K9ac and H3K27ac Tracks) in monocytes are shown. DNase hypersensitivity, H3K4me1, H3K4me3, H3K9ac and H3K27ac histone marks are associated with active regulatory elements.


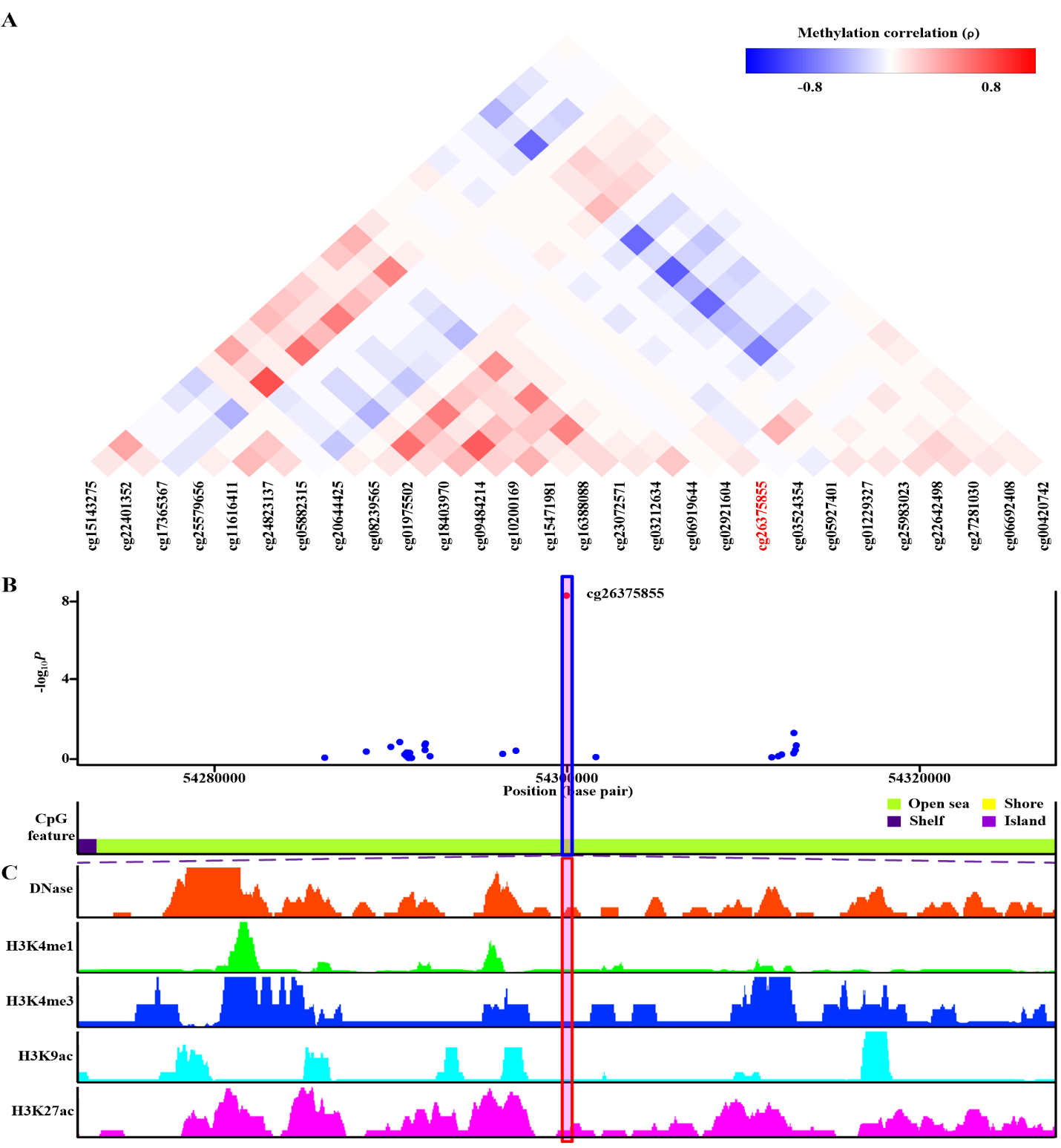


**Supplementary Figure 21. Co-methylation analysis and functional annotation of cg18886702.** (A) Patterns of co-methylation at the cytosine-phosphate-guanine dinucleotide (CpG) sites surrounding cg18886702. (B) Monocyte-specific regional association results along with position of nearby CpG island (dark violet), CpG shore (yellow), CpG shelf (indigo) and CpG open sea (green yellow). cg18886702 (highlighted in shaded box) is located in CpG open sea. (C) Functional annotation of cg18886702. DNase hypersensitive sites derived by DNase-seq (DNase Track) and histone marks surrounding cg18886702 (H3K4me1, H3K4me3, H3K9ac and H3K27ac Tracks) in monocytes are shown. DNase hypersensitivity, H3K4me1, H3K4me3, H3K9ac and H3K27ac histone marks are associated with active regulatory elements.


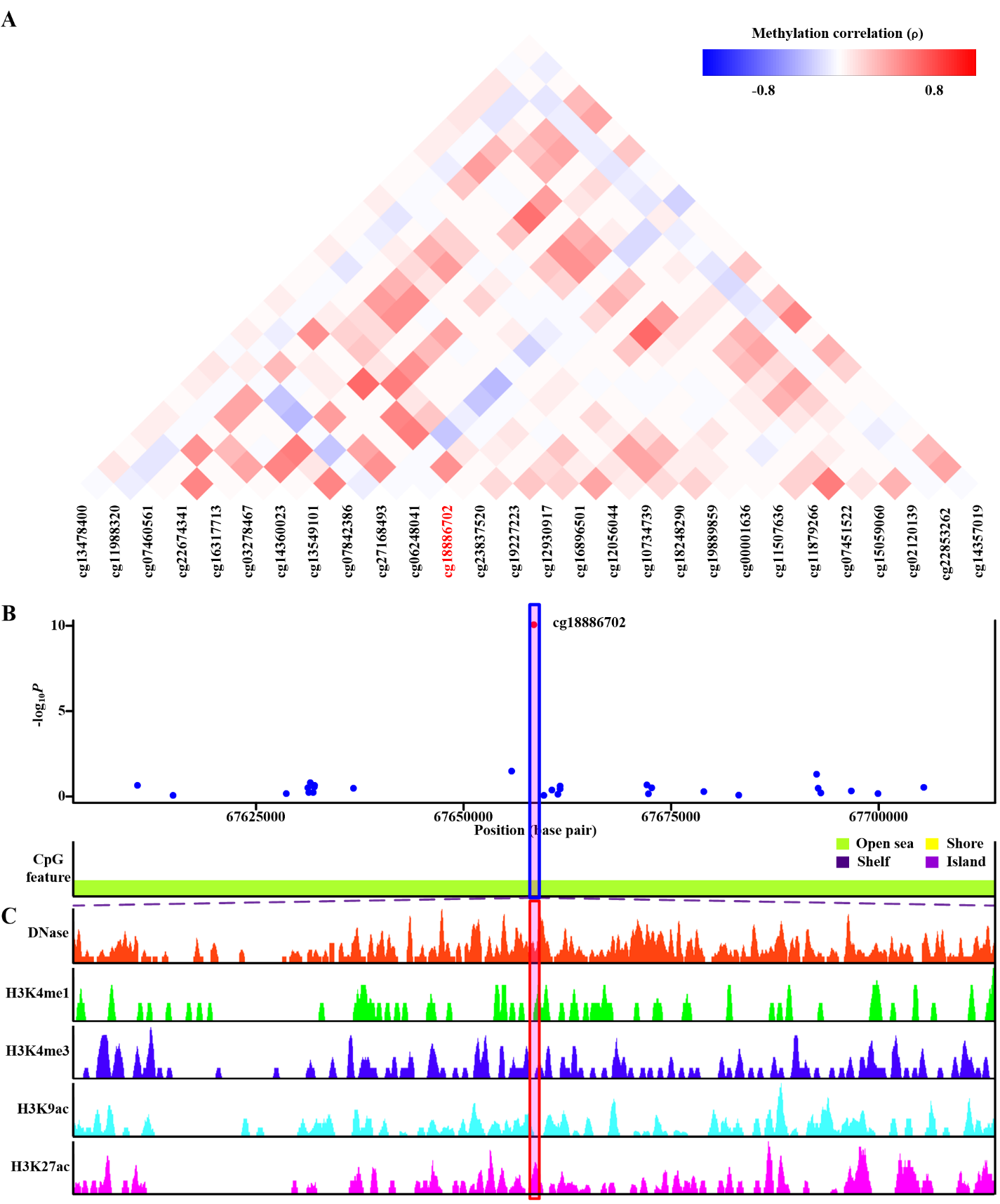


**Supplementary Figure 22. Co-methylation analysis and functional annotation of cg00091098.** (A) Patterns of co-methylation at the cytosine-phosphate-guanine dinucleotide (CpG) sites surrounding cg00091098. (B) Monocyte-specific regional association results along with position of nearby CpG island (dark violet), CpG shore (yellow), CpG shelf (indigo) and CpG open sea (green yellow). cg00091098 (highlighted in shaded box) is located in CpG open sea. (C) Functional annotation of cg00091098. DNase hypersensitive sites derived by DNase-seq (DNase Track) and histone marks surrounding cg00091098 (H3K4me1, H3K4me3, H3K9ac and H3K27ac Tracks) in monocytes are shown. DNase hypersensitivity, H3K4me1, H3K4me3, H3K9ac and H3K27ac histone marks are associated with active regulatory elements.


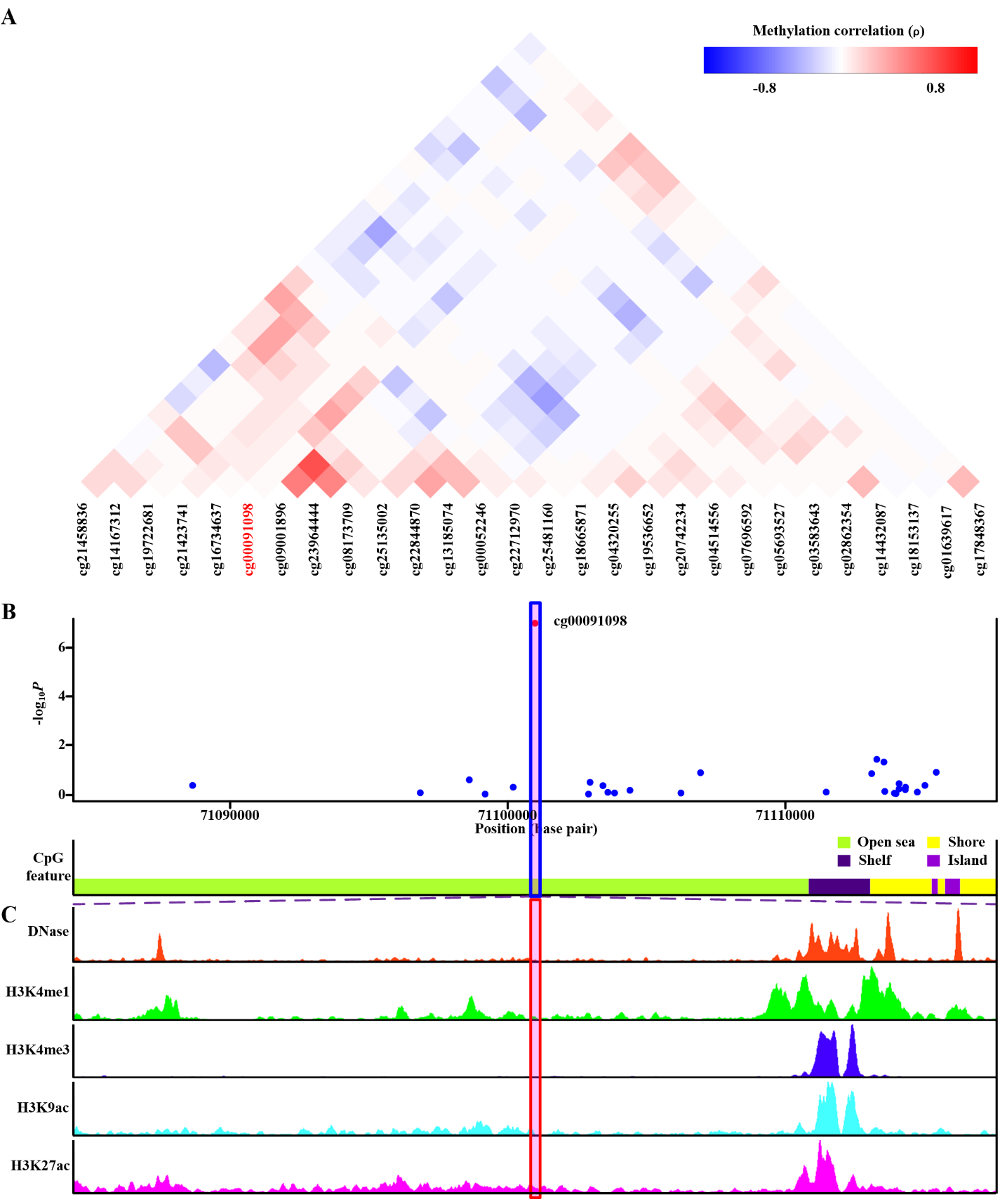


**Supplementary Figure 23. Co-methylation analysis and functional annotation of cg22408430.** (A) Patterns of co-methylation at the cytosine-phosphate-guanine dinucleotide (CpG) sites surrounding cg22408430. (B) Monocyte-specific regional association results along with position of nearby CpG island (dark violet), CpG shore (yellow), CpG shelf (indigo) and CpG open sea (green yellow). cg22408430 (highlighted in shaded box) is located in CpG shore. (C) Functional annotation of cg22408430. DNase hypersensitive sites derived by DNase-seq (DNase Track) and histone marks surrounding cg22408430 (H3K4me1, H3K4me3, H3K9ac and H3K27ac Tracks) in monocytes are shown. DNase hypersensitivity, H3K4me1, H3K4me3, H3K9ac and H3K27ac histone marks are associated with active regulatory elements.


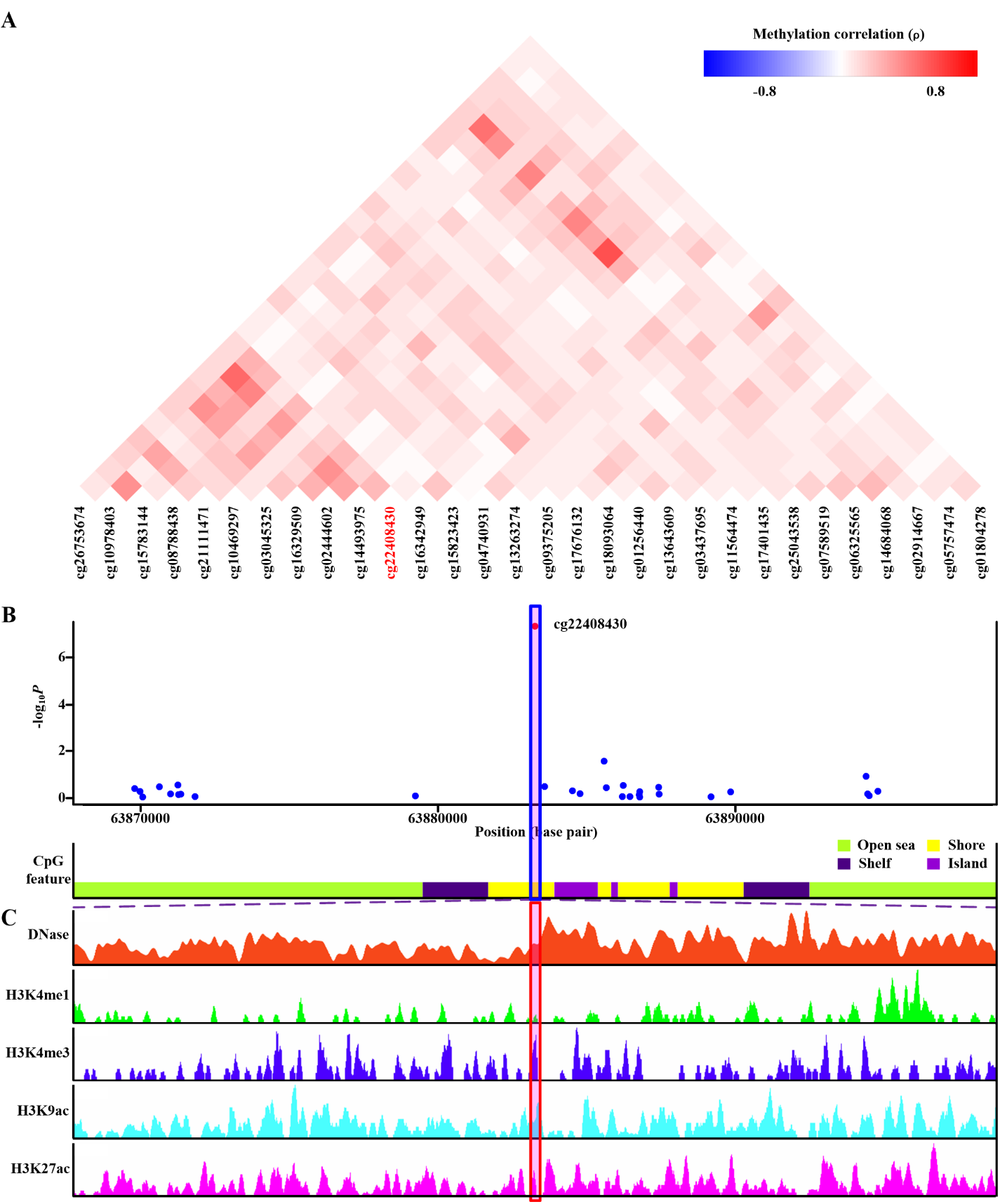


**Supplementary Figure 24. Co-methylation analysis and functional annotation of cg13204333.** (A) Patterns of co-methylation at the cytosine-phosphate-guanine dinucleotide (CpG) sites surrounding cg13204333. (B) Monocyte-specific regional association results along with position of nearby CpG island (dark violet), CpG shore (yellow), CpG shelf (indigo) and CpG open sea (green yellow). cg13204333 (highlighted in shaded box) is located in CpG open sea. (C) Functional annotation of cg13204333. DNase hypersensitive sites derived by DNase-seq (DNase Track) and histone marks surrounding cg13204333 (H3K4me1, H3K4me3, H3K9ac and H3K27ac Tracks) in monocytes are shown. DNase hypersensitivity, H3K4me1, H3K4me3, H3K9ac and H3K27ac histone marks are associated with active regulatory elements.


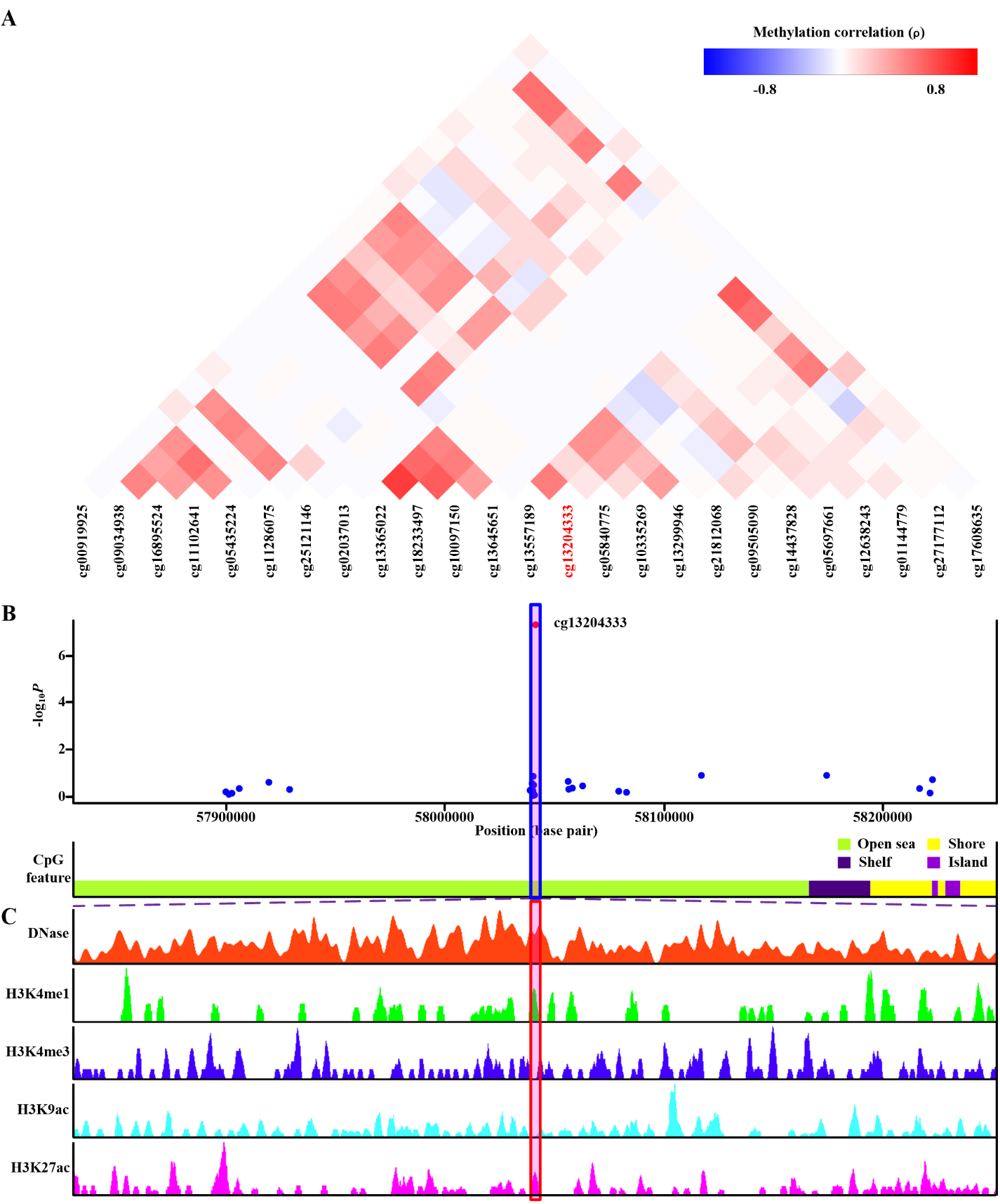


**Supplementary Figure 25. Co-methylation analysis and functional annotation of cg14326053.** (A) Patterns of co-methylation at the cytosine-phosphate-guanine dinucleotide (CpG) sites surrounding cg14326053. (B) Monocyte-specific regional association results along with position of nearby CpG island (dark violet), CpG shore (yellow), CpG shelf (indigo) and CpG open sea (green yellow). cg14326053 (highlighted in shaded box) is located in CpG open sea. (C) Functional annotation of cg14326053. DNase hypersensitive sites derived by DNase-seq (DNase Track) and histone marks surrounding cg14326053 (H3K4me1, H3K4me3, H3K9ac and H3K27ac Tracks) in monocytes are shown. DNase hypersensitivity, H3K4me1, H3K4me3, H3K9ac and H3K27ac histone marks are associated with active regulatory elements.


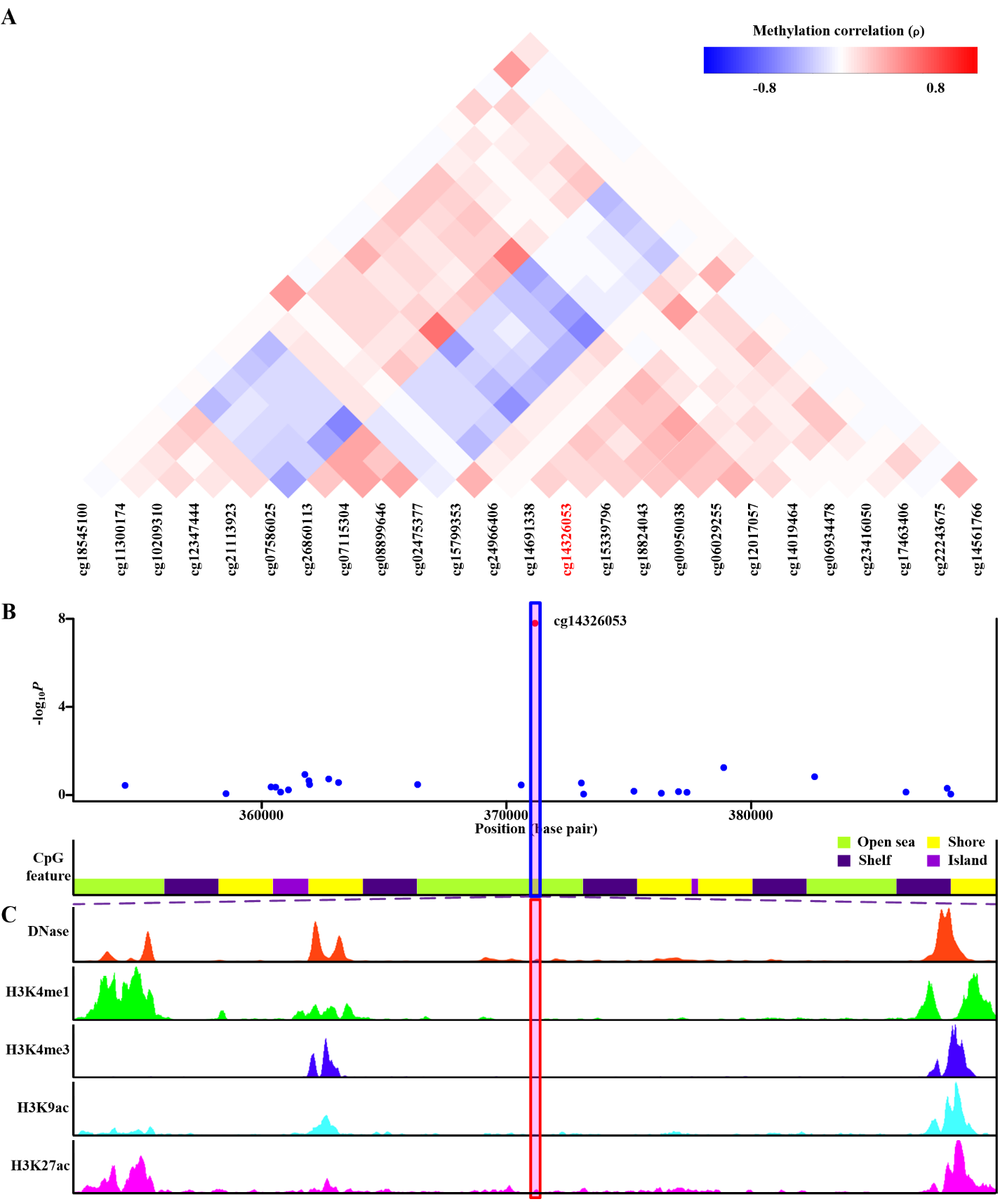


**Supplementary Figure 26. Co-methylation analysis and functional annotation of cg10027934.** (A) Patterns of co-methylation at the cytosine-phosphate-guanine dinucleotide (CpG) sites surrounding cg10027934. (B) Monocyte-specific regional association results along with position of nearby CpG island (dark violet), CpG shore (yellow), CpG shelf (indigo) and CpG open sea (green yellow). cg10027934 (highlighted in shaded box) is located in CpG shelf. (C) Functional annotation of cg10027934. DNase hypersensitive sites derived by DNase-seq (DNase Track) and histone marks surrounding cg10027934 (H3K4me1, H3K4me3, H3K9ac and H3K27ac Tracks) in monocytes are shown. DNase hypersensitivity, H3K4me1, H3K4me3, H3K9ac and H3K27ac histone marks are associated with active regulatory elements.


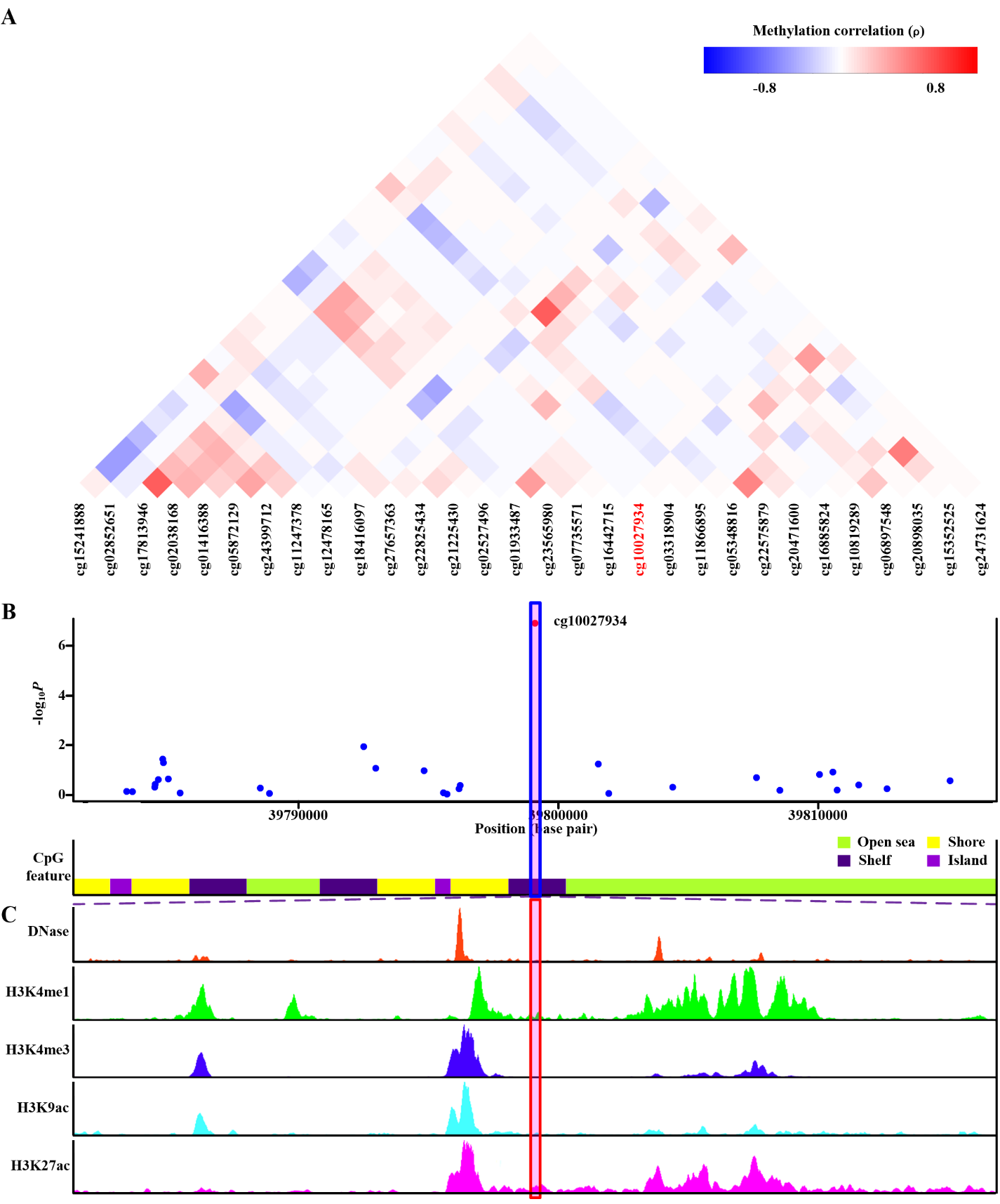


**Supplementary Figure 27. Co-methylation analysis and functional annotation of cg15158067.** (A) Patterns of co-methylation at the cytosine-phosphate-guanine dinucleotide (CpG) sites surrounding cg15158067. (B) Monocyte-specific regional association results along with position of nearby CpG island (dark violet), CpG shore (yellow), CpG shelf (indigo) and CpG open sea (green yellow). cg15158067 (highlighted in shaded box) is located in CpG shore. (C) Functional annotation of cg15158067. DNase hypersensitive sites derived by DNase-seq (DNase Track) and histone marks surrounding cg15158067 (H3K4me1, H3K4me3, H3K9ac and H3K27ac Tracks) in monocytes are shown. DNase hypersensitivity, H3K4me1, H3K4me3, H3K9ac and H3K27ac histone marks are associated with active regulatory elements.


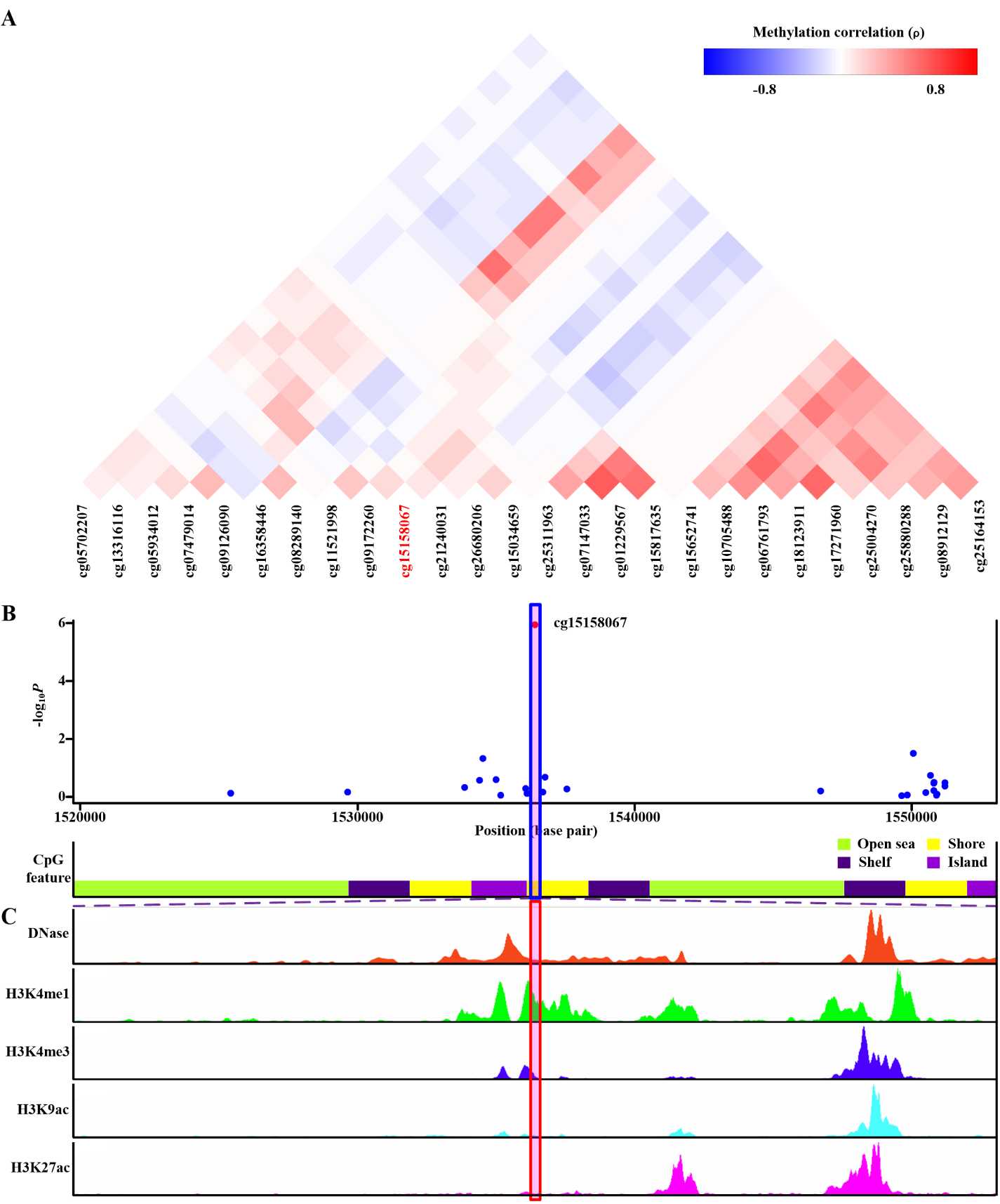


**Supplementary Figure 28. Co-methylation analysis and functional annotation of cg01718853.** (A) Patterns of co-methylation at the cytosine-phosphate-guanine dinucleotide (CpG) sites surrounding cg01718853. (B) Monocyte-specific regional association results along with position of nearby CpG island (dark violet), CpG shore (yellow), CpG shelf (indigo) and CpG open sea (green yellow). cg01718853 (highlighted in shaded box) is located in CpG shelf. (C) Functional annotation of cg01718853. DNase hypersensitive sites derived by DNase-seq (DNase Track) and histone marks surrounding cg01718853 (H3K4me1, H3K4me3, H3K9ac and H3K27ac Tracks) in monocytes are shown. DNase hypersensitivity, H3K4me1, H3K4me3, H3K9ac and H3K27ac histone marks are associated with active regulatory elements.


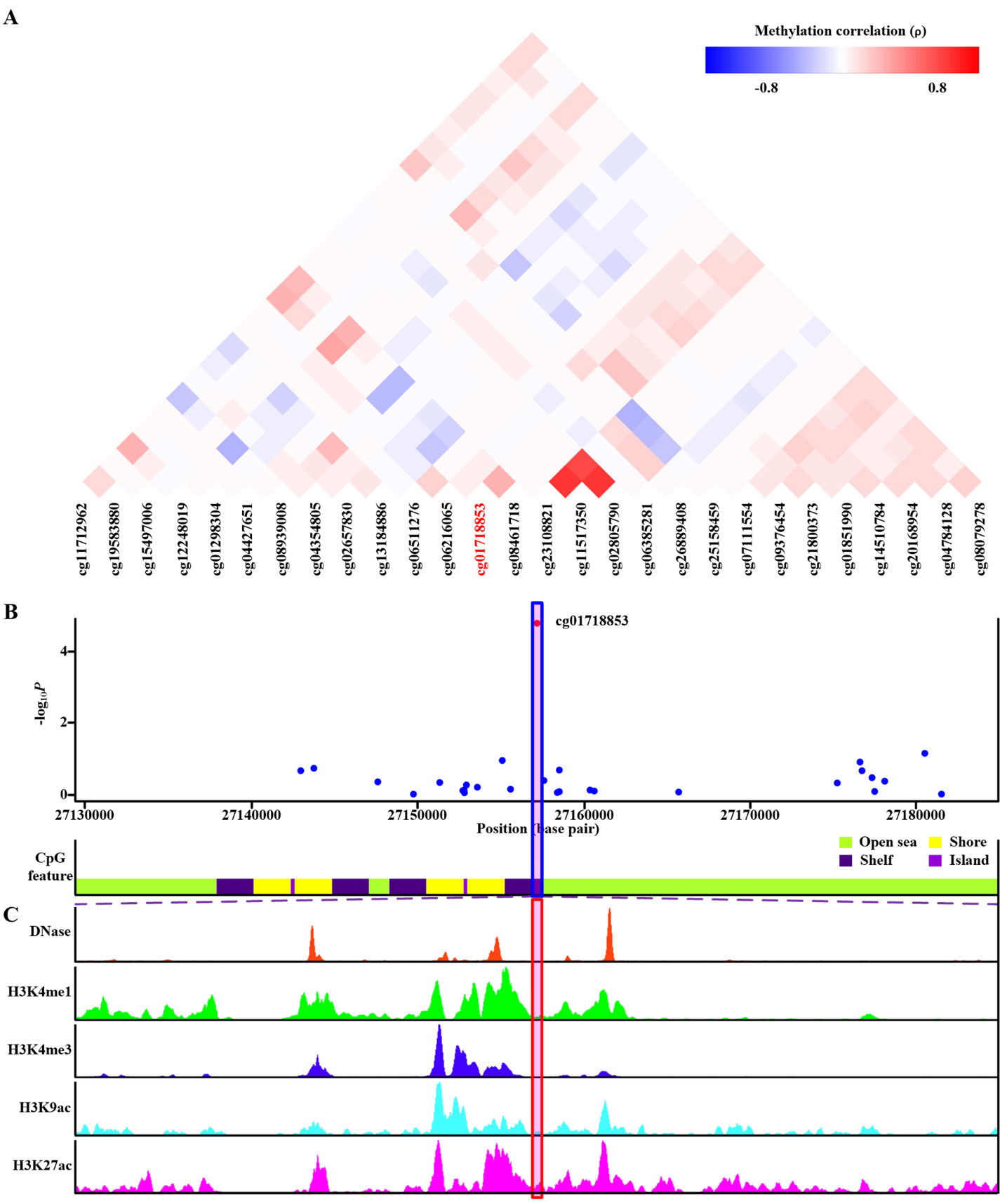


**Supplementary Figure 29. Co-methylation analysis and functional annotation of cg03789579.** (A) Patterns of co-methylation at the cytosine-phosphate-guanine dinucleotide (CpG) sites surrounding cg03789579. (B) Monocyte-specific regional association results along with position of nearby CpG island (dark violet), CpG shore (yellow), CpG shelf (indigo) and CpG open sea (green yellow). cg03789579 (highlighted in shaded box) is located in CpG shore. (C) Functional annotation of cg03789579. DNase hypersensitive sites derived by DNase-seq (DNase Track) and histone marks surrounding cg03789579 (H3K4me1, H3K4me3, H3K9ac and H3K27ac Tracks) in monocytes are shown. DNase hypersensitivity, H3K4me1, H3K4me3, H3K9ac and H3K27ac histone marks are associated with active regulatory elements.


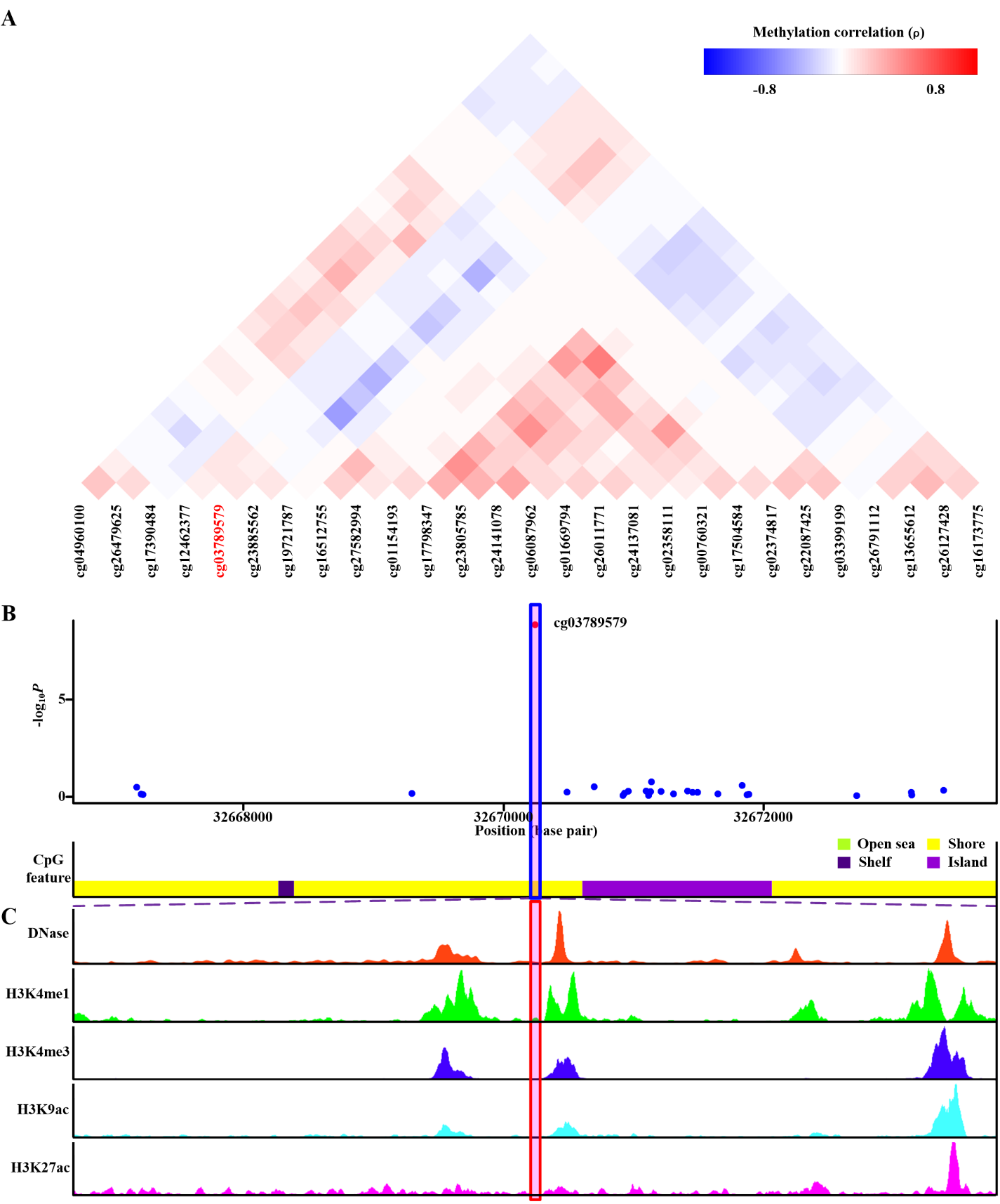


**Supplementary Figure 30. Co-methylation analysis and functional annotation of cg13559233.** (A) Patterns of co-methylation at the cytosine-phosphate-guanine dinucleotide (CpG) sites surrounding cg13559233. (B) Monocyte-specific regional association results along with position of nearby CpG island (dark violet), CpG shore (yellow), CpG shelf (indigo) and CpG open sea (green yellow). cg13559233 (highlighted in shaded box) is located in CpG shore. (C) Functional annotation of cg13559233. DNase hypersensitive sites derived by DNase-seq (DNase Track) and histone marks surrounding cg13559233 (H3K4me1, H3K4me3, H3K9ac and H3K27ac Tracks) in monocytes are shown. DNase hypersensitivity, H3K4me1, H3K4me3, H3K9ac and H3K27ac histone marks are associated with active regulatory elements.


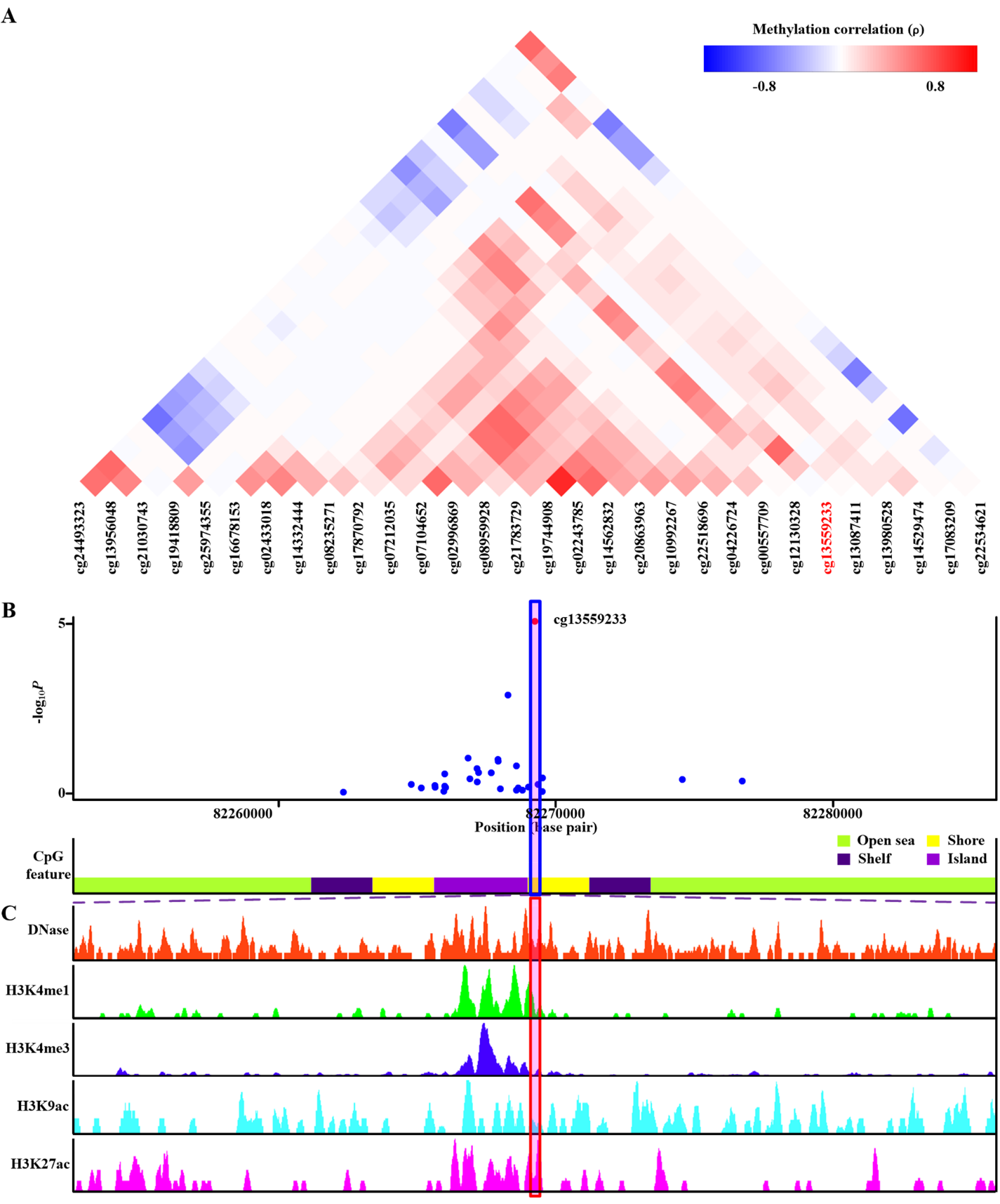


**Supplementary Figure 31. Co-methylation analysis and functional annotation of cg05638359.** (A) Patterns of co-methylation at the cytosine-phosphate-guanine dinucleotide (CpG) sites surrounding cg05638359. (B) Monocyte-specific regional association results along with position of nearby CpG island (dark violet), CpG shore (yellow), CpG shelf (indigo) and CpG open sea (green yellow). cg05638359 (highlighted in shaded box) is located in CpG open sea. (C) Functional annotation of cg05638359. DNase hypersensitive sites derived by DNase-seq (DNase Track) and histone marks surrounding cg05638359 (H3K4me1, H3K4me3, H3K9ac and H3K27ac Tracks) in monocytes are shown. DNase hypersensitivity, H3K4me1, H3K4me3, H3K9ac and H3K27ac histone marks are associated with active regulatory elements.


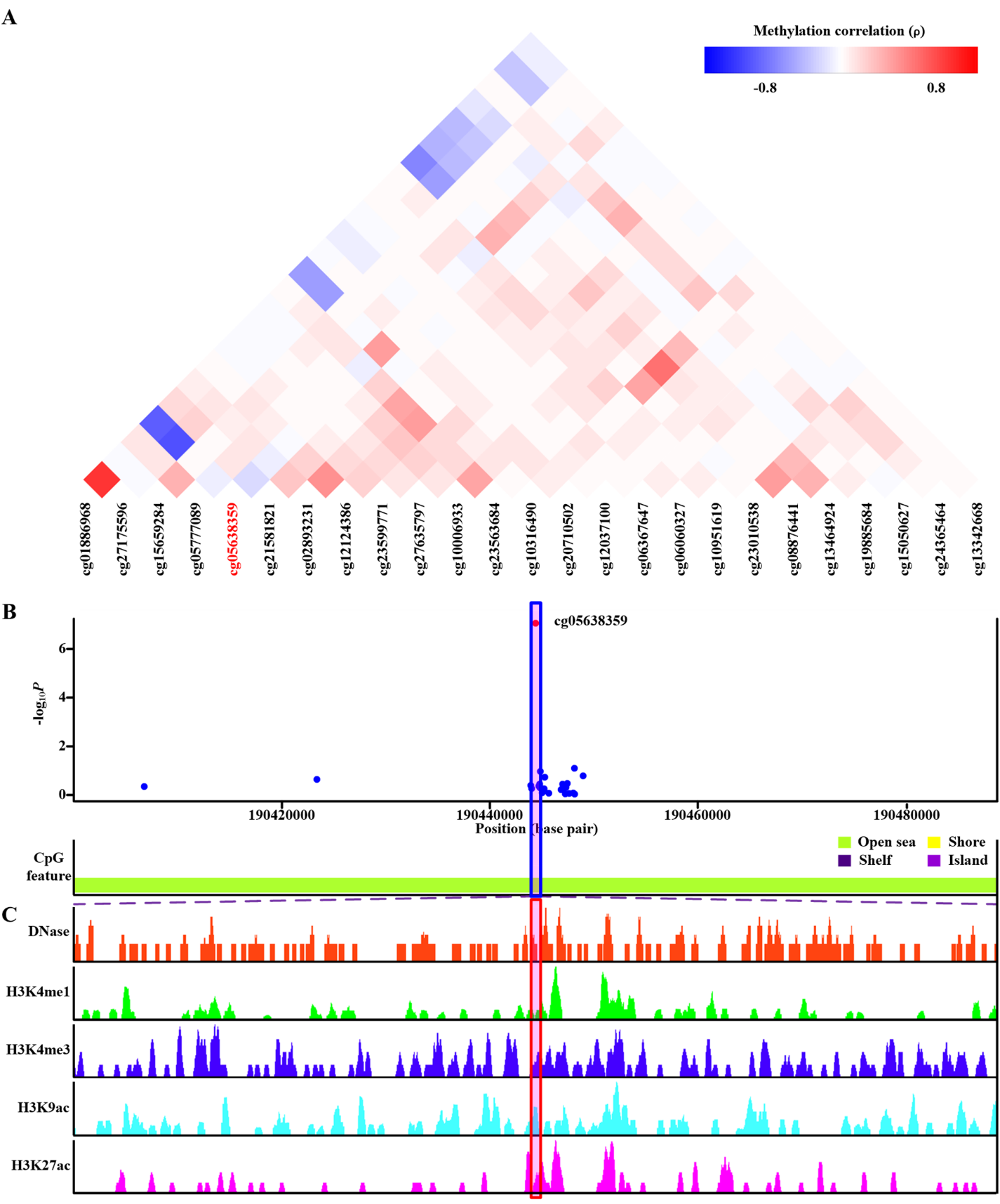


**Supplementary Figure 32. Co-methylation analysis and functional annotation of cg03945122.** (A) Patterns of co-methylation at the cytosine-phosphate-guanine dinucleotide (CpG) sites surrounding cg03945122. (B) Monocyte-specific regional association results along with position of nearby CpG island (dark violet), CpG shore (yellow), CpG shelf (indigo) and CpG open sea (green yellow). cg03945122 (highlighted in shaded box) is located in CpG shelf. (C) Functional annotation of cg03945122. DNase hypersensitive sites derived by DNase-seq (DNase Track) and histone marks surrounding cg03945122 (H3K4me1, H3K4me3, H3K9ac and H3K27ac Tracks) in monocytes are shown. DNase hypersensitivity, H3K4me1, H3K4me3, H3K9ac and H3K27ac histone marks are associated with active regulatory elements.


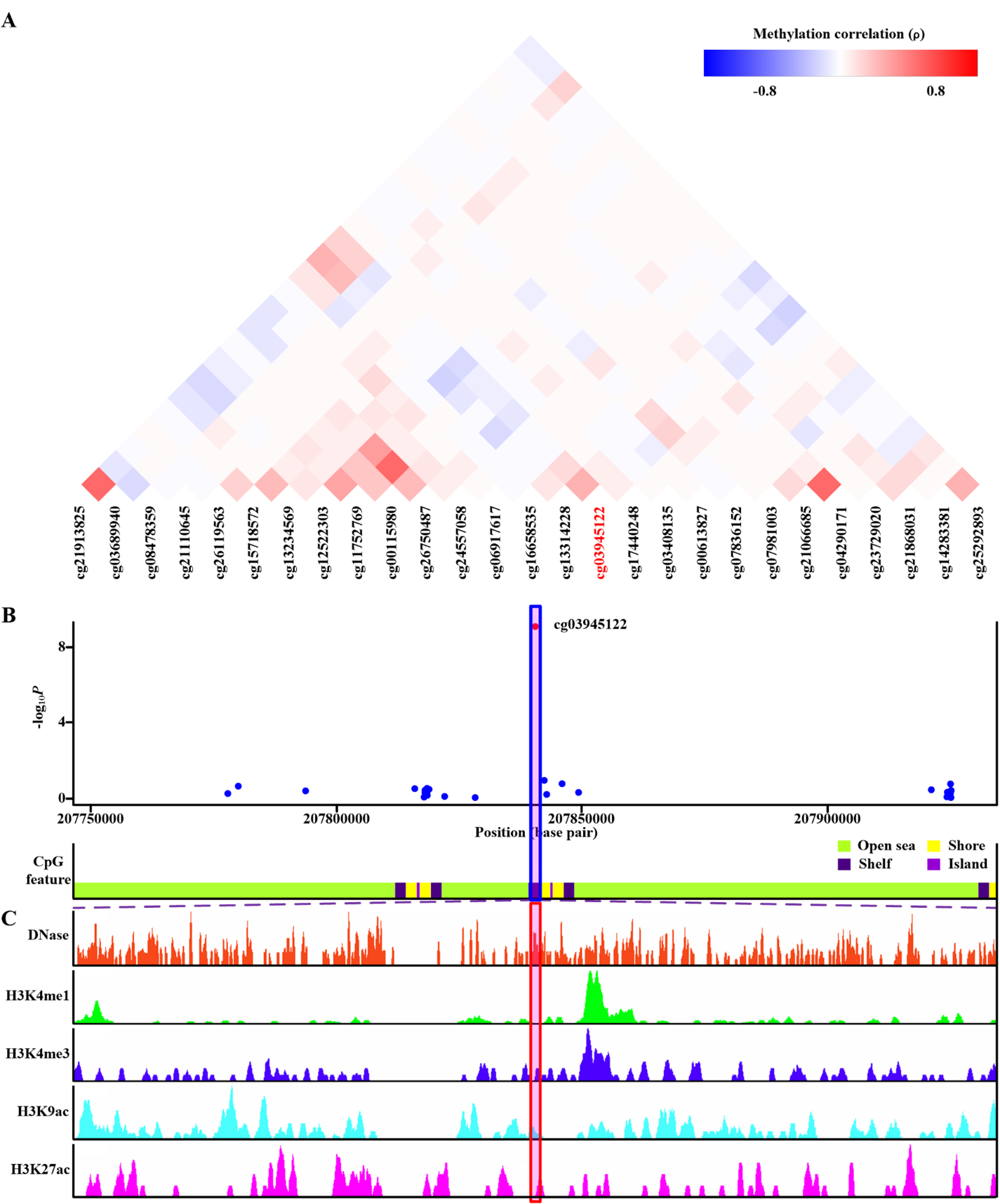


**Supplementary Figure 33. Co-methylation analysis and functional annotation of cg26461510.** (A) Patterns of co-methylation at the cytosine-phosphate-guanine dinucleotide (CpG) sites surrounding cg26461510. (B) Monocyte-specific regional association results along with position of nearby CpG island (dark violet), CpG shore (yellow), CpG shelf (indigo) and CpG open sea (green yellow). cg26461510 (highlighted in shaded box) is located in CpG open sea. (C) Functional annotation of cg26461510. DNase hypersensitive sites derived by DNase-seq (DNase Track) and histone marks surrounding cg26461510 (H3K4me1, H3K4me3, H3K9ac and H3K27ac Tracks) in monocytes are shown. DNase hypersensitivity, H3K4me1, H3K4me3, H3K9ac and H3K27ac histone marks are associated with active regulatory elements.


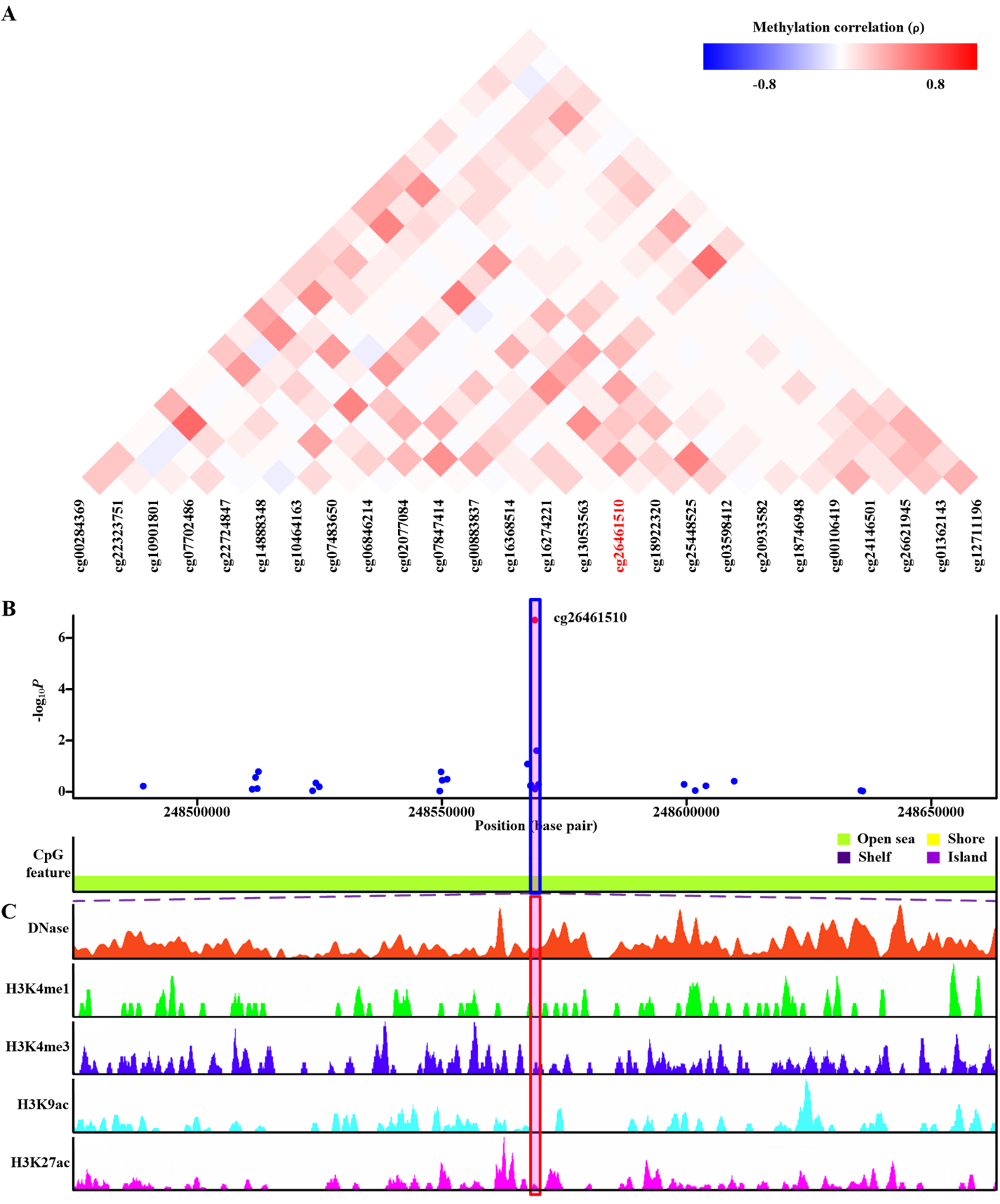


**Supplementary Figure 34. Co-methylation analysis and functional annotation of cg03275949.** (A) Patterns of co-methylation at the cytosine-phosphate-guanine dinucleotide (CpG) sites surrounding cg03275949. (B) Monocyte-specific regional association results along with position of nearby CpG island (dark violet), CpG shore (yellow), CpG shelf (indigo) and CpG open sea (green yellow). cg03275949 (highlighted in shaded box) is located in CpG island. (C) Functional annotation of cg03275949. DNase hypersensitive sites derived by DNase-seq (DNase Track) and histone marks surrounding cg03275949 (H3K4me1, H3K4me3, H3K9ac and H3K27ac Tracks) in monocytes are shown. DNase hypersensitivity, H3K4me1, H3K4me3, H3K9ac and H3K27ac histone marks are associated with active regulatory elements.


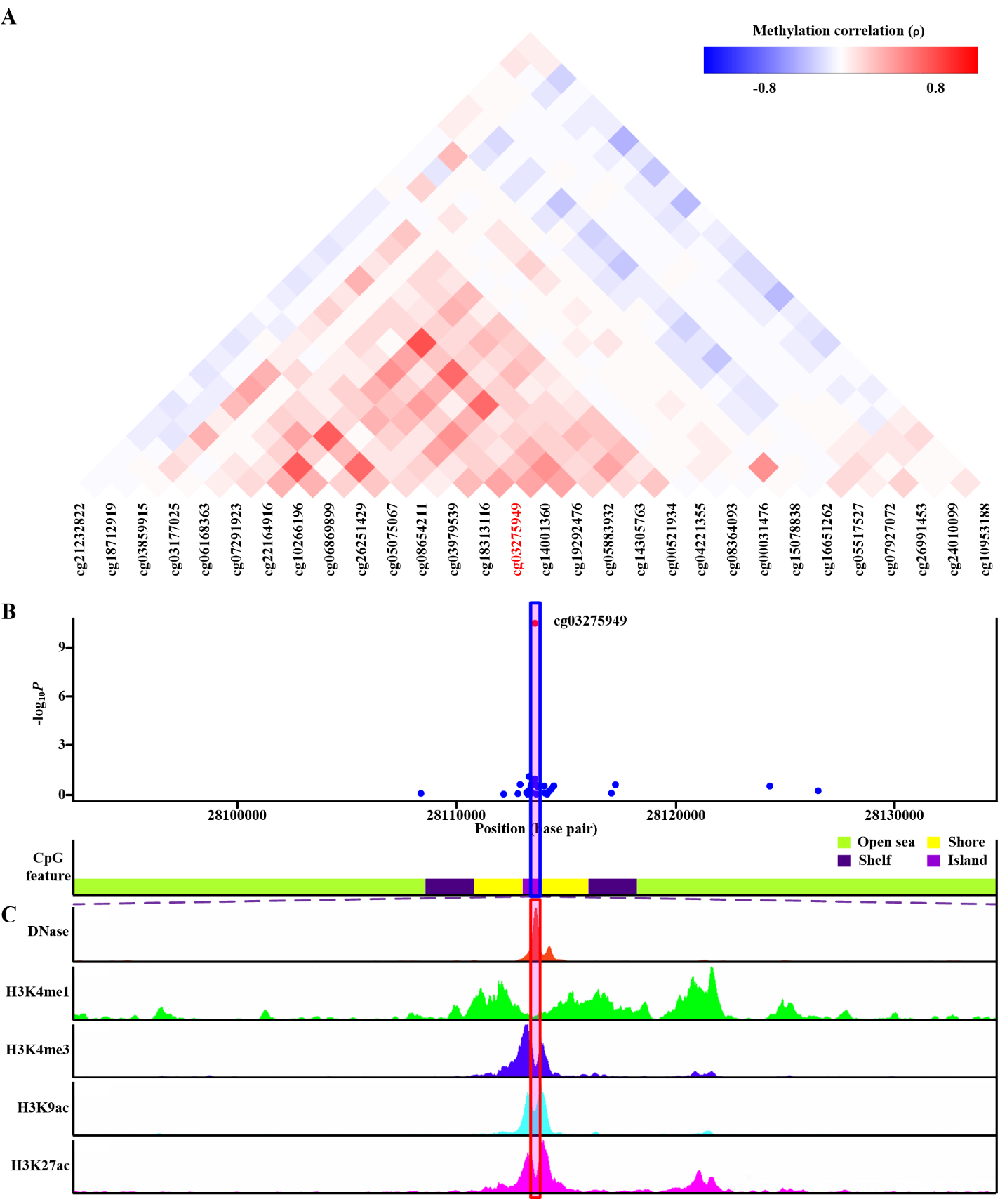


**Supplementary Figure 35. Co-methylation analysis and functional annotation of cg07083023.** (A) Patterns of co-methylation at the cytosine-phosphate-guanine dinucleotide (CpG) sites surrounding cg070830233. cg070830233 is highlighted with red while cg19824059 is highlighted with blue. (B) Monocyte-specific regional association results along with position of nearby CpG island (dark violet), CpG shore (yellow), CpG shelf (indigo) and CpG open sea (green yellow). cg070830233 (highlighted in shaded box) is located in CpG island. (C) Functional annotation of cg070830233. DNase hypersensitive sites derived by DNase-seq (DNase Track) and histone marks surrounding cg07083023 (H3K4me1, H3K4me3, H3K9ac and H3K27ac Tracks) in monocytes are shown. DNase hypersensitivity, H3K4me1, H3K4me3, H3K9ac and H3K27ac histone marks are associated with active regulatory elements.

**
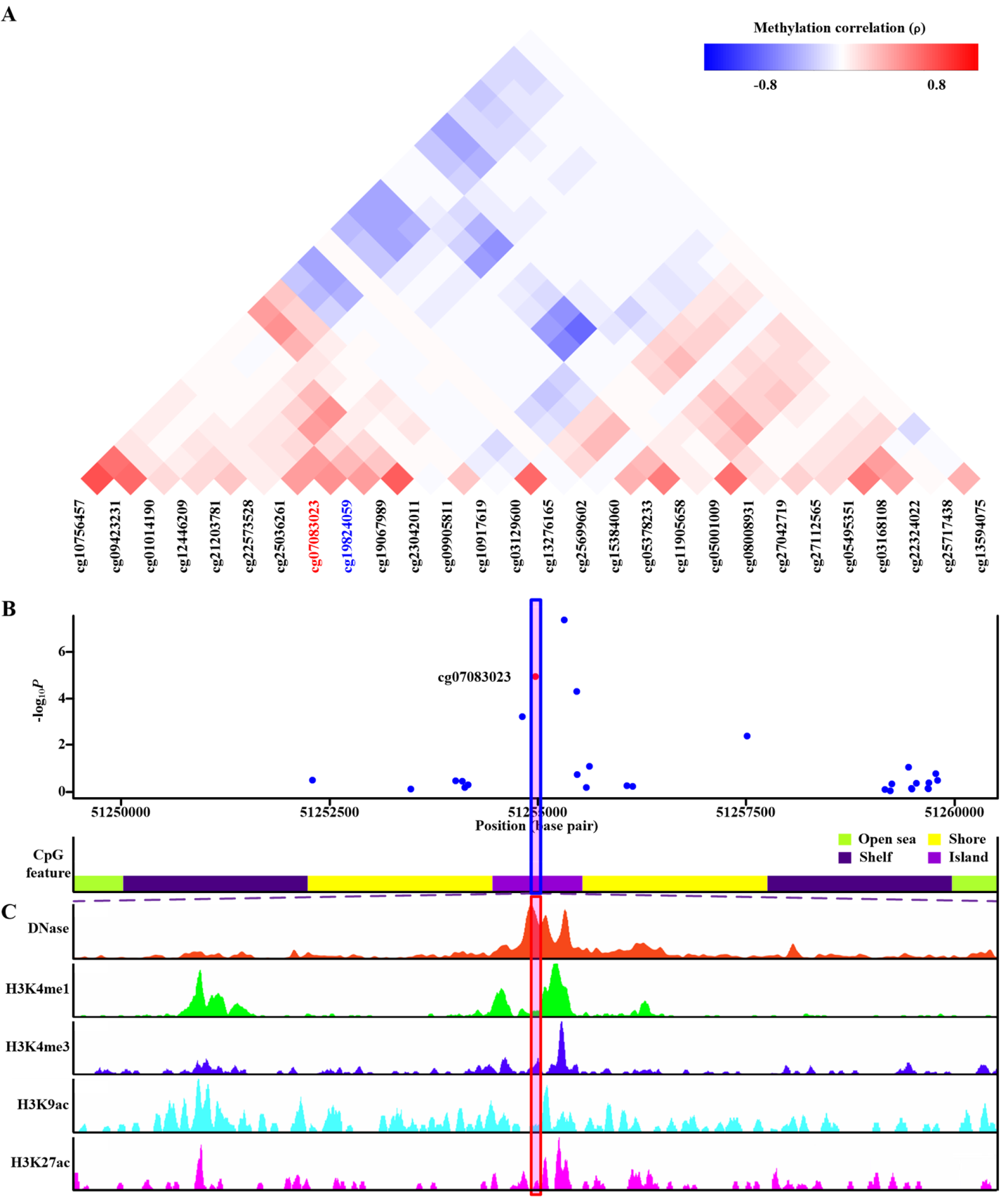
**

**Supplementary Figure 36. Co-methylation analysis and functional annotation of cg19824059.** (A) Patterns of co-methylation at the cytosine-phosphate-guanine dinucleotide (CpG) sites surrounding cg19824059. (B) Monocyte-specific regional association results along with position of nearby CpG island (dark violet), CpG shore (yellow), CpG shelf (indigo) and CpG open sea (green yellow). cg19824059 (highlighted in shaded box) is located in CpG island. (C) Functional annotation of cg19824059. DNase hypersensitive sites derived by DNase-seq (DNase Track) and histone marks surrounding cg19824059 (H3K4me1, H3K4me3, H3K9ac and H3K27ac Tracks) in monocytes are shown. DNase hypersensitivity, H3K4me1, H3K4me3, H3K9ac and H3K27ac histone marks are associated with active regulatory elements.

**
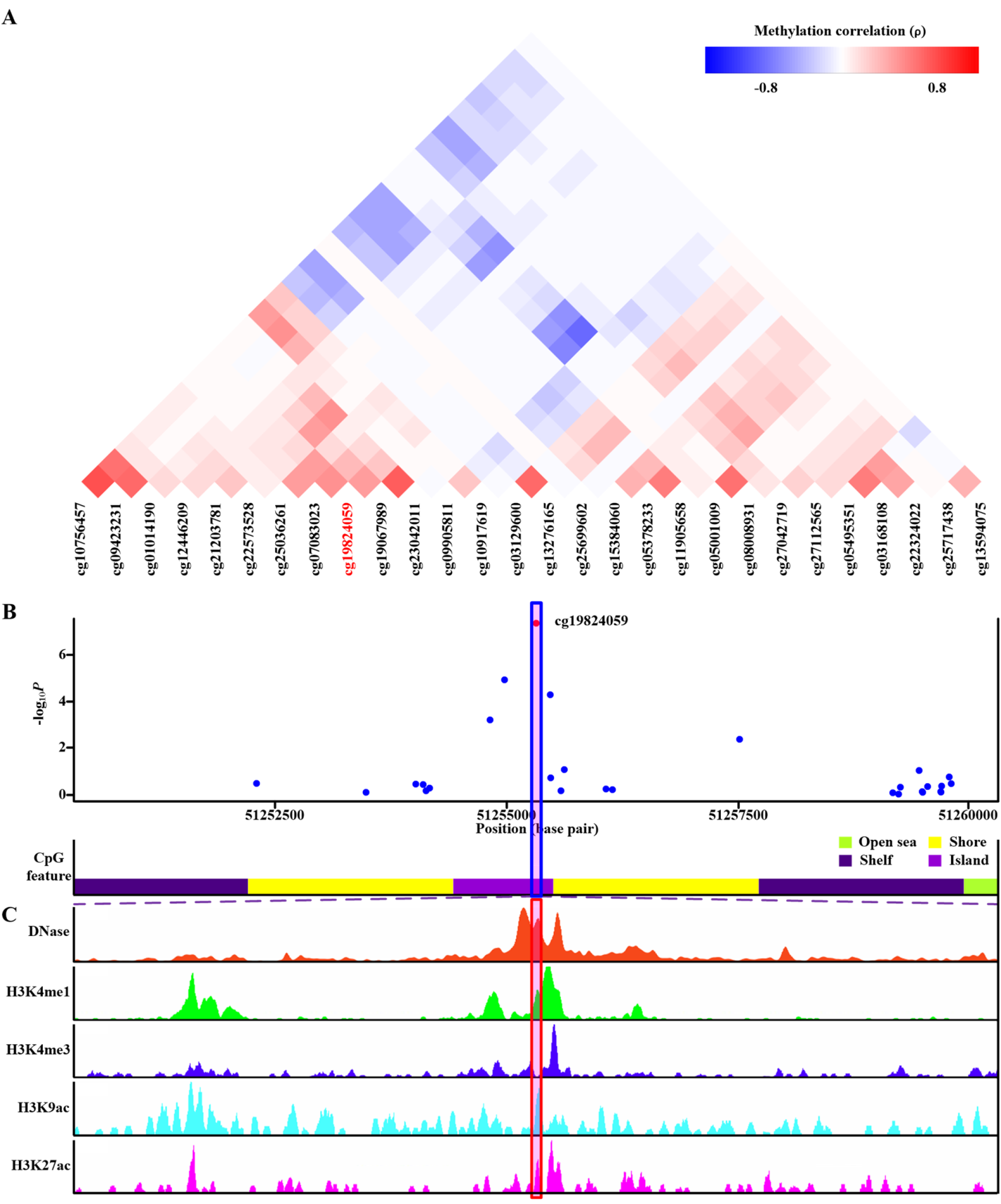
**

**Supplementary Figure 37. Co-methylation analysis and functional annotation of cg08797047.** (A) Patterns of co-methylation at the cytosine-phosphate-guanine dinucleotide (CpG) sites surrounding cg08797047. (B) Monocyte-specific regional association results along with position of nearby CpG island (dark violet), CpG shore (yellow), CpG shelf (indigo) and CpG open sea (green yellow). cg08797047 (highlighted in shaded box) is located in CpG open sea. (C) Functional annotation of cg08797047. DNase hypersensitive sites derived by DNase-seq (DNase Track) and histone marks surrounding cg08797047 (H3K4me1, H3K4me3, H3K9ac and H3K27ac Tracks) in monocytes are shown. DNase hypersensitivity, H3K4me1, H3K4me3, H3K9ac and H3K27ac histone marks are associated with active regulatory elements.

**
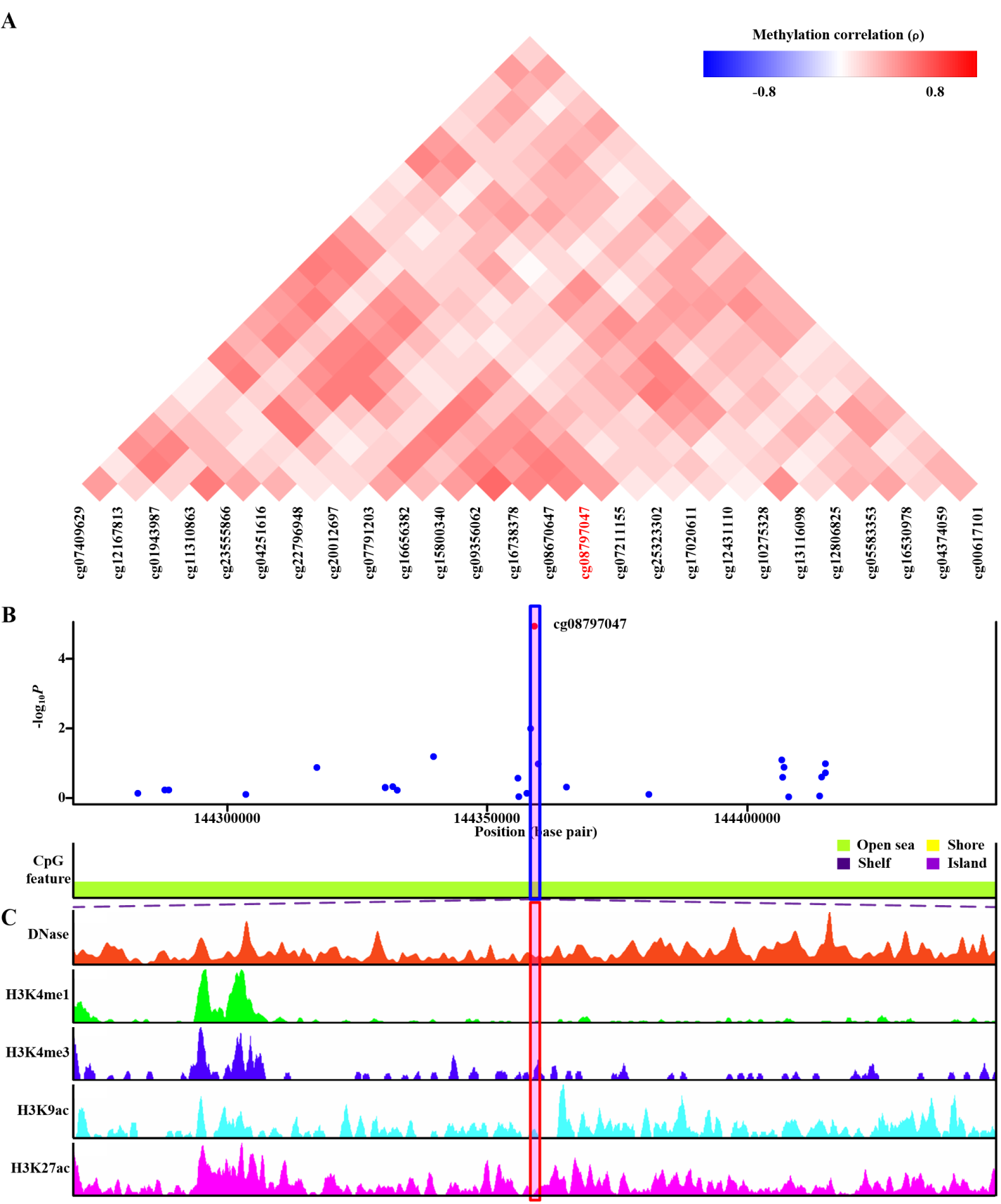
**

**Supplementary Figure 38. Co-methylation analysis and functional annotation of cg02993437.** (A) Patterns of co-methylation at the cytosine-phosphate-guanine dinucleotide (CpG) sites surrounding cg02993437. (B) Monocyte-specific regional association results along with position of nearby CpG island (dark violet), CpG shore (yellow), CpG shelf (indigo) and CpG open sea (green yellow). cg02993437 (highlighted in shaded box) is located in CpG open sea. (C) Functional annotation of cg02993437. DNase hypersensitive sites derived by DNase-seq (DNase Track) and histone marks surrounding cg02993437 (H3K4me1, H3K4me3, H3K9ac and H3K27ac Tracks) in monocytes are shown. DNase hypersensitivity, H3K4me1, H3K4me3, H3K9ac and H3K27ac histone marks are associated with active regulatory elements.

**
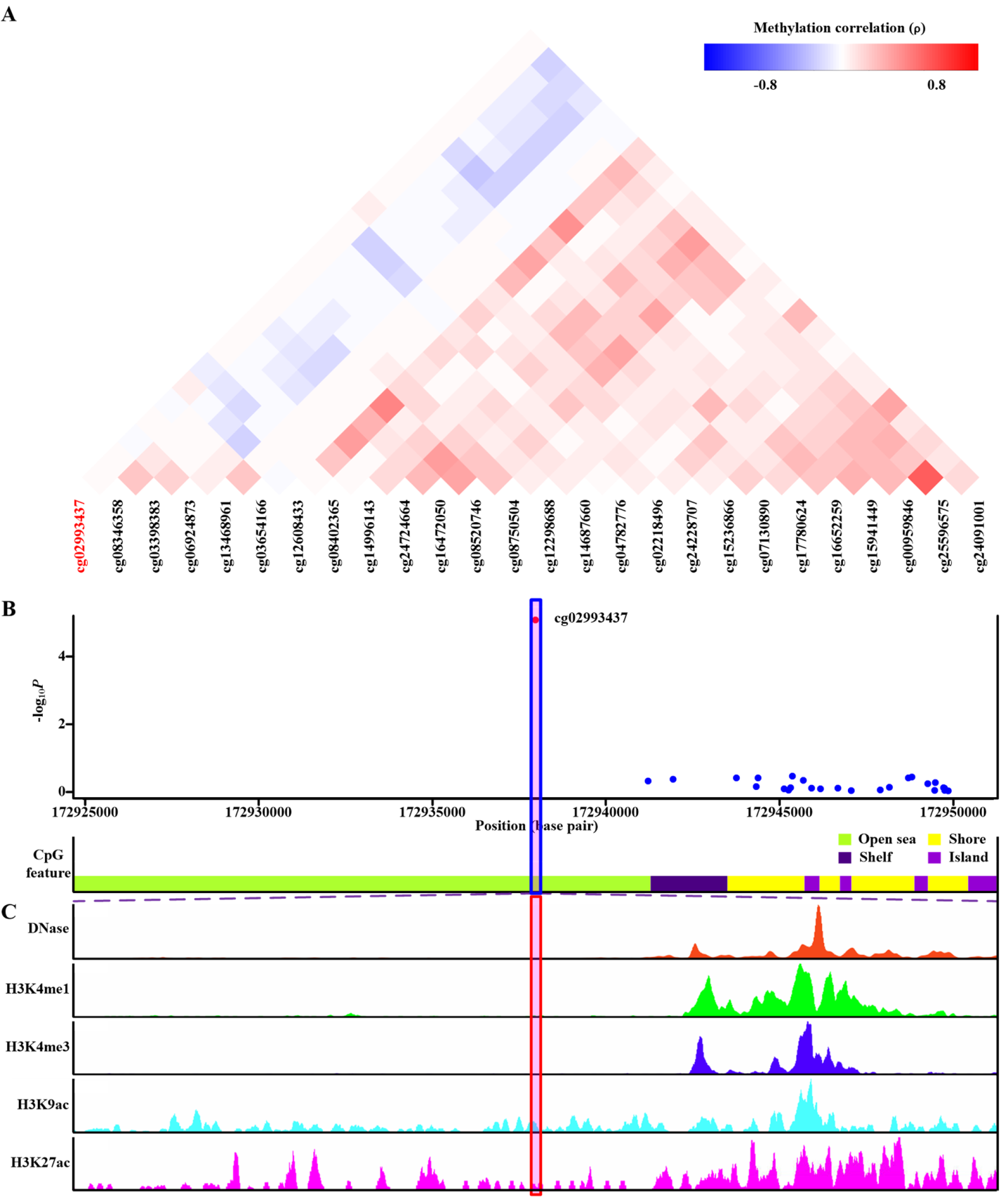
**

**Supplementary Figure 39. Co-methylation analysis and functional annotation of cg01080600.** (A) Patterns of co-methylation at the cytosine-phosphate-guanine dinucleotide (CpG) sites surrounding cg01080600. (B) Monocyte-specific regional association results along with position of nearby CpG island (dark violet), CpG shore (yellow), CpG shelf (indigo) and CpG open sea (green yellow). cg01080600 (highlighted in shaded box) is located in CpG shore. (C) Functional annotation of cg01080600. DNase hypersensitive sites derived by DNase-seq (DNase Track) and histone marks surrounding cg01080600 (H3K4me1, H3K4me3, H3K9ac and H3K27ac Tracks) in monocytes are shown. DNase hypersensitivity, H3K4me1, H3K4me3, H3K9ac and H3K27ac histone marks are associated with active regulatory elements.

**
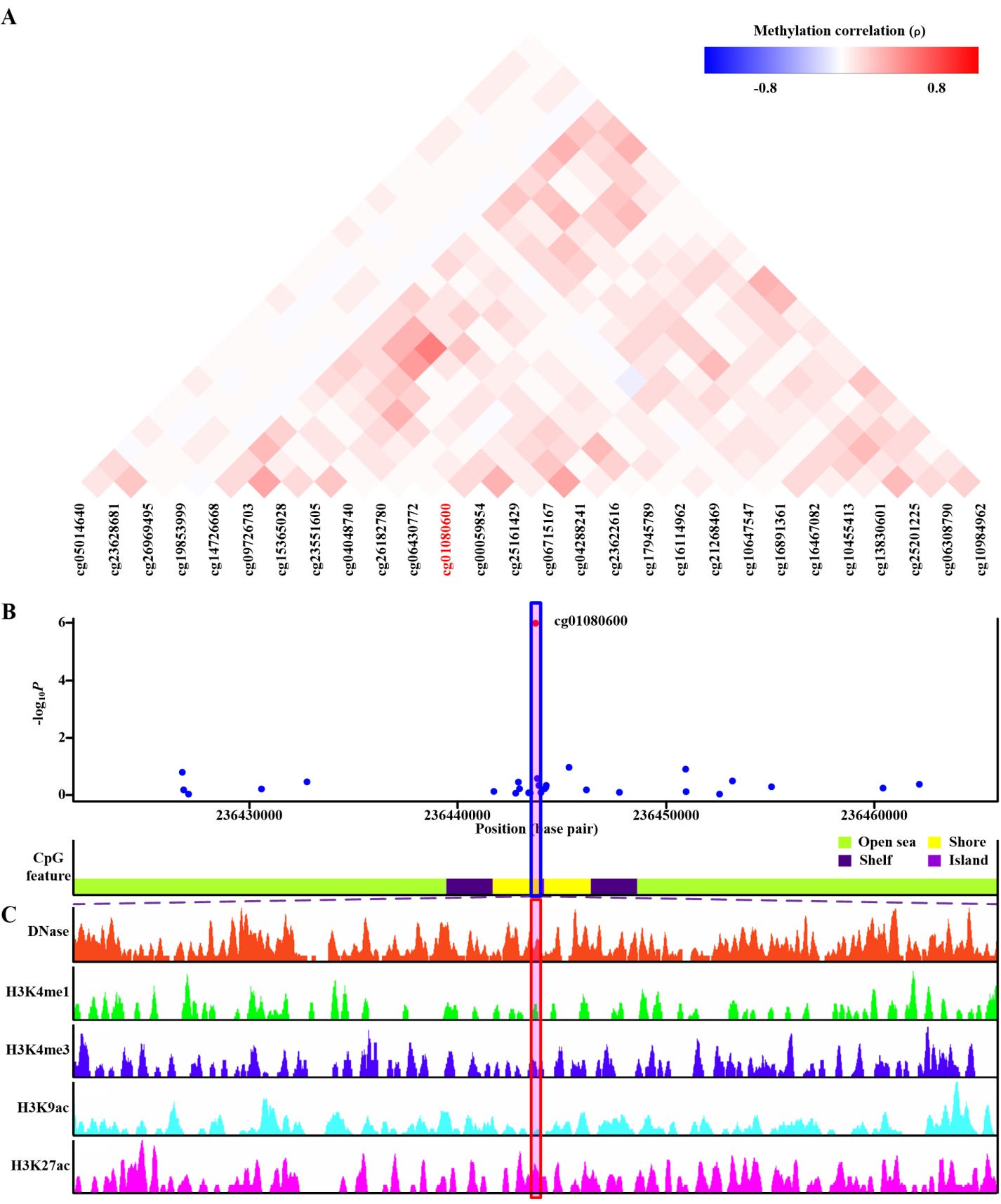
**

**Supplementary Figure 40. Co-methylation analysis and functional annotation of cg04258358.** (A) Patterns of co-methylation at the cytosine-phosphate-guanine dinucleotide (CpG) sites surrounding cg04258358. (B) Monocyte-specific regional association results along with position of nearby CpG island (dark violet), CpG shore (yellow), CpG shelf (indigo) and CpG open sea (green yellow). cg04258358 (highlighted in shaded box) is located in CpG shore. (C) Functional annotation of cg04258358. DNase hypersensitive sites derived by DNase-seq (DNase Track) and histone marks surrounding cg04258358 (H3K4me1, H3K4me3, H3K9ac and H3K27ac Tracks) in monocytes are shown. DNase hypersensitivity, H3K4me1, H3K4me3, H3K9ac and H3K27ac histone marks are associated with active regulatory elements.

**
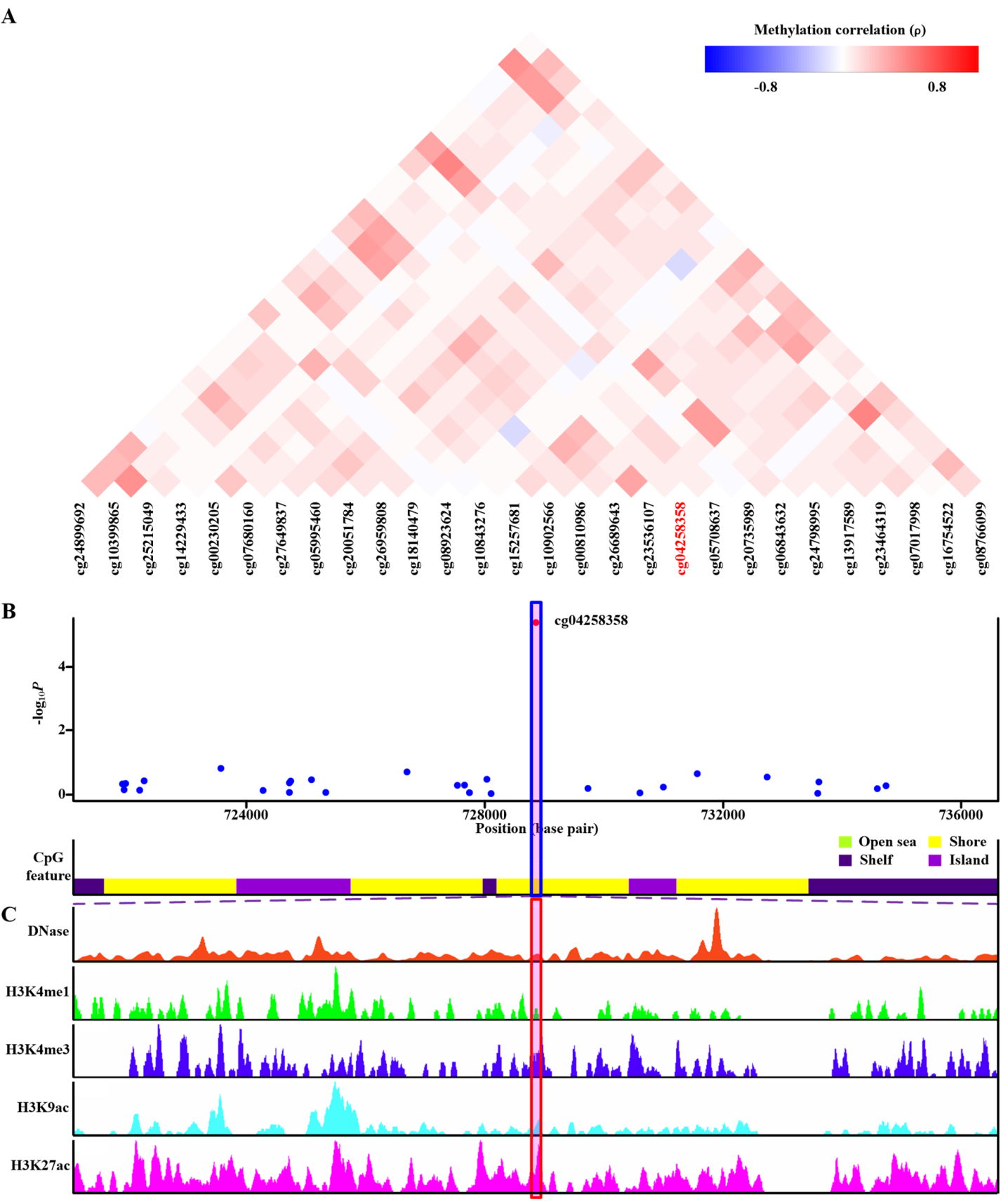
**

**Supplementary Figure 41. Co-methylation analysis and functional annotation of cg22517735.** (A) Patterns of co-methylation at the cytosine-phosphate-guanine dinucleotide (CpG) sites surrounding cg22517735. (B) Monocyte-specific regional association results along with position of nearby CpG island (dark violet), CpG shore (yellow), CpG shelf (indigo) and CpG open sea (green yellow). cg22517735 (highlighted in shaded box) is located in CpG island. (C) Functional annotation of cg22517735. DNase hypersensitive sites derived by DNase-seq (DNase Track) and histone marks surrounding cg22517735 (H3K4me1, H3K4me3, H3K9ac and H3K27ac Tracks) in monocytes are shown. DNase hypersensitivity, H3K4me1, H3K4me3, H3K9ac and H3K27ac histone marks are associated with active regulatory elements.

**
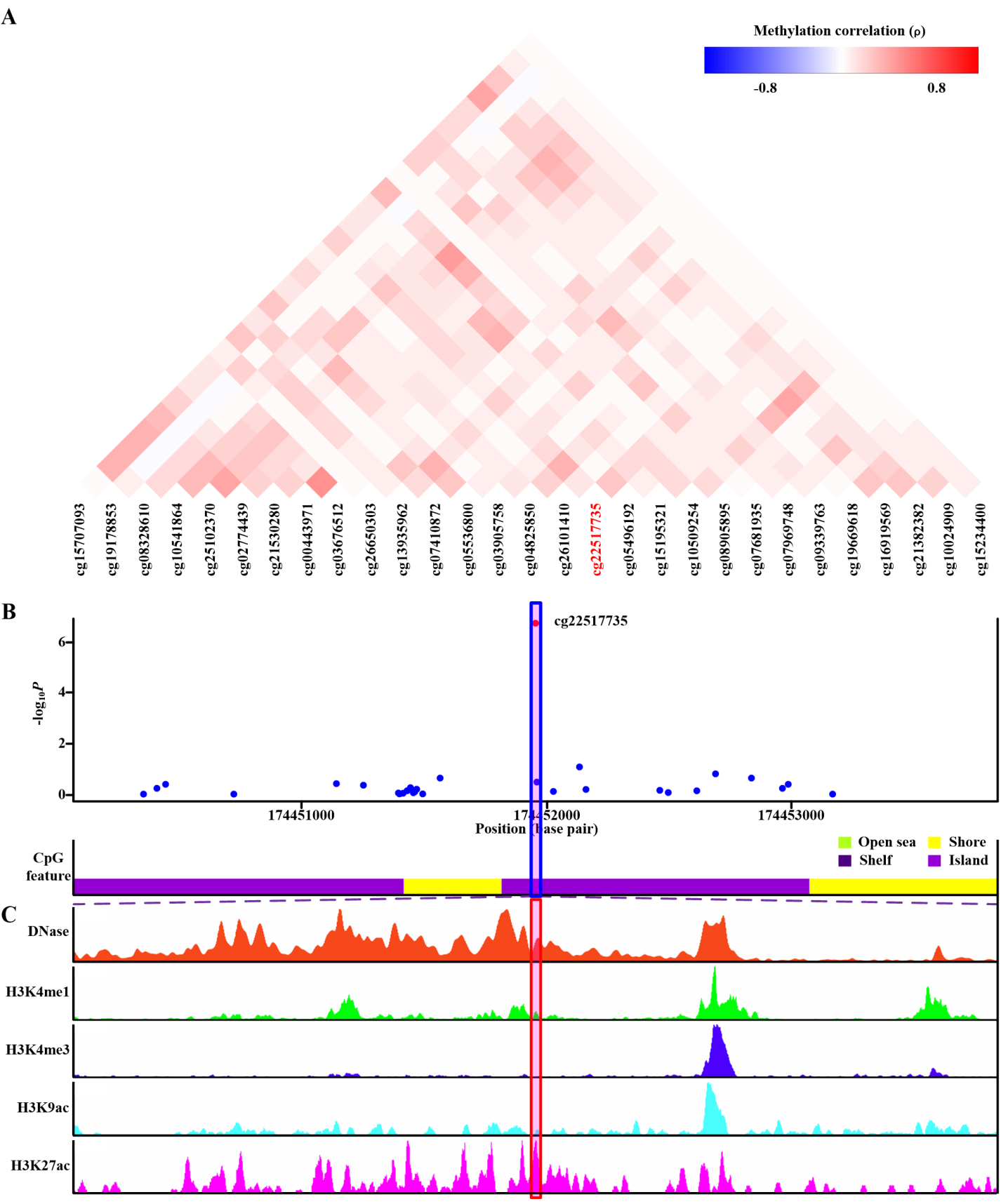
**

**Supplementary Figure 42. Co-methylation analysis and functional annotation of cg19459207.** (A) Patterns of co-methylation at the cytosine-phosphate-guanine dinucleotide (CpG) sites surrounding cg19459207. (B) Monocyte-specific regional association results along with position of nearby CpG island (dark violet), CpG shore (yellow), CpG shelf (indigo) and CpG open sea (green yellow). cg19459207 (highlighted in shaded box) is located in shore. (C) Functional annotation of cg19459207. DNase hypersensitive sites derived by DNase-seq (DNase Track) and histone marks surrounding cg19459207 (H3K4me1, H3K4me3, H3K9ac and H3K27ac Tracks) in monocytes are shown. DNase hypersensitivity, H3K4me1, H3K4me3, H3K9ac and H3K27ac histone marks are associated with active regulatory elements.

**
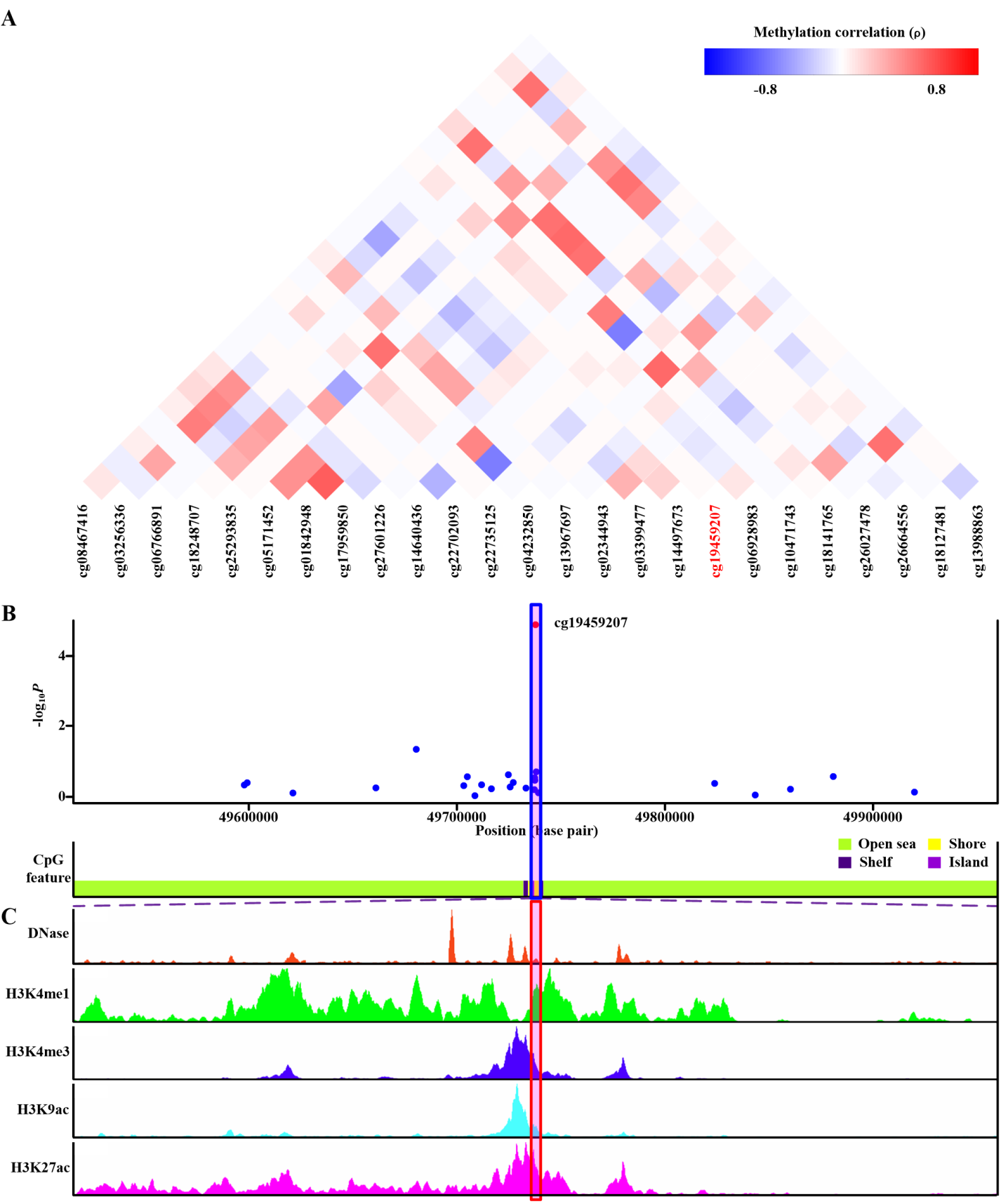
**

**Supplementary Figure 43. Co-methylation analysis and functional annotation of cg19868864.** (A) Patterns of co-methylation at the cytosine-phosphate-guanine dinucleotide (CpG) sites surrounding cg19868864. (B) Monocyte-specific regional association results along with position of nearby CpG island (dark violet), CpG shore (yellow), CpG shelf (indigo) and CpG open sea (green yellow). cg19868864 (highlighted in shaded box) is located in CpG open sea. (C) Functional annotation of cg19868864. DNase hypersensitive sites derived by DNase-seq (DNase Track) and histone marks surrounding cg19868864 (H3K4me1, H3K4me3, H3K9ac and H3K27ac Tracks) in monocytes are shown. DNase hypersensitivity, H3K4me1, H3K4me3, H3K9ac and H3K27ac histone marks are associated with active regulatory elements.

**
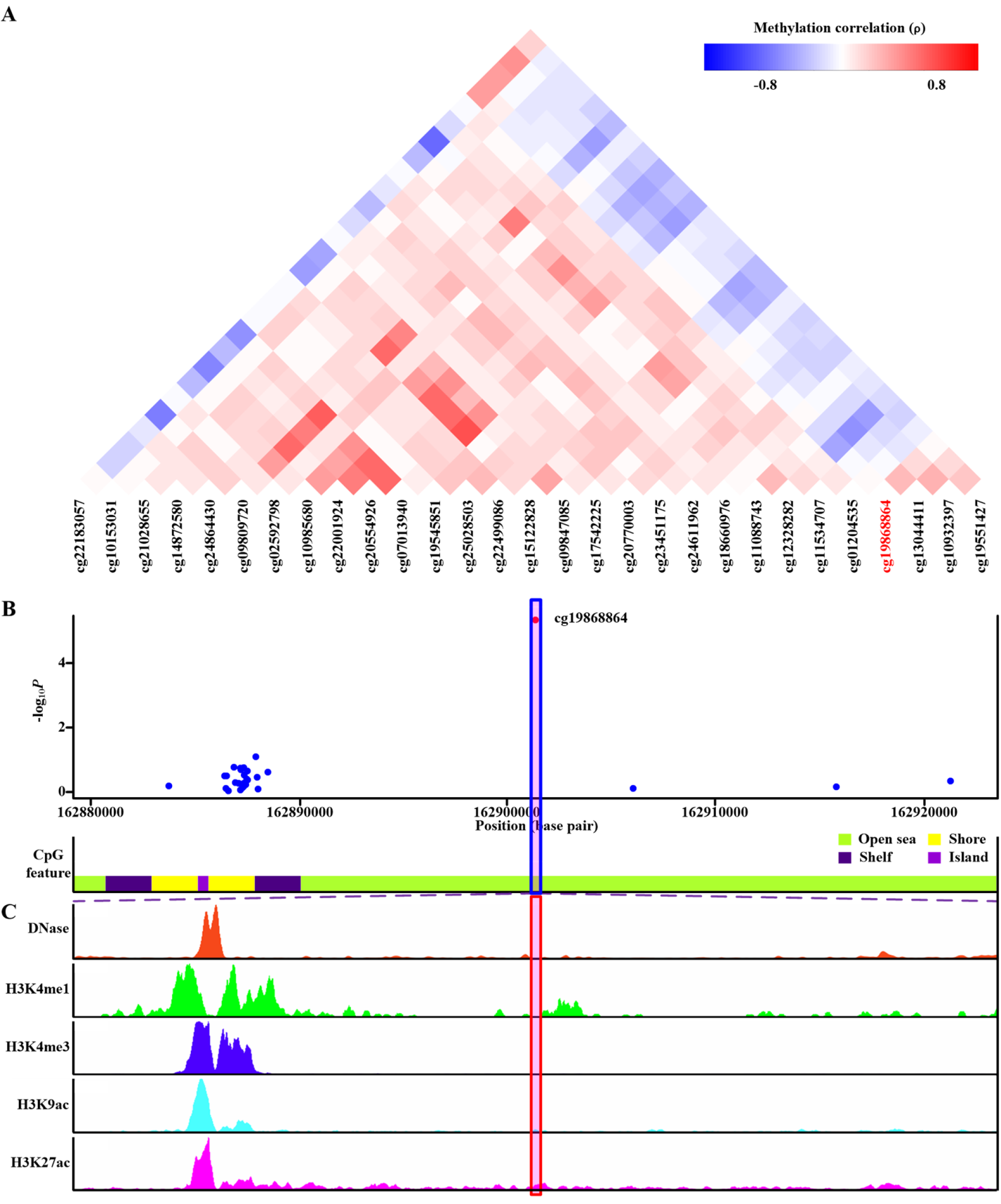
**

**Supplementary Figure 44. Co-methylation analysis and functional annotation of cg26993940** (A) Patterns of co-methylation at the cytosine-phosphate-guanine dinucleotide (CpG) sites surrounding cg26993940. (B) Monocyte-specific regional association results along with position of nearby CpG island (dark violet), CpG shore (yellow), CpG shelf (indigo) and CpG open sea (green yellow). cg26993940 (highlighted in shaded box) is located in CpG open sea. (C) Functional annotation of cg26993940. DNase hypersensitive sites derived by DNase-seq (DNase Track) and histone marks surrounding cg26993940 (H3K4me1, H3K4me3, H3K9ac and H3K27ac Tracks) in monocytes are shown. DNase hypersensitivity, H3K4me1, H3K4me3, H3K9ac and H3K27ac histone marks are associated with active regulatory elements.

**
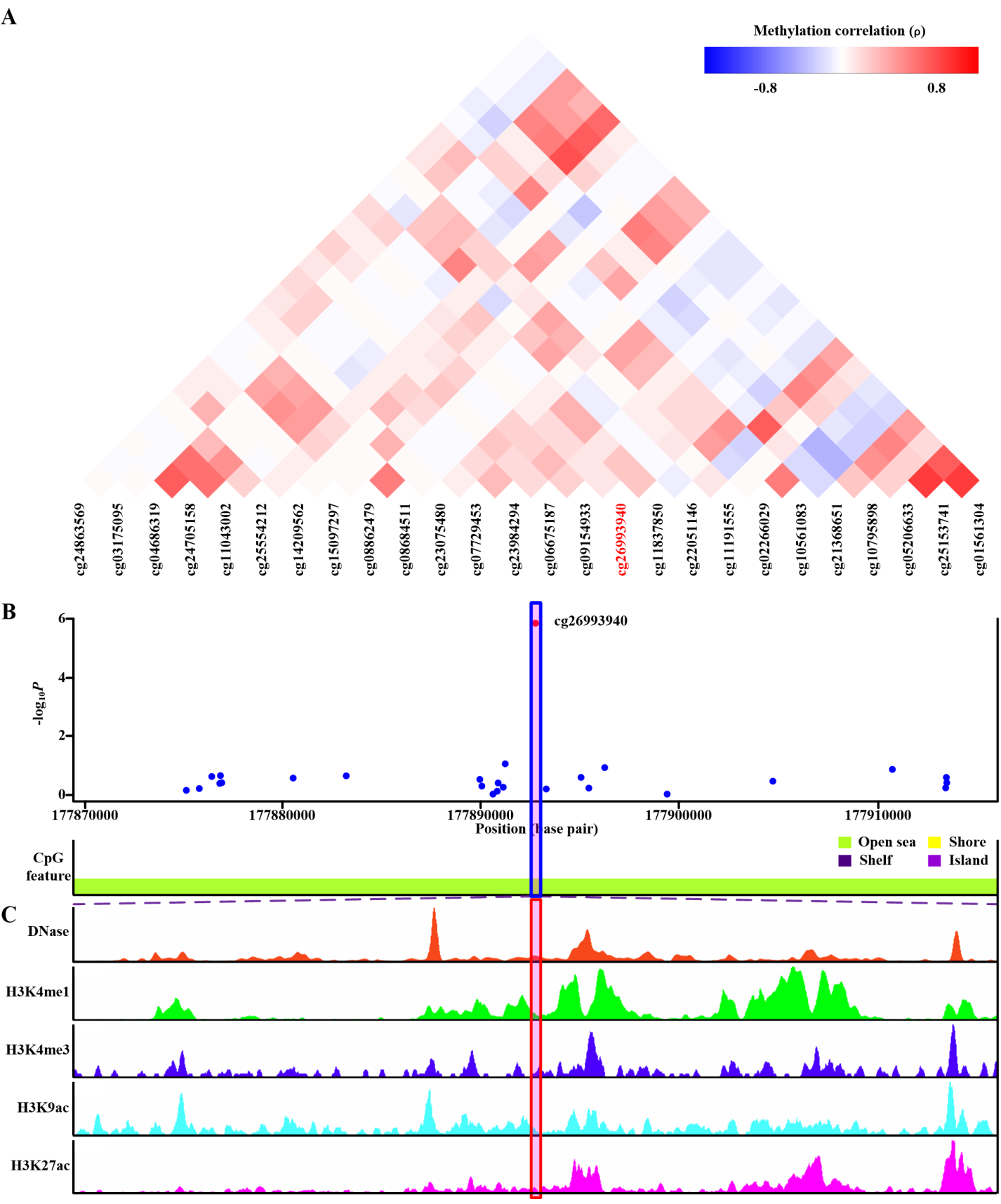
**

**Supplementary Figure 45. Co-methylation analysis and functional annotation of cg07235456.** (A) Patterns of co-methylation at the cytosine-phosphate-guanine dinucleotide (CpG) sites surrounding cg07235456. (B) Monocyte-specific regional association results along with position of nearby CpG island (dark violet), CpG shore (yellow), CpG shelf (indigo) and CpG open sea (green yellow). cg07235456 (highlighted in shaded box) is located in CpG open sea. (C) Functional annotation of cg07235456. DNase hypersensitive sites derived by DNase-seq (DNase Track) and histone marks surrounding cg07235456 (H3K4me1, H3K4me3, H3K9ac and H3K27ac Tracks) in monocytes are shown. DNase hypersensitivity, H3K4me1, H3K4me3, H3K9ac and H3K27ac histone marks are associated with active regulatory elements.

**
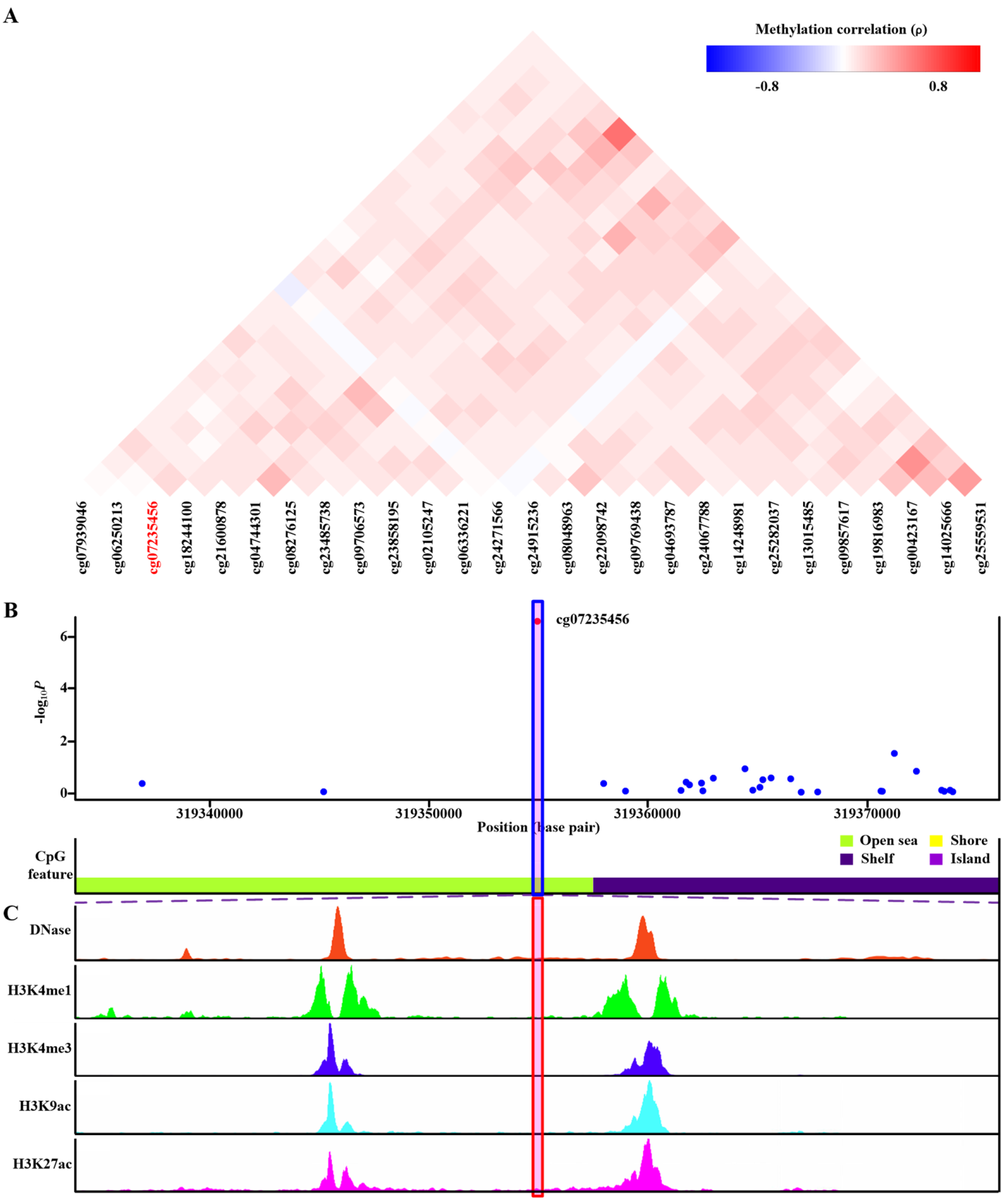
**

**Supplementary Figure 46. Co-methylation analysis and functional annotation of cg24070123.** (A) Patterns of co-methylation at the cytosine-phosphate-guanine dinucleotide (CpG) sites surrounding cg24070123. (B) Monocyte-specific regional association results along with position of nearby CpG island (dark violet), CpG shore (yellow), CpG shelf (indigo) and CpG open sea (green yellow). cg24070123 (highlighted in shaded box) is located in CpG shore. (C) Functional annotation of cg24070123. DNase hypersensitive sites derived by DNase-seq (DNase Track) and histone marks surrounding cg24070123 (H3K4me1, H3K4me3, H3K9ac and H3K27ac Tracks) in monocytes are shown. DNase hypersensitivity, H3K4me1, H3K4me3, H3K9ac and H3K27ac histone marks are associated with active regulatory elements.

**
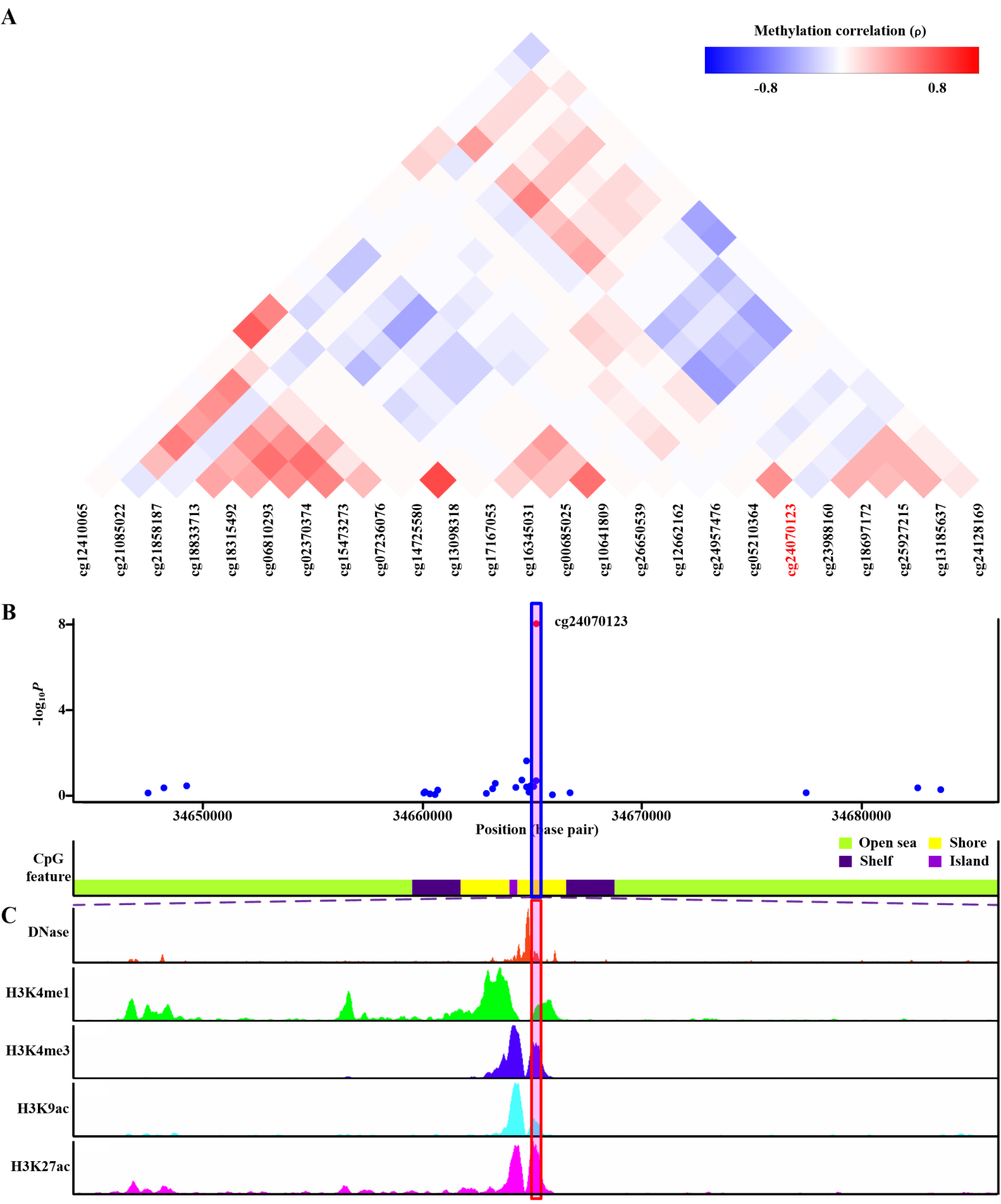
**

**Supplementary Figure 47. Co-methylation analysis and functional annotation of cg27478167.** (A) Patterns of co-methylation at the cytosine-phosphate-guanine dinucleotide (CpG) sites surrounding cg27478167. (B) Monocyte-specific regional association results along with position of nearby CpG island (dark violet), CpG shore (yellow), CpG shelf (indigo) and CpG open sea (green yellow). cg27478167 (highlighted in shaded box) is located in CpG shelf. (C) Functional annotation of cg27478167. DNase hypersensitive sites derived by DNase-seq (DNase Track) and histone marks surrounding cg27478167 (H3K4me1, H3K4me3, H3K9ac and H3K27ac Tracks) in monocytes are shown. DNase hypersensitivity, H3K4me1, H3K4me3, H3K9ac and H3K27ac histone marks are associated with active regulatory elements.

**
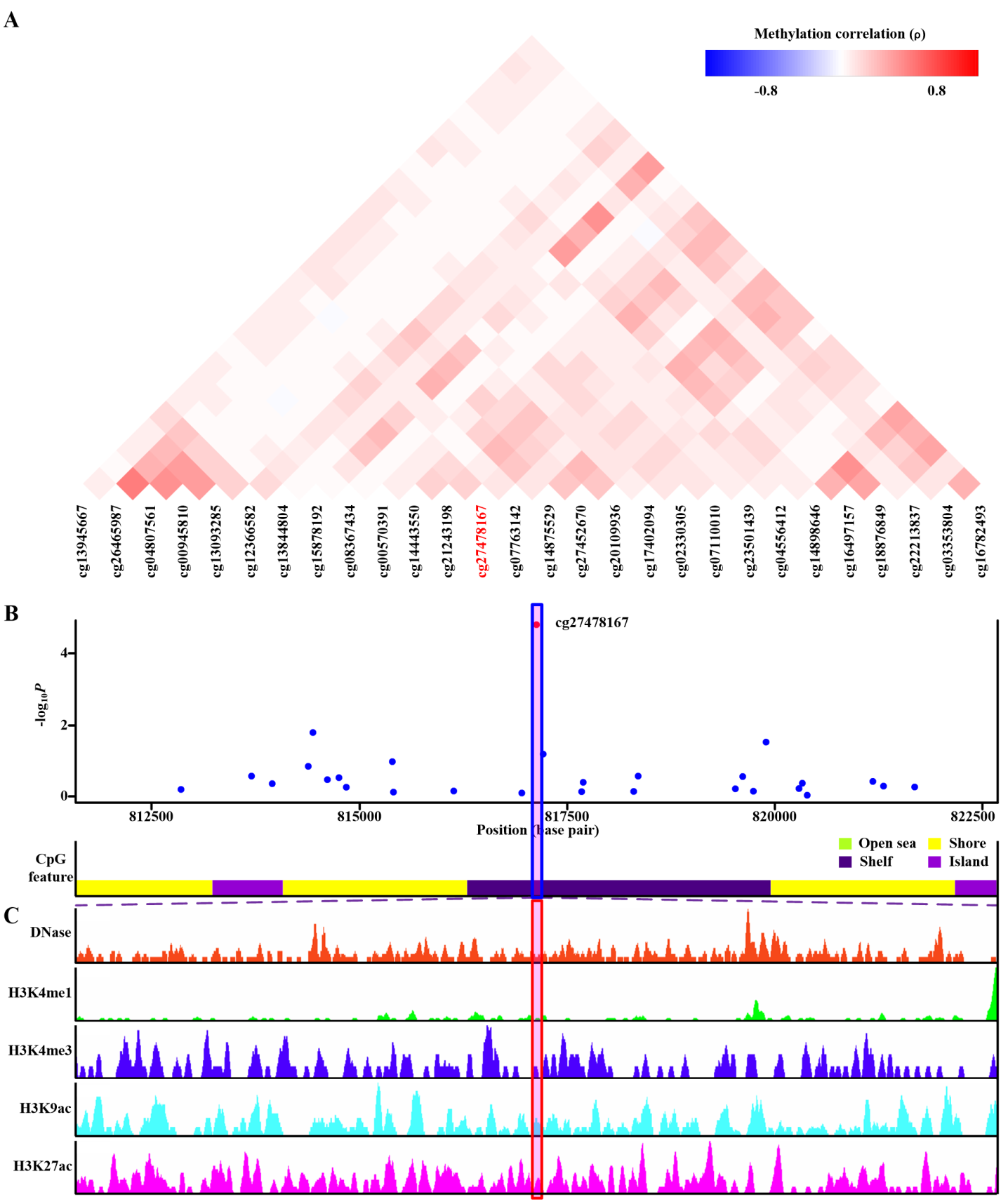
**

**Supplementary Figure 48. Co-methylation analysis and functional annotation of cg05096788.** (A) Patterns of co-methylation at the cytosine-phosphate-guanine dinucleotide (CpG) sites surrounding cg05096788. (B) Monocyte-specific regional association results along with position of nearby CpG island (dark violet), CpG shore (yellow), CpG shelf (indigo) and CpG open sea (green yellow). cg05096788 (highlighted in shaded box) is located in CpG open sea. (C) Functional annotation of cg05096788. DNase hypersensitive sites derived by DNase-seq (DNase Track) and histone marks surrounding cg05096788 (H3K4me1, H3K4me3, H3K9ac and H3K27ac Tracks) in monocytes are shown. DNase hypersensitivity, H3K4me1, H3K4me3, H3K9ac and H3K27ac histone marks are associated with active regulatory elements.

**
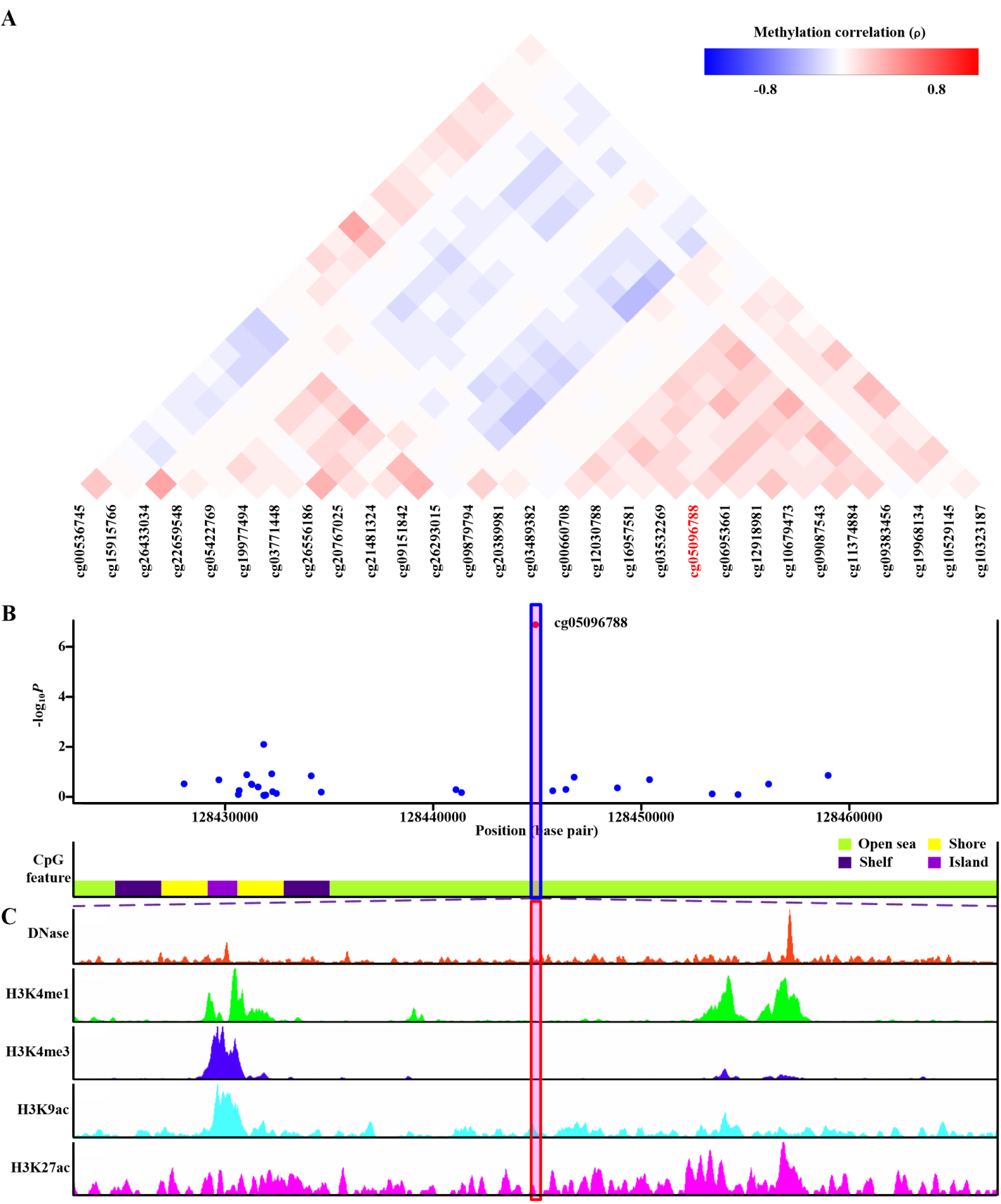
**

**Supplementary Figure 49. Co-methylation analysis and functional annotation of cg11768833.** (A) Patterns of co-methylation at the cytosine-phosphate-guanine dinucleotide (CpG) sites surrounding cg11768833. (B) Monocyte-specific regional association results along with position of nearby CpG island (dark violet), CpG shore (yellow), CpG shelf (indigo) and CpG open sea (green yellow). cg11768833 (highlighted in shaded box) is located in CpG open sea. (C) Functional annotation of cg11768833. DNase hypersensitive sites derived by DNase-seq (DNase Track) and histone marks surrounding cg11768833 (H3K4me1, H3K4me3, H3K9ac and H3K27ac Tracks) in monocytes are shown. DNase hypersensitivity, H3K4me1, H3K4me3, H3K9ac and H3K27ac histone marks are associated with active regulatory elements.

**
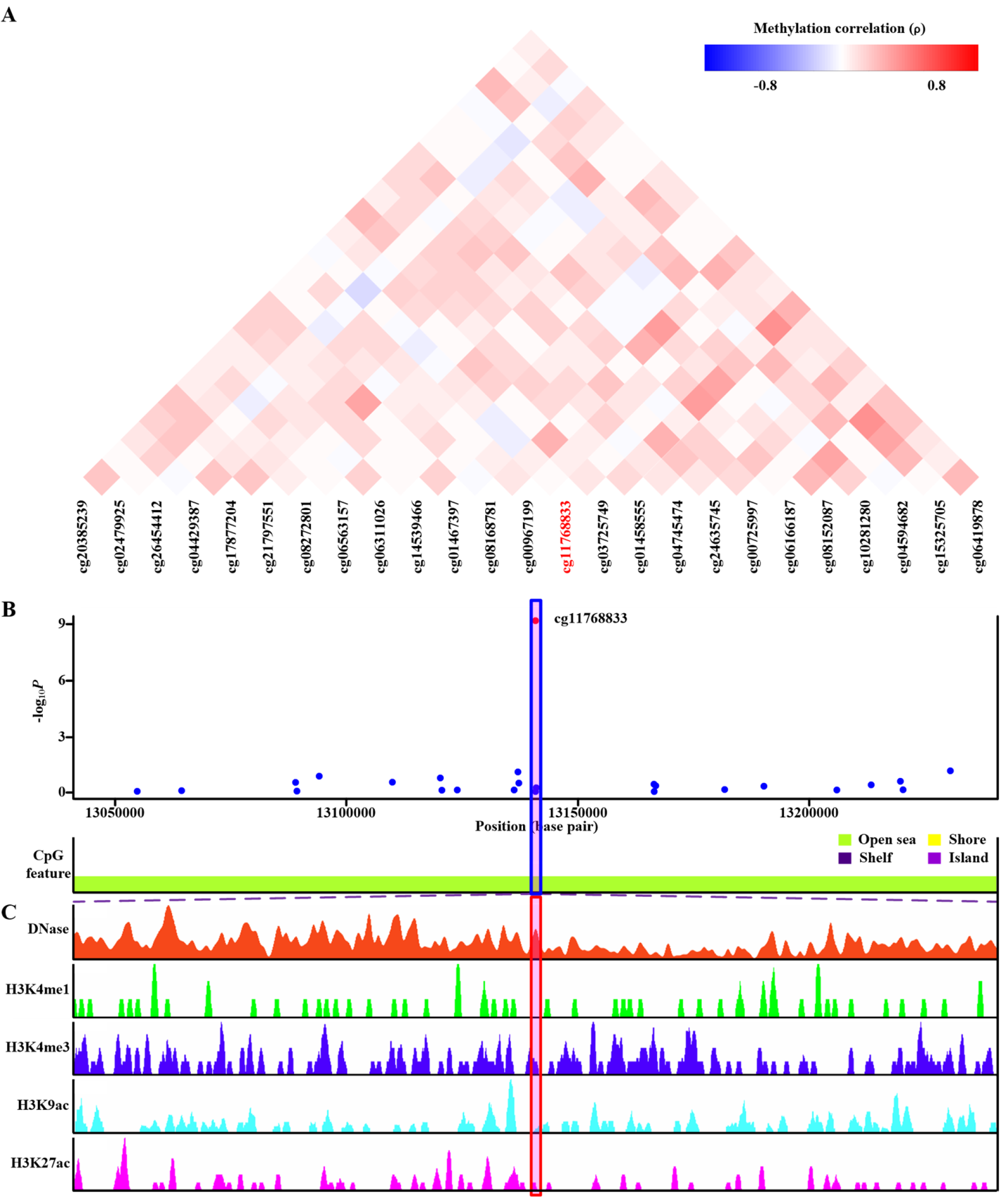
**

**Supplementary Figure 50. Co-methylation analysis and functional annotation of cg09762316.** (A) Patterns of co-methylation at the cytosine-phosphate-guanine dinucleotide (CpG) sites surrounding cg09762316. (B) Monocyte-specific regional association results along with position of nearby CpG island (dark violet), CpG shore (yellow), CpG shelf (indigo) and CpG open sea (green yellow). cg09762316 (highlighted in shaded box) is located in CpG shore. (C) Functional annotation of cg09762316. DNase hypersensitive sites derived by DNase-seq (DNase Track) and histone marks surrounding cg09762316 (H3K4me1, H3K4me3, H3K9ac and H3K27ac Tracks) in monocytes are shown. DNase hypersensitivity, H3K4me1, H3K4me3, H3K9ac and H3K27ac histone marks are associated with active regulatory elements.

**
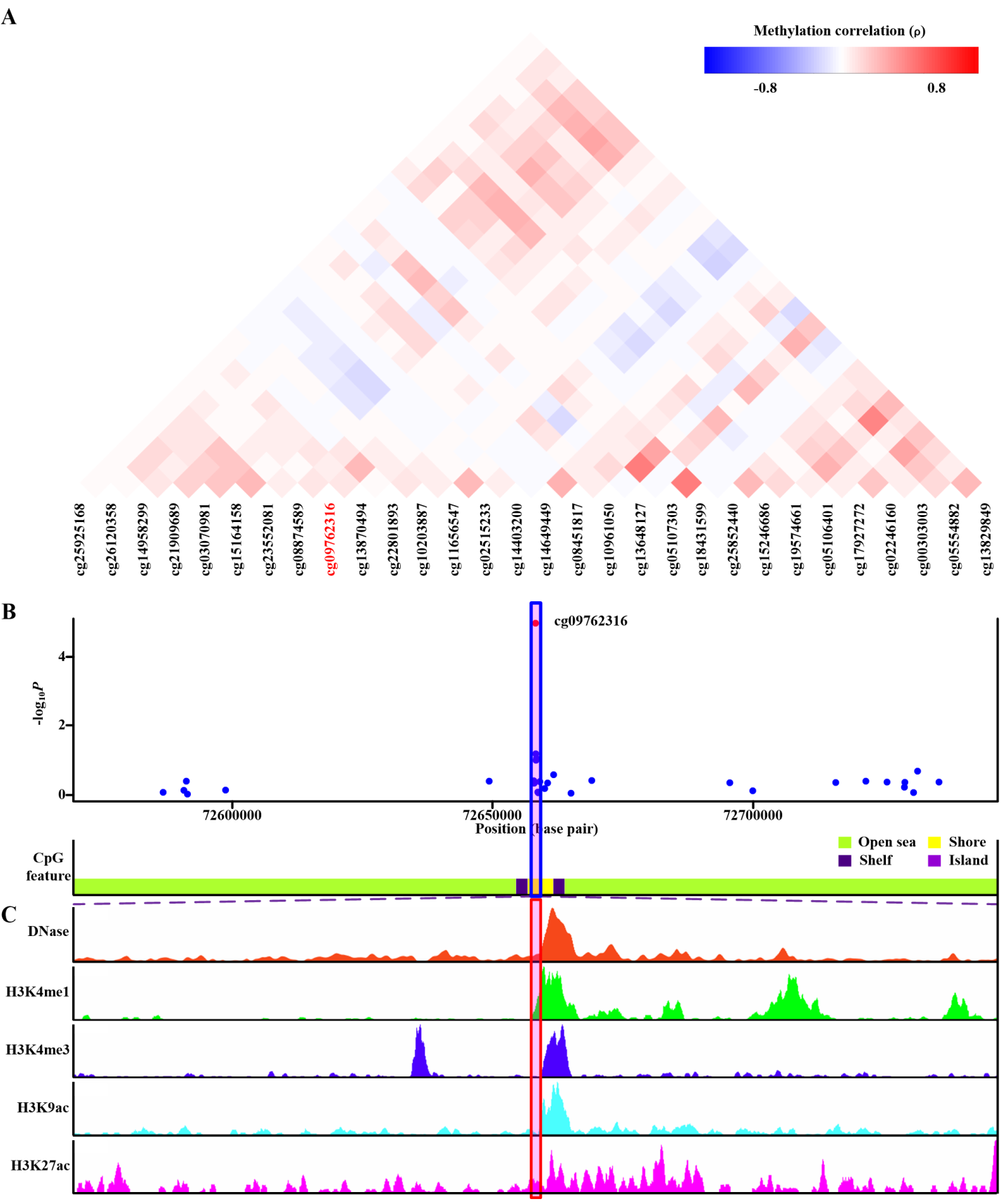
**

**Supplementary Figure 51. Co-methylation analysis and functional annotation of cg00748492.** (A) Patterns of co-methylation at the cytosine-phosphate-guanine dinucleotide (CpG) sites surrounding cg00748492. cg00748492 displays co-methylation with cg01904227 (ρ=0.81) (highlighted with purple) and cg26544725 (ρ=0.81) (highlighted with green) (B) Monocyte-specific regional association results along with position of nearby CpG island (dark violet), CpG shore (yellow), CpG shelf (indigo) and CpG open sea (green yellow). cg00748492 (highlighted in shaded box) is located in CpG open sea. Co-methylated CpG sites (cg01904227 and cg26544725) are highlighted with purple and green, respectively. (C) Functional annotation of cg00748492. DNase hypersensitive sites derived by DNase-seq (DNase Track) and histone marks surrounding cg00748492 (H3K4me1, H3K4me3, H3K9ac and H3K27ac Tracks) in monocytes are shown. DNase hypersensitivity, H3K4me1, H3K4me3, H3K9ac and H3K27ac histone marks are associated with active regulatory elements.

**
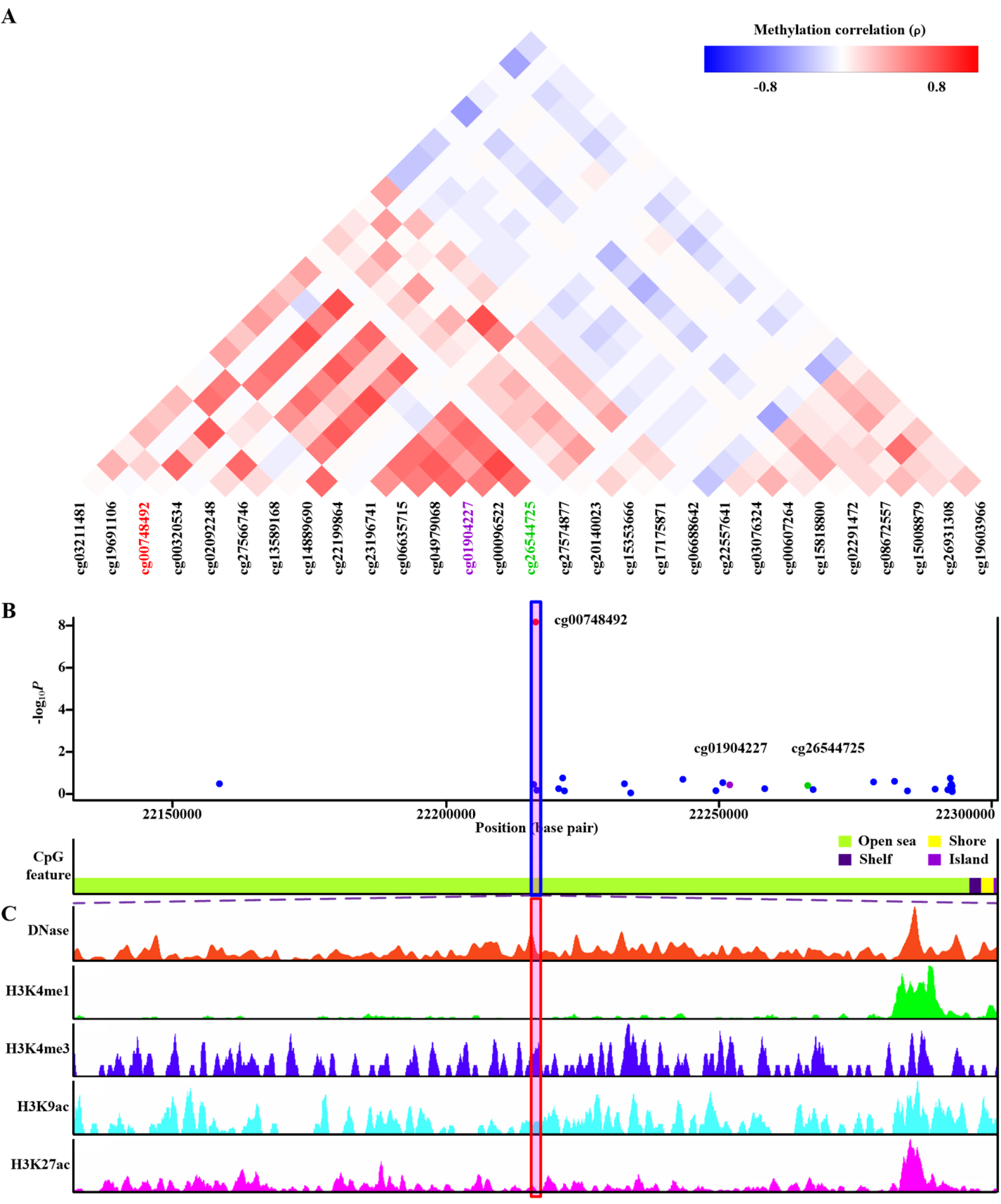
**

**Supplementary Figure 52. Co-methylation analysis and functional annotation of cg25692425.** (A) Patterns of co-methylation at the cytosine-phosphate-guanine dinucleotide (CpG) sites surrounding cg25692425. (B) Monocyte-specific regional association results along with position of nearby CpG island (dark violet), CpG shore (yellow), CpG shelf (indigo) and CpG open sea (green yellow). cg25692425 (highlighted in shaded box) is located in CpG open sea. (C) Functional annotation of cg25692425. DNase hypersensitive sites derived by DNase-seq (DNase Track) and histone marks surrounding cg25692425 (H3K4me1, H3K4me3, H3K9ac and H3K27ac Tracks) in monocytes are shown. DNase hypersensitivity, H3K4me1, H3K4me3, H3K9ac and H3K27ac histone marks are associated with active regulatory elements.

**
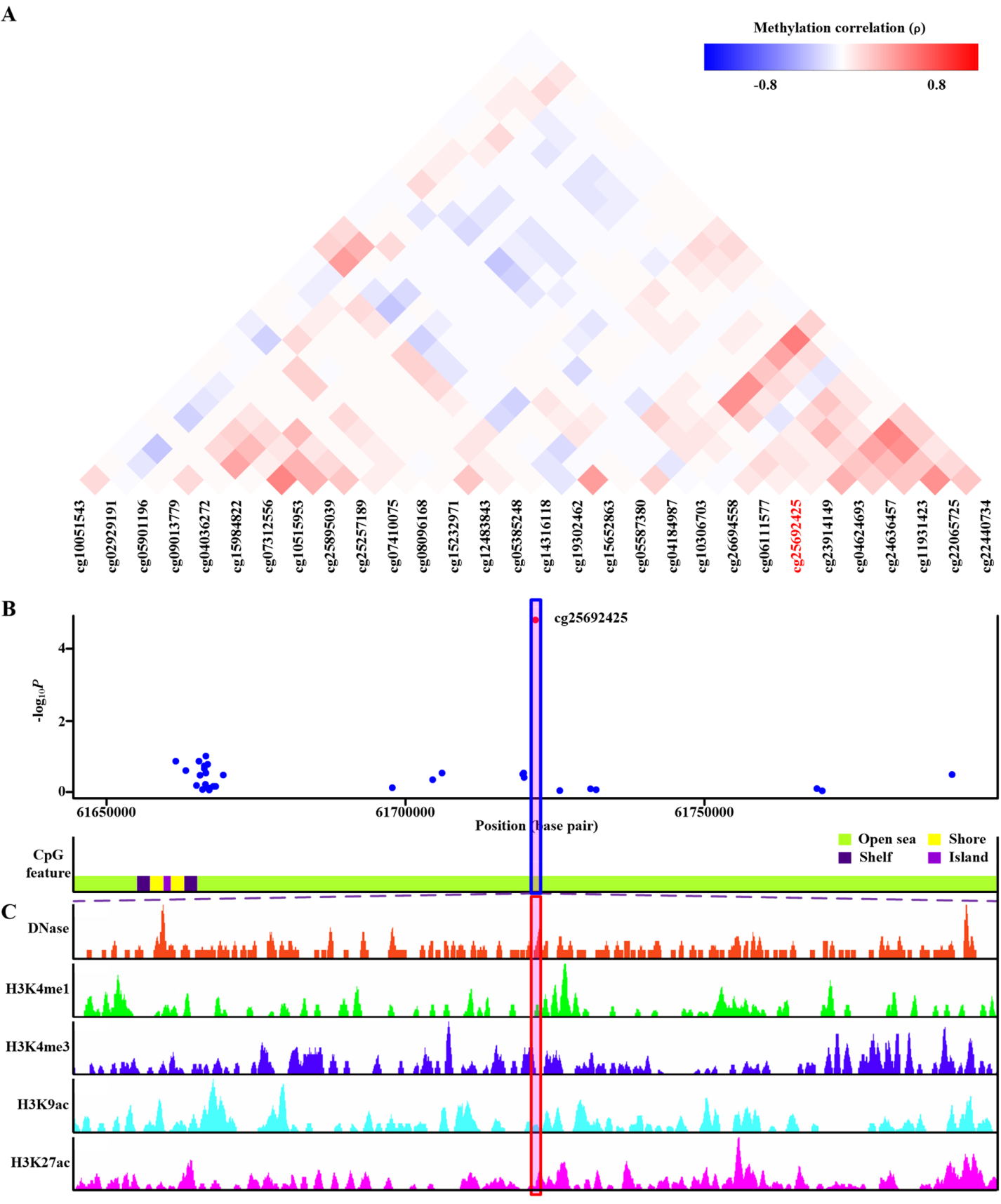
**

**Supplementary Figure 53. Co-methylation analysis and functional annotation of cg08234376.** (A) Patterns of co-methylation at the cytosine-phosphate-guanine dinucleotide (CpG) sites surrounding cg08234376. (B) Monocyte-specific regional association results along with position of nearby CpG island (dark violet), CpG shore (yellow), CpG shelf (indigo) and CpG open sea (green yellow). cg08234376 (highlighted in shaded box) is located in CpG open sea. (C) Functional annotation of cg08234376. DNase hypersensitive sites derived by DNase-seq (DNase Track) and histone marks surrounding cg08234376 (H3K4me1, H3K4me3, H3K9ac and H3K27ac Tracks) in monocytes are shown. DNase hypersensitivity, H3K4me1, H3K4me3, H3K9ac and H3K27ac histone marks are associated with active regulatory elements.

**
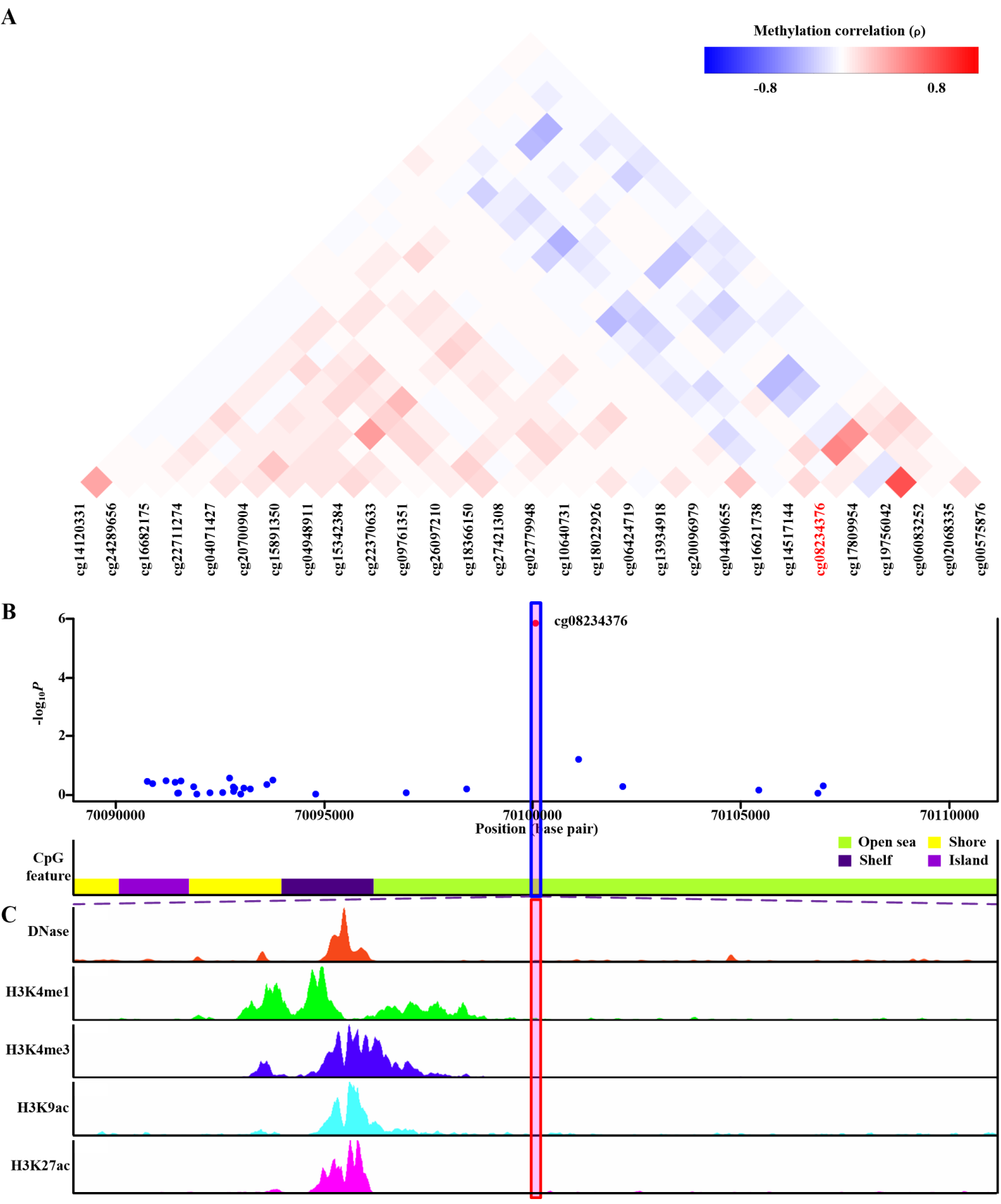
**

**Supplementary Figure 54. Co-methylation analysis and functional annotation of cg19411943.** (A) Patterns of co-methylation at the cytosine-phosphate-guanine dinucleotide (CpG) sites surrounding cg19411943. (B) Monocyte-specific regional association results along with position of nearby CpG island (dark violet), CpG shore (yellow), CpG shelf (indigo) and CpG open sea (green yellow). cg19411943 (highlighted in shaded box) is located in CpG open sea. (C) Functional annotation of cg19411943. DNase hypersensitive sites derived by DNase-seq (DNase Track) and histone marks surrounding cg19411943 (H3K4me1, H3K4me3, H3K9ac and H3K27ac Tracks) in monocytes are shown. DNase hypersensitivity, H3K4me1, H3K4me3, H3K9ac and H3K27ac histone marks are associated with active regulatory elements.

**
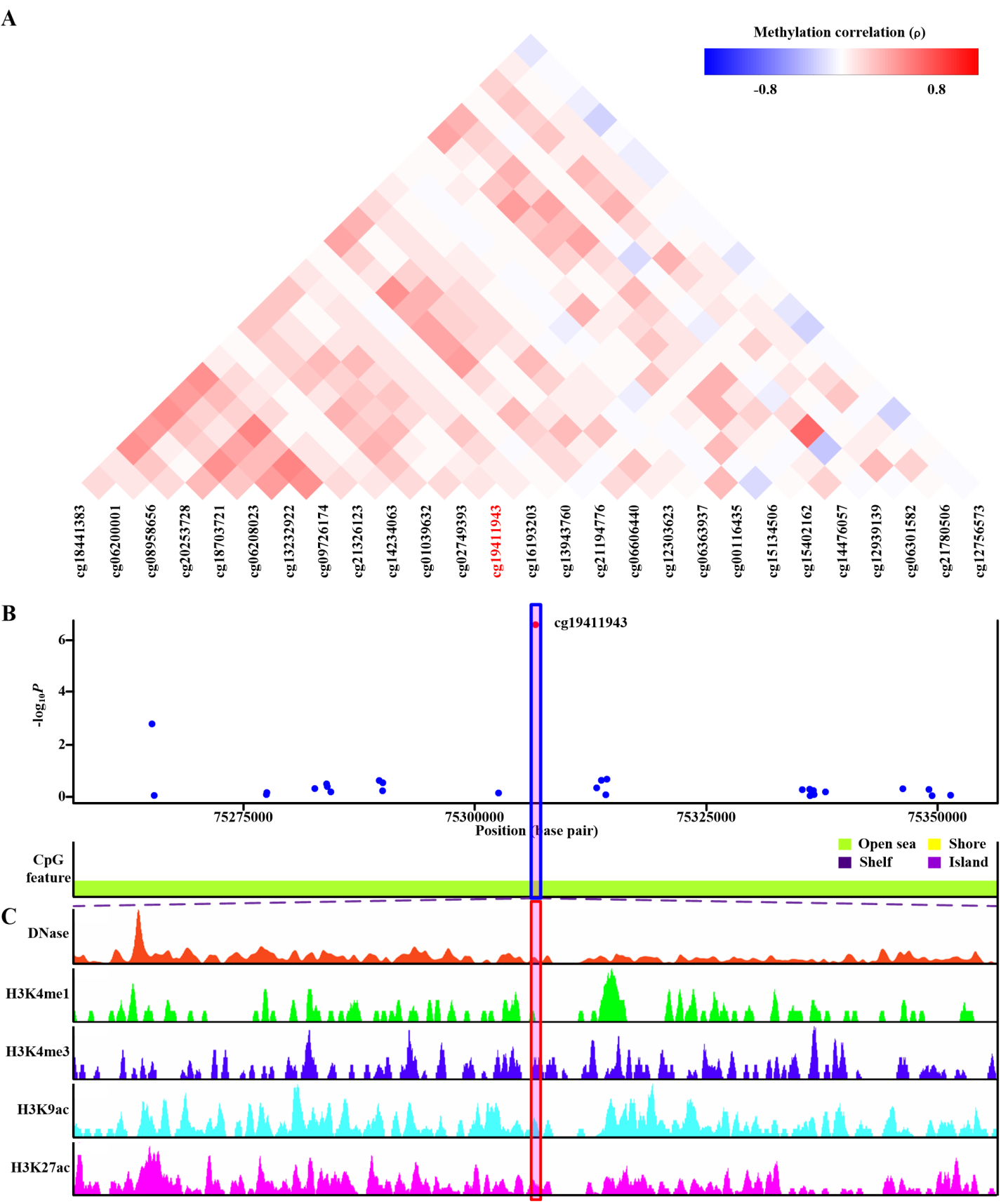
**

**Supplementary Figure 55. Co-methylation analysis and functional annotation of cg19469189.** (A) Patterns of co-methylation at the cytosine-phosphate-guanine dinucleotide (CpG) sites surrounding cg19469189. (B) Monocyte-specific regional association results along with position of nearby CpG island (dark violet), CpG shore (yellow), CpG shelf (indigo) and CpG open sea (green yellow). cg19469189 (highlighted in shaded box) is located in CpG open sea. (C) Functional annotation of cg19469189. DNase hypersensitive sites derived by DNase-seq (DNase Track) and histone marks surrounding cg19469189 (H3K4me1, H3K4me3, H3K9ac and H3K27ac Tracks) in monocytes are shown. DNase hypersensitivity, H3K4me1, H3K4me3, H3K9ac and H3K27ac histone marks are associated with active regulatory elements.

**
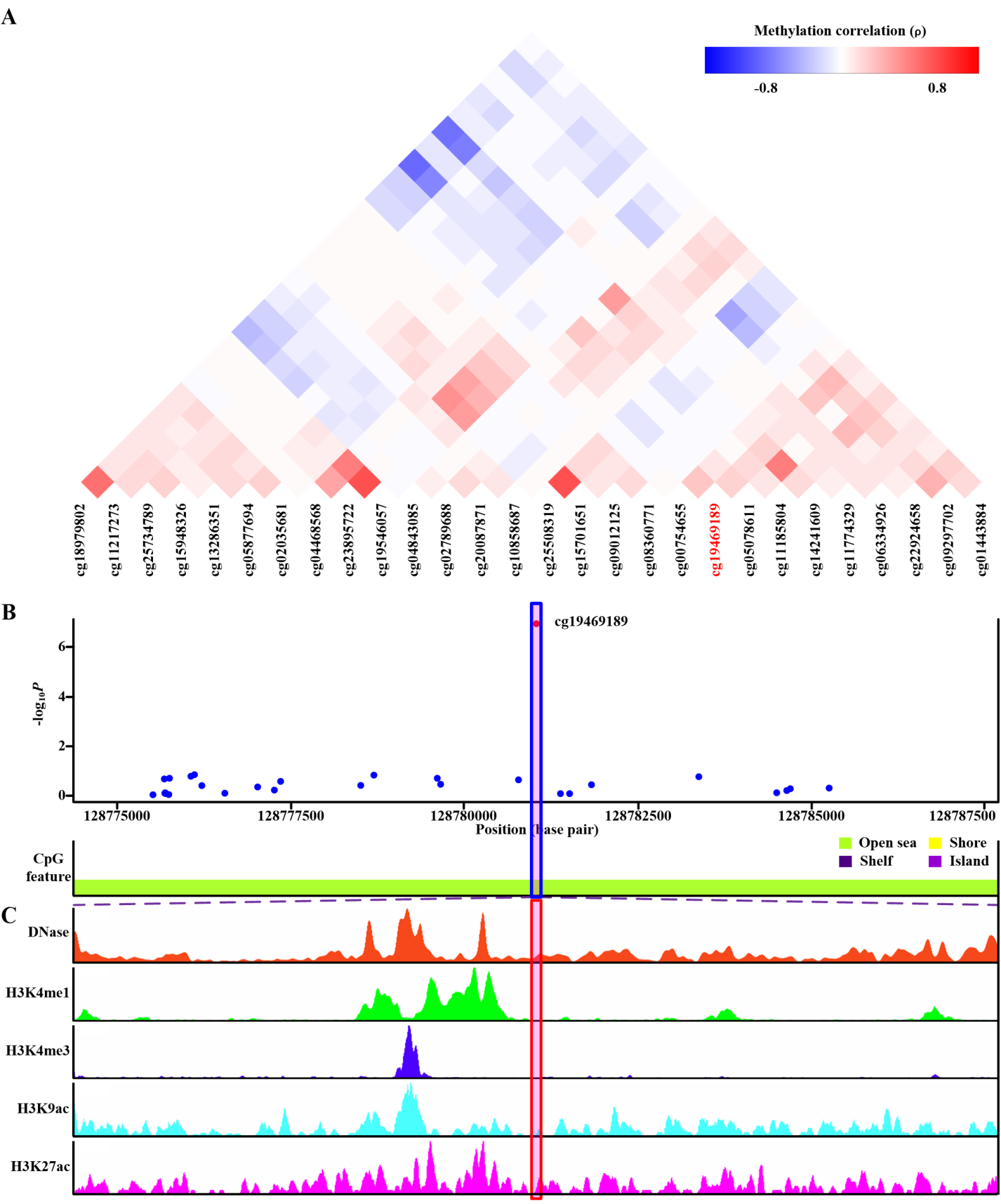
**

**Supplementary Figure 56. Co-methylation analysis and functional annotation of cg18961101.** (A) Patterns of co-methylation at the cytosine-phosphate-guanine dinucleotide (CpG) sites surrounding cg18961101. (B) Monocyte-specific regional association results along with position of nearby CpG island (dark violet), CpG shore (yellow), CpG shelf (indigo) and CpG open sea (green yellow). cg18961101 (highlighted in shaded box) is located in CpG shelf. (C) Functional annotation of cg18961101. DNase hypersensitive sites derived by DNase-seq (DNase Track) and histone marks surrounding cg18961101 (H3K4me1, H3K4me3, H3K9ac and H3K27ac Tracks) in monocytes are shown. DNase hypersensitivity, H3K4me1, H3K4me3, H3K9ac and H3K27ac histone marks are associated with active regulatory elements.

**
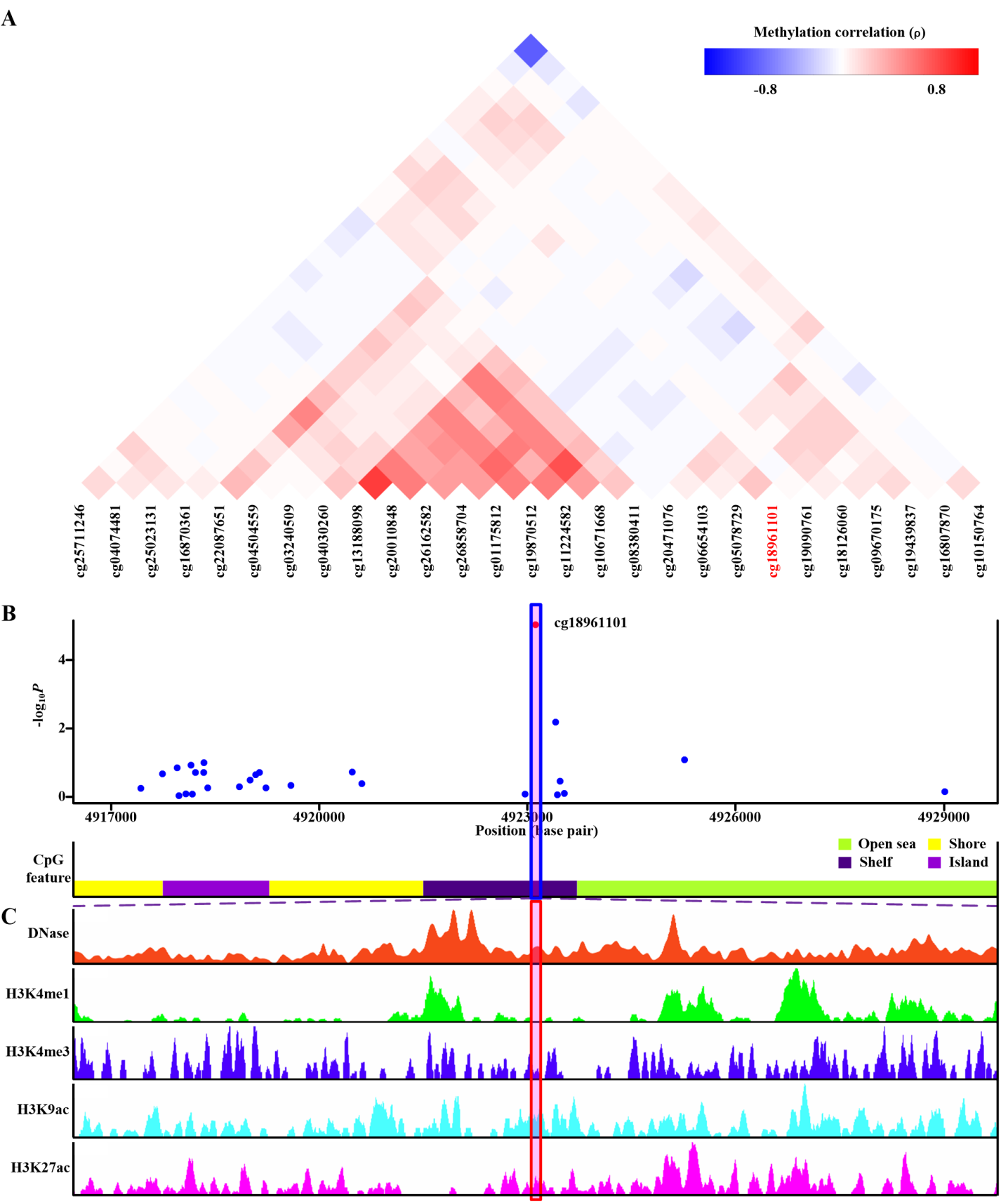
**

**Supplementary Figure 57. Co-methylation analysis and functional annotation of cg19532939.** (A) Patterns of co-methylation at the cytosine-phosphate-guanine dinucleotide (CpG) sites surrounding cg19532939. (B) Monocyte-specific regional association results along with position of nearby CpG island (dark violet), CpG shore (yellow), CpG shelf (indigo) and CpG open sea (green yellow). cg19532939 (highlighted in shaded box) is located in CpG open sea. (C) Functional annotation of cg19532939. DNase hypersensitive sites derived by DNase-seq (DNase Track) and histone marks surrounding cg19532939 (H3K4me1, H3K4me3, H3K9ac and H3K27ac Tracks) in monocytes are shown. DNase hypersensitivity, H3K4me1, H3K4me3, H3K9ac and H3K27ac histone marks are associated with active regulatory elements.

**
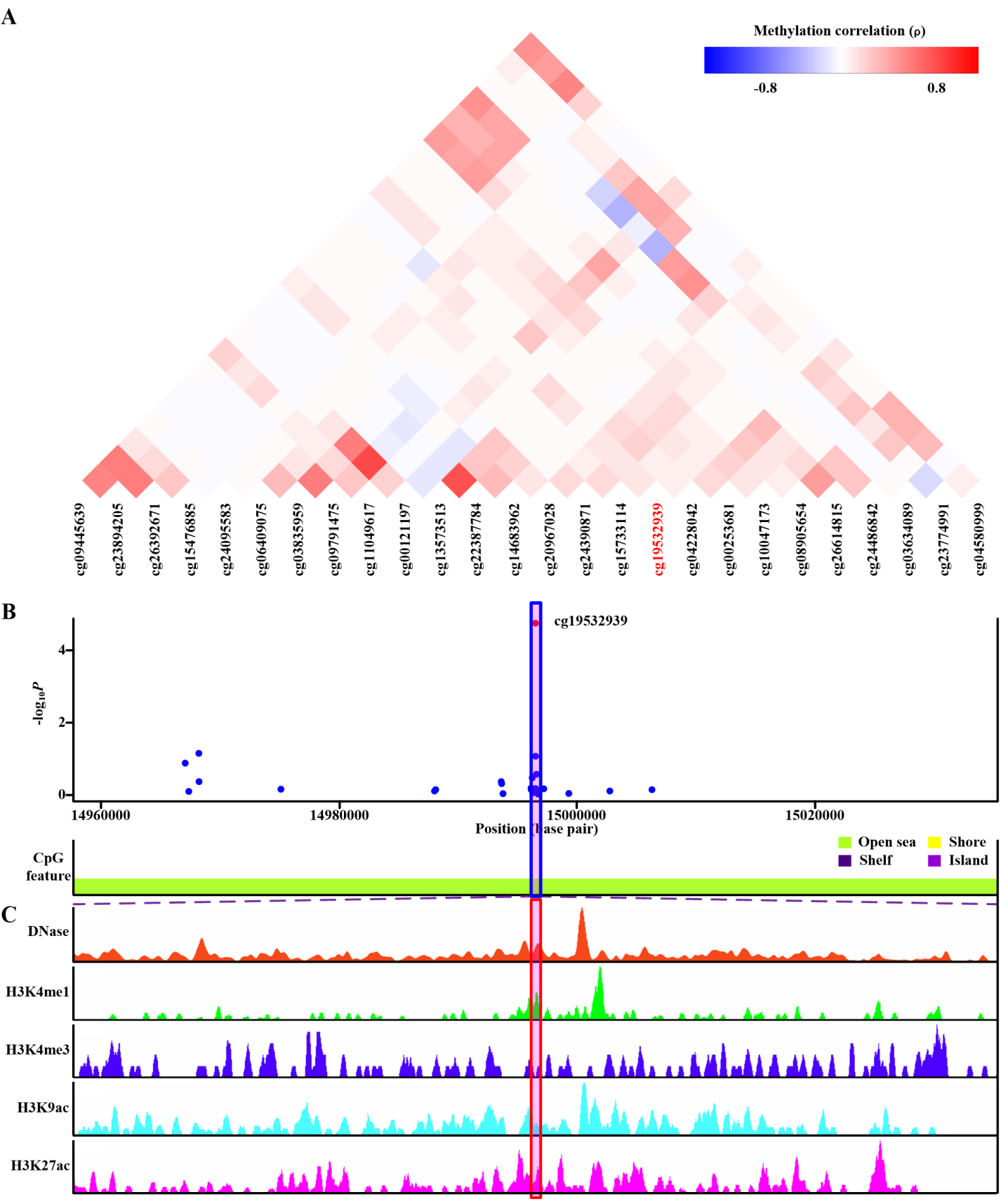
**

**Supplementary Figure 58. Co-methylation analysis and functional annotation of cg02325300.** (A) Patterns of co-methylation at the cytosine-phosphate-guanine dinucleotide (CpG) sites surrounding cg02325300. (B) Monocyte-specific regional association results along with position of nearby CpG island (dark violet), CpG shore (yellow), CpG shelf (indigo) and CpG open sea (green yellow). cg02325300 (highlighted in shaded box) is located in CpG open sea. (C) Functional annotation of cg02325300. DNase hypersensitive sites derived by DNase-seq (DNase Track) and histone marks surrounding cg02325300 (H3K4me1, H3K4me3, H3K9ac and H3K27ac Tracks) in monocytes are shown. DNase hypersensitivity, H3K4me1, H3K4me3, H3K9ac and H3K27ac histone marks are associated with active regulatory elements.

**
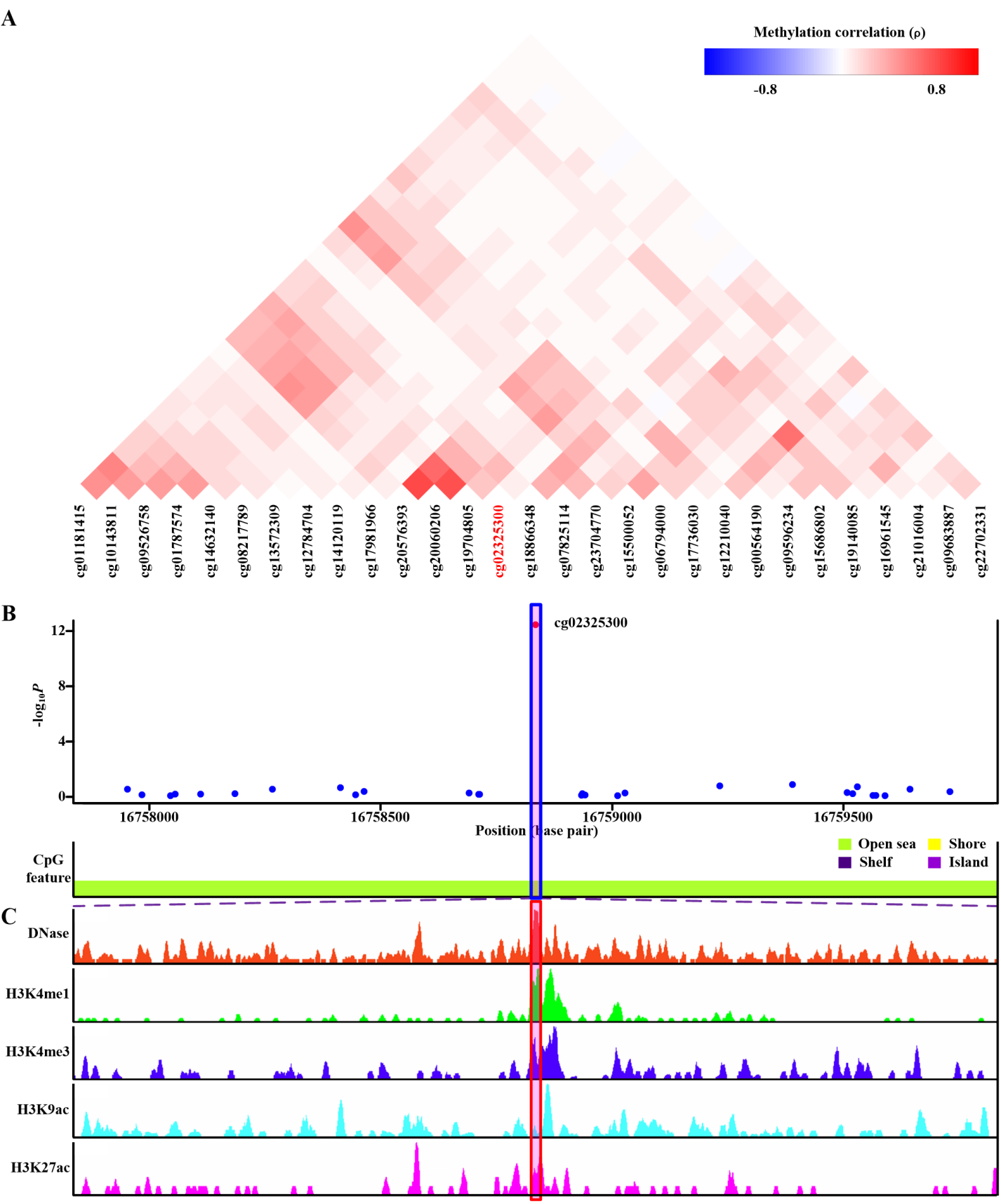
**

**Supplementary Figure 59. Co-methylation analysis and functional annotation of cg25983594.** (A) Patterns of co-methylation at the cytosine-phosphate-guanine dinucleotide (CpG) sites surrounding cg25983594. (B) Monocyte-specific regional association results along with position of nearby CpG island (dark violet), CpG shore (yellow), CpG shelf (indigo) and CpG open sea (green yellow). cg25983594 (highlighted in shaded box) is located in CpG shore. (C) Functional annotation of cg25983594. DNase hypersensitive sites derived by DNase-seq (DNase Track) and histone marks surrounding cg25983594 (H3K4me1, H3K4me3, H3K9ac and H3K27ac Tracks) in monocytes are shown. DNase hypersensitivity, H3K4me1, H3K4me3, H3K9ac and H3K27ac histone marks are associated with active regulatory elements.

**
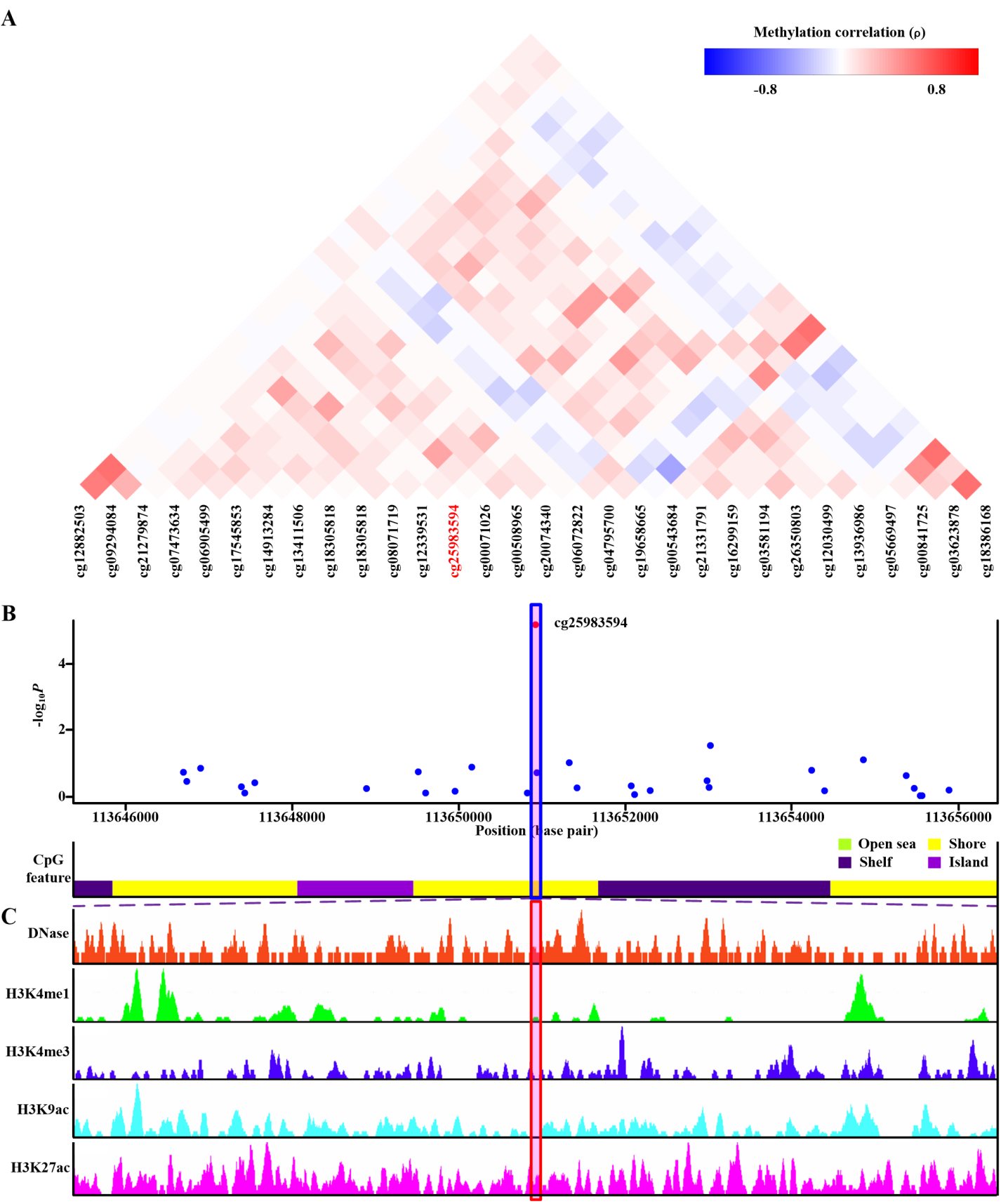
**

**Supplementary Figure 60. Co-methylation analysis and functional annotation of cg07445547.** (A) Patterns of co-methylation at the cytosine-phosphate-guanine dinucleotide (CpG) sites surrounding cg07445547. (B) Monocyte-specific regional association results along with position of nearby CpG island (dark violet), CpG shore (yellow), CpG shelf (indigo) and CpG open sea (green yellow). cg07445547 (highlighted in shaded box) is located in CpG island. (C) Functional annotation of cg07445547. DNase hypersensitive sites derived by DNase-seq (DNase Track) and histone marks surrounding cg07445547 (H3K4me1, H3K4me3, H3K9ac and H3K27ac Tracks) in monocytes are shown. DNase hypersensitivity, H3K4me1, H3K4me3, H3K9ac and H3K27ac histone marks are associated with active regulatory elements.

**
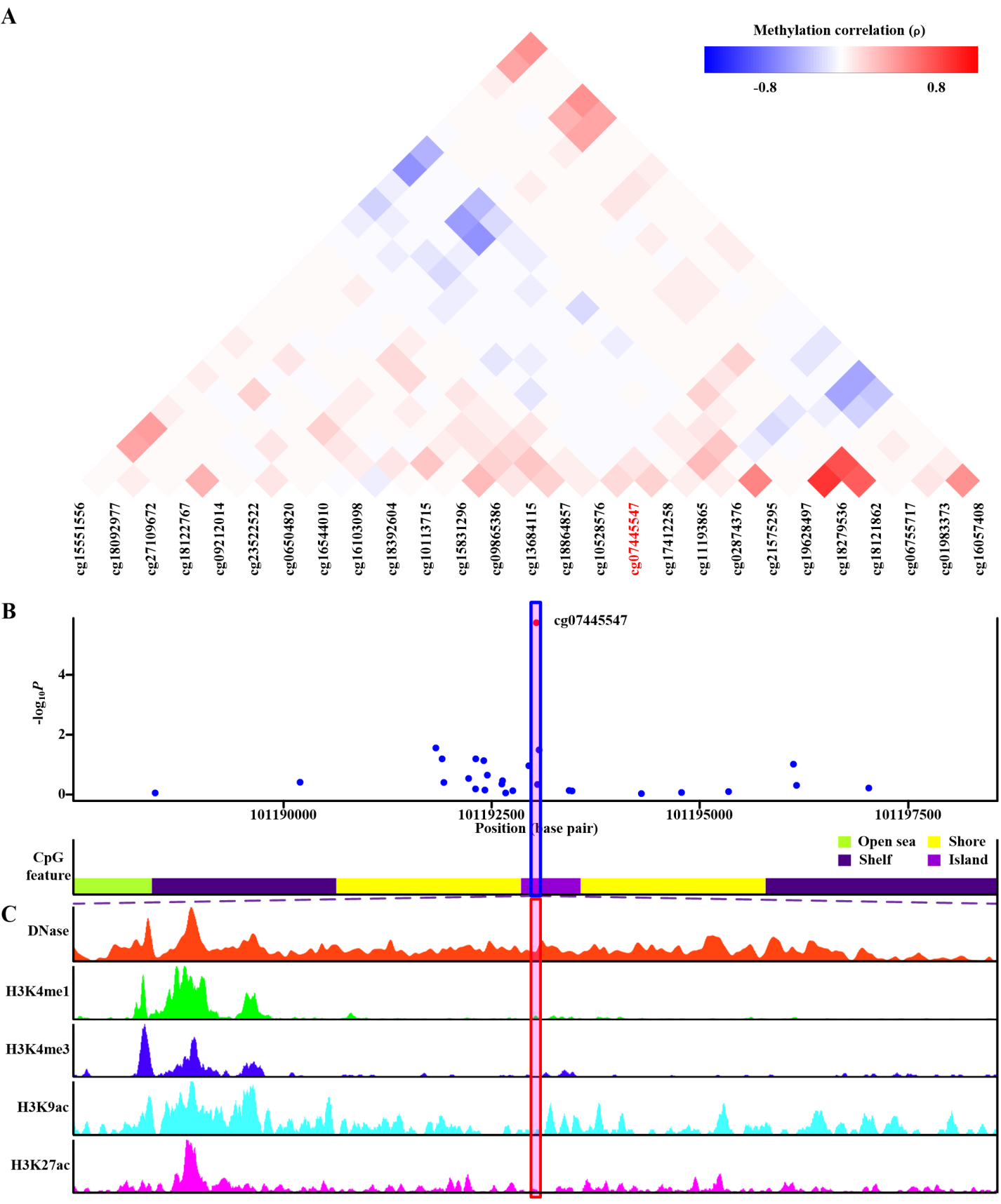
**

**Supplementary Figure 61. Co-methylation analysis and functional annotation of cg22241833.** (A) Patterns of co-methylation at the cytosine-phosphate-guanine dinucleotide (CpG) sites surrounding cg22241833. (B) Monocyte-specific regional association results along with position of nearby CpG island (dark violet), CpG shore (yellow), CpG shelf (indigo) and CpG open sea (green yellow). cg22241833 (highlighted in shaded box) is located in CpG open sea. (C) Functional annotation of cg22241833. DNase hypersensitive sites derived by DNase-seq (DNase Track) and histone marks surrounding cg22241833 (H3K4me1, H3K4me3, H3K9ac and H3K27ac Tracks) in monocytes are shown. DNase hypersensitivity, H3K4me1, H3K4me3, H3K9ac and H3K27ac histone marks are associated with active regulatory elements.

**
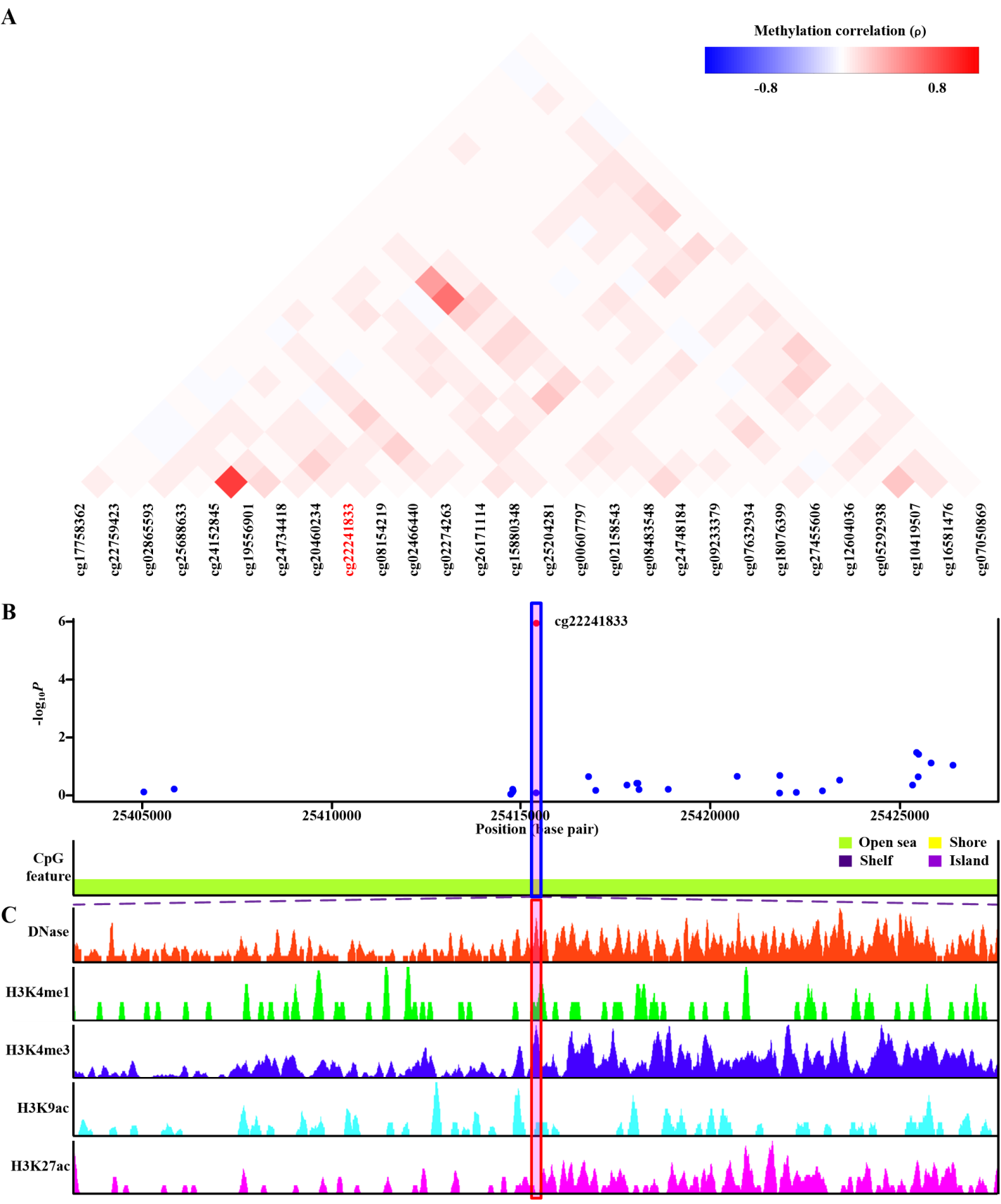
**

**Supplementary Figure 62. Co-methylation analysis and functional annotation of cg11169286.** (A) Patterns of co-methylation at the cytosine-phosphate-guanine dinucleotide (CpG) sites surrounding cg11169286. (B) Monocyte-specific regional association results along with position of nearby CpG island (dark violet), CpG shore (yellow), CpG shelf (indigo) and CpG island (green yellow). cg11169286 (highlighted in shaded box) is located in CpG island. (C) Functional annotation of cg11169286. DNase hypersensitive sites derived by DNase-seq (DNase Track) and histone marks surrounding cg11169286 (H3K4me1, H3K4me3, H3K9ac and H3K27ac Tracks) in monocytes are shown. DNase hypersensitivity, H3K4me1, H3K4me3, H3K9ac and H3K27ac histone marks are associated with active regulatory elements.

**
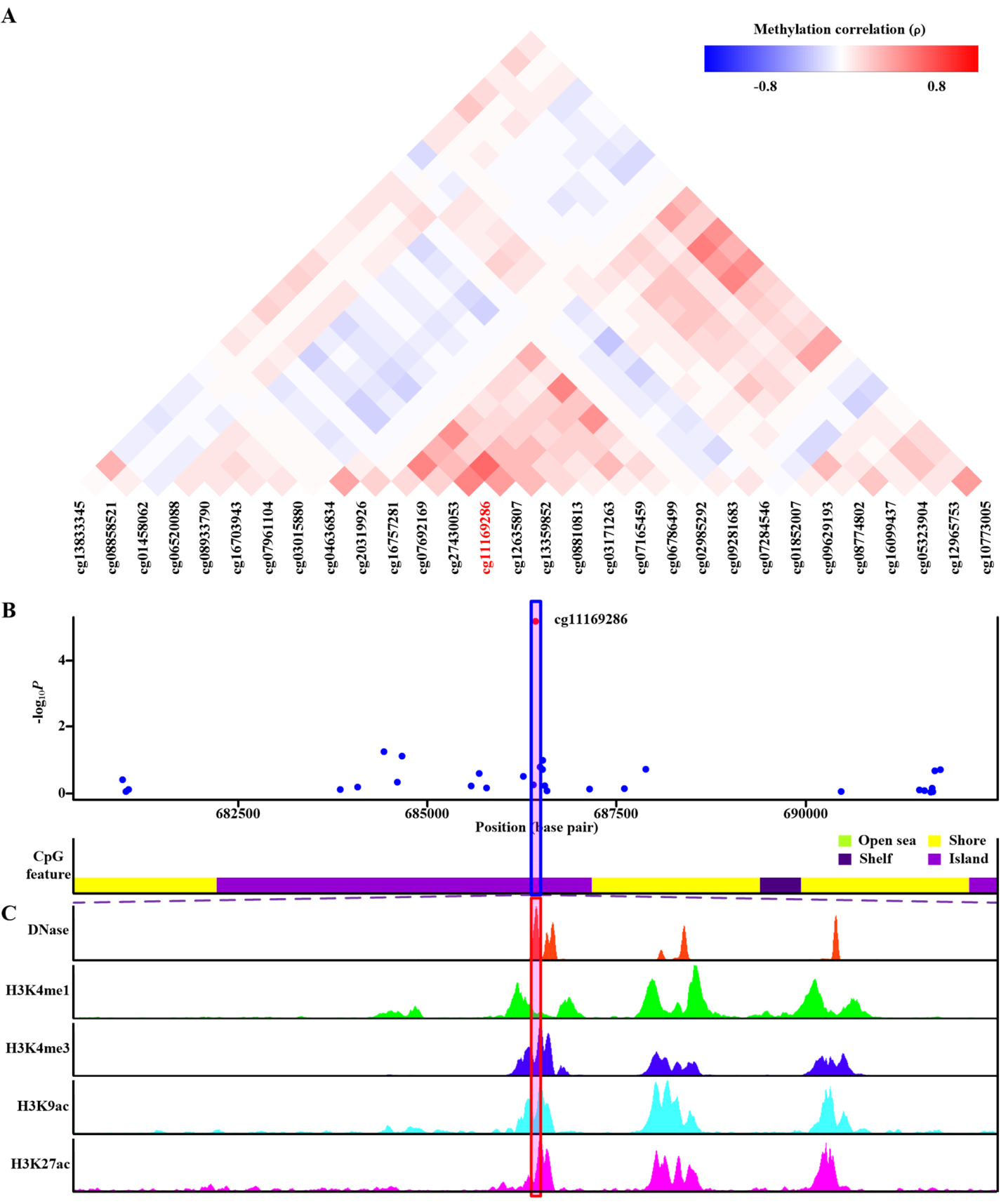
**

**Supplementary Figure 63. Co-methylation analysis and functional annotation of cg05438708.** (A) Patterns of co-methylation at the cytosine-phosphate-guanine dinucleotide (CpG) sites surrounding cg05438708. (B) Monocyte-specific regional association results along with position of nearby CpG island (dark violet), CpG shore (yellow), CpG shelf (indigo) and CpG open sea (green yellow). cg05438708 (highlighted in shaded box) is located in CpG open sea. (C) Functional annotation of cg05438708. DNase hypersensitive sites derived by DNase-seq (DNase Track) and histone marks surrounding cg05438708 (H3K4me1, H3K4me3, H3K9ac and H3K27ac Tracks) in monocytes are shown. DNase hypersensitivity, H3K4me1, H3K4me3, H3K9ac and H3K27ac histone marks are associated with active regulatory elements.

**
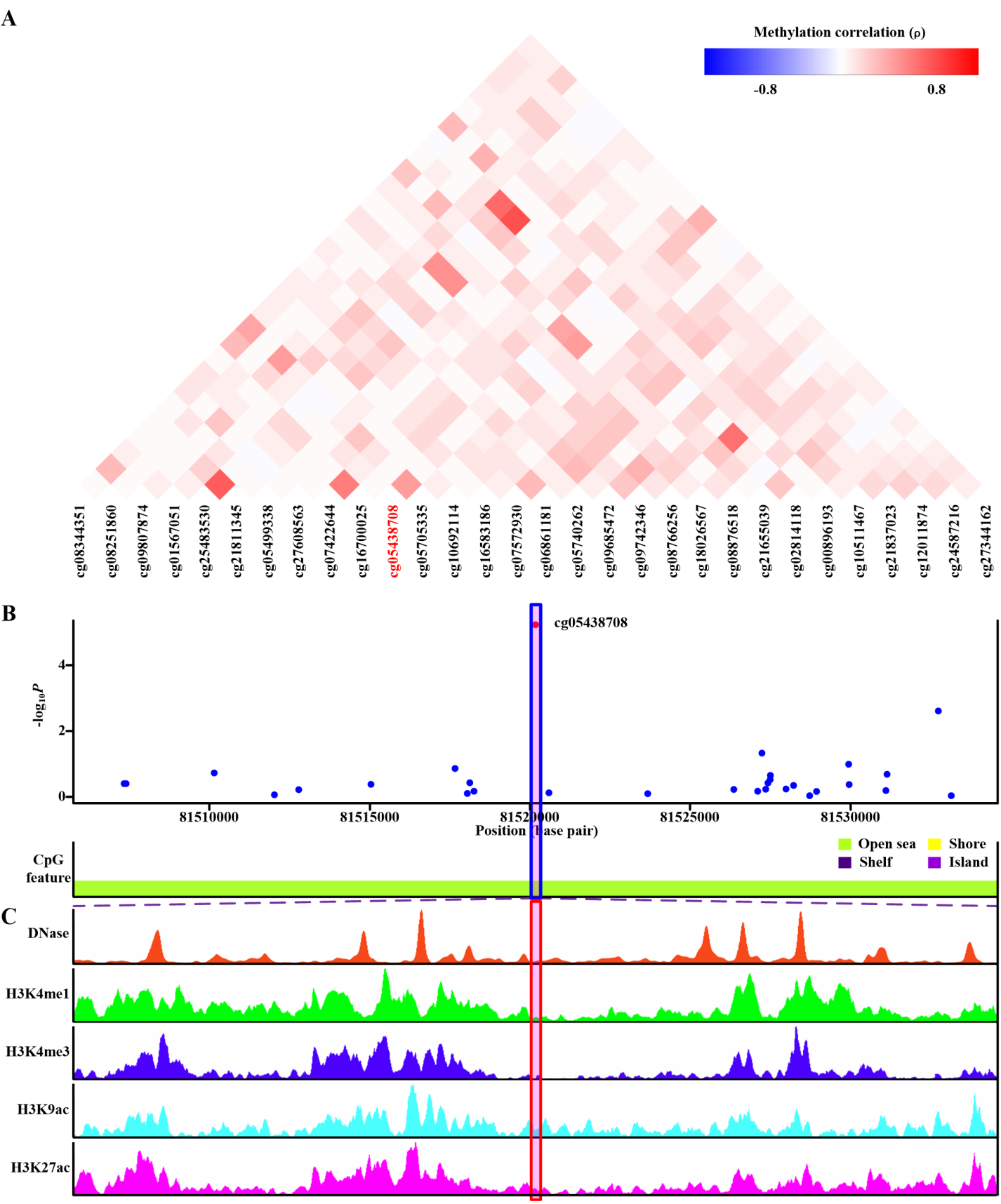
**

**Supplementary Figure 64. Co-methylation analysis and functional annotation of cg11367590.** (A) Patterns of co-methylation at the cytosine-phosphate-guanine dinucleotide (CpG) sites surrounding cg11367590. (B) Monocyte-specific regional association results along with position of nearby CpG island (dark violet), CpG shore (yellow), CpG shelf (indigo) and CpG open sea (green yellow). cg11367590 (highlighted in shaded box) is located in CpG open sea. (C) Functional annotation of cg11367590. DNase hypersensitive sites derived by DNase-seq (DNase Track) and histone marks surrounding cg11367590 (H3K4me1, H3K4me3, H3K9ac and H3K27ac Tracks) in monocytes are shown. DNase hypersensitivity, H3K4me1, H3K4me3, H3K9ac and H3K27ac histone marks are associated with active regulatory elements.

**
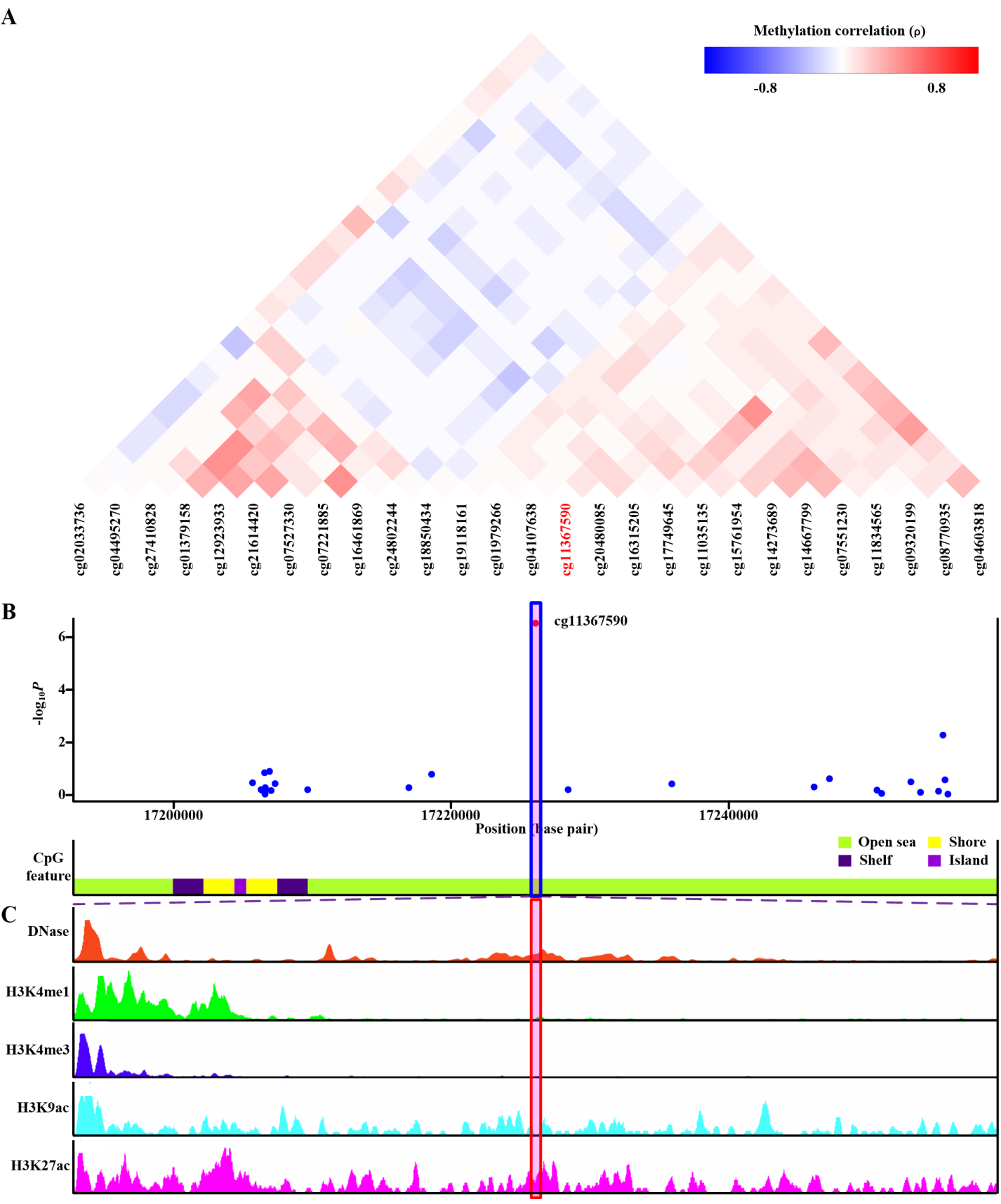
**

**Supplementary Figure 65. Co-methylation analysis and functional annotation of cg05464572.** (A) Patterns of co-methylation at the cytosine-phosphate-guanine dinucleotide (CpG) sites surrounding cg05464572. (B) Monocyte-specific regional association results along with position of nearby CpG island (dark violet), CpG shore (yellow), CpG shelf (indigo) and CpG open sea (green yellow). cg05464572 (highlighted in shaded box) is located in CpG open sea. (C) Functional annotation of cg05464572. DNase hypersensitive sites derived by DNase-seq (DNase Track) and histone marks surrounding cg05464572 (H3K4me1, H3K4me3, H3K9ac and H3K27ac Tracks) in monocytes are shown. DNase hypersensitivity, H3K4me1, H3K4me3, H3K9ac and H3K27ac histone marks are associated with active regulatory elements.

**
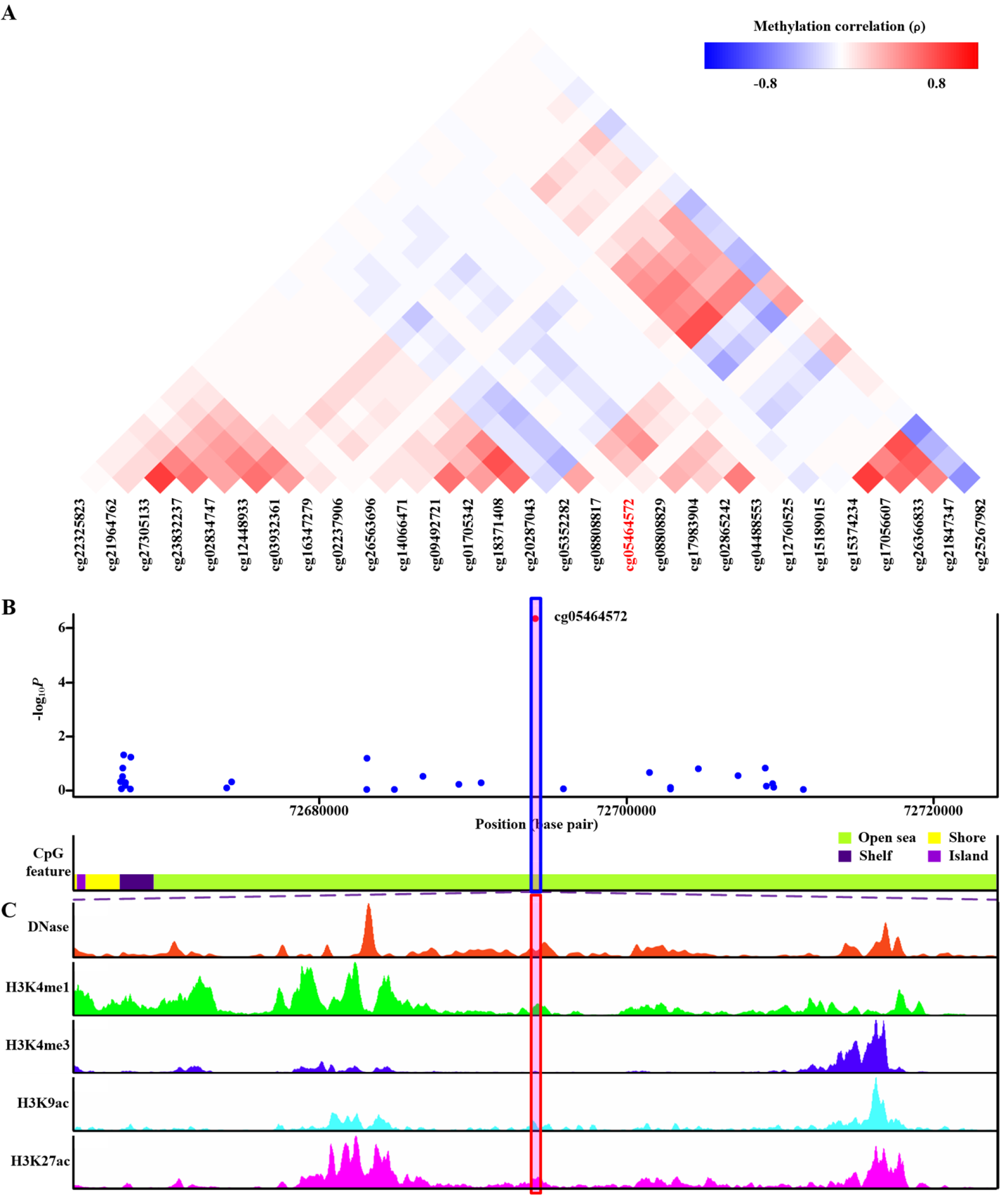
**

**Supplementary Figure 66. Co-methylation analysis and functional annotation of cg14165663.** (A) Patterns of co-methylation at the cytosine-phosphate-guanine dinucleotide (CpG) sites surrounding cg14165663. (B) Monocyte-specific regional association results along with position of nearby CpG island (dark violet), CpG shore (yellow), CpG shelf (indigo) and CpG open sea (green yellow). cg14165663 (highlighted in shaded box) is located in CpG shore. (C) Functional annotation of cg14165663. DNase hypersensitive sites derived by DNase-seq (DNase Track) and histone marks surrounding cg14165663 (H3K4me1, H3K4me3, H3K9ac and H3K27ac Tracks) in monocytes are shown. DNase hypersensitivity, H3K4me1, H3K4me3, H3K9ac and H3K27ac histone marks are associated with active regulatory elements.

**
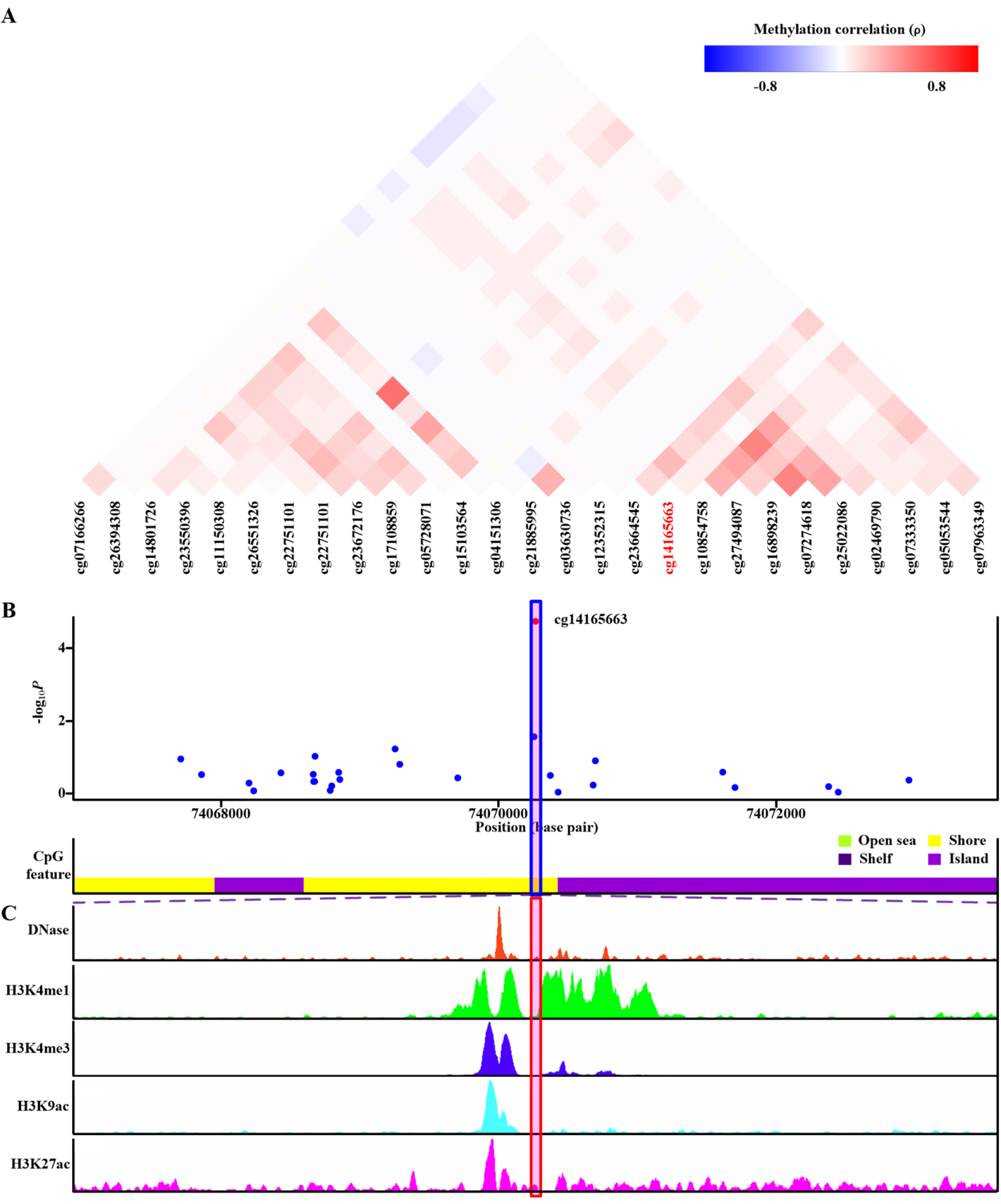
**

**Supplementary Figure 67. Co-methylation analysis and functional annotation of cg26351916.** (A) Patterns of co-methylation at the cytosine-phosphate-guanine dinucleotide (CpG) sites surrounding cg26351916. (B) Monocyte-specific regional association results along with position of nearby CpG island (dark violet), CpG shore (yellow), CpG shelf (indigo) and CpG open sea (green yellow). cg26351916 (highlighted in shaded box) is located in CpG shore. (C) Functional annotation of cg26351916. DNase hypersensitive sites derived by DNase-seq (DNase Track) and histone marks surrounding cg26351916 (H3K4me1, H3K4me3, H3K9ac and H3K27ac Tracks) in monocytes are shown. DNase hypersensitivity, H3K4me1, H3K4me3, H3K9ac and H3K27ac histone marks are associated with active regulatory elements.

**
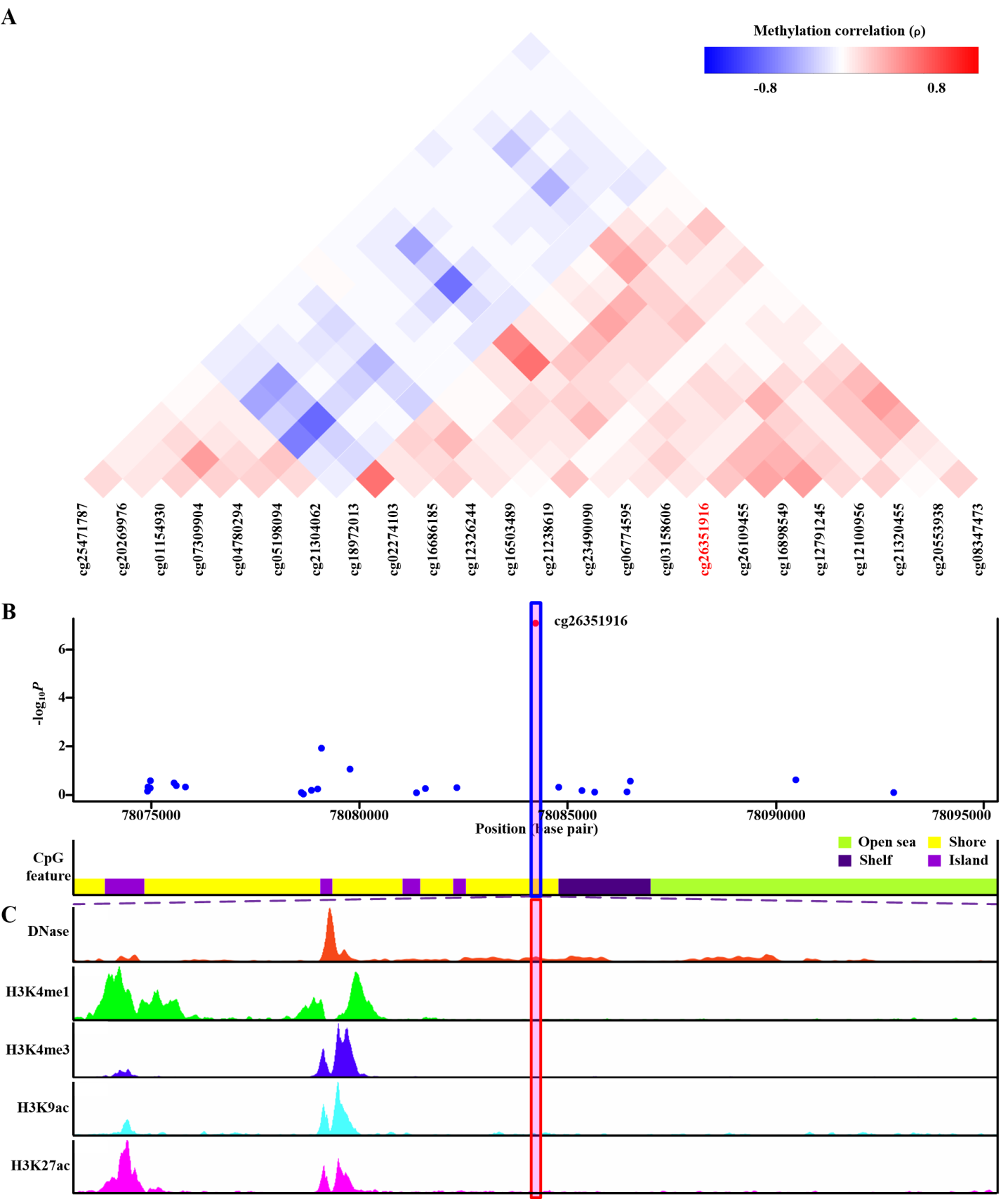
**

**Supplementary Figure 68. Co-methylation analysis and functional annotation of cg04810466.** (A) Patterns of co-methylation at the cytosine-phosphate-guanine dinucleotide (CpG) sites surrounding cg04810466. (B) Monocyte-specific regional association results along with position of nearby CpG island (dark violet), CpG shore (yellow), CpG shelf (indigo) and CpG open sea (green yellow). cg04810466 (highlighted in shaded box) is located in CpG open sea. (C) Functional annotation of cg04810466. DNase hypersensitive sites derived by DNase-seq (DNase Track) and histone marks surrounding cg04810466 (H3K4me1, H3K4me3, H3K9ac and H3K27ac Tracks) in monocytes are shown. DNase hypersensitivity, H3K4me1, H3K4me3, H3K9ac and H3K27ac histone marks are associated with active regulatory elements.

**
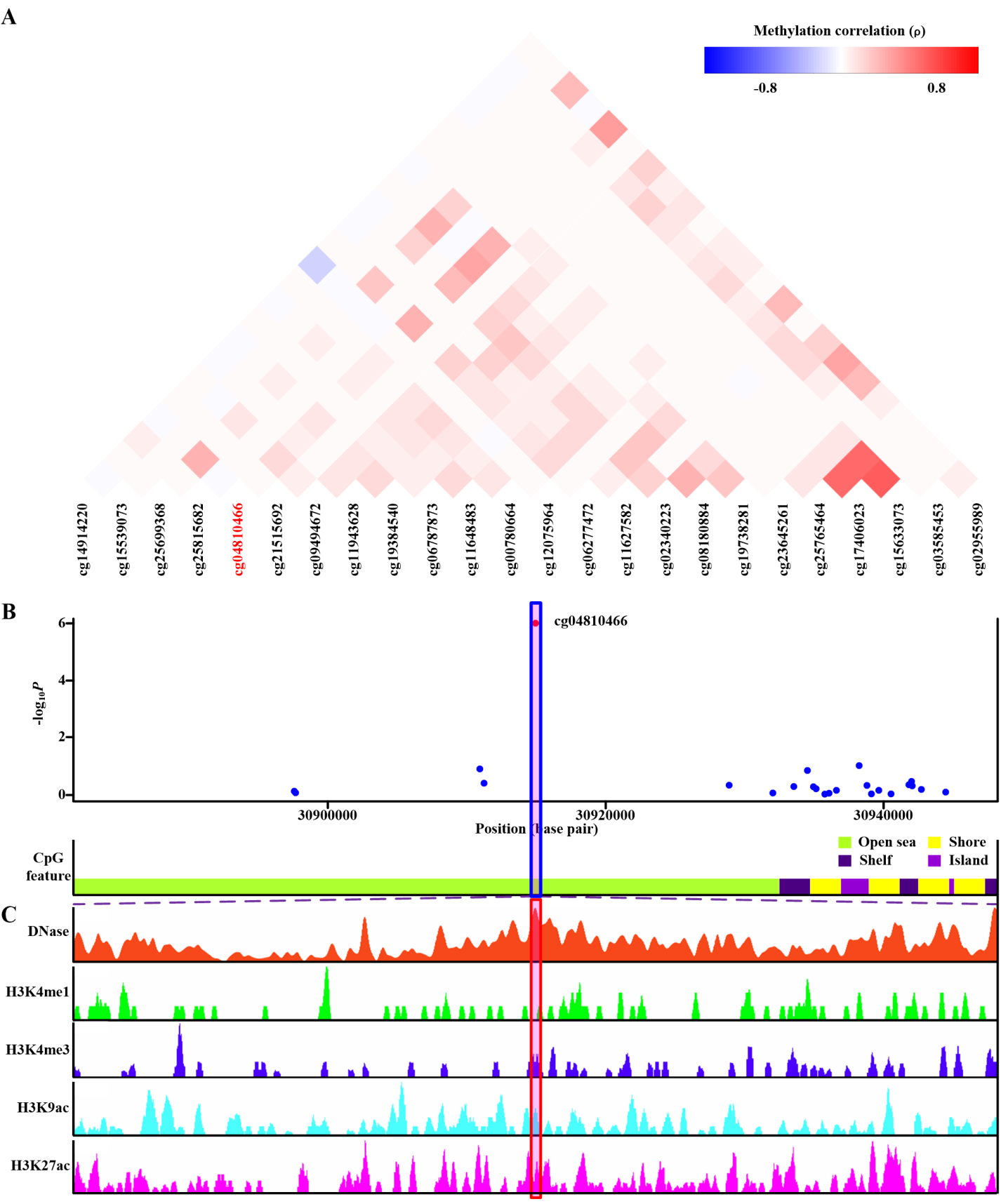
**

**Supplementary Figure 69. Co-methylation analysis and functional annotation of cg05532013.** (A) Patterns of co-methylation at the cytosine-phosphate-guanine dinucleotide (CpG) sites surrounding cg05532013. (B) Monocyte-specific regional association results along with position of nearby CpG island (dark violet), CpG shore (yellow), CpG shelf (indigo) and CpG open sea (green yellow). cg05532013 (highlighted in shaded box) is located in CpG open sea. (C) Functional annotation of cg05532013. DNase hypersensitive sites derived by DNase-seq (DNase Track) and histone marks surrounding cg05532013 (H3K4me1, H3K4me3, H3K9ac and H3K27ac Tracks) in monocytes are shown. DNase hypersensitivity, H3K4me1, H3K4me3, H3K9ac and H3K27ac histone marks are associated with active regulatory elements.

**
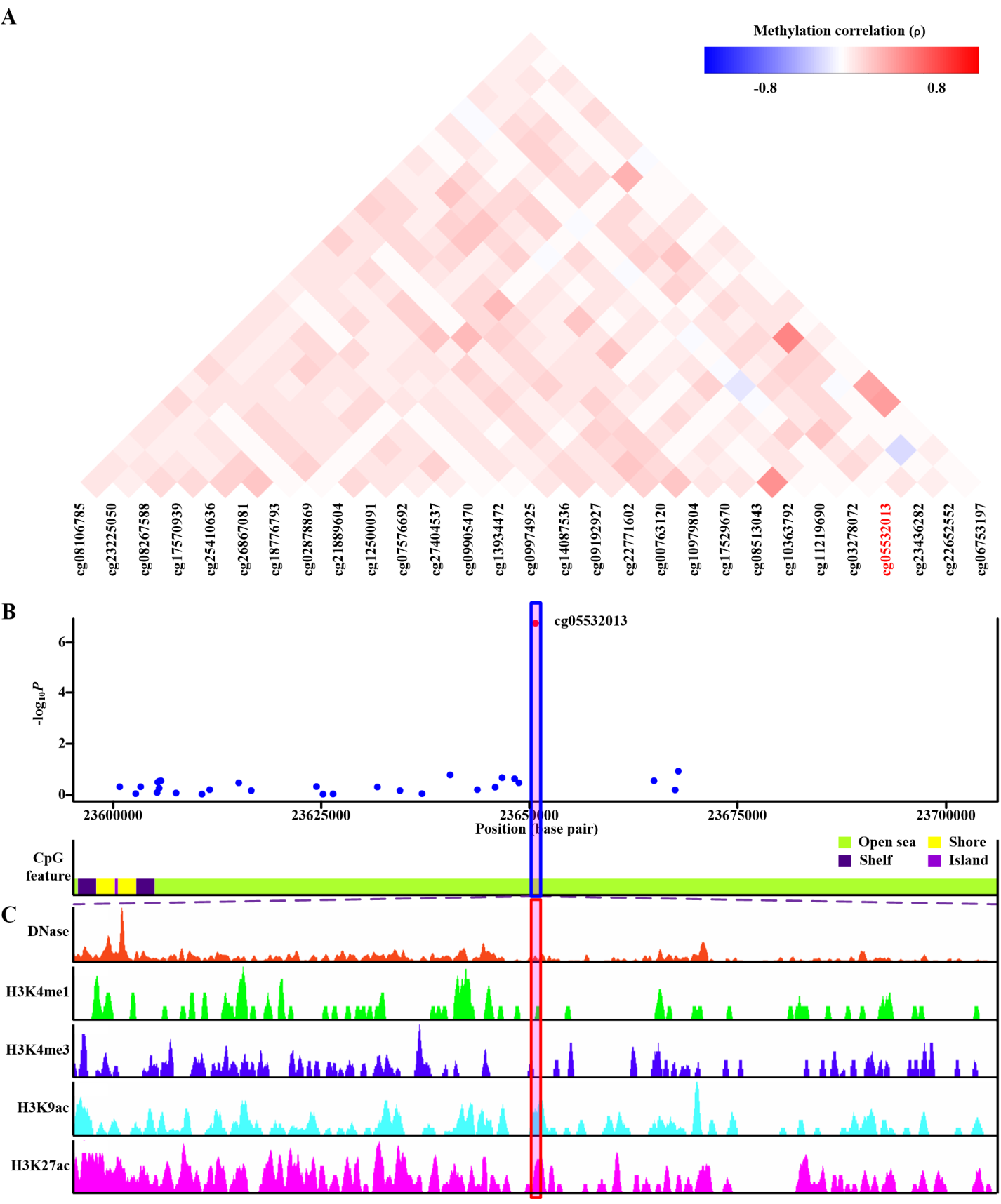
**

**Supplementary Figure 70. Potential transcription factors binding surviving methylation sites.** Potential binding of transcription factors to respective methylation sites according to MoLoTool are highlighted with green while binding of transcription factors to respective methylation sites according to ReMap are highlighted with red and binding of transcription factors to respective methylation sites in both MoLoTool and ReMap are highlighted with yellow. For example, *AHR* is identified to bind cg16630982 and cg17151991 by MoLoTool (green), *ATF2* binds cg03795507, cg16975613 and cg16630982 in ReMap (red) and *ZFX* binds cg03795507 in both MoLoTool and ReMap (yellow). Some transcription factors (*AHR*, *CEBPA*, *CEBPB*, *EGR1*, *HIF1A*, *KLF9*, *MED1*, *NFE2L1*, *PPARG*, *RELA*(*p65*), *SPI1*(*PU.1*), *STAT1*, *VDR*) (orange) shown to increase or decrease interleukin-1β (IL-1β) of monocytes/macrophages in the literature are highlighted (orange) and respective supporting articles [1-14] are listed in the following references (red: transcription factor increases IL-1β; blue: transcription factor decreases IL-1β). References [15-17] about transcription factors (*AHR*, *BRD4*, *PPARG*) reported to facilitate (pink) or inhibit (light green) gouty inflammation in the literature are shown in red.





**Reference**

1. Vogel CF, Sciullo E, Matsumura F. Activation of inflammatory mediators and potential role of ah-receptor ligands in foam cell formation. Cardiovasc Toxicol. 2004; 4:363-73.

2. Zhou J, Li H, Xia X, Herrera A, Pollock N, Reebye V, Sodergren MH, Dorman S, Littman BH, Doogan D, Huang KW, Habib R, Blakey D, et al. Anti-inflammatory Activity of MTL-CEBPA, a Small Activating RNA Drug, in LPS-Stimulated Monocytes and Humanized Mice. Mol Ther. 2019; 27:999-1016.

3. Gorgoni B, Maritano D, Marthyn P, Righi M, Poli V. C/EBP beta gene inactivation causes both impaired and enhanced gene expression and inverse regulation of IL-12 p40 and p35 mRNAs in macrophages. J Immunol. 2002; 168:4055-62.

4. Giri RK, Selvaraj SK, Kalra VK. Amyloid peptide-induced cytokine and chemokine expression in THP-1 monocytes is blocked by small inhibitory RNA duplexes for early growth response-1 messenger RNA. J Immunol. 2003; 170:5281-94.

5. Talwar H, Bouhamdan M, Bauerfeld C, Talreja J, Aoidi R, Houde N, Charron J, Samavati L. MEK2 Negatively Regulates Lipopolysaccharide-Mediated IL-1β Production through HIF-1α Expression. J Immunol. 2019; 202:1815-25.

6. Ai F, Zhao G, Lv W, Liu B, Lin J. Dexamethasone induces aberrant macrophage immune function and apoptosis. Oncol Rep. 2020; 43:427-36.

7. Liang B, Liu E. Abstract 457: Transcription Coactivator MED1 Protects Against Atherosclerosis by Modulation of Macrophage Polarization. Arterioscler Thromb Vasc Biol. 2016; 36:A457.

8. Wang H, Zhu J, Liu Z, Lv H, Lv P, Chen F, Fu J, Hou Y, Zhao R, Xu Y, Zhang Q, Pi J. Silencing of long isoforms of nuclear factor erythroid 2 like 1 primes macrophages towards M1 polarization. Free Radic Biol Med. 2018; 117:37-44.

9. Meier CA, Chicheportiche R, Juge-Aubry CE, Dreyer MG, Dayer JM. Regulation of the interleukin-1 receptor antagonist in THP-1 cells by ligands of the peroxisome proliferator-activated receptor gamma. Cytokine. 2002; 18:320-8.

10. Qian T, Wang K, Cui J, He Y, Yang Z. Angiopoietin-Like Protein 7 Promotes an Inflammatory Phenotype in RAW264.7 Macrophages Through the P38 MAPK Signaling Pathway. Inflammation. 2016; 39:974-85.

11. Wang M, Kong X, Xie Y, He C, Wang T, Zhou H. Role of TLR‑4 in anti‑β2‑glycoprotein I‑induced activation of peritoneal macrophages and vascular endothelial cells in mice. Mol Med Rep. 2019; 19:4353-63.

12. Liu C, Wang M, Sun W, Cai F, Geng S, Su X, Shi Y. PU.1 serves a critical role in the innate defense against Aspergillus fumigatus via dendritic cell-associated C-type lectin receptor-1 and toll-like receptors-2 and 4 in THP-1-derived macrophages. Mol Med Rep. 2017; 15:4084-92.

13. Liu Y, Tang H, Liu X, Chen H, Feng N, Zhang J, Wang C, Qiu M, Yang J, Zhou X. Frontline Science: Reprogramming COX-2, 5-LOX, and CYP4A-mediated arachidonic acid metabolism in macrophages by salidroside alleviates gouty arthritis. J Leukoc Biol. 2019; 105:11-24.

14. Liu X, Yin W, Xie D, He W, Jiang G, Fan J. 5-Aza-2'-deoxycytidine enhances the antimicrobial response of vitamin D receptor against Mycobacterium tuberculosis. RSC Adv. 2016; 6:61740.

15. Muku GE, Lahoti TS, Murray IA, Podolsky MA, Smith KJ, Hubbard TD, Kuzu G, Gowda K, Amin SG, Perdew GH. Ligand-mediated cytoplasmic retention of the Ah receptor inhibits macrophage-mediated acute inflammatory responses. Lab Invest. 2017; 97:1471-87.

16. Jiang F, Hu Q, Zhang Z, Li H, Zhang D, Ma Y, Xu J, Chen H, Cui Y, Zhi Y, Zhang Y, Zhu J, Lu T, et al. Discovery of Benzo[cd]indol-2(1H)-ones and Pyrrolo[4,3,2-de]quinolin-2(1H)-ones as Bromodomain and Extra-Terminal Domain (BET) Inhibitors with Selectivity for the First Bromodomain with Potential High Efficiency against Acute Gouty Arthritis. J Med Chem. 2019; 62:11080-107.

17. Akahoshi T, Namai R, Murakami Y, Watanabe M, Matsui T, Nishimura A, Kitasato H, Kameya T, Kondo H. Rapid induction of peroxisome proliferator-activated receptor gamma expression in human monocytes by monosodium urate monohydrate crystals. Arthritis Rheum. 2003; 48:231-9.

**Supplementary Figure 71. Associations of surviving loci methylation with gout familial clustering.** The plot shows monocyte-specific associations between surviving loci methylation and the number of first-degree relatives with gout. The red points denote loci displaying increased monocyte methylation with increasing number of first-degree relatives while the blue points denote loci displaying decreased monocyte methylation with increasing number of first-degree relatives. The dashed orange line indicates the significance threshold (*P* = 0.05). Monocyte-specific associations of cytosine-phosphate-guanine dinucleotide (CpG) methylation with the number of first-degree relatives with gout are analysed using CellDMC, adjusting for sex, age, alcohol drinking, smoking status, smoking history (total pack-years) and cell fractions.
