# Supplementary material for: Cell lineage-specific methylome and genome alterations in gout
Source: Aging (Albany NY). 2021 Jan 20;13(3):3843–65. doi: 10.18632/aging.202353 (PMC7906142; doi:10.18632/aging.202353)
Supplement: Supplementary Tables [file aging-13-202353-s002.docx]

Supplementary Tables

**Supplementary Table 1. Cells utilised to retrieve monocyte-specific transcription factor binding sites.**

| CD14 [1] | Macrophage | monocyte-derived macrophage (MDM) | MOLM-13 [1-2] |
| --- | --- | --- | --- |
| MOLM-14 [2-3] | Monocyte | NOMO1 [4] | MV4-11 [1, 5] |
| THP-1 [1, 4] | U-937 [6] |  |  |

**Reference**

1. John S, Chen H, Deng M, Gui X, Wu G, Chen W, Li Z, Zhang N, An Z, Zhang CC. A Novel Anti-LILRB4 CAR-T Cell for the Treatment of Monocytic AML. Mol Ther. 2018; 26:2487-95.

2. Bredholt T, Dimba EA, Hagland HR, Wergeland L, Skavland J, Fossan KO, Tronstad KJ, Johannessen AC, Vintermyr OK, Gjertsen BT. Camptothecin and khat (Catha edulis Forsk.) induced distinct cell death phenotypes involving modulation of c-FLIPL, Mcl-1, procaspase-8 and mitochondrial function in acute myeloid leukemia cell lines. Mol Cancer. 2009; 8:101.

3. Balachandran C, Emi N, Arun Y, Yamamoto N, Duraipandiyan V, Inaguma Y, Okamoto A, Ignacimuthu S, Al-Dhabi NA, Perumal PT. In vitro antiproliferative activity of 2,3-dihydroxy-9,10-anthraquinone induced apoptosis against COLO320 cells through cytochrome c release caspase mediated pathway with PI3K/AKT and COX-2 inhibition. Chem Biol Interact. 2016; 249:23-35.

4. Wang Q, Imamura R, Motani K, Kushiyama H, Nagata S, Suda T. Pyroptotic cells externalize eat-me and release find-me signals and are efficiently engulfed by macrophages. Int Immunol. 2013; 25:363-72.

5. Poh AR, Love CG, Masson F, Preaudet A, Tsui C, Whitehead L, Monard S, Khakham Y, Burstroem L, Lessene G, Sieber O, Lowell C, Putoczki TL, et al. Inhibition of Hematopoietic Cell Kinase Activity Suppresses Myeloid Cell-Mediated Colon Cancer Progression. Cancer Cell. 2017; 31:563-75.e5.

6. Mironov ME, Oleshko OS, Pokrovskii MA, Rybalova TV, Pechurov VK, Pokrovskii AG, Cheresis SV, Mishinov SV, Stupak VV, Shults EE. 6-(4'-Aryl-1',2',3'-triazolyl)-spirostan-3,5-diols and 6-(4'-Aryl-1',2',3'-triazolyl)-7-hydroxyspirosta-1,4-dien-3-ones: Synthesis and analysis of their cytotoxicity. Steroids. 2019; 151:108460.

**Supplementary Table 2. Differentially methylated sites in various cell lineages.**

| CpG site | Δβ^a^ | *P* | FDR | Chr | Position^b^ | Gene^c^ |
| --- | --- | --- | --- | --- | --- | --- |
| **Monocytes** |  |  |  |  |  |  |
| cg15158067 | -1.00 | 1.24×10^-6^ | 4.89×10^-3^ | 1 | 1536396 | *C1orf233* |
| cg22626579 | -0.69 | 1.81×10^-7^ | 9.63×10^-4^ | 1 | 2038628 | *PRKCZ* |
| cg09599979 | -1.00 | 4.03×10^-28^ | 1.48×10^-22^ | 1 | 6660000 | *KLHL21* |
| cg02254548 | -0.97 | 7.43×10^-6^ | 2.17×10^-2^ | 1 | 15585958 | *FHAD1* |
| cg15642494 | -0.46 | 1.62×10^-6^ | 6.04×10^-3^ | 1 | 19616829 |  |
| cg07973655 | 0.24 | 1.03×10^-5^ | 2.91×10^-2^ | 1 | 25566628 |  |
| cg04285956 | 0.14 | 1.23×10^-5^ | 3.31×10^-2^ | 1 | 25566753 |  |
| cg01718853 | 0.51 | 1.54×10^-5^ | 4.02×10^-2^ | 1 | 27157161 | *ZDHHC18* |
| cg03789579 | -1.00 | 1.35×10^-9^ | 1.40×10^-5^ | 1 | 32670241 | *IQCC* |
| cg20942219 | -1.00 | 6.75×10^-9^ | 5.85×10^-5^ | 1 | 44141161 | *KDM4A* |
| cg02696670 | 0.64 | 2.39×10^-7^ | 1.16×10^-3^ | 1 | 64669384 | *UBE2U* |
| cg18117228 | 1.00 | 2.68×10^-10^ | 3.35×10^-6^ | 1 | 65775772 | *DNAJC6* |
| cg18886702 | -0.68 | 8.48×10^-11^ | 1.20×10^-6^ | 1 | 67658441 | *IL23R* |
| cg13559233 | -1.00 | 8.38×10^-6^ | 2.42×10^-2^ | 1 | 82269258 | *LPHN2* |
| cg08721840 | -0.59 | 2.03×10^-6^ | 7.22×10^-3^ | 1 | 155293541 | *RUSC1* |
| cg05638359 | 1.00 | 8.51×10^-8^ | 5.27×10^-4^ | 1 | 190444387 | *FAM5C* |
| cg22889645 | 0.55 | 1.11×10^-7^ | 6.52×10^-4^ | 1 | 205326399 | *KLHDC8A* |
| cg03945122 | 1.00 | 7.40×10^-10^ | 8.02×10^-6^ | 1 | 207840521 | *CR1L* |
| cg03231504 | 0.91 | 7.24×10^-6^ | 2.13×10^-2^ | 1 | 228677993 | *RNF187* |
| cg11157725 | 1.00 | 1.87×10^-7^ | 9.63×10^-4^ | 1 | 230827973 | *COG2* |
| cg26461510 | -0.79 | 2.09×10^-7^ | 1.05×10^-3^ | 1 | 248569107 | *OR2T1* |
| cg07173100 | -0.70 | 4.04×10^-8^ | 2.83×10^-4^ | 2 | 3606140 | *RNASEH1* |
| cg05097535 | -0.71 | 4.26×10^-12^ | 8.31×10^-8^ | 2 | 27353339 | *ABHD1* |
| cg03275949 | 1.00 | 3.17×10^-11^ | 4.94×10^-7^ | 2 | 28113605 | *BRE-AS1* |
| cg05547766 | -0.41 | 3.92×10^-6^ | 1.28×10^-2^ | 2 | 47232971 | *TTC7A* |
| cg07083023 | -0.97 | 1.23×10^-5^ | 3.31×10^-2^ | 2 | 51254981 | *NRXN1* |
| cg19824059 | -1.00 | 4.53×10^-8^ | 3.12×10^-4^ | 2 | 51255324 | *NRXN1* |
| cg08086731 | 0.77 | 1.06×10^-5^ | 2.97×10^-2^ | 2 | 62429249 | *B3GNT2* |
| cg09586879 | -0.68 | 5.50×10^-8^ | 3.68×10^-4^ | 2 | 70900488 | *ADD2* |
| cg08682760 | 0.58 | 2.66×10^-8^ | 1.96×10^-4^ | 2 | 104694659 |  |
| cg22865501 | -0.69 | 1.05×10^-5^ | 2.96×10^-2^ | 2 | 105321544 |  |
| cg10155147 | -1.00 | 1.69×10^-11^ | 2.96×10^-7^ | 2 | 106500829 | *NCK2* |
| cg13040600 | -0.62 | 8.47×10^-7^ | 3.59×10^-3^ | 2 | 135037975 | *MGAT5* |
| cg18110444 | 0.90 | 1.59×10^-14^ | 5.57×10^-10^ | 2 | 136743460 | *DARS* |
| cg08797047 | -0.88 | 1.12×10^-5^ | 3.09×10^-2^ | 2 | 144359042 | *ARHGAP15* |
| cg05903707 | -1.00 | 6.04×10^-6^ | 1.85×10^-2^ | 2 | 158843896 |  |
| cg02439066 | -0.79 | 3.66×10^-7^ | 1.67×10^-3^ | 2 | 169214197 |  |
| cg23279346 | -0.74 | 7.56×10^-8^ | 4.84×10^-4^ | 2 | 170777718 | *UBR3* |
| cg02993437 | 1.00 | 7.88×10^-6^ | 2.29×10^-2^ | 2 | 172937981 | *METAP1D* |
| cg08119434 | 1.00 | 1.64×10^-5^ | 4.21×10^-2^ | 2 | 205036349 |  |
| cg01236747 | -0.56 | 1.17×10^-6^ | 4.68×10^-3^ | 2 | 208815027 | *PLEKHM3* |
| cg21084456 | -0.47 | 1.70×10^-5^ | 4.36×10^-2^ | 2 | 217674497 |  |
| cg01080600 | -0.80 | 1.01×10^-6^ | 4.14×10^-3^ | 2 | 236443773 | *AGAP1* |
| cg14357719 | 1.00 | 1.06×10^-11^ | 1.95×10^-7^ | 2 | 236500777 | *AGAP1* |
| cg22049753 | 0.66 | 9.45×10^-7^ | 3.96×10^-3^ | 2 | 240895815 |  |
| cg10314750 | -0.74 | 2.98×10^-6^ | 1.03×10^-2^ | 3 | 9922257 | *CIDEC* |
| cg24016448 | 0.68 | 1.33×10^-8^ | 1.07×10^-4^ | 3 | 11607988 | *VGLL4* |
| cg07759247 | 0.68 | 1.78×10^-7^ | 9.58×10^-4^ | 3 | 38040636 | *VILL* |
| cg07285807 | 1.00 | 1.20×10^-24^ | 1.77×10^-19^ | 3 | 49235826 | *CCDC36* |
| cg00091098 | 0.52 | 1.06×10^-7^ | 6.32×10^-4^ | 3 | 71100993 | *FOXP1* |
| cg24532093 | 0.84 | 1.90×10^-7^ | 9.73×10^-4^ | 3 | 98710785 |  |
| cg15766024 | 0.63 | 1.88×10^-8^ | 1.43×10^-4^ | 3 | 99904510 | *TMEM30C* |
| cg14775821 | -0.69 | 2.17×10^-7^ | 1.08×10^-3^ | 3 | 111622625 | *PHLDB2* |
| cg02797353 | 0.86 | 3.86×10^-9^ | 3.65×10^-5^ | 3 | 119531385 | *NR1I2* |
| cg10513770 | 0.65 | 1.78×10^-5^ | 4.54×10^-2^ | 3 | 122798912 | *PDIA5* |
| cg14603321 | -1.00 | 7.05×10^-10^ | 7.75×10^-6^ | 3 | 171920662 | *FNDC3B* |
| cg04738673 | 1.00 | 7.04×10^-14^ | 2.08×10^-9^ | 3 | 177482968 |  |
| cg04258358 | 0.69 | 3.95×10^-6^ | 1.29×10^-2^ | 4 | 728867 | *PCGF3* |
| cg12250142 | -0.65 | 2.83×10^-10^ | 3.47×10^-6^ | 4 | 15657883 | *FBXL5* |
| cg10053430 | -1.00 | 2.54×10^-17^ | 1.44×10^-12^ | 4 | 83500062 |  |
| cg07856552 | -1.00 | 3.37×10^-10^ | 4.00×10^-6^ | 4 | 90548906 |  |
| cg24467535 | -1.00 | 2.45×10^-10^ | 3.12×10^-6^ | 4 | 100244604 |  |
| cg17318769 | -1.00 | 1.79×10^-11^ | 3.07×10^-7^ | 4 | 154229091 | *TRIM2* |
| cg11383226 | -1.00 | 2.84×10^-8^ | 2.05×10^-4^ | 4 | 159236616 |  |
| cg22517735 | 0.30 | 1.65×10^-7^ | 8.92×10^-4^ | 4 | 174451954 | *NBLA00301* |
| cg03930088 | 0.86 | 2.65×10^-12^ | 5.77×10^-8^ | 4 | 187647395 |  |
| cg04859695 | -1.00 | 7.92×10^-15^ | 3.07×10^-10^ | 4 | 187666362 |  |
| cg16227022 | 0.51 | 1.71×10^-6^ | 6.35×10^-3^ | 4 | 189898680 |  |
| cg19940052 | -0.42 | 6.97×10^-7^ | 3.00×10^-3^ | 5 | 5474505 | *KIAA0947* |
| cg15288149 | -0.98 | 5.31×10^-11^ | 7.83×10^-7^ | 5 | 6409323 |  |
| cg10556369 | -0.42 | 4.96×10^-6^ | 1.56×10^-2^ | 5 | 36619118 | *SLC1A3* |
| cg19459207 | 1.00 | 1.30×10^-5^ | 3.46×10^-2^ | 5 | 49737773 | *EMB* |
| cg15120413 | 1.00 | 2.40×10-9 | 2.29×10^-5^ | 5 | 53753195 |  |
| cg04480129 | -1.00 | 1.33×10-6 | 5.13×10^-3^ | 5 | 66316631 | *MAST4* |
| cg10435952 | 1.00 | 3.26×10-8 | 2.33×10^-4^ | 5 | 81884312 |  |
| cg03344380 | -1.00 | 6.22×10^-6^ | 1.89×10^-2^ | 5 | 82308242 |  |
| cg26578049 | 1.00 | 9.69×10^-9^ | 7.93×10^-5^ | 5 | 87985911 |  |
| cg18368265 | -0.57 | 2.29×10^-9^ | 2.22×10^-5^ | 5 | 123968247 |  |
| cg03795507 | -1.00 | 3.42×10^-7^ | 1.60×10^-3^ | 5 | 133340866 | *VDAC1* |
| cg01919823 | 0.52 | 4.63×10^-6^ | 1.47×10^-2^ | 5 | 148741481 | *PCYOX1L* |
| cg07682547 | 0.88 | 2.81×10^-11^ | 4.50×10^-7^ | 5 | 153863417 |  |
| cg19868864 | 1.00 | 3.77×10^-6^ | 1.24×10^-2^ | 5 | 162901371 | *HMMR* |
| cg04093159 | 0.19 | 9.16×10^-8^ | 5.54×10^-4^ | 5 | 166211825 |  |
| cg15926342 | -0.47 | 6.12×10^-6^ | 1.87×10^-2^ | 5 | 171881757 | *SH3PXD2B* |
| cg26993940 | 0.70 | 1.39×10^-6^ | 5.32×10^-3^ | 5 | 177892758 | *COL23A1* |
| cg16958184 | -1.00 | 2.38×10^-17^ | 1.44×10^-12^ | 6 | 2685592 | *MYLK4* |
| cg01156392 | 0.73 | 2.32×10^-6^ | 8.18×10^-3^ | 6 | 5850005 |  |
| cg09146892 | 0.99 | 2.28×10^-6^ | 8.09×10^-3^ | 6 | 6971335 |  |
| cg24822462 | 0.47 | 4.17×10^-7^ | 1.89×10-3 | 6 | 15097408 |  |
| cg02147727 | 0.74 | 9.85×10^-7^ | 4.05×10^-3^ | 6 | 20500125 |  |
| cg07191121 | 1.00 | 1.12×10^-12^ | 2.65×10^-8^ | 6 | 28724650 |  |
| cg00937691 | -0.97 | 3.43×10^-6^ | 1.14×10^-2^ | 6 | 29796105 | *HLA-G* |
| cg10750989 | -0.45 | 5.89×10^-13^ | 1.52×10^-8^ | 6 | 31371330 | *MICA* |
| cg07235456 | 0.70 | 2.76×10^-7^ | 1.32×10^-3^ | 6 | 31935499 | *SKIV2L* |
| cg13347198 | 1.00 | 6.89×10^-28^ | 1.69×10^-22^ | 6 | 32798198 | *TAP2* |
| cg24070123 | 1.00 | 8.42×10^-9^ | 7.05×10^-5^ | 6 | 34665156 | *C6orf106* |
| cg24222512 | 0.88 | 6.77×10^-6^ | 2.01×10^-2^ | 6 | 38128777 |  |
| cg17004121 | -1.00 | 4.77×10^-9^ | 4.34×10^-5^ | 6 | 43258821 |  |
| cg19254793 | 1.00 | 7.83×10^-12^ | 1.48×10^-7^ | 6 | 44695348 |  |
| cg15347924 | -1.00 | 5.63×10^-9^ | 4.97×10^-5^ | 6 | 74073424 | *C6orf221* |
| cg16047765 | 1.00 | 4.84×10^-6^ | 1.53×10^-2^ | 6 | 98165073 |  |
| cg17388996 | 0.52 | 1.40×10^-9^ | 1.43×10^-5^ | 6 | 108145374 | *SCML4* |
| cg15406010 | 0.99 | 5.67×10^-9^ | 4.97×10^-5^ | 6 | 108478650 |  |
| cg01999537 | 0.69 | 1.47×10^-10^ | 1.97×10^-6^ | 6 | 111176837 | *AMD1* |
| cg17679781 | -0.56 | 1.23×10^-5^ | 3.31×10^-2^ | 6 | 114663223 |  |
| cg15226648 | -0.72 | 1.97×10^-5^ | 4.92×10^-2^ | 6 | 122273447 |  |
| cg20043883 | -1.00 | 1.58×10^-9^ | 1.57×10^-5^ | 6 | 149289357 | *UST* |
| cg16966529 | -1.00 | 3.08×10^-6^ | 1.06×10^-2^ | 6 | 150004505 | *LATS1* |
| cg27478167 | 0.49 | 1.58×10^-5^ | 4.10×10^-2^ | 7 | 817139 | *HEATR2* |
| cg23594597 | 0.61 | 1.28×10^-8^ | 1.04×10^-4^ | 7 | 27444401 |  |
| cg14505161 | 0.30 | 5.86×10^-7^ | 2.59×10^-3^ | 7 | 30544458 | *GGCT* |
| cg04985696 | -0.31 | 8.91×10^-8^ | 5.47×10^-4^ | 7 | 44622204 | *TMED4* |
| cg27528426 | -0.30 | 4.88×10^-14^ | 1.50×10^-9^ | 7 | 65540851 | *ASL* |
| cg18189615 | 1.00 | 1.81×10^-26^ | 3.34×10^-21^ | 7 | 75386749 |  |
| cg16639595 | -0.46 | 2.84×10^-6^ | 9.82×10^-3^ | 7 | 87856984 | *SRI* |
| cg14719076 | 1.00 | 2.05×10^-14^ | 6.85×10^-10^ | 7 | 96653747 | *DLX5* |
| cg17460586 | 1.00 | 8.05×10^-8^ | 5.07×10^-4^ | 7 | 106089713 |  |
| cg27019749 | -1.00 | 1.93×10^-18^ | 1.58×10^-13^ | 7 | 112635781 | *HRAT17* |
| cg05096788 | -0.71 | 1.34×10^-7^ | 7.59×10^-4^ | 7 | 128444910 | *CCDC136* |
| cg13504856 | 1.00 | 1.89×10^-24^ | 2.28×10^-19^ | 7 | 128956859 | *AHCYL2* |
| cg00213189 | 0.52 | 4.53×10^-6^ | 1.45×10^-2^ | 7 | 143012820 | *CLCN1* |
| cg07499066 | 0.88 | 4.54×10^-19^ | 4.18×10^-14^ | 7 | 149171379 | *ZNF746* |
| cg00827082 | 0.66 | 3.54×10^-7^ | 1.64×10^-3^ | 8 | 407482 | *FBXO25* |
| cg12792075 | 1.00 | 1.97×10^-5^ | 4.92×10^-2^ | 8 | 12244565 | *FAM66A* |
| cg21341889 | -0.51 | 5.71×10^-6^ | 1.78×10^-2^ | 8 | 17773367 |  |
| cg07085824 | 0.16 | 1.82×10^-6^ | 6.67×10^-3^ | 8 | 42948105 | *SGK196* |
| cg07370726 | -1.00 | 1.58×10^-7^ | 8.66×10^-4^ | 8 | 47980380 |  |
| cg18979780 | -0.61 | 1.01×10^-5^ | 2.88×10^-2^ | 8 | 57033930 |  |
| cg25356825 | -0.94 | 1.35×10^-7^ | 7.62×10^-4^ | 8 | 59988692 | *TOX* |
| cg04742453 | 0.64 | 1.29×10^-6^ | 5.01×10^-3^ | 8 | 61685545 | *CHD7* |
| cg03214200 | 1.00 | 3.44×10^-17^ | 1.81×10^-12^ | 8 | 65553669 | *CYP7B1* |
| cg17380533 | 0.60 | 1.63×10^-9^ | 1.60×10^-5^ | 8 | 72875102 | *MSC-AS1* |
| cg07961887 | 0.12 | 1.26×10^-6^ | 4.95×10^-3^ | 8 | 82192601 | *FABP5* |
| cg23048068 | 0.13 | 3.19×10^-7^ | 1.51×10^-3^ | 8 | 82192604 | *FABP5* |
| cg13076537 | 0.25 | 3.22×10^-11^ | 4.94×10^-7^ | 8 | 82192945 | *FABP5* |
| cg14317230 | 1.00 | 1.85×10^-6^ | 6.71×10^-3^ | 8 | 98708278 | *MTDH* |
| cg22516182 | 1.00 | 1.82×10^-15^ | 8.39×10^-11^ | 8 | 102400152 |  |
| cg02526790 | -1.00 | 1.97×10^-11^ | 3.31×10^-7^ | 8 | 134044590 | *TG* |
| cg11768833 | -1.00 | 5.91×10^-10^ | 6.70×10^-6^ | 9 | 13140958 | *MPDZ* |
| cg25958241 | 1.00 | 6.70×10^-13^ | 1.65×10^-8^ | 9 | 35278307 | *UNC13B* |
| cg03081134 | -0.28 | 6.53×10^-6^ | 1.97×10^-2^ | 9 | 36036806 | *RECK* |
| cg09762316 | -1.00 | 1.04×10^-5^ | 2.93×10^-2^ | 9 | 72658354 | *MAMDC2* |
| cg14571425 | 0.89 | 2.36×10^-6^ | 8.28×10^-3^ | 9 | 109494762 |  |
| cg01760174 | -0.97 | 1.83×10^-7^ | 9.63×10^-4^ | 9 | 109879157 |  |
| cg13469953 | -0.44 | 3.23×10^-6^ | 1.10×10^-2^ | 9 | 117271936 |  |
| cg19890156 | 1.00 | 1.85×10^-6^ | 6.71×10^-3^ | 9 | 139934859 | *NPDC1* |
| cg18535642 | 1.00 | 1.18×10^-6^ | 4.71×10^-3^ | 10 | 8080012 |  |
| cg02523824 | 0.97 | 3.69×10^-7^ | 1.68×10^-3^ | 10 | 11298786 | *CUGBP2* |
| cg12629936 | 0.75 | 6.68×10^-11^ | 9.65×10^-7^ | 10 | 12250941 | *CDC123* |
| cg00748492 | -1.00 | 6.91×10^-9^ | 5.92×10^-5^ | 10 | 22216446 | *DNAJC1* |
| cg24914935 | 0.44 | 1.30×10^-6^ | 5.03×10^-3^ | 10 | 30589494 |  |
| cg17504814 | 0.43 | 9.39×10^-6^ | 2.68×10^-2^ | 10 | 35540771 | *CCNY* |
| cg25692425 | -0.56 | 1.51×10^-5^ | 3.94×10^-2^ | 10 | 61721716 | *C10orf40* |
| cg08234376 | 0.71 | 1.42×10^-6^ | 5.38×10^-3^ | 10 | 70100076 | *HNRNPH3* |
| cg19411943 | 0.69 | 2.21×10^-7^ | 1.09×10^-3^ | 10 | 75306538 | *USP54* |
| cg24603634 | -0.59 | 9.27×10^-6^ | 2.67×10^-2^ | 10 | 90925301 |  |
| cg06930332 | 1.00 | 7.03×10^-10^ | 7.75×10^-6^ | 10 | 102006954 | *CWF19L1* |
| cg15640580 | -1.00 | 4.67×10^-7^ | 2.09×10^-3^ | 10 | 115076373 |  |
| cg04465599 | -0.09 | 1.32×10^-7^ | 7.55×10^-4^ | 10 | 121652136 | *SEC23IP* |
| cg17642426 | 0.96 | 1.61×10^-15^ | 7.90×10^-11^ | 10 | 124739963 | *PSTK* |
| cg09216081 | 1.00 | 5.96×10^-13^ | 1.52×10^-8^ | 10 | 134940484 | *GPR123* |
| cg27010328 | 1.00 | 7.69×10^-29^ | 5.67×10^-23^ | 11 | 238081 | *PSMD13* |
| cg22408430 | -0.93 | 4.66×10^-8^ | 3.18×10^-4^ | 11 | 63883266 | *MACROD1* |
| cg10257063 | -0.71 | 2.59×10^-6^ | 9.04×10^-3^ | 11 | 68587159 | *CPT1A* |
| cg06318893 | -0.84 | 1.58×10^-7^ | 8.66×10^-4^ | 11 | 75097167 |  |
| cg19815354 | 1.00 | 6.20×10^-7^ | 2.70×10^-3^ | 11 | 77450740 | *RSF1* |
| cg21451530 | -0.63 | 1.19×10^-6^ | 4.72×10^-3^ | 11 | 102115640 |  |
| cg16975613 | 1.00 | 2.66×10^-12^ | 5.77×10^-8^ | 11 | 102217719 | *BIRC2* |
| cg05962325 | -0.93 | 1.09×10^-7^ | 6.42×10^-4^ | 11 | 107992005 | *ACAT1* |
| cg26572438 | 1.00 | 4.22×10^-8^ | 2.93×10^-4^ | 11 | 118123982 | *MPZL3* |
| cg18224077 | -0.52 | 8.27×10^-6^ | 2.40×10^-2^ | 11 | 121972185 | *LOC399959* |
| cg06038239 | 0.94 | 1.48×10^-7^ | 8.23×10^-4^ | 11 | 123612522 | *ZNF202* |
| cg19649564 | 0.97 | 3.08×10^-18^ | 2.27×10^-13^ | 11 | 124932892 | *SLC37A2* |
| cg19469189 | -0.56 | 1.07×10^-7^ | 6.34×10^-4^ | 11 | 128781035 | *KCNJ5* |
| cg11550340 | 0.68 | 1.79×10^-8^ | 1.39×10^-4^ | 12 | 3217332 | *TSPAN9* |
| cg18961101 | -0.62 | 9.33×10^-6^ | 2.68×10^-2^ | 12 | 4923116 | *KCNA6* |
| cg02074878 | -1.00 | 1.21×10^-11^ | 2.17×10^-7^ | 12 | 10961294 |  |
| cg19532939 | -0.91 | 1.83×10^-5^ | 4.64×10^-2^ | 12 | 14996525 | *ART4* |
| cg02325300 | 0.72 | 3.64×10^-13^ | 9.95×10^-9^ | 12 | 16758836 | *LMO3* |
| cg10873203 | 0.67 | 4.29×10^-12^ | 8.31×10^-8^ | 12 | 25942772 |  |
| cg06405045 | -0.56 | 9.06×10^-8^ | 5.51×10^-4^ | 12 | 31479141 | *FAM60A* |
| cg25598488 | -0.89 | 8.80×10^-7^ | 3.70×10^-3^ | 12 | 34258505 |  |
| cg10736528 | 1.00 | 7.20×10^-6^ | 2.12×10^-2^ | 12 | 52772961 | *KRT84* |
| cg17129918 | 0.87 | 5.99×10^-7^ | 2.63×10^-3^ | 12 | 53098560 | *KRT77* |
| cg01708411 | 1.00 | 1.20×10^-9^ | 1.28×10^-5^ | 12 | 57631839 | *NDUFA4L2* |
| cg26223054 | -0.68 | 4.74×10^-9^ | 4.34×10^-5^ | 12 | 58201681 | *AVIL* |
| cg05691053 | 0.43 | 5.08×10^-6^ | 1.59×10^-2^ | 12 | 72104344 |  |
| cg14529331 | 1.00 | 2.32×10^-7^ | 1.13×10^-3^ | 12 | 97856995 |  |
| cg03916864 | -0.49 | 1.17×10^-5^ | 3.18×10^-2^ | 12 | 109535384 | *UNG* |
| cg11550950 | -0.92 | 4.20×10^-6^ | 1.36×10^-2^ | 13 | 32297002 |  |
| cg11772527 | -0.70 | 1.88×10^-6^ | 6.77×10^-3^ | 13 | 94161642 | *GPC6* |
| cg15858426 | 0.69 | 5.10×10^-9^ | 4.59×10^-5^ | 13 | 95252369 |  |
| cg15953892 | -0.59 | 1.61×10^-10^ | 2.12×10^-6^ | 13 | 111672726 |  |
| cg25983594 | -0.68 | 6.64×10^-6^ | 1.99×10^-2^ | 13 | 113650922 | *MCF2L* |
| cg15852141 | 0.51 | 1.97×10^-7^ | 1.00×10^-3^ | 14 | 22891383 |  |
| cg06243610 | -0.43 | 1.49×10^-5^ | 3.91×10^-2^ | 14 | 52373839 | *GNG2* |
| cg00018758 | -0.52 | 1.59×10^-7^ | 8.66×10^-4^ | 14 | 54936774 |  |
| cg21140981 | 1.00 | 3.24×10^-6^ | 1.10×10^-2^ | 14 | 91766264 | *CCDC88C* |
| cg05794283 | 0.87 | 1.12×10^-5^ | 3.09×10^-2^ | 14 | 93686639 | *UBR7* |
| cg07445547 | 0.59 | 1.86×10^-6^ | 6.71×10^-3^ | 14 | 101193006 | *DLK1* |
| cg11185653 | -0.84 | 3.44×10^-7^ | 1.60×10^-3^ | 14 | 105669480 |  |
| cg22241833 | -0.47 | 1.15×10^-6^ | 4.63×10-3 | 15 | 25415399 | *SNORD115-1* |
| cg26995364 | 1.00 | 2.16×10^-24^ | 2.28×10^-19^ | 15 | 29336425 | *APBA2* |
| cg09289674 | 0.56 | 6.72×10^-7^ | 2.91×10^-3^ | 15 | 51699392 | *GLDN* |
| cg07947039 | -1.00 | 3.95×10^-10^ | 4.62×10^-6^ | 15 | 56292422 |  |
| cg26409497 | 0.56 | 1.38×10^-5^ | 3.65×10^-2^ | 15 | 58331384 | *ALDH1A2* |
| cg15100456 | 0.41 | 1.17×10^-5^ | 3.17×10^-2^ | 15 | 68809904 |  |
| cg27514624 | 1.00 | 1.09×10^-17^ | 7.33×10^-13^ | 15 | 69706456 | *KIF23* |
| cg09585074 | 1.00 | 2.60×10^-13^ | 7.36×10^-9^ | 15 | 72638395 | *HEXA* |
| cg21406075 | 0.52 | 3.42×10^-6^ | 1.14×10^-2^ | 15 | 96906354 |  |
| cg05477921 | 0.65 | 1.88×10^-8^ | 1.43×10^-4^ | 15 | 100685723 | *ADAMTS17* |
| cg11169286 | 0.17 | 6.66×10^-6^ | 1.99×10^-2^ | 16 | 686434 | *C16orf13* |
| cg16784970 | -0.53 | 1.87×10^-7^ | 9.63×10^-4^ | 16 | 2123215 | *TSC2* |
| cg02560615 | -0.39 | 4.41×10^-6^ | 1.41×10^-2^ | 16 | 2274210 | *E4F1* |
| cg14939446 | 0.39 | 2.71×10^-6^ | 9.41×10^-3^ | 16 | 2571015 | *AMDHD2* |
| cg00454447 | 0.65 | 2.32×10^-7^ | 1.13×10^-3^ | 16 | 3728185 | *TRAP1* |
| cg26570867 | 0.49 | 2.70×10^-7^ | 1.30×10^-3^ | 16 | 12308181 | *SNX29* |
| cg19415738 | -0.67 | 1.46×10^-8^ | 1.15×10^-4^ | 16 | 30418482 | *ZNF771* |
| cg18129863 | -0.70 | 7.74×10^-8^ | 4.92×10^-4^ | 16 | 30886426 | *MIR762HG* |
| cg05438708 | -0.50 | 5.84×10^-6^ | 1.81×10^-2^ | 16 | 81520178 | *CMIP* |
| cg16777782 | 0.61 | 1.96×10^-10^ | 2.53×10^-6^ | 16 | 82671333 | *CDH13* |
| cg16053428 | -0.41 | 1.09×10^-6^ | 4.41×10^-3^ | 16 | 83990544 | *OSGIN1* |
| cg06925882 | -0.38 | 3.38×10^-8^ | 2.39×10^-4^ | 16 | 85157885 |  |
| cg07391271 | -0.68 | 8.19×10^-9^ | 6.94×10^-5^ | 16 | 89588846 | *SPG7* |
| cg01466631 | 0.38 | 1.73×10^-6^ | 6.38×10^-3^ | 17 | 1094369 |  |
| cg03477621 | 0.83 | 7.11×10^-6^ | 2.10×10^-2^ | 17 | 9472738 | *STX8* |
| cg11367590 | 0.98 | 2.92×10^-7^ | 1.39×10^-3^ | 17 | 17226082 | *NT5M* |
| cg10228608 | 0.57 | 1.21×10^-9^ | 1.28×10^-5^ | 17 | 19804476 |  |
| cg02558505 | 0.15 | 3.93×10^-11^ | 5.92×10^-7^ | 17 | 21156701 | *NATD1* |
| cg17235374 | 0.93 | 1.84×10^-7^ | 9.63×10^-4^ | 17 | 27717757 | *TAOK1* |
| cg11295144 | -0.58 | 7.52×10^-7^ | 3.22×10^-3^ | 17 | 33447001 | *RAD51L3* |
| cg21336116 | 0.95 | 1.39×10^-6^ | 5.32×10^-3^ | 17 | 39093658 | *KRT23* |
| cg16630982 | -0.20 | 3.31×10^-6^ | 1.12×10^-2^ | 17 | 41277394 | *BRCA1* |
| cg12182452 | -0.35 | 1.14×10^-5^ | 3.12×10^-2^ | 17 | 41277730 | *BRCA1* |
| cg17182054 | -0.46 | 3.97×10^-6^ | 1.29×10^-2^ | 17 | 54588075 |  |
| cg05031081 | 0.66 | 2.07×10^-7^ | 1.04×10^-3^ | 17 | 72239436 | *TTYH2* |
| cg05464572 | 0.79 | 4.48×10^-7^ | 2.01×10^-3^ | 17 | 72694068 | *RAB37* |
| cg14022616 | 0.73 | 7.13×10^-8^ | 4.61×10^-4^ | 17 | 72765872 |  |
| cg07027472 | 0.47 | 1.13×10^-7^ | 6.57×10^-4^ | 17 | 73391422 | *GRB2* |
| cg19169246 | 0.48 | 2.15×10^-11^ | 3.52×10^-7^ | 17 | 73887208 | *TRIM65* |
| cg14165663 | 1.00 | 1.80×10^-5^ | 4.58×10^-2^ | 17 | 74070269 | *GALR2* |
| cg15275493 | 0.88 | 1.53×10^-9^ | 1.55×10^-5^ | 17 | 75310725 | *9-Sep* |
| cg26351916 | 0.99 | 8.47×10^-8^ | 5.27×10^-4^ | 17 | 78084228 | *GAA* |
| cg16681349 | 0.57 | 9.56×10^-7^ | 3.98×10^-3^ | 17 | 80614369 |  |
| cg18089670 | 0.86 | 3.36×10^-6^ | 1.13×10^-2^ | 18 | 660756 | *TYMS* |
| cg24614245 | 0.55 | 1.02×10^-5^ | 2.90×10^-2^ | 18 | 6603943 |  |
| cg23286333 | -1.00 | 1.54×10^-6^ | 5.74×10^-3^ | 18 | 39112519 |  |
| cg04870412 | 0.66 | 2.99×10^-10^ | 3.61×10^-6^ | 18 | 54655403 | *WDR7* |
| cg26635170 | 0.42 | 1.49×10^-5^ | 3.91×10^-2^ | 18 | 55907926 | *NEDD4L* |
| cg13204333 | -1.00 | 5.13×10^-8^ | 3.47×10^-4^ | 18 | 58041198 | *MC4R* |
| cg25185915 | 0.63 | 1.10×10^-10^ | 1.50×10^-6^ | 18 | 74507281 | *LOC100131655* |
| cg17185889 | -0.36 | 6.03×10^-6^ | 1.85×10^-2^ | 18 | 74685615 |  |
| cg17102627 | 0.65 | 6.42×10^-6^ | 1.95×10^-2^ | 18 | 77230598 | *NFATC1* |
| cg17151991 | -0.52 | 1.42×10^-6^ | 5.38×10^-3^ | 19 | 1207421 | *STK11* |
| cg19561908 | -0.80 | 7.58×10^-7^ | 3.23×10^-3^ | 19 | 2859106 |  |
| cg04272577 | -0.73 | 9.82×10^-11^ | 1.37×10^-6^ | 19 | 4258906 | *CCDC94* |
| cg09510776 | 0.90 | 1.09×10^-5^ | 3.04×10^-2^ | 19 | 4729882 |  |
| cg22933199 | -1.00 | 1.76×10^-12^ | 4.05×10^-8^ | 19 | 12163909 | *ZNF878* |
| cg20276511 | -0.22 | 4.89×10^-15^ | 2.11×10^-10^ | 19 | 15543527 | *WIZ* |
| cg23397718 | -1.00 | 3.61×10^-7^ | 1.66×10^-3^ | 19 | 18630997 | *ELL* |
| cg04810466 | -1.00 | 9.68×10^-7^ | 4.01×10^-3^ | 19 | 30914969 | *ZNF536* |
| cg03422185 | 1.00 | 5.15×10^-15^ | 2.11×10^-10^ | 19 | 41921278 | *BCKDHA* |
| cg26020365 | 0.84 | 3.79×10^-12^ | 7.77×10^-8^ | 19 | 44932260 | *ZNF229* |
| cg01009059 | 0.71 | 1.47×10^-14^ | 5.41×10^-10^ | 19 | 46391024 |  |
| cg26375855 | 0.64 | 4.61×10^-9^ | 4.30×10^-5^ | 19 | 54299976 | *NLRP12* |
| cg14326053 | -0.75 | 1.50×10^-8^ | 1.18×10^-4^ | 20 | 371146 | *TRIB3* |
| cg05927427 | 0.54 | 8.78×10^-9^ | 7.27×10^-5^ | 20 | 25187813 | *ENTPD6* |
| cg00514271 | -0.52 | 6.73×10^-6^ | 2.01×10^-2^ | 20 | 30593353 |  |
| cg07273583 | 0.62 | 2.81×10^-8^ | 2.05×10^-4^ | 20 | 34516631 | *PHF20* |
| cg26364766 | 0.68 | 3.34×10^-12^ | 7.03×10^-8^ | 20 | 48570238 | *RNF114* |
| cg05494607 | -0.48 | 3.80×10^-6^ | 1.25×10^-2^ | 20 | 50416707 | *SALL4* |
| cg12750641 | 0.55 | 2.23×10^-8^ | 1.67×10^-4^ | 20 | 50772956 | *ZFP64* |
| cg17734137 | -0.33 | 1.95×10^-5^ | 4.90×10^-2^ | 20 | 58647209 | *C20orf197* |
| cg01320322 | -0.77 | 5.38×10^-10^ | 6.19×10^-6^ | 21 | 27206630 |  |
| cg24444091 | 0.86 | 5.18×10^-6^ | 1.62×10^-2^ | 21 | 27462541 | *APP* |
| cg01158367 | 0.82 | 1.48×10^-6^ | 5.56×10^-3^ | 21 | 30258251 | *N6AMT1* |
| cg02330424 | 1.00 | 6.89×10^-8^ | 4.53×10^-4^ | 22 | 21135726 | *SERPIND1* |
| cg05532013 | 0.60 | 1.86×10^-7^ | 9.63×10^-4^ | 22 | 23650781 | *BCR* |
| cg23185207 | -0.39 | 1.93×10^-5^ | 4.87×10^-2^ | 22 | 27705236 |  |
| cg04696109 | 0.56 | 2.86×10^-14^ | 9.16×10^-10^ | 22 | 36081855 |  |
| cg10027934 | -0.94 | 1.27×10^-7^ | 7.31×10^-4^ | 22 | 39799092 | *MAP3K7IP1* |
| cg08762150 | -0.41 | 1.31×10^-5^ | 3.49×10^-2^ | 22 | 44644994 | *KIAA1644* |
| cg26679753 | 0.61 | 4.22×10^-6^ | 1.36×10^-2^ | 22 | 46747353 | *TRMU* |
| **Eosinophils** | | | | | | |
| cg25769755 | 1.00 | 4.63×10^-7^ | 1.44×10^-3^ | 1 | 1342720 | *MRPL20* |
| cg06633963 | -1.00 | 8.87×10^-13^ | 5.84×10^-9^ | 1 | 5938826 | *NPHP4* |
| cg11723698 | -1.00 | 1.87×10^-17^ | 1.51×10^-13^ | 1 | 6325058 | *ACOT7* |
| cg09513469 | -1.00 | 2.42×10^-6^ | 6.19×10^-3^ | 1 | 6421502 | *ACOT7* |
| cg17594860 | 1.00 | 1.22×10^-6^ | 3.41×10^-3^ | 1 | 19615358 | *AKR7A3* |
| cg05937630 | 1.00 | 8.76×10^-6^ | 1.88×10^-2^ | 1 | 19615480 | *AKR7A3* |
| cg07909786 | 1.00 | 1.52×10^-8^ | 6.65×10^-5^ | 1 | 21444331 | *EIF4G3* |
| cg07882021 | 1.00 | 3.28×10^-7^ | 1.07×10^-3^ | 1 | 26989497 |  |
| cg04123310 | -1.00 | 2.12×10^-12^ | 1.33×10^-8^ | 1 | 27677072 | *SYTL1* |
| cg15449899 | 1.00 | 4.44×10^-6^ | 1.07×10^-2^ | 1 | 40367671 | *MYCL* |
| cg03317400 | -1.00 | 1.78×10^-5^ | 3.48×10^-2^ | 1 | 40420823 | *MFSD2A* |
| cg07313701 | 1.00 | 4.43×10^-6^ | 1.07×10^-2^ | 1 | 42447165 |  |
| cg21445612 | -1.00 | 2.33×10^-9^ | 1.13×10^-5^ | 1 | 47504028 | *CYP4X1* |
| cg12911732 | 1.00 | 1.32×10^-8^ | 5.84×10^-5^ | 1 | 50744322 |  |
| cg08702671 | 1.00 | 4.99×10^-6^ | 1.17×10^-2^ | 1 | 52093583 | *OSBPL9* |
| cg21655710 | -1.00 | 1.91×10^-5^ | 3.69×10^-2^ | 1 | 54856511 | *SSBP3* |
| cg19633027 | -1.00 | 9.05×10^-10^ | 4.67×10^-6^ | 1 | 56279464 |  |
| cg26457504 | 1.00 | 6.19×10^-6^ | 1.40×10^-2^ | 1 | 75199144 | *CRYZ* |
| cg22618350 | -1.00 | 3.59×10^-6^ | 8.81×10^-3^ | 1 | 91487603 | *ZNF644* |
| cg27001914 | -1.00 | 5.70×10^-85^ | 2.21×10^-80^ | 1 | 97215359 | *PTBP2* |
| cg11904114 | -1.00 | 7.30×10^-8^ | 2.70×10^-4^ | 1 | 100294993 |  |
| cg17138369 | 1.00 | 6.73×10^-8^ | 2.54×10^-4^ | 1 | 153606383 | *C1orf77* |
| cg07653043 | -1.00 | 2.75×10^-32^ | 4.51×10^-28^ | 1 | 165854897 | *UCK2* |
| cg25977304 | -1.00 | 2.01×10^-6^ | 5.25×10^-3^ | 1 | 167189793 | *POU2F1* |
| cg05001758 | -1.00 | 3.92×10^-7^ | 1.25×10^-3^ | 1 | 173794044 | *CENPL* |
| cg18941211 | -1.00 | 1.40×10^-18^ | 1.23×10^-14^ | 1 | 179266761 | *SOAT1* |
| cg11649969 | -1.00 | 2.50×10^-5^ | 4.62×10^-2^ | 1 | 180940378 |  |
| cg15569801 | -1.00 | 1.89×10^-98^ | 8.19×10^-94^ | 1 | 184197096 |  |
| cg19600670 | -1.00 | 2.37×10^-5^ | 4.44×10^-2^ | 1 | 193090350 | *CDC73* |
| cg10021424 | -1.00 | 4.32×10^-9^ | 2.02×10^-5^ | 1 | 206615585 | *SRGAP2* |
| cg03778523 | -1.00 | 1.09×10^-5^ | 2.29×10^-2^ | 1 | 207125141 |  |
| cg10113157 | -1.00 | 8.13×10^-7^ | 2.35×10^-3^ | 1 | 207666347 |  |
| cg06123700 | -1.00 | 2.27×10^-5^ | 4.29×10^-2^ | 1 | 211430780 |  |
| cg03327327 | -1.00 | 8.35×10^-7^ | 2.39×10^-3^ | 1 | 214779620 | *CENPF* |
| cg05531018 | -1.00 | 1.50×10^-5^ | 3.02×10^-2^ | 1 | 226788767 | *C1orf95* |
| cg16132288 | -1.00 | 1.22×10^-19^ | 1.15×10^-15^ | 1 | 228141133 |  |
| cg23777318 | -1.00 | 1.72×10^-57^ | 4.23×10^-53^ | 1 | 228332970 | *GUK1* |
| cg03997773 | -1.00 | 5.04×10^-23^ | 5.81×10^-19^ | 1 | 230992011 | *C1orf198* |
| cg13584052 | -1.00 | 2.26×10^-12^ | 1.41×10^-8^ | 1 | 235079078 |  |
| cg04826576 | -1.00 | 4.65×10^-6^ | 1.10×10^-2^ | 1 | 244816905 | *PPPDE1* |
| cg23369767 | -1.00 | 8.24×10-6 | 1.77×10^-2^ | 2 | 1730937 | *PXDN* |
| cg10276650 | -1.00 | 2.05×10-20 | 2.07×10^-16^ | 2 | 3245997 | *TSSC1* |
| cg04161956 | -1.00 | 1.49×10^-79^ | 4.98×10^-75^ | 2 | 11975650 |  |
| cg03381515 | -1.00 | 1.79×10^-7^ | 6.05×10^-4^ | 2 | 20251148 | *LAPTM4A* |
| cg03927729 | -1.00 | 5.23×10^-10^ | 2.79×10^-6^ | 2 | 33713028 | *RASGRP3* |
| cg04795282 | -1.00 | 6.86×10^-7^ | 2.02×10^-3^ | 2 | 37311583 | *HEATR5B* |
| cg27428208 | -1.00 | 3.60×10^-45^ | 7.16×10^-41^ | 2 | 42229179 |  |
| cg01560354 | -1.00 | 2.45×10^-5^ | 4.55×10^-2^ | 2 | 43767446 | *THADA* |
| cg25370657 | -1.00 | 4.16×10^-28^ | 5.89×10^-24^ | 2 | 44383264 |  |
| cg16098955 | -1.00 | 5.73×10^-13^ | 3.91×10^-9^ | 2 | 55172992 | *EML6* |
| cg14406727 | -1.00 | 1.22×10^-7^ | 4.38×10^-4^ | 2 | 65577769 | *SPRED2* |
| cg16986851 | -1.00 | 7.64×10^-6^ | 1.66×10^-2^ | 2 | 73052567 | *EXOC6B* |
| cg24223207 | -1.00 | 1.96×10^-19^ | 1.81×10^-15^ | 2 | 75830828 |  |
| cg26209001 | -1.00 | 1.21×10^-5^ | 2.50×10^-2^ | 2 | 100874495 |  |
| cg07297467 | -1.00 | 3.15×10^-13^ | 2.19×10^-9^ | 2 | 101635691 | *RPL31* |
| cg09352202 | -1.00 | 6.53×10^-7^ | 1.94×10^-3^ | 2 | 102314757 | *MAP4K4* |
| cg09587345 | -1.00 | 1.96×10^-23^ | 2.33×10^-19^ | 2 | 102437128 | *MAP4K4* |
| cg26372201 | -1.00 | 3.65×10^-9^ | 1.75×10^-5^ | 2 | 113815433 | *IL36RN* |
| cg00583406 | -1.00 | 1.72×10^-137^ | 1.27×10^-132^ | 2 | 120511525 |  |
| cg09287938 | -1.00 | 6.98×10^-46^ | 1.47×10^-41^ | 2 | 128186258 | *PROC* |
| cg27386168 | -1.00 | 1.16×10^-98^ | 5.35×10^-94^ | 2 | 130136834 |  |
| cg09255493 | -1.00 | 1.07×10^-218^ | 9.89×10^-214^ | 2 | 130897863 | *CCDC74B* |
| cg02204529 | -1.00 | 3.25×10^-11^ | 1.95×10^-7^ | 2 | 132215465 |  |
| cg06754557 | 1.00 | 1.85×10^-5^ | 3.58×10^-2^ | 2 | 169025892 | *STK39* |
| cg21654303 | -1.00 | 1.61×10^-6^ | 4.32×10^-3^ | 2 | 176866349 | *KIAA1715* |
| cg17287767 | -1.00 | 4.02×10^-8^ | 1.59×10^-4^ | 2 | 192015279 | *STAT4* |
| cg20299002 | -1.00 | 4.09×10^-7^ | 1.29×10^-3^ | 2 | 197036414 | *STK17B* |
| cg20577027 | 1.00 | 2.31×10^-5^ | 4.35×10^-2^ | 2 | 198651356 | *BOLL* |
| cg10543582 | -1.00 | 1.05×10^-40^ | 1.89×10^-36^ | 2 | 199202432 |  |
| cg25945659 | -1.00 | 6.80×10^-13^ | 4.59×10^-9^ | 2 | 202591000 | *ALS2* |
| cg14706579 | 1.00 | <5.00×10^-324^ | <5.00×10^-324^ | 2 | 203736710 | *ICA1L* |
| cg04184623 | -1.00 | 2.14×10^-5^ | 4.06×10^-2^ | 2 | 217277352 | *SMARCAL1* |
| cg27385421 | -1.00 | 1.53×10^-5^ | 3.08×10^-2^ | 2 | 219982256 | *NHEJ1* |
| cg05103387 | -1.00 | 1.04×10^-7^ | 3.79×10^-4^ | 2 | 224702699 | *AP1S3* |
| cg14824579 | -1.00 | 1.10×10^-6^ | 3.09×10^-3^ | 2 | 232646096 | *PDE6D* |
| cg06632323 | -1.00 | 9.60×10^-13^ | 6.21×10^-9^ | 2 | 234669143 | *UGT1A1* |
| cg20628003 | -1.00 | 2.81×10^-6^ | 7.06×10^-3^ | 2 | 242195317 | *HDLBP* |
| cg19024381 | 1.00 | 1.95×10^-6^ | 5.15×10^-3^ | 2 | 242626441 | *DTYMK* |
| cg00916365 | -1.00 | 7.63×10^-13^ | 5.06×10^-9^ | 3 | 13188247 |  |
| cg07624815 | 1.00 | 3.90×10^-7^ | 1.25×10^-3^ | 3 | 14922656 | *FGD5* |
| cg19719475 | -1.00 | 2.06×10^-14^ | 1.53×10^-10^ | 3 | 23849114 | *UBE2E1* |
| cg05145393 | -1.00 | 3.38×10^-23^ | 3.95×10^-19^ | 3 | 27404197 | *NEK10* |
| cg14340541 | -1.00 | 4.60×10^-6^ | 1.10×10^-2^ | 3 | 27691539 |  |
| cg02725123 | -1.00 | 2.17×10^-6^ | 5.63×10^-3^ | 3 | 32619278 |  |
| cg17167289 | -1.00 | 1.55×10^-6^ | 4.23×10^-3^ | 3 | 42846087 | *HIGD1A* |
| cg01648578 | -1.00 | 1.73×10^-5^ | 3.41×10^-2^ | 3 | 45233625 |  |
| cg13510812 | 1.00 | 2.46×10^-5^ | 4.57×10^-2^ | 3 | 45429626 | *LARS2* |
| cg17683948 | 1.00 | 4.75×10^-6^ | 1.12×10^-2^ | 3 | 45429800 | *LARS2* |
| cg22887981 | -1.00 | 7.39×10^-10^ | 3.83×10^-6^ | 3 | 46530353 |  |
| cg13293757 | 1.00 | 1.23×10^-6^ | 3.41×10^-3^ | 3 | 48487975 | *ATRIP* |
| cg04244987 | 1.00 | 9.05×10^-7^ | 2.57×10^-3^ | 3 | 48723638 | *NCKIPSD* |
| cg11704396 | 1.00 | 4.69×10^-10^ | 2.54×10^-6^ | 3 | 48723701 | *NCKIPSD* |
| cg19392504 | -1.00 | 1.73×10^-10^ | 9.68×10^-7^ | 3 | 57811622 | *SLMAP* |
| cg14844401 | -1.00 | 1.99×10^-13^ | 1.43×10^-9^ | 3 | 123133741 | *ADCY5* |
| cg03021329 | 1.00 | 1.16×10^-9^ | 5.94×10^-6^ | 3 | 124554058 | *ITGB5* |
| cg04059835 | 1.00 | 1.64×10^-7^ | 5.60×10^-4^ | 3 | 128598095 | *ACAD9* |
| cg11943389 | -1.00 | 8.07×10^-26^ | 1.08×10^-21^ | 3 | 132231785 | *DNAJC13* |
| cg03474609 | -1.00 | 2.08×10^-25^ | 2.69×10^-21^ | 3 | 156207194 | *KCNAB1* |
| cg12215541 | -1.00 | 6.39×10^-19^ | 5.81×10^-15^ | 3 | 169171418 | *MECOM* |
| cg07777225 | 1.00 | 2.62×10-7 | 8.64×10^-4^ | 3 | 179244465 |  |
| cg18214045 | -1.00 | 1.43×10^-46^ | 3.20×10^-42^ | 3 | 182225799 |  |
| cg12976555 | -1.00 | 1.18×10^-5^ | 2.43×10^-2^ | 3 | 184917727 | *EHHADH* |
| cg20184464 | -1.00 | 6.73×10^-16^ | 5.22×10^-12^ | 3 | 187739371 |  |
| cg18084083 | -1.00 | 5.15×10^-6^ | 1.20×10^-2^ | 3 | 190232258 | *IL1RAP* |
| cg05480120 | -1.00 | 1.33×10^-5^ | 2.70×10^-2^ | 3 | 194097104 |  |
| cg25276849 | 1.00 | 7.14×10^-7^ | 2.09×10^-3^ | 3 | 195808919 | *TFRC* |
| cg19860178 | -1.00 | 2.10×10^-8^ | 8.83×10^-5^ | 3 | 196594579 | *SENP5* |
| cg11411884 | 1.00 | 6.02×10^-7^ | 1.82×10^-3^ | 4 | 1016333 | *FGFRL1* |
| cg18406106 | -1.00 | 3.16×10^-7^ | 1.04×10^-3^ | 4 | 1198308 | *LOC100130872* |
| cg12940965 | 1.00 | 1.20×10^-8^ | 5.35×10^-5^ | 4 | 1580944 |  |
| cg27636047 | 1.00 | 6.54×10^-10^ | 3.42×10^-6^ | 4 | 1581088 |  |
| cg00935307 | 1.00 | 3.91×10^-13^ | 2.69×10^-9^ | 4 | 1581921 |  |
| cg23502023 | 1.00 | 9.88×10^-18^ | 8.09×10^-14^ | 4 | 1581993 |  |
| cg19704288 | 1.00 | 2.96×10^-11^ | 1.79×10^-7^ | 4 | 1582181 |  |
| cg08604533 | 1.00 | 2.15×10^-18^ | 1.86×10^-14^ | 4 | 1582248 |  |
| cg23495837 | -1.00 | 1.09×10^-5^ | 2.29×10^-2^ | 4 | 3480318 | *DOK7* |
| cg20946504 | -1.00 | 6.96×10^-6^ | 1.55×10^-2^ | 4 | 69215786 | *YTHDC1* |
| cg07944258 | -1.00 | 4.03×10^-9^ | 1.91×10^-5^ | 4 | 76407419 | *RCHY1* |
| cg00685004 | -1.00 | 8.06×10^-6^ | 1.74×10^-2^ | 4 | 83752663 | *SEC31A* |
| cg22404125 | -1.00 | 3.07×10^-43^ | 5.81×10^-39^ | 4 | 99402843 | *TSPAN5* |
| cg19899561 | -1.00 | 3.33×10^-29^ | 4.91×10^-25^ | 4 | 104059265 | *CENPE* |
| cg14451216 | -1.00 | 1.39×10^-122^ | 7.88×10^-118^ | 4 | 110751842 | *RRH* |
| cg09618380 | -1.00 | 4.18×10^-6^ | 1.02×10^-2^ | 4 | 123839727 | *NUDT6* |
| cg11779273 | 1.00 | 9.06×10^-76^ | 2.90×10^-71^ | 4 | 128553930 | *INTU* |
| cg12771751 | -1.00 | 2.44×10^-7^ | 8.10×10^-4^ | 4 | 128647957 |  |
| cg23979876 | -1.00 | 5.03×10^-6^ | 1.18×10^-2^ | 4 | 141299001 | *SCOC* |
| cg14138747 | -1.00 | 1.04×10^-12^ | 6.68×10^-9^ | 4 | 141825221 | *RNF150* |
| cg14202946 | -1.00 | 6.28×10^-7^ | 1.87×10^-3^ | 4 | 169931448 | *CBR4* |
| cg26296532 | -1.00 | 6.44×10^-6^ | 1.46×10^-2^ | 4 | 186514057 | *SORBS2* |
| cg14684251 | -1.00 | 1.61×10^-6^ | 4.32×10^-3^ | 4 | 187687178 |  |
| cg17751153 | -1.00 | 1.42×10^-7^ | 4.95×10^-4^ | 5 | 447880 | *EXOC3* |
| cg02314394 | -1.00 | 5.71×10^-7^ | 1.74×10^-3^ | 5 | 646139 | *CEP72* |
| cg23270724 | -1.00 | 7.95×10^-6^ | 1.72×10^-2^ | 5 | 23466515 |  |
| cg19483803 | -1.00 | 7.94×10^-6^ | 1.72×10^-2^ | 5 | 37044054 | *NIPBL* |
| cg26124136 | -1.00 | 2.74×10^-8^ | 1.12×10^-4^ | 5 | 59442548 | *PDE4D* |
| cg08728761 | 1.00 | 1.37×10^-7^ | 4.82×10^-4^ | 5 | 72861782 | *UTP15* |
| cg12100742 | -1.00 | 3.43×10^-25^ | 4.36×10^-21^ | 5 | 75006001 | *POC5* |
| cg04277128 | -1.00 | 8.62×10^-15^ | 6.48×10^-11^ | 5 | 80537941 | *CKMT2* |
| cg23649326 | -1.00 | 7.57×10^-14^ | 5.52×10^-10^ | 5 | 80604119 | *ZCCHC9* |
| cg15297153 | -1.00 | 1.73×10^-5^ | 3.41×10^-2^ | 5 | 88185314 | *MEF2C* |
| cg06553959 | -1.00 | 1.31×10^-19^ | 1.22×10^-15^ | 5 | 94080833 | *MCTP1* |
| cg19775700 | -1.00 | 4.11×10^-9^ | 1.94×10^-5^ | 5 | 101940667 | *LINC00492* |
| cg18282812 | -1.00 | 1.06×10^-7^ | 3.84×10^-4^ | 5 | 114880667 | *FEM1C* |
| cg04294438 | -1.00 | 2.12×10^-13^ | 1.50×10^-9^ | 5 | 132106332 | *8-Sep* |
| cg23402356 | -1.00 | <5.00×10^-324^ | <5.00×10^-324^ | 5 | 133978822 |  |
| cg16876577 | -1.00 | 6.34×10^-89^ | 2.59×10^-84^ | 5 | 137460270 | *NME5* |
| cg26619047 | 1.00 | 4.16×10^-9^ | 1.95×10^-5^ | 5 | 139028068 | *CXXC5* |
| cg17258972 | -1.00 | 2.00×10^-22^ | 2.23×10^-18^ | 5 | 141249366 | *PCDH1* |
| cg20044189 | 1.00 | 2.90×10^-32^ | 4.65×10^-28^ | 5 | 141704548 | *SPRY4* |
| cg24074149 | -1.00 | 3.02×10^-6^ | 7.48×10^-3^ | 5 | 145145263 | *PRELID2* |
| cg06431905 | 1.00 | 2.31×10^-5^ | 4.35×10^-2^ | 5 | 148931119 | *CSNK1A1* |
| cg19430878 | -1.00 | 1.48×10^-7^ | 5.14×10^-4^ | 5 | 152780823 |  |
| cg02352687 | -1.00 | 1.29×10^-5^ | 2.63×10^-2^ | 5 | 172199101 | *DUSP1* |
| cg10832470 | -1.00 | 1.93×10^-5^ | 3.72×10^-2^ | 5 | 179105290 |  |
| cg01096398 | -1.00 | 4.60×10^-6^ | 1.10×10^-2^ | 6 | 1312475 | *FOXQ1* |
| cg22231012 | -1.00 | 1.93×10^-6^ | 5.11×10^-3^ | 6 | 2990206 | *DKFZP686I15217* |
| cg17740406 | -1.00 | 1.98×10^-6^ | 5.20×10^-3^ | 6 | 5261091 | *LYRM4* |
| cg12047860 | -1.00 | 1.36×10^-7^ | 4.81×10^-4^ | 6 | 14122063 | *CD83* |
| cg20183705 | 1.00 | 9.75×10^-7^ | 2.75×10^-3^ | 6 | 16439567 | *ATXN1* |
| cg11992375 | 1.00 | 1.17×10^-8^ | 5.28×10^-5^ | 6 | 27840957 | *HIST1H4L* |
| cg13207333 | -1.00 | 5.50×10^-9^ | 2.54×10^-5^ | 6 | 29974798 | *HLA-J* |
| cg26722322 | -1.00 | 5.27×10^-6^ | 1.22×10^-2^ | 6 | 30585676 | *MRPS18B* |
| cg09470958 | -1.00 | 2.49×10^-5^ | 4.61×10^-2^ | 6 | 31055471 |  |
| cg23715728 | -1.00 | 1.40×10^-7^ | 4.93×10^-4^ | 6 | 31379258 | *MICA* |
| cg25260255 | 1.00 | <5.00×10^-324^ | <5.00×10^-324^ | 6 | 31670897 | *BAT5* |
| cg11299207 | 1.00 | 6.56×10^-6^ | 1.47×10^-2^ | 6 | 31865794 | *EHMT2* |
| cg14361162 | -1.00 | 1.28×10^-5^ | 2.62×10^-2^ | 6 | 32935738 | *BRD2* |
| cg15748122 | -1.00 | 2.22×10^-9^ | 1.08×10^-5^ | 6 | 33195307 |  |
| cg18370200 | -1.00 | 1.98×10^-6^ | 5.20×10^-3^ | 6 | 33267248 | *RGL2* |
| cg27495579 | 1.00 | 6.60×10^-6^ | 1.48×10^-2^ | 6 | 36646483 | *CDKN1A* |
| cg04176813 | -1.00 | 1.75×10^-6^ | 4.67×10^-3^ | 6 | 37017355 |  |
| cg00778079 | -1.00 | 3.41×10^-6^ | 8.41×10^-3^ | 6 | 65683232 | *EYS* |
| cg18654873 | -1.00 | 1.62×10^-5^ | 3.22×10^-2^ | 6 | 91296666 | *MAP3K7* |
| cg23046921 | -1.00 | 1.31×10^-9^ | 6.52×10^-6^ | 6 | 108065649 | *SCML4* |
| cg14225532 | 1.00 | 7.62×10^-6^ | 1.66×10^-2^ | 6 | 111912751 | *TRAF3IP2-AS1* |
| cg11070989 | 1.00 | 3.46×10^-8^ | 1.39×10^-4^ | 6 | 113768503 |  |
| cg25773730 | -1.00 | 7.18×10^-19^ | 6.45×10^-15^ | 6 | 122279199 |  |
| cg01317979 | -1.00 | 5.30×10^-6^ | 1.22×10^-2^ | 6 | 147226879 | *STXBP5-AS1* |
| cg07885833 | -1.00 | 5.08×10^-41^ | 9.36×10^-37^ | 6 | 158388466 |  |
| cg07702098 | 1.00 | 7.98×10^-7^ | 2.31×10^-3^ | 7 | 1544360 | *INTS1* |
| cg19071989 | -1.00 | 1.97×10^-10^ | 1.08×10^-6^ | 7 | 2566113 | *LFNG* |
| cg00761242 | -1.00 | 1.89×10^-58^ | 4.81×10^-54^ | 7 | 5254009 | *WIPI2* |
| cg06003197 | 1.00 | 9.07×10^-22^ | 9.83×10^-18^ | 7 | 5569392 | *ACTB* |
| cg18542177 | -1.00 | 7.50×10^-6^ | 1.64×10^-2^ | 7 | 12726431 | *ARL4A* |
| cg24356769 | -1.00 | 2.45×10^-20^ | 2.44×10^-16^ | 7 | 23936971 |  |
| cg20554228 | 1.00 | 4.49×10^-6^ | 1.08×10^-2^ | 7 | 30029238 | *SCRN1* |
| cg14684441 | -1.00 | 6.65×10^-7^ | 1.97×10^-3^ | 7 | 40174653 | *C7orf10* |
| cg04350646 | -1.00 | 9.51×10^-6^ | 2.02×10^-2^ | 7 | 42972170 | *MRPL32* |
| cg21789367 | -1.00 | 2.00×10^-5^ | 3.81×10^-2^ | 7 | 45038325 | *CCM2* |
| cg06143328 | -1.00 | 5.41×10^-9^ | 2.51×10^-5^ | 7 | 73667763 | *RFC2* |
| cg19508409 | -1.00 | 1.84×10^-46^ | 3.99×10^-42^ | 7 | 73959946 | *GTF2IRD1* |
| cg12730381 | 1.00 | 2.23×10^-8^ | 9.27×10^-5^ | 7 | 75988212 | *YWHAG* |
| cg25898281 | -1.00 | 5.59×10^-8^ | 2.13×10^-4^ | 7 | 88964363 | *ZNF804B* |
| cg03429034 | 1.00 | 1.55×10^-7^ | 5.32×10^-4^ | 7 | 99613105 | *ZKSCAN1* |
| cg16089483 | -1.00 | 6.76×10^-6^ | 1.51×10^-2^ | 7 | 100873366 |  |
| cg04528378 | -1.00 | 2.61×10^-5^ | 4.80×10^-2^ | 7 | 106373036 |  |
| cg01910910 | -1.00 | 1.07×10^-8^ | 4.88×10^-5^ | 7 | 122844266 |  |
| cg00307737 | -1.00 | 1.82×10^-13^ | 1.32×10^-9^ | 7 | 124402490 | *GPR37* |
| cg10986355 | -1.00 | 1.68×10^-6^ | 4.50×10^-3^ | 7 | 126893362 | *GRM8* |
| cg19678561 | 1.00 | 5.38×10^-6^ | 1.24×10^-2^ | 7 | 129252859 | *NRF1* |
| cg13559478 | 1.00 | 3.86×10^-8^ | 1.54×10^-4^ | 7 | 148959298 | *ZNF783* |
| cg05797685 | -1.00 | 8.95×10^-6^ | 1.91×10^-2^ | 7 | 150130694 | *LINC00996* |
| cg04023439 | -1.00 | 9.24×10^-35^ | 1.62×10^-30^ | 7 | 154690004 |  |
| cg06978661 | -1.00 | 1.68×10^-8^ | 7.22×10^-5^ | 7 | 158808509 | *LINC00689* |
| cg11856918 | -1.00 | 4.70×10^-7^ | 1.46×10^-3^ | 8 | 20054956 | *ATP6V1B2* |
| cg08059714 | -1.00 | 5.71×10^-10^ | 3.03×10^-6^ | 8 | 21544248 |  |
| cg24924221 | -1.00 | 1.13×10^-5^ | 2.35×10^-2^ | 8 | 22856702 | *RHOBTB2* |
| cg05439756 | -1.00 | 1.29×10^-5^ | 2.63×10^-2^ | 8 | 26484766 | *DPYSL2* |
| cg15695962 | -1.00 | 1.80×10^-5^ | 3.51×10^-2^ | 8 | 37620478 | *PROSC* |
| cg02639993 | -1.00 | 6.27×10^-7^ | 1.87×10^-3^ | 8 | 38324024 | *FGFR1* |
| cg21798926 | 1.00 | 4.89×10^-8^ | 1.89×10^-4^ | 8 | 61429570 | *RAB2A* |
| cg22438847 | -1.00 | 2.06×10^-5^ | 3.93×10^-2^ | 8 | 61631641 | *CHD7* |
| cg07118376 | -1.00 | 2.28×10^-6^ | 5.90×10^-3^ | 8 | 62624872 | *ASPH* |
| cg24857609 | -1.00 | 1.01×10^-5^ | 2.12×10^-2^ | 8 | 71315397 | *NCOA2* |
| cg18635968 | 1.00 | 1.39×10^-11^ | 8.55×10^-8^ | 8 | 101225181 | *SPAG1* |
| cg13687825 | 1.00 | 1.46×10^-5^ | 2.94×10^-2^ | 8 | 101225344 | *SPAG1* |
| cg11478320 | 1.00 | 4.92×10^-64^ | 1.45×10^-59^ | 8 | 114446368 | *CSMD3* |
| cg13758290 | -1.00 | 8.34×10^-82^ | 3.07×10^-77^ | 8 | 123833524 | *ZHX2* |
| cg26472183 | -1.00 | 1.20×10^-9^ | 6.05×10^-6^ | 8 | 126445744 | *TRIB1* |
| cg26054541 | -1.00 | 1.69×10^-9^ | 8.32×10^-6^ | 8 | 129983632 |  |
| cg26959306 | -1.00 | 1.55×10^-6^ | 4.23×10^-3^ | 8 | 135792474 |  |
| cg13116158 | -1.00 | 9.92×10^-6^ | 2.10×10^-2^ | 8 | 143111655 |  |
| cg23351479 | 1.00 | 7.00×10^-8^ | 2.62×10^-4^ | 8 | 145043296 | *PLEC1* |
| cg03513643 | -1.00 | 3.35×10^-7^ | 1.09×10^-3^ | 8 | 145536188 | *HSF1* |
| cg11177152 | -1.00 | 2.61×10^-5^ | 4.80×10^-2^ | 9 | 536951 | *KANK1* |
| cg04217218 | -1.00 | 3.94×10^-25^ | 4.92×10^-21^ | 9 | 14693888 | *ZDHHC21* |
| cg16059473 | -1.00 | 6.17×10^-6^ | 1.40×10^-2^ | 9 | 29831191 |  |
| cg14082582 | -1.00 | 2.60×10^-14^ | 1.92×10^-10^ | 9 | 33602735 |  |
| cg13935102 | -1.00 | 1.28×10^-32^ | 2.15×10^-28^ | 9 | 35816956 |  |
| cg10142656 | -1.00 | 1.56×10^-8^ | 6.79×10^-5^ | 9 | 37753047 | *TRMT10B* |
| cg23709771 | -1.00 | 7.44×10^-18^ | 6.30×10^-14^ | 9 | 89452483 |  |
| cg18928442 | -1.00 | 5.22×10^-20^ | 5.06×10^-16^ | 9 | 114742754 |  |
| cg03877745 | 1.00 | 6.27×10^-8^ | 2.38×10^-4^ | 9 | 132369966 | *NTMT1* |
| cg19069968 | -1.00 | 2.18×10^-21^ | 2.26×10^-17^ | 9 | 134146766 | *FAM78A* |
| cg14463995 | 1.00 | 8.93×10^-6^ | 1.91×10^-2^ | 9 | 139048330 |  |
| cg14512731 | 1.00 | 2.06×10^-8^ | 8.72×10^-5^ | 9 | 139378529 | *C9orf163* |
| cg25397718 | 1.00 | 5.73×10^-17^ | 4.54×10^-13^ | 9 | 140317786 | *EXD3* |
| cg10212457 | 1.00 | 2.23×10^-5^ | 4.21×10^-2^ | 10 | 1142144 | *WDR37* |
| cg13333233 | -1.00 | 2.01×10^-7^ | 6.73×10^-4^ | 10 | 10380913 |  |
| cg08259506 | -1.00 | 1.35×10^-8^ | 5.97×10^-5^ | 10 | 11937255 |  |
| cg15647533 | -1.00 | 6.56×10^-6^ | 1.47×10^-2^ | 10 | 49809225 | *ARHGAP22* |
| cg01315067 | -1.00 | 2.47×10^-27^ | 3.44×10^-23^ | 10 | 51576232 | *NCOA4* |
| cg22388396 | -1.00 | 3.52×10^-7^ | 1.13×10^-3^ | 10 | 57326850 |  |
| cg13170704 | -1.00 | 5.86×10^-7^ | 1.78×10^-3^ | 10 | 59999428 | *IPMK* |
| cg26416615 | -1.00 | 4.91×10^-7^ | 1.51×10^-3^ | 10 | 63751843 | *ARID5B* |
| cg26845430 | -1.00 | 3.76×10^-6^ | 9.16×10^-3^ | 10 | 72384603 |  |
| cg05097887 | 1.00 | 1.03×10^-5^ | 2.17×10^-2^ | 10 | 75184282 | *ZMYND17* |
| cg23123731 | -1.00 | 3.89×10^-18^ | 3.33×10^-14^ | 10 | 80161611 |  |
| cg12337840 | -1.00 | 1.22×10^-59^ | 3.20×10^-55^ | 10 | 104220465 | *TMEM180* |
| cg24463290 | -1.00 | 3.55×10^-20^ | 3.48×10^-16^ | 10 | 114922641 | *TCF7L2* |
| cg09659072 | -1.00 | 3.20×10^-7^ | 1.05×10^-3^ | 10 | 114939288 |  |
| cg00981832 | -1.00 | 1.26×10^-10^ | 7.15×10^-7^ | 10 | 118719993 | *SHTN1* |
| cg14056583 | -1.00 | 4.68×10^-8^ | 1.81×10^-4^ | 10 | 127505359 | *UROS* |
| cg24492945 | -1.00 | 1.42×10^-10^ | 8.00×10^-7^ | 10 | 133648247 |  |
| cg08276752 | -1.00 | 1.98×10^-5^ | 3.80×10^-2^ | 11 | 7951221 | *OR10A6* |
| cg03741931 | -1.00 | 1.74×10^-7^ | 5.90×10^-4^ | 11 | 8204883 |  |
| cg23722437 | -1.00 | 1.17×10^-5^ | 2.43×10^-2^ | 11 | 13983009 | *SPON1* |
| cg08309747 | -1.00 | 1.64×10^-5^ | 3.25×10^-2^ | 11 | 13983705 | *SPON1* |
| cg26255604 | -1.00 | 1.02×10^-5^ | 2.14×10^-2^ | 11 | 13983719 | *SPON1* |
| cg25486824 | -1.00 | 3.63×10^-6^ | 8.89×10^-3^ | 11 | 13983790 | *SPON1* |
| cg17848362 | -1.00 | <5.00×10^-324^ | <5.00×10^-324^ | 11 | 46662488 | *ATG13* |
| cg03342113 | -1.00 | 5.70×10^-7^ | 1.74×10^-3^ | 11 | 61197477 | *CPSF7* |
| cg07800325 | 1.00 | 1.91×10^-5^ | 3.69×10^-2^ | 11 | 61722937 | *BEST1* |
| cg23934909 | 1.00 | 2.43×10^-5^ | 4.53×10^-2^ | 11 | 64884499 | *ZNHIT2* |
| cg16209630 | -1.00 | 9.61×10^-7^ | 2.72×10^-3^ | 11 | 64885070 | *ZNHIT2* |
| cg09005399 | -1.00 | 3.32×10^-8^ | 1.34×10^-4^ | 11 | 65809995 | *GAL3ST3* |
| cg02933962 | -1.00 | 9.28×10^-8^ | 3.42×10^-4^ | 11 | 66827615 | *RHOD* |
| cg20655434 | -1.00 | 2.61×10^-6^ | 6.60×10^-3^ | 11 | 67159118 | *LOC100130987* |
| cg14814999 | 1.00 | 4.03×10^-8^ | 1.59×10^-4^ | 11 | 67982011 | *SUV420H1* |
| cg04838454 | -1.00 | 4.40×10^-8^ | 1.72×10^-4^ | 11 | 75273344 | *SERPINH1* |
| cg01924993 | -1.00 | 1.78×10^-6^ | 4.74×10^-3^ | 11 | 118966382 | *H2AFX* |
| cg16633142 | -1.00 | 2.81×10^-27^ | 3.84×10^-23^ | 11 | 124272612 |  |
| cg24859617 | -1.00 | 4.87×10^-51^ | 1.16×10^-46^ | 11 | 125656966 | *PATE3* |
| cg23985147 | -1.00 | 7.39×10^-6^ | 1.63×10^-2^ | 11 | 126081641 | *RPUSD4* |
| cg21638242 | -1.00 | <5.00×10^-324^ | <5.00×10^-324^ | 11 | 134257617 | *B3GAT1* |
| cg00850947 | -1.00 | 8.19×10^-7^ | 2.36×10^-3^ | 11 | 134781395 |  |
| cg05125521 | -1.00 | 3.59×10^-135^ | 2.40×10^-130^ | 12 | 6391359 |  |
| cg15258033 | 1.00 | 2.65×10^-5^ | 4.85×10^-2^ | 12 | 10826753 | *STYK1* |
| cg08577441 | -1.00 | 5.79×10^-17^ | 4.54×10^-13^ | 12 | 12507213 |  |
| cg09444226 | -1.00 | 2.50×10^-6^ | 6.38×10^-3^ | 12 | 12887740 | *APOLD1* |
| cg04383469 | -1.00 | 2.00×10^-5^ | 3.81×10^-2^ | 12 | 41581775 | *PDZRN4* |
| cg02383245 | 1.00 | 8.54×10^-7^ | 2.44×10^-3^ | 12 | 41671164 | *PDZRN4* |
| cg01978144 | -1.00 | 2.95×10^-8^ | 1.20×10^-4^ | 12 | 42720088 | *PPHLN1* |
| cg21939233 | -1.00 | 1.57×10^-5^ | 3.14×10^-2^ | 12 | 43316251 |  |
| cg01025282 | -1.00 | 3.38×10^-11^ | 2.01×10^-7^ | 12 | 44170173 | *IRAK4* |
| cg05940536 | -1.00 | 1.32×10^-7^ | 4.68×10^-4^ | 12 | 52344971 | *ACVR1B* |
| cg09889291 | -1.00 | 5.45×10^-8^ | 2.09×10^-4^ | 12 | 52400650 | *GRASP* |
| cg15415743 | -1.00 | 1.31×10^-5^ | 2.66×10^-2^ | 12 | 53694130 | *C12orf10* |
| cg02002664 | -1.00 | 7.27×10^-8^ | 2.70×10^-4^ | 12 | 56325736 | *DGKA* |
| cg22507723 | -1.00 | 5.46×10^-6^ | 1.25×10^-2^ | 12 | 58131768 | *AGAP2* |
| cg07007807 | -1.00 | 1.82×10^-7^ | 6.12×10^-4^ | 12 | 117176115 | *RNFT2* |
| cg16955074 | -1.00 | 1.26×10^-47^ | 2.90×10^-43^ | 12 | 121671600 | *P2RX4* |
| cg11437331 | -1.00 | 1.17×10^-8^ | 5.28×10^-5^ | 12 | 124893649 | *NCOR2* |
| cg09569958 | -1.00 | 8.58×10^-102^ | 4.22×10^-97^ | 12 | 125349215 | *SCARB1* |
| cg07052262 | -1.00 | 3.02×10^-6^ | 7.48×10^-3^ | 12 | 133398988 | *GOLGA3* |
| cg09102880 | -1.00 | 2.00×10^-8^ | 8.53×10^-5^ | 13 | 27834168 |  |
| cg20402552 | -1.00 | 2.24×10^-7^ | 7.46×10^-4^ | 13 | 28397359 |  |
| cg13782382 | -1.00 | 3.76×10^-7^ | 1.21×10^-3^ | 13 | 30425099 | *UBL3* |
| cg21682474 | -1.00 | 2.29×10^-6^ | 5.90×10^-3^ | 13 | 33112475 | *N4BP2L2* |
| cg23199907 | -1.00 | 3.00×10^-6^ | 7.48×10^-3^ | 13 | 33305966 | *PDS5B* |
| cg10288525 | 1.00 | 2.57×10^-6^ | 6.54×10^-3^ | 13 | 33455187 |  |
| cg15695106 | 1.00 | 9.96×10^-26^ | 1.31×10^-21^ | 13 | 60738207 | *DIAPH3* |
| cg17162510 | -1.00 | 7.45×10^-6^ | 1.64×10^-2^ | 13 | 80341447 |  |
| cg16311158 | 1.00 | 1.57×10^-6^ | 4.27×10^-3^ | 13 | 99853156 | *UBAC2* |
| cg05725090 | 1.00 | 1.25×10^-10^ | 7.15×10^-7^ | 13 | 113242514 | *TUBGCP3* |
| cg07402411 | -1.00 | 2.10×10^-44^ | 4.07×10^-40^ | 13 | 113543125 |  |
| cg26256771 | -1.00 | 2.69×10^-22^ | 2.96×10^-18^ | 13 | 113642784 | *MCF2L* |
| cg14531340 | -1.00 | 1.52×10^-7^ | 5.24×10^-4^ | 13 | 113694058 | *MCF2L* |
| cg18774180 | -1.00 | 4.32×10^-6^ | 1.05×10^-2^ | 14 | 48109953 | *MDGA2* |
| cg10241319 | 1.00 | 1.10×10_-21_ | 1.17×10^-17^ | 14 | 61747853 | *TMEM30B* |
| cg23254057 | -1.00 | 1.66×10^-8^ | 7.16×10^-5^ | 14 | 64503000 | *SYNE2* |
| cg22095965 | -1.00 | 1.12×10^-7^ | 4.04×10^-4^ | 14 | 69059601 | *RAD51B* |
| cg18806438 | -1.00 | 2.33×10^-6^ | 5.97×10^-3^ | 14 | 73925183 | *NUMB* |
| cg00707881 | -1.00 | 2.71×10^-5^ | 4.96×10^-2^ | 14 | 77571003 | *KIAA1737* |
| cg22882310 | 1.00 | 2.91×10^-6^ | 7.30×10^-3^ | 14 | 78721428 | *NRXN3* |
| cg01859621 | -1.00 | 1.77×10^-5^ | 3.47×10^-2^ | 14 | 88511714 | *LINC01146* |
| cg18284469 | -1.00 | 1.09×10^-19^ | 1.05×10^-15^ | 14 | 91332189 |  |
| cg22897193 | -1.00 | 5.00×10^-6^ | 1.17×10^-2^ | 14 | 94406618 | *ASB2* |
| cg09385936 | -1.00 | 7.32×10^-11^ | 4.31×10^-7^ | 14 | 100791876 | *C14orf68* |
| cg09251423 | -1.00 | 1.13×10^-29^ | 1.70×10^-25^ | 14 | 101706089 |  |
| cg18412984 | -1.00 | <5.00×10^-324^ | <5.00×10^-324^ | 14 | 102604722 | *WDR20* |
| cg22503024 | -1.00 | 1.24×10^-10^ | 7.13×10^-7^ | 15 | 25100974 | *SNRPN* |
| cg14598522 | -1.00 | 7.42×10^-7^ | 2.16×10^-3^ | 15 | 25508414 | *SNORD115-45* |
| cg07325092 | 1.00 | 1.45×10^-6^ | 3.99×10^-3^ | 15 | 34875825 | *GOLGA8B* |
| cg08032884 | -1.00 | 8.99×10^-25^ | 1.10×10^-20^ | 15 | 41024014 | *RAD51* |
| cg10081723 | -1.00 | 1.60×10^-6^ | 4.32×10^-3^ | 15 | 45003329 | *B2M* |
| cg22300252 | -1.00 | 9.36×10^-31^ | 1.44×10^-26^ | 15 | 47705256 | *SEMA6D* |
| cg04513125 | -1.00 | 1.69×10^-9^ | 8.32×10^-6^ | 15 | 57891240 | *GCOM1* |
| cg12013685 | -1.00 | 6.24×10^-7^ | 1.87×10^-3^ | 15 | 63243220 |  |
| cg08304887 | -1.00 | 6.86×10^-12^ | 4.25×10^-8^ | 15 | 63569613 | *APH1B* |
| cg06710306 | -1.00 | 1.10×10^-63^ | 3.10×10^-59^ | 15 | 65248015 | *ANKDD1A* |
| cg04024170 | -1.00 | 1.33×10^-5^ | 2.69×10^-2^ | 15 | 74220756 | *LOXL1-AS1* |
| cg21712625 | -1.00 | 2.34×10^-72^ | 7.18×10^-68^ | 15 | 75191843 |  |
| cg24226371 | -1.00 | 4.44×10^-7^ | 1.39×10^-3^ | 15 | 75945038 | *SNX33* |
| cg19905236 | -1.00 | 1.74×10^-5^ | 3.42×10^-2^ | 15 | 78069099 | *LINGO1* |
| cg19674349 | -1.00 | 8.17×10^-19^ | 7.25×10^-15^ | 15 | 85483350 | *SLC28A1* |
| cg14584565 | -1.00 | 1.23×10^-5^ | 2.52×10^-2^ | 15 | 90068496 | *LOC283761* |
| cg20622470 | 1.00 | 9.30×10^-11^ | 5.40×10^-7^ | 15 | 91445801 |  |
| cg24446134 | -1.00 | 2.16×10^-6^ | 5.63×10^-3^ | 16 | 604033 | *CAPN15* |
| cg02878544 | -1.00 | 5.56×10^-6^ | 1.27×10^-2^ | 16 | 2559709 |  |
| cg03664605 | -1.00 | 1.25×10^-6^ | 3.46×10^-3^ | 16 | 16170435 | *ABCC1* |
| cg22412612 | 1.00 | 1.79×10^-5^ | 3.48×10^-2^ | 16 | 25123200 | *LCMT1* |
| cg10783242 | 1.00 | 3.28×10^-17^ | 2.63×10^-13^ | 16 | 46918335 | *GPT2* |
| cg05054953 | -1.00 | 1.84×10^-11^ | 1.12×10^-7^ | 16 | 66611099 | *CMTM1* |
| cg06866208 | -1.00 | 2.27×10^-24^ | 2.74×10^-20^ | 16 | 67560245 |  |
| cg08520423 | -1.00 | 2.99×10^-10^ | 1.63×10^-6^ | 16 | 68013968 | *DPEP3* |
| cg23401756 | -1.00 | 8.57×10^-9^ | 3.92×10^-5^ | 16 | 68013981 | *DPEP3* |
| cg02893490 | -1.00 | 2.58×10^-8^ | 1.06×10^-4^ | 16 | 68014110 | *DPEP3* |
| cg11999562 | -1.00 | 7.32×10^-7^ | 2.14×10^-3^ | 16 | 68014680 | *DPEP3* |
| cg07804857 | -1.00 | 7.78×10^-18^ | 6.51×10^-14^ | 16 | 84208222 | *LRRC50* |
| cg05424527 | -1.00 | 1.66×10^-5^ | 3.30×10^-2^ | 16 | 84485599 | *ATP2C2* |
| cg03158314 | -1.00 | 5.90×10^-29^ | 8.52×10^-25^ | 16 | 84513159 | *KIAA1609* |
| cg08761469 | -1.00 | 6.18×10^-10^ | 3.25×10^-6^ | 16 | 87118888 |  |
| cg15105282 | -1.00 | 1.47×10^-34^ | 2.51×10^-30^ | 16 | 89663409 | *CPNE7* |
| cg21110915 | -1.00 | 1.18×10^-6^ | 3.30×10^-3^ | 17 | 8888894 |  |
| cg21089050 | -1.00 | 1.47×10^-63^ | 4.00×10^-59^ | 17 | 10048963 | *GAS7* |
| cg00977293 | -1.00 | 6.43×10^-317^ | 6.77×10^-312^ | 17 | 13994507 | *COX10* |
| cg04259400 | -1.00 | 1.11×10^-7^ | 4.01×10^-4^ | 17 | 21160021 |  |
| cg13236370 | -1.00 | 1.66×10^-8^ | 7.16×10^-5^ | 17 | 36497241 | *GPR179* |
| cg24113565 | -1.00 | 7.90×10^-18^ | 6.54×10^-14^ | 17 | 43512820 |  |
| cg22571038 | 1.00 | 3.09×10^-6^ | 7.64×10^-3^ | 17 | 48585470 | *MYCBPAP* |
| cg02744699 | -1.00 | 4.05×10^-7^ | 1.28×10^-3^ | 17 | 80197898 |  |
| cg12328272 | 1.00 | 5.06×10^-21^ | 5.18×10^-17^ | 17 | 80478152 | *FOXK2* |
| cg04517114 | -1.00 | 4.12×10^-8^ | 1.61×10^-4^ | 18 | 5297041 | *ZBTB14* |
| cg03273509 | 1.00 | 5.28×10^-6^ | 1.22×10^-2^ | 18 | 9913806 | *VAPA* |
| cg00121346 | 1.00 | 4.86×10^-6^ | 1.15×10^-2^ | 18 | 13070566 | *CEP192* |
| cg11000116 | -1.00 | 1.27×10^-9^ | 6.36×10^-6^ | 18 | 13070587 | *CEP192* |
| cg27062159 | 1.00 | 2.38×10^-15^ | 1.81×10^-11^ | 18 | 34408844 | *C18orf10* |
| cg17154092 | -1.00 | 1.18×10^-9^ | 5.97×10^-6^ | 18 | 77675457 | *PQLC1* |
| cg13080551 | -1.00 | 3.13×10^-13^ | 2.19×10^-9^ | 19 | 815528 | *LPPR3* |
| cg21575667 | -1.00 | 3.94×10^-129^ | 2.42×10^-124^ | 19 | 1621692 | *TCF3* |
| cg02370644 | -1.00 | 1.55×10^-5^ | 3.10×10^-2^ | 19 | 2339509 | *SPPL2B* |
| cg06647026 | 1.00 | 7.24×10^-6^ | 1.61×10^-2^ | 19 | 3964837 | *DAPK3* |
| cg17313494 | 1.00 | 7.43×10^-13^ | 4.98×10^-9^ | 19 | 8645769 | *ADAMTS10* |
| cg07843419 | -1.00 | 3.55×10^-8^ | 1.42×10^-4^ | 19 | 10176296 | *C3P1* |
| cg24939813 | 1.00 | 2.38×10^-5^ | 4.44×10^-2^ | 19 | 18942818 | *UPF1* |
| cg09403024 | -1.00 | 3.39×10^-7^ | 1.09×10^-3^ | 19 | 23390590 |  |
| cg21279648 | -1.00 | 9.44×10^-13^ | 6.15×10^-9^ | 19 | 35629758 | *FXYD1* |
| cg05663558 | -1.00 | 9.75×10^-16^ | 7.49×10^-12^ | 19 | 35782257 | *MAG* |
| cg10714492 | 1.00 | 2.44×10^-8^ | 1.01×10^-4^ | 19 | 51321395 |  |
| cg20115802 | 1.00 | 4.82×10^-10^ | 2.59×10^-6^ | 19 | 51321569 |  |
| cg05906502 | -1.00 | 9.28×10^-11^ | 5.40×10^-7^ | 19 | 57702167 | *ZNF264* |
| cg06855087 | -1.00 | 1.85×10^-10^ | 1.03×10^-6^ | 20 | 7703904 |  |
| cg22580346 | -1.00 | 2.17×10^-21^ | 2.26×10^-17^ | 20 | 19928826 | *RIN2* |
| cg03454559 | -1.00 | 2.63×10^-6^ | 6.63×10^-3^ | 20 | 35445015 | *SOGA1* |
| cg13774308 | -1.00 | 4.62×10^-7^ | 1.44×10^-3^ | 20 | 36322200 | *CTNNBL1* |
| cg04923604 | -1.00 | 2.61×10^-9^ | 1.25×10^-5^ | 20 | 50138879 | *NFATC2* |
| cg13628960 | 1.00 | 5.75×10^-23^ | 6.52×10^-19^ | 20 | 62258574 | *GMEB2* |
| cg24982848 | 1.00 | 3.03×10^-103^ | 1.60×10^-98^ | 21 | 33784903 | *EVA1C* |
| cg18094366 | -1.00 | 2.11×10^-8^ | 8.84×10^-5^ | 22 | 24237343 | *MIF* |
| cg08202043 | -1.00 | 8.30×10^-146^ | 6.79×10^-141^ | 22 | 26990180 |  |
| cg07972762 | -1.00 | 8.71×10^-6^ | 1.87×10^-2^ | 22 | 33196384 | *SYN3* |
| cg25333298 | -1.00 | 5.47×10^-6^ | 1.25×10^-2^ | 22 | 36851113 |  |
| cg23612438 | -1.00 | 3.13×10^-45^ | 6.41×10^-41^ | 22 | 42513119 | *NDUFA6-AS1* |
| cg12520986 | -1.00 | 7.39×10^-6^ | 1.63×10^-2^ | 22 | 49411355 |  |
| **Neutrophils** | | | | | | |
| cg09599979 | 0.06 | 4.03×10^-10^ | 4.64×10^-6^ | 1 | 6660000 | *KLHL21* |
| cg15619966 | -0.03 | 1.31×10^-9^ | 1.23×10^-5^ | 1 | 9883889 | *CLSTN1* |
| cg23803120 | -0.06 | 2.00×10^-7^ | 1.06×10^-3^ | 1 | 14477617 |  |
| cg25322382 | -0.13 | 2.20×10^-5^ | 4.18×10^-2^ | 1 | 15942364 |  |
| cg25850484 | -0.08 | 9.23×10^-12^ | 2.00×10^-7^ | 1 | 17559348 | *PADI1* |
| cg07973655 | -0.03 | 5.06×10^-7^ | 2.23×10^-3^ | 1 | 25566628 |  |
| cg04285956 | -0.01 | 8.96×10^-7^ | 3.49×10^-3^ | 1 | 25566753 |  |
| cg16930569 | -0.07 | 1.11×10^-10^ | 1.52×10^-6^ | 1 | 41476918 | *CTPS1* |
| cg04179151 | 0.07 | 3.19×10^-6^ | 9.79×10^-3^ | 1 | 54520118 | *TMEM59* |
| cg20793071 | 0.14 | 2.52×10^-7^ | 1.27×10^-3^ | 1 | 54520450 | *C1orf83* |
| cg19126690 | -0.07 | 3.68×10^-10^ | 4.30×10^-6^ | 1 | 61390978 |  |
| cg10881514 | -0.07 | 7.52×10^-14^ | 2.52×10^-9^ | 1 | 64239618 | *ROR1* |
| cg23042676 | 0.10 | 5.24×10^-8^ | 3.24×10^-4^ | 1 | 67855787 | *IL12RB2* |
| cg19729188 | -0.21 | 3.04×10^-8^ | 2.02×10^-4^ | 1 | 85725931 | *C1orf52* |
| cg13581506 | 0.11 | 4.28×10^-6^ | 1.23×10^-2^ | 1 | 99456900 | *LPPR5* |
| cg21859597 | 0.01 | 6.65×10^-7^ | 2.77×10^-3^ | 1 | 115323395 | *SIKE1* |
| cg18189893 | 0.03 | 2.16×10^-6^ | 7.08×10^-3^ | 1 | 150981356 | *FAM63A* |
| cg19742929 | -0.05 | 1.05×10^-5^ | 2.44×10^-2^ | 1 | 160318820 | *NCSTN* |
| cg21963643 | 0.06 | 4.31×10^-6^ | 1.23×10^-2^ | 1 | 181451911 | *CACNA1E* |
| cg16657077 | 0.08 | 3.99×10^-6^ | 1.17×10^-2^ | 1 | 204055307 | *SOX13* |
| cg07692949 | -0.04 | 1.14×10^-5^ | 2.59×10^-2^ | 1 | 204425773 | *PIK3C2B* |
| cg22889645 | -0.06 | 1.17×10^-9^ | 1.15×10^-5^ | 1 | 205326399 | *KLHDC8A* |
| cg20879765 | -0.06 | 2.05×10^-6^ | 6.76×10^-3^ | 1 | 220264793 | *RNU5F-1* |
| cg15122697 | -0.05 | 7.64×10^-6^ | 1.91×10^-2^ | 1 | 224401807 | *LOC101927143* |
| cg03231504 | -0.14 | 7.25×10^-14^ | 2.52×10^-9^ | 1 | 228677993 | *RNF187* |
| cg11157725 | -0.15 | 1.57×10^-5^ | 3.28×10^-2^ | 1 | 230827973 | *COG2* |
| cg05290450 | 0.09 | 8.83×10^-6^ | 2.13×10^-2^ | 2 | 3272915 | *TSSC1* |
| cg07173100 | 0.10 | 1.04×10^-15^ | 6.95×10^-11^ | 2 | 3606140 | *RNASEH1* |
| cg23469963 | 0.06 | 1.25×10^-5^ | 2.76×10^-2^ | 2 | 9453539 | *ASAP2* |
| cg03240747 | 0.10 | 3.46×10^-8^ | 2.21×10^-4^ | 2 | 9668004 | *ADAM17* |
| cg08343699 | -0.14 | 4.64×10^-10^ | 5.11×10^-6^ | 2 | 37474493 | *NDUFAF7* |
| cg08086731 | -0.09 | 8.45×10^-8^ | 4.86×10^-4^ | 2 | 62429249 | *B3GNT2* |
| cg18560720 | 0.05 | 2.39×10^-12^ | 6.53×10^-8^ | 2 | 96952519 | *SNRNP200* |
| cg01334522 | -0.06 | 1.40×10^-12^ | 3.95×10^-8^ | 2 | 105720678 | *UTAT33* |
| cg10155147 | 0.13 | 5.22×10^-17^ | 5.41×10^-12^ | 2 | 106500829 | *NCK2* |
| cg09160302 | -0.06 | 3.27×10^-8^ | 2.13×10^-4^ | 2 | 121269906 |  |
| cg18110444 | -0.10 | 7.29×10^-20^ | 1.07×10^-14^ | 2 | 136743460 | *DARS* |
| cg09903921 | -0.06 | 1.24×10^-6^ | 4.54×10^-3^ | 2 | 143930082 | *ARHGAP15* |
| cg09425616 | -0.06 | 1.76×10^-9^ | 1.60×10^-5^ | 2 | 144969733 | *GTDC1* |
| cg01093934 | -0.12 | 5.35×10^-10^ | 5.80×10^-6^ | 2 | 177465789 | *MIR1246* |
| cg00372801 | 0.04 | 1.18×10^-5^ | 2.63×10^-2^ | 2 | 191219050 | *INPP1* |
| cg16652648 | -0.03 | 1.90×10^-6^ | 6.41×10^-3^ | 2 | 197791396 | *PGAP1* |
| cg04148467 | -0.05 | 1.96×10^-6^ | 6.54×10^-3^ | 2 | 207022459 | *NDUFS1* |
| cg01236747 | 0.06 | 4.23×10^-8^ | 2.64×10^-4^ | 2 | 208815027 | *PLEKHM3* |
| cg14357719 | -0.11 | 1.23×10^-8^ | 9.00×10^-5^ | 2 | 236500777 | *AGAP1* |
| cg21195450 | -0.14 | 3.53×10^-6^ | 1.07×10^-2^ | 2 | 237897606 |  |
| cg19125791 | 0.15 | 1.39×10^-5^ | 2.99×10^-2^ | 2 | 240265915 | *HDAC4* |
| cg11491420 | 0.07 | 7.90×10^-6^ | 1.96×10^-2^ | 2 | 242543287 | *THAP4* |
| cg24016448 | -0.08 | 3.09×10^-12^ | 8.13×10^-8^ | 3 | 11607988 | *VGLL4* |
| cg03395885 | -0.07 | 8.42×10^-9^ | 6.46×10^-5^ | 3 | 39259352 |  |
| cg10893140 | -0.01 | 3.55×10^-6^ | 1.07×10^-2^ | 3 | 49158297 | *USP19* |
| cg25554596 | 0.06 | 3.92×10^-6^ | 1.16×10^-2^ | 3 | 49230019 | *C3orf84* |
| cg07285807 | -0.10 | 4.12×10^-10^ | 4.67×10^-6^ | 3 | 49235826 | *CCDC36* |
| cg25291064 | -0.06 | 7.38×10^-6^ | 1.86×10^-2^ | 3 | 64130592 | *PRICKLE2* |
| cg18131244 | -0.05 | 6.52×10^-6^ | 1.70×10^-2^ | 3 | 117808713 |  |
| cg02797353 | -0.06 | 1.03×10^-5^ | 2.39×10^-2^ | 3 | 119531385 | *NR1I2* |
| cg06648971 | -0.02 | 4.13×10^-7^ | 1.89×10^-3^ | 3 | 125239030 | *SNX4* |
| cg08958945 | 0.05 | 9.64×10^-6^ | 2.28×10^-2^ | 3 | 133591690 | *RAB6B* |
| cg08760818 | -0.08 | 5.24×10^-6^ | 1.43×10^-2^ | 3 | 167425609 | *PDCD10* |
| cg08192869 | -0.05 | 1.79×10^-11^ | 3.31×10^-7^ | 3 | 170068801 |  |
| cg14603321 | 0.09 | 2.33×10^-8^ | 1.57×10^-4^ | 3 | 171920662 | *FNDC3B* |
| cg04738673 | -0.12 | 9.42×10^-10^ | 9.78×10^-6^ | 3 | 177482968 |  |
| cg27301092 | -0.03 | 1.72×10^-5^ | 3.50×10^-2^ | 4 | 1352452 | *KIAA1530* |
| cg19002058 | 0.07 | 6.31×10^-7^ | 2.69×10^-3^ | 4 | 6347514 | *PPP2R2C* |
| cg01138944 | 0.07 | 9.45×10^-7^ | 3.65×10^-3^ | 4 | 7781082 | *AFAP1* |
| cg10262842 | -0.06 | 2.77×10^-10^ | 3.40×10^-6^ | 4 | 15233600 | *LOC101929095* |
| cg10604765 | -0.05 | 4.28×10^-6^ | 1.23×10^-2^ | 4 | 33882451 |  |
| cg13433561 | 0.05 | 6.81×10^-8^ | 4.11×10^-4^ | 4 | 42706688 |  |
| cg07243941 | -0.13 | 1.57×10^-9^ | 1.45×10^-5^ | 4 | 47840162 | *CORIN* |
| cg24461090 | -0.15 | 4.71×10-6 | 1.32×10^-2^ | 4 | 79847813 | *PAQR3* |
| cg07856552 | 0.19 | 3.30×10-24 | 6.09×10^-19^ | 4 | 90548906 |  |
| cg17318769 | 0.14 | 8.76×10^-16^ | 6.45×10^-11^ | 4 | 154229091 | *TRIM2* |
| cg11383226 | 0.13 | 1.90×10^-11^ | 3.42×10-7 | 4 | 159236616 |  |
| cg06695338 | -0.10 | 1.47×10^-5^ | 3.13×10-2 | 4 | 187518024 | *FAT1* |
| cg03930088 | -0.14 | 1.32×10^-31^ | 3.24×10^-26^ | 4 | 187647395 |  |
| cg16034787 | -0.14 | 2.97×10^-8^ | 1.99×10^-4^ | 5 | 508349 | *SLC9A3* |
| cg16886578 | 0.05 | 1.31×10^-5^ | 2.83×10^-2^ | 5 | 2093634 |  |
| cg18731024 | -0.05 | 3.99×10^-6^ | 1.17×10^-2^ | 5 | 2998264 |  |
| cg04480129 | 0.12 | 3.65×10^-9^ | 3.09×10^-5^ | 5 | 66316631 | *MAST4* |
| cg16320199 | 0.01 | 7.19×10^-8^ | 4.30×10^-4^ | 5 | 70883065 | *MCCC2* |
| cg18788891 | 0.02 | 2.21×10^-9^ | 1.96×10^-5^ | 5 | 70883072 | *MCCC2* |
| cg13238200 | -0.13 | 2.06×10^-7^ | 1.08×10^-3^ | 5 | 77335062 | *AP3B1* |
| cg17693122 | -0.07 | 2.67×10^-6^ | 8.45×10^-3^ | 5 | 83492287 | *EDIL3* |
| cg17860174 | -0.13 | 1.80×10^-5^ | 3.59×10^-2^ | 5 | 122180484 | *SNX24* |
| cg27658391 | -0.06 | 8.32×10^-6^ | 2.04×10^-2^ | 5 | 140023555 | *TMCO6* |
| cg04306564 | -0.12 | 6.75×10^-12^ | 1.60×10^-7^ | 5 | 146889771 | *DPYSL3* |
| cg01919823 | -0.06 | 1.89×10^-7^ | 1.01×10^-3^ | 5 | 148741481 | *PCYOX1L* |
| cg07682547 | -0.11 | 6.73×10^-18^ | 8.27×10^-13^ | 5 | 153863417 |  |
| cg04093159 | -0.01 | 1.42×10^-5^ | 3.03×10^-2^ | 5 | 166211825 |  |
| cg09146892 | -0.13 | 3.36×10^-11^ | 5.63×10^-7^ | 6 | 6971335 |  |
| cg20284834 | 0.03 | 7.13×10^-6^ | 1.82×10^-2^ | 6 | 15499615 | *JARID2* |
| cg06463685 | 0.14 | 1.83×10^-5^ | 3.60×10^-2^ | 6 | 18380900 |  |
| cg07191121 | -0.10 | 4.74×10^-6^ | 1.32×10^-2^ | 6 | 28724650 |  |
| cg18281744 | 0.06 | 1.14×10^-5^ | 2.59×10^-2^ | 6 | 29455512 | *MAS1L* |
| cg24531536 | -0.06 | 3.13×10^-6^ | 9.64×10^-3^ | 6 | 29520698 |  |
| cg00937691 | 0.08 | 1.99×10^-5^ | 3.87×10^-2^ | 6 | 29796105 | *HLA-G* |
| cg10750989 | 0.03 | 1.75×10^-6^ | 6.00×10^-3^ | 6 | 31371330 | *MICA* |
| cg13347198 | -0.11 | 1.21×10^-11^ | 2.54×10^-7^ | 6 | 32798198 | *TAP2* |
| cg19254793 | -0.10 | 1.19×10^-7^ | 6.70×10^-4^ | 6 | 44695348 |  |
| cg02791384 | -0.05 | 6.19×10^-6^ | 1.63×10^-2^ | 6 | 56408859 | *RNU6-71P* |
| cg01567563 | 0.06 | 2.86×10^-6^ | 8.87×10^-3^ | 6 | 90685843 | *BACH2* |
| cg17388996 | -0.05 | 4.81×10^-11^ | 7.71×10^-7^ | 6 | 108145374 | *SCML4* |
| cg15406010 | -0.11 | 1.31×10^-11^ | 2.67×10^-7^ | 6 | 108478650 |  |
| cg16966529 | 0.15 | 2.15×10^-11^ | 3.77×10^-7^ | 6 | 150004505 | *LATS1* |
| cg12341553 | -0.06 | 6.79×10^-6^ | 1.74×10^-2^ | 6 | 160692423 |  |
| cg02905206 | -0.06 | 9.28×10^-11^ | 1.34×10^-6^ | 6 | 168321999 | *MLLT4* |
| cg04985696 | 0.03 | 3.16×10^-8^ | 2.08×10^-4^ | 7 | 44622204 | *TMED4* |
| cg27528426 | 0.02 | 1.03×10^-9^ | 1.02×10^-5^ | 7 | 65540851 | *ASL* |
| cg06394442 | -0.05 | 1.01×10-6 | 3.87×10^-3^ | 7 | 73908364 | *GTF2IRD1* |
| cg18189615 | -0.10 | 8.84×10-10 | 9.30×10^-6^ | 7 | 75386749 |  |
| cg16639595 | 0.06 | 2.02×10^-9^ | 1.81×10^-5^ | 7 | 87856984 | *SRI* |
| cg07098123 | -0.11 | 8.60×10^-7^ | 3.41×10^-3^ | 7 | 91507250 | *MTERF* |
| cg14719076 | -0.15 | 5.38×10^-32^ | 1.98×10^-26^ | 7 | 96653747 | *DLX5* |
| cg26724642 | 0.11 | 1.38×10^-11^ | 2.75×10^-7^ | 7 | 97895513 |  |
| cg19900764 | 0.10 | 2.39×10^-6^ | 7.69×10^-3^ | 7 | 111560233 | *DOCK4* |
| cg13504856 | -0.10 | 5.00×10^-9^ | 4.05×10^-5^ | 7 | 128956859 | *AHCYL2* |
| cg00213189 | -0.08 | 1.89×10^-13^ | 6.05×10^-9^ | 7 | 143012820 | *CLCN1* |
| cg07499066 | -0.06 | 6.46×10^-12^ | 1.59×10^-7^ | 7 | 149171379 | *ZNF746* |
| cg19454081 | -0.11 | 4.68×10^-7^ | 2.10×10^-3^ | 7 | 152593563 |  |
| cg18941360 | 0.05 | 6.09×10^-6^ | 1.61×10^-2^ | 7 | 153112069 |  |
| cg20419410 | -0.02 | 1.55×10^-8^ | 1.07×10^-4^ | 7 | 155089803 | *INSIG1* |
| cg00827082 | -0.05 | 2.05×10^-5^ | 3.96×10^-2^ | 8 | 407482 | *FBXO25* |
| cg07058086 | -0.08 | 2.10×10^-5^ | 4.05×10^-2^ | 8 | 29120186 | *KIF13B* |
| cg27627674 | 0.06 | 2.66×10^-9^ | 2.33×10^-5^ | 8 | 33448880 | *DUSP26* |
| cg07370726 | 0.13 | 9.94×10^-7^ | 3.82×10^-3^ | 8 | 47980380 |  |
| cg03214200 | -0.10 | 7.23×10^-7^ | 2.96×10^-3^ | 8 | 65553669 | *CYP7B1* |
| cg17380533 | -0.05 | 1.12×10^-6^ | 4.21×10^-3^ | 8 | 72875102 | *MSC-AS1* |
| cg14317230 | -0.15 | 1.70×10^-5^ | 3.49×10^-2^ | 8 | 98708278 | *MTDH* |
| cg22516182 | -0.10 | 4.85×10^-6^ | 1.34×10^-2^ | 8 | 102400152 |  |
| cg25118752 | -0.06 | 2.68×10^-6^ | 8.45×10^-3^ | 8 | 110381021 | *PKHD1L1* |
| cg22594690 | 0.06 | 2.39×10^-7^ | 1.22×10^-3^ | 8 | 121718948 | *SNTB1* |
| cg06158237 | -0.11 | 8.07×10^-7^ | 3.23×10^-3^ | 8 | 131661720 |  |
| cg02526790 | 0.13 | 5.87×10^-17^ | 5.41×10^-12^ | 8 | 134044590 | *TG* |
| cg02390569 | -0.08 | 1.24×10^-6^ | 4.54×10^-3^ | 8 | 145624983 | *CPSF1* |
| cg25958241 | -0.17 | 3.33×10^-9^ | 2.85×10^-5^ | 9 | 35278307 | *UNC13B* |
| cg03081134 | 0.02 | 1.80×10^-5^ | 3.59×10^-2^ | 9 | 36036806 | *RECK* |
| cg13931845 | -0.06 | 7.41×10^-8^ | 4.37×10^-4^ | 9 | 89894865 |  |
| cg14220146 | 0.09 | 9.98×10^-8^ | 5.65×10^-4^ | 9 | 96068079 | *WNK2* |
| cg14583686 | 0.05 | 4.66×10^-9^ | 3.82×10^-5^ | 9 | 130226589 | *LRSAM1* |
| cg13521319 | -0.04 | 1.35×10^-6^ | 4.89×10^-3^ | 9 | 133423844 |  |
| cg19890156 | -0.10 | 3.66×10^-7^ | 1.73×10^-3^ | 9 | 139934859 | *NPDC1* |
| cg02523824 | -0.11 | 8.16×10^-10^ | 8.71×10^-6^ | 10 | 11298786 | *CUGBP2* |
| cg24405543 | -0.06 | 1.36×10^-8^ | 9.83×10^-5^ | 10 | 11872888 | *C10orf47* |
| cg15882987 | 0.06 | 1.23×10^-7^ | 6.76×10^-4^ | 10 | 16790575 | *RSU1* |
| cg01161217 | -0.05 | 1.51×10^-9^ | 1.41×10^-5^ | 10 | 26758768 | *APBB1IP* |
| cg24914935 | -0.07 | 1.66×10^-15^ | 1.02×10^-10^ | 10 | 30589494 |  |
| cg13556387 | -0.06 | 8.89×10^-9^ | 6.68×10^-5^ | 10 | 33224969 | *ITGB1* |
| cg17504814 | -0.05 | 6.58×10^-7^ | 2.77×10^-3^ | 10 | 35540771 | *CCNY* |
| cg06993191 | 0.04 | 2.06×10^-6^ | 6.76×10^-3^ | 10 | 76514031 |  |
| cg01536438 | 0.06 | 2.59×10^-7^ | 1.28×10^-3^ | 10 | 90712625 | *ACTA2* |
| cg06930332 | -0.12 | 7.63×10^-11^ | 1.15×10^-6^ | 10 | 102006954 | *CWF19L1* |
| cg16499947 | 0.06 | 2.65×10^-6^ | 8.40×10^-3^ | 10 | 123329113 | *FGFR2* |
| cg17997363 | -0.06 | 2.20×10^-5^ | 4.18×10^-2^ | 10 | 123353337 | *FGFR2* |
| cg14897471 | -0.04 | 1.79×10^-5^ | 3.59×10^-2^ | 10 | 127548239 | *DHX32* |
| cg09216081 | -0.08 | 6.65×10^-7^ | 2.77×10^-3^ | 10 | 134940484 | *GPR123* |
| cg27010328 | -0.11 | 7.18×10^-12^ | 1.65×10^-7^ | 11 | 238081 | *PSMD13* |
| cg18844120 | -0.06 | 6.02×10^-6^ | 1.60×10^-2^ | 11 | 14915223 | *CYP2R1* |
| cg21728100 | -0.08 | 2.69×10^-5^ | 4.90×10^-2^ | 11 | 57640698 |  |
| cg01559206 | -0.07 | 4.56×10^-6^ | 1.28×10^-2^ | 11 | 61070063 | *DDB1* |
| cg09098150 | -0.04 | 6.62×10^-6^ | 1.71×10^-2^ | 11 | 61216180 |  |
| cg13263274 | 0.06 | 8.93×10^-6^ | 2.14×10^-2^ | 11 | 63885584 | *FLRT1* |
| cg19815354 | -0.12 | 2.44×10^-5^ | 4.54×10^-2^ | 11 | 77450740 | *RSF1* |
| cg11916248 | 0.05 | 4.07×10^-6^ | 1.19×10^-2^ | 11 | 95214092 |  |
| cg00570685 | -0.06 | 7.15×10^-6^ | 1.82×10^-2^ | 11 | 107123373 |  |
| cg05962325 | 0.07 | 2.15×10^-5^ | 4.10×10^-2^ | 11 | 107992005 | *ACAT1* |
| cg26572438 | -0.14 | 1.48×10^-11^ | 2.80×10^-7^ | 11 | 118123982 | *MPZL3* |
| cg06038239 | -0.13 | 3.10×10^-15^ | 1.63×10^-10^ | 11 | 123612522 | *ZNF202* |
| cg27371539 | 0.08 | 9.85×10^-6^ | 2.31×10^-2^ | 11 | 124488923 | *PANX3* |
| cg19649564 | -0.14 | 1.00×10^-39^ | 7.40×10^-34^ | 11 | 124932892 | *SLC37A2* |
| cg10968396 | -0.14 | 1.83×10^-5^ | 3.60×10^-2^ | 12 | 1352926 | *ERC1* |
| cg03165826 | -0.06 | 6.33×10^-8^ | 3.86×10^-4^ | 12 | 15881211 | *EPS8* |
| cg21239961 | -0.05 | 1.41×10^-8^ | 1.01×10^-4^ | 12 | 20197870 | *LOC100506393* |
| cg10873203 | -0.05 | 5.95×10^-9^ | 4.66×10^-5^ | 12 | 25942772 |  |
| cg25598488 | 0.10 | 5.74×10^-9^ | 4.55×10^-5^ | 12 | 34258505 |  |
| cg06349909 | -0.08 | 1.68×10^-5^ | 3.47×10^-2^ | 12 | 51608175 | *POU6F1* |
| cg17129918 | -0.11 | 8.90×10^-12^ | 1.99×10^-7^ | 12 | 53098560 | *KRT77* |
| cg14519793 | 0.07 | 1.18×10^-9^ | 1.15×10^-5^ | 12 | 66696305 | *HELB* |
| cg05691053 | -0.06 | 1.45×10^-10^ | 1.94×10^-6^ | 12 | 72104344 |  |
| cg03916864 | 0.05 | 2.19×10^-7^ | 1.14×10^-3^ | 12 | 109535384 | *UNG* |
| cg09544079 | -0.07 | 2.24×10^-7^ | 1.16×10^-3^ | 12 | 110808689 |  |
| cg14079445 | 0.06 | 3.80×10^-7^ | 1.77×10^-3^ | 12 | 111493997 | *CUX2* |
| cg05696801 | 0.04 | 1.26×10^-5^ | 2.76×10^-2^ | 12 | 118573886 | *PEBP1* |
| cg08134342 | -0.03 | 1.27×10^-6^ | 4.63×10^-3^ | 12 | 122239035 | *LOC338799* |
| cg25348433 | -0.04 | 5.91×10^-6^ | 1.58×10^-2^ | 13 | 78519244 | *EDNRB* |
| cg24526477 | -0.07 | 4.01×10^-13^ | 1.23×10^-8^ | 13 | 95908531 | *ABCC4* |
| cg12285887 | -0.05 | 9.74×10^-6^ | 2.29×10^-2^ | 14 | 55588682 |  |
| cg13436449 | -0.06 | 1.48×10^-11^ | 2.80×10^-7^ | 14 | 59600377 |  |
| cg15989617 | -0.07 | 8.73×10^-6^ | 2.12×10^-2^ | 14 | 61574389 |  |
| cg19021355 | -0.05 | 6.67×10^-7^ | 2.77×10^-3^ | 14 | 74771221 | *ABCD4* |
| cg21140981 | -0.11 | 5.28×10^-7^ | 2.29×10^-3^ | 14 | 91766264 | *CCDC88C* |
| cg05794283 | -0.09 | 8.87×10^-7^ | 3.47×10^-3^ | 14 | 93686639 | *UBR7* |
| cg06265809 | 0.10 | 1.44×10^-5^ | 3.08×10^-2^ | 14 | 101525784 | *MIR496* |
| cg13078563 | -0.12 | 3.83×10^-8^ | 2.41×10^-4^ | 15 | 27016796 | *GABRB3* |
| cg26995364 | -0.10 | 8.85×10^-9^ | 6.68×10^-5^ | 15 | 29336425 | *APBA2* |
| cg07709681 | -0.03 | 1.48×10^-5^ | 3.13×10^-2^ | 15 | 34393712 | *PGBD4* |
| cg02765225 | -0.08 | 6.45×10^-6^ | 1.69×10^-2^ | 15 | 44083979 | *SERF2* |
| cg09289674 | -0.07 | 2.47×10^-10^ | 3.14×10^-6^ | 15 | 51699392 | *GLDN* |
| cg07947039 | 0.13 | 7.34×10^-13^ | 2.16×10^-8^ | 15 | 56292422 |  |
| cg09770632 | -0.05 | 1.01×10^-10^ | 1.43×10^-6^ | 15 | 56310025 |  |
| cg03841750 | 0.09 | 1.93×10^-5^ | 3.76×10^-2^ | 15 | 70244882 |  |
| cg09585074 | -0.13 | 3.88×10^-15^ | 1.90×10^-10^ | 15 | 72638395 | *HEXA* |
| cg18753337 | -0.12 | 4.75×10^-7^ | 2.12×10^-3^ | 15 | 80206998 | *ST20* |
| cg19715141 | -0.05 | 2.46×10^-5^ | 4.56×10^-2^ | 15 | 86363646 |  |
| cg11670074 | -0.09 | 1.81×10^-5^ | 3.60×10^-2^ | 15 | 93635693 |  |
| cg00078759 | -0.05 | 6.35×10^-11^ | 9.96×10^-7^ | 16 | 1600969 | *IFT140* |
| cg16784970 | 0.06 | 1.08×10^-10^ | 1.50×10^-6^ | 16 | 2123215 | *TSC2* |
| cg14939446 | -0.04 | 9.79×10^-8^ | 5.59×10^-4^ | 16 | 2571015 | *AMDHD2* |
| cg00454447 | -0.07 | 5.60×10^-9^ | 4.49×10^-5^ | 16 | 3728185 | *TRAP1* |
| cg26570867 | -0.07 | 4.90×10^-14^ | 2.00×10^-9^ | 16 | 12308181 | *SNX29* |
| cg11586377 | -0.08 | 3.70×10^-11^ | 6.06×10^-7^ | 16 | 22263785 | *EEF2K* |
| cg19415738 | 0.07 | 3.56×10^-10^ | 4.23×10^-6^ | 16 | 30418482 | *ZNF771* |
| cg00131272 | 0.04 | 3.40×10^-7^ | 1.62×10^-3^ | 16 | 46957465 | *GPT2* |
| cg13944965 | -0.06 | 1.13×10^-8^ | 8.43×10^-5^ | 16 | 66889028 |  |
| cg19141655 | 0.06 | 1.99×10^-5^ | 3.86×10^-2^ | 16 | 70456139 | *ST3GAL2* |
| cg16777782 | -0.04 | 3.85×10^-6^ | 1.15×10^-2^ | 16 | 82671333 | *CDH13* |
| cg01466631 | -0.04 | 3.23×10^-7^ | 1.56×10^-3^ | 17 | 1094369 |  |
| cg10735834 | -0.34 | 1.10×10^-5^ | 2.52×10^-2^ | 17 | 6618119 | *SLC13A5* |
| cg03477621 | -0.09 | 7.01×10^-7^ | 2.89×10^-3^ | 17 | 9472738 | *STX8* |
| cg06511117 | -0.06 | 1.19×10^-8^ | 8.75×10^-5^ | 17 | 19216903 | *EPN2* |
| cg10228608 | -0.06 | 2.60×10^-10^ | 3.24×10^-6^ | 17 | 19804476 |  |
| cg17235374 | -0.11 | 6.83×10^-11^ | 1.05×10^-6^ | 17 | 27717757 | *TAOK1* |
| cg13227200 | -0.04 | 4.56×10^-6^ | 1.28×10^-2^ | 17 | 30687348 | *ZNF207* |
| cg21336116 | -0.09 | 5.67×10^-7^ | 2.43×10^-3^ | 17 | 39093658 | *KRT23* |
| cg05031081 | -0.08 | 1.79×10^-10^ | 2.35×10^-6^ | 17 | 72239436 | *TTYH2* |
| cg07027472 | -0.05 | 4.57×10^-9^ | 3.78×10^-5^ | 17 | 73391422 | *GRB2* |
| cg04546917 | -0.05 | 5.31×10^-7^ | 2.29×10^-3^ | 17 | 73667384 | *SAP30BP* |
| cg19169246 | -0.03 | 2.67×10^-5^ | 4.87×10^-2^ | 17 | 73887208 | *TRIM65* |
| cg13039908 | -0.06 | 3.88×10^-6^ | 1.15×10^-2^ | 17 | 75170663 | *SEC14L1* |
| cg15275493 | -0.06 | 1.54×10^-5^ | 3.25×10^-2^ | 17 | 75310725 | *9-Sep* |
| cg24138691 | -0.02 | 1.03×10^-9^ | 1.02×10^-5^ | 17 | 79251354 | *SLC38A10* |
| cg16681349 | -0.06 | 4.66×10^-7^ | 2.10×10^-3^ | 17 | 80614369 |  |
| cg18089670 | -0.14 | 2.05×10^-14^ | 8.88×10^-10^ | 18 | 660756 | *TYMS* |
| cg12306367 | -0.04 | 4.36×10^-6^ | 1.24×10^-2^ | 18 | 19777305 | *GATA6* |
| cg20218161 | -0.06 | 7.27×10^-7^ | 2.96×10^-3^ | 18 | 61836736 | *LOC284294* |
| cg06294408 | -0.06 | 1.70×10^-6^ | 5.90×10^-3^ | 18 | 71749347 | *FBXO15* |
| cg25185915 | -0.05 | 1.63×10^-8^ | 1.12×10^-4^ | 18 | 74507281 | *LOC100131655* |
| cg17102627 | -0.09 | 4.22×10^-10^ | 4.71×10^-6^ | 18 | 77230598 | *NFATC1* |
| cg14700108 | 0.08 | 1.18×10^-5^ | 2.63×10^-2^ | 19 | 2637722 | *GNG7* |
| cg22933199 | 0.09 | 9.99×10^-10^ | 1.02×10^-5^ | 19 | 12163909 | *ZNF878* |
| cg01636019 | -0.07 | 1.91×10^-6^ | 6.41×10^-3^ | 19 | 12691334 | *ZNF490* |
| cg20276511 | 0.01 | 1.62×10^-6^ | 5.68×10^-3^ | 19 | 15543527 | *WIZ* |
| cg23397718 | 0.13 | 1.44×10^-8^ | 1.02×10^-4^ | 19 | 18630997 | *ELL* |
| cg10105623 | 0.04 | 3.44×10^-8^ | 2.21×10^-4^ | 19 | 34893958 | *PDCD2L* |
| cg03422185 | -0.12 | 4.36×10^-15^ | 2.01×10^-10^ | 19 | 41921278 | *BCKDHA* |
| cg04775950 | 0.07 | 1.72×10^-6^ | 5.93×10^-3^ | 19 | 44115372 | *ZNF428* |
| cg13566596 | 0.09 | 2.37×10^-6^ | 7.65×10^-3^ | 19 | 45033572 | *CEACAM20* |
| cg01009059 | -0.05 | 1.31×10^-9^ | 1.23×10^-5^ | 19 | 46391024 |  |
| cg25028162 | -0.06 | 1.96×10^-8^ | 1.34×10^-4^ | 19 | 52150233 | *SIGLEC14* |
| cg05619639 | -0.10 | 5.31×10^-6^ | 1.44×10^-2^ | 19 | 53269486 | *ZNF600* |
| cg05927427 | -0.06 | 2.45×10^-11^ | 4.20×10^-7^ | 20 | 25187813 | *ENTPD6* |
| cg00514271 | 0.06 | 3.85×10^-7^ | 1.77×10^-3^ | 20 | 30593353 |  |
| cg15118314 | -0.07 | 1.51×10^-6^ | 5.38×10^-3^ | 20 | 34019699 |  |
| cg09921555 | -0.06 | 1.02×10^-5^ | 2.37×10^-2^ | 20 | 43590026 | *TOMM34* |
| cg03431585 | -0.12 | 1.69×10^-6^ | 5.87×10^-3^ | 20 | 44995633 | *ELMO2* |
| cg05494607 | 0.08 | 4.45×10^-16^ | 3.64×10^-11^ | 20 | 50416707 | *SALL4* |
| cg12750641 | -0.04 | 1.71×10^-5^ | 3.50×10^-2^ | 20 | 50772956 | *ZFP64* |
| cg17734137 | 0.05 | 2.44×10^-10^ | 3.14×10^-6^ | 20 | 58647209 | *C20orf197* |
| cg24444091 | -0.08 | 1.18×10^-5^ | 2.63×10^-2^ | 21 | 27462541 | *APP* |
| cg13095416 | 0.01 | 2.42×10^-5^ | 4.52×10^-2^ | 21 | 33941714 |  |
| cg23609630 | -0.07 | 3.30×10^-7^ | 1.59×10^-3^ | 21 | 35126912 | *ITSN1* |
| cg02330424 | -0.22 | 3.45×10^-10^ | 4.16×10^-6^ | 22 | 21135726 | *SERPIND1* |
| cg23185207 | 0.06 | 4.89×10^-12^ | 1.24×10^-7^ | 22 | 27705236 |  |
| cg17758280 | 0.07 | 6.24×10^-14^ | 2.30×10^-9^ | 22 | 29702766 | *GAS2L1* |
| cg04696109 | -0.05 | 9.07×10-11 | 1.34×10^-6^ | 22 | 36081855 |  |
| cg19972814 | -0.05 | 2.26×10-6 | 7.34×10^-3^ | 22 | 38059813 | *PDXP* |
| cg22632063 | -0.06 | 3.09×10-15 | 1.63×10^-10^ | 22 | 38696180 | *CSNK1E* |
| cg26679753 | -0.07 | 2.74×10^-9^ | 2.37×10^-5^ | 22 | 46747353 | *TRMU* |
| **NK cells** |  |  |  |  |  |  |
| cg20737459 | -0.25 | 7.13×10^-12^ | 6.74×10^-8^ | 1 | 6523514 | *TNFRSF25* |
| cg16591948 | -0.12 | 9.82×10^-6^ | 2.39×10^-2^ | 1 | 8716348 | *RERE* |
| cg17223279 | -0.40 | 2.57×10^-9^ | 1.81×10^-5^ | 1 | 11202866 | *MTOR-AS1* |
| cg16081846 | -0.25 | 2.70×10^-8^ | 1.49×10^-4^ | 1 | 17418725 | *PADI2* |
| cg15642494 | -0.27 | 5.08×10^-15^ | 7.80×10^-11^ | 1 | 19616829 |  |
| cg24399432 | 0.12 | 1.48×10^-6^ | 4.70×10^-3^ | 1 | 24796905 | *NIPAL3* |
| cg08540745 | -0.91 | 7.08×10^-34^ | 6.52×10^-29^ | 1 | 25867637 |  |
| cg19112780 | 0.11 | 9.13×10^-6^ | 2.27×10^-2^ | 1 | 29563147 | *PTPRU* |
| cg24693287 | 0.20 | 4.20×10^-12^ | 4.24×10^-8^ | 1 | 31885850 | *SERINC2* |
| cg15436480 | 0.26 | 1.47×10^-5^ | 3.26×10^-2^ | 1 | 33738213 | *ZNF362* |
| cg11537507 | -0.22 | 3.55×10^-6^ | 9.95×10^-3^ | 1 | 37177498 |  |
| cg23341459 | 0.24 | 4.88×10^-6^ | 1.31×10^-2^ | 1 | 40204657 | *PPIE* |
| cg20942219 | -0.56 | 2.53×10^-18^ | 4.90×10^-14^ | 1 | 44141161 | *KDM4A* |
| cg19551082 | -0.08 | 1.61×10^-5^ | 3.52×10^-2^ | 1 | 45956882 | *TESK2* |
| cg21228875 | -0.34 | 9.10×10^-8^ | 4.36×10^-4^ | 1 | 53132253 |  |
| cg02752228 | -0.24 | 2.37×10^-5^ | 4.72×10^-2^ | 1 | 53531092 | *PODN* |
| cg27431882 | -0.17 | 2.43×10^-5^ | 4.78×10^-2^ | 1 | 56724990 |  |
| cg16896501 | -0.43 | 2.59×10^-26^ | 9.09×10^-22^ | 1 | 67661575 | *IL23R* |
| cg09078503 | -0.61 | 3.03×10^-11^ | 2.72×10^-7^ | 1 | 71823474 |  |
| cg26153364 | 0.09 | 2.36×10^-5^ | 4.72×10^-2^ | 1 | 72749064 | *NEGR1* |
| cg25341338 | 0.25 | 1.77×10^-7^ | 7.82×10^-4^ | 1 | 116380575 | *NHLH2* |
| cg08721840 | 0.50 | 6.69×10^-30^ | 3.52×10^-25^ | 1 | 155293541 | *RUSC1* |
| cg17161697 | -1.00 | 3.61×10^-7^ | 1.44×10^-3^ | 1 | 155388145 | *ASH1L* |
| cg07204658 | 0.19 | 2.12×10^-6^ | 6.44×10^-3^ | 1 | 160139687 | *ATP1A4* |
| cg16698369 | -0.34 | 1.46×10^-6^ | 4.64×10^-3^ | 1 | 160231374 | *DCAF8* |
| cg20131145 | -0.96 | 8.93×10^-20^ | 1.88×10^-15^ | 1 | 160296456 | *COPA* |
| cg14484016 | -0.32 | 1.51×10^-5^ | 3.34×10^-2^ | 1 | 180915632 |  |
| cg07195452 | -0.26 | 7.99×10^-15^ | 1.18×10^-10^ | 1 | 182109534 |  |
| cg26913977 | 0.07 | 5.71×10^-6^ | 1.52×10^-2^ | 1 | 192777635 | *RGS2* |
| cg09177518 | 0.12 | 1.82×10^-5^ | 3.87×10^-2^ | 1 | 196578010 | *KCNT2* |
| cg07692949 | 0.15 | 2.32×10^-6^ | 6.96×10^-3^ | 1 | 204425773 | *PIK3C2B* |
| cg05122861 | 0.19 | 4.48×10^-6^ | 1.22×10^-2^ | 1 | 206137774 | *FAM72A* |
| cg19667051 | -0.19 | 9.43×10^-7^ | 3.29×10^-3^ | 1 | 207046933 |  |
| cg16186036 | -0.33 | 4.69×10^-7^ | 1.78×10^-3^ | 1 | 208203760 | *PLXNA2* |
| cg20282303 | 0.25 | 2.44×10^-6^ | 7.21×10^-3^ | 1 | 236924392 | *ACTN2* |
| cg19222330 | -0.19 | 1.94×10^-5^ | 4.08×10^-2^ | 1 | 240635282 | *FMN2* |
| cg03853274 | -0.15 | 6.67×10^-9^ | 4.28×10^-5^ | 2 | 1052702 | *SNTG2* |
| cg13360758 | 0.31 | 2.45×10^-6^ | 7.23×10^-3^ | 2 | 3467963 | *TRAPPC12* |
| cg11768422 | -0.49 | 9.06×10^-6^ | 2.26×10^-2^ | 2 | 39552637 | *MAP4K3* |
| cg09417539 | 0.10 | 9.72×10^-7^ | 3.35×10^-3^ | 2 | 58655075 |  |
| cg26819428 | 0.13 | 5.83×10^-6^ | 1.54×10^-2^ | 2 | 60784878 |  |
| cg11203889 | -0.54 | 5.58×10^-7^ | 2.08×10^-3^ | 2 | 65547670 | *SPRED2* |
| cg00013475 | 0.16 | 1.40×10^-5^ | 3.13×10^-2^ | 2 | 71680721 | *DYSF* |
| cg01695225 | 0.21 | 2.05×10^-6^ | 6.31×10^-3^ | 2 | 73152672 | *EMX1* |
| cg03562266 | 0.09 | 1.38×10^-7^ | 6.35×10^-4^ | 2 | 85359992 | *TCF7L1* |
| cg22865501 | 0.28 | 2.97×10^-7^ | 1.24×10^-3^ | 2 | 105321544 |  |
| cg22168709 | -0.28 | 1.19×10^-7^ | 5.61×10^-4^ | 2 | 106403934 | *NCK2* |
| cg09392940 | 0.11 | 8.42×10^-7^ | 2.98×10^-3^ | 2 | 109745828 | *LOC100287216* |
| cg00940894 | 0.18 | 2.67×10^-7^ | 1.13×10^-3^ | 2 | 122018686 | *TFCP2L1* |
| cg23460843 | 0.24 | 1.43×10^-16^ | 2.50×10^-12^ | 2 | 127644075 |  |
| cg17942639 | 0.08 | 2.11×10^-5^ | 4.37×10^-2^ | 2 | 154335184 | *RPRM* |
| cg20539752 | 0.27 | 2.38×10^-9^ | 1.69×10^-5^ | 2 | 176994305 | *HOXD8* |
| cg00372801 | -0.19 | 8.50×10^-8^ | 4.12×10^-4^ | 2 | 191219050 | *INPP1* |
| cg20391764 | 0.04 | 8.51×10^-6^ | 2.13×10^-2^ | 2 | 192110565 | *MYO1B* |
| cg16498770 | 0.08 | 1.19×10^-6^ | 3.96×10^-3^ | 2 | 216796168 |  |
| cg01352705 | 0.08 | 2.14×10^-7^ | 9.16×10^-4^ | 2 | 216979551 | *XRCC5* |
| cg25108567 | 0.07 | 6.79×10^-6^ | 1.76×10^-2^ | 2 | 216979652 | *XRCC5* |
| cg21084456 | 0.17 | 1.01×10^-5^ | 2.44×10^-2^ | 2 | 217674497 |  |
| cg01793397 | -0.29 | 6.12×10^-11^ | 5.12×10^-7^ | 2 | 218725987 | *TNS1* |
| cg13739417 | 0.12 | 8.28×10^-9^ | 5.20×10^-5^ | 2 | 218990627 | *CXCR2* |
| cg11702639 | 0.18 | 6.74×10^-12^ | 6.53×10^-8^ | 2 | 233285454 |  |
| cg00034781 | 0.15 | 1.03×10^-6^ | 3.51×10^-3^ | 2 | 234400085 | *USP40* |
| cg16801535 | -0.19 | 1.66×10^-5^ | 3.61×10^-2^ | 3 | 9291372 | *SRGAP3* |
| cg10330019 | 0.20 | 9.48×10^-10^ | 6.92×10^-6^ | 3 | 24562864 |  |
| cg23212202 | -0.25 | 8.81×10^-14^ | 1.12×10^-9^ | 3 | 30489255 |  |
| cg26843074 | 0.14 | 6.52×10^-8^ | 3.31×10^-4^ | 3 | 36422061 | *STAC* |
| cg26615813 | 0.39 | 6.39×10^-11^ | 5.29×10^-7^ | 3 | 36422209 | *STAC* |
| cg06197503 | 0.47 | 7.56×10^-12^ | 7.05×10^-8^ | 3 | 36422406 | *STAC* |
| cg13695953 | 0.29 | 1.12×10^-5^ | 2.63×10^-2^ | 3 | 36718919 |  |
| cg07759247 | -0.22 | 1.28×10^-6^ | 4.18×10^-3^ | 3 | 38040636 | *VILL* |
| cg21194499 | -0.15 | 3.43×10^-7^ | 1.39×10^-3^ | 3 | 52188578 | *WDR51A* |
| cg10130162 | -0.53 | 4.68×10^-7^ | 1.78×10^-3^ | 3 | 52324257 | *GLYCTK* |
| cg02517134 | 0.14 | 6.18×10^-8^ | 3.16×10^-4^ | 3 | 89156795 | *EPHA3* |
| cg05148217 | -0.26 | 1.44×10^-5^ | 3.23×10^-2^ | 3 | 108836878 | *MORC1* |
| cg14775821 | 0.20 | 1.20×10^-5^ | 2.77×10^-2^ | 3 | 111622625 | *PHLDB2* |
| cg07707817 | -0.24 | 9.57×10^-7^ | 3.31×10^-3^ | 3 | 121285706 | *ARGFX* |
| cg08049441 | -0.29 | 1.80×10^-5^ | 3.84×10^-2^ | 3 | 129106543 | *RPL32P3* |
| cg08958945 | -0.20 | 1.06×10^-6^ | 3.58×10^-3^ | 3 | 133591690 | *RAB6B* |
| cg14986229 | -0.25 | 1.68×10^-12^ | 1.88×10^-8^ | 3 | 157485683 |  |
| cg06332127 | -0.38 | 5.81×10^-7^ | 2.15×10^-3^ | 3 | 158763606 |  |
| cg22329918 | 0.41 | 3.10×10^-9^ | 2.09×10^-5^ | 3 | 184430010 | *MAGEF1* |
| cg19798703 | 0.27 | 3.74×10^-6^ | 1.04×10^-2^ | 3 | 186327747 |  |
| cg10339098 | -0.41 | 2.89×10^-42^ | 4.26×10^-37^ | 3 | 188313650 | *LPP* |
| cg11213655 | -0.83 | 9.22×10^-11^ | 7.55×10^-7^ | 3 | 190276776 | *IL1RAP* |
| cg03637670 | 0.15 | 2.08×10^-5^ | 4.32×10^-2^ | 3 | 193500764 |  |
| cg18279658 | 0.48 | 3.82×10^-28^ | 1.48×10^-23^ | 4 | 1341442 | *UVSSA* |
| cg22609114 | -0.59 | 1.14×10^-8^ | 6.79×10^-5^ | 4 | 4239516 | *TMEM128* |
| cg12237849 | 0.15 | 3.89×10^-7^ | 1.53×10^-3^ | 4 | 7014942 | *TBC1D14* |
| cg18679164 | -0.24 | 4.74×10^-11^ | 4.02×10^-7^ | 4 | 7875418 | *AFAP1* |
| cg13382475 | -0.24 | 4.56×10^-11^ | 3.91×10^-7^ | 4 | 7896151 | *AFAP1* |
| cg25688164 | 0.18 | 4.66×10^-6^ | 1.26×10^-2^ | 4 | 10110163 | *WDR1* |
| cg10262842 | 0.16 | 3.54×10^-6^ | 9.95×10^-3^ | 4 | 15233600 | *LOC101929095* |
| cg01120514 | -0.24 | 1.05×10^-7^ | 5.01×10^-4^ | 4 | 25314414 | *ZCCHC4* |
| cg22599477 | -0.38 | 4.49×10^-7^ | 1.73×10^-3^ | 4 | 25507245 |  |
| cg20445582 | 0.06 | 3.35×10^-12^ | 3.52×10^-8^ | 4 | 55083843 |  |
| cg02722188 | 0.22 | 1.46×10^-7^ | 6.64×10^-4^ | 4 | 55099058 | *PDGFRA* |
| cg18930905 | -0.31 | 7.35×10^-6^ | 1.89×10^-2^ | 4 | 57180387 | *KIAA1211* |
| cg14538788 | 0.24 | 1.69×10^-5^ | 3.66×10^-2^ | 4 | 74402714 |  |
| cg02886293 | -0.41 | 4.26×10^-11^ | 3.74×10^-7^ | 4 | 111462716 | *ENPEP* |
| cg04966600 | -0.60 | 3.87×10^-8^ | 2.04×10^-4^ | 4 | 154430913 | *KIAA0922* |
| cg12836011 | 0.07 | 2.07×10^-6^ | 6.36×10^-3^ | 4 | 166795260 | *TLL1* |
| cg19940052 | -0.21 | 7.72×10^-12^ | 7.11×10^-8^ | 5 | 5474505 | *KIAA0947* |
| cg10556369 | 0.16 | 4.02×10^-7^ | 1.58×10^-3^ | 5 | 36619118 | *SLC1A3* |
| cg22232815 | -0.36 | 2.22×10^-5^ | 4.55×10^-2^ | 5 | 37169220 | *C5orf42* |
| cg26965238 | 0.18 | 1.52×10^-5^ | 3.35×10^-2^ | 5 | 50679374 | *ISL1* |
| cg05405124 | -0.24 | 1.93×10^-7^ | 8.31×10^-4^ | 5 | 71748948 | *ZNF366* |
| cg13665642 | -0.19 | 7.87×10^-8^ | 3.87×10^-4^ | 5 | 73631417 |  |
| cg23941527 | 0.42 | 1.03×10^-5^ | 2.48×10^-2^ | 5 | 92868380 | *FLJ42709* |
| cg14981372 | -0.45 | 1.01×10^-5^ | 2.44×10^-2^ | 5 | 95189852 | *LINC01554* |
| cg18738397 | -0.30 | 9.84×10^-7^ | 3.37×10^-3^ | 5 | 96237212 | *ERAP2* |
| cg05491087 | -0.25 | 2.68×10^-10^ | 2.06×10^-6^ | 5 | 111039790 | *STARD4-AS1* |
| cg02103653 | -0.60 | 2.51×10^-14^ | 3.43×10^-10^ | 5 | 111504758 | *EPB41L4A* |
| cg26201826 | -0.26 | 1.58×10^-7^ | 7.05×10^-4^ | 5 | 114598579 | *PGGT1B* |
| cg05883541 | -0.42 | 6.40×10^-9^ | 4.14×10^-5^ | 5 | 131637405 | *SLC22A4* |
| cg04577715 | -0.52 | 6.66×10^-10^ | 4.91×10^-6^ | 5 | 147211181 | *SPINK1* |
| cg20548393 | 0.15 | 4.47×10^-6^ | 1.22×10^-2^ | 5 | 156934039 | *ADAM19* |
| cg00982641 | -0.07 | 2.91×10^-6^ | 8.40×10^-3^ | 5 | 169659845 | *C5orf58* |
| cg17338430 | 0.07 | 1.09×10^-5^ | 2.59×10^-2^ | 5 | 170739853 |  |
| cg11565366 | -0.32 | 1.76×10^-5^ | 3.78×10^-2^ | 5 | 171482633 | *STK10* |
| cg17807913 | -0.45 | 2.19×10^-48^ | 8.06×10^-43^ | 5 | 176525355 |  |
| cg11466504 | -0.56 | 1.81×10^-13^ | 2.18×10^-9^ | 5 | 179565362 | *RASGEF1C* |
| cg09103423 | -0.46 | 1.49×10^-7^ | 6.75×10^-4^ | 6 | 4665238 |  |
| cg21641834 | 0.17 | 3.89×10^-7^ | 1.53×10^-3^ | 6 | 5068684 |  |
| cg09341695 | 0.19 | 1.95×10^-5^ | 4.08×10^-2^ | 6 | 20545304 | *CDKAL1* |
| cg16454551 | 0.43 | 5.98×10^-9^ | 3.90×10^-5^ | 6 | 27125990 |  |
| cg24544803 | 0.10 | 2.32×10^-5^ | 4.70×10^-2^ | 6 | 29596840 | *GABBR1* |
| cg13434772 | -0.32 | 1.79×10^-6^ | 5.57×10^-3^ | 6 | 30173342 | *TRIM26* |
| cg14219599 | 0.07 | 1.61×10^-5^ | 3.52×10^-2^ | 6 | 30523389 | *GNL1* |
| cg06332410 | 0.21 | 6.40×10^-12^ | 6.29×10^-8^ | 6 | 30523414 | *GNL1* |
| cg26905324 | -0.09 | 1.01×10^-5^ | 2.44×10^-2^ | 6 | 31510332 | *SNORD84* |
| cg16918667 | -0.35 | 1.42×10^-13^ | 1.74×10^-9^ | 6 | 31827573 | *NEU1* |
| cg09996288 | 0.05 | 1.27×10^-8^ | 7.48×10^-5^ | 6 | 32055517 | *TNXB* |
| cg17001689 | 0.09 | 1.78×10^-7^ | 7.82×10^-4^ | 6 | 32055525 | *TNXB* |
| cg02591634 | -0.97 | 8.72×10^-30^ | 4.28×10^-25^ | 6 | 32363268 | *BTNL2* |
| cg05700142 | 0.05 | 3.29×10^-6^ | 9.36×10^-3^ | 6 | 33280454 | *TAPBP* |
| cg01654446 | 0.15 | 1.31×10^-14^ | 1.83×10^-10^ | 6 | 33280476 | *TAPBP* |
| cg18930100 | 0.14 | 3.92×10^-12^ | 4.07×10^-8^ | 6 | 33280478 | *TAPBP* |
| cg05742293 | 0.19 | 1.03×10^-6^ | 3.51×10^-3^ | 6 | 33374195 | *KIFC1* |
| cg15689154 | -0.19 | 2.14×10^-5^ | 4.40×10^-2^ | 6 | 37137227 | *PIM1* |
| cg27315243 | -0.10 | 9.50×10^-6^ | 2.32×10^-2^ | 6 | 40554736 | *LRFN2* |
| cg17004121 | 0.33 | 6.93×10^-8^ | 3.45×10^-4^ | 6 | 43258821 |  |
| cg00071161 | -0.35 | 4.06×10^-12^ | 4.15×10^-8^ | 6 | 44117573 | *TMEM63B* |
| cg20102583 | -0.22 | 1.19×10^-10^ | 9.40×10^-7^ | 6 | 44171055 |  |
| cg26289989 | -0.62 | 1.06×10^-10^ | 8.49×10^-7^ | 6 | 51085750 |  |
| cg23215729 | 0.11 | 7.51×10^-6^ | 1.92×10^-2^ | 6 | 84562928 | *RIPPLY2* |
| cg22442841 | 0.45 | 9.52×10^-11^ | 7.71×10^-7^ | 6 | 87646804 | *HTR1E* |
| cg26532199 | 0.53 | 2.09×10^-5^ | 4.34×10^-2^ | 6 | 106671344 | *ATG5* |
| cg07884432 | -0.32 | 1.15×10^-12^ | 1.31×10^-8^ | 6 | 112513042 | *LAMA4* |
| cg17679781 | 0.52 | 3.77×10^-29^ | 1.63×10^-24^ | 6 | 114663223 |  |
| cg10369203 | -0.97 | 5.79×10^-21^ | 1.33×10^-16^ | 6 | 143250783 | *HIVEP2* |
| cg20043883 | -0.56 | 1.06×10^-21^ | 2.60×10^-17^ | 6 | 149289357 | *UST* |
| cg00034668 | -0.23 | 6.50×10^-10^ | 4.84×10^-6^ | 6 | 162531181 | *PARK2* |
| cg08504806 | -0.28 | 1.16×10^-12^ | 1.31×10^-8^ | 7 | 2027239 | *MAD1L1* |
| cg00135038 | -0.26 | 1.21×10^-6^ | 3.97×10^-3^ | 7 | 5391419 | *TNRC18* |
| cg04408923 | 0.06 | 1.77×10^-6^ | 5.54×10^-3^ | 7 | 5464875 |  |
| cg09339462 | 0.16 | 2.05×10^-8^ | 1.18×10^-4^ | 7 | 25935223 |  |
| cg14505161 | -0.10 | 2.75×10^-6^ | 8.02×10^-3^ | 7 | 30544458 | *GGCT* |
| cg21999940 | -0.38 | 1.19×10^-6^ | 3.96×10^-3^ | 7 | 44799435 | *ZMIZ2* |
| cg17067067 | -0.29 | 3.94×10^-6^ | 1.09×10^-2^ | 7 | 81582101 | *CACNA2D1* |
| cg00786335 | -0.37 | 7.53×10^-6^ | 1.92×10^-2^ | 7 | 82984236 |  |
| cg20561100 | -0.13 | 3.73×10^-6^ | 1.04×10^-2^ | 7 | 107204135 | *COG5* |
| cg03192996 | 0.15 | 2.41×10^-5^ | 4.76×10^-2^ | 7 | 116072114 |  |
| cg22384902 | 0.05 | 1.84×10^-7^ | 8.04×10^-4^ | 7 | 127671261 | *LRRC4* |
| cg12891311 | -0.52 | 1.39×10^-8^ | 8.11×10^-5^ | 7 | 133093517 | *EXOC4* |
| cg15542496 | -0.41 | 9.90×10^-14^ | 1.24×10^-9^ | 7 | 142828356 | *PIP* |
| cg09383172 | -0.12 | 1.38×10^-6^ | 4.41×10^-3^ | 7 | 157932921 | *PTPRN2* |
| cg15614872 | -0.23 | 9.63×10^-6^ | 2.35×10^-2^ | 7 | 158531752 | *ESYT2* |
| cg05779585 | -0.49 | 8.60×10^-8^ | 4.14×10^-4^ | 8 | 1246817 |  |
| cg13636978 | -0.34 | 2.52×10^-5^ | 4.94×10^-2^ | 8 | 15598297 | *TUSC3* |
| cg16663570 | 0.10 | 8.45×10^-6^ | 2.13×10^-2^ | 8 | 22014724 | *LGI3* |
| cg07085824 | -0.05 | 2.00×10^-5^ | 4.17×10^-2^ | 8 | 42948105 | *SGK196* |
| cg17484036 | 0.11 | 2.66×10^-6^ | 7.78×10^-3^ | 8 | 63160761 | *NKAIN3* |
| cg23809497 | 0.26 | 2.52×10^-8^ | 1.41×10^-4^ | 8 | 72469888 |  |
| cg19098726 | -0.70 | 4.71×10^-7^ | 1.78×10^-3^ | 8 | 96216628 |  |
| cg03224572 | 0.14 | 1.27×10^-5^ | 2.88×10^-2^ | 8 | 97172012 | *GDF6* |
| cg14159108 | -0.26 | 3.69×10^-9^ | 2.45×10^-5^ | 8 | 106370733 | *ZFPM2* |
| cg18833573 | 0.31 | 1.08×10^-14^ | 1.57×10^-10^ | 8 | 144822305 |  |
| cg11321802 | 0.19 | 1.84×10^-5^ | 3.90×10^-2^ | 9 | 18438845 |  |
| cg19133618 | 0.05 | 1.10×10^-5^ | 2.59×10^-2^ | 9 | 22008970 | *CDKN2B* |
| cg15988322 | -0.46 | 5.60×10^-15^ | 8.42×10^-11^ | 9 | 22098908 | *CDKN2B-AS1* |
| cg09587920 | -0.47 | 3.11×10^-13^ | 3.70×10^-9^ | 9 | 73005204 | *KLF9* |
| cg26196886 | 0.28 | 3.05×10^-6^ | 8.77×10^-3^ | 9 | 74102260 |  |
| cg05043065 | 0.22 | 2.16×10^-5^ | 4.44×10^-2^ | 9 | 84229438 | *TLE1* |
| cg14185994 | -0.13 | 1.71×10^-7^ | 7.58×10^-4^ | 9 | 98637332 | *C9orf130* |
| cg14067419 | 0.06 | 2.22×10^-6^ | 6.70×10^-3^ | 9 | 98783308 | *NCRNA00092* |
| cg13653482 | 0.03 | 1.35×10^-7^ | 6.26×10^-4^ | 9 | 103361209 |  |
| cg14614881 | -0.68 | 1.10×10^-22^ | 3.01×10^-18^ | 9 | 116982494 | *COL27A1* |
| cg03484259 | -0.43 | 1.76×10^-42^ | 3.24×10^-37^ | 9 | 117049185 | *COL27A1* |
| cg13469953 | 0.16 | 1.15×10^-6^ | 3.85×10^-3^ | 9 | 117271936 |  |
| cg10796900 | 0.21 | 5.86×10^-6^ | 1.55×10^-2^ | 9 | 117314805 |  |
| cg10755973 | 0.30 | 8.59×10^-19^ | 1.71×10^-14^ | 9 | 126773879 | *LHX2* |
| cg04456155 | 0.18 | 6.21×10^-10^ | 4.67×10^-6^ | 9 | 126773885 | *LHX2* |
| cg06177522 | 0.08 | 3.50×10^-8^ | 1.90×10^-4^ | 9 | 126773887 | *LHX2* |
| cg02992617 | -0.24 | 1.77×10^-5^ | 3.78×10^-2^ | 9 | 127264323 | *NR5A1* |
| cg05593880 | 0.11 | 1.25×10^-5^ | 2.87×10^-2^ | 9 | 131798822 | *FAM73B* |
| cg14094375 | -0.28 | 3.42×10^-9^ | 2.29×10^-5^ | 9 | 133306791 |  |
| cg13521319 | 0.20 | 8.33×10^-9^ | 5.20×10^-5^ | 9 | 133423844 |  |
| cg13875536 | 0.07 | 1.34×10^-6^ | 4.32×10^-3^ | 9 | 135464814 | *BARHL1* |
| cg21389456 | 0.30 | 9.28×10^-6^ | 2.29×10^-2^ | 10 | 675937 | *DIP2C* |
| cg26873457 | -0.30 | 2.40×10^-6^ | 7.15×10^-3^ | 10 | 729204 | *DIP2C* |
| cg02773337 | 0.09 | 1.67×10^-6^ | 5.24×10^-3^ | 10 | 3171970 | *PFKP* |
| cg08707849 | 0.37 | 1.33×10^-5^ | 3.00×10^-2^ | 10 | 13061436 | *CCDC3* |
| cg05101502 | 0.32 | 9.49×10^-6^ | 2.32×10^-2^ | 10 | 15767601 |  |
| cg07659571 | 0.15 | 1.22×10^-5^ | 2.81×10^-2^ | 10 | 15839446 | *FAM188A* |
| cg18950772 | -0.26 | 5.90×10^-6^ | 1.55×10^-2^ | 10 | 44758342 |  |
| cg14325837 | 0.16 | 1.88×10^-5^ | 3.96×10^-2^ | 10 | 49731563 | *ARHGAP22* |
| cg25721039 | 0.15 | 3.30×10^-7^ | 1.34×10^-3^ | 10 | 49732419 | *ARHGAP22* |
| cg18858343 | 0.11 | 5.37×10^-6^ | 1.43×10^-2^ | 10 | 72972901 | *UNC5B* |
| cg04326863 | -0.68 | 6.16×10^-12^ | 6.13×10^-8^ | 10 | 87923307 | *GRID1* |
| cg19130698 | -0.16 | 4.51×10^-7^ | 1.73×10^-3^ | 10 | 91011807 | *LIPA* |
| cg06093253 | -0.19 | 1.06×10^-6^ | 3.58×10^-3^ | 10 | 100194230 | *HPS1* |
| cg10632966 | -0.31 | 1.49×10^-5^ | 3.30×10^-2^ | 10 | 105001051 |  |
| cg19876672 | -0.32 | 1.32×10^-5^ | 2.99×10^-2^ | 10 | 118891266 | *VAX1* |
| cg14990252 | -0.38 | 9.51×10^-7^ | 3.31×10^-3^ | 10 | 125691586 |  |
| cg26078803 | -0.28 | 3.24×10^-20^ | 7.03×10^-16^ | 10 | 126298642 | *LHPP* |
| cg00561124 | -0.50 | 6.41×10^-7^ | 2.36×10^-3^ | 10 | 134410680 | *INPP5A* |
| cg24915508 | -0.25 | 1.89×10^-5^ | 3.97×10^-2^ | 10 | 134647082 |  |
| cg11633280 | -0.36 | 4.70×10^-8^ | 2.44×10^-4^ | 11 | 3254452 | *MRGPRE* |
| cg06407663 | 0.05 | 4.26×10^-9^ | 2.81×10^-5^ | 11 | 33279084 | *HIPK3* |
| cg00149585 | 0.07 | 7.68×10^-8^ | 3.80×10^-4^ | 11 | 47207601 | *PACSIN3* |
| cg06992252 | -0.30 | 2.46×10^-7^ | 1.05×10^-3^ | 11 | 71258482 | *KRTAP5-9* |
| cg18646301 | 0.23 | 8.75×10^-7^ | 3.07×10^-3^ | 11 | 78089098 | *GAB2* |
| cg10043393 | 0.31 | 7.47×10^-7^ | 2.66×10^-3^ | 11 | 83354605 | *DLG2* |
| cg09114151 | -0.30 | 3.12×10^-7^ | 1.29×10^-3^ | 11 | 85956200 | *EED* |
| cg08402652 | 0.20 | 3.08×10^-6^ | 8.84×10^-3^ | 11 | 94501461 | *AMOTL1* |
| cg24783356 | 0.17 | 6.79×10^-6^ | 1.76×10^-2^ | 11 | 98891211 | *CNTN5* |
| cg24452821 | 0.21 | 3.03×10^-7^ | 1.25×10^-3^ | 11 | 113953812 | *ZBTB16* |
| cg05297666 | 0.16 | 3.24×10^-6^ | 9.25×10^-3^ | 11 | 128400310 | *ETS1* |
| cg06072950 | 0.15 | 8.48×10^-7^ | 2.99×10^-3^ | 11 | 129736336 | *NFRKB* |
| cg16181883 | -0.25 | 2.23×10^-12^ | 2.41×10^-8^ | 11 | 129738984 | *NFRKB* |
| cg02986334 | -0.50 | 2.30×10^-6^ | 6.93×10^-3^ | 12 | 292211 |  |
| cg00573623 | -0.28 | 6.44×10^-7^ | 2.36×10^-3^ | 12 | 3724731 | *EFCAB4B* |
| cg20392607 | 0.07 | 4.29×10^-6^ | 1.18×10^-2^ | 12 | 5018798 | *KCNA1* |
| cg19651132 | 0.08 | 4.48×10^-6^ | 1.22×10^-2^ | 12 | 5018805 | *KCNA1* |
| cg12682976 | -0.41 | 1.31×10^-6^ | 4.24×10^-3^ | 12 | 9391384 | *LINC00987* |
| cg03434384 | 0.38 | 8.35×10^-6^ | 2.11×10^-2^ | 12 | 27703097 | *PPFIBP1* |
| cg18130934 | -0.43 | 1.20×10^-7^ | 5.61×10^-4^ | 12 | 50452837 | *ASIC1* |
| cg00425708 | 0.15 | 6.71×10^-7^ | 2.45×10^-3^ | 12 | 66217779 | *HMGA2* |
| cg23657865 | 0.15 | 3.57×10^-7^ | 1.43×10^-3^ | 12 | 66217793 | *HMGA2* |
| cg08709915 | -0.28 | 1.23×10^-5^ | 2.83×10^-2^ | 12 | 94620199 | *PLXNC1* |
| cg11259038 | 0.43 | 1.69×10^-5^ | 3.67×10^-2^ | 12 | 99392605 | *ANKS1B* |
| cg11032439 | -0.21 | 2.59×10^-6^ | 7.61×10^-3^ | 12 | 106564208 |  |
| cg03240232 | -1.00 | 1.93×10^-31^ | 1.42×10^-26^ | 12 | 113448327 | *OAS2* |
| cg27584467 | 0.06 | 3.24×10^-11^ | 2.87×10^-7^ | 12 | 114029423 |  |
| cg04150382 | 0.48 | 6.14×10^-25^ | 1.97×10^-20^ | 12 | 121533801 |  |
| cg05146493 | -0.40 | 1.75×10^-5^ | 3.76×10^-2^ | 12 | 121953475 | *KDM2B* |
| cg27508046 | -0.23 | 1.19×10^-5^ | 2.77×10^-2^ | 12 | 124249428 | *DNAH10* |
| cg05253716 | 0.22 | 2.40×10^-5^ | 4.75×10^-2^ | 12 | 129299332 | *SLC15A4* |
| cg22040672 | 0.29 | 4.22×10^-7^ | 1.65×10^-3^ | 12 | 129299605 | *MGC16384* |
| cg02656049 | -0.19 | 1.77×10^-5^ | 3.78×10^-2^ | 12 | 133100051 | *FBRSL1* |
| cg10788674 | -0.05 | 2.23×10^-8^ | 1.27×10^-4^ | 12 | 133214645 | *POLE* |
| cg14168939 | 0.21 | 2.35×10^-5^ | 4.72×10^-2^ | 12 | 133417324 | *CHFR* |
| cg16708680 | -0.21 | 1.66×10^-6^ | 5.24×10^-3^ | 13 | 30996972 | *LINC01058* |
| cg13777984 | -0.44 | 9.51×10^-45^ | 2.34×10^-39^ | 13 | 43931730 | *ENOX1* |
| cg10253720 | -0.32 | 2.35×10^-5^ | 4.72×10^-2^ | 13 | 52596008 | *ALG11* |
| cg25348433 | 0.14 | 1.10×10^-5^ | 2.60×10^-2^ | 13 | 78519244 | *EDNRB* |
| cg03637815 | 0.11 | 3.76×10^-8^ | 2.01×10^-4^ | 13 | 95359718 |  |
| cg23235217 | -0.08 | 2.77×10^-7^ | 1.16×10^-3^ | 13 | 100741026 | *PCCA* |
| cg16342824 | -0.44 | 5.52×10^-7^ | 2.06×10^-3^ | 13 | 106929305 |  |
| cg24640561 | -0.47 | 1.46×10^-7^ | 6.64×10^-4^ | 13 | 108979138 |  |
| cg16256094 | 0.09 | 5.41×10^-14^ | 7.24×10^-10^ | 14 | 23652905 | *SLC7A8* |
| cg10769343 | -0.22 | 1.94×10^-8^ | 1.12×10^-4^ | 14 | 24617188 | *PSME2* |
| cg18884313 | -0.32 | 2.68×10^-7^ | 1.13×10^-3^ | 14 | 51287294 | *NIN* |
| cg00100184 | -0.15 | 1.26×10^-5^ | 2.88×10^-2^ | 14 | 56014589 |  |
| cg06333164 | 0.22 | 3.82×10^-8^ | 2.03×10^-4^ | 14 | 68752237 | *RAD51B* |
| cg01680773 | -0.14 | 2.12×10^-6^ | 6.44×10^-3^ | 14 | 74960124 | *NPC2* |
| cg07510327 | -0.26 | 7.07×10^-12^ | 6.74×10^-8^ | 14 | 76450897 | *IFT43* |
| cg12838644 | 0.17 | 4.08×10^-23^ | 1.16×10^-18^ | 14 | 77413642 |  |
| cg22903735 | 0.20 | 3.68×10^-22^ | 9.36×10^-18^ | 14 | 77413674 |  |
| cg24703525 | -0.37 | 7.59×10^-9^ | 4.82×10^-5^ | 14 | 79665634 | *NRXN3* |
| cg05454389 | -0.42 | 6.80×10^-8^ | 3.43×10^-4^ | 15 | 22992979 | *CYFIP1* |
| cg10656128 | -0.26 | 5.35×10^-6^ | 1.43×10^-2^ | 15 | 29261921 | *APBA2* |
| cg15336988 | -0.40 | 2.91×10^-22^ | 7.65×10^-18^ | 15 | 31566306 |  |
| cg07709681 | -0.13 | 1.26×10^-6^ | 4.14×10^-3^ | 15 | 34393712 | *PGBD4* |
| cg19999645 | 0.09 | 2.79×10^-9^ | 1.92×10^-5^ | 15 | 35047394 | *GJD2* |
| cg22134372 | -0.19 | 3.01×10^-9^ | 2.05×10^-5^ | 15 | 52455211 | *GNB5* |
| cg11774529 | 0.29 | 2.09×10^-6^ | 6.38×10^-3^ | 15 | 57933038 | *GCOM1* |
| cg21496797 | -0.35 | 3.20×10^-8^ | 1.75×10^-4^ | 15 | 67433037 | *SMAD3* |
| cg15100456 | -0.34 | 4.55×10^-25^ | 1.52×10^-20^ | 15 | 68809904 |  |
| cg02687928 | 0.08 | 3.36×10^-6^ | 9.53×10^-3^ | 15 | 72612470 | *CELF6* |
| cg16711004 | 0.05 | 9.18×10^-9^ | 5.69×10^-5^ | 15 | 75249027 | *RPP25* |
| cg11637053 | 0.13 | 7.46×10^-6^ | 1.92×10^-2^ | 15 | 78432103 |  |
| cg20081969 | 0.24 | 2.25×10^-8^ | 1.27×10^-4^ | 15 | 80697055 | *ARNT2* |
| cg24049235 | -0.30 | 9.12×10^-6^ | 2.27×10^-2^ | 15 | 90235068 | *PEX11A* |
| cg10462898 | -0.41 | 8.79×10^-13^ | 1.03×10^-8^ | 15 | 92075216 |  |
| cg26739280 | 0.09 | 2.45×10^-12^ | 2.61×10^-8^ | 15 | 92936818 | *ST8SIA2* |
| cg27475593 | -0.91 | 2.77×10^-9^ | 1.92×10^-5^ | 15 | 96602954 |  |
| cg13398806 | 0.13 | 1.28×10^-5^ | 2.89×10^-2^ | 15 | 98503783 | *ARRDC4* |
| cg04822808 | 0.08 | 4.19×10^-6^ | 1.16×10^-2^ | 15 | 100882162 | *ADAMTS17* |
| cg05027458 | 0.11 | 1.09×10^-5^ | 2.59×10^-2^ | 15 | 100882165 | *ADAMTS17* |
| cg01037989 | -0.63 | 9.45×10^-28^ | 3.48×10^-23^ | 16 | 966659 | *LMF1* |
| cg00730182 | 0.20 | 7.25×10^-7^ | 2.59×10^-3^ | 16 | 12161636 | *SNX29* |
| cg11851825 | -0.21 | 2.13×10^-5^ | 4.40×10^-2^ | 16 | 51735350 |  |
| cg18262140 | -0.43 | 5.00×10^-15^ | 7.80×10^-11^ | 16 | 55082847 |  |
| cg02945354 | -0.43 | 4.21×10^-21^ | 1.00×10^-16^ | 16 | 64295125 |  |
| cg14029669 | 0.17 | 6.96×10^-7^ | 2.51×10^-3^ | 16 | 84330741 | *WFDC1* |
| cg19317774 | 0.18 | 1.54×10^-5^ | 3.39×10^-2^ | 16 | 84861727 | *CRISPLD2* |
| cg02711510 | 0.38 | 8.34×10^-8^ | 4.07×10^-4^ | 16 | 85220747 |  |
| cg07545858 | -0.34 | 1.30×10^-7^ | 6.04×10^-4^ | 16 | 88482626 |  |
| cg00538591 | 0.43 | 2.56×10^-5^ | 5.00×10^-2^ | 16 | 89008994 | *CBFA2T3* |
| cg04329264 | 0.12 | 2.09×10^-9^ | 1.50×10^-5^ | 16 | 89557219 | *ANKRD11* |
| cg01287428 | 0.16 | 3.20×10^-7^ | 1.31×10^-3^ | 16 | 89572067 |  |
| cg15249062 | 0.09 | 7.98×10^-6^ | 2.02×10^-2^ | 17 | 2415618 | *METT10D* |
| cg26574709 | 0.08 | 5.05×10^-7^ | 1.90×10^-3^ | 17 | 4651439 |  |
| cg01524893 | 0.29 | 3.91×10^-8^ | 2.04×10^-4^ | 17 | 12877227 | *RICH2* |
| cg13405332 | 0.19 | 1.92×10^-6^ | 5.94×10^-3^ | 17 | 19483367 |  |
| cg11295144 | 0.51 | 2.67×10^-33^ | 2.19×10^-28^ | 17 | 33447001 | *RAD51L3* |
| cg24761507 | 0.09 | 6.91×10^-8^ | 3.45×10^-4^ | 17 | 35293930 | *LHX1* |
| cg21848909 | -0.49 | 2.67×10^-18^ | 5.04×10^-14^ | 17 | 43365148 | *MAP3K14* |
| cg26892115 | 0.05 | 5.70×10^-14^ | 7.49×10^-10^ | 17 | 48636898 | *CACNA1G-AS1* |
| cg06685177 | 0.08 | 9.17×10^-6^ | 2.27×10^-2^ | 17 | 71161322 | *SSTR2* |
| cg08976894 | 0.11 | 1.69×10^-6^ | 5.30×10^-3^ | 17 | 74100186 | *EXOC7* |
| cg21487261 | 0.11 | 1.08×10^-8^ | 6.47×10^-5^ | 17 | 79368981 |  |
| cg18089670 | 0.33 | 6.83×10^-7^ | 2.48×10^-3^ | 18 | 660756 | *TYMS* |
| cg18554492 | -0.85 | 9.82×10^-9^ | 5.98×10^-5^ | 18 | 19406813 | *MIR133A1HG* |
| cg23663881 | -0.51 | 3.62×10^-8^ | 1.95×10^-4^ | 18 | 19664794 |  |
| cg23286333 | -0.47 | 1.03×10^-8^ | 6.20×10^-5^ | 18 | 39112519 |  |
| cg13309716 | 0.33 | 7.23×10^-6^ | 1.87×10^-2^ | 18 | 46271671 | *CTIF* |
| cg27113059 | 0.16 | 1.63×10^-9^ | 1.18×10^-5^ | 18 | 47088248 | *LIPG* |
| cg26635170 | -0.35 | 3.63×10^-23^ | 1.07×10^-18^ | 18 | 55907926 | *NEDD4L* |
| cg17185889 | -0.22 | 1.13×10^-14^ | 1.60×10^-10^ | 18 | 74685615 |  |
| cg16421653 | -0.51 | 3.09×10^-16^ | 5.06×10^-12^ | 18 | 77251876 | *NFATC1* |
| cg08091147 | 0.20 | 9.36×10^-9^ | 5.74×10^-5^ | 19 | 1676011 |  |
| cg26090256 | -0.25 | 1.08×10^-5^ | 2.57×10^-2^ | 19 | 3958507 | *DAPK3* |
| cg15810996 | 0.20 | 1.26×10^-5^ | 2.88×10^-2^ | 19 | 4045686 | *ZBTB7A* |
| cg04592958 | -0.14 | 9.51×10^-17^ | 1.71×10^-12^ | 19 | 5138093 | *KDM4B* |
| cg21658616 | 0.03 | 7.94×10^-6^ | 2.02×10^-2^ | 19 | 5214593 | *PTPRS* |
| cg19412076 | 0.19 | 1.19×10^-5^ | 2.77×10^-2^ | 19 | 12661732 | *ZNF564* |
| cg22071238 | 0.77 | 2.33×10^-16^ | 3.90×10^-12^ | 19 | 36630543 | *CAPNS1* |
| cg07722395 | -0.31 | 2.33×10^-10^ | 1.82×10^-6^ | 19 | 42396778 | *ARHGEF1* |
| cg10632000 | 0.20 | 2.29×10^-5^ | 4.67×10^-2^ | 19 | 42806201 | *PRR19* |
| cg27337277 | -0.42 | 3.54×10^-7^ | 1.42×10^-3^ | 19 | 44040457 |  |
| cg07101466 | -0.57 | 1.51×10^-24^ | 4.65×10^-20^ | 19 | 47197139 | *PRKD2* |
| cg23300543 | 0.60 | 1.25×10^-29^ | 5.74×10^-25^ | 19 | 48707195 |  |
| cg01622465 | 0.58 | 1.21×10^-36^ | 1.48×10^-31^ | 19 | 48707291 |  |
| cg15073624 | 0.59 | 4.59×10^-29^ | 1.88×10^-24^ | 19 | 48707384 |  |
| cg19771773 | 0.70 | 2.36×10^-31^ | 1.58×10^-26^ | 19 | 48707517 |  |
| cg10714492 | 0.24 | 6.75×10^-6^ | 1.76×10^-2^ | 19 | 51321395 |  |
| cg15985184 | 0.33 | 1.55×10^-7^ | 6.97×10^-4^ | 19 | 51830311 | *IGLON5* |
| cg07382943 | 0.14 | 6.24×10^-6^ | 1.64×10^-2^ | 19 | 51830477 | *IGLON5* |
| cg02876237 | 0.20 | 4.79×10^-8^ | 2.47×10^-4^ | 19 | 52531528 | *ZNF614* |
| cg24021890 | 0.08 | 1.32×10^-6^ | 4.28×10^-3^ | 19 | 52552120 | *ZNF432* |
| cg11919525 | 0.11 | 7.06×10^-7^ | 2.54×10^-3^ | 19 | 52552461 | *ZNF432* |
| cg08278357 | 0.07 | 2.43×10^-6^ | 7.21×10^-3^ | 19 | 59050122 |  |
| cg03925157 | 0.08 | 4.77×10^-6^ | 1.29×10^-2^ | 20 | 17549955 | *DSTN* |
| cg22736850 | 0.15 | 2.37×10^-5^ | 4.72×10^-2^ | 20 | 18039663 | *OVOL2* |
| cg13330919 | -0.44 | 1.29×10^-30^ | 7.93×10^-26^ | 20 | 20205957 | *CFAP61* |
| cg15592828 | -0.31 | 9.32×10^-21^ | 2.08×10^-16^ | 20 | 36890518 | *KIAA1755* |
| cg02551396 | 0.11 | 1.74×10^-5^ | 3.75×10^-2^ | 20 | 42815882 | *JPH2* |
| cg13690178 | 0.10 | 4.55×10^-11^ | 3.91×10^-7^ | 20 | 45338329 | *SLC2A10* |
| cg05494607 | -0.19 | 4.38×10^-7^ | 1.70×10^-3^ | 20 | 50416707 | *SALL4* |
| cg10671478 | 0.48 | 2.49×10^-5^ | 4.89×10^-2^ | 20 | 52790299 | *CYP24A1* |
| cg05120028 | 0.22 | 2.22×10^-16^ | 3.81×10^-12^ | 20 | 52790712 | *CYP24A1* |
| cg02712555 | 0.12 | 2.98×10^-19^ | 6.09×10^-15^ | 20 | 52790733 | *CYP24A1* |
| cg02673418 | 0.16 | 3.41×10^-17^ | 6.29×10^-13^ | 20 | 52790978 | *CYP24A1* |
| cg06373721 | -0.19 | 2.33×10^-5^ | 4.71×10^-2^ | 20 | 62745242 |  |
| cg08015776 | 0.10 | 2.96×10^-15^ | 4.75×10^-11^ | 21 | 27543229 | *APP* |
| cg01158367 | -0.70 | 4.68×10^-30^ | 2.65×10^-25^ | 21 | 30258251 | *N6AMT1* |
| cg13108038 | -1.00 | 1.96×10^-77^ | 1.44×10^-71^ | 21 | 30607031 | *LINC00189* |
| cg11073863 | -0.27 | 1.71×10^-12^ | 1.88×10^-8^ | 21 | 38010424 |  |
| cg04477510 | -0.44 | 1.20×10^-6^ | 3.96×10^-3^ | 21 | 44582492 |  |
| cg15396990 | 0.09 | 2.39×10^-5^ | 4.75×10^-2^ | 21 | 45758391 | *C21orf2* |
| cg00375340 | -0.41 | 1.08×10^-5^ | 2.57×10^-2^ | 22 | 22549484 |  |
| cg19335064 | 0.27 | 3.41×10^-6^ | 9.63×10^-3^ | 22 | 36586044 | *APOL4* |
| cg06424697 | 0.02 | 1.19×10^-5^ | 2.77×10^-2^ | 22 | 37915699 | *CARD10* |
| cg23106733 | -0.32 | 5.12×10^-10^ | 3.89×10^-6^ | 22 | 38325762 | *MICALL1* |
| cg10995503 | 0.11 | 1.89×10^-7^ | 8.17×10^-4^ | 22 | 42765727 | *LINC01315* |
| cg08762150 | 0.14 | 1.45×10^-5^ | 3.25×10^-2^ | 22 | 44644994 | *KIAA1644* |
| cg19621268 | 0.06 | 2.48×10^-8^ | 1.39×10^-4^ | 22 | 50709258 | *MAPK11* |
| **B cells** |  |  |  |  |  |  |
| cg25369015 | 0.22 | 2.87×10^-9^ | 1.56×10^-5^ | 1 | 9599256 | *SLC25A33* |
| cg02254548 | -1.00 | 3.62×10^-6^ | 8.99×10^-3^ | 1 | 15585958 | *FHAD1* |
| cg07973655 | 0.25 | 4.32×10^-6^ | 1.04×10^-2^ | 1 | 25566628 |  |
| cg02107269 | 0.08 | 1.38×10^-5^ | 2.96×10^-2^ | 1 | 27248138 | *NUDC* |
| cg15702478 | -0.91 | 5.14×10^-6^ | 1.20×10^-2^ | 1 | 32897864 |  |
| cg22369954 | -0.61 | 2.24×10^-6^ | 6.02×10^-3^ | 1 | 36184863 | *C1orf216* |
| cg03063365 | -1.00 | 2.70×10^-23^ | 1.33×10^-18^ | 1 | 36939085 | *CSF3R* |
| cg24323341 | 1.00 | 4.54×10^-16^ | 8.15×10^-12^ | 1 | 42501936 |  |
| cg12413474 | -0.63 | 1.83×10^-7^ | 7.09×10^-4^ | 1 | 45687697 |  |
| cg15738268 | 1.00 | 1.74×10^-5^ | 3.61×10^-2^ | 1 | 61439110 |  |
| cg02696670 | -0.67 | 6.60×10^-8^ | 2.84×10^-4^ | 1 | 64669384 | *UBE2U* |
| cg00010742 | 1.00 | 8.05×10^-7^ | 2.54×10^-3^ | 1 | 91182989 | *BARHL2* |
| cg02796548 | -0.54 | 7.16×10^-13^ | 7.87×10^-9^ | 1 | 92372096 | *TGFBR3* |
| cg03293269 | -0.70 | 6.41×10^-7^ | 2.11×10^-3^ | 1 | 93286299 |  |
| cg19083659 | -0.86 | 7.25×10^-12^ | 6.07×10^-8^ | 1 | 100877610 | *CDC14A* |
| cg13860179 | -0.47 | 9.75×10^-7^ | 2.93×10^-3^ | 1 | 113153660 | *ST7L* |
| cg21730450 | -0.65 | 4.19×10^-9^ | 2.19×10^-5^ | 1 | 116866482 |  |
| cg22872553 | -0.76 | 4.17×10^-16^ | 7.68×10^-12^ | 1 | 120216639 |  |
| cg16460669 | -0.82 | 3.96×10^-16^ | 7.68×10^-12^ | 1 | 199659497 |  |
| cg23258678 | 1.00 | 5.87×10^-8^ | 2.56×10^-4^ | 1 | 200004095 | *NR5A2* |
| cg19667051 | 0.49 | 1.08×10^-5^ | 2.37×10^-2^ | 1 | 207046933 |  |
| cg11004672 | -0.74 | 1.57×10^-5^ | 3.31×10^-2^ | 1 | 224939595 |  |
| cg04158884 | 1.00 | 4.24×10^-6^ | 1.02×10^-2^ | 1 | 233086346 | *C1orf57* |
| cg13360758 | -0.93 | 1.02×10^-6^ | 3.03×10^-3^ | 2 | 3467963 | *TRAPPC12* |
| cg07173100 | -0.60 | 2.47×10^-6^ | 6.46×10^-3^ | 2 | 3606140 | *RNASEH1* |
| cg08182009 | -0.96 | 5.22×10^-15^ | 7.69×10^-11^ | 2 | 33329231 | *LTBP1* |
| cg19454294 | 1.00 | 1.42×10^-7^ | 5.82×10^-4^ | 2 | 68466335 | *PPP3R1* |
| cg20030528 | -0.68 | 8.27×10^-6^ | 1.84×10^-2^ | 2 | 70138723 |  |
| cg25535468 | -0.92 | 1.61×10^-7^ | 6.44×10^-4^ | 2 | 98986314 | *CNGA3* |
| cg10155147 | -1.00 | 2.94×10^-12^ | 2.82×10^-8^ | 2 | 106500829 | *NCK2* |
| cg00940894 | -0.51 | 2.14×10^-7^ | 8.01×10^-4^ | 2 | 122018686 | *TFCP2L1* |
| cg18110444 | 0.85 | 2.50×10^-13^ | 2.88×10^-9^ | 2 | 136743460 | *DARS* |
| cg22290832 | -1.00 | 1.27×10^-6^ | 3.60×10^-3^ | 2 | 143171775 |  |
| cg08822497 | -1.00 | 8.79×10^-24^ | 4.63×10^-19^ | 2 | 158025305 |  |
| cg10115182 | 0.19 | 2.06×10^-5^ | 4.13×10^-2^ | 2 | 162164700 | *PSMD14* |
| cg10830649 | -0.44 | 6.46×10^-6^ | 1.48×10^-2^ | 2 | 166650933 | *GALNT3* |
| cg19648955 | -0.64 | 1.71×10^-10^ | 1.17×10^-6^ | 2 | 177134095 | *MTX2* |
| cg15133917 | 0.82 | 4.88×10^-6^ | 1.15×10^-2^ | 2 | 179672637 | *TTN* |
| cg21166445 | -0.84 | 2.22×10^-7^ | 8.28×10^-4^ | 2 | 181843895 | *UBE2E3* |
| cg00372801 | 0.57 | 7.81×10^-9^ | 3.86×10^-5^ | 2 | 191219050 | *INPP1* |
| cg24345062 | 1.00 | 2.32×10^-7^ | 8.55×10^-4^ | 2 | 200334439 | *FLJ32063* |
| cg10574213 | -1.00 | 2.86×10^-8^ | 1.29×10^-4^ | 2 | 208615379 | *CCNYL1* |
| cg01236747 | -0.64 | 2.94×10^-8^ | 1.32×10^-4^ | 2 | 208815027 | *PLEKHM3* |
| cg26551569 | 0.39 | 1.46×10^-5^ | 3.12×10^-2^ | 2 | 230873081 | *FBXO36* |
| cg14357719 | -1.00 | 1.41×10^-9^ | 7.96×10^-6^ | 2 | 236500777 | *AGAP1* |
| cg16801535 | 0.64 | 3.10×10^-7^ | 1.10×10^-3^ | 3 | 9291372 | *SRGAP3* |
| cg16512192 | -1.00 | 7.65×10^-12^ | 6.33×10^-8^ | 3 | 10805672 | *LINC00606* |
| cg21864304 | -1.00 | 4.03×10^-7^ | 1.40×10^-3^ | 3 | 11746722 | *VGLL4* |
| cg16020689 | -0.97 | 3.72×10^-10^ | 2.38×10^-6^ | 3 | 35299732 | *LOC101928135* |
| cg06926555 | -1.00 | 3.89×10^-6^ | 9.56×10^-3^ | 3 | 42700251 | *ZBTB47* |
| cg22754660 | 0.80 | 2.15×10^-6^ | 5.80×10^-3^ | 3 | 47462875 | *SCAP* |
| cg21194499 | 0.47 | 1.08×10^-8^ | 5.16×10^-5^ | 3 | 52188578 | *WDR51A* |
| cg04216480 | -0.80 | 6.19×10^-20^ | 2.53×10^-15^ | 3 | 60587837 | *FHIT* |
| cg04880248 | -0.66 | 2.53×10^-5^ | 4.93×10^-2^ | 3 | 121982083 | *CASR* |
| cg25714057 | -0.95 | 6.38×10^-10^ | 3.88×10^-6^ | 3 | 123139160 | *ADCY5* |
| cg06176270 | -1.00 | 4.80×10^-6^ | 1.13×10^-2^ | 3 | 127454802 | *MGLL* |
| cg08958945 | 0.60 | 2.42×10^-7^ | 8.83×10^-4^ | 3 | 133591690 | *RAB6B* |
| cg23232849 | -1.00 | 8.89×10^-7^ | 2.72×10^-3^ | 3 | 140817977 | *SPSB4* |
| cg27586272 | -0.88 | 1.75×10^-15^ | 2.81×10^-11^ | 3 | 148884929 | *HPS3* |
| cg12483503 | -0.95 | 6.81×10^-6^ | 1.55×10^-2^ | 3 | 152914700 |  |
| cg22068985 | -0.34 | 1.66×10^-5^ | 3.47×10^-2^ | 3 | 155571279 | *SLC33A1* |
| cg17503645 | -1.00 | 3.70×10^-7^ | 1.30×10^-3^ | 3 | 155837026 | *KCNAB1* |
| cg04738673 | -1.00 | 2.75×10^-10^ | 1.79×10^-6^ | 3 | 177482968 |  |
| cg13681385 | -0.88 | 2.68×10^-6^ | 6.93×10^-3^ | 3 | 183239414 | *KLHL6* |
| cg12942328 | 0.39 | 3.25×10^-6^ | 8.15×10^-3^ | 3 | 187455734 | *BCL6* |
| cg16653588 | -1.00 | 4.14×10^-19^ | 1.61×10^-14^ | 3 | 194298696 |  |
| cg04125205 | -1.00 | 7.35×10^-9^ | 3.66×10^-5^ | 4 | 649540 | *PDE6B* |
| cg03504160 | -1.00 | 7.34×10^-18^ | 2.00×10^-13^ | 4 | 6659194 |  |
| cg12237849 | -0.42 | 7.36×10^-7^ | 2.38×10^-3^ | 4 | 7014942 | *TBC1D14* |
| cg09599564 | -1.00 | 1.22×10^-5^ | 2.65×10^-2^ | 4 | 8472629 | *TRMT44* |
| cg00598021 | -1.00 | 1.69×10^-9^ | 9.30×10^-6^ | 4 | 10113794 | *WDR1* |
| cg01120514 | 0.73 | 2.20×10^-8^ | 1.02×10^-4^ | 4 | 25314414 | *ZCCHC4* |
| cg07520341 | 0.58 | 1.34×10^-6^ | 3.78×10^-3^ | 4 | 54132843 | *SCFD2* |
| cg13899293 | 0.93 | 1.76×10^-7^ | 6.88×10^-4^ | 4 | 79175365 | *FRAS1* |
| cg23528488 | -1.00 | 5.78×10^-11^ | 4.10×10^-7^ | 4 | 85818121 | *WDFY3* |
| cg10773082 | -0.48 | 2.37×10^-8^ | 1.09×10^-4^ | 4 | 100928575 | *LOC256880* |
| cg02886293 | 0.75 | 1.79×10^-5^ | 3.70×10^-2^ | 4 | 111462716 | *ENPEP* |
| cg11123177 | -0.84 | 1.11×10^-18^ | 4.08×10^-14^ | 4 | 122063045 | *TNIP3* |
| cg19702777 | 1.00 | 3.58×10^-9^ | 1.92×10^-5^ | 4 | 150474366 |  |
| cg17318769 | -1.00 | 4.17×10^-12^ | 3.75×10^-8^ | 4 | 154229091 | *TRIM2* |
| cg11383226 | -1.00 | 4.84×10^-9^ | 2.49×10^-5^ | 4 | 159236616 |  |
| cg09029959 | 1.00 | 1.62×10^-5^ | 3.41×10^-2^ | 4 | 163905924 |  |
| cg26511502 | 0.21 | 1.70×10^-5^ | 3.54×10^-2^ | 4 | 186125372 | *CFAP97* |
| cg25744610 | -0.85 | 1.26×10^-7^ | 5.22×10^-4^ | 4 | 189896614 |  |
| cg20310920 | -0.75 | 2.19×10^-5^ | 4.39×10^-2^ | 5 | 370421 | *AHRR* |
| cg11498652 | 1.00 | 1.79×10^-9^ | 9.79×10^-6^ | 5 | 40834691 | *RPL37* |
| cg12893351 | -1.00 | 2.53×10^-7^ | 9.11×10^-4^ | 5 | 43268644 | *NIM1K* |
| cg01506293 | -1.00 | 5.11×10^-10^ | 3.22×10^-6^ | 5 | 54054033 |  |
| cg04480129 | -1.00 | 9.50×10^-8^ | 4.00×10^-4^ | 5 | 66316631 | *MAST4* |
| cg10435952 | -1.00 | 8.62×10^-12^ | 6.91×10^-8^ | 5 | 81884312 |  |
| cg21878859 | 0.55 | 2.69×10^-6^ | 6.93×10^-3^ | 5 | 95767724 | *PCSK1* |
| cg18840956 | 0.59 | 1.13×10^-5^ | 2.46×10^-2^ | 5 | 95769005 | *PCSK1* |
| cg25511332 | -0.55 | 1.25×10^-5^ | 2.71×10^-2^ | 5 | 113391339 |  |
| cg26201826 | 0.75 | 5.76×10^-8^ | 2.53×10^-4^ | 5 | 114598579 | *PGGT1B* |
| cg18058352 | -0.91 | 2.20×10^-12^ | 2.19×10^-8^ | 5 | 127675527 | *FBN2* |
| cg23165500 | -0.69 | 2.60×10^-8^ | 1.19×10^-4^ | 5 | 152949095 | *GRIA1* |
| cg07682547 | 0.98 | 1.41×10^-13^ | 1.67×10^-9^ | 5 | 153863417 |  |
| cg20548393 | -0.47 | 3.45×10^-7^ | 1.22×10^-3^ | 5 | 156934039 | *ADAM19* |
| cg17613241 | -0.92 | 1.70×10^-11^ | 1.31×10^-7^ | 5 | 167069972 | *TENM2* |
| cg01883164 | -1.00 | 1.28×10^-24^ | 7.85×10^-20^ | 5 | 167533108 | *TENM2* |
| cg15998629 | -1.00 | 1.27×10^-9^ | 7.17×10^-6^ | 5 | 172000858 |  |
| cg06974839 | 0.29 | 5.34×10^-7^ | 1.78×10^-3^ | 5 | 177019233 | *TMED9* |
| cg03730027 | -0.96 | 3.84×10^-14^ | 4.80×10^-10^ | 5 | 180041908 | *FLT4* |
| cg12930304 | 1.00 | 4.65×10^-17^ | 1.07×10^-12^ | 6 | 2818991 |  |
| cg11809418 | -0.60 | 1.73×10^-7^ | 6.81×10^-4^ | 6 | 8889063 |  |
| cg08571475 | -1.00 | 5.51×10^-18^ | 1.56×10^-13^ | 6 | 11494909 |  |
| cg09838562 | 0.21 | 9.98×10^-6^ | 2.21×10^-2^ | 6 | 15248173 | *JARID2* |
| cg20284834 | -1.00 | 1.01×10^-33^ | 2.48×10^-28^ | 6 | 15499615 | *JARID2* |
| cg27250759 | 0.29 | 2.61×10^-8^ | 1.19×10^-4^ | 6 | 20401608 | *E2F3* |
| cg02147727 | -0.71 | 2.95×10^-6^ | 7.49×10^-3^ | 6 | 20500125 |  |
| cg02565494 | -1.00 | 1.16×10^-9^ | 6.62×10^-6^ | 6 | 25287859 | *LRRC16A* |
| cg04139791 | -0.87 | 2.06×10^-5^ | 4.13×10^-2^ | 6 | 25456463 | *LRRC16A* |
| cg18281744 | -0.74 | 4.28×10^-8^ | 1.91×10^-4^ | 6 | 29455512 | *MAS1L* |
| cg10750989 | -0.29 | 2.50×10^-6^ | 6.47×10^-3^ | 6 | 31371330 | *MICA* |
| cg05742293 | -0.54 | 9.28×10^-7^ | 2.81×10^-3^ | 6 | 33374195 | *KIFC1* |
| cg15689154 | 0.54 | 2.04×10^-5^ | 4.12×10^-2^ | 6 | 37137227 | *PIM1* |
| cg08864240 | -0.48 | 1.24×10^-7^ | 5.14×10^-4^ | 6 | 39895717 | *MOCS1* |
| cg14925155 | -0.60 | 2.36×10^-7^ | 8.64×10^-4^ | 6 | 42195146 | *TRERF1* |
| cg20102583 | 0.69 | 2.71×10^-12^ | 2.66×10^-8^ | 6 | 44171055 |  |
| cg19254793 | -1.00 | 3.15×10^-11^ | 2.35×10^-7^ | 6 | 44695348 |  |
| cg11517630 | -0.61 | 1.92×10^-7^ | 7.32×10^-4^ | 6 | 72318447 |  |
| cg12267800 | 0.72 | 1.98×10^-6^ | 5.39×10^-3^ | 6 | 106240391 |  |
| cg17388996 | 0.57 | 3.88×10^-11^ | 2.83×10^-7^ | 6 | 108145374 | *SCML4* |
| cg15406010 | 0.99 | 6.31×10^-9^ | 3.16×10^-5^ | 6 | 108478650 |  |
| cg04591119 | 0.58 | 9.77×10^-7^ | 2.93×10^-3^ | 6 | 111197245 | *AMD1* |
| cg16155724 | -1.00 | 1.51×10^-18^ | 4.84×10^-14^ | 6 | 112102807 | *FYN* |
| cg09035093 | -0.57 | 2.61×10^-7^ | 9.35×10^-4^ | 6 | 132181821 | *ENPP1* |
| cg26112901 | 0.42 | 1.74×10^-6^ | 4.79×10^-3^ | 6 | 137494988 | *IL22RA2* |
| cg05905568 | -1.00 | 7.75×10^-7^ | 2.47×10^-3^ | 6 | 139453359 |  |
| cg07488161 | -1.00 | 8.82×10^-7^ | 2.72×10^-3^ | 6 | 169845176 |  |
| cg01368780 | -0.50 | 7.52×10^-7^ | 2.42×10^-3^ | 6 | 169937196 | *WDR27* |
| cg12407882 | -0.89 | 6.49×10^-7^ | 2.13×10^-3^ | 7 | 205759 | *FAM20C* |
| cg25668117 | -1.00 | 1.41×10^-8^ | 6.67×10^-5^ | 7 | 959904 | *ADAP1* |
| cg21998840 | -1.00 | 1.52×10^-17^ | 3.74×10^-13^ | 7 | 2212030 | *MAD1L1* |
| cg03088413 | -1.00 | 1.03×10^-6^ | 3.05×10^-3^ | 7 | 6525016 | *KDELR2* |
| cg21503490 | -0.64 | 6.63×10^-14^ | 8.01×10^-10^ | 7 | 6773787 | *PMS2CL* |
| cg09847222 | 0.66 | 1.53×10^-5^ | 3.25×10^-2^ | 7 | 47348487 | *TNS3* |
| cg00611847 | -0.71 | 5.92×10^-6^ | 1.37×10^-2^ | 7 | 50599369 | *DDC-AS1* |
| cg12029626 | -0.40 | 1.82×10^-5^ | 3.74×10^-2^ | 7 | 65216224 | *CCT6P1* |
| cg03242877 | 0.11 | 7.29×10^-6^ | 1.65×10^-2^ | 7 | 65540778 | *ASL* |
| cg27528426 | 0.29 | 2.60×10^-13^ | 2.94×10^-9^ | 7 | 65540851 | *ASL* |
| cg14453392 | -0.89 | 1.17×10^-5^ | 2.55×10^-2^ | 7 | 69899745 | *AUTS2* |
| cg16639595 | -0.48 | 1.17×10^-6^ | 3.42×10^-3^ | 7 | 87856984 | *SRI* |
| cg01602252 | -0.96 | 5.33×10^-6^ | 1.24×10^-2^ | 7 | 95959891 |  |
| cg10256052 | 0.10 | 4.56×10^-7^ | 1.56×10^-3^ | 7 | 99036534 | *CPSF4* |
| cg15703422 | -0.97 | 5.66×10^-16^ | 9.93×10^-12^ | 7 | 100176855 | *LRCH4* |
| cg27084965 | 0.43 | 1.96×10^-7^ | 7.44×10^-4^ | 7 | 103871917 |  |
| cg26095262 | 0.54 | 7.59×10^-6^ | 1.70×10^-2^ | 7 | 106620404 |  |
| cg19620668 | 0.76 | 7.55×10^-7^ | 2.42×10^-3^ | 7 | 129214879 |  |
| cg07683330 | 0.74 | 2.02×10^-7^ | 7.65×10^-4^ | 7 | 130125390 | *MEST* |
| cg08011768 | 0.70 | 1.27×10^-6^ | 3.60×10^-3^ | 7 | 131241256 | *PODXL* |
| cg21793382 | -1.00 | 1.51×10^-18^ | 4.84×10^-14^ | 7 | 134379148 |  |
| cg07499066 | -0.75 | 1.13×10^-14^ | 1.55×10^-10^ | 7 | 149171379 | *ZNF746* |
| cg23371350 | -0.51 | 4.65×10^-6^ | 1.10×10^-2^ | 7 | 149416674 | *KRBA1* |
| cg14141610 | -1.00 | 1.08×10^-9^ | 6.30×10^-6^ | 7 | 150099648 |  |
| cg05470643 | 1.00 | 2.44×10^-11^ | 1.85×10^-7^ | 7 | 155579830 |  |
| cg00827082 | -0.61 | 2.32×10^-6^ | 6.17×10^-3^ | 8 | 407482 | *FBXO25* |
| cg15640339 | -1.00 | 4.11×10^-11^ | 2.97×10^-7^ | 8 | 2017508 | *MYOM2* |
| cg23332189 | -0.72 | 4.88×10^-7^ | 1.65×10^-3^ | 8 | 16477058 |  |
| cg21341889 | -0.94 | 9.62×10^-17^ | 2.02×10^-12^ | 8 | 17773367 |  |
| cg13646041 | -0.95 | 2.02×10^-6^ | 5.47×10^-3^ | 8 | 19103595 | *LOC100128993* |
| cg23523780 | -0.97 | 3.04×10^-28^ | 2.24×10^-23^ | 8 | 22972234 | *TNFRSF10C* |
| cg19134399 | -1.00 | 1.84×10^-6^ | 5.02×10^-3^ | 8 | 41403507 | *LOC102723729* |
| cg12148515 | -1.00 | 4.87×10^-15^ | 7.32×10^-11^ | 8 | 41517301 | *ANK1* |
| cg04596499 | 0.79 | 5.94×10^-8^ | 2.58×10^-4^ | 8 | 42213370 | *POLB* |
| cg07370726 | -1.00 | 2.41×10^-6^ | 6.37×10^-3^ | 8 | 47980380 |  |
| cg14694176 | 0.28 | 6.95×10^-6^ | 1.58×10^-2^ | 8 | 54935016 | *TCEA1* |
| cg25356825 | 1.00 | 8.02×10^-12^ | 6.57×10^-8^ | 8 | 59988692 | *TOX* |
| cg04742453 | -0.62 | 2.25×10^-6^ | 6.02×10^-3^ | 8 | 61685545 | *CHD7* |
| cg02934300 | -0.96 | 2.06×10^-7^ | 7.76×10^-4^ | 8 | 67317506 |  |
| cg17380533 | -0.57 | 8.67×10^-9^ | 4.20×10^-5^ | 8 | 72875102 | *MSC-AS1* |
| cg07028161 | 0.45 | 4.63×10^-6^ | 1.10×10^-2^ | 8 | 80720093 | *LOC101927040* |
| cg00923443 | -0.77 | 2.77×10^-6^ | 7.09×10^-3^ | 8 | 120449749 |  |
| cg07229027 | 1.00 | 1.17×10^-28^ | 1.08×10^-23^ | 8 | 124408743 | *ATAD2* |
| cg15171273 | -0.67 | 3.38×10^-12^ | 3.20×10^-8^ | 8 | 131539725 |  |
| cg02526790 | -1.00 | 1.16×10^-12^ | 1.24×10^-8^ | 8 | 134044590 | *TG* |
| cg15463309 | 1.00 | 2.51×10^-5^ | 4.92×10^-2^ | 8 | 141598157 | *EIF2C2* |
| cg16177887 | 0.20 | 2.25×10^-6^ | 6.02×10^-3^ | 8 | 145151087 | *CYC1* |
| cg03195539 | -1.00 | 1.45×10^-7^ | 5.92×10^-4^ | 9 | 16236971 | *C9orf92* |
| cg03785827 | 0.40 | 2.25×10^-5^ | 4.48×10^-2^ | 9 | 16423719 | *BNC2* |
| cg11230847 | 0.07 | 8.36×10^-7^ | 2.61×10^-3^ | 9 | 26947505 | *PLAA* |
| cg25958241 | -1.00 | 7.34×10^-6^ | 1.66×10^-2^ | 9 | 35278307 | *UNC13B* |
| cg14185994 | 0.37 | 7.91×10^-8^ | 3.37×10^-4^ | 9 | 98637332 | *C9orf130* |
| cg06853088 | 1.00 | 5.62×10^-12^ | 4.87×10^-8^ | 9 | 109040403 |  |
| cg14571425 | -1.00 | 1.78×10^-14^ | 2.38×10^-10^ | 9 | 109494762 |  |
| cg14004271 | -0.58 | 9.69×10^-9^ | 4.67×10^-5^ | 9 | 115650248 | *SLC46A2* |
| cg15221416 | -1.00 | 5.60×10^-17^ | 1.25×10^-12^ | 9 | 125021726 | *RBM18* |
| cg17618153 | -1.00 | 1.07×10^-31^ | 1.97×10^-26^ | 9 | 129874991 | *ANGPTL2* |
| cg13429719 | -0.90 | 1.51×10^-7^ | 6.09×10^-4^ | 9 | 130327532 | *FAM129B* |
| cg02812124 | -0.70 | 4.17×10^-9^ | 2.19×10^-5^ | 9 | 131553285 | *TBC1D13* |
| cg13803765 | -0.63 | 2.52×10^-5^ | 4.92×10^-2^ | 9 | 133760464 | *ABL1* |
| cg24981837 | -1.00 | 5.68×10^-11^ | 4.06×10^-7^ | 10 | 2113283 |  |
| cg05686843 | 1.00 | 2.96×10^-13^ | 3.31×10^-9^ | 10 | 6111408 |  |
| cg10197664 | -0.90 | 2.69×10^-7^ | 9.57×10^-4^ | 10 | 7984711 | *TAF3* |
| cg27392773 | 0.73 | 2.48×10^-6^ | 6.46×10^-3^ | 10 | 10994094 | *LINC00710* |
| cg08875451 | -0.40 | 1.16×10^-5^ | 2.52×10^-2^ | 10 | 11322268 | *CUGBP2* |
| cg15532545 | -0.82 | 2.16×10^-18^ | 6.64×10^-14^ | 10 | 14825999 |  |
| cg07659571 | -0.45 | 4.22×10^-6^ | 1.02×10^-2^ | 10 | 15839446 | *FAM188A* |
| cg24618739 | -1.00 | 2.41×10^-10^ | 1.60×10^-6^ | 10 | 25154684 | *PRTFDC1* |
| cg12622378 | -0.48 | 7.38×10^-6^ | 1.66×10^-2^ | 10 | 33552748 | *NRP1* |
| cg14196028 | 1.00 | 3.36×10^-29^ | 4.12×10^-24^ | 10 | 69523483 |  |
| cg01390840 | -0.86 | 2.66×10^-15^ | 4.17×10^-11^ | 10 | 76067347 | *ADK* |
| cg06993191 | -0.73 | 1.25×10^-15^ | 2.10×10^-11^ | 10 | 76514031 |  |
| cg19130698 | 0.39 | 1.53×10^-5^ | 3.25×10^-2^ | 10 | 91011807 | *LIPA* |
| cg10756593 | -1.00 | 2.27×10^-16^ | 4.51×10^-12^ | 10 | 104591244 | *CYP17A1* |
| cg05237489 | -0.58 | 3.76×10^-6^ | 9.27×10^-3^ | 10 | 108623805 | *SORCS1* |
| cg16550453 | 0.81 | 8.14×10^-6^ | 1.82×10^-2^ | 10 | 115939018 | *TDRD1* |
| cg04465599 | 0.10 | 6.07×10^-9^ | 3.07×10^-5^ | 10 | 121652136 | *SEC23IP* |
| cg25239156 | -1.00 | 4.51×10^-12^ | 3.96×10^-8^ | 10 | 131566948 |  |
| cg18615506 | -0.68 | 8.65×10^-17^ | 1.88×10^-12^ | 10 | 134177612 | *LRRC27* |
| cg19100464 | -0.77 | 1.05×10^-8^ | 5.02×10^-5^ | 10 | 134861410 |  |
| cg16674116 | -1.00 | 4.51×10^-12^ | 3.96×10^-8^ | 11 | 1319689 | *TOLLIP* |
| cg06080571 | -1.00 | 2.99×10^-25^ | 2.00×10^-20^ | 11 | 2349807 |  |
| cg26344859 | -1.00 | 3.84×10^-9^ | 2.05×10^-5^ | 11 | 2584629 | *KCNQ1* |
| cg03085335 | -1.00 | 1.28×10^-7^ | 5.27×10^-4^ | 11 | 2747204 | *KCNQ1* |
| cg00055860 | -0.98 | 1.11×10^-14^ | 1.54×10^-10^ | 11 | 5951424 |  |
| cg01494614 | 1.00 | 5.84×10^-15^ | 8.43×10^-11^ | 11 | 10562837 | *RNF141* |
| cg01385795 | 0.45 | 3.15×10^-6^ | 7.93×10^-3^ | 11 | 14913124 | *CYP2R1* |
| cg05454237 | 0.28 | 3.94×10^-6^ | 9.62×10^-3^ | 11 | 14913849 | *CYP2R1* |
| cg16802027 | 0.49 | 6.97×10^-7^ | 2.26×10^-3^ | 11 | 14913860 | *CYP2R1* |
| cg03544918 | 0.35 | 6.94×10^-10^ | 4.19×10^-6^ | 11 | 14913944 | *CYP2R1* |
| cg16544887 | 0.37 | 1.39×10^-12^ | 1.45×10^-8^ | 11 | 14913979 | *CYP2R1* |
| cg00455178 | 0.42 | 5.42×10^-18^ | 1.56×10^-13^ | 11 | 14913981 | *CYP2R1* |
| cg11980188 | 0.41 | 3.11×10^-14^ | 4.03×10^-10^ | 11 | 14914012 | *CYP2R1* |
| cg07731639 | -0.74 | 2.35×10^-5^ | 4.65×10^-2^ | 11 | 31437871 | *DNAJC24* |
| cg05400075 | -1.00 | 2.53×10^-7^ | 9.11×10^-4^ | 11 | 36144887 | *LDLRAD3* |
| cg12652180 | -0.95 | 6.13×10^-7^ | 2.02×10^-3^ | 11 | 45929820 | *C11orf94* |
| cg09592219 | -1.00 | 1.50×10^-5^ | 3.21×10^-2^ | 11 | 47997690 |  |
| cg21319427 | 0.88 | 3.94×10^-6^ | 9.62×10^-3^ | 11 | 60511143 | *MS4A18* |
| cg14228873 | -1.00 | 9.41×10^-7^ | 2.84×10^-3^ | 11 | 61547669 | *MYRF* |
| cg16083168 | -1.00 | 1.85×10^-7^ | 7.11×10^-4^ | 11 | 75678837 | *UVRAG* |
| cg16663931 | 0.97 | 4.94×10^-7^ | 1.66×10^-3^ | 11 | 75882654 |  |
| cg19815354 | -1.00 | 2.44×10^-5^ | 4.79×10^-2^ | 11 | 77450740 | *RSF1* |
| cg05962325 | 0.99 | 1.86×10^-8^ | 8.68×10^-5^ | 11 | 107992005 | *ACAT1* |
| cg13186466 | 1.00 | 5.91×10^-10^ | 3.63×10^-6^ | 11 | 111957596 | *TIMM8B* |
| cg13485335 | -1.00 | 4.16×10^-14^ | 5.11×10^-10^ | 11 | 120161548 | *POU2F3* |
| cg13607511 | 0.13 | 4.33×10^-6^ | 1.04×10^-2^ | 11 | 125439223 | *EI24* |
| cg05297666 | -0.45 | 4.14×10^-6^ | 1.01×10^-2^ | 11 | 128400310 | *ETS1* |
| cg06072950 | -0.46 | 5.23×10^-8^ | 2.31×10^-4^ | 11 | 129736336 | *NFRKB* |
| cg11550340 | -0.77 | 2.07×10^-10^ | 1.39×10^-6^ | 12 | 3217332 | *TSPAN9* |
| cg22237777 | 0.32 | 6.27×10^-6^ | 1.45×10^-2^ | 12 | 6602920 | *MRPL51* |
| cg04636406 | -0.67 | 4.80×10^-23^ | 2.21×10^-18^ | 12 | 6993009 | *RPL13P5* |
| cg10201141 | -1.00 | 1.98×10^-10^ | 1.34×10^-6^ | 12 | 7032793 | *ENO2* |
| cg10873203 | -0.67 | 3.96×10^-12^ | 3.69×10^-8^ | 12 | 25942772 |  |
| cg18287149 | 0.10 | 1.82×10^-5^ | 3.74×10^-2^ | 12 | 29301794 | *FAR2* |
| cg06405045 | 0.90 | 1.32×10^-17^ | 3.35×10^-13^ | 12 | 31479141 | *FAM60A* |
| cg25598488 | -0.97 | 7.38×10^-8^ | 3.16×10^-4^ | 12 | 34258505 |  |
| cg22418434 | -1.00 | 9.25×10^-7^ | 2.81×10^-3^ | 12 | 34259653 |  |
| cg18062384 | 0.40 | 2.56×10^-5^ | 4.96×10^-2^ | 12 | 49764345 | *SPATS2* |
| cg19963684 | -0.47 | 3.50×10^-6^ | 8.73×10^-3^ | 12 | 94508273 |  |
| cg15505491 | 1.00 | 1.69×10^-9^ | 9.30×10^-6^ | 12 | 101127597 |  |
| cg22874494 | 0.69 | 1.57×10^-5^ | 3.31×10^-2^ | 12 | 105381266 | *C12orf45* |
| cg11771936 | -0.78 | 3.84×10^-7^ | 1.34×10^-3^ | 12 | 120602003 | *GCN1* |
| cg02142767 | 0.21 | 1.18×10^-6^ | 3.42×10^-3^ | 12 | 125348525 | *SCARB1* |
| cg15244603 | -1.00 | 5.75×10^-31^ | 8.48×10^-26^ | 12 | 125607520 | *AACS* |
| cg10788674 | 0.13 | 1.34×10^-6^ | 3.78×10^-3^ | 12 | 133214645 | *POLE* |
| cg08197826 | -1.00 | 3.25×10^-14^ | 4.12×10^-10^ | 13 | 19240901 |  |
| cg16708680 | 0.61 | 8.08×10^-7^ | 2.55×10^-3^ | 13 | 30996972 | *LINC01058* |
| cg10362742 | -0.85 | 4.08×10^-12^ | 3.73×10^-8^ | 13 | 31736223 | *HSPH1* |
| cg03138282 | -0.72 | 2.04×10^-5^ | 4.12×10^-2^ | 13 | 39187482 |  |
| cg07818646 | 0.13 | 2.25×10^-5^ | 4.48×10^-2^ | 13 | 51483769 | *RNASEH2B* |
| cg20814935 | -0.49 | 4.31×10^-6^ | 1.04×10^-2^ | 13 | 60743824 |  |
| cg08762237 | 1.00 | 3.69×10^-6^ | 9.13×10^-3^ | 13 | 78667308 | *RNF219-AS1* |
| cg12144451 | -0.57 | 2.29×10^-5^ | 4.54×10^-2^ | 13 | 94767766 | *GPC6* |
| cg23235217 | 0.23 | 2.29×10^-7^ | 8.46×10^-4^ | 13 | 100741026 | *PCCA* |
| cg01552748 | 0.38 | 1.94×10^-5^ | 3.95×10^-2^ | 13 | 112225526 |  |
| cg00902895 | -0.79 | 2.56×10^-5^ | 4.96×10^-2^ | 13 | 113425756 | *ATP11A* |
| cg08508348 | -1.00 | 9.61×10^-11^ | 6.68×10^-7^ | 13 | 114862793 | *RASA3* |
| cg17864209 | -0.98 | 2.16×10^-12^ | 2.18×10^-8^ | 14 | 35805102 |  |
| cg09529835 | -1.00 | 1.25×10^-6^ | 3.57×10^-3^ | 14 | 36491342 |  |
| cg06243610 | -1.00 | 8.24×10^-29^ | 8.68×10^-24^ | 14 | 52373839 | *GNG2* |
| cg08606441 | 0.87 | 5.46×10^-7^ | 1.81×10^-3^ | 14 | 55142748 | *SAMD4A* |
| cg00100184 | 0.48 | 1.22×10^-6^ | 3.53×10^-3^ | 14 | 56014589 |  |
| cg15912778 | -0.87 | 4.73×10^-7^ | 1.61×10^-3^ | 14 | 74727333 | *VSX2* |
| cg01680773 | 0.48 | 4.63×10^-9^ | 2.40×10^-5^ | 14 | 74960124 | *NPC2* |
| cg10112231 | -1.00 | 1.84×10^-7^ | 7.09×10^-4^ | 14 | 77774990 | *POMT2* |
| cg19953646 | 1.00 | 3.04×10^-6^ | 7.71×10^-3^ | 14 | 86000942 | *FLRT2* |
| cg14519717 | -0.81 | 5.71×10^-12^ | 4.89×10^-8^ | 14 | 90744790 | *C14orf102* |
| cg08015762 | -0.56 | 1.23×10^-6^ | 3.55×10^-3^ | 14 | 100014785 | *CCDC85C* |
| cg10170654 | 0.47 | 1.79×10^-5^ | 3.70×10^-2^ | 14 | 104152238 | *KLC1* |
| cg07709681 | 0.42 | 1.64×10^-8^ | 7.70×10^-5^ | 15 | 34393712 | *PGBD4* |
| cg24245086 | -1.00 | 1.37×10^-16^ | 2.81×10^-12^ | 15 | 44164534 | *FRMD5* |
| cg27198899 | -1.00 | 3.60×10^-11^ | 2.65×10^-7^ | 15 | 51197587 |  |
| cg22134372 | 0.55 | 8.63×10^-10^ | 5.13×10^-6^ | 15 | 52455211 | *GNB5* |
| cg07947039 | -1.00 | 7.36×10^-10^ | 4.41×10^-6^ | 15 | 56292422 |  |
| cg13361868 | -1.00 | 9.02×10^-10^ | 5.32×10^-6^ | 15 | 72610659 | *CELF6* |
| cg06364961 | -1.00 | 4.76×10^-10^ | 3.03×10^-6^ | 15 | 74490113 | *STRA6* |
| cg01413612 | 0.36 | 8.60×10^-6^ | 1.91×10^-2^ | 15 | 76351413 | *C15orf27* |
| cg05477921 | -0.60 | 2.43×10^-7^ | 8.83×10^-4^ | 15 | 100685723 | *ADAMTS17* |
| cg07967632 | -0.38 | 2.40×10^-6^ | 6.37×10^-3^ | 15 | 101601131 | *LRRK1* |
| cg08681117 | -0.87 | 2.46×10^-6^ | 6.45×10^-3^ | 16 | 1179798 |  |
| cg16784970 | -0.62 | 1.50×10^-9^ | 8.39×10^-6^ | 16 | 2123215 | *TSC2* |
| cg02560615 | 0.55 | 1.64×10^-10^ | 1.13×10^-6^ | 16 | 2274210 | *E4F1* |
| cg14939446 | 1.00 | 1.97×10^-44^ | 7.24×10^-39^ | 16 | 2571015 | *AMDHD2* |
| cg08392784 | -0.51 | 1.83×10^-6^ | 5.01×10^-3^ | 16 | 3546517 | *C16orf90* |
| cg00655143 | -0.86 | 2.53×10^-11^ | 1.90×10^-7^ | 16 | 3985958 |  |
| cg00730182 | -0.49 | 1.99×10^-5^ | 4.04×10^-2^ | 16 | 12161636 | *SNX29* |
| cg02196379 | -0.74 | 2.68×10^-10^ | 1.76×10^-6^ | 16 | 16215875 | *ABCC1* |
| cg20438949 | 0.26 | 4.21×10^-7^ | 1.46×10^-3^ | 16 | 28834126 | *ATXN2L* |
| cg18129863 | 1.00 | 1.08×10^-17^ | 2.83×10^-13^ | 16 | 30886426 | *MIR762HG* |
| cg27067771 | -0.92 | 2.40×10^-5^ | 4.72×10^-2^ | 16 | 34981394 | *FLJ26245* |
| cg17606327 | -1.00 | 3.28×10^-10^ | 2.12×10^-6^ | 16 | 34987165 | *FLJ26245* |
| cg01122672 | 0.16 | 6.86×10^-6^ | 1.56×10^-2^ | 16 | 47178067 | *NETO2* |
| cg16798669 | -1.00 | 2.94×10^-12^ | 2.82×10^-8^ | 16 | 50658973 | *NKD1* |
| cg27453761 | -0.39 | 2.76×10^-6^ | 7.08×10^-3^ | 16 | 66624346 |  |
| cg12247787 | -0.71 | 6.59×10^-7^ | 2.15×10^-3^ | 16 | 67165711 | *C16orf70* |
| cg02727363 | 0.18 | 1.35×10^-5^ | 2.91×10^-2^ | 16 | 70284928 | *EXOSC6* |
| cg16053428 | -0.39 | 2.46×10^-6^ | 6.45×10^-3^ | 16 | 83990544 | *OSGIN1* |
| cg10274403 | -0.80 | 1.29×10^-11^ | 1.00×10^-7^ | 16 | 84178812 | *LRRC50* |
| cg14029669 | -0.50 | 1.68×10^-7^ | 6.71×10^-4^ | 16 | 84330741 | *WFDC1* |
| cg16455798 | -1.00 | 8.85×10^-7^ | 2.72×10^-3^ | 16 | 85083652 | *KIAA0513* |
| cg05262224 | 0.48 | 3.62×10^-6^ | 8.99×10^-3^ | 16 | 86482459 |  |
| cg10810352 | 0.20 | 3.96×10^-9^ | 2.10×10^-5^ | 16 | 87417434 | *FBXO31* |
| cg10499753 | -1.00 | 1.54×10^-7^ | 6.21×10^-4^ | 16 | 89654085 | *CPNE7* |
| cg21507719 | -1.00 | 8.38×10^-11^ | 5.88×10^-7^ | 17 | 7256673 | *KCTD11* |
| cg20072901 | -0.96 | 9.11×10^-8^ | 3.86×10^-4^ | 17 | 10563216 |  |
| cg17235374 | 0.97 | 4.81×10^-8^ | 2.13×10^-4^ | 17 | 27717757 | *TAOK1* |
| cg15487375 | -0.84 | 6.61×10^-12^ | 5.60×10^-8^ | 17 | 38105237 |  |
| cg23058170 | -1.00 | 5.85×10^-6^ | 1.36×10^-2^ | 17 | 41165176 | *IFI35* |
| cg10420015 | -1.00 | 1.32×10^-18^ | 4.64×10^-14^ | 17 | 43116171 | *DCAKD* |
| cg02859521 | -0.81 | 2.19×10^-17^ | 5.20×10^-13^ | 17 | 43332672 | *MAP3K14-AS1* |
| cg14295458 | -0.96 | 1.62×10^-21^ | 7.03×10^-17^ | 17 | 45891164 | *OSBPL7* |
| cg20672426 | 0.49 | 1.35×10^-6^ | 3.78×10^-3^ | 17 | 53851054 | *PCTP* |
| cg17182054 | 0.48 | 1.16×10^-6^ | 3.40×10^-3^ | 17 | 54588075 |  |
| cg19169246 | 0.33 | 2.90×10^-6^ | 7.38×10^-3^ | 17 | 73887208 | *TRIM65* |
| cg05585141 | 0.24 | 1.10×10^-6^ | 3.23×10^-3^ | 17 | 75231847 |  |
| cg11988568 | -0.79 | 1.06×10^-11^ | 8.43×10^-8^ | 17 | 78518917 | *RPTOR* |
| cg22882093 | -0.77 | 5.98×10^-9^ | 3.04×10^-5^ | 17 | 80158055 | *CCDC57* |
| cg07431961 | -1.00 | 4.10×10^-12^ | 3.73×10^-8^ | 17 | 80902556 | *B3GNTL1* |
| cg16717605 | -0.89 | 1.12×10^-6^ | 3.30×10^-3^ | 18 | 691284 | *ENOSF1* |
| cg09288233 | -0.72 | 1.14×10^-9^ | 6.56×10^-6^ | 18 | 3449455 | *TGIF1* |
| cg18208602 | -0.86 | 2.28×10^-13^ | 2.67×10^-9^ | 18 | 10488229 | *APCDD1* |
| cg13373376 | -1.00 | 1.70×10^-5^ | 3.54×10^-2^ | 18 | 13182069 |  |
| cg02565102 | -0.52 | 4.45×10^-7^ | 1.53×10^-3^ | 18 | 20406876 |  |
| cg06878786 | -0.90 | 1.38×10^-12^ | 1.45×10^-8^ | 18 | 21693037 | *TTC39C* |
| cg14389183 | 1.00 | 1.89×10^-5^ | 3.86×10^-2^ | 18 | 47808321 | *MBD1* |
| cg05616303 | -1.00 | 1.24×10^-6^ | 3.55×10^-3^ | 18 | 65321470 | *LOC643542* |
| cg08117750 | -1.00 | 1.54×10^-6^ | 4.28×10^-3^ | 18 | 71904767 |  |
| cg25185915 | -0.75 | 1.88×10^-14^ | 2.47×10^-10^ | 18 | 74507281 | *LOC100131655* |
| cg05535853 | -0.90 | 7.22×10^-15^ | 1.02×10^-10^ | 18 | 74781719 | *MBP* |
| cg06168021 | -0.94 | 1.12×10^-9^ | 6.51×10^-6^ | 18 | 75399320 |  |
| cg14700108 | -1.00 | 1.38×10^-15^ | 2.26×10^-11^ | 19 | 2637722 | *GNG7* |
| cg06384607 | -0.79 | 4.35×10^-6^ | 1.04×10^-2^ | 19 | 3603453 | *TBXA2R* |
| cg22933199 | 0.82 | 5.32×10^-7^ | 1.78×10^-3^ | 19 | 12163909 | *ZNF878* |
| cg22930196 | -1.00 | 1.11×10^-5^ | 2.43×10^-2^ | 19 | 14937167 | *OR7A5* |
| cg18025793 | -0.98 | 8.01×10^-13^ | 8.68×10^-9^ | 19 | 17097121 | *CPAMD8* |
| cg23397718 | -1.00 | 3.09×10^-6^ | 7.81×10^-3^ | 19 | 18630997 | *ELL* |
| cg14387983 | 0.24 | 1.36×10^-6^ | 3.78×10^-3^ | 19 | 33865280 | *CEBPG* |
| cg23390269 | -0.50 | 1.71×10^-6^ | 4.72×10^-3^ | 19 | 34371262 |  |
| cg23026246 | 0.57 | 1.23×10^-11^ | 9.67×10^-8^ | 19 | 41029387 | *SPTBN4* |
| cg05269058 | -0.89 | 3.08×10^-15^ | 4.73×10^-11^ | 19 | 45285916 | *CBLC* |
| cg01009059 | -0.65 | 1.54×10^-12^ | 1.57×10^-8^ | 19 | 46391024 |  |
| cg09407049 | 0.38 | 1.04×10^-6^ | 3.07×10^-3^ | 19 | 50144997 | *SCAF1* |
| cg25028162 | 0.52 | 1.11×10^-5^ | 2.43×10^-2^ | 19 | 52150233 | *SIGLEC14* |
| cg24021890 | -0.27 | 8.40×10^-9^ | 4.10×10^-5^ | 19 | 52552120 | *ZNF432* |
| cg14944575 | -0.30 | 6.43×10^-6^ | 1.48×10^-2^ | 19 | 52552197 | *ZNF432* |
| cg22612295 | 1.00 | 1.11×10^-7^ | 4.63×10^-4^ | 20 | 18393916 | *DZANK1* |
| cg00514271 | -0.50 | 1.53×10^-5^ | 3.25×10^-2^ | 20 | 30593353 |  |
| cg17420774 | -0.77 | 4.93×10^-6^ | 1.16×10^-2^ | 20 | 47931623 |  |
| cg22378865 | -0.90 | 6.68×10^-6^ | 1.53×10^-2^ | 20 | 55781506 | *BMP7* |
| cg17431446 | -0.26 | 1.71×10^-7^ | 6.78×10^-4^ | 20 | 58786338 | *MIR646HG* |
| cg22841856 | -0.98 | 8.12×10^-7^ | 2.55×10^-3^ | 21 | 31743965 | *KRTAP13-2* |
| cg18574499 | -1.00 | 1.70×10^-28^ | 1.39×10^-23^ | 21 | 40669669 | *BRWD1* |
| cg16472408 | -0.84 | 7.91×10^-9^ | 3.89×10^-5^ | 21 | 47984170 | *DIP2A* |
| cg12457928 | -0.64 | 8.52×10^-7^ | 2.65×10^-3^ | 22 | 23076774 |  |
| cg05281340 | 0.17 | 7.84×10^-7^ | 2.49×10^-3^ | 22 | 24191802 |  |
| cg25903490 | -1.00 | 7.24×10^-24^ | 4.10×10^-19^ | 22 | 25477333 | *KIAA1671* |
| cg18307996 | 1.00 | 5.97×10^-9^ | 3.04×10^-5^ | 22 | 31581270 | *RNF185* |
| cg05700365 | -1.00 | 4.08×10^-16^ | 7.68×10^-12^ | 22 | 32362687 |  |
| cg04696109 | -0.50 | 8.51×10^-12^ | 6.89×10^-8^ | 22 | 36081855 |  |
| cg23106733 | 0.91 | 5.31×10^-10^ | 3.32×10^-6^ | 22 | 38325762 | *MICALL1* |
| cg19570171 | 0.16 | 8.68×10^-7^ | 2.69×10^-3^ | 22 | 39239822 | *NPTXR* |
| cg03707464 | 1.00 | 1.70×10^-6^ | 4.70×10^-3^ | 22 | 43842664 | *MPPED1* |
| cg04655303 | 0.15 | 2.35×10^-5^ | 4.65×10^-2^ | 22 | 50699863 | *MAPK12* |
| **CD4+ T cells** | | | | | | |
| cg08730743 | -0.21 | 3.95×10^-6^ | 1.11×10^-2^ | 1 | 2189027 | *SKI* |
| cg12551983 | -0.25 | 6.42×10^-6^ | 1.65×10^-2^ | 1 | 3720588 |  |
| cg20737459 | 0.26 | 1.86×10^-8^ | 1.16×10^-4^ | 1 | 6523514 | *TNFRSF25* |
| cg15619966 | 0.10 | 1.82×10^-5^ | 3.71×10^-2^ | 1 | 9883889 | *CLSTN1* |
| cg15642494 | 0.30 | 1.80×10^-11^ | 3.32×10^-7^ | 1 | 19616829 |  |
| cg08540745 | 0.67 | 4.01×10^-12^ | 9.85×10^-8^ | 1 | 25867637 |  |
| cg10103002 | 0.16 | 6.38×10^-6^ | 1.65×10^-2^ | 1 | 30161580 |  |
| cg22369954 | 0.50 | 4.08×10^-17^ | 2.74×10^-12^ | 1 | 36184863 | *C1orf216* |
| cg03063365 | 0.48 | 4.58×10^-9^ | 3.52×10^-5^ | 1 | 36939085 | *CSF3R* |
| cg20942219 | 0.57 | 3.89×10^-12^ | 9.85×10^-8^ | 1 | 44141161 | *KDM4A* |
| cg27431882 | 0.24 | 2.91×10^-6^ | 8.75×10^-3^ | 1 | 56724990 |  |
| cg26789332 | 0.21 | 1.73×10^-6^ | 5.67×10^-3^ | 1 | 63788255 | *FOXD3* |
| cg17328839 | -0.32 | 1.84×10^-5^ | 3.73×10^-2^ | 1 | 64127751 |  |
| cg10881514 | 0.26 | 9.17×10^-10^ | 9.43×10^-6^ | 1 | 64239618 | *ROR1* |
| cg18117228 | -0.31 | 2.63×10^-5^ | 4.90×10^-2^ | 1 | 65775772 | *DNAJC6* |
| cg16896501 | 0.36 | 2.30×10^-12^ | 6.35×10^-8^ | 1 | 67661575 | *IL23R* |
| cg23042676 | -0.40 | 1.81×10^-6^ | 5.86×10^-3^ | 1 | 67855787 | *IL12RB2* |
| cg27538686 | -0.09 | 2.47×10^-5^ | 4.69×10^-2^ | 1 | 76540641 | *ST6GALNAC3* |
| cg11168446 | 0.28 | 3.04×10^-8^ | 1.76×10^-4^ | 1 | 94312877 | *BCAR3* |
| cg04506601 | 0.17 | 2.29×10^-7^ | 9.87×10^-4^ | 1 | 100435212 | *SLC35A3* |
| cg19083659 | 0.43 | 8.83×10^-14^ | 3.25×10^-9^ | 1 | 100877610 | *CDC14A* |
| cg13860179 | 0.27 | 5.19×10^-10^ | 5.71×10^-6^ | 1 | 113153660 | *ST7L* |
| cg21730450 | 0.22 | 7.19×10^-6^ | 1.80×10^-2^ | 1 | 116866482 |  |
| cg26212868 | -0.29 | 2.43×10^-5^ | 4.61×10^-2^ | 1 | 118087436 |  |
| cg09295759 | 0.25 | 1.50×10^-5^ | 3.19×10^-2^ | 1 | 153282790 | *PGLYRP3* |
| cg20131145 | 0.69 | 3.04×10^-7^ | 1.26×10^-3^ | 1 | 160296456 | *COPA* |
| cg12858504 | -0.17 | 6.62×10^-6^ | 1.69×10^-2^ | 1 | 183184696 | *LAMC2* |
| cg16460669 | 0.25 | 4.29×10^-8^ | 2.26×10^-4^ | 1 | 199659497 |  |
| cg06383339 | -0.26 | 1.96×10^-6^ | 6.27×10^-3^ | 1 | 202380215 | *PPP1R12B* |
| cg22889645 | 0.24 | 2.81×10^-7^ | 1.17×10^-3^ | 1 | 205326399 | *KLHDC8A* |
| cg16186036 | 0.36 | 1.87×10^-5^ | 3.76×10^-2^ | 1 | 208203760 | *PLXNA2* |
| cg04158884 | -0.57 | 6.83×10^-6^ | 1.72×10^-2^ | 1 | 233086346 | *C1orf57* |
| cg19956752 | -0.22 | 1.81×10^-7^ | 8.22×10^-4^ | 2 | 1064077 | *SNTG2* |
| cg07131274 | -0.33 | 6.09×10^-6^ | 1.59×10^-2^ | 2 | 3270259 | *TSSC1* |
| cg03240747 | -0.38 | 3.61×10^-6^ | 1.03×10^-2^ | 2 | 9668004 | *ADAM17* |
| cg10614445 | -0.39 | 1.33×10^-11^ | 2.66×10^-7^ | 2 | 25526696 | *DNMT3A* |
| cg05097535 | 0.30 | 1.32×10^-10^ | 2.06×10^-6^ | 2 | 27353339 | *ABHD1* |
| cg13266592 | -0.29 | 8.57×10^-7^ | 3.14×10^-3^ | 2 | 27385387 |  |
| cg08182009 | 0.26 | 4.21×10^-6^ | 1.16×10^-2^ | 2 | 33329231 | *LTBP1* |
| cg07323088 | -0.49 | 5.79×10^-10^ | 6.28×10^-6^ | 2 | 36741839 | *CRIM1* |
| cg05547766 | 0.24 | 3.86×10^-9^ | 3.16×10^-5^ | 2 | 47232971 | *TTC7A* |
| cg01262626 | -0.36 | 2.36×10^-5^ | 4.53×10^-2^ | 2 | 64828660 |  |
| cg05884910 | -0.29 | 2.96×10^-6^ | 8.82×10^-3^ | 2 | 69693756 | *AAK1* |
| cg20030528 | 0.45 | 2.12×10^-10^ | 2.87×10^-6^ | 2 | 70138723 |  |
| cg09586879 | 0.36 | 2.56×10^-10^ | 3.25×10^-6^ | 2 | 70900488 | *ADD2* |
| cg18560720 | -0.15 | 2.42×10^-5^ | 4.60×10^-2^ | 2 | 96952519 | *SNRNP200* |
| cg13040600 | 0.27 | 2.44×10^-6^ | 7.63×10^-3^ | 2 | 135037975 | *MGAT5* |
| cg08822497 | 0.21 | 8.93×10^-6^ | 2.15×10^-2^ | 2 | 158025305 |  |
| cg23279346 | 0.30 | 1.58×10^-6^ | 5.22×10^-3^ | 2 | 170777718 | *UBR3* |
| cg01167615 | -0.20 | 4.96×10^-6^ | 1.35×10^-2^ | 2 | 174496001 |  |
| cg01093934 | 0.51 | 3.72×10^-8^ | 2.01×10^-4^ | 2 | 177465789 | *MIR1246* |
| cg20640042 | -0.43 | 7.88×10^-7^ | 2.92×10^-3^ | 2 | 189849525 | *COL3A1* |
| cg10902732 | 0.21 | 2.97×10^-8^ | 1.74×10^-4^ | 2 | 203238382 |  |
| cg01352705 | -0.12 | 2.25×10^-8^ | 1.36×10^-4^ | 2 | 216979551 | *XRCC5* |
| cg14660948 | -0.43 | 3.09×10^-8^ | 1.77×10^-4^ | 2 | 217277593 | *SMARCAL1* |
| cg11932445 | 0.29 | 3.69×10^-9^ | 3.09×10^-5^ | 2 | 219263228 | *CTDSP1* |
| cg22049753 | -0.34 | 3.60×10^-8^ | 1.98×10^-4^ | 2 | 240895815 |  |
| cg23212202 | 0.25 | 3.96×10^-9^ | 3.18×10^-5^ | 3 | 30489255 |  |
| cg16020689 | 0.34 | 1.06×10^-6^ | 3.72×10^-3^ | 3 | 35299732 | *LOC101928135* |
| cg21194499 | 0.18 | 2.54×10^-6^ | 7.87×10^-3^ | 3 | 52188578 | *WDR51A* |
| cg04216480 | 0.18 | 4.61×10^-6^ | 1.26×10^-2^ | 3 | 60587837 | *FHIT* |
| cg15766024 | -0.35 | 1.01×10^-11^ | 2.07×10^-7^ | 3 | 99904510 | *TMEM30C* |
| cg25932713 | -0.33 | 8.14×10^-6^ | 2.00×10^-2^ | 3 | 122284563 | *PARP9* |
| cg10513770 | -0.31 | 7.24×10^-6^ | 1.80×10^-2^ | 3 | 122798912 | *PDIA5* |
| cg04960939 | -0.96 | 1.97×10^-10^ | 2.74×10^-6^ | 3 | 130952905 | *NEK11* |
| cg27586272 | 0.23 | 2.96×10^-6^ | 8.82×10^-3^ | 3 | 148884929 | *HPS3* |
| cg22068985 | 0.15 | 2.38×10^-5^ | 4.54×10^-2^ | 3 | 155571279 | *SLC33A1* |
| cg17503645 | 0.55 | 4.44×10^-7^ | 1.76×10^-3^ | 3 | 155837026 | *KCNAB1* |
| cg06332127 | 0.43 | 1.24×10^-5^ | 2.74×10^-2^ | 3 | 158763606 |  |
| cg21125611 | -0.42 | 2.04×10^-5^ | 4.03×10^-2^ | 3 | 160994303 |  |
| cg08760818 | -0.40 | 1.02×10^-6^ | 3.62×10^-3^ | 3 | 167425609 | *PDCD10* |
| cg01787382 | 0.20 | 1.32×10^-5^ | 2.82×10^-2^ | 3 | 169041586 | *MECOM* |
| cg02151273 | -0.38 | 1.06×10^-5^ | 2.47×10^-2^ | 3 | 170019826 | *PRKCI* |
| cg08192869 | 0.16 | 3.96×10^-6^ | 1.11×10^-2^ | 3 | 170068801 |  |
| cg19953985 | -0.20 | 1.15×10^-5^ | 2.58×10^-2^ | 3 | 170923449 | *TNIK* |
| cg10111146 | -0.35 | 2.79×10^-8^ | 1.66×10^-4^ | 3 | 172471603 | *ECT2* |
| cg04738673 | 0.41 | 1.14×10^-5^ | 2.58×10^-2^ | 3 | 177482968 |  |
| cg12942328 | -0.20 | 2.52×10^-7^ | 1.07×10^-3^ | 3 | 187455734 | *BCL6* |
| cg10339098 | 0.33 | 2.10×10^-17^ | 1.72×10^-12^ | 3 | 188313650 | *LPP* |
| cg18279658 | 0.24 | 1.51×10^-5^ | 3.19×10^-2^ | 4 | 1341442 | *UVSSA* |
| cg21471778 | -0.27 | 7.44×10^-8^ | 3.70×10^-4^ | 4 | 1987188 | *WHSC2* |
| cg24588528 | -0.75 | 2.11×10^-5^ | 4.12×10^-2^ | 4 | 3027957 | *GRK4* |
| cg05452911 | -0.36 | 2.14×10^-10^ | 2.87×10^-6^ | 4 | 3221566 | *HTT* |
| cg24709001 | 0.55 | 1.03×10^-6^ | 3.64×10^-3^ | 4 | 3235319 | *HTT* |
| cg03504160 | 0.48 | 1.17×10^-6^ | 4.01×10^-3^ | 4 | 6659194 |  |
| cg12250142 | 0.33 | 3.18×10^-12^ | 8.38×10^-8^ | 4 | 15657883 | *FBXL5* |
| cg01120514 | 0.31 | 1.53×10^-7^ | 7.10×10^-4^ | 4 | 25314414 | *ZCCHC4* |
| cg11669532 | -0.24 | 3.67×10^-8^ | 2.01×10^-4^ | 4 | 53831271 | *SCFD2* |
| cg20445582 | -0.05 | 5.77×10^-6^ | 1.53×10^-2^ | 4 | 55083843 |  |
| cg27585454 | -0.26 | 1.65×10^-5^ | 3.40×10^-2^ | 4 | 56224196 | *SRD5A3* |
| cg06586734 | 0.82 | 1.54×10^-5^ | 3.23×10^-2^ | 4 | 62009326 |  |
| cg13899293 | -0.72 | 2.09×10^-18^ | 2.60×10^-13^ | 4 | 79175365 | *FRAS1* |
| cg07856552 | -0.47 | 2.25×10^-7^ | 9.83×10^-4^ | 4 | 90548906 |  |
| cg10819960 | 0.21 | 2.04×10^-5^ | 4.03×10^-2^ | 4 | 95916983 | *BMPR1B* |
| cg11123177 | 0.23 | 1.98×10^-7^ | 8.75×10^-4^ | 4 | 122063045 | *TNIP3* |
| cg01931840 | -0.23 | 2.74×10^-6^ | 8.39×10^-3^ | 4 | 129192374 | *PGRMC2* |
| cg13339708 | 0.26 | 9.79×10^-6^ | 2.30×10^-2^ | 4 | 185549367 | *CASP3* |
| cg03930088 | 0.24 | 1.12×10^-5^ | 2.55×10^-2^ | 4 | 187647395 |  |
| cg16227022 | -0.32 | 5.41×10^-11^ | 9.28×10^-7^ | 4 | 189898680 |  |
| cg19940052 | 0.23 | 5.52×10^-9^ | 4.11×10^-5^ | 5 | 5474505 | *KIAA0947* |
| cg15288149 | 0.31 | 5.93×10^-6^ | 1.57×10^-2^ | 5 | 6409323 |  |
| cg10952993 | -0.28 | 2.15×10^-7^ | 9.44×10^-4^ | 5 | 31530637 | *DROSHA* |
| cg23790876 | 0.34 | 9.54×10^-6^ | 2.25×10^-2^ | 5 | 31823849 | *PDZD2* |
| cg12053796 | 0.26 | 1.29×10^-5^ | 2.79×10^-2^ | 5 | 58513446 | *PDE4D* |
| cg13665642 | 0.25 | 5.00×10^-8^ | 2.60×10^-4^ | 5 | 73631417 |  |
| cg21436971 | -0.34 | 9.17×10^-7^ | 3.31×10^-3^ | 5 | 107145270 |  |
| cg03667968 | 0.51 | 1.72×10^-10^ | 2.48×10^-6^ | 5 | 112073438 | *APC* |
| cg25511332 | 0.29 | 3.89×10^-7^ | 1.57×10^-3^ | 5 | 113391339 |  |
| cg26201826 | 0.31 | 7.70×10^-7^ | 2.87×10^-3^ | 5 | 114598579 | *PGGT1B* |
| cg25966737 | -0.35 | 4.43×10^-6^ | 1.22×10^-2^ | 5 | 131555010 | *MIR6830* |
| cg16092370 | -0.48 | 2.63×10^-7^ | 1.11×10^-3^ | 5 | 135285974 | *LECT2* |
| cg20540806 | 0.54 | 1.11×10^-6^ | 3.85×10^-3^ | 5 | 140019523 | *TMCO6* |
| cg18394648 | -0.37 | 6.23×10^-17^ | 3.82×10^-12^ | 5 | 141538333 |  |
| cg14615553 | 0.62 | 5.45×10^-7^ | 2.11×10^-3^ | 5 | 147093466 | *JAKMIP2* |
| cg17613241 | 0.27 | 1.99×10^-5^ | 3.97×10^-2^ | 5 | 167069972 | *TENM2* |
| cg01883164 | 0.51 | 1.19×10^-9^ | 1.15×10^-5^ | 5 | 167533108 | *TENM2* |
| cg23066234 | -0.27 | 9.80×10^-7^ | 3.50×10^-3^ | 5 | 174178135 | *MIR4634* |
| cg17807913 | 0.35 | 1.69×10^-19^ | 4.15×10^-14^ | 5 | 176525355 |  |
| cg03730027 | 0.25 | 1.26×10^-5^ | 2.77×10^-2^ | 5 | 180041908 | *FLT4* |
| cg12930304 | -0.47 | 7.12×10^-9^ | 5.20×10^-5^ | 6 | 2818991 |  |
| cg08571475 | 0.51 | 2.01×10^-6^ | 6.40×10^-3^ | 6 | 11494909 |  |
| cg11687389 | -0.24 | 5.72×10^-9^ | 4.21×10^-5^ | 6 | 12900062 | *PHACTR1* |
| cg16269002 | 0.21 | 2.05×10^-6^ | 6.51×10^-3^ | 6 | 13713186 | *RANBP9* |
| cg24822462 | -0.27 | 9.90×10^-11^ | 1.66×10^-6^ | 6 | 15097408 |  |
| cg20284834 | 0.19 | 1.87×10^-7^ | 8.33×10^-4^ | 6 | 15499615 | *JARID2* |
| cg02565494 | 0.49 | 2.52×10^-10^ | 3.25×10^-6^ | 6 | 25287859 | *LRRC16A* |
| cg10750989 | 0.25 | 9.67×10^-19^ | 1.78×10^-13^ | 6 | 31371330 | *MICA* |
| cg09441568 | -0.27 | 2.86×10^-7^ | 1.19×10^-3^ | 6 | 32130993 | *EGFL8* |
| cg02591634 | 0.74 | 1.40×10^-11^ | 2.72×10^-7^ | 6 | 32363268 | *BTNL2* |
| cg08713367 | -0.11 | 2.63×10^-7^ | 1.11×10^-3^ | 6 | 37225473 | *TMEM217* |
| cg27315243 | 0.13 | 2.73×10^-6^ | 8.39×10^-3^ | 6 | 40554736 | *LRFN2* |
| cg00071161 | 0.37 | 1.41×10^-8^ | 9.19×10^-5^ | 6 | 44117573 | *TMEM63B* |
| cg19254793 | 0.40 | 1.53×10^-5^ | 3.22×10^-2^ | 6 | 44695348 |  |
| cg26289989 | 0.74 | 2.57×10^-9^ | 2.23×10^-5^ | 6 | 51085750 |  |
| cg07544038 | 0.08 | 8.96×10^-6^ | 2.15×10^-2^ | 6 | 71122681 | *FAM135A* |
| cg15347924 | 0.65 | 2.75×10^-10^ | 3.44×10^-6^ | 6 | 74073424 | *C6orf221* |
| cg22442841 | -0.39 | 1.37×10^-5^ | 2.92×10^-2^ | 6 | 87646804 | *HTR1E* |
| cg26112901 | -0.34 | 2.98×10^-17^ | 2.20×10^-12^ | 6 | 137494988 | *IL22RA2* |
| cg10369203 | 0.74 | 1.93×10^-8^ | 1.19×10^-4^ | 6 | 143250783 | *HIVEP2* |
| cg20043883 | 0.55 | 2.70×10^-13^ | 9.48×10^-9^ | 6 | 149289357 | *UST* |
| cg21998840 | 0.39 | 1.15×10^-5^ | 2.58×10^-2^ | 7 | 2212030 | *MAD1L1* |
| cg13972711 | -0.47 | 2.79×10^-9^ | 2.39×10^-5^ | 7 | 5391031 | *TNRC18* |
| cg00135038 | -0.30 | 1.75×10^-5^ | 3.59×10^-2^ | 7 | 5391419 | *TNRC18* |
| cg21503490 | 0.18 | 5.16×10^-6^ | 1.39×10^-2^ | 7 | 6773787 | *PMS2CL* |
| cg04985696 | -0.16 | 4.49×10^-9^ | 3.51×10^-5^ | 7 | 44622204 | *TMED4* |
| cg12029626 | 0.19 | 1.10×10^-5^ | 2.53×10^-2^ | 7 | 65216224 | *CCT6P1* |
| cg27528426 | -0.09 | 1.65×10^-6^ | 5.43×10^-3^ | 7 | 65540851 | *ASL* |
| cg06394442 | 0.22 | 1.64×10^-5^ | 3.39×10^-2^ | 7 | 73908364 | *GTF2IRD1* |
| cg26220323 | -0.23 | 2.24×10^-6^ | 7.05×10^-3^ | 7 | 75372083 |  |
| cg14719076 | 0.30 | 9.12×10^-7^ | 3.31×10^-3^ | 7 | 96653747 | *DLX5* |
| cg26724642 | -0.44 | 5.47×10^-9^ | 4.11×10^-5^ | 7 | 97895513 |  |
| cg21751245 | -0.29 | 4.16×10^-10^ | 4.71×10^-6^ | 7 | 99077235 | *ZNF789* |
| cg27084965 | -0.23 | 1.13×10^-9^ | 1.14×10^-5^ | 7 | 103871917 |  |
| cg14865284 | 0.26 | 2.77×10^-6^ | 8.39×10^-3^ | 7 | 104688010 | *MLL5* |
| cg20561100 | 0.16 | 1.85×10^-5^ | 3.75×10^-2^ | 7 | 107204135 | *COG5* |
| cg00269643 | 0.29 | 1.43×10^-8^ | 9.23×10^-5^ | 7 | 128784416 | *TSPAN33* |
| cg12891311 | 0.55 | 3.27×10^-6^ | 9.52×10^-3^ | 7 | 133093517 | *EXOC4* |
| cg21793382 | 0.23 | 1.04×10^-5^ | 2.43×10^-2^ | 7 | 134379148 |  |
| cg07499066 | 0.23 | 1.49×10^-7^ | 6.99×10^-4^ | 7 | 149171379 | *ZNF746* |
| cg10735344 | 0.27 | 6.33×10^-6^ | 1.64×10^-2^ | 7 | 151629493 |  |
| cg20419410 | 0.09 | 4.99×10^-7^ | 1.96×10^-3^ | 7 | 155089803 | *INSIG1* |
| cg22960901 | -0.64 | 4.08×10^-8^ | 2.16×10^-4^ | 7 | 157606434 | *PTPRN2* |
| cg15614872 | 0.29 | 2.55×10^-5^ | 4.79×10^-2^ | 7 | 158531752 | *ESYT2* |
| cg05779585 | 0.59 | 5.66×10^-7^ | 2.16×10^-3^ | 8 | 1246817 |  |
| cg08919372 | -0.14 | 6.66×10^-6^ | 1.69×10^-2^ | 8 | 1370327 |  |
| cg21944972 | -0.23 | 3.53×10^-8^ | 1.96×10^-4^ | 8 | 2020566 | *MYOM2* |
| cg12792075 | -0.82 | 3.03×10^-11^ | 5.31×10^-7^ | 8 | 12244565 | *FAM66A* |
| cg08476824 | 0.61 | 5.10×10^-6^ | 1.38×10^-2^ | 8 | 17172560 | *MTMR7* |
| cg21341889 | 0.22 | 2.04×10^-5^ | 4.03×10^-2^ | 8 | 17773367 |  |
| cg23523780 | 0.22 | 3.83×10^-8^ | 2.06×10^-4^ | 8 | 22972234 | *TNFRSF10C* |
| cg26263051 | -0.23 | 1.86×10^-8^ | 1.16×10^-4^ | 8 | 23114023 | *CHMP7* |
| cg03349819 | 0.28 | 1.59×10^-5^ | 3.31×10^-2^ | 8 | 32404691 | *NRG1* |
| cg12148515 | 0.52 | 1.47×10^-6^ | 4.91×10^-3^ | 8 | 41517301 | *ANK1* |
| cg16725225 | -0.27 | 2.18×10^-9^ | 1.94×10^-5^ | 8 | 53126611 | *ST18* |
| cg18245660 | 0.11 | 1.30×10^-5^ | 2.80×10^-2^ | 8 | 54793541 | *RGS20* |
| cg25356825 | -0.56 | 8.12×10^-12^ | 1.74×10^-7^ | 8 | 59988692 | *TOX* |
| cg03194311 | 0.55 | 3.74×10^-7^ | 1.52×10^-3^ | 8 | 74891031 | *TMEM70* |
| cg07028161 | -0.33 | 4.08×10^-13^ | 1.37×10^-8^ | 8 | 80720093 | *LOC101927040* |
| cg07961887 | -0.07 | 1.22×10^-8^ | 8.43×10^-5^ | 8 | 82192601 | *FABP5* |
| cg23048068 | -0.07 | 1.30×10^-8^ | 8.69×10^-5^ | 8 | 82192604 | *FABP5* |
| cg13076537 | -0.10 | 1.61×10^-9^ | 1.47×10^-5^ | 8 | 82192945 | *FABP5* |
| cg22727641 | 0.40 | 2.10×10^-5^ | 4.10×10^-2^ | 8 | 101157742 | *FBXO43* |
| cg14602154 | -0.18 | 1.29×10^-5^ | 2.79×10^-2^ | 8 | 116618346 | *TRPS1* |
| cg07229027 | -0.35 | 2.43×10^-10^ | 3.20×10^-6^ | 8 | 124408743 | *ATAD2* |
| cg21674704 | 0.32 | 3.63×10^-6^ | 1.03×10^-2^ | 8 | 131455249 |  |
| cg15171273 | 0.25 | 1.85×10^-8^ | 1.16×10^-4^ | 8 | 131539725 |  |
| cg21313067 | -0.30 | 7.02×10^-6^ | 1.76×10^-2^ | 8 | 139164698 | *FAM135B* |
| cg07466463 | -1.00 | 1.64×10^-7^ | 7.52×10^-4^ | 8 | 141574207 | *EIF2C2* |
| cg15988322 | 0.34 | 9.14×10^-6^ | 2.19×10^-2^ | 9 | 22098908 | *CDKN2B-AS1* |
| cg14412639 | -0.35 | 2.31×10^-5^ | 4.46×10^-2^ | 9 | 34098451 | *DCAF12* |
| cg21169617 | 0.29 | 1.89×10^-8^ | 1.17×10^-4^ | 9 | 34989434 | *DNAJB5* |
| cg09587920 | 0.37 | 6.80×10^-6^ | 1.72×10^-2^ | 9 | 73005204 | *KLF9* |
| cg13998223 | 0.83 | 3.60×10^-6^ | 1.03×10^-2^ | 9 | 98389082 |  |
| cg14185994 | 0.16 | 5.65×10^-7^ | 2.16×10^-3^ | 9 | 98637332 | *C9orf130* |
| cg00447672 | -0.34 | 3.14×10^-8^ | 1.77×10^-4^ | 9 | 104184172 | *ALDOB* |
| cg14614881 | 0.57 | 1.23×10^-10^ | 1.97×10^-6^ | 9 | 116982494 | *COL27A1* |
| cg03484259 | 0.33 | 9.94×10^-17^ | 5.23×10^-12^ | 9 | 117049185 | *COL27A1* |
| cg15221416 | 0.60 | 5.64×10^-8^ | 2.91×10^-4^ | 9 | 125021726 | *RBM18* |
| cg10755973 | -0.24 | 3.14×10^-8^ | 1.77×10^-4^ | 9 | 126773879 | *LHX2* |
| cg04456155 | -0.16 | 2.20×10^-5^ | 4.27×10^-2^ | 9 | 126773885 | *LHX2* |
| cg17618153 | 0.60 | 5.81×10^-15^ | 2.38×10^-10^ | 9 | 129874991 | *ANGPTL2* |
| cg14583686 | -0.22 | 1.03×10^-7^ | 5.01×10^-4^ | 9 | 130226589 | *LRSAM1* |
| cg23806774 | 0.29 | 5.52×10^-7^ | 2.13×10^-3^ | 9 | 131218112 | *ODF2* |
| cg17806866 | -0.22 | 6.33×10^-6^ | 1.64×10^-2^ | 9 | 134089754 | *NUP214* |
| cg13875536 | -0.08 | 1.14×10^-5^ | 2.58×10^-2^ | 9 | 135464814 | *BARHL1* |
| cg12233833 | -0.07 | 1.24×10^-5^ | 2.74×10^-2^ | 10 | 830825 |  |
| cg17039793 | -0.14 | 1.95×10^-6^ | 6.27×10^-3^ | 10 | 830909 |  |
| cg26808618 | -0.48 | 5.58×10^-6^ | 1.49×10^-2^ | 10 | 3824016 | *KLF6* |
| cg04430797 | -0.12 | 2.26×10^-6^ | 7.09×10^-3^ | 10 | 6206259 | *PFKFB3* |
| cg27392773 | -0.34 | 1.20×10^-6^ | 4.07×10^-3^ | 10 | 10994094 | *LINC00710* |
| cg15532545 | 0.18 | 1.61×10^-5^ | 3.34×10^-2^ | 10 | 14825999 |  |
| cg24914935 | 0.19 | 3.88×10^-6^ | 1.10×10^-2^ | 10 | 30589494 |  |
| cg09532994 | -0.49 | 9.20×10^-6^ | 2.19×10^-2^ | 10 | 30793024 |  |
| cg14704669 | 0.16 | 6.43×10^-6^ | 1.65×10^-2^ | 10 | 32046612 |  |
| cg26839288 | -0.46 | 5.77×10^-6^ | 1.53×10^-2^ | 10 | 44287132 | *HNRNPA3P1* |
| cg06399914 | -0.36 | 3.29×10^-6^ | 9.55×10^-3^ | 10 | 50589125 | *DRGX* |
| cg14196028 | -0.40 | 2.98×10^-10^ | 3.65×10^-6^ | 10 | 69523483 |  |
| cg01390840 | 0.26 | 9.56×10^-8^ | 4.73×10^-4^ | 10 | 76067347 | *ADK* |
| cg06993191 | 0.18 | 2.03×10^-5^ | 4.03×10^-2^ | 10 | 76514031 |  |
| cg01536438 | -0.28 | 6.66×10^-8^ | 3.39×10^-4^ | 10 | 90712625 | *ACTA2* |
| cg24603634 | 0.26 | 2.35×10^-5^ | 4.53×10^-2^ | 10 | 90925301 |  |
| cg05776902 | -0.51 | 3.12×10^-8^ | 1.77×10^-4^ | 10 | 93349680 | *HECTD2-AS1* |
| cg22949458 | 0.06 | 1.08×10^-6^ | 3.76×10^-3^ | 10 | 97667581 | *C10orf131* |
| cg06930332 | 0.46 | 1.88×10^-7^ | 8.33×10^-4^ | 10 | 102006954 | *CWF19L1* |
| cg15973546 | 0.30 | 1.28×10^-9^ | 1.23×10^-5^ | 10 | 104429532 |  |
| cg10756593 | 0.40 | 1.58×10^-5^ | 3.30×10^-2^ | 10 | 104591244 | *CYP17A1* |
| cg02583335 | -0.22 | 1.05×10^-5^ | 2.44×10^-2^ | 10 | 112519424 | *RBM20* |
| cg07457429 | 0.20 | 1.27×10^-5^ | 2.77×10^-2^ | 10 | 114604800 |  |
| cg16550453 | -0.36 | 1.29×10^-5^ | 2.79×10^-2^ | 10 | 115939018 | *TDRD1* |
| cg07303968 | -0.08 | 9.43×10^-6^ | 2.23×10^-2^ | 10 | 118502243 | *HSPA12A* |
| cg04465599 | -0.04 | 4.46×10^-6^ | 1.22×10^-2^ | 10 | 121652136 | *SEC23IP* |
| cg17642426 | -0.25 | 6.65×10^-6^ | 1.69×10^-2^ | 10 | 124739963 | *PSTK* |
| cg12496783 | -1.00 | 3.25×10^-6^ | 9.51×10^-3^ | 10 | 131568811 |  |
| cg18615506 | 0.21 | 1.27×10^-8^ | 8.61×10^-5^ | 10 | 134177612 | *LRRC27* |
| cg00561124 | 0.56 | 1.73×10^-5^ | 3.56×10^-2^ | 10 | 134410680 | *INPP5A* |
| cg09907570 | -0.36 | 3.25×10^-6^ | 9.51×10^-3^ | 10 | 134839817 |  |
| cg06080571 | 0.41 | 1.87×10^-7^ | 8.33×10^-4^ | 11 | 2349807 |  |
| cg08999895 | -0.29 | 2.23×10^-5^ | 4.32×10^-2^ | 11 | 2925869 | *SLC22A18* |
| cg01494614 | -0.43 | 3.27×10^-7^ | 1.35×10^-3^ | 11 | 10562837 | *RNF141* |
| cg27206735 | 0.30 | 1.58×10^-9^ | 1.47×10^-5^ | 11 | 13708995 | *FAR1* |
| cg00455178 | -0.10 | 5.97×10^-6^ | 1.57×10^-2^ | 11 | 14913981 | *CYP2R1* |
| cg00149585 | -0.07 | 9.74×10^-6^ | 2.29×10^-2^ | 11 | 47207601 | *PACSIN3* |
| cg08628580 | -0.33 | 1.19×10^-5^ | 2.66×10^-2^ | 11 | 58954091 | *DTX4* |
| cg04021098 | -0.47 | 1.50×10^-7^ | 7.00×10^-4^ | 11 | 59482698 | *OR10V1* |
| cg11766552 | 0.38 | 7.68×10^-10^ | 8.21×10^-6^ | 11 | 65547881 | *DKFZp761E198* |
| cg03613003 | -0.36 | 7.58×10^-9^ | 5.47×10^-5^ | 11 | 69949263 | *ANO1* |
| cg09100910 | -0.31 | 1.35×10^-7^ | 6.40×10^-4^ | 11 | 72020704 | *CLPB* |
| cg10495997 | -0.38 | 1.93×10^-6^ | 6.25×10^-3^ | 11 | 72438402 | *ARAP1* |
| cg16663931 | -0.77 | 5.24×10^-18^ | 5.51×10^-13^ | 11 | 75882654 |  |
| cg16745952 | 0.30 | 6.66×10^-8^ | 3.39×10^-4^ | 11 | 98891665 | *CNTN5* |
| cg15314149 | 0.21 | 5.36×10^-6^ | 1.44×10^-2^ | 11 | 108074227 | *NPAT* |
| cg18224077 | 0.37 | 7.92×10^-12^ | 1.74×10^-7^ | 11 | 121972185 | *LOC399959* |
| cg19649564 | 0.28 | 2.90×10^-8^ | 1.71×10^-4^ | 11 | 124932892 | *SLC37A2* |
| cg05297666 | -0.20 | 5.98×10^-6^ | 1.57×10^-2^ | 11 | 128400310 | *ETS1* |
| cg06072950 | -0.17 | 2.05×10^-5^ | 4.03×10^-2^ | 11 | 129736336 | *NFRKB* |
| cg02986334 | 0.61 | 8.26×10^-6^ | 2.01×10^-2^ | 12 | 292211 |  |
| cg04636406 | 0.15 | 9.32×10^-7^ | 3.35×10^-3^ | 12 | 6993009 | *RPL13P5* |
| cg10873203 | 0.21 | 1.18×10^-6^ | 4.01×10^-3^ | 12 | 25942772 |  |
| cg10736528 | -0.72 | 1.26×10^-12^ | 3.73×10^-8^ | 12 | 52772961 | *KRT84* |
| cg17129918 | 0.33 | 2.52×10^-5^ | 4.73×10^-2^ | 12 | 53098560 | *KRT77* |
| cg01708411 | -0.51 | 1.60×10^-9^ | 1.47×10^-5^ | 12 | 57631839 | *NDUFA4L2* |
| cg26223054 | 0.33 | 3.75×10^-10^ | 4.32×10^-6^ | 12 | 58201681 | *AVIL* |
| cg15505491 | -0.61 | 2.38×10^-5^ | 4.54×10^-2^ | 12 | 101127597 |  |
| cg14079445 | -0.27 | 6.76×10^-7^ | 2.54×10^-3^ | 12 | 111493997 | *CUX2* |
| cg03240232 | 0.61 | 6.94×10^-8^ | 3.50×10^-4^ | 12 | 113448327 | *OAS2* |
| cg05696801 | 0.26 | 2.16×10^-9^ | 1.94×10^-5^ | 12 | 118573886 | *PEBP1* |
| cg23608041 | 0.34 | 2.07×10-5 | 4.06×10^-2^ | 12 | 122326398 | *PSMD9* |
| cg15244603 | 0.29 | 3.20×10^-9^ | 2.71×10^-5^ | 12 | 125607520 | *AACS* |
| cg26996082 | -0.24 | 1.31×10^-6^ | 4.41×10^-3^ | 13 | 20797622 | *GJB6* |
| cg07397503 | 0.23 | 1.34×10^-7^ | 6.37×10^-4^ | 13 | 23471764 |  |
| cg13777984 | 0.34 | 7.13×10^-17^ | 4.04×10^-12^ | 13 | 43931730 | *ENOX1* |
| cg03860241 | -0.54 | 4.10×10^-6^ | 1.14×10^-2^ | 13 | 46863058 |  |
| cg08762237 | -0.73 | 1.96×10^-10^ | 2.74×10^-6^ | 13 | 78667308 | *RNF219-AS1* |
| cg11772527 | 0.33 | 9.09×10^-7^ | 3.31×10^-3^ | 13 | 94161642 | *GPC6* |
| cg20455802 | -0.42 | 1.09×10^-5^ | 2.52×10^-2^ | 13 | 112062882 |  |
| cg01552748 | -0.30 | 4.50×10^-13^ | 1.44×10^-8^ | 13 | 112225526 |  |
| cg27134342 | 0.34 | 1.34×10^-8^ | 8.80×10^-5^ | 14 | 21166074 | *RNASE4* |
| cg15852141 | -0.24 | 4.55×10^-8^ | 2.38×10^-4^ | 14 | 22891383 |  |
| cg18366651 | 0.23 | 2.79×10^-6^ | 8.42×10^-3^ | 14 | 23305239 | *MMP14* |
| cg17864209 | 0.27 | 2.49×10^-5^ | 4.70×10^-2^ | 14 | 35805102 |  |
| cg06243610 | 0.22 | 2.76×10^-6^ | 8.39×10^-3^ | 14 | 52373839 | *GNG2* |
| cg10376133 | -0.46 | 1.10×10^-5^ | 2.53×10^-2^ | 14 | 57049860 | *C14orf101* |
| cg24192328 | 0.07 | 8.16×10^-6^ | 2.00×10^-2^ | 14 | 75422347 | *PGF* |
| cg12838644 | -0.11 | 1.12×10^-6^ | 3.87×10^-3^ | 14 | 77413642 |  |
| cg22903735 | -0.13 | 1.79×10^-6^ | 5.85×10^-3^ | 14 | 77413674 |  |
| cg14519717 | 0.25 | 2.64×10^-6^ | 8.14×10^-3^ | 14 | 90744790 | *C14orf102* |
| cg03325535 | 0.43 | 1.24×10^-8^ | 8.45×10^-5^ | 15 | 27111949 | *GABRA5* |
| cg15336988 | 0.31 | 3.97×10^-9^ | 3.18×10^-5^ | 15 | 31566306 |  |
| cg07709681 | 0.15 | 1.29×10^-5^ | 2.79×10^-2^ | 15 | 34393712 | *PGBD4* |
| cg24245086 | 0.52 | 1.38×10^-6^ | 4.62×10^-3^ | 15 | 44164534 | *FRMD5* |
| cg26409497 | -0.31 | 1.01×10^-7^ | 4.95×10^-4^ | 15 | 58331384 | *ALDH1A2* |
| cg16301926 | -0.34 | 2.50×10^-5^ | 4.70×10^-2^ | 15 | 91653565 | *SV2B* |
| cg26739280 | -0.08 | 1.06×10^-6^ | 3.72×10^-3^ | 15 | 92936818 | *ST8SIA2* |
| cg12523790 | -0.19 | 3.91×10^-6^ | 1.10×10^-2^ | 15 | 94898129 | *MCTP2* |
| cg01037989 | -0.32 | 1.32×10^-5^ | 2.83×10^-2^ | 16 | 966659 | *LMF1* |
| cg14939446 | -0.24 | 1.40×10^-10^ | 2.13×10^-6^ | 16 | 2571015 | *AMDHD2* |
| cg16385478 | -0.13 | 2.58×10^-5^ | 4.82×10^-2^ | 16 | 3723376 | *TRAP1* |
| cg00454447 | 0.25 | 1.13×10^-5^ | 2.57×10^-2^ | 16 | 3728185 | *TRAP1* |
| cg11586377 | 0.24 | 1.19×10^-5^ | 2.66×10^-2^ | 16 | 22263785 | *EEF2K* |
| cg19415738 | -0.34 | 3.47×10^-10^ | 4.06×10^-6^ | 16 | 30418482 | *ZNF771* |
| cg04342763 | -0.47 | 2.27×10^-7^ | 9.85×10^-4^ | 16 | 49937177 |  |
| cg12722136 | -0.37 | 4.88×10^-7^ | 1.92×10^-3^ | 16 | 59275014 |  |
| cg02945354 | 0.40 | 6.35×10^-12^ | 1.51×10^-7^ | 16 | 64295125 |  |
| cg27453761 | 0.24 | 4.54×10^-10^ | 5.07×10^-6^ | 16 | 66624346 |  |
| cg19989371 | -0.22 | 3.25×10^-6^ | 9.51×10^-3^ | 16 | 67335686 | *KCTD19* |
| cg08183066 | -0.14 | 1.20×10^-7^ | 5.77×10^-4^ | 16 | 70333124 | *DDX19B* |
| cg16777782 | -0.35 | 2.38×10^-15^ | 1.10×10^-10^ | 16 | 82671333 | *CDH13* |
| cg16053428 | 0.28 | 5.33×10^-13^ | 1.64×10^-8^ | 16 | 83990544 | *OSGIN1* |
| cg07391271 | 0.30 | 3.43×10^-8^ | 1.91×10^-4^ | 16 | 89588846 | *SPG7* |
| cg26822161 | 0.17 | 3.77×10^-7^ | 1.53×10^-3^ | 16 | 89772612 |  |
| cg16732769 | -0.38 | 2.34×10^-9^ | 2.05×10^-5^ | 16 | 89973661 | *TCF25* |
| cg02558505 | -0.06 | 9.56×10^-9^ | 6.77×10^-5^ | 17 | 21156701 | *NATD1* |
| cg04556555 | -0.26 | 8.19×10^-6^ | 2.00×10^-2^ | 17 | 33477186 | *UNC45B* |
| cg11862993 | 0.27 | 2.22×10^-5^ | 4.31×10^-2^ | 17 | 36103978 | *HNF1B* |
| cg02859521 | 0.22 | 2.47×10^-7^ | 1.06×10^-3^ | 17 | 43332672 | *MAP3K14-AS1* |
| cg21848909 | 0.32 | 8.22×10^-6^ | 2.00×10^-2^ | 17 | 43365148 | *MAP3K14* |
| cg14295458 | 0.21 | 2.47×10^-6^ | 7.68×10^-3^ | 17 | 45891164 | *OSBPL7* |
| cg26892115 | -0.05 | 6.21×10^-7^ | 2.34×10^-3^ | 17 | 48636898 | *CACNA1G-AS1* |
| cg20672426 | -0.37 | 1.50×10^-15^ | 7.35×10^-11^ | 17 | 53851054 | *PCTP* |
| cg17182054 | -0.26 | 4.50×10^-9^ | 3.51×10^-5^ | 17 | 54588075 |  |
| cg23635206 | -0.22 | 5.02×10^-7^ | 1.96×10^-3^ | 17 | 71620913 | *SDK2* |
| cg07027472 | 0.19 | 3.41×10^-6^ | 9.86×10^-3^ | 17 | 73391422 | *GRB2* |
| cg19169246 | -0.28 | 9.38×10^-18^ | 8.64×10^-13^ | 17 | 73887208 | *TRIM65* |
| cg15275493 | -0.42 | 1.43×10^-10^ | 2.13×10^-6^ | 17 | 75310725 | *9-Sep* |
| cg03103060 | -0.45 | 6.99×10^-7^ | 2.62×10^-3^ | 17 | 79340526 |  |
| cg21487261 | -0.11 | 1.30×10^-5^ | 2.80×10^-2^ | 17 | 79368981 |  |
| cg16681349 | 0.25 | 3.20×10^-6^ | 9.51×10^-3^ | 17 | 80614369 |  |
| cg07431961 | 0.40 | 1.24×10^-5^ | 2.74×10^-2^ | 17 | 80902556 | *B3GNTL1* |
| cg16717605 | -0.36 | 1.17×10^-5^ | 2.63×10^-2^ | 18 | 691284 | *ENOSF1* |
| cg09288233 | 0.57 | 9.86×10^-26^ | 3.63×10^-20^ | 18 | 3449455 | *TGIF1* |
| cg18554492 | 0.84 | 1.03×10^-5^ | 2.42×10^-2^ | 18 | 19406813 | *MIR133A1HG* |
| cg25185915 | 0.25 | 1.22×10^-8^ | 8.43×10^-5^ | 18 | 74507281 | *LOC100131655* |
| cg17185889 | 0.22 | 9.22×10^-10^ | 9.43×10^-6^ | 18 | 74685615 |  |
| cg05535853 | 0.26 | 4.36×10^-7^ | 1.75×10^-3^ | 18 | 74781719 | *MBP* |
| cg16421653 | 0.42 | 1.21×10^-7^ | 5.77×10^-4^ | 18 | 77251876 | *NFATC1* |
| cg00942551 | -0.08 | 1.12×10^-5^ | 2.56×10^-2^ | 19 | 3585406 | *GIPC3* |
| cg26090256 | -0.33 | 5.32×10^-6^ | 1.43×10^-2^ | 19 | 3958507 | *DAPK3* |
| cg04272577 | 0.31 | 1.18×10^-9^ | 1.15×10^-5^ | 19 | 4258906 | *CCDC94* |
| cg08243301 | -0.42 | 8.13×10^-9^ | 5.81×10^-5^ | 19 | 5079317 | *KDM4B* |
| cg21973527 | -0.36 | 4.04×10^-8^ | 2.16×10^-4^ | 19 | 5090962 | *KDM4B* |
| cg04592958 | 0.11 | 1.58×10^-7^ | 7.27×10^-4^ | 19 | 5138093 | *KDM4B* |
| cg10585208 | 0.34 | 1.82×10^-5^ | 3.71×10^-2^ | 19 | 7854021 | *CLEC4GP1* |
| cg09777386 | -0.19 | 1.96×10^-5^ | 3.92×10^-2^ | 19 | 9648349 | *ZNF426* |
| cg05212440 | -0.41 | 1.86×10^-7^ | 8.33×10^-4^ | 19 | 10973874 | *C19orf38* |
| cg22933199 | -0.44 | 3.78×10^-9^ | 3.13×10^-5^ | 19 | 12163909 | *ZNF878* |
| cg19412076 | 0.33 | 4.70×10^-9^ | 3.57×10^-5^ | 19 | 12661732 | *ZNF564* |
| cg18025793 | 0.27 | 2.07×10^-5^ | 4.06×10^-2^ | 19 | 17097121 | *CPAMD8* |
| cg19350808 | -0.42 | 1.77×10^-5^ | 3.62×10^-2^ | 19 | 28401874 |  |
| cg04715864 | -0.61 | 1.86×10^-5^ | 3.76×10^-2^ | 19 | 33888321 | *PEPD* |
| cg10105623 | -0.19 | 6.19×10^-7^ | 2.34×10^-3^ | 19 | 34893958 | *PDCD2L* |
| cg22071238 | -0.64 | 1.17×10^-7^ | 5.67×10^-4^ | 19 | 36630543 | *CAPNS1* |
| cg14465187 | -0.40 | 1.08×10^-10^ | 1.76×10^-6^ | 19 | 41220981 | *ADCK4* |
| cg01009059 | 0.20 | 1.57×10^-6^ | 5.21×10^-3^ | 19 | 46391024 |  |
| cg07101466 | 0.40 | 1.74×10^-8^ | 1.11×10^-4^ | 19 | 47197139 | *PRKD2* |
| cg23300543 | -0.43 | 3.02×10^-10^ | 3.65×10^-6^ | 19 | 48707195 |  |
| cg01622465 | -0.39 | 1.44×10^-11^ | 2.72×10^-7^ | 19 | 48707291 |  |
| cg15073624 | -0.41 | 1.15×10^-9^ | 1.14×10^-5^ | 19 | 48707384 |  |
| cg19771773 | -0.51 | 2.81×10^-11^ | 5.05×10^-7^ | 19 | 48707517 |  |
| cg19904905 | -0.42 | 8.94×10^-10^ | 9.41×10^-6^ | 19 | 49469667 | *FTL* |
| cg12404700 | -0.28 | 1.42×10^-9^ | 1.34×10^-5^ | 19 | 51822506 | *IGLON5* |
| cg13330919 | 0.30 | 3.38×10^-10^ | 4.02×10^-6^ | 20 | 20205957 | *CFAP61* |
| cg23738323 | 0.36 | 7.91×10^-6^ | 1.96×10^-2^ | 20 | 32206313 | *CBFA2T2* |
| cg07273583 | -0.27 | 7.12×10^-8^ | 3.57×10^-4^ | 20 | 34516631 | *PHF20* |
| cg14086952 | -0.37 | 8.63×10^-6^ | 2.09×10^-2^ | 20 | 35156428 | *DLGAP4* |
| cg02396020 | -0.39 | 2.33×10^-12^ | 6.35×10^-8^ | 20 | 44778076 |  |
| cg01029027 | -0.44 | 1.06×10^-8^ | 7.42×10^-5^ | 20 | 48557688 | *RNF114* |
| cg26364766 | -0.39 | 2.12×10^-18^ | 2.60×10^-13^ | 20 | 48570238 | *RNF114* |
| cg09740920 | 0.25 | 3.89×10^-6^ | 1.10×10^-2^ | 20 | 50123930 | *NFATC2* |
| cg00382808 | 0.34 | 9.32×10^-6^ | 2.21×10^-2^ | 20 | 51156597 |  |
| cg17431446 | 0.11 | 4.41×10^-7^ | 1.76×10^-3^ | 20 | 58786338 | *MIR646HG* |
| cg08015776 | -0.10 | 1.45×10^-10^ | 2.13×10^-6^ | 21 | 27543229 | *APP* |
| cg13108038 | 0.78 | 1.42×10^-27^ | 1.05×10^-21^ | 21 | 30607031 | *LINC00189* |
| cg18574499 | 0.25 | 1.32×10^-8^ | 8.75×10^-5^ | 21 | 40669669 | *BRWD1* |
| cg04477510 | 0.52 | 6.78×10^-6^ | 1.72×10^-2^ | 21 | 44582492 |  |
| cg10691742 | -0.30 | 8.27×10^-12^ | 1.74×10^-7^ | 21 | 46570664 | *ADARB1* |
| cg05281340 | -0.08 | 8.18×10^-7^ | 3.01×10^-3^ | 22 | 24191802 |  |
| cg25903490 | 0.26 | 1.96×10^-8^ | 1.19×10^-4^ | 22 | 25477333 | *KIAA1671* |
| cg04483623 | -0.21 | 1.58×10^-5^ | 3.30×10^-2^ | 22 | 29949575 | *THOC5* |
| cg04696109 | 0.15 | 3.60×10^-6^ | 1.03×10^-2^ | 22 | 36081855 |  |
| cg22632063 | 0.19 | 1.18×10^-6^ | 4.01×10^-3^ | 22 | 38696180 | *CSNK1E* |
| cg06511098 | 1.00 | 1.89×10^-5^ | 3.79×10^-2^ | 22 | 48314866 |  |
| **CD8+ T cells** | | | | | | |
| cg09599979 | 0.39 | 4.00×10^-15^ | 1.09×10^-10^ | 1 | 6660000 | *KLHL21* |
| cg25369015 | -0.10 | 1.47×10^-6^ | 4.64×10^-3^ | 1 | 9599256 | *SLC25A33* |
| cg04969937 | 0.31 | 1.76×10^-5^ | 3.79×10^-2^ | 1 | 9883887 | *CLSTN1* |
| cg15619966 | 0.33 | 7.10×10^-30^ | 1.05×10^-24^ | 1 | 9883889 | *CLSTN1* |
| cg12621203 | -0.48 | 5.19×10^-6^ | 1.37×10^-2^ | 1 | 23750509 | *TCEA3* |
| cg21369695 | -0.62 | 6.15×10^-7^ | 2.21×10^-3^ | 1 | 32120996 | *COL16A1* |
| cg06772729 | 0.58 | 1.58×10^-8^ | 8.98×10^-5^ | 1 | 33835165 | *PHC2* |
| cg03063365 | 0.87 | 2.73×10^-19^ | 1.26×10^-14^ | 1 | 36939085 | *CSF3R* |
| cg23341459 | -0.39 | 1.69×10^-6^ | 5.24×10^-3^ | 1 | 40204657 | *PPIE* |
| cg06619906 | 0.24 | 1.65×10^-5^ | 3.65×10^-2^ | 1 | 47996107 |  |
| cg15738268 | -1.00 | 5.48×10^-12^ | 5.68×10^-8^ | 1 | 61439110 |  |
| cg10881514 | 0.71 | 5.75×10^-45^ | 4.24×10^-39^ | 1 | 64239618 | *ROR1* |
| cg23042676 | -1.00 | 1.55×10^-31^ | 3.23×10^-26^ | 1 | 67855787 | *IL12RB2* |
| cg02796548 | 0.18 | 3.32×10^-6^ | 9.27×10^-3^ | 1 | 92372096 | *TGFBR3* |
| cg10096873 | 0.81 | 2.04×10^-10^ | 1.63×10^-6^ | 1 | 92546470 | *BTBD8* |
| cg03293269 | 0.32 | 1.74×10^-5^ | 3.77×10^-2^ | 1 | 93286299 |  |
| cg13860179 | -0.24 | 2.88×10^-6^ | 8.25×10^-3^ | 1 | 113153660 | *ST7L* |
| cg21730450 | 0.25 | 1.45×10^-5^ | 3.24×10^-2^ | 1 | 116866482 |  |
| cg26212868 | -0.58 | 5.38×10^-13^ | 8.04×10^-9^ | 1 | 118087436 |  |
| cg10827479 | 0.73 | 2.39×10^-7^ | 9.65×10^-4^ | 1 | 152061363 | *TCHHL1* |
| cg08721840 | 0.41 | 7.51×10^-10^ | 5.27×10^-6^ | 1 | 155293541 | *RUSC1* |
| cg07195452 | -0.24 | 2.04×10^-6^ | 6.19×10^-3^ | 1 | 182109534 |  |
| cg13579034 | -0.49 | 8.24×10^-8^ | 3.82×10^-4^ | 1 | 186510597 |  |
| cg16460669 | 0.40 | 7.08×10^-14^ | 1.54×10^-9^ | 1 | 199659497 |  |
| cg07692949 | 0.25 | 2.53×10^-7^ | 1.00×10^-3^ | 1 | 204425773 | *PIK3C2B* |
| cg19667051 | -0.39 | 6.35×10^-11^ | 5.63×10^-7^ | 1 | 207046933 |  |
| cg09261111 | -0.80 | 1.56×10^-8^ | 8.94×10^-5^ | 1 | 229736278 | *TAF5L* |
| cg11809582 | -0.51 | 1.24×10^-13^ | 2.41×10^-9^ | 1 | 235291861 | *SNORA14B* |
| cg03240747 | -1.00 | 1.75×10^-31^ | 3.23×10^-26^ | 2 | 9668004 | *ADAM17* |
| cg10927841 | 0.12 | 8.80×10^-14^ | 1.80×10^-9^ | 2 | 26407743 | *FAM59B* |
| cg08182009 | 0.46 | 2.50×10^-12^ | 2.93×10^-8^ | 2 | 33329231 | *LTBP1* |
| cg06281904 | -0.53 | 2.54×10^-13^ | 4.16×10^-9^ | 2 | 37041582 | *VIT* |
| cg11768422 | -0.84 | 3.77×10^-7^ | 1.43×10^-3^ | 2 | 39552637 | *MAP4K3* |
| cg19454294 | -0.59 | 2.09×10^-6^ | 6.30×10^-3^ | 2 | 68466335 | *PPP3R1* |
| cg15127613 | 0.64 | 1.07×10^-6^ | 3.49×10^-3^ | 2 | 75299705 | *TACR1* |
| cg25535468 | 0.42 | 8.02×10^-6^ | 1.98×10^-2^ | 2 | 98986314 | *CNGA3* |
| cg14595315 | 0.31 | 1.18×10^-5^ | 2.73×10^-2^ | 2 | 101609869 | *NPAS2* |
| cg22865501 | 0.45 | 6.21×10^-8^ | 2.97×10^-4^ | 2 | 105321544 |  |
| cg23460843 | 0.19 | 7.37×10^-6^ | 1.84×10^-2^ | 2 | 127644075 |  |
| cg02265177 | 0.44 | 2.46×10^-6^ | 7.30×10^-3^ | 2 | 146882515 |  |
| cg08822497 | 0.25 | 8.45×10^-6^ | 2.07×10^-2^ | 2 | 158025305 |  |
| cg05569109 | 0.07 | 1.03×10^-5^ | 2.42×10^-2^ | 2 | 166650805 | *GALNT3* |
| cg24217704 | 0.37 | 2.08×10^-9^ | 1.33×10^-5^ | 2 | 166650930 | *GALNT3* |
| cg10830649 | 0.34 | 6.31×10^-11^ | 5.63×10^-7^ | 2 | 166650933 | *GALNT3* |
| cg20146030 | 0.23 | 2.50×10^-6^ | 7.36×10^-3^ | 2 | 166650965 | *GALNT3* |
| cg19648955 | 0.24 | 8.53×10^-6^ | 2.08×10^-2^ | 2 | 177134095 | *MTX2* |
| cg21166445 | 0.44 | 3.47×10^-7^ | 1.34×10^-3^ | 2 | 181843895 | *UBE2E3* |
| cg00372801 | -0.46 | 6.80×10^-18^ | 2.95×10^-13^ | 2 | 191219050 | *INPP1* |
| cg16652648 | 0.26 | 8.71×10^-16^ | 2.79×10^-11^ | 2 | 197791396 | *PGAP1* |
| cg04865026 | -0.57 | 2.95×10^-6^ | 8.40×10^-3^ | 2 | 201936505 | *NDUFB3* |
| cg21084456 | 0.28 | 9.96×10^-7^ | 3.33×10^-3^ | 2 | 217674497 |  |
| cg20174636 | 0.66 | 9.68×10^-7^ | 3.27×10^-3^ | 2 | 220299900 | *SPEG* |
| cg14616942 | -0.96 | 1.31×10^-5^ | 2.98×10^-2^ | 2 | 220369237 | *GMPPA* |
| cg23127249 | 0.16 | 2.52×10^-6^ | 7.38×10^-3^ | 2 | 232790974 | *NPPC* |
| cg11491420 | -0.59 | 1.83×10^-12^ | 2.25×10^-8^ | 2 | 242543287 | *THAP4* |
| cg16512192 | 0.74 | 1.26×10^-7^ | 5.60×10^-4^ | 3 | 10805672 | *LINC00606* |
| cg02690148 | 0.64 | 1.39×10^-7^ | 6.04×10^-4^ | 3 | 17375660 | *TBC1D5* |
| cg25336332 | 0.42 | 2.03×10^-8^ | 1.11×10^-4^ | 3 | 24537309 | *THRB* |
| cg16020689 | 0.43 | 1.92×10^-7^ | 7.99×10^-4^ | 3 | 35299732 | *LOC101928135* |
| cg08986653 | -0.31 | 3.66×10^-7^ | 1.40×10^-3^ | 3 | 40498475 | *RPL14* |
| cg25831568 | 0.45 | 1.71×10^-5^ | 3.71×10^-2^ | 3 | 47843022 | *DHX30* |
| cg10893140 | 0.15 | 1.09×10^-17^ | 4.46×10^-13^ | 3 | 49158297 | *USP19* |
| cg07285807 | -0.75 | 3.38×10^-15^ | 9.58×10^-11^ | 3 | 49235826 | *CCDC36* |
| cg04216480 | 0.30 | 1.53×10^-10^ | 1.25×10^-6^ | 3 | 60587837 | *FHIT* |
| cg15721436 | 0.59 | 2.84×10^-6^ | 8.17×10^-3^ | 3 | 84928932 |  |
| cg05148217 | -0.41 | 6.71×10^-6^ | 1.69×10^-2^ | 3 | 108836878 | *MORC1* |
| cg02797353 | -0.36 | 2.79×10^-6^ | 8.07×10^-3^ | 3 | 119531385 | *NR1I2* |
| cg17033827 | 0.21 | 1.75×10^-5^ | 3.77×10^-2^ | 3 | 121727373 | *ILDR1* |
| cg14208151 | -0.43 | 9.31×10^-8^ | 4.26×10^-4^ | 3 | 123476889 | *MYLK* |
| cg06648971 | 0.09 | 8.76×10^-6^ | 2.12×10^-2^ | 3 | 125239030 | *SNX4* |
| cg02080175 | -0.52 | 4.72×10^-7^ | 1.75×10^-3^ | 3 | 128056483 | *EEFSEC* |
| cg08958945 | -0.47 | 2.75×10^-14^ | 6.54×10^-10^ | 3 | 133591690 | *RAB6B* |
| cg11363648 | -0.41 | 1.81×10^-7^ | 7.63×10^-4^ | 3 | 142374636 | *PLS1* |
| cg27586272 | 0.36 | 7.48×10^-10^ | 5.27×10^-6^ | 3 | 148884929 | *HPS3* |
| cg04766958 | -0.33 | 8.22×10^-11^ | 7.04×10^-7^ | 3 | 150474306 | *SIAH2* |
| cg17976545 | -0.38 | 1.28×10^-8^ | 7.41×10^-5^ | 3 | 152278124 |  |
| cg02151273 | -0.50 | 7.06×10^-7^ | 2.48×10^-3^ | 3 | 170019826 | *PRKCI* |
| cg16653588 | 0.29 | 6.75×10^-6^ | 1.70×10^-2^ | 3 | 194298696 |  |
| cg04125205 | 0.47 | 7.38×10^-6^ | 1.84×10^-2^ | 4 | 649540 | *PDE6B* |
| cg27301092 | 0.27 | 3.39×10^-9^ | 2.12×10^-5^ | 4 | 1352452 | *KIAA1530* |
| cg17764992 | -0.60 | 1.15×10^-6^ | 3.70×10^-3^ | 4 | 3853774 |  |
| cg03504160 | 0.72 | 7.41×10^-10^ | 5.27×10^-6^ | 4 | 6659194 |  |
| cg00598021 | 0.77 | 1.33×10^-5^ | 2.99×10^-2^ | 4 | 10113794 | *WDR1* |
| cg00035889 | 0.51 | 2.12×10^-8^ | 1.15×10^-4^ | 4 | 38689910 | *KLF3* |
| cg15522136 | -0.42 | 9.34×10^-6^ | 2.24×10^-2^ | 4 | 42893968 | *GRXCR1* |
| cg07520341 | -0.45 | 3.56×10^-12^ | 3.97×10^-8^ | 4 | 54132843 | *SCFD2* |
| cg11424561 | -0.55 | 1.26×10^-8^ | 7.36×10^-5^ | 4 | 58042912 | *IGFBP7-AS1* |
| cg10053430 | 0.47 | 1.42×10^-6^ | 4.51×10^-3^ | 4 | 83500062 |  |
| cg02886293 | -0.64 | 7.04×10^-12^ | 7.21×10^-8^ | 4 | 111462716 | *ENPEP* |
| cg11123177 | 0.32 | 3.12×10^-10^ | 2.44×10^-6^ | 4 | 122063045 | *TNIP3* |
| cg11695370 | 0.43 | 2.65×10^-8^ | 1.37×10^-4^ | 4 | 160311361 |  |
| cg09029959 | -1.00 | 4.28×10^-14^ | 9.55×10^-10^ | 4 | 163905924 |  |
| cg10163536 | 0.61 | 9.91×10^-6^ | 2.36×10^-2^ | 4 | 174845485 |  |
| cg13809671 | -0.49 | 6.98×10^-7^ | 2.46×10^-3^ | 5 | 2369186 |  |
| cg24298788 | 0.48 | 2.52×10^-6^ | 7.38×10^-3^ | 5 | 36214561 | *NADK2* |
| cg10556369 | 0.25 | 1.94×10^-7^ | 8.01×10^-4^ | 5 | 36619118 | *SLC1A3* |
| cg23190340 | -0.49 | 9.09×10^-6^ | 2.19×10^-2^ | 5 | 36665475 | *SLC1A3* |
| cg15120413 | -0.59 | 2.34×10^-5^ | 4.94×10^-2^ | 5 | 53753195 |  |
| cg05691871 | -0.45 | 1.26×10^-5^ | 2.87×10^-2^ | 5 | 58535559 | *PDE4D* |
| cg18058352 | 0.36 | 2.80×10^-7^ | 1.09×10^-3^ | 5 | 127675527 | *FBN2* |
| cg24887267 | 0.05 | 5.92×10^-6^ | 1.54×10^-2^ | 5 | 131563504 | *P4HA2* |
| cg14615362 | -0.51 | 1.53×10^-9^ | 1.00×10^-5^ | 5 | 131973793 | *RAD50* |
| cg17465930 | -0.34 | 3.92×10^-8^ | 1.94×10^-4^ | 5 | 138630302 | *MATR3* |
| cg08691836 | 0.12 | 2.26×10^-10^ | 1.79×10^-6^ | 5 | 150460750 | *TNIP1* |
| cg23165500 | 0.35 | 1.31×10^-7^ | 5.78×10^-4^ | 5 | 152949095 | *GRIA1* |
| cg22851670 | -0.43 | 1.45×10^-9^ | 9.60×10^-6^ | 5 | 156531741 | *HAVCR2* |
| cg01883164 | 0.70 | 8.05×10^-13^ | 1.12×10^-8^ | 5 | 167533108 | *TENM2* |
| cg15926342 | 0.24 | 1.80×10^-5^ | 3.86×10^-2^ | 5 | 171881757 | *SH3PXD2B* |
| cg12982374 | -0.59 | 2.89×10^-8^ | 1.46×10^-4^ | 5 | 176523370 | *FGFR4* |
| cg01169396 | -0.60 | 1.06×10^-6^ | 3.49×10^-3^ | 5 | 179372900 |  |
| cg03730027 | 0.49 | 5.47×10^-13^ | 8.04×10^-9^ | 5 | 180041908 | *FLT4* |
| cg16958184 | 0.45 | 4.00×10^-6^ | 1.09×10^-2^ | 6 | 2685592 | *MYLK4* |
| cg12930304 | -0.68 | 4.40×10^-13^ | 6.76×10^-9^ | 6 | 2818991 |  |
| cg21641834 | 0.23 | 2.98×10^-6^ | 8.46×10^-3^ | 6 | 5068684 |  |
| cg08571475 | 0.76 | 1.50×10^-9^ | 9.84×10^-6^ | 6 | 11494909 |  |
| cg20284834 | 0.24 | 4.48×10^-8^ | 2.19×10^-4^ | 6 | 15499615 | *JARID2* |
| cg21305774 | -0.61 | 6.56×10^-11^ | 5.75×10^-7^ | 6 | 17729087 |  |
| cg02565494 | -0.48 | 1.38×10^-7^ | 6.04×10^-4^ | 6 | 25287859 | *LRRC16A* |
| cg07191121 | -0.68 | 1.23×10^-7^ | 5.48×10^-4^ | 6 | 28724650 |  |
| cg00609363 | -0.37 | 2.55×10^-9^ | 1.62×10^-5^ | 6 | 30028955 | *ZNRD1* |
| cg26905324 | -0.17 | 6.26×10^-8^ | 2.98×10^-4^ | 6 | 31510332 | *SNORD84* |
| cg16918667 | -0.32 | 8.21×10^-6^ | 2.02×10^-2^ | 6 | 31827573 | *NEU1* |
| cg03083146 | -0.54 | 1.30×10^-10^ | 1.09×10^-6^ | 6 | 32781235 | *HLA-DOB* |
| cg13347198 | -0.70 | 2.20×10^-14^ | 5.39×10^-10^ | 6 | 32798198 | *TAP2* |
| cg20102583 | -0.54 | 2.81×10^-24^ | 2.02×10^-19^ | 6 | 44171055 |  |
| cg15347924 | 0.52 | 1.53×10^-5^ | 3.41×10^-2^ | 6 | 74073424 | *C6orf221* |
| cg22011809 | -0.36 | 5.07×10^-9^ | 3.09×10^-5^ | 6 | 105605934 | *POPDC3* |
| cg12267800 | -0.42 | 2.37×10^-7^ | 9.64×10^-4^ | 6 | 106240391 |  |
| cg12801619 | 0.25 | 3.54×10^-6^ | 9.74×10^-3^ | 6 | 108976918 | *FOXO3* |
| cg01999537 | -0.27 | 1.64×10^-6^ | 5.11×10^-3^ | 6 | 111176837 | *AMD1* |
| cg17679781 | 0.43 | 5.58×10^-10^ | 4.11×10^-6^ | 6 | 114663223 |  |
| cg05051393 | -0.71 | 3.18×10^-11^ | 3.12×10^-7^ | 6 | 119228846 | *ASF1A* |
| cg09035093 | 0.28 | 3.01×10^-6^ | 8.49×10^-3^ | 6 | 132181821 | *ENPP1* |
| cg24086611 | 0.72 | 1.15×10^-12^ | 1.52×10^-8^ | 6 | 136394121 | *PDE7B* |
| cg12117518 | -0.47 | 5.52×10^-8^ | 2.66×10^-4^ | 6 | 143234024 | *HIVEP2* |
| cg12227626 | 0.36 | 3.92×10^-14^ | 9.03×10^-10^ | 6 | 151196753 | *MTHFD1L* |
| cg21998840 | 0.72 | 3.42×10^-12^ | 3.88×10^-8^ | 7 | 2212030 | *MAD1L1* |
| cg21229153 | 0.60 | 1.13×10^-5^ | 2.65×10^-2^ | 7 | 2518188 |  |
| cg21503490 | 0.30 | 3.60×10^-11^ | 3.49×10^-7^ | 7 | 6773787 | *PMS2CL* |
| cg06327962 | 0.13 | 2.45×10^-13^ | 4.16×10^-9^ | 7 | 8009176 | *GLCCI1* |
| cg11876022 | 0.38 | 3.31×10^-6^ | 9.27×10^-3^ | 7 | 19812479 | *TMEM196* |
| cg21657955 | 0.41 | 1.70×10^-7^ | 7.27×10^-4^ | 7 | 19812578 | *TMEM196* |
| cg25184481 | 0.22 | 2.39×10^-5^ | 4.99×10^-2^ | 7 | 19813001 | *TMEM196* |
| cg09798023 | 0.23 | 5.03×10^-6^ | 1.34×10^-2^ | 7 | 27161624 | *HOXA3* |
| cg15410169 | -1.00 | 1.35×10^-14^ | 3.56×10^-10^ | 7 | 29518429 | *CHN2* |
| cg09847222 | -0.39 | 1.86×10^-6^ | 5.70×10^-3^ | 7 | 47348487 | *TNS3* |
| cg18189615 | -0.66 | 2.01×10^-13^ | 3.57×10^-9^ | 7 | 75386749 |  |
| cg07098123 | 0.56 | 2.19×10^-5^ | 4.63×10^-2^ | 7 | 91507250 | *MTERF* |
| cg26724642 | -1.00 | 2.59×10^-40^ | 9.53×10^-35^ | 7 | 97895513 |  |
| cg15703422 | 0.36 | 2.67×10^-8^ | 1.38×10^-4^ | 7 | 100176855 | *LRCH4* |
| cg24193325 | 0.58 | 2.92×10^-8^ | 1.46×10^-4^ | 7 | 100184756 | *LRCH4* |
| cg27084965 | 0.20 | 4.12×10^-6^ | 1.12×10^-2^ | 7 | 103871917 |  |
| cg27019749 | 0.43 | 1.83×10^-6^ | 5.64×10^-3^ | 7 | 112635781 | *HRAT17* |
| cg14937748 | -0.50 | 1.62×10^-8^ | 8.99×10^-5^ | 7 | 117578339 |  |
| cg13504856 | -0.68 | 3.86×10^-12^ | 4.13×10^-8^ | 7 | 128956859 | *AHCYL2* |
| cg07683330 | -0.57 | 7.30×10^-14^ | 1.54×10^-9^ | 7 | 130125390 | *MEST* |
| cg21963683 | -0.56 | 8.95×10^-13^ | 1.22×10^-8^ | 7 | 139754071 | *PARP12* |
| cg20419410 | 0.24 | 1.72×10^-29^ | 2.11×10^-24^ | 7 | 155089803 | *INSIG1* |
| cg05470643 | -0.66 | 1.45×10^-7^ | 6.26×10^-4^ | 7 | 155579830 |  |
| cg02694994 | -1.00 | 3.20×10^-13^ | 5.11×10^-9^ | 7 | 157612661 | *PTPRN2* |
| cg10414350 | 0.31 | 6.39×10^-6^ | 1.65×10^-2^ | 8 | 10210282 | *MSRA* |
| cg21341889 | 0.26 | 1.70×10^-5^ | 3.71×10^-2^ | 8 | 17773367 |  |
| cg23523780 | 0.25 | 5.19×10^-8^ | 2.52×10^-4^ | 8 | 22972234 | *TNFRSF10C* |
| cg12148515 | 0.90 | 1.69×10^-12^ | 2.15×10^-8^ | 8 | 41517301 | *ANK1* |
| cg07085824 | -0.09 | 1.05×10^-6^ | 3.47×10^-3^ | 8 | 42948105 | *SGK196* |
| cg03214200 | -0.67 | 1.31×10^-8^ | 7.55×10^-5^ | 8 | 65553669 | *CYP7B1* |
| cg17680873 | -1.00 | 9.34×10^-13^ | 1.25×10^-8^ | 8 | 81878186 |  |
| cg19333493 | -0.48 | 1.22×10^-5^ | 2.80×10^-2^ | 8 | 97401115 | *LOC102724804* |
| cg22516182 | -0.65 | 1.39×10^-7^ | 6.04×10^-4^ | 8 | 102400152 |  |
| cg21128963 | 0.53 | 1.50×10^-5^ | 3.35×10^-2^ | 8 | 103307352 | *UBR5* |
| cg27163126 | -0.38 | 3.49×10^-6^ | 9.66×10^-3^ | 8 | 103953809 | *AZIN1-AS1* |
| cg07229027 | -0.55 | 4.12×10^-17^ | 1.60×10^-12^ | 8 | 124408743 | *ATAD2* |
| cg15171273 | 0.33 | 7.95×10^-11^ | 6.89×10^-7^ | 8 | 131539725 |  |
| cg26217694 | 0.52 | 5.18×10^-6^ | 1.37×10^-2^ | 8 | 132532575 |  |
| cg15969189 | 0.49 | 2.41×10^-7^ | 9.65×10^-4^ | 8 | 134745691 |  |
| cg15463309 | -0.96 | 3.73×10^-12^ | 4.04×10^-8^ | 8 | 141598157 | *EIF2C2* |
| cg01744949 | -0.47 | 4.08×10^-11^ | 3.90×10^-7^ | 9 | 2467204 |  |
| cg01853552 | -0.23 | 3.73×10^-6^ | 1.02×10^-2^ | 9 | 78378884 |  |
| cg19282250 | 0.44 | 8.75×10^-8^ | 4.03×10^-4^ | 9 | 79328522 | *PRUNE2* |
| cg13980266 | 0.20 | 3.72×10^-6^ | 1.02×10^-2^ | 9 | 97022269 | *ZNF169* |
| cg06853088 | -0.47 | 1.03×10^-8^ | 6.10×10^-5^ | 9 | 109040403 |  |
| cg15221416 | 0.95 | 1.97×10^-13^ | 3.57×10^-9^ | 9 | 125021726 | *RBM18* |
| cg17618153 | 0.97 | 2.35×10^-26^ | 2.47×10^-21^ | 9 | 129874991 | *ANGPTL2* |
| cg14583686 | -0.50 | 1.38×10^-25^ | 1.27×10^-20^ | 9 | 130226589 | *LRSAM1* |
| cg13429719 | 0.40 | 1.07×10^-5^ | 2.51×10^-2^ | 9 | 130327532 | *FAM129B* |
| cg14165241 | 0.17 | 2.40×10^-6^ | 7.17×10^-3^ | 9 | 131315156 | *SPTAN1* |
| cg13521319 | 0.31 | 4.28×10^-9^ | 2.65×10^-5^ | 9 | 133423844 |  |
| cg02746771 | -0.41 | 4.88×10^-9^ | 3.00×10^-5^ | 10 | 527872 | *DIP2C* |
| cg21389456 | 0.56 | 6.76×10^-8^ | 3.17×10^-4^ | 10 | 675937 | *DIP2C* |
| cg02773337 | 0.13 | 7.70×10^-6^ | 1.92×10^-2^ | 10 | 3171970 | *PFKP* |
| cg05686843 | -0.34 | 6.69×10^-6^ | 1.69×10^-2^ | 10 | 6111408 |  |
| cg12377641 | 0.36 | 9.99×10^-6^ | 2.37×10^-2^ | 10 | 11868179 | *C10orf47* |
| cg12629936 | -0.27 | 1.36×10^-5^ | 3.05×10^-2^ | 10 | 12250941 | *CDC123* |
| cg15882987 | -0.64 | 2.84×10^-21^ | 1.39×10^-16^ | 10 | 16790575 | *RSU1* |
| cg08581745 | 0.23 | 4.40×10^-7^ | 1.64×10^-3^ | 10 | 17050868 | *CUBN* |
| cg14196028 | -0.55 | 1.23×10^-13^ | 2.41×10^-9^ | 10 | 69523483 |  |
| cg13398715 | 0.81 | 6.63×10^-7^ | 2.36×10^-3^ | 10 | 72692264 |  |
| cg01390840 | 0.39 | 1.51×10^-11^ | 1.51×10^-7^ | 10 | 76067347 | *ADK* |
| cg17960717 | 0.76 | 4.15×10^-8^ | 2.04×10^-4^ | 10 | 76569871 |  |
| cg06706894 | -0.57 | 5.92×10^-7^ | 2.15×10^-3^ | 10 | 80167002 |  |
| cg01536438 | -0.61 | 3.09×10^-23^ | 1.90×10^-18^ | 10 | 90712625 | *ACTA2* |
| cg06093253 | -0.40 | 3.71×10^-12^ | 4.04×10^-8^ | 10 | 100194230 | *HPS1* |
| cg10756593 | 0.62 | 9.30×10^-9^ | 5.57×10^-5^ | 10 | 104591244 | *CYP17A1* |
| cg11416669 | 0.45 | 6.16×10^-6^ | 1.59×10^-2^ | 10 | 120069000 | *C10orf84* |
| cg16499947 | -0.49 | 4.71×10^-11^ | 4.45×10^-7^ | 10 | 123329113 | *FGFR2* |
| cg25239156 | 0.66 | 1.94×10^-5^ | 4.14×10^-2^ | 10 | 131566948 |  |
| cg18615506 | 0.32 | 2.48×10^-13^ | 4.16×10^-9^ | 10 | 134177612 | *LRRC27* |
| cg11862565 | 0.68 | 3.04×10^-6^ | 8.54×10^-3^ | 10 | 134832196 |  |
| cg23152617 | 0.29 | 3.54×10^-6^ | 9.74×10^-3^ | 10 | 135104676 | *TUBGCP2* |
| cg27010328 | -0.63 | 2.61×10^-12^ | 3.00×10^-8^ | 11 | 238081 | *PSMD13* |
| cg16674116 | 0.43 | 1.06×10^-7^ | 4.76×10^-4^ | 11 | 1319689 | *TOLLIP* |
| cg06080571 | 0.71 | 2.15×10^-14^ | 5.39×10^-10^ | 11 | 2349807 |  |
| cg26344859 | 0.83 | 4.47×10^-6^ | 1.20×10^-2^ | 11 | 2584629 | *KCNQ1* |
| cg00055860 | 0.41 | 1.35×10^-9^ | 9.15×10^-6^ | 11 | 5951424 |  |
| cg01494614 | -0.69 | 3.93×10^-12^ | 4.14×10^-8^ | 11 | 10562837 | *RNF141* |
| cg03544918 | -0.16 | 2.35×10^-7^ | 9.63×10^-4^ | 11 | 14913944 | *CYP2R1* |
| cg16544887 | -0.14 | 8.82×10^-7^ | 3.01×10^-3^ | 11 | 14913979 | *CYP2R1* |
| cg00455178 | -0.18 | 1.25×10^-11^ | 1.26×10^-7^ | 11 | 14913981 | *CYP2R1* |
| cg11980188 | -0.18 | 3.38×10^-10^ | 2.60×10^-6^ | 11 | 14914012 | *CYP2R1* |
| cg07530325 | 0.57 | 6.66×10^-6^ | 1.69×10^-2^ | 11 | 17444992 | *ABCC8* |
| cg14578028 | -0.45 | 1.86×10^-7^ | 7.78×10^-4^ | 11 | 33796289 | *FBXO3* |
| cg05079837 | 0.30 | 5.94×10^-7^ | 2.15×10^-3^ | 11 | 45889072 | *CRY2* |
| cg11558328 | 0.29 | 9.31×10^-6^ | 2.24×10^-2^ | 11 | 46722331 | *ARHGAP1* |
| cg22189307 | 0.13 | 1.08×10^-6^ | 3.50×10^-3^ | 11 | 61061699 | *VWCE* |
| cg09098150 | 0.27 | 6.80×10^-7^ | 2.41×10^-3^ | 11 | 61216180 |  |
| cg05721771 | 0.27 | 1.60×10^-6^ | 5.00×10^-3^ | 11 | 76910708 | *MYO7A* |
| cg08402652 | 0.31 | 7.67×10^-7^ | 2.65×10^-3^ | 11 | 94501461 | *AMOTL1* |
| cg13186466 | -0.56 | 6.68×10^-6^ | 1.69×10^-2^ | 11 | 111957596 | *TIMM8B* |
| cg13485335 | 0.60 | 2.84×10^-8^ | 1.44×10^-4^ | 11 | 120161548 | *POU2F3* |
| cg02921249 | -0.23 | 1.24×10^-5^ | 2.84×10^-2^ | 11 | 125483986 | *STT3A* |
| cg18578939 | -0.39 | 5.11×10^-11^ | 4.77×10^-7^ | 12 | 2919557 |  |
| cg04636406 | 0.25 | 1.50×10^-12^ | 1.94×10^-8^ | 12 | 6993009 | *RPL13P5* |
| cg10201141 | 0.62 | 3.35×10^-6^ | 9.32×10^-3^ | 12 | 7032793 | *ENO2* |
| cg14437324 | 0.47 | 2.29×10^-12^ | 2.77×10^-8^ | 12 | 31812558 | *C12orf72* |
| cg05705035 | 0.55 | 1.37×10^-13^ | 2.59×10^-9^ | 12 | 31812580 | *C12orf72* |
| cg17849509 | -0.47 | 1.61×10^-5^ | 3.57×10^-2^ | 12 | 59151977 |  |
| cg27615095 | 0.39 | 2.01×10^-5^ | 4.28×10^-2^ | 12 | 63049980 | *PPM1H* |
| cg05136471 | 0.32 | 2.41×10^-7^ | 9.65×10^-4^ | 12 | 94577837 | *PLXNC1* |
| cg18907202 | 0.27 | 1.66×10^-5^ | 3.66×10^-2^ | 12 | 94853658 | *CCDC41* |
| cg22874494 | -0.51 | 2.67×10^-9^ | 1.68×10^-5^ | 12 | 105381266 | *C12orf45* |
| cg14079445 | -0.63 | 8.53×10^-23^ | 4.84×10^-18^ | 12 | 111493997 | *CUX2* |
| cg11771936 | 0.37 | 4.62×10^-6^ | 1.24×10^-2^ | 12 | 120602003 | *GCN1* |
| cg04150382 | 0.39 | 2.54×10^-8^ | 1.34×10^-4^ | 12 | 121533801 |  |
| cg15244603 | 0.33 | 6.93×10^-9^ | 4.19×10^-5^ | 12 | 125607520 | *AACS* |
| cg26977086 | -0.69 | 7.72×10^-8^ | 3.60×10^-4^ | 12 | 132329805 | *MMP17* |
| cg10788674 | -0.12 | 2.36×10^-15^ | 6.96×10^-11^ | 12 | 133214645 | *POLE* |
| cg00666749 | 0.55 | 5.04×10^-6^ | 1.34×10^-2^ | 13 | 21637118 | *LATS2* |
| cg25745379 | -0.15 | 9.67×10^-7^ | 3.27×10^-3^ | 13 | 27333481 | *GPR12* |
| cg10362742 | 0.30 | 5.39×10^-6^ | 1.41×10^-2^ | 13 | 31736223 | *HSPH1* |
| cg03795663 | -0.20 | 1.52×10^-5^ | 3.39×10^-2^ | 13 | 42188218 | *VWA8* |
| cg25348433 | 0.27 | 1.61×10^-8^ | 8.99×10^-5^ | 13 | 78519244 | *EDNRB* |
| cg15858426 | -0.29 | 2.63×10^-6^ | 7.66×10^-3^ | 13 | 95252369 |  |
| cg10769343 | -0.29 | 1.97×10^-6^ | 6.01×10^-3^ | 14 | 24617188 | *PSME2* |
| cg17864209 | 0.37 | 4.32×10^-7^ | 1.62×10^-3^ | 14 | 35805102 |  |
| cg06243610 | 0.30 | 3.47×10^-8^ | 1.73×10^-4^ | 14 | 52373839 | *GNG2* |
| cg08606441 | -0.57 | 1.06×10^-9^ | 7.36×10^-6^ | 14 | 55142748 | *SAMD4A* |
| cg00100184 | -0.38 | 1.82×10^-12^ | 2.25×10^-8^ | 14 | 56014589 |  |
| cg24057218 | -0.42 | 2.42×10^-7^ | 9.65×10^-4^ | 14 | 68660680 | *RAD51L1* |
| cg02192794 | 0.31 | 2.47×10^-6^ | 7.31×10^-3^ | 14 | 73409967 | *DCAF4* |
| cg14519717 | 0.35 | 2.77×10^-8^ | 1.42×10^-4^ | 14 | 90744790 | *C14orf102* |
| cg24547356 | 0.04 | 2.36×10^-5^ | 4.96×10^-2^ | 14 | 103995608 | *TRMT61A* |
| cg26995364 | -0.71 | 5.75×10^-13^ | 8.15×10^-9^ | 15 | 29336425 | *APBA2* |
| cg18157976 | 0.30 | 1.04×10^-6^ | 3.44×10^-3^ | 15 | 33166054 | *FMN1* |
| cg24245086 | 0.79 | 4.98×10^-10^ | 3.74×10^-6^ | 15 | 44164534 | *FRMD5* |
| cg22134372 | -0.40 | 9.20×10^-17^ | 3.39×10^-12^ | 15 | 52455211 | *GNB5* |
| cg09293769 | -0.56 | 2.75×10^-7^ | 1.08×10^-3^ | 15 | 52728704 | *MYO5A* |
| cg07503408 | -0.48 | 1.06×10^-8^ | 6.27×10^-5^ | 15 | 59496592 | *MYO1E* |
| cg10476347 | -0.54 | 1.18×10^-6^ | 3.80×10^-3^ | 15 | 64676664 |  |
| cg12329286 | 0.52 | 2.62×10^-8^ | 1.37×10^-4^ | 15 | 66024890 | *DENND4A* |
| cg15100456 | -0.28 | 1.60×10^-8^ | 8.98×10^-5^ | 15 | 68809904 |  |
| cg27514624 | -0.33 | 4.95×10^-7^ | 1.82×10^-3^ | 15 | 69706456 | *KIF23* |
| cg03841750 | -1.00 | 2.83×10^-16^ | 9.66×10^-12^ | 15 | 70244882 |  |
| cg11637053 | 0.23 | 3.01×10^-7^ | 1.17×10^-3^ | 15 | 78432103 |  |
| cg20788083 | 0.29 | 1.94×10^-6^ | 5.92×10^-3^ | 15 | 91566802 | *VPS33B* |
| cg06831439 | 0.21 | 1.17×10^-5^ | 2.71×10^-2^ | 15 | 92509585 | *SLCO3A1* |
| cg16336121 | -0.30 | 6.09×10^-6^ | 1.58×10^-2^ | 15 | 93469019 | *CHD2* |
| cg24524702 | 0.28 | 1.82×10^-8^ | 1.00×10^-4^ | 15 | 100109440 | *MEF2A* |
| cg27040115 | -0.47 | 3.95×10^-7^ | 1.49×10^-3^ | 15 | 101168559 | *ASB7* |
| cg13469949 | -0.54 | 6.67×10^-8^ | 3.15×10^-4^ | 16 | 556212 | *RAB11FIP3* |
| cg14939446 | -0.27 | 7.24×10^-10^ | 5.23×10^-6^ | 16 | 2571015 | *AMDHD2* |
| cg16468174 | -0.57 | 1.67×10^-5^ | 3.66×10^-2^ | 16 | 3177234 |  |
| cg00655143 | 0.45 | 5.39×10^-11^ | 4.96×10^-7^ | 16 | 3985958 |  |
| cg02196379 | 0.27 | 1.32×10^-5^ | 2.99×10^-2^ | 16 | 16215875 | *ABCC1* |
| cg20438949 | -0.12 | 1.02×10^-5^ | 2.42×10^-2^ | 16 | 28834126 | *ATXN2L* |
| cg00570642 | 0.27 | 1.71×10^-5^ | 3.71×10^-2^ | 16 | 30022576 | *DOC2A* |
| cg00131272 | -0.51 | 1.07×10^-24^ | 8.78×10^-20^ | 16 | 46957465 | *GPT2* |
| cg20397142 | 0.26 | 5.62×10^-7^ | 2.05×10^-3^ | 16 | 57494926 | *COQ9* |
| cg16053428 | 0.20 | 5.02×10^-6^ | 1.34×10^-2^ | 16 | 83990544 | *OSGIN1* |
| cg10274403 | 0.27 | 1.80×10^-5^ | 3.86×10^-2^ | 16 | 84178812 | *LRRC50* |
| cg16791721 | 0.13 | 1.16×10^-5^ | 2.68×10^-2^ | 17 | 259991 | *C17orf97* |
| cg20197989 | 0.39 | 2.39×10^-12^ | 2.84×10^-8^ | 17 | 260058 | *C17orf97* |
| cg19214977 | -0.48 | 9.91×10^-6^ | 2.36×10^-2^ | 17 | 2777960 | *RAP1GAP2* |
| cg11295144 | 0.40 | 1.49×10^-10^ | 1.24×10^-6^ | 17 | 33447001 | *RAD51L3* |
| cg15487375 | 0.31 | 1.60×10^-6^ | 5.00×10^-3^ | 17 | 38105237 |  |
| cg02859521 | 0.32 | 5.66×10^-10^ | 4.13×10^-6^ | 17 | 43332672 | *MAP3K14-AS1* |
| cg24142633 | 0.34 | 7.29×10^-7^ | 2.55×10^-3^ | 17 | 54893277 | *C17orf67* |
| cg16901896 | -0.75 | 7.72×10^-6^ | 1.92×10^-2^ | 17 | 59914735 | *BRIP1* |
| cg11988568 | 0.26 | 2.13×10^-5^ | 4.52×10^-2^ | 17 | 78518917 | *RPTOR* |
| cg22882093 | 0.33 | 2.71×10^-6^ | 7.86×10^-3^ | 17 | 80158055 | *CCDC57* |
| cg12306367 | 0.23 | 2.28×10^-6^ | 6.85×10^-3^ | 18 | 19777305 | *GATA6* |
| cg19900989 | -0.36 | 5.30×10^-7^ | 1.94×10^-3^ | 18 | 42321831 | *SETBP1* |
| cg04870412 | -0.28 | 3.74×10^-7^ | 1.43×10^-3^ | 18 | 54655403 | *WDR7* |
| cg26635170 | -0.25 | 2.33×10^-6^ | 6.97×10^-3^ | 18 | 55907926 | *NEDD4L* |
| cg14887576 | -0.90 | 1.19×10^-9^ | 8.22×10^-6^ | 18 | 59058725 |  |
| cg05535853 | 0.39 | 3.14×10^-10^ | 2.44×10^-6^ | 18 | 74781719 | *MBP* |
| cg06168021 | 0.46 | 2.15×10^-8^ | 1.15×10^-4^ | 18 | 75399320 |  |
| cg01794046 | -0.36 | 1.15×10^-5^ | 2.67×10^-2^ | 19 | 578648 | *BSG* |
| cg03437204 | 0.22 | 9.99×10^-7^ | 3.33×10^-3^ | 19 | 1401372 | *GAMT* |
| cg04742763 | -0.63 | 6.20×10^-11^ | 5.63×10^-7^ | 19 | 13364862 | *CACNA1A* |
| cg20276511 | 0.08 | 9.79×10^-8^ | 4.43×10^-4^ | 19 | 15543527 | *WIZ* |
| cg10819847 | 0.18 | 6.63×10^-6^ | 1.69×10^-2^ | 19 | 16435375 | *KLF2* |
| cg18025793 | 0.44 | 1.99×10^-9^ | 1.29×10^-5^ | 19 | 17097121 | *CPAMD8* |
| cg19788600 | 0.17 | 9.47×10^-6^ | 2.27×10^-2^ | 19 | 21688828 | *ZNF429* |
| cg02478247 | 0.61 | 8.73×10^-7^ | 2.99×10^-3^ | 19 | 23941207 | *ZNF681* |
| cg10105623 | -0.47 | 3.02×10^-24^ | 2.02×10^-19^ | 19 | 34893958 | *PDCD2L* |
| cg17126929 | -0.55 | 5.56×10^-13^ | 8.04×10^-9^ | 19 | 38781995 | *SPINT2* |
| cg23026246 | -0.23 | 2.72×10^-7^ | 1.07×10^-3^ | 19 | 41029387 | *SPTBN4* |
| cg26020365 | -0.36 | 2.21×10^-8^ | 1.17×10^-4^ | 19 | 44932260 | *ZNF229* |
| cg13566596 | -1.00 | 8.90×10^-22^ | 4.68×10^-17^ | 19 | 45033572 | *CEACAM20* |
| cg24021890 | 0.20 | 2.20×10^-15^ | 6.75×10^-11^ | 19 | 52552120 | *ZNF432* |
| cg14944575 | 0.22 | 5.38×10^-10^ | 4.00×10^-6^ | 19 | 52552197 | *ZNF432* |
| cg13584718 | 0.22 | 1.84×10^-10^ | 1.49×10^-6^ | 19 | 52552383 | *ZNF432* |
| cg02729352 | 0.12 | 8.00×10^-6^ | 1.98×10^-2^ | 19 | 52552443 | *ZNF432* |
| cg11919525 | 0.17 | 2.21×10^-7^ | 9.11×10^-4^ | 19 | 52552461 | *ZNF432* |
| cg00289084 | 0.52 | 1.37×10^-9^ | 9.20×10^-6^ | 20 | 3828030 | *MAVS* |
| cg13989964 | 0.30 | 1.22×10^-6^ | 3.91×10^-3^ | 20 | 10595286 | *SLX4IP* |
| cg17579287 | -1.00 | 5.34×10^-6^ | 1.40×10^-2^ | 20 | 23326847 |  |
| cg16326902 | -0.54 | 1.72×10^-7^ | 7.31×10^-4^ | 20 | 49253364 | *FAM65C* |
| cg02712555 | 0.09 | 1.54×10^-6^ | 4.85×10^-3^ | 20 | 52790733 | *CYP24A1* |
| cg09124152 | -0.38 | 9.78×10^-7^ | 3.29×10^-3^ | 21 | 16230738 |  |
| cg01158367 | -0.57 | 4.10×10^-10^ | 3.12×10^-6^ | 21 | 30258251 | *N6AMT1* |
| cg06561442 | -0.36 | 1.21×10^-9^ | 8.28×10^-6^ | 21 | 31125799 | *GRIK1-AS1* |
| cg06253164 | -0.27 | 1.30×10^-5^ | 2.96×10^-2^ | 21 | 33346276 | *HUNK* |
| cg02292859 | 0.48 | 7.65×10^-7^ | 2.65×10^-3^ | 21 | 34924526 | *SON* |
| cg18574499 | 0.26 | 6.63×10^-7^ | 2.36×10^-3^ | 21 | 40669669 | *BRWD1* |
| cg12231697 | 0.38 | 4.39×10^-6^ | 1.18×10^-2^ | 21 | 47666788 | *MCM3AP* |
| cg14740204 | 0.18 | 2.15×10^-8^ | 1.15×10^-4^ | 22 | 24236549 | *MIF* |
| cg25903490 | 0.40 | 2.03×10^-13^ | 3.57×10^-9^ | 22 | 25477333 | *KIAA1671* |
| cg08202043 | 0.25 | 6.47×10^-6^ | 1.66×10^-2^ | 22 | 26990180 |  |
| cg16160151 | 0.25 | 1.81×10^-7^ | 7.63×10^-4^ | 22 | 36663868 |  |
| cg23106733 | -0.64 | 2.88×10^-16^ | 9.66×10^-12^ | 22 | 38325762 | *MICALL1* |
| cg03707464 | -1.00 | 3.26×10^-13^ | 5.11×10^-9^ | 22 | 43842664 | *MPPED1* |
| cg08762150 | 0.25 | 4.62×10^-7^ | 1.72×10^-3^ | 22 | 44644994 | *KIAA1644* |
| cg27528247 | 0.58 | 1.27×10^-6^ | 4.07×10^-3^ | 22 | 51045178 | *MAPK8IP2* |

Chr: chromosome; CpG: cytosine-phosphate-guanine dinucleotide; FDR: false discovery rate.

^a^Methylation levels of gout-methylation levels of non-gout in various cell lineages after adjusting for age, sex, alcohol drinking, smoking status, smoking history (total pack-years) and cell fractions with CellDMC.

^b^Positions of the CpG sites in hg19.

^c^Gene names provided in EPIC array. For CpG probes located in intergenic region, no corresponding gene names are provided in EPIC array.

**Supplementary Table 3. Failed monocyte-specific methylation sites which regulates interleukin-1β (IL-1β).**

| CpG site | Δβ^a^ | *P* | FDR | Chr | Position^b^ | Gene (Alias) | Genomic features | CpG features | References^c^ |
| --- | --- | --- | --- | --- | --- | --- | --- | --- | --- |
| **Increases/decreases interleukin-1β (IL-1β) in monocyte/macrophage lineage cells** | | | | | | | | | |
| cg18886702 | -0.68 | 8.48×10^-11^ | 1.20×10^-6^ | 1 | 67658441 | *IL23R* | Body | open sea | 🡩 IL-1β [1] |
| cg00091098 | 0.52 | 1.06×10^-7^ | 6.32×10^-4^ | 3 | 71100993 | *FOXP1* | Body | open sea | 🡫 IL-1β [2] |
| cg14326053 | -0.75 | 1.50×10^-8^ | 1.18×10^-4^ | 20 | 371146 | *TRIB3*(*TRB3*) [3] | Body | open sea | 🡫 IL-1β [Figure 4B of 4] |
| cg10027934 | -0.94 | 1.27×10^-7^ | 7.31×10^-4^ | 22 | 39799092 | *MAP3K7IP1*(*TAB1*) [5] | Body | shelf | 🡩 IL-1β [Figure 4B of 6] |
| **Increases IL-1β and expressed in monocyte/macrophage lineage cells** | | | | | | | | | |
| cg22408430 | -0.93 | 4.66×10^-8^ | 3.18×10^-4^ | 11 | 63883266 | *MACROD1*(*LRP16*) [7] | Body | shore | 🡩 IL-1β [Figure 3-4 of 7, Supplementary Table 1 of 8] |
| cg13204333 | -1.00 | 5.13×10^-8^ | 3.47×10^-4^ | 18 | 58041198 | *MC4R* | TSS1500 | open sea | 🡩 IL-1β [Fiugure 3 of 9, 10] |

Chr: chromosome; CpG: cytosine-phosphate-guanine dinucleotide; FDR: false discovery rate.

^a^Methylation levels of gout-methylation levels of non-gout in monocytes after adjusting for age, sex, alcohol drinking, smoking status, smoking history (total pack-years) and cell fractions with CellDMC.

^b^Positions of the CpG sites in hg19.

^c^References about genes in relationship to IL-1β production and supporting its expression in monocyte/macrophage lineage cells.

**Reference**

1. Sun R, Hedl M, Abraham C. IL23 induces IL23R recycling and amplifies innate receptor-induced signalling and cytokines in human macrophages, and the IBD-protective IL23R R381Q variant modulates these outcomes. Gut. 2020; 69:264-73.

2. Shi C, Sakuma M, Mooroka T, Liscoe A, Gao H, Croce KJ, Sharma A, Kaplan D, Greaves DR, Wang Y, Simon DI. Down-regulation of the forkhead transcription factor Foxp1 is required for monocyte differentiation and macrophage function. Blood. 2008; 112:4699-711.

3. Tsai DH, Chung CH, Lee KT. Antrodia cinnamomea induces autophagic cell death via the CHOP/TRB3/Akt/mTOR pathway in colorectal cancer cells. Sci Rep. 2018; 8:17424.

4. Steverson D, Tian L, Fu Y, Zhang W, Ma E, Garvey WT. Tribbles Homolog 3 Promotes Foam Cell Formation Associated with Decreased Proinflammatory Cytokine Production in Macrophages: Evidence for Reciprocal Regulation of Cholesterol Uptake and Inflammation. Metab Syndr Relat Disord. 2016; 14:7-15.

5. Bollino D, Colunga A, Li B, Aurelian L. ΔPK oncolytic activity includes modulation of the tumour cell milieu. J Gen Virol. 2016; 97:496-508.

6. Chen H, Liu Y, Li D, Song J, Xia M. PGC-1β suppresses saturated fatty acid-induced macrophage inflammation by inhibiting TAK1 activation. IUBMB Life. 2016; 68:145-55.

7. Zang L, Hong Q, Yang G, Gu W, Wang A, Dou J, Mu Y, Wu D, Lyu Z. MACROD1/LRP16 Enhances LPS-Stimulated Inflammatory Responses by Up-Regulating a Rac1-Dependent Pathway in Adipocytes. Cell Physiol Biochem. 2018; 51:2591-603.

8. Johnston JM, Angyal A, Bauer RC, Hamby S, Suvarna SK, Baidžajevas K, Hegedus Z, Dear TN, Turner M, Wilson HL, Goodall AH, Rader DJ, Shoulders CC, et al. Myeloid Tribbles 1 induces early atherosclerosis via enhanced foam cell expansion. Sci Adv. 2019; 5:eaax9183.

9. Zhao Y, Xin Y, Chu H. MC4R Is Involved in Neuropathic Pain by Regulating JNK Signaling Pathway After Chronic Constriction Injury. Front Neurosci. 2019; 13:919.

10. Carniglia L, Durand D, Caruso C, Lasaga M. Effect of NDP-α-MSH on PPAR-γ and -β expression and anti-inflammatory cytokine release in rat astrocytes and microglia. PLoS One. 2013; 8:e57313.

| **Supplementary Table 4. Monocyte-specific methylation sites located in genes not regulating interleukin-1β (IL-1β).** | | | | | | | | | |
| --- | --- | --- | --- | --- | --- | --- | --- | --- | --- |
| CpG site | Δβ^a^ | *P* | FDR | Chr | Position^b^ | Gene (Alias) | Genomic features | CpG features | Biologic functions |
| cg15158067 | -1.00 | 1.24×10^-6^ | 4.89×10^-3^ | 1 | 1536396 | *C1orf233* | TSS1500 | shore | unknown |
| cg01718853 | 0.51 | 1.54×10^-5^ | 4.02×10^-2^ | 1 | 27157161 | *ZDHHC18* | Body | shelf | regulates proneural-to-mesenchymal transition and associated with schizophrenia [1-2] |
| cg03789579 | -1.00 | 1.35×10^-9^ | 1.40×10^-5^ | 1 | 32670241 | *IQCC* | TSS1500 | shore | associated with breast cancer [3] |
| cg13559233 | -1.00 | 8.38×10^-6^ | 2.42×10^-2^ | 1 | 82269258 | *LPHN2* (*ADGRL2*) [4] | 5'UTR | shore | implicated in neurodevelopment [4-6], cocaine use [7], lung function [8], oral submucous fibrosis [9], cleft lip and/or cleft palate [10], cancer chemoresistance [11], subcutaneous fat thickness [12], learning [13], and disease severity [14] |
| cg05638359 | 1.00 | 8.51×10^-8^ | 5.27×10^-4^ | 1 | 190444387 | *FAM5C* (*BRINP3*) [15] | 5'UTR | open sea | modulates cell proliferation, migration, invasion [16] and differentiation [17], regulates norepinephrine [18], cardiovascular development and disease [15,19-20], cancer survival [21] and implicated in aggressive periodontitis [22] and fracture non-union [23] |
| cg03945122 | 1.00 | 7.40×10^-10^ | 8.02×10^-6^ | 1 | 207840521 | *CR1L* | Body | shelf | unknown |
| cg26461510 | -0.79 | 2.09×10^-7^ | 1.05×10^-3^ | 1 | 248569107 | *OR2T1* | TSS200 | open sea | unknown |
| cg03275949 | 1.00 | 3.17×10^-11^ | 4.94×10^-7^ | 2 | 28113605 | *BRE-AS1* | Body | island | regulates cell proliferation and apoptosis [24-25] |
| cg07083023 | -0.97 | 1.23×10-5 | 3.31×10^-2^ | 2 | 51254981 | *NRXN1* | Body | island | implicated in various aspects of neurodevelopment [26-32] and numerous neuropsychiatric disorders [33-48], smoking addiction [49], psychiatric disorder treatment response [50-51], astigmatism [52], neoplasm [53-54], physical trait [55] and serum coenzyme Q10 levels [56] |
| cg19824059 | -1.00 | 4.53×10^-8^ | 3.12×10^-4^ | 2 | 51255324 | *NRXN1* | Body | island |
| cg08797047 | -0.88 | 1.12×10^-5^ | 3.09×10^-2^ | 2 | 144359042 | *ARHGAP15* | Body | open sea | regulates Rac/GTPase signaling [57-61], participates in neurogenesis [62], cell proliferation/apoptosis/migration/invasion [63-68] and diverticulum [69] |
| cg02993437 | 1.00 | 7.88×10^-6^ | 2.29×10^-2^ | 2 | 172937981 | *METAP1D* | Body | open sea | implicated in menopause and terminal duct lobular unit involution in normal breast [70] |
| cg01080600 | -0.80 | 1.01×10^-6^ | 4.14×10^-3^ | 2 | 236443773 | *AGAP1* (*GGAP1, CENTG2)* [71-72] | Body | shore | controls cytoskeleton, protein trafficking, mitogen-activated protein kinase signaling pathway [73-75], modulates neuritogenesis [76], synapse transmission [77], involved in schizophrenia [72], autism [78] and cerebral palsy [79] |
| cg04258358 | 0.69 | 3.95×10^-6^ | 1.29×10^-2^ | 4 | 728867 | *PCGF3* | Body | shore | influences transcription at neuronal differentiation [80] and participates in X chromosome inactivation [81] |
| cg22517735 | 0.30 | 1.65×10^-7^ | 8.92×10^-4^ | 4 | 174451954 | *NBLA00301* (HAND2-AS1) [82] | Body | island | acts as a tumor suppressor [82] |
| cg19459207 | 1.00 | 1.30×10^-5^ | 3.46×10^-2^ | 5 | 49737773 | *EMB* (*Embigin*) [83] | TSS1500 | shore | enhances cell-substratum adhesion [84], sprouting of nerve terminal [85], regulates cell quiescence/apoptosis/proliferation/migration/invasion [83,86-88] |
| cg19868864 | 1.00 | 3.77×10^-6^ | 1.24×10^-2^ | 5 | 162901371 | *HMMR* (*RHAMM*, CD168) [89] | Body | open sea | regulates Akt phosphorylation [90], genome stability [91], matrix binding [92], cell differentiation [93], adhesion [94], polarization [95], motility [96-98], epithelial-mesenchymal transition [99], implicated in fibrosis [100-101], mineralization [102], fertility [103], nervous system and mammary gland development [104-106], adipogenesis [107], tumorigenesis [89,108-117], drug sensitivity [118] and chemotherapy-induced neutropenia [119] |
| cg26993940 | 0.70 | 1.39×10^-6^ | 5.32×10^-3^ | 5 | 177892758 | *COL23A1* | Body | open sea | involved in oncogenesis [120], increased in dermal fibroblast of systemic sclerosis [121] and associated with facial shape [122] and body weight change [123] |
| cg07235456 | 0.70 | 2.76×10^-7^ | 1.32×10^-3^ | 6 | 31935499 | *SKIV2L* (*SKI2W)* [124] | Body | open sea | an RNA helicase limiting RIG-I-like receptors activation [125-126], associated with autoimmune disorder [127-129], trichohepatoenteric syndrome [130], severe infantile cholestatic disease [131], age-related macular degeneration [132] and polypoidal choroidal vasculopathy [133] |
| cg24070123 | 1.00 | 8.42×10^-9^ | 7.05×10^-5^ | 6 | 34665156 | *C6orf106* | TSS1500 | shore | suppresses innate antiviral response [134], accelerates cancer proliferation/invasion [135] and associated with body mass index [136] |
| cg27478167 | 0.49 | 1.58×10^-5^ | 4.10×10^-2^ | 7 | 817139 | *HEATR2* | Body | shelf | involved in ciliogenesis and primary ciliary dyskinesia [137-138] |
| cg05096788 | -0.71 | 1.34×10^-7^ | 7.59×10^-4^ | 7 | 128444910 | *CCDC136* (*NAG6)* [139] | Body | open sea | involved in reading performance [139] and fertilization [140] |
| cg11768833 | -1.00 | 5.91×10^-10^ | 6.70×10^-6^ | 9 | 13140958 | *MPDZ* (*MUPP1*) [141] | Body | open sea | promotes Gi coupling to receptor [142], controls ion transport [143], cell exocytosis [144], cell polarity complex expression [145], cell transformation [146], cell junction [147], involved in neuron signaling [141, 148], central nervous system hyperexcitability [149], neurodevelopment [150-151], intellectual disability [152], neuropsychiatric illness [Supplementary Table 2 of 153], opioid tolerance and opioid-induced hyperalgesia [154], alcoholism and alcohol withdrawal [155-156], pain sensation [157], congenital hereditary endothelial dystrophy/ endothelial corneal dystrophy [158], keratoconus [159], angiogenesis [160], malignancy [161] |
| cg09762316 | -1.00 | 1.04×10^-5^ | 2.93×10^-2^ | 9 | 72658354 | *MAMDC2* | TSS200 | shore | increased in atherosclerosis [162], involved in mental retardation and intellectual disability [163-164] |
| cg00748492 | -1.00 | 6.91×10^-9^ | 5.92×10^-5^ | 10 | 22216446 | *DNAJC1* (*MTJ1, HTJ1, ERj1*) [165-166] | Body | open sea | participates in protein folding and trafficking, apoptosis, immune regulation [165], metabolism [167], endothelial cell function [168] and associated with asthma, body mass index and subcutaneous fat [169-171] |
| cg25692425 | -0.56 | 1.51×10^-5^ | 3.94×10^-2^ | 10 | 61721716 | *C10orf40* | TSS1500 | open sea | unknown |
| cg08234376 | 0.71 | 1.42×10^-6^ | 5.38×10^-3^ | 10 | 70100076 | *HNRNPH3* | Body | open sea | associated with lymphoma survival [172] |
| cg19411943 | 0.69 | 2.21×10^-7^ | 1.09×10^-3^ | 10 | 75306538 | *USP54* | Body | open sea | promotes cancer metastasis [173] |
| cg19469189 | -0.56 | 1.07×10^-7^ | 6.34×10^-4^ | 11 | 128781035 | *KCNJ5* (*GIRK4)* [174] | 5'UTR | open sea | stimulates aldosterone production [175] and associated with primary aldosteronism/aldosterone-producing adenomas [176], electrophysiological properties of heart [177] and various arrhythmia [178-181], metabolic syndrome [182], obesity [183] and fertility [184] |
| cg18961101 | -0.62 | 9.33×10^-6^ | 2.68×10^-2^ | 12 | 4923116 | *KCNA6* (*Kv1.6, IK*(*DR*)) [185-186] | 3'UTR | shelf | modulates cell proliferation and migration [187-188] and food intake [189] |
| cg19532939 | -0.91 | 1.83×10^-5^ | 4.64×10^-2^ | 12 | 14996525 | *ART4* (*Rod1*) [190] | TSS200 | open sea | regulates hexose transporter trafficking [190] |
| cg02325300 | 0.72 | 3.64×10^-13^ | 9.95×10^-9^ | 12 | 16758836 | *LMO3* | 5'UTR | open sea | involved in neuron development [191], hydrocephalus [192], anxiety, depression, long-term potentiation [193-194], behavioral responses to ethanol [195], adipocyte differentiation and adipogenesis [196-197] and various aspects of oncogenesis [198-202] |
| cg25983594 | -0.68 | 6.64×10^-6^ | 1.99×10^-2^ | 13 | 113650922 | *MCF2L* (*Dbs, Ost*) [203-204] | Body | shore | regulates receptor endocytosis [205], synapse formation [206], NF-kB activation [207], cell senescence [204], proliferation [208-209] and migration [210-211], implicated in cancer [212], atherosclerosis [213] and osteoarthritis [203] |
| cg07445547 | 0.59 | 1.86×10^-6^ | 6.71×10^-3^ | 14 | 101193006 | *DLK1* (*Pref1*) [214] | TSS200 | island | interferes with NOTCH1 signaling [215], affects adipocyte browning and adipogenesis [216-217], obesity, metabolic adaptations, nutrient metabolism [218-220], hormone secretion [221-222], hematopoiesis [223-224], cell proliferation/differentiation/apoptosis [225-226], angiogenesis, motility, invasion and epithelial-mesenchymal transition [227] |
| cg22241833 | -0.47 | 1.15×10^-6^ | 4.63×10^-3^ | 15 | 25415399 | *SNORD115-1* | TSS1500 | open sea | unknown |
| cg11169286 | 0.17 | 6.66×10^-6^ | 1.99×10^-2^ | 16 | 686434 | *C16orf13* | TSS200 | island | unknown |
| cg05438708 | -0.50 | 5.84×10^-6^ | 1.81×10^-2^ | 16 | 81520178 | *CMIP* | Body | open sea | affects cell signaling and cytoskeleton [228-230] and associated with podocyte damage, glomerular diseases and nephrotic syndrome [231-234], oncogenesis [235-236], cholesterol efflux capacity and lipolysis [237-238], adiponectin level, physical traits and diabetes [239-240], language, reading, hearing function [241-243] and autism spectrum disorder [244] |
| cg11367590 | 0.98 | 2.92×10^-7^ | 1.39×10^-3^ | 17 | 17226082 | *NT5M* | Body | open sea | unknown |
| cg05464572 | 0.79 | 4.48×10^-7^ | 2.01×10^-3^ | 17 | 72694068 | *RAB37* | Body | open sea | participates in exocytosis and cellular secretion [245-248], autophagy [249], cell proliferation, migration, invasion, angiogenesis, metastasis, epithelial-mesenchymal transition [250-255] |
| cg14165663 | 1.00 | 1.80×10^-5^ | 4.58×10^-2^ | 17 | 74070269 | *GALR2* | TSS1500 | shore | activates MAPK [256] and decreases fibrosis [257], involved in neurogenesis [258-260], long-term potentiation, anxiety, analgesia, allodynia, morphine conditioned place preference, alcohol consumption and multiple sclerosis [261-268] and participates in insulin resistance/glucose metabolism/lipolysis [269-271], bowel movement/bile acid synthesis [272-273], cellular apoptosis/proliferation/invasion/angiogenesis [274-278] |
| cg26351916 | 0.99 | 8.47×10^-8^ | 5.27×10^-4^ | 17 | 78084228 | *GAA*(*acid-α-glucosidase*) [279] | Body | shore | associated with coronary artery disease and glycogen storage disease [280-281] |
| cg04810466 | -1.00 | 9.68×10^-7^ | 4.01×10^-3^ | 19 | 30914969 | *ZNF536* | 5'UTR | open sea | regulates neuron differentiation and brain development [282-283], associated with bipolar disorder with comorbid binge eating history [284], antidepressant response [285] and liposarcoma [286] |
| cg05532013 | 0.60 | 1.86×10^-7^ | 9.63×10^-4^ | 22 | 23650781 | *BCR* | Body | open sea | activates extracellular signal-regulated kinase and transactivation domain of RelA/p65 [287-288], modulates respiratory burst [289], cellular differentiation, proliferation and angiogenesis [290-293], synapse formation and neural development [294-298], involved in memory, bipolar disorder and subsequent treatment response [299-301] |
| Chr: chromosome; CpG: cytosine-phosphate-guanine dinucleotide; FDR: false discovery rate. | | | | | | | | | |
| ^a^Methylation levels of gout-methylation levels of non-gout in monocytes after adjusting for age, sex, alcohol drinking, smoking status, smoking history (total pack-years) and cell fractions with CellDMC. | | | | | | | | | |
| ^b^Positions of the CpG sites in hg19. | | | | | | | | | |
| ^c^Gene names and their alias. | | | | | | | | | |
| **Reference** | | | | | | | | | |
| 1. Chen X, Hu L, Yang H, Ma H, Ye K, Zhao C, Zhao Z, Dai H, Wang H, Fang Z. DHHC protein family targets different subsets of glioma stem cells in specific niches. J Exp Clin Cancer Res. 2019; 38:25. | | | | | | | | | |
| 2. Zhao Y, He A, Zhu F, Ding M, Hao J, Fan Q, Li P, Liu L, Du Y, Liang X, Guo X, Zhang F, Ma X. Integrating genome-wide association study and expression quantitative trait locus study identifies multiple genes and gene sets associated with schizophrenia. Prog Neuropsychopharmacol Biol Psychiatry. 2018; 81:50-4. | | | | | | | | | |
| 3. Pendharkar N, Dhali S, Abhang S. A Novel Strategy to Investigate Tissue-Secreted Tumor Microenvironmental Proteins in Serum toward Development of Breast Cancer Early Diagnosis Biomarker Signature. Proteomics Clin Appl. 2019; 13:e1700119. | | | | | | | | | |
| 4. Vezain M, Lecuyer M, Rubio M, Dupé V, Ratié L, David V, Pasquier L, Odent S, Coutant S, Tournier I, Trestard L, Adle-Biassette H, Vivien D, et al. A de novo variant in ADGRL2 suggests a novel mechanism underlying the previously undescribed association of extreme microcephaly with severely reduced sulcation and rhombencephalosynapsis. Acta Neuropathol Commun. 2018; 6:109. | | | | | | | | | |
| 5. Yokote N, Suzuki-Kosaka MY, Michiue T, Hara T, Tanegashima K. Latrophilin2 is involved in neural crest cell migration and placode patterning in Xenopus laevis. Int J Dev Biol. 2019; 63:29-35. | | | | | | | | | |
| 6. Anderson GR, Maxeiner S, Sando R, Tsetsenis T, Malenka RC, Südhof TC. Postsynaptic adhesion GPCR latrophilin-2 mediates target recognition in entorhinal-hippocampal synapse assembly. J Cell Biol. 2017; 216:3831-46. | | | | | | | | | |
| 7. Sun J, Kranzler HR, Gelernter J, Bi J. A genome-wide association study of cocaine use disorder accounting for phenotypic heterogeneity and gene–environment interaction. J Psychiatry Neurosci. 2020; 45:34-44. | | | | | | | | | |
| 8. Obeidat M, Nie Y, Fishbane N, Li X, Bossé Y, Joubert P, Nickle DC, Hao K, Postma DS, Timens W, Sze MA, Shannon CP, Hollander Z, et al. Integrative Genomics of Emphysema-Associated Genes Reveals Potential Disease Biomarkers. Am J Respir Cell Mol Biol. 2017; 57:411-8. | | | | | | | | | |
| 9. Teh MT, Tilakaratne WM, Chaplin T, Young BD, Ariyawardana A, Pitiyage G, Lalli A, Stewart JE, Hagi-Pavli E, Cruchley A, Waseem A, Fortune F. Fingerprinting genomic instability in oral submucous fibrosis. J Oral Pathol Med. 2008; 37:430-6. | | | | | | | | | |
| 10. Mohamad Shah NS, Salahshourifar I, Sulong S, Wan Sulaiman WA, Halim AS. Discovery of candidate genes for nonsyndromic cleft lip palate through genome-wide linkage analysis of large extended families in the Malay population. BMC Genet. 2016; 17:39. | | | | | | | | | |
| 11. Jeon MS, Song SH, Yun J, Kang JY, Kim HP, Han SW, Kim TY. Aberrant Epigenetic Modifications of LPHN2 Function as a Potential Cisplatin-Specific Biomarker for Human Gastrointestinal Cancer. Cancer Res Treat. 2016; 48:676-86. | | | | | | | | | |
| 12. Lee KT, Byun MJ, Kang KS, Park EW, Lee SH, Cho S, Kim H, Kim KW, Lee T, Park JE, Park W, Shin D, Park HS, et al. Neuronal genes for subcutaneous fat thickness in human and pig are identified by local genomic sequencing and combined SNP association study. PLoS One. 2011; 6:e16356. | | | | | | | | | |
| 13. Moreno-Salinas AL, Avila-Zozaya M, Ugalde-Silva P, Hernández-Guzmán DA, Missirlis F, Boucard AA. Latrophilins: A Neuro-Centric View of an Evolutionary Conserved Adhesion G Protein-Coupled Receptor Subfamily. Front Neurosci. 2019; 13:700. | | | | | | | | | |
| 14. Ndila CM, Uyoga S, Macharia AW, Nyutu G, Peshu N, Ojal J, Shebe M, Awuondo KO, Mturi N, Tsofa B, Sepúlveda N, Clark TG, Band G, et al. Human candidate gene polymorphisms and risk of severe malaria in children in Kilifi, Kenya: a case-control association study. Lancet Haematol. 2018; 5:e333-e45. | | | | | | | | | |
| 15. Cline JL, Beckie TM. The relationships between FAM5C SNP (rs10920501) variability and metabolic syndrome and inflammation in women with coronary heart disease. Biol Res Nurs. 2013; 15:160-6. | | | | | | | | | |
| 16. Shorts-Cary L, Xu M, Ertel J, Kleinschmidt-Demasters BK, Lillehei K, Matsuoka I, Nielsen-Preiss S, Wierman ME. Bone morphogenetic protein and retinoic acid-inducible neural specific protein-3 is expressed in gonadotrope cell pituitary adenomas and induces proliferation, migration, and invasion. Endocrinology. 2007; 148:967-75. | | | | | | | | | |
| 17. Tanaka K, Matsumoto E, Higashimaki Y, Sugimoto T, Seino S, Kaji H. FAM5C is a soluble osteoblast differentiation factor linking muscle to bone. Biochem Biophys Res Commun. 2012; 418:134-9. | | | | | | | | | |
| 18. Karoly HC, Stevens CJ, Magnan RE, Harlaar N, Hutchison KE, Bryan AD. Genetic Influences on Physiological and Subjective Responses to an Aerobic Exercise Session among Sedentary Adults. J Cancer Epidemiol. 2012; 2012:540563. | | | | | | | | | |
| 19. Larson MG, Atwood LD, Benjamin EJ, Cupples LA, D'Agostino RB, Fox CS, Govindaraju DR, Guo CY, Heard-Costa NL, Hwang SJ, Murabito JM, et al. Framingham Heart Study 100K project: genome-wide associations for cardiovascular disease outcomes. BMC Med Genet. 2007; 8 Suppl 1:S5. | | | | | | | | | |
| 20. Vasan RS, Larson MG, Aragam J, Wang TJ, Mitchell GF, Kathiresan S, Newton-Cheh C, Vita JA, Keyes MJ, O'Donnell CJ, Levy D, Benjamin EJ. Genome-wide association of echocardiographic dimensions, brachial artery endothelial function and treadmill exercise responses in the Framingham Heart Study. BMC Med Genet. 2007; 8 Suppl 1:S2. | | | | | | | | | |
| 21. Zheng P, Eleanor RM, Guo T, Dejun S, Vishnu R. Prognostic Genomic Biomarkers for Acute Myeloid Leukemia (AML) Based on French-American-British (FAB) Subtypes. Blood. 2016; 128:5259. | | | | | | | | | |
| 22. Carvalho FM, Tinoco EM, Deeley K, Duarte PM, Faveri M, Marques MR, Mendonça AC, Wang X, Cuenco K, Menezes R, Garlet GP, Vieira AR.FAM5C contributes to aggressive periodontitis. PLoS One. 2010; 5:e10053. | | | | | | | | | |
| 23. Guimarães JM, Guimarães IC, Duarte ME, Vieira T, Vianna VF, Fernandes MB, Vieira AR, Casado PL. Polymorphisms in BMP4 and FGFR1 genes are associated with fracture non-union. J Orthop Res. 2013; 31:1971-9. | | | | | | | | | |
| 24. Zhang M, Wu J, Zhong W, Zhao Z, Liu Z. Long non-coding RNA BRE-AS1 represses non-small cell lung cancer cell growth and survival via up-regulating NR4A3. Arch Biochem Biophys. 2018; 660:53-63. | | | | | | | | | |
| 25. Chen Z, Zhen M, Zhou J. LncRNA BRE-AS1 interacts with miR-145-5p to regulate cancer cell proliferation and apoptosis in prostate carcinoma and has early diagnostic values. Biosci Rep. 2019; 39:BSR20182097. | | | | | | | | | |
| 26. Raciti M, Salma J, Spulber S, Gaudenzi G, Khalajzeyqami Z, Conti M, Anderlid BM, Falk A, Hermanson O, Ceccatelli S. NRXN1 Deletion and Exposure to Methylmercury Increase Astrocyte Differentiation by Different Notch-Dependent Transcriptional Mechanisms. Front Genet. 2019; 10:593. | | | | | | | | | |
| 27. Lam M, Moslem M, Bryois J, Pronk RJ, Uhlin E, Ellström ID, Laan L, Olive J, Morse R, Rönnholm H, Louhivuori L, Korol SV, Dahl N, et al. Single cell analysis of autism patient with bi-allelic NRXN1-alpha deletion reveals skewed fate choice in neural progenitors and impaired neuronal functionality. Exp Cell Res. 2019; 383:111469. | | | | | | | | | |
| 28. Pak C, Danko T, Zhang Y, Aoto J, Anderson G, Maxeiner S, Yi F, Wernig M, Südhof TC. Human Neuropsychiatric Disease Modeling using Conditional Deletion Reveals Synaptic Transmission Defects Caused by Heterozygous Mutations in NRXN1. Cell Stem Cell. 2015; 17:316-28. | | | | | | | | | |
| 29. Brockhaus J, Schreitmüller M, Repetto D, Klatt O, Reissner C, Elmslie K, Heine M, Missler M. α-Neurexins Together with α2δ-1 Auxiliary Subunits Regulate Ca2+ Influx through Cav2.1 Channels. J Neurosci. 2018; 38:8277-94. | | | | | | | | | |
| 30. Traunmüller L, Gomez AM, Nguyen TM, Scheiffele P. Control of neuronal synapse specification by a highly dedicated alternative splicing program. Science. 2016; 352:982-6. | | | | | | | | | |
| 31. Zeng L, Zhang P, Shi L, Yamamoto V, Lu W, Wang K. Functional impacts of NRXN1 knockdown on neurodevelopment in stem cell models. PLoS One. 2013; 8:e59685. | | | | | | | | | |
| 32. Alliey-Rodriguez N, Grey TA, Shafee R, Asif H, Lutz O, Bolo NR, Padmanabhan J, Tandon N, Klinger M, Reis K, Spring J, Coppes L, Zeng V, et al. NRXN1 is associated with enlargement of the temporal horns of the lateral ventricles in psychosis. Transl Psychiatry. 2019; 9:230. | | | | | | | | | |
| 33. Schaaf CP, Boone PM, Sampath S, Williams C, Bader PI, Mueller JM, Shchelochkov OA, Brown CW, Crawford HP, Phalen JA, Tartaglia NR, Evans P, Campbell WM, et al. Phenotypic spectrum and genotype-phenotype correlations of NRXN1 exon deletions. Eur J Hum Genet. 2012; 20:1240-7. | | | | | | | | | |
| 34. Gudmundsson OO, Walters GB, Ingason A, Johansson S, Zayats T, Athanasiu L, Sonderby IE, Gustafsson O, Nawaz MS, Jonsson GF, Jonsson L, Knappskog PM, Ingvarsdottir E, et al. Attention-deficit hyperactivity disorder shares copy number variant risk with schizophrenia and autism spectrum disorder. Transl Psychiatry. 2019; 9:258. | | | | | | | | | |
| 35. Lowther C, Speevak M, Armour CM, Goh ES, Graham GE, Li C, Zeesman S, Nowaczyk MJ, Schultz LA, Morra A, Nicolson R, Bikangaga P, Samdup D, et al. Molecular characterization of NRXN1 deletions from 19,263 clinical microarray cases identifies exons important for neurodevelopmental disease expression. Genet Med. 2017; 19:53-61. | | | | | | | | | |
| 36. Castronovo P, Baccarin M, Ricciardello A, Picinelli C, Tomaiuolo P, Cucinotta F, Frittoli M, Lintas C, Sacco R, Persico AM. Phenotypic spectrum of NRXN1 mono- and bi-allelic deficiency: A systematic review. Clin Genet. 2020; 97:125-37. | | | | | | | | | |
| 37. Rochtus AM, Trowbridge S, Goldstein RD, Sheidley BR, Prabhu SP, Haynes R, Kinney HC, Poduri AH. Mutations in NRXN1 and NRXN2 in a patient with early-onset epileptic encephalopathy and respiratory depression. Cold Spring Harb Mol Case Stud. 2019; 5:a003442. | | | | | | | | | |
| 38. Curran S, Ahn JW, Grayton H, Collier DA, Ogilvie CM. NRXN1 deletions identified by array comparative genome hybridisation in a clinical case series - further understanding of the relevance of NRXN1 to neurodevelopmental disorders. J Mol Psychiatry. 2013; 1:4. | | | | | | | | | |
| 39. Al Shehhi M, Forman EB, Fitzgerald JE, McInerney V, Krawczyk J, Shen S, Betts DR, Ardle LM, Gorman KM, King MD, Green A, Gallagher L, Lynch SA. NRXN1 deletion syndrome; phenotypic and penetrance data from 34 families. Eur J Med Genet. 2019; 62:204-9. | | | | | | | | | |
| 40. Dabell MP, Rosenfeld JA, Bader P, Escobar LF, El-Khechen D, Vallee SE, Dinulos MB, Curry C, Fisher J, Tervo R, Hannibal MC, Siefkas K, Wyatt PR, et al. Investigation of NRXN1 deletions: clinical and molecular characterization. Am J Med Genet A. 2013; 161A:717-31. | | | | | | | | | |
| 41. Brignell A, St John M, Boys A, Bruce A, Dinale C, Pigdon L, Hildebrand MS, Amor DJ, Morgan AT. Characterization of speech and language phenotype in children with NRXN1 deletions. Am J Med Genet B Neuropsychiatr Genet. 2018; 177:700-8. | | | | | | | | | |
| 42. Yuan H, Wang Q, Liu Y, Yang W, He Y, Gusella JF, Song J, Shen Y. A rare exonic NRXN3 deletion segregating with neurodevelopmental and neuropsychiatric conditions in a three-generation Chinese family. Am J Med Genet B Neuropsychiatr Genet. 2018; 177:589-95. | | | | | | | | | |
| 43. Li Y, Liu H, Dong Y. Significance of neurexin and neuroligin polymorphisms in regulating risk of Hirschsprung's disease. J Investig Med. 2018; 66:1-8. | | | | | | | | | |
| 44. Huang AY, Yu D, Davis LK, Sul JH, Tsetsos F, Ramensky V, Zelaya I, Ramos EM, Osiecki L, Chen JA, McGrath LM, Illmann C, Sandor P, et al. Rare Copy Number Variants in NRXN1 and CNTN6 Increase Risk for Tourette Syndrome. Neuron. 2017; 94:1101-11.e7. | | | | | | | | | |
| 45. Noh HJ, Tang R, Flannick J, O'Dushlaine C, Swofford R, Howrigan D, Genereux DP, Johnson J, van Grootheest G, Grünblatt E, Andersson E, Djurfeldt DR, Patel PD, et al. Integrating evolutionary and regulatory information with a multispecies approach implicates genes and pathways in obsessive-compulsive disorder. Nat Commun. 2017; 8:774. | | | | | | | | | |
| 46. Pérez-Palma E, Helbig I, Klein KM, Anttila V, Horn H, Reinthaler EM, Gormley P, Ganna A, Byrnes A, Pernhorst K, Toliat MR, Saarentaus E, Howrigan DP, et al. Heterogeneous contribution of microdeletions in the development of common generalised and focal epilepsies. J Med Genet. 2017; 54:598-606. | | | | | | | | | |
| 47. Twining RC, Vantrease JE, Love S, Padival M, Rosenkranz JA. An intra-amygdala circuit specifically regulates social fear learning. Nat Neurosci. 2017; 20:459-69. | | | | | | | | | |
| 48. Rietveld CA, Esko T, Davies G, Pers TH, Turley P, Benyamin B, Chabris CF, Emilsson V, Johnson AD, Lee JJ, de Leeuw C, Marioni RE, Medland SE, et al. Common genetic variants associated with cognitive performance identified using the proxy-phenotype method. Proc Natl Acad Sci U S A. 2014; 111:13790-4. | | | | | | | | | |
| 49. Pérez-Rubio G, Pérez-Rodríguez ME, Fernández-López JC, Ramírez-Venegas A, García-Colunga J, Ávila-Moreno F, Camarena A, Sansores RH, Falfán-Valencia R.SNPs in NRXN1 and CHRNA5 are associated to smoking and regulation of GABAergic and glutamatergic pathways. Pharmacogenomics. 2016; 17:1145-58. | | | | | | | | | |
| 50. Lett TA, Tiwari AK, Meltzer HY, Lieberman JA, Potkin SG, Voineskos AN, Kennedy JL, Müller DJ. The putative functional rs1045881 marker of neurexin-1 in schizophrenia and clozapine response. Schizophr Res. 2011; 132:121-4. | | | | | | | | | |
| 51. Tansey KE, Rucker JJ, Kavanagh DH, Guipponi M, Perroud N, Bondolfi G, Domenici E, Evans DM, Hauser J, Henigsberg N, Jerman B, Maier W, Mors O, et al. Copy number variants and therapeutic response to antidepressant medication in major depressive disorder. Pharmacogenomics J. 2014; 14:395-9. | | | | | | | | | |
| 52. Li Q, Wojciechowski R, Simpson CL, Hysi PG, Verhoeven VJ, Ikram MK, Höhn R, Vitart V, Hewitt AW, Oexle K, Mäkelä KM, MacGregor S, Pirastu M, et al. Genome-wide association study for refractive astigmatism reveals genetic co-determination with spherical equivalent refractive error: the CREAM consortium. Hum Genet. 2015; 134:131-46. | | | | | | | | | |
| 53. Hesson LB, Ng B, Zarzour P, Srivastava S, Kwok CT, Packham D, Nunez AC, Beck D, Ryan R, Dower A, Ford CE, Pimanda JE, Sloane MA, et al. Integrated Genetic, Epigenetic, and Transcriptional Profiling Identifies Molecular Pathways in the Development of Laterally Spreading Tumors. Mol Cancer Res. 2016; 14:1217-28. | | | | | | | | | |
| 54. Kim JH, Park K, Yim SH, Choi JE, Sung JS, Park JY, Choi YY, Jeon HS, Yoon HK, Kim YH, Yoo BS, Kim YT, Hu HJ, et al. Genome-wide association study of lung cancer in Korean non-smoking women. J Korean Med Sci. 2013; 28:840-7. | | | | | | | | | |
| 55. Owen D, Bracher-Smith M, Kendall KM, Rees E, Einon M, Escott-Price V, Owen MJ, O'Donovan MC, Kirov G. Effects of pathogenic CNVs on physical traits in participants of the UK Biobank. BMC Genomics. 2018; 19:867. | | | | | | | | | |
| 56. Degenhardt F, Niklowitz P, Szymczak S, Jacobs G, Lieb W, Menke T, Laudes M, Esko T, Weidinger S, Franke A, Döring F, Onur S. Genome-wide association study of serum coenzyme Q10 levels identifies susceptibility loci linked to neuronal diseases. Hum Mol Genet. 2016; 25:2881-91. | | | | | | | | | |
| 57. Zamboni V, Armentano M, Sarò G, Ciraolo E, Ghigo A, Germena G, Umbach A, Valnegri P, Passafaro M, Carabelli V, Gavello D, Bianchi V, D'Adamo P, et al. Disruption of ArhGAP15 results in hyperactive Rac1, affects the architecture and function of hippocampal inhibitory neurons and causes cognitive deficits. Sci Rep. 2016; 6:34877. | | | | | | | | | |
| 58. Seoh ML, Ng CH, Yong J, Lim L, Leung T. ArhGAP15, a novel human RacGAP protein with GTPase binding property. FEBS Lett. 2003; 539:131-7. | | | | | | | | | |
| 59. Radu M, Rawat SJ, Beeser A, Iliuk A, Tao WA, Chernoff J. ArhGAP15, a Rac-specific GTPase-activating protein, plays a dual role in inhibiting small GTPase signaling. J Biol Chem. 2013; 288:21117-25. | | | | | | | | | |
| 60. Graziano BR, Gong D, Anderson KE, Pipathsouk A, Goldberg AR, Weiner OD. A module for Rac temporal signal integration revealed with optogenetics. J Cell Biol. 2017; 216:2515-31. | | | | | | | | | |
| 61. Campa CC, Germena G, Ciraolo E, Copperi F, Sapienza A, Franco I, Ghigo A, Camporeale A, Di Savino A, Martini M, Perino A, Megens RT, Kurz AR, et al. Rac signal adaptation controls neutrophil mobilization from the bone marrow. Sci Signal. 2016; 9:ra124. | | | | | | | | | |
| 62. Zamboni V, Armentano M, Berto G, Ciraolo E, Ghigo A, Garzotto D, Umbach A, DiCunto F, Parmigiani E, Boido M, Vercelli A, El-Assawy N, Mauro A, et al. Hyperactivity of Rac1-GTPase pathway impairs neuritogenesis of cortical neurons by altering actin dynamics. Sci Rep. 2018; 8:7254. | | | | | | | | | |
| 63. Costa C, Germena G, Martin-Conte EL, Molineris I, Bosco E, Marengo S, Azzolino O, Altruda F, Ranieri VM, Hirsch E. The RacGAP ArhGAP15 is a master negative regulator of neutrophil functions. Blood. 2011; 118:1099-108. | | | | | | | | | |
| 64. Pan S, Deng Y, Fu J, Zhang Y, Zhang Z, Ru X, Qin X. Decreased expression of ARHGAP15 promotes the development of colorectal cancer through PTEN/AKT/FOXO1 axis. Cell Death Dis. 2018; 9:673. | | | | | | | | | |
| 65. Liu ZD, Mou ZX, Che XH, Wang K, Li HX, Chen XY, Guo XM. ARHGAP15 regulates lung cancer cell proliferation and metastasis via the STAT3 pathway. Eur Rev Med Pharmacol Sci. 2019; 23:5840-50. | | | | | | | | | |
| 66. Takagi K, Miki Y, Onodera Y, Ishida T, Watanabe M, Sasano H, Suzuki T. ARHGAP15 in Human Breast Carcinoma: A Potent Tumor Suppressor Regulated by Androgens. Int J Mol Sci. 2018; 19:804. | | | | | | | | | |
| 67. Sun Z, Zhang B, Wang C, Fu T, Li L, Wu Q, Cai Y, Wang J. Forkhead box P3 regulates ARHGAP15 expression and affects migration of glioma cells through the Rac1 signaling pathway. Cancer Sci. 2017; 108:61-72. | | | | | | | | | |
| 68. Ullah I, Lee HY, Kim MJ, Shah SA, Badshah H, Kim TH, Chung HJ, Yang BC, Kim MO. Rho GTPase activating protein 15 (arhGAP15) siRNA effect apoptosis-induced by ethanol in bovine fibroblast cells. Pak J Pharm Sci. 2013; 26:605-10. | | | | | | | | | |
| 69. Schafmayer C, Harrison JW, Buch S, Lange C, Reichert MC, Hofer P, Cossais F, Kupcinskas J, von Schönfels W, Schniewind B, Kruis W, Tepel J, Zobel M, et al. Genome-wide association analysis of diverticular disease points towards neuromuscular, connective tissue and epithelial pathomechanisms. Gut. 2019; 68:854-65. | | | | | | | | | |
| 70. Oh H, Bodelon C, Palakal M, Chatterjee N, Sherman ME, Linville L, Geller BM, Vacek PM, Weaver DL, Chicoine RE, Papathomas D, Patel DA, Xiang J, et al. Ages at menarche- and menopause-related genetic variants in relation to terminal duct lobular unit involution in normal breast tissue. Breast Cancer Res Treat. 2016; 158:341-50. | | | | | | | | | |
| 71. Ahn JY, Ye K. PIKE GTPase signaling and function. Int J Biol Sci. 2005; 1:44-50. | | | | | | | | | |
| 72. Shi J, Levinson DF, Duan J, Sanders AR, Zheng Y, Pe'er I, Dudbridge F, Holmans PA, Whittemore AS, Mowry BJ, Olincy A, Amin F, Cloninger CR, et al. Common variants on chromosome 6p22.1 are associated with schizophrenia. Nature. 2009; 460:753-7. | | | | | | | | | |
| 73. Luo R, Chen PW, Wagenbach M, Jian X, Jenkins L, Wordeman L, Randazzo PA. Direct Functional Interaction of the Kinesin-13 Family Member Kinesin-like Protein 2A (Kif2A) and Arf GAP with GTP-binding Protein-like, Ankyrin Repeats and PH Domains1 (AGAP1). J Biol Chem. 2016; 291:21350-62. | | | | | | | | | |
| 74. Nie Z, Boehm M, Boja ES, Vass WC, Bonifacino JS, Fales HM, Randazzo PA. Specific regulation of the adaptor protein complex AP-3 by the Arf GAP AGAP1. Dev Cell. 2003; 5:513-21. | | | | | | | | | |
| 75. Xia C, Ma W, Stafford LJ, Liu C, Gong L, Martin JF, Liu M. GGAPs, a new family of bifunctional GTP-binding and GTPase-activating proteins. Mol Cell Biol. 2003; 23:2476-88. | | | | | | | | | |
| 76. Arnold M, Cross R, Singleton KS, Zlatic S, Chapleau C, Mullin AP, Rolle I, Moore CC, Theibert A, Pozzo-Miller L, Faundez V, Larimore J. The Endosome Localized Arf-GAP AGAP1 Modulates Dendritic Spine Morphology Downstream of the Neurodevelopmental Disorder Factor Dysbindin. Front Cell Neurosci. 2016; 10:218. | | | | | | | | | |
| 77. Bendor J, Lizardi-Ortiz JE, Westphalen RI, Brandstetter M, Hemmings HC, Sulzer D, Flajolet M, Greengard P. AGAP1/AP-3-dependent endocytic recycling of M5 muscarinic receptors promotes dopamine release. EMBO J. 2010; 29:2813-26. | | | | | | | | | |
| 78. Wassink TH, Piven J, Vieland VJ, Jenkins L, Frantz R, Bartlett CW, Goedken R, Childress D, Spence MA, Smith M, Sheffield VC. Evaluation of the chromosome 2q37.3 gene CENTG2 as an autism susceptibility gene. Am J Med Genet B Neuropsychiatr Genet. 2005; 136B:36-44. | | | | | | | | | |
| 79. McMichael G, Bainbridge MN, Haan E, Corbett M, Gardner A, Thompson S, van Bon BW, van Eyk CL, Broadbent J, Reynolds C, O'Callaghan ME, Nguyen LS, Adelson DL, et al. Whole-exome sequencing points to considerable genetic heterogeneity of cerebral palsy. Mol Psychiatry. 2015; 20:176-82. | | | | | | | | | |
| 80. Monderer-Rothkoff G, Tal N, Risman M, Shani O, Nissim-Rafinia M, Malki-Feldman L, Medvedeva V, Groszer M, Meshorer E, Shifman S. AUTS2 isoforms control neuronal differentiation. Mol Psychiatry. 2019. [Epub ahead of print]. https://doi.org/10.1038/s41380-019-0409-1. | | | | | | | | | |
| 81. Almeida M, Pintacuda G, Masui O, Koseki Y, Gdula M, Cerase A, Brown D, Mould A, Innocent C, Nakayama M, Schermelleh L, Nesterova TB, Koseki H, et al. PCGF3/5-PRC1 initiates Polycomb recruitment in X chromosome inactivation. Science. 2017; 356:1081-4. | | | | | | | | | |
| 82. Khajehdehi M, Khalaj-Kondori M, Ghasemi T, Jahanghiri B, Damaghi M. Long Noncoding RNAs in Gastrointestinal Cancer: Tumor Suppression Versus Tumor Promotion. Dig Dis Sci. 2020;10.1007/s10620-020-06200-x. doi:10.1007/s10620-020-06200-x. | | | | | | | | | |
| 83. Ruma IMW, Kinoshita R, Tomonobu N, Inoue Y, Kondo E, Yamauchi A, Sato H, Sumardika IW, Chen Y, Yamamoto KI, Murata H, Toyooka S, Nishibori M, et al. Embigin Promotes Prostate Cancer Progression by S100A4-Dependent and-Independent Mechanisms. Cancers (Basel). 2018; 10:239. | | | | | | | | | |
| 84. Huang RP, Ozawa M, Kadomatsu K, Muramatsu T. Embigin, a member of the immunoglobulin superfamily expressed in embryonic cells, enhances cell-substratum adhesion. Dev Biol. 1993; 155:307-14. | | | | | | | | | |
| 85. Lain E, Carnejac S, Escher P, Wilson MC, Lømo T, Gajendran N, Brenner HR. A novel role for embigin to promote sprouting of motor nerve terminals at the neuromuscular junction. J Biol Chem. 2009; 284:8930-9. | | | | | | | | | |
| 86. Silberstein L, Goncalves KA, Kharchenko PV, Turcotte R, Kfoury Y, Mercier F, Baryawno N, Severe N, Bachand J, Spencer JA, Papazian A, Lee D, Chitteti BR, et al. Proximity-Based Differential Single-Cell Analysis of the Niche to Identify Stem/Progenitor Cell Regulators. Cell Stem Cell. 2016; 19:530-43. | | | | | | | | | |
| 87. Chao F, Zhang J, Zhang Y, Liu H, Yang C, Wang J, Guo Y, Wen X, Zhang K, Huang B, Liu D, Li Y. Embigin, regulated by HOXC8, plays a suppressive role in breast tumorigenesis. Oncotarget. 2015; 6:23496-509. | | | | | | | | | |
| 88. Jung DE, Kim JM, Kim C, Song SY. Embigin is overexpressed in pancreatic ductal adenocarcinoma and regulates cell motility through epithelial to mesenchymal transition via the TGF-β pathway. Mol Carcinog. 2016; 55:633-45. | | | | | | | | | |
| 89. Gust KM, Hofer MD, Perner SR, Kim R, Chinnaiyan AM, Varambally S, Moller P, Rinnab L, Rubin MA, Greiner J, Schmitt M, Kuefer R, Ringhoffer M. RHAMM (CD168) is overexpressed at the protein level and may constitute an immunogenic antigen in advanced prostate cancer disease. Neoplasia. 2009; 11:956-63. | | | | | | | | | |
| 90. Onodera Y, Teramura T, Takehara T, Fukuda K. Hyaluronic acid regulates a key redox control factor Nrf2 via phosphorylation of Akt in bovine articular chondrocytes. FEBS Open Bio. 2015; 5:476-84. | | | | | | | | | |
| 91. Chen H, Connell M, Mei L, Reid GSD, Maxwell CA. The nonmotor adaptor HMMR dampens Eg5-mediated forces to preserve the kinetics and integrity of chromosome segregation. Mol Biol Cell. 2018; 29:786-96. | | | | | | | | | |
| 92. Veiseh M, Leith SJ, Tolg C, Elhayek SS, Bahrami SB, Collis L, Hamilton S, McCarthy JB, Bissell MJ, Turley E. Uncovering the dual role of RHAMM as an HA receptor and a regulator of CD44 expression in RHAMM-expressing mesenchymal progenitor cells. Front Cell Dev Biol. 2015; 3:63. | | | | | | | | | |
| 93. Jiang J, Mohan P, Maxwell CA. The cytoskeletal protein RHAMM and ERK1/2 activity maintain the pluripotency of murine embryonic stem cells. PLoS One. 2013; 8:e73548. | | | | | | | | | |
| 94. Kouvidi K, Berdiaki A, Nikitovic D, Katonis P, Afratis N, Hascall VC, Karamanos NK, Tzanakakis GN. Role of receptor for hyaluronic acid-mediated motility (RHAMM) in low molecular weight hyaluronan (LMWHA)-mediated fibrosarcoma cell adhesion. J Biol Chem. 2011; 286:38509-20. | | | | | | | | | |
| 95. Maxwell CA, Benítez J, Gómez-Baldó L, Osorio A, Bonifaci N, Fernández-Ramires R, Costes SV, Guinó E, Chen H, Evans GJ, Mohan P, Català I, Petit A, et al. Interplay between BRCA1 and RHAMM regulates epithelial apicobasal polarization and may influence risk of breast cancer. PLoS Biol. 2011; 9:e1001199. | | | | | | | | | |
| 96. Samuel SK, Hurta RA, Spearman MA, Wright JA, Turley EA, Greenberg AH. TGF-beta 1 stimulation of cell locomotion utilizes the hyaluronan receptor RHAMM and hyaluronan. J Cell Biol. 1993; 123:749-58. | | | | | | | | | |
| 97. Hamilton SR, Fard SF, Paiwand FF, Tolg C, Veiseh M, Wang C, McCarthy JB, Bissell MJ, Koropatnick J, Turley EA. The hyaluronan receptors CD44 and Rhamm (CD168) form complexes with ERK1,2 that sustain high basal motility in breast cancer cells. J Biol Chem. 2007; 282:16667-80. | | | | | | | | | |
| 98. Tolg C, Hamilton SR, Nakrieko KA, Kooshesh F, Walton P, McCarthy JB, Bissell MJ, Turley EA. Rhamm-/- fibroblasts are defective in CD44-mediated ERK1,2 motogenic signaling, leading to defective skin wound repair. J Cell Biol. 2006; 175:1017-28. | | | | | | | | | |
| 99. Missinato MA, Tobita K, Romano N, Carroll JA, Tsang M. Extracellular component hyaluronic acid and its receptor Hmmr are required for epicardial EMT during heart regeneration. Cardiovasc Res. 2015; 107:487-98. | | | | | | | | | |
| 100. Truong JL, Liu M, Tolg C, Barr M, Dai C, Raissi TC, Wong E, DeLyzer T, Yazdani A, Turley EA. Creating a Favorable Microenvironment for Fat Grafting in a Novel Model of Radiation-Induced Mammary Fat Pad Fibrosis. Plast Reconstr Surg. 2020; 145:116-26. | | | | | | | | | |
| 101. Cui Z, Liao J, Cheong N, Longoria C, Cao G, DeLisser HM, Savani RC. The Receptor for Hyaluronan-Mediated Motility (CD168) promotes inflammation and fibrosis after acute lung injury. Matrix Biol. 2019; 78-79:255-71. | | | | | | | | | |
| 102. Baugh L, Watson MC, Kemmerling EC, Hinds PW, Huggins GS, Black LD. Knockdown of CD44 expression decreases valve interstitial cell calcification in vitro. Am J Physiol Heart Circ Physiol. 2019; 317:H26-H36. | | | | | | | | | |
| 103. Li H, Moll J, Winkler A, Frappart L, Brunet S, Hamann J, Kroll T, Verlhac MH, Heuer H, Herrlich P, Ploubidou A. RHAMM deficiency disrupts folliculogenesis resulting in female hypofertility. Biol Open. 2015; 4:562-71. | | | | | | | | | |
| 104. Connell M, Chen H, Jiang J, Kuan CW, Fotovati A, Chu TL, He Z, Lengyell TC, Li H, Kroll T, Li AM, Goldowitz D, Frappart L, et al. HMMR acts in the PLK1-dependent spindle positioning pathway and supports neural development. ELife. 2017; 6:e28672. | | | | | | | | | |
| 105. Prager A, Hagenlocher C, Ott T, Schambony A, Feistel K. hmmr mediates anterior neural tube closure and morphogenesis in the frog Xenopus. Dev Biol. 2017; 430:188-201. | | | | | | | | | |
| 106. Tolg C, Yuan H, Flynn SM, Basu K, Ma J, Tse KCK, Kowalska B, Vulkanesku D, Cowman MK, McCarthy JB, Turley EA. Hyaluronan modulates growth factor induced mammary gland branching in a size dependent manner. Matrix Biol. 2017; 63:117-32. | | | | | | | | | |
| 107. Bahrami SB, Tolg C, Peart T, Symonette C, Veiseh M, Umoh JU, Holdsworth DW, McCarthy JB, Luyt LG, Bissell MJ, Yazdani A, Turley EA. Receptor for hyaluronan mediated motility (RHAMM/HMMR) is a novel target for promoting subcutaneous adipogenesis. Integr Biol (Camb). 2017; 9:223-37. | | | | | | | | | |
| 108. Zhang H, Ren L, Ding Y, Li F, Chen X, Ouyang Y, Zhang Y, Zhang D. Hyaluronan-mediated motility receptor confers resistance to chemotherapy via TGFβ/Smad2-induced epithelial-mesenchymal transition in gastric cancer. FASEB J. 2019; 33:6365-77. | | | | | | | | | |
| 109. Blanco I, Kuchenbaecker K, Cuadras D, Wang X, Barrowdale D, de Garibay GR, Librado P, Sánchez-Gracia A, Rozas J, Bonifaci N, McGuffog L, Pankratz VS, Islam A, et al. Assessing associations between the AURKA-HMMR-TPX2-TUBG1 functional module and breast cancer risk in BRCA1/2 mutation carriers. PLoS One. 2015; 10:e0120020. | | | | | | | | | |
| 110. Tilghman J, Wu H, Sang Y, Shi X, Guerrero-Cazares H, Quinones-Hinojosa A, Eberhart CG, Laterra J, Ying M. HMMR maintains the stemness and tumorigenicity of glioblastoma stem-like cells. Cancer Res. 2014; 74:3168-79. | | | | | | | | | |
| 111. Stafford JL, Dyson G, Levin NK, Chaudhry S, Rosati R, Kalpage H, Wernette C, Petrucelli N, Simon MS, Tainsky MA. Reanalysis of BRCA1/2 negative high risk ovarian cancer patients reveals novel germline risk loci and insights into missing heritability. PLoS One. 2017; 12:e0178450. | | | | | | | | | |
| 112. Stevens LE, Cheung WKC, Adua SJ, Arnal-Estapé A, Zhao M, Liu Z, Brewer K, Herbst RS, Nguyen DX. Extracellular Matrix Receptor Expression in Subtypes of Lung Adenocarcinoma Potentiates Outgrowth of Micrometastases. Cancer Res. 2017; 77:1905-17. | | | | | | | | | |
| 113. Tano K, Mizuno R, Okada T, Rakwal R, Shibato J, Masuo Y, Ijiri K, Akimitsu N. MALAT-1 enhances cell motility of lung adenocarcinoma cells by influencing the expression of motility-related genes. FEBS Lett. 2010; 584:4575-80. | | | | | | | | | |
| 114. Mantripragada KK, Spurlock G, Kluwe L, Chuzhanova N, Ferner RE, Frayling IM, Dumanski JP, Guha A, Mautner V, Upadhyaya M. High-resolution DNA copy number profiling of malignant peripheral nerve sheath tumors using targeted microarray-based comparative genomic hybridization. Clin Cancer Res. 2008; 14:1015-24. | | | | | | | | | |
| 115. Thangavel C, Boopathi E, Liu Y, Haber A, Ertel A, Bhardwaj A, Addya S, Williams N, Ciment SJ, Cotzia P, Dean JL, Snook A, McNair C, et al. RB Loss Promotes Prostate Cancer Metastasis. Cancer Res. 2017; 77:982-95. | | | | | | | | | |
| 116. Oldenburg D, Ru Y, Weinhaus B, Cash S, Theodorescu D, Guin S. CD44 and RHAMM are essential for rapid growth of bladder cancer driven by loss of Glycogen Debranching Enzyme (AGL). BMC Cancer. 2016; 16:713. | | | | | | | | | |
| 117. Mele V, Sokol L, Kölzer VH, Pfaff D, Muraro MG, Keller I, Stefan Z, Centeno I, Terracciano LM, Dawson H, Zlobec I, Iezzi G, Lugli A. The hyaluronan-mediated motility receptor RHAMM promotes growth, invasiveness and dissemination of colorectal cancer. Oncotarget. 2017; 8:70617-29. | | | | | | | | | |
| 118. Mohan P, Castellsague J, Jiang J, Allen K, Chen H, Nemirovsky O, Spyra M, Hu K, Kluwe L, Pujana MA, Villanueva A, Mautner VF, Keats JJ, et al. Genomic imbalance of HMMR/RHAMM regulates the sensitivity and response of malignant peripheral nerve sheath tumour cells to aurora kinase inhibition. Oncotarget. 2013; 4:80-93. | | | | | | | | | |
| 119. Bidadi B, Liu D, Kalari KR, Rubner M, Hein A, Beckmann MW, Rack B, Janni W, Fasching PA, Weinshilboum RM, Wang L. Pathway-Based Analysis of Genome-Wide Association Data Identified SNPs in HMMR as Biomarker for Chemotherapy-Induced Neutropenia in Breast Cancer Patients. Front Pharmacol. 2018; 9:158. | | | | | | | | | |
| 120. Xu F, Chang K, Ma J, Qu Y, Xie H, Dai B, Gan H, Zhang H, Shi G, Zhu Y, Shen Y, Ye D. The Oncogenic Role of COL23A1 in Clear Cell Renal Cell Carcinoma. Sci Rep. 2017; 7:9846. | | | | | | | | | |
| 121. Altorok N, Tsou PS, Coit P, Khanna D, Sawalha AH. Genome-wide DNA methylation analysis in dermal fibroblasts from patients with diffuse and limited systemic sclerosis reveals common and subset-specific DNA methylation aberrancies. Ann Rheum Dis. 2015; 74:1612-20. | | | | | | | | | |
| 122. Qiao L, Yang Y, Fu P, Hu S, Zhou H, Peng S, Tan J, Lu Y, Lou H, Lu D, Wu S, Guo J, Jin L, et al. Genome-wide variants of Eurasian facial shape differentiation and a prospective model of DNA based face prediction. J Genet Genomics. 2018; 45:419-32. | | | | | | | | | |
| 123. Roumans NJ, Vink RG, Gielen M, Zeegers MP, Holst C, Wang P, Astrup A, Saris WH, Valsesia A, Hager J, van Baak MA, Mariman EC. Variation in extracellular matrix genes is associated with weight regain after weight loss in a sex-specific manner. Genes Nutr. 2015; 10:56. | | | | | | | | | |
| 124. Yang Z, Shen L, Dangel AW, Wu LC, Yu CY. Four ubiquitously expressed genes, RD (D6S45)-SKI2W (SKIV2L)-DOM3Z-RP1 (D6S60E), are present between complement component genes factor B and C4 in the class III region of the HLA. Genomics. 1998; 53:338-47. | | | | | | | | | |
| 125. Yang Z, Qu X, Yu CY. Features of the two gene pairs RD-SKI2W and DOM3Z-RP1 located between complement component genes factor B and C4 at the MHC class III region. Front Biosci. 2001; 6:D927-35. | | | | | | | | | |
| 126. Eckard SC, Rice GI, Fabre A, Badens C, Gray EE, Hartley JL, Crow YJ, Stetson DB. The SKIV2L RNA exosome limits activation of the RIG-I-like receptors. Nat Immunol. 2014; 15:839-45. | | | | | | | | | |
| 127. Fernando MM, Stevens CR, Sabeti PC, Walsh EC, McWhinnie AJ, Shah A, Green T, Rioux JD, Vyse TJ. Identification of two independent risk factors for lupus within the MHC in United Kingdom families. PLoS Genet. 2007; 3:e192. | | | | | | | | | |
| 128. Zhou R, Lin X, Li DY, Wang XF, Greenbaum J, Chen YC, Zeng CP, Lu JM, Ao ZX, Peng LP, Bai XC, Shen J, Deng HW. Identification of novel genetic loci for osteoporosis and/or rheumatoid arthritis using cFDR approach. PLoS One. 2017; 12:e0183842. | | | | | | | | | |
| 129. Ashton JJ, Andreoletti G, Coelho T, Haggarty R, Batra A, Afzal NA, Beattie RM, Ennis S. Identification of Variants in Genes Associated with Single-gene Inflammatory Bowel Disease by Whole-exome Sequencing. Inflamm Bowel Dis. 2016; 22:2317-27. | | | | | | | | | |
| 130. Bourgeois P, Esteve C, Chaix C, Béroud C, Lévy N, Fabre A, Badens C, consortium Tc. Tricho-Hepato-Enteric Syndrome mutation update: Mutations spectrum of TTC37 and SKIV2L, clinical analysis and future prospects. Hum Mutat. 2018; 39:774-89. | | | | | | | | | |
| 131. Morgan NV, Hartley JL, Setchell KD, Simpson MA, Brown R, Tee L, Kirkham S, Pasha S, Trembath RC, Maher ER, Gissen P, Kelly DA. A combination of mutations in AKR1D1 and SKIV2L in a family with severe infantile liver disease. Orphanet J Rare Dis. 2013; 8:74. | | | | | | | | | |
| 132. Shuai P, Ye Z, Liu Y, Qu C, Liu X, Luo H, Feng X, Li X, Shi Y, Gong B. Association between SKIV2L polymorphism rs429608 and age-related macular degeneration: A meta-analysis. Ophthalmic Genet. 2017; 38:245-51. | | | | | | | | | |
| 133. Ma L, Li Z, Liu K, Rong SS, Brelen ME, Young AL, Kumaramanickavel G, Pang CP, Chen H, Chen LJ. Association of Genetic Variants with Polypoidal Choroidal Vasculopathy: A Systematic Review and Updated Meta-analysis. Ophthalmology. 2015; 122:1854-65. | | | | | | | | | |
| 134. Ambrose RL, Liu YC, Adams TE, Bean AGD, Stewart CR. C6orf106 is a novel inhibitor of the interferon-regulatory factor 3-dependent innate antiviral response. J Biol Chem. 2018; 293:10561-73. | | | | | | | | | |
| 135. Li X, Dong M, Zhou J, Zhu D, Zhao J, Sheng W. C6orf106 accelerates pancreatic cancer cell invasion and proliferation via activating ERK signaling pathway. Mol Cell Biochem. 2019; 454:87-95. | | | | | | | | | |
| 136. Ahmad S, Poveda A, Shungin D, Barroso I, Hallmans G, Renström F, Franks PW. Established BMI-associated genetic variants and their prospective associations with BMI and other cardiometabolic traits: the GLACIER Study. Int J Obes (Lond). 2016; 40:1346-52. | | | | | | | | | |
| 137. Horani A, Ustione A, Huang T, Firth AL, Pan J, Gunsten SP, Haspel JA, Piston DW, Brody SL Establishment of the early cilia preassembly protein complex during motile ciliogenesis. Proc Natl Acad Sci U S A. 2018; 115:E1221-E8. | | | | | | | | | |
| 138. Horani A, Druley TE, Zariwala MA, Patel AC, Levinson BT, Van Arendonk LG, Thornton KC, Giacalone JC, Albee AJ, Wilson KS, Turner EH, Nickerson DA, Shendure J, et al. Whole-exome capture and sequencing identifies HEATR2 mutation as a cause of primary ciliary dyskinesia. Am J Hum Genet. 2012; 91:685-93. | | | | | | | | | |
| 139. Adams AK, Smith SD, Truong DT, Willcutt EG, Olson RK, DeFries JC, Pennington BF, Gruen JR. Enrichment of putatively damaging rare variants in the DYX2 locus and the reading-related genes CCDC136 and FLNC. Hum Genet. 2017; 136:1395-405. | | | | | | | | | |
| 140. Geng Q, Ni L, Ouyang B, Hu Y, Zhao Y, Guo J. A Novel Testis-Specific Gene, Ccdc136, Is Required for Acrosome Formation and Fertilization in Mice. Reprod Sci. 2016; 23:1387-96. | | | | | | | | | |
| 141. Baumgart S, Jansen F, Bintig W, Kalbe B, Herrmann C, Klumpers F, Köster SD, Scholz P, Rasche S, Dooley R, Metzler-Nolte N, Spehr M, Hatt H, et al. The scaffold protein MUPP1 regulates odorant-mediated signaling in olfactory sensory neurons. J Cell Sci. 2014; 127:2518-27. | | | | | | | | | |
| 142. Guillaume JL, Daulat AM, Maurice P, Levoye A, Migaud M, Brydon L, Malpaux B, Borg-Capra C, Jockers R. The PDZ protein mupp1 promotes Gi coupling and signaling of the Mt1 melatonin receptor. J Biol Chem. 2008; 283:16762-71. | | | | | | | | | |
| 143. Sindic A, Huang C, Chen AP, Ding Y, Miller-Little WA, Che D, Romero MF, Miller RT. MUPP1 complexes renal K+ channels to alter cell surface expression and whole cell currents. Am J Physiol Renal Physiol. 2009; 297:F36-45. | | | | | | | | | |
| 144. Ackermann F, Zitranski N, Heydecke D, Wilhelm B, Gudermann T, Boekhoff I. The Multi-PDZ domain protein MUPP1 as a lipid raft-associated scaffolding protein controlling the acrosome reaction in mammalian spermatozoa. J Cell Physiol. 2008; 214:757-68. | | | | | | | | | |
| 145. Assémat E, Crost E, Ponserre M, Wijnholds J, Le Bivic A, Massey-Harroche D. The multi-PDZ domain protein-1 (MUPP-1) expression regulates cellular levels of the PALS-1/PATJ polarity complex. Exp Cell Res. 2013; 319:2514-25. | | | | | | | | | |
| 146. Massimi P, Gammoh N, Thomas M, Banks L. HPV E6 specifically targets different cellular pools of its PDZ domain-containing tumour suppressor substrates for proteasome-mediated degradation. Oncogene. 2004; 23:8033-9. | | | | | | | | | |
| 147. Ngok SP, Geyer R, Liu M, Kourtidis A, Agrawal S, Wu C, Seerapu HR, Lewis-Tuffin LJ, Moodie KL, Huveldt D, Marx R, Baraban JM, Storz P, et al. VEGF and Angiopoietin-1 exert opposing effects on cell junctions by regulating the Rho GEF Syx. J Cell Biol. 2012; 199:1103-15. | | | | | | | | | |
| 148. Balasubramanian S, Fam SR, Hall RA. GABAB receptor association with the PDZ scaffold Mupp1 alters receptor stability and function. J Biol Chem. 2007; 282:4162-71. | | | | | | | | | |
| 149. Reilly MT, Milner LC, Shirley RL, Crabbe JC, Buck KJ. 5-HT2C and GABAB receptors influence handling-induced convulsion severity in chromosome 4 congenic and DBA/2J background strain mice. Brain Res. 2008; 1198:124-31. | | | | | | | | | |
| 150. Marivin A, Garcia-Marcos M. DAPLE and MPDZ bind to each other and cooperate to promote apical cell constriction. Mol Biol Cell. 2019; 30:1900-10. | | | | | | | | | |
| 151. Yang J, Simonneau C, Kilker R, Oakley L, Byrne MD, Nichtova Z, Stefanescu I, Pardeep-Kumar F, Tripathi S, Londin E, Saugier-Veber P, Willard B, Thakur M, et al. Murine MPDZ-linked hydrocephalus is caused by hyperpermeability of the choroid plexus. EMBO Mol Med. 2019; 11:e9540. | | | | | | | | | |
| 152. Harripaul R, Vasli N, Mikhailov A, Rafiq MA, Mittal K, Windpassinger C, Sheikh TI, Noor A, Mahmood H, Downey S, Johnson M, Vleuten K, Bell L, et al. Mapping autosomal recessive intellectual disability: combined microarray and exome sequencing identifies 26 novel candidate genes in 192 consanguineous families. Mol Psychiatry. 2018; 23:973-84. | | | | | | | | | |
| 153. Fromer M, Pocklington AJ, Kavanagh DH, Williams HJ, Dwyer S, Gormley P, Georgieva L, Rees E, Palta P, Ruderfer DM, Carrera N, Humphreys I, Johnson JS, et al. De novo mutations in schizophrenia implicate synaptic networks. Nature. 2014; 506:179-84. | | | | | | | | | |
| 154. Donaldson R, Sun Y, Liang DY, Zheng M, Sahbaie P, Dill DL, Peltz G, Buck KJ, Clark JD. The multiple PDZ domain protein Mpdz/MUPP1 regulates opioid tolerance and opioid-induced hyperalgesia. BMC Genomics. 2016; 17:313. | | | | | | | | | |
| 155. Karpyak VM, Kim JH, Biernacka JM, Wieben ED, Mrazek DA, Black JL, Choi DS. Sequence variations of the human MPDZ gene and association with alcoholism in subjects with European ancestry. Alcohol Clin Exp Res. 2009; 33:712-21. | | | | | | | | | |
| 156. Milner LC, Shirley RL, Kozell LB, Walter NA, Kruse LC, Komiyama NH, Grant SG, Buck KJ. Novel MPDZ/MUPP1 transgenic and knockdown models confirm Mpdz's role in ethanol withdrawal and support its role in voluntary ethanol consumption. Addict Biol. 2015; 20:143-7. | | | | | | | | | |
| 157. Smith SB, Mir E, Bair E, Slade GD, Dubner R, Fillingim RB, Greenspan JD, Ohrbach R, Knott C, Weir B, Maixner W, Diatchenko L. Genetic variants associated with development of TMD and its intermediate phenotypes: the genetic architecture of TMD in the OPPERA prospective cohort study. J Pain. 2013; 14:T91-101.e1-3. | | | | | | | | | |
| 158. Moazzeni H, Javadi MA, Asgari D, Khani M, Emami M, Moghadam A, Panahi-Bazaz MR, Hosseini Tehrani M, Karimian F, Hosseini B, Nekuie Moghadam T, Hassanpour H, Akbari MT, et al. Observation of nine previously reported and 10 non-reported SLC4A11 mutations among 20 Iranian CHED probands and identification of an MPDZ mutation as possible cause of CHED and FECD in one family. Br J Ophthalmol. 2019. [Epub ahead of print]. http://dx.doi.org/10.1136/bjophthalmol-2019-314377. | | | | | | | | | |
| 159. Liskova P, Dudakova L, Krepelova A, Klema J, Hysi PG. Replication of SNP associations with keratoconus in a Czech cohort. PLoS One. 2017; 12:e0172365. | | | | | | | | | |
| 160. Tetzlaff F, Adam MG, Feldner A, Moll I, Menuchin A, Rodriguez-Vita J, Sprinzak D, Fischer A. MPDZ promotes DLL4-induced Notch signaling during angiogenesis. Elife. 2018; 7:e32860. | | | | | | | | | |
| 161. Yoo EH, Park KJ, Won HH, Park JH, Lee ST, Kim HJ, Bang SM, Chi HS, Jung CW, Kim SH, Yun H, Sun CH, Park I, et al. Genetic Characteristics of Polycythemia Vera and Essential Thrombocythemia in Korean Patients. J Clin Lab Anal. 2016; 30:1061-70. | | | | | | | | | |
| 162. Wierer M, Prestel M, Schiller HB, Yan G, Schaab C, Azghandi S, Werner J, Kessler T, Malik R, Murgia M, Aherrahrou Z, Schunkert H, Dichgans M, et al. Compartment-resolved Proteomic Analysis of Mouse Aorta during Atherosclerotic Plaque Formation Reveals Osteoclast-specific Protein Expression. Mol Cell Proteomics. 2018; 17:321-34. | | | | | | | | | |
| 163. Kuniba H, Yoshiura K, Kondoh T, Ohashi H, Kurosawa K, Tonoki H, Nagai T, Okamoto N, Kato M, Fukushima Y, Kaname T, Naritomi K, Matsumoto T, et al. Molecular karyotyping in 17 patients and mutation screening in 41 patients with Kabuki syndrome. J Hum Genet. 2009; 54:304-9. | | | | | | | | | |
| 164. Anazi S, Maddirevula S, Faqeih E, Alsedairy H, Alzahrani F, Shamseldin HE, Patel N, Hashem M, Ibrahim N, Abdulwahab F, Ewida N, Alsaif HS, Al Sharif H, et al. Clinical genomics expands the morbid genome of intellectual disability and offers a high diagnostic yield. Mol Psychiatry. 2017; 22:615-24. | | | | | | | | | |
| 165. Unverricht-Yeboah M, Giesen U, Kriehuber R. Comparative gene expression analysis after exposure to 123I-iododeoxyuridine, γ- and α-radiation-potential biomarkers for the discrimination of radiation qualities. J Radiat Res. 2018; 59:411-29. | | | | | | | | | |
| 166. Nicoll WS, Botha M, McNamara C, Schlange M, Pesce ER, Boshoff A, Ludewig MH, Zimmermann R, Cheetham ME, Chapple JP, Blatch GL. Cytosolic and ER J-domains of mammalian and parasitic origin can functionally interact with DnaK. Int J Biochem Cell Biol. 2007; 39:736-51. | | | | | | | | | |
| 167. Nakatsuka A, Wada J, Iseda I, Teshigawara S, Higashio K, Murakami K, Kanzaki M, Inoue K, Terami T, Katayama A, Hida K, Eguchi J, Horiguchi CS, et al. Vaspin is an adipokine ameliorating ER stress in obesity as a ligand for cell-surface GRP78/MTJ-1 complex. Diabetes. 2012; 61:2823-32. | | | | | | | | | |
| 168. Birukova AA, Singleton PA, Gawlak G, Tian X, Mirzapoiazova T, Mambetsariev B, Dubrovskyi O, Oskolkova OV, Bochkov VN, Birukov KG. GRP78 is a novel receptor initiating a vascular barrier protective response to oxidized phospholipids. Mol Biol Cell. 2014; 25:2006-16. | | | | | | | | | |
| 169. Akhabir L, Sandford AJ. Genome-wide association studies for discovery of genes involved in asthma. Respirology. 2011; 16:396-406. | | | | | | | | | |
| 170. Ramos-Lopez O, Riezu-Boj JI, Milagro FI, Martinez JA, Project M. DNA methylation signatures at endoplasmic reticulum stress genes are associated with adiposity and insulin resistance. Mol Genet Metab. 2018; 123:50-8. | | | | | | | | | |
| 171. Sung YJ, Pérusse L, Sarzynski MA, Fornage M, Sidney S, Sternfeld B, Rice T, Terry JG, Jacobs DR, Katzmarzyk P, Curran JE, Jeffrey Carr J, Blangero J, et al. Genome-wide association studies suggest sex-specific loci associated with abdominal and visceral fat. Int J Obes (Lond). 2016; 40:662-74. | | | | | | | | | |
| 172. Zhang R, Lin P, Yang X, He RQ, Wu HY, Dang YW, Gu YY, Peng ZG, Feng ZB, Chen G. Survival associated alternative splicing events in diffuse large B-cell lymphoma. Am J Transl Res. 2018; 10:2636-47. | | | | | | | | | |
| 173. Fraile JM, Campos-Iglesias D, Rodríguez F, Español Y, Freije JM. The deubiquitinase USP54 is overexpressed in colorectal cancer stem cells and promotes intestinal tumorigenesis. Oncotarget. 2016; 7:74427-34. | | | | | | | | | |
| 174. Holmegard HN, Theilade J, Benn M, Duno M, Haunso S, Svendsen JH. Genetic variation in the inwardly rectifying K channel subunits KCNJ3 (GIRK1) and KCNJ5 (GIRK4) in patients with sinus node dysfunction. Cardiology. 2010; 115:176-81. | | | | | | | | | |
| 175. Hardege I, Long L, Al Maskari R, Figg N, O'Shaughnessy KM. Targeted disruption of the Kcnj5 gene in the female mouse lowers aldosterone levels. Clin Sci (Lond). 2018; 132:145-56. | | | | | | | | | |
| 176..Seidel E, Schewe J, Scholl UI. Genetic causes of primary aldosteronism. Exp Mol Med. 2019; 51:1-12. | | | | | | | | | |
| 177. Kulkarni K, Xie X, Marron Fernandez de Velasco E, Anderson A, Martemyanov KA, Wickman K, Tolkacheva EG. The influences of the M2R-GIRK4-RGS6 dependent parasympathetic pathway on electrophysiological properties of the mouse heart. PLoS One. 2018; 13:e0193798. | | | | | | | | | |
| 178. Kuß J, Stallmeyer B, Goldstein M, Rinné S, Pees C, Zumhagen S, Seebohm G, Decher N, Pott L, Kienitz MC, Schulze-Bahr E. Familial Sinus Node Disease Caused by a Gain of GIRK (G-Protein Activated Inwardly Rectifying K+ Channel) Channel Function. Circ Genom Precis Med. 2019; 12:e002238. | | | | | | | | | |
| 179. Yamada N, Asano Y, Fujita M, Yamazaki S, Inanobe A, Matsuura N, Kobayashi H, Ohno S, Ebana Y, Tsukamoto O, Ishino S, Takuwa A, Kioka H, et al. Mutant KCNJ3 and KCNJ5 Potassium Channels as Novel Molecular Targets in Bradyarrhythmias and Atrial Fibrillation. Circulation. 2019; 139:2157-69. | | | | | | | | | |
| 180. Kokunai Y, Nakata T, Furuta M, Sakata S, Kimura H, Aiba T, Yoshinaga M, Osaki Y, Nakamori M, Itoh H, Sato T, Kubota T, Kadota K, et al. A Kir3.4 mutation causes Andersen-Tawil syndrome by an inhibitory effect on Kir2.1. Neurology. 2014; 82:1058-64. | | | | | | | | | |
| 181. Wang F, Liu J, Hong L, Liang B, Graff C, Yang Y, Christiansen M, Olesen SP, Zhang L, Kanters JK. The phenotype characteristics of type 13 long QT syndrome with mutation in KCNJ5 (Kir3.4-G387R). Heart Rhythm. 2013; 10:1500-6. | | | | | | | | | |
| 182. Li N, Zhang D, Zhang J, Guo Y, Yan Z, Wang H, Zhou L, Hong J, Wang X, A Z. Influence of age on the association of GIRK4 with metabolic syndrome. Ann Clin Biochem. 2012; 49:369-76. | | | | | | | | | |
| 183. Perry CA, Pravetoni M, Teske JA, Aguado C, Erickson DJ, Medrano JF, Luján R, Kotz CM, Wickman K. Predisposition to late-onset obesity in GIRK4 knockout mice. Proc Natl Acad Sci U S A. 2008; 105:8148-53. | | | | | | | | | |
| 184. Laqqan M, Solomayer EF, Hammadeh M. Aberrations in sperm DNA methylation patterns are associated with abnormalities in semen parameters of subfertile males. Reprod Biol. 2017; 17:246-51. | | | | | | | | | |
| 185. Charron S, Roubertie F, Benoist D, Dubes V, Gilbert SH, Constantin M, Vieillot D, Elbes D, Quesson B, Bordachar P, Haissaguerre M, Bernus O, Thambo JB, et al. Identification of Region-Specific Myocardial Gene Expression Patterns in a Chronic Swine Model of Repaired Tetralogy of Fallot. PLoS One. 2015; 10:e0134146. | | | | | | | | | |
| 186. Han Y, Chen JD, Liu ZM, Zhou Y, Xia JH, Du XL, Jin MW. Functional ion channels in mouse cardiac c-kit(+) cells. Am J Physiol Cell Physiol. 2010; 298:C1109-17. | | | | | | | | | |
| 187. Vautier F, Belachew S, Chittajallu R, Gallo V. Shaker-type potassium channel subunits differentially control oligodendrocyte progenitor proliferation. Glia. 2004; 48:337-45. | | | | | | | | | |
| 188. Silver K, Littlejohn A, Thomas L, Marsh E, Lillich JD. Inhibition of Kv channel expression by NSAIDs depolarizes membrane potential and inhibits cell migration by disrupting calpain signaling. Biochem Pharmacol. 2015; 98:614-28. | | | | | | | | | |
| 189. Raimondi L, Alfarano C, Pacini A, Livi S, Ghelardini C, DeSiena G, Pirisino R. Methylamine-dependent release of nitric oxide and dopamine in the CNS modulates food intake in fasting rats. Br J Pharmacol. 2007; 150:1003-10. | | | | | | | | | |
| 190. O'Donnell AF, McCartney RR, Chandrashekarappa DG, Zhang BB, Thorner J, Schmidt MC. 2-Deoxyglucose impairs Saccharomyces cerevisiae growth by stimulating Snf1-regulated and α-arrestin-mediated trafficking of hexose transporters 1 and 3. Mol Cell Biol. 2015; 35:939-55. | | | | | | | | | |
| 191. Au E, Ahmed T, Karayannis T, Biswas S, Gan L, Fishell G. A modular gain-of-function approach to generate cortical interneuron subtypes from ES cells. Neuron. 2013; 80:1145-58. | | | | | | | | | |
| 192. Isogai E, Okumura K, Saito M, Yoshizawa Y, Itoh K, Tando S, Ohira M, Haraguchi S, Nakagawara A, Fushiki S, Nagase H, Wakabayashi Y. Oncogenic Lmo3 cooperates with Hen2 to induce hydrocephalus in mice. Exp Anim. 2015; 64:407-14. | | | | | | | | | |
| 193. Reisinger SN, Bilban M, Stojanovic T, Derdak S, Yang J, Cicvaric A, Horvath O, Sideromenos S, Zambon A, Monje FJ, Boehm S, Pollak DD. Lmo3 deficiency in the mouse is associated with alterations in mood-related behaviors and a depression-biased amygdala transcriptome. Psychoneuroendocrinology. 2020; 111:104480. | | | | | | | | | |
| 194. Savarese A, Lasek AW. Regulation of anxiety-like behavior and Crhr1 expression in the basolateral amygdala by LMO3. Psychoneuroendocrinology. 2018; 92:13-20. | | | | | | | | | |
| 195. Savarese A, Zou ME, Kharazia V, Maiya R, Lasek AW. Increased behavioral responses to ethanol in Lmo3 knockout mice. Genes Brain Behav. 2014; 13:777-83. | | | | | | | | | |
| 196. Sun W, Yu S, Han H, Yuan Q, Chen J, Xu G. Resveratrol Inhibits Human Visceral Preadipocyte Proliferation and Differentiation in vitro. Lipids. 2019; 54:679-86. | | | | | | | | | |
| 197. Lindroos J, Husa J, Mitterer G, Haschemi A, Rauscher S, Haas R, Gröger M, Loewe R, Kohrgruber N, Schrögendorfer KF, Prager G, Beck H, Pospisilik JA, et al. Human but not mouse adipogenesis is critically dependent on LMO3. Cell Metab. 2013; 18:62-74. | | | | | | | | | |
| 198. Larsen S, Yokochi T, Isogai E, Nakamura Y, Ozaki T, Nakagawara A. LMO3 interacts with p53 and inhibits its transcriptional activity. Biochem Biophys Res Commun. 2010; 392:252-7. | | | | | | | | | |
| 199. Hui L, Ji C, Hui B, Lv T, Ha X, Yang J, Cai W. The oncoprotein LMO3 interacts with calcium- and integrin-binding protein CIB. Brain Res. 2009; 1265:24-9. | | | | | | | | | |
| 200. Chen D, Zhang Y, Lin Y, Shen F, Zhang Z, Zhou J. MicroRNA-382 inhibits cancer cell growth and metastasis in NSCLC via targeting LMO3. Exp Ther Med. 2019; 17:2417-24. | | | | | | | | | |
| 201. Qiu YS, Jiang NN, Zhou Y, Yu KY, Gong HY, Liao GJ. LMO3 promotes gastric cancer cell invasion and proliferation through Akt-mTOR and Akt-GSK3β signaling. Int J Mol Med. 2018; 41:2755-63. | | | | | | | | | |
| 202. Cheng Y, Hou T, Ping J, Chen T, Yin B. LMO3 promotes hepatocellular carcinoma invasion, metastasis and anoikis inhibition by directly interacting with LATS1 and suppressing Hippo signaling. J Exp Clin Cancer Res. 2018; 37:228. | | | | | | | | | |
| 203. Shepherd C, Skelton AJ, Rushton MD, Reynard LN, Loughlin J. Expression analysis of the osteoarthritis genetic susceptibility locus mapping to an intron of the MCF2L gene and marked by the polymorphism rs11842874. BMC Med Genet. 2015; 16:108. | | | | | | | | | |
| 204. Kortlever RM, Brummelkamp TR, van Meeteren LA, Moolenaar WH, Bernards R. Suppression of the p53-dependent replicative senescence response by lysophosphatidic acid signaling. Mol Cancer Res. 2008; 6:1452-60. | | | | | | | | | |
| 205. Ieguchi K, Ueda S, Kataoka T, Satoh T. Role of the guanine nucleotide exchange factor Ost in negative regulation of receptor endocytosis by the small GTPase Rac1. J Biol Chem. 2007; 282:23296-305. | | | | | | | | | |
| 206. Hayashi T, Yoshida T, Ra M, Taguchi R, Mishina M. IL1RAPL1 associated with mental retardation and autism regulates the formation and stabilization of glutamatergic synapses of cortical neurons through RhoA signaling pathway. PLoS One. 2013; 8:e66254. | | | | | | | | | |
| 207. Montaner S, Perona R, Saniger L, Lacal JC. Multiple signalling pathways lead to the activation of the nuclear factor kappaB by the Rho family of GTPases. J Biol Chem. 1998; 273:12779-85. | | | | | | | | | |
| 208. Yamauchi J, Hirasawa A, Miyamoto Y, Kokubu H, Nishii H, Okamoto M, Sugawara Y, Tsujimoto G, Itoh H. Role of Dbl's big sister in the anti-mitogenic pathway from alpha1B-adrenergic receptor to c-Jun N-terminal kinase. Biochem Biophys Res Commun. 2002; 296:85-92. | | | | | | | | | |
| 209. Klinger MB, Guilbault B, Kay RJ. The RhoA- and CDC42-specific exchange factor Dbs promotes expansion of immature thymocytes and deletion of double-positive and single-positive thymocytes. Eur J Immunol. 2004; 34:806-16. | | | | | | | | | |
| 210. Fitzpatrick ER, Hu T, Ciccarelli BT, Whitehead IP. Regulation of vesicle transport and cell motility by Golgi-localized Dbs. Small GTPases. 2014; 5:1-12. | | | | | | | | | |
| 211. Liu Z, Adams HC3rd, Whitehead IP. The rho-specific guanine nucleotide exchange factor Dbs regulates breast cancer cell migration. J Biol Chem. 2009; 284:15771-80. | | | | | | | | | |
| 212. Figueroa JD, Middlebrooks CD, Banday AR, Ye Y, Garcia-Closas M, Chatterjee N, Koutros S, Kiemeney LA, Rafnar T, Bishop T, Furberg H, Matullo G, Golka K, et al. Identification of a novel susceptibility locus at 13q34 and refinement of the 20p12.2 region as a multi-signal locus associated with bladder cancer risk in individuals of European ancestry. Hum Mol Genet. 2016; 25:1203-14. | | | | | | | | | |
| 213. Maiwald S, Motazacker MM, van Capelleveen JC, Sivapalaratnam S, van der Wal AC, van der Loos C, Kastelein JJ, Ouwehand WH, Hovingh GK, Trip MD, van Buul JD, Dallinga-Thie GM. A rare variant in MCF2L identified using exclusion linkage in a pedigree with premature atherosclerosis. Eur J Hum Genet. 2016; 24:86-91. | | | | | | | | | |
| 214. Hadjidemetriou I, Mariniello K, Ruiz-Babot G, Pittaway J, Mancini A, Mariannis D, Gomez-Sanchez CE, Parvanta L, Drake WM, Chung TT, Abdel-Aziz TE, DiMarco A, Palazzo FF, et al. DLK1/PREF1 marks a novel cell population in the human adrenal cortex. J Steroid Biochem Mol Biol. 2019; 193:105422. | | | | | | | | | |
| 215. González MJ, Ruiz-García A, Monsalve EM, Sánchez-Prieto R, Laborda J, Díaz-Guerra MJ, Ruiz-Hidalgo MJ. DLK1 is a novel inflammatory inhibitor which interferes with NOTCH1 signaling in TLR-activated murine macrophages. Eur J Immunol. 2015; 45:2615-27. | | | | | | | | | |
| 216. Nueda ML, González-Gómez MJ, Rodríguez-Cano MM, Monsalve EM, Díaz-Guerra MJM, Sánchez-Solana B, Laborda J, Baladrón V. DLK proteins modulate NOTCH signaling to influence a brown or white 3T3-L1 adipocyte fate. Sci Rep. 2018; 8:16923. | | | | | | | | | |
| 217. Wang Y, Kim KA, Kim JH, Sul HS. Pref-1, a preadipocyte secreted factor that inhibits adipogenesis. J Nutr. 2006; 136:2953-6. | | | | | | | | | |
| 218. Jensen CH, Kosmina R, Rydén M, Baun C, Hvidsten S, Andersen MS, Christensen LL, Gastaldelli A, Marraccini P, Arner P, Jørgensen CD, Laborda J, Holst JJ, et al. The imprinted gene Delta like non-canonical notch ligand 1 (Dlk1) associates with obesity and triggers insulin resistance through inhibition of skeletal muscle glucose uptake. EBioMedicine. 2019; 46:368-80. | | | | | | | | | |
| 219. Cleaton MA, Dent CL, Howard M, Corish JA, Gutteridge I, Sovio U, Gaccioli F, Takahashi N, Bauer SR, Charnock-Jones DS, Powell TL, Smith GC, Ferguson-Smith AC, et al. Fetus-derived DLK1 is required for maternal metabolic adaptations to pregnancy and is associated with fetal growth restriction. Nat Genet. 2016; 48:1473-80. | | | | | | | | | |
| 220. Charalambous M, Da Rocha ST, Radford EJ, Medina-Gomez G, Curran S, Pinnock SB, Ferrón SR, Vidal-Puig A, Ferguson-Smith AC. DLK1/PREF1 regulates nutrient metabolism and protects from steatosis. Proc Natl Acad Sci U S A. 2014; 111:16088-93. | | | | | | | | | |
| 221. Cheung LY, Rizzoti K, Lovell-Badge R, Le Tissier PR. Pituitary phenotypes of mice lacking the notch signalling ligand delta-like 1 homologue. J Neuroendocrinol. 2013; 25:391-401. | | | | | | | | | |
| 222. Dauber A, Cunha-Silva M, Macedo DB, Brito VN, Abreu AP, Roberts SA, Montenegro LR, Andrew M, Kirby A, Weirauch MT, Labilloy G, Bessa DS, Carroll RS, et al. Paternally Inherited DLK1 Deletion Associated With Familial Central Precocious Puberty. J Clin Endocrinol Metab. 2017; 102:1557-67. | | | | | | | | | |
| 223. Gerlach JC, Thompson RL, Gridelli B, Schmelzer E. Effects of Delta-Like Noncanonical Notch Ligand 1 Expression of Human Fetal Liver Hepatoblasts on Hematopoietic Progenitors. Stem Cells Int. 2019; 2019:7916275. | | | | | | | | | |
| 224. Mirshekar-Syahkal B, Haak E, Kimber GM, van Leusden K, Harvey K, O'Rourke J, Laborda J, Bauer SR, de Bruijn MF, Ferguson-Smith AC, Dzierzak E, Ottersbach K. Dlk1 is a negative regulator of emerging hematopoietic stem and progenitor cells. Haematologica. 2013; 98:163-71. | | | | | | | | | |
| 225. Dong M, Guda K, Nambiar PR, Nakanishi M, Lichtler AC, Nishikawa M, Giardina C, Rosenberg DW. Azoxymethane-induced pre-adipocyte factor 1 (Pref-1) functions as a differentiation inhibitor in colonic epithelial cells. Carcinogenesis. 2004; 25:2239-46. | | | | | | | | | |
| 226. Schober A, Nazari-Jahantigh M, Wei Y, Bidzhekov K, Gremse F, Grommes J, Megens RT, Heyll K, Noels H, Hristov M, Wang S, Kiessling F, Olson EN, et al. MicroRNA-126-5p promotes endothelial proliferation and limits atherosclerosis by suppressing Dlk1. Nat Med. 2014; 20:368-76. | | | | | | | | | |
| 227. Huang CC, Cheng SH, Wu CH, Li WY, Wang JS, Kung ML, Chu TH, Huang ST, Feng CT, Huang SC, Tai MH. Delta-like 1 homologue promotes tumorigenesis and epithelial-mesenchymal transition of ovarian high-grade serous carcinoma through activation of Notch signaling. Oncogene. 2019; 38:3201-15. | | | | | | | | | |
| 228. Kamal M, Valanciute A, Dahan K, Ory V, Pawlak A, Lang P, Guellaen G, Sahali D. C-mip interacts physically with RelA and inhibits nuclear factor kappa B activity. Mol Immunol. 2009; 46:991-8. | | | | | | | | | |
| 229. Oniszczuk J, Sendeyo K, Chhuon C, Savas B, Cogné E, Vachin P, Henique C, Guerrera IC, Astarita G, Frontera V, Pawlak A, Audard V, Sahali D, et al. CMIP is a negative regulator of T cell signaling. Cell Mol Immunol. 2019. [Epub ahead of print]. https://doi.org/10.1038/s41423-019-0266-5. | | | | | | | | | |
| 230. Yu L, Ye J, Liu Q, Feng J, Gu X, Sun Q, Lu G. c‑Maf inducing protein inhibits cofilin‑1 activity and alters podocyte cytoskeleton organization. Mol Med Rep. 2017; 16:4955-63. | | | | | | | | | |
| 231. Liu Y, Su L, Lin Q, Han Y, You P, Fan Q. Induction of C-Mip by IL-17 Plays an Important Role in Adriamycin-Induced Podocyte Damage. Cell Physiol Biochem. 2015; 36:1274-90. | | | | | | | | | |
| 232. Izzedine H, Mangier M, Ory V, Zhang SY, Sendeyo K, Bouachi K, Audard V, Péchoux C, Soria JC, Massard C, Bahleda R, Bourry E, Khayat D, et al. Expression patterns of RelA and c-mip are associated with different glomerular diseases following anti-VEGF therapy. Kidney Int. 2014; 85:457-70. | | | | | | | | | |
| 233. Sendeyo K, Audard V, Zhang SY, Fan Q, Bouachi K, Ollero M, Rucker-Martin C, Gouadon E, Desvaux D, Bridoux F, Guellaën G, Ronco P, Lang P, et al. Upregulation of c-mip is closely related to podocyte dysfunction in membranous nephropathy. Kidney Int. 2013; 83:414-25. | | | | | | | | | |
| 234. Zhang SY, Kamal M, Dahan K, Pawlak A, Ory V, Desvaux D, Audard V, Candelier M, BenMohamed F, Mohamed FB, Matignon M, Christov C, Decrouy X, et al. c-mip impairs podocyte proximal signaling and induces heavy proteinuria. Sci Signal. 2010; 3:ra39. | | | | | | | | | |
| 235. Zhang J, Huang J, Wang X, Chen W, Tang Q, Fang M, Qian Y. CMIP is oncogenic in human gastric cancer cells. Mol Med Rep. 2017; 16:7277-86. | | | | | | | | | |
| 236. Wang B, Wu ZS, Wu Q. CMIP Promotes Proliferation and Metastasis in Human Glioma. Biomed Res Int. 2017; 2017:5340160. | | | | | | | | | |
| 237. Sayols-Baixeras S, Hernáez A, Subirana I, Lluis-Ganella C, Muñoz D, Fitó M, Marrugat J, Elosua R. DNA Methylation and High-Density Lipoprotein Functionality-Brief Report: The REGICOR Study (Registre Gironi del Cor). Arterioscler Thromb Vasc Biol. 2017; 37:567-9. | | | | | | | | | |
| 238. Strawbridge RJ, Laumen H, Hamsten A, Breier M, Grallert H, Hauner H, Arner P, Dahlman I. Effects of Genetic Loci Associated with Central Obesity on Adipocyte Lipolysis. PLoS One. 2016; 11:e0153990. | | | | | | | | | |
| 239. Wu Y, Gao H, Li H, Tabara Y, Nakatochi M, Chiu YF, Park EJ, Wen W, Adair LS, Borja JB, Cai Q, Chang YC, Chen P, et al. A meta-analysis of genome-wide association studies for adiponectin levels in East Asians identifies a novel locus near WDR11-FGFR2. Hum Mol Genet. 2014; 23:1108-19. | | | | | | | | | |
| 240. Cao Y, Wang T, Wu Y, Juan J, Qin X, Tang X, Wu T, Hu Y. Opposite Genetic Effects of CMIP Polymorphisms on the Risk of Type 2 Diabetes and Obesity: A Family-Based Study in China. Int J Mol Sci. 2018; 19:1011. | | | | | | | | | |
| 241. Eicher JD, Gruen JR. Language impairment and dyslexia genes influence language skills in children with autism spectrum disorders. Autism Res. 2015; 8:229-34. | | | | | | | | | |
| 242. Scerri TS, Morris AP, Buckingham LL, Newbury DF, Miller LL, Monaco AP, Bishop DV, Paracchini S. DCDC2, KIAA0319 and CMIP are associated with reading-related traits. Biol Psychiatry. 2011; 70:237-45. | | | | | | | | | |
| 243. Girotto G, Pirastu N, Sorice R, Biino G, Campbell H, d'Adamo AP, Hastie ND, Nutile T, Polasek O, Portas L, Rudan I, Ulivi S, Zemunik T, et al. Hearing function and thresholds: a genome-wide association study in European isolated populations identifies new loci and pathways. J Med Genet. 2011; 48:369-74. | | | | | | | | | |
| 244. Luo M, Fan J, Wenger TL, Harr MH, Racobaldo M, Mulchandani S, Dubbs H, Zackai EH, Spinner NB, Conlin LK. CMIP haploinsufficiency in two patients with autism spectrum disorder and co-occurring gastrointestinal issues. Am J Med Genet A. 2017; 173:2101-7. | | | | | | | | | |
| 245. Tzeng HT, Su CC, Chang CP, Lai WW, Su WC, Wang YC. Rab37 in lung cancer mediates exocytosis of soluble ST2 and thus skews macrophages toward tumor-suppressing phenotype. Int J Cancer. 2018; 143:1753-63. | | | | | | | | | |
| 246. Ljubicic S, Bezzi P, Brajkovic S, Nesca V, Guay C, Ohbayashi N, Fukuda M, Abderrhamani A, Regazzi R. The GTPase Rab37 Participates in the Control of Insulin Exocytosis. PLoS One. 2013; 8:e68255. | | | | | | | | | |
| 247. Mori R, Ikematsu K, Kitaguchi T, Kim SE, Okamoto M, Chiba T, Miyawaki A, Shimokawa I, Tsuboi T. Release of TNF-α from macrophages is mediated by small GTPase Rab37. Eur J Immunol. 2011; 41:3230-9. | | | | | | | | | |
| 248. Higashio H, Satoh Y, Saino T. Mast cell degranulation is negatively regulated by the Munc13-4-binding small-guanosine triphosphatase Rab37. Sci Rep. 2016; 6:22539. | | | | | | | | | |
| 249. Sheng Y, Song Y, Li Z, Wang Y, Lin H, Cheng H, Zhou R. RAB37 interacts directly with ATG5 and promotes autophagosome formation via regulating ATG5-12-16 complex assembly. Cell Death Differ. 2018; 25:918-34. | | | | | | | | | |
| 250. Sundberg TB, Darricarrere N, Cirone P, Li X, McDonald L, Mei X, Westlake CJ, Slusarski DC, Beynon RJ, Crews CM. Disruption of Wnt planar cell polarity signaling by aberrant accumulation of the MetAP-2 substrate Rab37. Chem Biol. 2011; 18:1300-11. | | | | | | | | | |
| 251. Dobashi S, Katagiri T, Hirota E, Ashida S, Daigo Y, Shuin T, Fujioka T, Miki T, Nakamura Y. Involvement of TMEM22 overexpression in the growth of renal cell carcinoma cells. Oncol Rep. 2009; 21:305-12. | | | | | | | | | |
| 252. Wu CY, Tseng RC, Hsu HS, Wang YC, Hsu MT. Frequent down-regulation of hRAB37 in metastatic tumor by genetic and epigenetic mechanisms in lung cancer. Lung Cancer. 2009; 63:360-7. | | | | | | | | | |
| 253. Tsai CH, Cheng HC, Wang YS, Lin P, Jen J, Kuo IY, Chang YH, Liao PC, Chen RH, Yuan WC, Hsu HS, Yang MH, Hsu MT, et al. Small GTPase Rab37 targets tissue inhibitor of metalloproteinase 1 for exocytosis and thus suppresses tumour metastasis. Nat Commun. 2014; 5:4804. | | | | | | | | | |
| 254. Li Y, Yang X, Du X, Lei Y, He Q, Hong X, Tang X, Wen X, Zhang P, Sun Y, Zhang J, Wang Y, Ma J, Liu N. RAB37 Hypermethylation Regulates Metastasis and Resistance to Docetaxel-Based Induction Chemotherapy in Nasopharyngeal Carcinoma. Clin Cancer Res. 2018; 24:6495-508. | | | | | | | | | |
| 255. Tzeng HT, Tsai CH, Yen YT, Cheng HC, Chen YC, Pu SW, Wang YS, Shan YS, Tseng YL, Su WC, Lai WW, Wu LW, Wang YC. Dysregulation of Rab37-Mediated Cross-talk between Cancer Cells and Endothelial Cells via Thrombospondin-1 Promotes Tumor Neovasculature and Metastasis. Clin Cancer Res. 2017; 23:2335-45. | | | | | | | | | |
| 256. Wang S, Hashemi T, Fried S, Clemmons AL, Hawes BE. Differential intracellular signaling of the GalR1 and GalR2 galanin receptor subtypes. Biochemistry. 1998; 37:6711-7. | | | | | | | | | |
| 257. He L, Li Z, Zhou D, Ding Y, Xu L, Chen Y, Fan J. Galanin receptor 2 mediates antifibrogenic effects of galanin on hepatic stellate cells. Exp Ther Med. 2016; 12:3375-80. | | | | | | | | | |
| 258. Elliott-Hunt CR, Pope RJ, Vanderplank P, Wynick D. Activation of the galanin receptor 2 (GalR2) protects the hippocampus from neuronal damage. J Neurochem. 2007; 100:780-9. | | | | | | | | | |
| 259. Mazarati A, Lu X, Kilk K, Langel U, Wasterlain C, Bartfai T. Galanin type 2 receptors regulate neuronal survival, susceptibility to seizures and seizure-induced neurogenesis in the dentate gyrus. Eur J Neurosci. 2004; 19:3235-44. | | | | | | | | | |
| 260. Keimpema E, Zheng K, Barde SS, Berghuis P, Dobszay MB, Schnell R, Mulder J, Luiten PG, Xu ZD, Runesson J, Langel Ü, Lu B, Hökfelt T, et al. GABAergic terminals are a source of galanin to modulate cholinergic neuron development in the neonatal forebrain. Cereb Cortex. 2014; 24:3277-88. | | | | | | | | | |
| 261. Badie-Mahdavi H, Lu X, Behrens MM, Bartfai T. Role of galanin receptor 1 and galanin receptor 2 activation in synaptic plasticity associated with 3',5'-cyclic AMP response element-binding protein phosphorylation in the dentate gyrus: studies with a galanin receptor 2 agonist and galanin receptor 1 knockout mice. Neuroscience. 2005; 133:591-604. | | | | | | | | | |
| 262. Narváez M, Borroto-Escuela DO, Santín L, Millón C, Gago B, Flores-Burgess A, Barbancho MA, Pérez de la Mora M, Narváez J, Díaz-Cabiale Z, Fuxe K. A Novel Integrative Mechanism in Anxiolytic Behavior Induced by Galanin 2/Neuropeptide Y Y1 Receptor Interactions on Medial Paracapsular Intercalated Amygdala in Rats. Front Cell Neurosci. 2018; 12:119. | | | | | | | | | |
| 263. Narváez M, Millón C, Borroto-Escuela D, Flores-Burgess A, Santín L, Parrado C, Gago B, Puigcerver A, Fuxe K, Narváez JA, Díaz-Cabiale Z. Galanin receptor 2-neuropeptide Y Y1 receptor interactions in the amygdala lead to increased anxiolytic actions. Brain Struct Funct. 2015; 220:2289-301. | | | | | | | | | |
| 264. Metcalf CS, Klein BD, McDougle DR, Zhang L, Smith MD, Bulaj G, White HS. Analgesic properties of a peripherally acting and GalR2 receptor-preferring galanin analog in inflammatory, neuropathic, and acute pain models. J Pharmacol Exp Ther. 2015; 352:185-93. | | | | | | | | | |
| 265. Chen SH, Lue JH, Hsiao YJ, Lai SM, Wang HY, Lin CT, Chen YC, Tsai YJ. Elevated galanin receptor type 2 primarily contributes to mechanical hypersensitivity after median nerve injury. PLoS One. 2018; 13:e0199512. | | | | | | | | | |
| 266. Einstein EB, Asaka Y, Yeckel MF, Higley MJ, Picciotto MR. Galanin-induced decreases in nucleus accumbens/striatum excitatory postsynaptic potentials and morphine conditioned place preference require both galanin receptor 1 and galanin receptor 2. Eur J Neurosci. 2013; 37:1541-9. | | | | | | | | | |
| 267. Millón C, Flores-Burgess A, Castilla-Ortega E, Gago B, García-Fernandez M, Serrano A, Rodriguez de Fonseca F, Narváez JA, Fuxe K, Santín L, Díaz-Cabiale Z. Central administration of galanin N-terminal fragment 1-15 decreases the voluntary alcohol intake in rats. Addict Biol. 2019; 24:76-87. | | | | | | | | | |
| 268. Garcia-Rosa S, Trivella DB, Marques VD, Serafim RB, Pereira JG, Lorenzi JC, Molfetta GA, Christo PP, Olival GS, Marchitto VB, Brum DG, Sabedot TS, Noushmehr H, et al. A non-functional galanin receptor-2 in a multiple sclerosis patient. Pharmacogenomics J. 2019; 19:72-82. | | | | | | | | | |
| 269. Fang P, Zhang L, Yu M, Sheng Z, Shi M, Zhu Y, Zhang Z, Bo P. Activiated galanin receptor 2 attenuates insulin resistance in skeletal muscle of obese mice. Peptides. 2018; 99:92-8. | | | | | | | | | |
| 270. Fang P, He B, Yu M, Shi M, Zhu Y, Zhang Z, Bo P. Central galanin receptor 2 mediates galanin action to promote systemic glucose metabolism of type 2 diabetic rats. Biochem Pharmacol. 2018; 156:241-7. | | | | | | | | | |
| 271. Kolodziejski PA, Pruszynska-Oszmalek E, Micker M, Skrzypski M, Wojciechowicz T, Szwarckopf P, Skieresz-Szewczyk K, Nowak KW, Strowski MZ. Spexin: A novel regulator of adipogenesis and fat tissue metabolism. Biochim Biophys Acta Mol Cell Biol Lipids. 2018; 1863:1228-36. | | | | | | | | | |
| 272. Lin CY, Zhang M, Huang T, Yang LL, Fu HB, Zhao L, Zhong LL, Mu HX, Shi XK, Leung CF, Fan BM, Jiang M, Lu AP, et al. Spexin Enhances Bowel Movement through Activating L-type Voltage-dependent Calcium Channel via Galanin Receptor 2 in Mice. Sci Rep. 2015; 5:12095. | | | | | | | | | |
| 273. Lin CY, Zhao L, Huang T, Lu L, Khan M, Liu J, Zhong LLD, Cai ZW, Fan BM, Wong AOL, Bian ZX. Spexin Acts as Novel Regulator for Bile Acid Synthesis. Front Physiol. 2018; 9:378. | | | | | | | | | |
| 274. Kanazawa T, Misawa K, Misawa Y, Maruta M, Uehara T, Kawada K, Nagatomo T, Ichimura K. Galanin receptor 2 utilizes distinct signaling pathways to suppress cell proliferation and induce apoptosis in HNSCC. Mol Med Rep. 2014; 10:1289-94. | | | | | | | | | |
| 275. Tofighi R, Joseph B, Xia S, Xu ZQ, Hamberger B, Hökfelt T, Ceccatelli S. Galanin decreases proliferation of PC12 cells and induces apoptosis via its subtype 2 receptor (GalR2). Proc Natl Acad Sci U S A. 2008; 105:2717-22. | | | | | | | | | |
| 276. Misawa Y, Misawa K, Kanazawa T, Uehara T, Endo S, Mochizuki D, Yamatodani T, Carey TE, Mineta H. Tumor suppressor activity and inactivation of galanin receptor type 2 by aberrant promoter methylation in head and neck cancer. Cancer. 2014; 120:205-13. | | | | | | | | | |
| 277. Scanlon CS, Banerjee R, Inglehart RC, Liu M, Russo N, Hariharan A, van Tubergen EA, Corson SL, Asangani IA, Mistretta CM, Chinnaiyan AM, D'Silva NJ. Galanin modulates the neural niche to favour perineural invasion in head and neck cancer. Nat Commun. 2015; 6:6885. | | | | | | | | | |
| 278. Banerjee R, Van Tubergen EA, Scanlon CS, Vander Broek R, Lints JP, Liu M, Russo N, Inglehart RC, Wang Y, Polverini PJ, Kirkwood KL, D'Silva NJ. The G protein-coupled receptor GALR2 promotes angiogenesis in head and neck cancer. Mol Cancer Ther. 2014; 13:1323-33. | | | | | | | | | |
| 279. Shemesh A, Wang Y, Yang Y, Yang GS, Johnson DE, Backer JM, Pessin JE, Zong H. Suppression of mTORC1 activation in acid-α-glucosidase-deficient cells and mice is ameliorated by leucine supplementation. Am J Physiol Regul Integr Comp Physiol. 2014; 307:R1251-9. | | | | | | | | | |
| 280. Zhang J, Ma L, Huang J, Wei G, Liu L, Yan B. Altered expression of lysosomal hydrolase, acid α-glucosidase, gene in coronary artery disease. Coron Artery Dis. 2016; 27:104-8. | | | | | | | | | |
| 281. Hermans MM, van Leenen D, Kroos MA, Beesley CE, Van Der Ploeg AT, Sakuraba H, Wevers R, Kleijer W, Michelakakis H, Kirk EP, Fletcher J, Bosshard N, Basel-Vanagaite L, et al. Twenty-two novel mutations in the lysosomal alpha-glucosidase gene (GAA) underscore the genotype-phenotype correlation in glycogen storage disease type II. Hum Mutat. 2004; 23:47-56. | | | | | | | | | |
| 282. Qin Z, Ren F, Xu X, Ren Y, Li H, Wang Y, Zhai Y, Chang Z. ZNF536, a novel zinc finger protein specifically expressed in the brain, negatively regulates neuron differentiation by repressing retinoic acid-induced gene transcription. Mol Cell Biol. 2009; 29:3633-43. | | | | | | | | | |
| 283. Thyme SB, Pieper LM, Li EH, Pandey S, Wang Y, Morris NS, Sha C, Choi JW, Herrera KJ, Soucy ER, Zimmerman S, Randlett O, Greenwood J, et al. Phenotypic Landscape of Schizophrenia-Associated Genes Defines Candidates and Their Shared Functions. Cell. 2019; 177:478-91.e20. | | | | | | | | | |
| 284. Winham SJ, Cuellar-Barboza AB, McElroy SL, Oliveros A, Crow S, Colby CL, Choi DS, Chauhan M, Frye MA, Biernacka JM. Bipolar disorder with comorbid binge eating history: a genome-wide association study implicates APOB. J Affect Disord. 2014; 165:151-8. | | | | | | | | | |
| 285. Lin E, Kuo PH, Liu YL, Yu YW, Yang AC, Tsai SJ. A Deep Learning Approach for Predicting Antidepressant Response in Major Depression Using Clinical and Genetic Biomarkers. Front Psychiatry. 2018; 9:290. | | | | | | | | | |
| 286. Somaiah N, Beird HC, Barbo A, Song J, Mills Shaw KR, Wang WL, Eterovic K, Chen K, Lazar A, Conley AP, Ravi V, Hwu P, Futreal A, et al. Targeted next generation sequencing of well-differentiated/dedifferentiated liposarcoma reveals novel gene amplifications and mutations. Oncotarget. 2018; 9:19891-9. | | | | | | | | | |
| 287. Che W, Abe J, Yoshizumi M, Huang Q, Glassman M, Ohta S, Melaragno MG, Poppa V, Yan C, Lerner-Marmarosh N, Zhang C, Wu Y, Arlinghaus R, et al. p160 Bcr mediates platelet-derived growth factor activation of extracellular signal-regulated kinase in vascular smooth muscle cells. Circulation. 2001; 104:1399-406. | | | | | | | | | |
| 288. Korus M, Mahon GM, Cheng L, Whitehead IP. p38 MAPK-mediated activation of NF-kappaB by the RhoGEF domain of Bcr. Oncogene. 2002; 21:4601-12. | | | | | | | | | |
| 289. Voncken JW, van Schaick H, Kaartinen V, Deemer K, Coates T, Landing B, Pattengale P, Dorseuil O, Bokoch GM, Groffen J. Increased neutrophil respiratory burst in bcr-null mutants. Cell. 1995; 80:719-28. | | | | | | | | | |
| 290. Dubash AD, Koetsier JL, Amargo EV, Najor NA, Harmon RM, Green KJ. The GEF Bcr activates RhoA/MAL signaling to promote keratinocyte differentiation via desmoglein-1. J Cell Biol. 2013; 202:653-66. | | | | | | | | | |
| 291. Lee WR, Sacharidou A, Behling-Kelly E, Oltmann SC, Zhu W, Ahmed M, Gerard RD, Hui DY, Abe J, Shaul PW, Mineo C. PDZK1 prevents neointima formation via suppression of breakpoint cluster region kinase in vascular smooth muscle. PLoS One. 2015; 10:e0124494. | | | | | | | | | |
| 292. Alexis JD, Wang N, Che W, Lerner-Marmarosh N, Sahni A, Korshunov VA, Zou Y, Ding B, Yan C, Berk BC, Abe J. Bcr kinase activation by angiotensin II inhibits peroxisome-proliferator-activated receptor gamma transcriptional activity in vascular smooth muscle cells. Circ Res. 2009; 104:69-78. | | | | | | | | | |
| 293. Ramalingam D, Happel C, Ziegelbauer JM. Kaposi's sarcoma-associated herpesvirus microRNAs repress breakpoint cluster region protein expression, enhance Rac1 activity, and increase in vitro angiogenesis. J Virol. 2015; 89:4249-61. | | | | | | | | | |
| 294. Yang XY, Stanley RE, Ross AP, Robitaille AM, Gray JA, Cheyette BNR. Sestd1 Encodes a Developmentally Dynamic Synapse Protein That Complexes With BCR Rac1-GAP to Regulate Forebrain Dendrite, Spine and Synapse Formation. Cereb Cortex. 2019; 29:1217. | | | | | | | | | |
| 295. Um K, Niu S, Duman JG, Cheng JX, Tu YK, Schwechter B, Liu F, Hiles L, Narayanan AS, Ash RT, Mulherkar S, Alpadi K, Smirnakis SM, et al. Dynamic control of excitatory synapse development by a Rac1 GEF/GAP regulatory complex. Dev Cell. 2014; 29:701-15. | | | | | | | | | |
| 296. Laurent CE, Smithgall TE. The c-Fes tyrosine kinase cooperates with the breakpoint cluster region protein (Bcr) to induce neurite extension in a Rac- and Cdc42-dependent manner. Exp Cell Res. 2004; 299:188-98. | | | | | | | | | |
| 297. Park AR, Oh D, Lim SH, Choi J, Moon J, Yu DY, Park SG, Heisterkamp N, Kim E, Myung PK, Lee JR Regulation of dendritic arborization by BCR Rac1 GTPase-activating protein, a substrate of PTPRT. J Cell Sci. 2012; 125:4518-31. | | | | | | | | | |
| 298. Narayanan AS, Reyes SB, Um K, McCarty JH, Tolias KF. The Rac-GAP Bcr is a novel regulator of the Par complex that controls cell polarity. Mol Biol Cell. 2013; 24:3857-68. | | | | | | | | | |
| 299. Oh D, Han S, Seo J, Lee JR, Choi J, Groffen J, Kim K, Cho YS, Choi HS, Shin H, Woo J, Won H, Park SK, et al. Regulation of synaptic Rac1 activity, long-term potentiation maintenance, and learning and memory by BCR and ABR Rac GTPase-activating proteins. J Neurosci. 2010; 30:14134-44. | | | | | | | | | |
| 300. Hashimoto R, Okada T, Kato T, Kosuga A, Tatsumi M, Kamijima K, Kunugi H. The breakpoint cluster region gene on chromosome 22q11 is associated with bipolar disorder. Biol Psychiatry. 2005; 57:1097-102. | | | | | | | | | |
| 301. Masui T, Hashimoto R, Kusumi I, Suzuki K, Tanaka T, Nakagawa S, Suzuki T, Iwata N, Ozaki N, Kato T, Takeda M, Kunugi H, Koyama T. A possible association between missense polymorphism of the breakpoint cluster region gene and lithium prophylaxis in bipolar disorder. Prog Neuropsychopharmacol Biol Psychiatry. 2008; 32:204-8. | | | | | | | | | |

**Supplementary Table 5. Associations of monocyte-specific methylation sites with metabolic traits and hyperuricemia.**

|  | P_LDL_ | P_TG_ | P_HbA1c_ | Specific to gout |
| --- | --- | --- | --- | --- |
| **Surviving monocyte-specific methylation sites regulating which regulates interleukin-1β** | | | | |
| cg22626579 | 7.97×10^-1^ | 2.99×10^-1^ | 4.96×10^-1^ | Y |
| cg10314750 | 7.35×10^-2^ | 2.71×10^-1^ | 9.07×10^-1^ | Y |
| cg03795507 | 5.83×10^-1^ | 2.33×10^-1^ | 5.98×10^-1^ | Y |
| cg10257063 | 9.07×10^-1^ | 5.42×10^-1^ | 4.83×10^-1^ | Y |
| cg16975613 | 1.03×10^-1^ | 5.44×10^-2^ | 5.12×10-^2^ | Y |
| cg16630982 | 8.32×10^-1^ | 4.58×10^-1^ | 7.01×10^-1^ | Y |
| cg12182452 | 3.70×10^-1^ | 6.53×10^-1^ | 6.48×10^-1^ | Y |
| cg17151991 | 6.65×10^-1^ | 3.71×10^-1^ | 5.76×10^-1^ | Y |
| cg26375855 | 9.68×10^-2^ | 5.73×10^-1^ | 7.11×10^-1^ | Y |
| **Failed monocyte-specific methylation sites regulating which regulates interleukin-1β** | | | | |
| cg18886702 | 3.55×10^-2^ | 1.26×10^-1^ | 1.39×10^-2^ | N |
| cg00091098 | 4.98×10^-3^ | 2.86×10^-1^ | 3.52×10^-1^ | N |
| cg22408430 | 6.92×10^-2^ | 5.39×10^-1^ | 1.41×10^-2^ | N |
| cg13204333 | 9.40×10^-3^ | 1.20×10^-1^ | 9.11×10^-2^ | N |
| cg14326053 | 1.16×10^-2^ | 5.45×10^-2^ | 2.36×10^-1^ | N |
| cg10027934 | 1.91×10^-3^ | 2.30×10^-1^ | 9.22×10^-1^ | N |
| **Monocyte-specific methylation sites located in genes not regulating interleukin-1β** | | | | |
| cg15158067 | 9.62×10^-1^ | 4.15×10^-1^ | 6.74×10^-1^ | Y |
| cg01718853 | 5.53×10^-3^ | 5.63×10^-1^ | 7.30×10^-1^ | N |
| cg03789579 | 3.60×10^-1^ | 2.42×10^-1^ | 9.19×10^-1^ | Y |
| cg13559233 | 3.29×10^-2^ | 9.59×10^-1^ | 1.90×10^-1^ | N |
| cg05638359 | 9.27×10^-1^ | 2.83×10^-1^ | 7.85×10^-1^ | Y |
| cg03945122 | 2.77×10^-1^ | 9.99×10^-1^ | 4.07×10^-1^ | Y |
| cg26461510 | 9.49×10^-1^ | 3.70×10^-1^ | 1.34×10^-1^ | Y |
| cg03275949 | 2.57×10^-1^ | 2.96×10^-2^ | 3.15×10^-2^ | N |
| cg07083023 | 1.85×10^-1^ | 4.99×10-1 | 5.02×10^-1^ | Y |
| cg19824059 | 1.82×10^-1^ | 2.92×10^-1^ | 4.23×10^-1^ | Y |
| cg08797047 | 1.46×10^-1^ | 3.12×10^-1^ | 2.22×10^-1^ | Y |
| cg02993437 | 1.29×10^-1^ | 2.30×10^-1^ | 4.77×10^-1^ | Y |
| cg01080600 | 4.23×10^-1^ | 5.17×10^-1^ | 4.98×10^-1^ | Y |
| cg04258358 | 4.80×10^-1^ | 8.17×10^-1^ | 2.01×10^-1^ | Y |
| cg22517735 | 1.19×10^-1^ | 5.12×10^-1^ | 8.68×10^-1^ | Y |
| cg19459207 | 3.23×10^-1^ | 4.53×10^-1^ | 8.47×10^-1^ | Y |
| cg19868864 | 6.85×10^-2^ | 7.82×10^-1^ | 7.27×10^-1^ | Y |
| cg26993940 | 5.52×10^-1^ | 5.87×10^-1^ | 6.19×10^-2^ | Y |
| cg07235456 | 1.52×10^-2^ | 7.92×10^-1^ | 6.18×10^-1^ | N |
| cg24070123 | 6.05×10^-1^ | 1.89×10^-1^ | 3.06×10^-4^ | Y |
| cg27478167 | 8.09×10^-1^ | 6.69×10^-1^ | 9.04×10^-1^ | Y |
| cg05096788 | 8.73×10^-1^ | 7.26×10^-1^ | 9.69×10^-1^ | Y |
| cg11768833 | 5.61×10^-1^ | 8.64×10^-1^ | 8.26×10^-1^ | Y |
| cg09762316 | 2.76×10^-1^ | 9.89×10^-1^ | 7.92×10^-1^ | Y |
| cg00748492 | 5.84×10^-1^ | 7.47×10^-1^ | 2.96×10^-1^ | Y |
| cg25692425 | 1.68×10^-3^ | 6.61×10^-1^ | 3.95×10^-1^ | N |
| cg08234376 | 1.81×10^-1^ | 7.10×10^-1^ | 7.64×10^-1^ | Y |
| cg19411943 | 1.90×10^-1^ | 5.72×10^-1^ | 5.51×10^-1^ | Y |
| cg19469189 | 9.14×10^-1^ | 7.61×10^-1^ | 6.76×10^-2^ | Y |
| cg18961101 | 2.85×10^-1^ | 5.61×10^-1^ | 9.83×10^-1^ | Y |
| cg19532939 | 6.61×10^-2^ | 1.51×10^-1^ | 1.23×10^-1^ | Y |
| cg02325300 | 5.26×10^-1^ | 1.18×10^-1^ | 6.77×10^-2^ | Y |
| cg25983594 | 4.12×10^-1^ | 4.68×10^-1^ | 4.32×10^-1^ | Y |
| cg07445547 | 5.79×10^-1^ | 7.03×10^-1^ | 3.80×10^-1^ | Y |
| cg22241833 | 4.05×10^-1^ | 3.54×10^-1^ | 2.46×10^-1^ | Y |
| cg11169286 | 3.09×10^-1^ | 1.79×10^-2^ | 3.91×10^-1^ | N |
| cg05438708 | 4.21×10^-2^ | 8.40×10^-1^ | 3.08×10^-1^ | N |
| cg11367590 | 1.63×10^-1^ | 9.16×10^-1^ | 8.71×10^-1^ | Y |
| cg05464572 | 5.02×10^-1^ | 4.21×10^-1^ | 2.00×10^-1^ | Y |
| cg14165663 | 6.18×10^-1^ | 3.82×10^-1^ | 8.65×10^-1^ | Y |
| cg26351916 | 7.46×10^-1^ | 8.83×10^-1^ | 6.49×10^-1^ | Y |
| cg04810466 | 3.70×10^-2^ | 2.40×10^-1^ | 5.56×10^-1^ | N |
| cg05532013 | 6.47×10^-1^ | 9.02×10^-1^ | 7.59×10^-1^ | Y |

P_LDL_: P value of associations with low density lipoprotein; P_TG_: P value of associations with triglyceride; P_HbA1c_: P value of associations with glycated hemoglobin (HbA_1c_); P_Hyperuricemia_: P value of associations with hyperuricemia. P values less than 0.05 are highlighsted with shded box.

**Supplementary Table 6. Methylation sites associated with body mass index in EWASdb.**

| cg00029233 | cg00077904 | cg00996053 | cg06683487 |
| --- | --- | --- | --- |
| cg06694446 | cg07505725 | cg08734931 | cg11514288 |
| cg12685731 | cg13506158 | cg20502501 |  |

**Supplementary Table 7. Loci associated with uric acid at genome-wide significance (P < 5×10^-8^).**

| **Genetic variant** | **Chr** | **Position^a^** | **Gene** | ***P^b^*** | **Reference** |
| --- | --- | --- | --- | --- | --- |
| *rs9728345* | **1** | 145597417 | *POLR3C* | 3.15×10^-8^ | [1] |
| *rs11587821* | 1 | 145599038 | *POLR3C* | 2.01×10^-8^ | [1] |
| *rs10752826* | 1 | 145602791 | *POLR3C* | 1.89×10^-8^ | [1] |
| *rs4970859* | 1 | 145605318 | *POLR3C* | 3.57×10^-8^ | [1] |
| *rs12402867* | 1 | 145636226 | *RNF115* | 1.96×10^-8^ | [1] |
| *rs12724816* | 1 | 145639324 | *RNF115* | 1.87×10^-8^ | [1] |
| *rs12405132* | 1 | 145644984 | *RNF115* | 3.01×10^-8^ | [1] |
| *rs2040086* | 1 | 145651876 | *RNF115* | 2.56×10^-8^ | [1] |
| *rs2318299* | 1 | 145662666 | *RNF115* | 1.56×10^-8^ | [1] |
| *rs11591191* | 1 | 145675931 | *RNF115* | 2.09×10^-8^ | [1] |
| *rs12750384* | 1 | 145681484 | *RNF115* | 1.36×10^-8^ | [1] |
| *rs17354678* | 1 | 145689271 | *RNF115* | 1.70×10^-9^ | [1] |
| *rs12123298* | 1 | 145690472 | *RNF115* | 1.55×10^-9^ | [1] |
| *rs17352469* | 1 | 145693383 | *3' of CD160* | 4.51×10^-9^ | [1] |
| *rs2231375* | 1 | 145696694 | *CD160* | 3.06×10^-9^ | [1] |
| *rs1023945* | 1 | 145703115 | *CD160* | 2.51×10^-8^ | [1] |
| *rs1471628* | 1 | 145707057 | *CD160* | 9.51×10^-9^ | [1] |
| *rs4970874* | 1 | 145709394 | *CD160* | 1.81×10^-10^ | [1] |
| *rs744877* | 1 | 145714376 | *CD160* | 6.82×10^-9^ | [1] |
| *rs9728526* | 1 | 145716763 | *5' of CD160* | 3.68×10^-8^ | [1] |
| *rs3753436* | 1 | 145718113 | *5' of CD160* | 2.36×10^-8^ | [1] |
| *rs10910845* | 1 | 145723120 | *5' of PDZK1* | 4.87×10^-25^ | [1] |
| *rs1967017* | 1 | 145723645 | *5' of PDZK1* | 4.00×10^-8^ | [2] |
| *rs1471633* | 1 | 145723739 | *5' of PDZK1* | 1.16×10^-29^ | [1] |
| *rs12129861* | 1 | 145725689 | *5' of PDZK1* | 2.68×10^-9^ | [3] |
| *rs900347* | 1 | 145726727 | *5' of PDZK1* | 2.14×10^-18^ | [1] |
| *rs1298954* | 1 | 145730160 | *PDZK1* | 7.16×10^-19^ | [1] |
| *rs9659930* | 1 | 145730701 | *PDZK1* | 7.32×10^-14^ | [1] |
| *rs882210* | 1 | 145732946 | *PDZK1* | 1.72×10^-8^ | [1] |
| *rs9728619* | 1 | 145736438 | *PDZK1* | 3.11×10^-21^ | [1] |
| *rs4971059* | 1 | 155148781 | *KRTCAP2* | 3.02×10^-9^ | [1] |
| *rs3814316* | 1 | 155149718 | *KRTCAP2* | 6.48×10^-11^ | [1] |
| *rs11264341* | 1 | 155151493 | *KRTCAP2* | 6.22×10^-19^ | [1] |
| *rs9426886* | 1 | 155151754 | *KRTCAP2* | 1.12×10^-14^ | [1] |
| *rs4971100* | 1 | 155155731 | *KRTCAP2* | 1.82×10^-14^ | [1] |
| *rs4072037* | 1 | 155162067 | *MUC1* | 3.91×10^-9^ | [1] |
| *rs2990245* | 1 | 155197462 | *3' of GBA* | 2.63×10^-8^ | [1] |
| *rs189129662* | 1 | 201047231 | *CACNA1S* | 2.00×10^-16^ | [4] |
| *rs12734001* | 1 | 202390914 | *PPP1R12B* | 2.29×10^-9^ | [5] |
| *rs1395* | 2 | 27424636 | *SLC5A6* | 2.92×10^-8^ | [1] |
| *rs13404327* | 2 | 27519153 | *TRIM54* | 1.35×10^-9^ | [1] |
| *rs13404446* | 2 | 27519254 | *TRIM54* | 1.36×10^-9^ | [1] |
| *rs4665963* | 2 | 27528692 | *TRIM54* | 1.21×10^-9^ | [1] |
| *rs4665965* | 2 | 27536380 | *MPV17* | 9.18×10^-10^ | [1] |
| *rs1049817* | 2 | 27550967 | *GTF3C2* | 1.05×10^-16^ | [1] |
| *rs3739095* | 2 | 27556721 | *GTF3C2* | 4.54×10^-21^ | [1] |
| *rs11684134* | 2 | 27558252 | *GTF3C2* | 5.51×10^-18^ | [1] |
| *rs11689803* | 2 | 27566520 | *GTF3C2* | 4.87×10^-13^ | [1] |
| *rs6743819* | 2 | 27567407 | *GTF3C2* | 5.50×10^-17^ | [1] |
| *rs10205219* | 2 | 27568565 | *GTF3C2* | 5.45×10^-17^ | [1] |
| *rs4665969* | 2 | 27574953 | *GTF3C2* | 1.15×10^-16^ | [1] |
| *rs6760828* | 2 | 27579231 | *AC074117.10* | 9.10×10^-17^ | [1] |
| *rs7586601* | 2 | 27584666 | *AC074117.10* | 7.52×10^-20^ | [1] |
| *rs2280737* | 2 | 27589810 | *AC074117.10* | 4.31×10^-16^ | [1] |
| *rs7602534* | 2 | 27592423 | *EIF2B4* | 4.16×10^-16^ | [1] |
| *rs1528533* | 2 | 27595756 | *SNX17* | 1.58×10^-15^ | [1] |
| *rs13472* | 2 | 27600239 | *ZNF513* | 5.06×10^-16^ | [1] |
| *rs4582* | 2 | 27604279 | *PPM1G* | 6.64×10^-13^ | [1] |
| *rs2384629* | 2 | 27606098 | *PPM1G* | 3.74×10^-9^ | [1] |
| *rs1647284* | 2 | 27608115 | *PPM1G* | 1.54×10^-15^ | [1] |
| *rs7594812* | 2 | 27611469 | *PPM1G* | 1.36×10^-15^ | [1] |
| *rs12476704* | 2 | 27613031 | *PPM1G* | 5.67×10^-16^ | [1] |
| *rs2911712* | 2 | 27626945 | *PPM1G* | 1.62×10^-15^ | [1] |
| *rs7563162* | 2 | 27631191 | *PPM1G* | 2.30×10^-15^ | [1] |
| *rs1728918* | 2 | 27635463 | *5' of PPM1G* | 6.70×10^-9^ | [6] |
| *rs1060525* | 2 | 27635582 | *5' of PPM1G* | 1.70×10^-15^ | [1] |
| *rs2010087* | 2 | 27637235 | *5' of PPM1G* | 5.71×10^-16^ | [1] |
| *rs4665976* | 2 | 27640325 | *5' of PPM1G* | 1.58×10^-15^ | [1] |
| *rs11675428* | 2 | 27642734 | *5' of NRBP1* | 1.94×10^-8^ | [1] |
| *rs1728922* | 2 | 27644464 | *5' of NRBP1* | 1.99×10^-15^ | [1] |
| *rs6547626* | 2 | 27646770 | *5' of NRBP1* | 1.92×10^-15^ | [1] |
| *rs4665978* | 2 | 27648726 | *5' of NRBP1* | 1.16×10^-19^ | [1] |
| *rs780100* | 2 | 27652153 | *NRBP1* | 1.84×10^-15^ | [1] |
| *rs704791* | 2 | 27657167 | *NRBP1* | 2.16×10^-15^ | [1] |
| *rs780102* | 2 | 27659491 | *NRBP1* | 2.24×10^-15^ | [1] |
| *rs1260341* | 2 | 27663215 | *NRBP1* | 1.88×10^-15^ | [1] |
| *rs1260342* | 2 | 27663416 | *NRBP1* | 1.95×10^-15^ | [1] |
| *rs4803* | 2 | 27667297 | *KRTCAP3* | 2.03×10^-15^ | [1] |
| *rs780104* | 2 | 27677691 | *IFT172* | 2.24×10^-15^ | [1] |
| *rs780106* | 2 | 27681598 | *IFT172* | 1.17×10^-15^ | [1] |
| *rs780107* | 2 | 27684734 | *IFT172* | 1.25×10^-15^ | [1] |
| *rs780110* | 2 | 27685388 | *IFT172* | 9.11×10^-20^ | [1] |
| *rs1647276* | 2 | 27688601 | *IFT172* | 1.39×10^-15^ | [1] |
| *rs1647266* | 2 | 27693485 | *IFT172* | 1.41×10^-15^ | [1] |
| *rs780117* | 2 | 27698343 | *IFT172* | 1.16×10^-15^ | [1] |
| *rs1260345* | 2 | 27703495 | *IFT172* | 7.03×10^-13^ | [1] |
| *rs2272417* | 2 | 27706640 | *IFT172* | 3.39×10^-13^ | [1] |
| *rs8395* | 2 | 27715207 | *FNDC4* | 7.73×10^-13^ | [1] |
| *rs2303369* | 2 | 27715416 | *FNDC4* | 5.17×10^-16^ | [1] |
| *rs704795* | 2 | 27716494 | *FNDC4* | 1.50×10^-15^ | [1] |
| *rs780090* | 2 | 27718474 | *5' of FNDC4* | 6.00×10^-16^ | [1] |
| *rs813592* | 2 | 27721971 | *GCKR* | 2.70×10^-15^ | [1] |
| *rs1260320* | 2 | 27722416 | *GCKR* | 1.53×10^-15^ | [1] |
| *rs2293572* | 2 | 27728777 | *GCKR* | 4.94×10^-16^ | [1] |
| *rs2293571* | 2 | 27729480 | *GCKR* | 6.40×10^-16^ | [1] |
| *rs1260326* | 2 | 27730940 | *GCKR* | 1.25×10^-44^ | [1] |
| *rs3817588* | 2 | 27731212 | *GCKR* | 1.70×10^-10^ | [1] |
| *rs4425043* | 2 | 27733452 | *GCKR* | 8.55×10^-16^ | [1] |
| *rs780094* | 2 | 27741237 | *GCKR* | 6.52×10^-39^ | [1] |
| *rs780093* | 2 | 27742603 | *GCKR* | 3.80×10^-17^ | [7] |
| *rs780092* | 2 | 27743154 | *GCKR* | 8.46×10^-10^ | [1] |
| *rs814295* | 2 | 27743215 | *GCKR* | 3.23×10^-9^ | [1] |
| *rs11681351* | 2 | 27743423 | *GCKR* | 7.62×10^-15^ | [1] |
| *rs8179252* | 2 | 27746832 | *3' of GCKR* | 1.02×10^-14^ | [1] |
| *rs1260333* | 2 | 27748624 | *3' of GCKR* | 5.01×10^-33^ | [1] |
| *rs2911711* | 2 | 27750546 | *3' of GCKR* | 4.55×10^-33^ | [1] |
| *rs4665987* | 2 | 27755825 | *5' of AC109829.1* | 4.34×10^-13^ | [1] |
| *rs4665991* | 2 | 27766284 | *AC109829.1* | 7.11×10^-13^ | [1] |
| *rs4665382* | 2 | 27783801 | *AC109829.1* | 6.13×10^-13^ | [1] |
| *rs10208529* | 2 | 27786188 | *AC109829.1* | 6.28×10^-13^ | [1] |
| *rs4665383* | 2 | 27791555 | *3' of AC109829.1* | 6.35×10^-13^ | [1] |
| *rs1919127* | 2 | 27801493 | *C2orf16* | 7.33×10^-13^ | [1] |
| *rs1919128* | 2 | 27801759 | *C2orf16* | 5.85×10^-13^ | [1] |
| *rs12478841* | 2 | 27811722 | *ZNF512* | 6.85×10^-13^ | [1] |
| *rs6760250* | 2 | 27812252 | *ZNF512* | 6.73×10^-13^ | [1] |
| *rs13022873* | 2 | 27815510 | *ZNF512* | 6.22×10^-11^ | [1] |
| *rs12467476* | 2 | 27825715 | *ZNF512* | 5.52×10^-13^ | [1] |
| *rs2384656* | 2 | 27832055 | *ZNF512* | 7.07×10^-13^ | [1] |
| *rs4666000* | 2 | 27839369 | *ZNF512* | 4.17×10^-11^ | [1] |
| *rs2068834* | 2 | 27839539 | *ZNF512* | 4.28×10^-11^ | [1] |
| *rs4666002* | 2 | 27840640 | *ZNF512* | 3.52×10^-10^ | [1] |
| *rs3749147* | 2 | 27851918 | *ZNF512* | 1.62×10^-9^ | [1] |
| *rs13002853* | 2 | 27853245 | *ZNF512* | 8.91×10^-12^ | [1] |
| *rs2272406* | 2 | 27892023 | *SLC4A1AP* | 5.79×10^-9^ | [1] |
| *rs2178198* | 2 | 27895073 | *SLC4A1AP* | 1.58×10^-12^ | [1] |
| *rs13023094* | 2 | 27910706 | *SLC4A1AP* | 8.12×10^-9^ | [1] |
| *rs13030973* | 2 | 27928797 | *AC074091.13* | 1.62×10^-8^ | [1] |
| *rs6727388* | 2 | 27932587 | *AC074091.13* | 1.49×10^-8^ | [1] |
| *rs4616435* | 2 | 27933642 | *AC074091.13* | 1.48×10^-8^ | [1] |
| *rs6727215* | 2 | 27934731 | *AC074091.13* | 1.65×10^-8^ | [1] |
| *rs13023194* | 2 | 27967260 | *5' of MRPL33* | 1.89×10^-9^ | [1] |
| *rs12104449* | 2 | 27972833 | *5' of MRPL33* | 6.09×10^-13^ | [1] |
| *rs13030345* | 2 | 28003174 | *MRPL33* | 1.44×10^-8^ | [1] |
| *rs4401177* | 2 | 28344285 | *BRE* | 2.77×10^-9^ | [1] |
| *rs17709034* | 2 | 28424472 | *BRE* | 6.16×10^-9^ | [1] |
| *rs7349418* | 2 | 28443050 | *BRE* | 1.59×10^-8^ | [1] |
| *rs2311597* | 2 | 121305771 | *5' of AC073257.2* | 1.59×10^-8^ | [1] |
| *rs17050272* | 2 | 121306440 | *5' of AC073257.2* | 1.60×10^-10^ | [1] |
| *rs2030746* | 2 | 121309488 | *3' of AC073257.1* | 3.38×10^-8^ | [1] |
| *rs6706968* | 2 | 121310269 | *3' of AC073257.1* | 1.48×10^-8^ | [1] |
| *rs1424949* | 2 | 148542963 | *5' of ACVR2A* | 3.99×10^-8^ | [1] |
| *rs12479193* | 2 | 148557911 | *5' of ACVR2A* | 4.99×10^-8^ | [1] |
| *rs12472058* | 2 | 148558041 | *5' of ACVR2A* | 4.72×10^-8^ | [1] |
| *rs4972326* | 2 | 148558848 | *5' of ACVR2A* | 4.65×10^-8^ | [1] |
| *rs7605029* | 2 | 148559921 | *5' of ACVR2A* | 4.29×10^-8^ | [1] |
| *rs986508* | 2 | 148562031 | *5' of ACVR2A* | 3.17×10^-8^ | [1] |
| *rs13032660* | 2 | 148562909 | *5' of ACVR2A* | 3.59×10^-8^ | [1] |
| *rs1863153* | 2 | 148564193 | *5' of ACVR2A* | 3.60×10^-8^ | [1] |
| *rs1863152* | 2 | 148564405 | *5' of ACVR2A* | 3.79×10^-8^ | [1] |
| *rs6734998* | 2 | 148566321 | *5' of ACVR2A* | 4.18×10^-8^ | [1] |
| *rs1991169* | 2 | 148566919 | *5' of ACVR2A* | 3.70×10^-8^ | [1] |
| *rs12990959* | 2 | 148572160 | *5' of ACVR2A* | 3.71×10^-8^ | [1] |
| *rs11894371* | 2 | 148575872 | *5' of ACVR2A* | 2.43×10^-8^ | [1] |
| *rs1014064* | 2 | 148612154 | *ACVR2A* | 3.76×10^-8^ | [1] |
| *rs10803523* | 2 | 148615831 | *ACVR2A* | 2.74×10^-8^ | [1] |
| *rs2113793* | 2 | 148623453 | *ACVR2A* | 4.76×10^-8^ | [1] |
| *rs929939* | 2 | 148627528 | *ACVR2A* | 3.16×10^-8^ | [1] |
| *rs6711673* | 2 | 148643259 | *ACVR2A* | 3.10×10^-8^ | [1] |
| *rs13033696* | 2 | 148645327 | *ACVR2A* | 4.55×10^-8^ | [1] |
| *rs2161983* | 2 | 148649386 | *ACVR2A* | 2.95×10^-8^ | [1] |
| *rs13019809* | 2 | 148650473 | *ACVR2A* | 2.44×10^-8^ | [1] |
| *rs1128919* | 2 | 148657117 | *AC009480.3* | 3.68×10^-8^ | [1] |
| *rs13026650* | 2 | 148674201 | *ACVR2A* | 2.87×10^-8^ | [1] |
| *rs3764955* | 2 | 148674797 | *ACVR2A* | 3.15×10^-8^ | [1] |
| *rs1345994* | 2 | 148695126 | *ORC4* | 1.93×10^-8^ | [1] |
| *rs13012311* | 2 | 148707032 | *ORC4* | 1.54×10^-8^ | [1] |
| *rs13027200* | 2 | 148709653 | *ORC4* | 2.41×10^-8^ | [1] |
| *rs12463798* | 2 | 148716099 | *ORC4* | 1.45×10^-8^ | [1] |
| *rs2307394* | 2 | 148716428 | *ORC4L* | 7.26×10^-9^ | [1] |
| *rs6729465* | 2 | 148723126 | *ORC4* | 1.64×10^-8^ | [1] |
| *rs11901963* | 2 | 148726771 | *ORC4* | 1.34×10^-8^ | [1] |
| *rs13027706* | 2 | 148732703 | *ORC4* | 2.11×10^-8^ | [1] |
| *rs7594075* | 2 | 148739823 | *ORC4* | 1.85×10^-8^ | [1] |
| *rs13004041* | 2 | 148741621 | *ORC4* | 2.38×10^-8^ | [1] |
| *rs12463554* | 2 | 148751612 | *ORC4* | 3.33×10^-8^ | [1] |
| *rs13008838* | 2 | 148754825 | *ORC4* | 2.89×10^-8^ | [1] |
| *rs13035475* | 2 | 148754862 | *ORC4* | 1.72×10^-8^ | [1] |
| *rs13014936* | 2 | 148755237 | *ORC4* | 1.71×10^-8^ | [1] |
| *rs13022962* | 2 | 148756624 | *ORC4* | 1.46×10^-8^ | [1] |
| *rs12053401* | 2 | 148756668 | *ORC4* | 1.58×10^-8^ | [1] |
| *rs13028348* | 2 | 148757144 | *ORC4* | 1.51×10^-8^ | [1] |
| *rs1598207* | 2 | 148757870 | *ORC4* | 1.76×10^-8^ | [1] |
| *rs7598361* | 2 | 148758479 | *ORC4* | 3.22×10^-8^ | [1] |
| *rs2382201* | 2 | 148765496 | *ORC4* | 3.34×10^-8^ | [1] |
| *rs12989250* | 2 | 148776438 | *ORC4* | 3.19×10^-8^ | [1] |
| *rs1975748* | 2 | 148776859 | *ORC4* | 2.06×10^-8^ | [1] |
| *rs1015096* | 2 | 148782358 | *MBD5* | 2.64×10^-8^ | [1] |
| *rs13007770* | 2 | 148784324 | *MBD5* | 3.26×10^-8^ | [1] |
| *rs2890915* | 2 | 148789543 | *MBD5* | 3.88×10^-8^ | [1] |
| *rs144081819* | 2 | 170030639 | *LRP2* | 5.60×10^-10^ | [4] |
| *rs147287428* | 2 | 170063223 | *LRP2* | 2.00×10^-16^ | [4] |
| *rs145365776* | 2 | 170089947 | *LRP2* | 1.80×10^-9^ | [4] |
| *rs200469773* | 2 | 170093742 | *LRP2* | 2.00×10^-16^ | [4] |
| *rs145669628* | 2 | 170096097 | *LRP2* | 3.20×10^-8^ | [4] |
| *rs140061784* | 2 | 170100029 | *LRP2* | 2.00×10^-16^ | [4] |
| *rs2544390* | 2 | 170204846 | *LRP2* | 3.74×10^-8^ | [3] |
| *rs199592697* | 3 | 13896129 | *WNT7A* | 2.00×10^-16^ | [4] |
| *rs186459505* | 3 | 52941099 | *SFMBT1* | 2.00×10^-16^ | [4] |
| *rs2581824* | 3 | 53022408 | *SFMBT1* | 8.35×10^-16^ | [1] |
| *rs2564934* | 3 | 53024580 | *SFMBT1* | 2.67×10^-15^ | [1] |
| *rs2564938* | 3 | 53026384 | *SFMBT1* | 8.73×10^-11^ | [1] |
| *rs11708675* | 3 | 53031912 | *SFMBT1* | 9.24×10^-11^ | [1] |
| *rs2564919* | 3 | 53033295 | *SFMBT1* | 5.92×10^-11^ | [1] |
| *rs12635298* | 3 | 53033796 | *SFMBT1* | 7.54×10^-11^ | [1] |
| *rs2581792* | 3 | 53035044 | *SFMBT1* | 3.65×10^-8^ | [1] |
| *rs2564917* | 3 | 53037695 | *SFMBT1* | 2.92×10^-11^ | [1] |
| *rs2581795* | 3 | 53038786 | *SFMBT1* | 1.52×10^-11^ | [1] |
| *rs1529544* | 3 | 53039455 | *SFMBT1* | 2.01×10^-11^ | [1] |
| *rs17304694* | 3 | 53051577 | *SFMBT1* | 1.49×10^-8^ | [1] |
| *rs2581777* | 3 | 53054727 | *SFMBT1* | 7.35×10^-10^ | [1] |
| *rs2244552* | 3 | 53055522 | *SFMBT1* | 2.08×10^-15^ | [1] |
| *rs2244461* | 3 | 53055842 | *SFMBT1* | 1.09×10^-9^ | [1] |
| *rs9847710* | 3 | 53062661 | *SFMBT1* | 6.42×10^-16^ | [1] |
| *rs2581806* | 3 | 53063360 | *SFMBT1* | 8.52×10^-10^ | [1] |
| *rs2564956* | 3 | 53070462 | *SFMBT1* | 1.00×10^-15^ | [1] |
| *rs2581818* | 3 | 53071652 | *SFMBT1* | 4.24×10^-10^ | [1] |
| *rs6770152* | 3 | 53100214 | *3' of AC096887.1* | 2.62×10^-16^ | [1] |
| *rs2581790* | 3 | 53101780 | *3' of AC096887.1* | 3.29×10^-9^ | [1] |
| *rs202007714* | 3 | 66433522 | *SLC25A26* | 2.00×10^-16^ | [4] |
| *rs61746315* | 3 | 66433730 | *SLC25A26* | 2.00×10^-16^ | [4] |
| *rs201874364* | 3 | 185783611 | *ETV5* | 2.00×10^-16^ | [4] |
| *rs200263685* | 3 | 187451339 | *BCL6* | 2.00×10^-16^ | [4] |
| *rs13114077* | 4 | 9413105 | *5' of RN5S153* | 1.37×10^-12^ | [1] |
| *rs6811069* | 4 | 9415008 | *5' of RN5S153* | 6.91×10^-17^ | [1] |
| *rs1949921* | 4 | 9419322 | *5' of RN5S153* | 3.38×10^-17^ | [1] |
| *rs11722202* | 4 | 9443894 | *5' of DEFB131* | 6.85×10^-11^ | [1] |
| *rs4974886* | 4 | 9503349 | *3' of AC116655.1* | 1.84×10^-14^ | [1] |
| *rs12513044* | 4 | 9506678 | *3' of AC116655.1* | 2.52×10^-22^ | [1] |
| *rs11722359* | 4 | 9508214 | *3' of AC116655.1* | 1.20×10^-21^ | [1] |
| *rs11737509* | 4 | 9511324 | *3' of AC116655.1* | 1.84×10^-16^ | [1] |
| *rs6448742* | 4 | 9542759 | *3' of MIR548I2* | 2.83×10^-15^ | [1] |
| *rs12233771* | 4 | 9552965 | *3' of MIR548I2* | 1.25×10^-19^ | [1] |
| *rs10013288* | 4 | 9559218 | *5' of MIR548I2* | 5.18×10^-16^ | [1] |
| *rs2170252* | 4 | 9560062 | *5' of MIR548I2* | 4.38×10^-16^ | [1] |
| *rs13117722* | 4 | 9561639 | *5' of MIR548I2* | 2.26×10^-16^ | [1] |
| *rs2061995* | 4 | 9561739 | *5' of MIR548I2* | 4.35×10^-10^ | [1] |
| *rs13116764* | 4 | 9561759 | *5' of MIR548I2* | 5.93×10^-22^ | [1] |
| *rs13131681* | 4 | 9563930 | *5' of MIR548I2* | 3.27×10^-16^ | [1] |
| *rs6448816* | 4 | 9564484 | *5' of MIR548I2* | 9.14×10^-16^ | [1] |
| *rs12646317* | 4 | 9565838 | *5' of MIR548I2* | 2.21×10^-17^ | [1] |
| *rs11939517* | 4 | 9567260 | *5' of MIR548I2* | 6.62×10^-17^ | [1] |
| *rs4102942* | 4 | 9568478 | *5' of MIR548I2* | 7.82×10^-21^ | [1] |
| *rs1825043* | 4 | 9573740 | *5' of MIR548I2* | 6.67×10^-14^ | [1] |
| *rs7669441* | 4 | 9576926 | *5' of MIR548I2* | 7.82×10^-12^ | [1] |
| *rs9683876* | 4 | 9584058 | *3' of AC097493.1* | 3.09×10^-12^ | [1] |
| *rs13138961* | 4 | 9585147 | *3' of AC097493.1* | 4.81×10^-35^ | [1] |
| *rs12512447* | 4 | 9586313 | *3' of AC097493.1* | 2.32×10^-43^ | [1] |
| *rs10049659* | 4 | 9586764 | *3' of AC097493.1* | 8.28×10^-12^ | [1] |
| *rs1811570* | 4 | 9587999 | *3' of AC097493.1* | 6.94×10^-14^ | [1] |
| *rs6824806* | 4 | 9589427 | *3' of AC097493.1* | 1.86×10^-14^ | [1] |
| *rs6815602* | 4 | 9592093 | *3' of AC097493.1* | 1.11×10^-31^ | [1] |
| *rs16898588* | 4 | 9595751 | *3' of AC097493.1* | 1.51×10^-23^ | [1] |
| *rs6448858* | 4 | 9595918 | *3' of AC097493.1* | 1.40×10^-13^ | [1] |
| *rs4974823* | 4 | 9602719 | *5' of AC097493.1* | 1.06×10^-18^ | [1] |
| *rs12509677* | 4 | 9602724 | *5' of AC097493.1* | 7.39×10^-24^ | [1] |
| *rs13103207* | 4 | 9603672 | *5' of AC097493.1* | 2.88×10^-41^ | [1] |
| *rs2077679* | 4 | 9608089 | *5' of AC097493.1* | 1.09×10^-18^ | [1] |
| *rs4974853* | 4 | 9609115 | *5' of AC097493.1* | 1.61×10^-18^ | [1] |
| *rs7375281* | 4 | 9609506 | *5' of AC097493.1* | 1.36×10^-19^ | [1] |
| *rs11724183* | 4 | 9609894 | *5' of AC097493.1* | 9.23×10^-19^ | [1] |
| *rs4974812* | 4 | 9614598 | *5' of AC097493.1* | 5.96×10^-19^ | [1] |
| *rs11735475* | 4 | 9614926 | *5' of AC097493.1* | 2.00×10^-19^ | [1] |
| *rs6838199* | 4 | 9621333 | *5' of AC097493.1* | 1.92×10^-13^ | [1] |
| *rs6837106* | 4 | 9626304 | *5' of AC097493.1* | 2.29×10^-19^ | [1] |
| *rs10049735* | 4 | 9626409 | *5' of AC097493.1* | 1.10×10^-21^ | [1] |
| *rs10005684* | 4 | 9626897 | *5' of AC097493.1* | 2.87×10^-17^ | [1] |
| *rs9998663* | 4 | 9627053 | *5' of AC097493.1* | 3.46×10^-17^ | [1] |
| *rs13151183* | 4 | 9627066 | *5' of AC097493.1* | 1.33×10^-33^ | [1] |
| *rs13107086* | 4 | 9628088 | *5' of AC097493.1* | 1.65×10^-31^ | [1] |
| *rs10939427* | 4 | 9628538 | *5' of AC097493.1* | 7.61×10^-19^ | [1] |
| *rs7693695* | 4 | 9629380 | *5' of AC097493.1* | 1.75×10^-29^ | [1] |
| *rs11939895* | 4 | 9630402 | *5' of AC097493.1* | 2.22×10^-17^ | [1] |
| *rs9291589* | 4 | 9633410 | *5' of AC097493.1* | 5.24×10^-18^ | [1] |
| *rs6842855* | 4 | 9633990 | *5' of AC097493.1* | 1.35×10^-18^ | [1] |
| *rs11731624* | 4 | 9637608 | *5' of AC097493.1* | 4.31×10^-16^ | [1] |
| *rs11737243* | 4 | 9641272 | *5' of AC097493.1* | 6.64×10^-19^ | [1] |
| *rs11733687* | 4 | 9643374 | *5' of AC097493.1* | 2.02×10^-23^ | [1] |
| *rs10939436* | 4 | 9647135 | *5' of AC097493.1* | 6.38×10^-19^ | [1] |
| *rs7440232* | 4 | 9651744 | *5' of AC097493.1* | 5.38×10^-19^ | [1] |
| *rs7654169* | 4 | 9654071 | *5' of AC097493.1* | 5.27×10^-19^ | [1] |
| *rs6812811* | 4 | 9672000 | *5' of AC097493.1* | 1.31×10^-17^ | [1] |
| *rs7434744* | 4 | 9672688 | *5' of AC097493.1* | 1.10×10^-16^ | [1] |
| *rs10002984* | 4 | 9680733 | *5' of AC097493.1* | 1.58×10^-17^ | [1] |
| *rs6858093* | 4 | 9681650 | *5' of AC097493.1* | 6.04×10^-17^ | [1] |
| *rs13104360* | 4 | 9684804 | *5' of AC097493.1* | 2.41×10^-17^ | [1] |
| *rs7671092* | 4 | 9687441 | *3' of SLC2A9* | 1.79×10^-15^ | [1] |
| *rs7658414* | 4 | 9687969 | *3' of SLC2A9* | 4.12×10^-10^ | [1] |
| *rs7691759* | 4 | 9688014 | *3' of SLC2A9* | 1.14×10^-10^ | [1] |
| *rs6821253* | 4 | 9690731 | *3' of SLC2A9* | 6.20×10^-34^ | [1] |
| *rs10022012* | 4 | 9693182 | *3' of SLC2A9* | 7.79×10^-15^ | [1] |
| *rs7678732* | 4 | 9693253 | *3' of SLC2A9* | 1.54×10^-18^ | [1] |
| *rs12649073* | 4 | 9694490 | *3' of SLC2A9* | 1.66×10^-14^ | [1] |
| *rs13103452* | 4 | 9694662 | *3' of SLC2A9* | 4.90×10^-34^ | [1] |
| *rs4554078* | 4 | 9701544 | *3' of SLC2A9* | 2.53×10^-18^ | [1] |
| *rs10805313* | 4 | 9703061 | *3' of SLC2A9* | 5.20×10^-18^ | [1] |
| *rs9685887* | 4 | 9706342 | *3' of SLC2A9* | 8.31×10^-19^ | [1] |
| *rs9291607* | 4 | 9708798 | *3' of SLC2A9* | 3.09×10^-17^ | [1] |
| *rs6858393* | 4 | 9709577 | *3' of SLC2A9* | 5.89×10^-22^ | [1] |
| *rs13136075* | 4 | 9714968 | *3' of SLC2A9* | 5.12×10^-33^ | [1] |
| *rs6448974* | 4 | 9715105 | *3' of SLC2A9* | 3.12×10^-16^ | [1] |
| *rs9684176* | 4 | 9716386 | *3' of SLC2A9* | 4.59×10^-33^ | [1] |
| *rs12501880* | 4 | 9717975 | *3' of SLC2A9* | 3.07×10^-33^ | [1] |
| *rs11730320* | 4 | 9719901 | *3' of SLC2A9* | 5.07×10^-31^ | [1] |
| *rs11727674* | 4 | 9719981 | *3' of SLC2A9* | 1.80×10-17 | [1] |
| *rs11724606* | 4 | 9720444 | *3' of SLC2A9* | 1.19×10^-31^ | [1] |
| *rs7659176* | 4 | 9721066 | *3' of SLC2A9* | 1.65×10^-18^ | [1] |
| *rs10939472* | 4 | 9721616 | *3' of SLC2A9* | 2.49×10^-18^ | [1] |
| *rs12645989* | 4 | 9722133 | *3' of SLC2A9* | 2.55×10^-19^ | [1] |
| *rs13121465* | 4 | 9722275 | *3' of SLC2A9* | 4.81×10^-54^ | [1] |
| *rs13136217* | 4 | 9722292 | *3' of SLC2A9* | 1.44×10^-18^ | [1] |
| *rs13128435* | 4 | 9723672 | *3' of SLC2A9* | 1.87×10^-45^ | [1] |
| *rs12647851* | 4 | 9724519 | *3' of SLC2A9* | 1.38×10^-10^ | [1] |
| *rs2037313* | 4 | 9724711 | *3' of SLC2A9* | 2.10×10^-18^ | [1] |
| *rs7685513* | 4 | 9728599 | *3' of SLC2A9* | 1.95×10^-18^ | [1] |
| *rs13127001* | 4 | 9731215 | *3' of SLC2A9* | 2.45×10^-48^ | [1] |
| *rs6811848* | 4 | 9732161 | *3' of SLC2A9* | 8.97×10^-17^ | [1] |
| *rs7684214* | 4 | 9734585 | *3' of SLC2A9* | 2.00×10^-17^ | [1] |
| *rs12645163* | 4 | 9735520 | *3' of SLC2A9* | 1.59×10^-11^ | [1] |
| *rs7675599* | 4 | 9736516 | *3' of SLC2A9* | 3.08×10^-18^ | [1] |
| *rs7667385* | 4 | 9736696 | *3' of SLC2A9* | 4.15×10^-11^ | [1] |
| *rs6832128* | 4 | 9738151 | *3' of SLC2A9* | 4.48×10^-11^ | [1] |
| *rs6448981* | 4 | 9740325 | *3' of SLC2A9* | 1.29×10^-36^ | [1] |
| *rs1818670* | 4 | 9745390 | *3' of SLC2A9* | 7.65×10^-19^ | [1] |
| *rs12508809* | 4 | 9748034 | *3' of SLC2A9* | 5.68×10^-16^ | [1] |
| *rs6834270* | 4 | 9756710 | *3' of SLC2A9* | 7.31×10^-20^ | [1] |
| *rs6834512* | 4 | 9756836 | *3' of SLC2A9* | 4.08×10^-14^ | [1] |
| *rs6834697* | 4 | 9756914 | *3' of SLC2A9* | 2.12×10^-18^ | [1] |
| *rs11727873* | 4 | 9758469 | *3' of SLC2A9* | 9.14×10^-34^ | [1] |
| *rs10011621* | 4 | 9760239 | *3' of SLC2A9* | 6.10×10^-34^ | [1] |
| *rs13140817* | 4 | 9762975 | *3' of SLC2A9* | 8.87×10^-14^ | [8] |
| *rs11733815* | 4 | 9764282 | *3' of SLC2A9* | 4.42×10^-19^ | [1] |
| *rs11734974* | 4 | 9764564 | *3' of SLC2A9* | 5.76×10^-15^ | [1] |
| *rs11729600* | 4 | 9764686 | *3' of SLC2A9* | 4.05×10^-18^ | [1] |
| *rs7655090* | 4 | 9765875 | *3' of SLC2A9* | 2.83×10^-40^ | [1] |
| *rs11731100* | 4 | 9767180 | *3' of SLC2A9* | 4.44×10^-60^ | [1] |
| *rs10939504* | 4 | 9767370 | *3' of SLC2A9* | 4.42×10^-18^ | [1] |
| *rs11732272* | 4 | 9768258 | *3' of SLC2A9* | 5.76×10^-43^ | [1] |
| *rs10939507* | 4 | 9768823 | *3' of SLC2A9* | 5.92×10^-18^ | [1] |
| *rs10939514* | 4 | 9773196 | *SLC2A9* | 5.50×10^-18^ | [1] |
| *rs10939515* | 4 | 9773296 | *SLC2A9* | 3.39×10^-40^ | [1] |
| *rs10033951* | 4 | 9779580 | *SLC2A9* | 4.86×10^-40^ | [1] |
| *rs2867383* | 4 | 9787935 | *SLC2A9* | 4.96×10^-40^ | [1] |
| *rs1850744* | 4 | 9790712 | *SLC2A9* | 2.16×10^-17^ | [1] |
| *rs7685396* | 4 | 9794724 | *SLC2A9* | 8.76×10^-54^ | [1] |
| *rs1850739* | 4 | 9797343 | *SLC2A9* | 2.11×10^-17^ | [1] |
| *rs13106539* | 4 | 9797703 | *SLC2A9* | 6.10×10^-60^ | [1] |
| *rs6449000* | 4 | 9798612 | *SLC2A9* | 9.80×10^-17^ | [1] |
| *rs2280207* | 4 | 9799776 | *SLC2A9* | 7.34×10^-60^ | [1] |
| *rs2280208* | 4 | 9800043 | *SLC2A9* | 2.24×10^-17^ | [1] |
| *rs938556* | 4 | 9802366 | *SLC2A9* | 1.09×10^-16^ | [1] |
| *rs1519097* | 4 | 9802853 | *SLC2A9* | 1.48×10^-41^ | [1] |
| *rs1533615* | 4 | 9804524 | *SLC2A9* | 7.66×10^-17^ | [1] |
| *rs1519096* | 4 | 9805872 | *SLC2A9* | 5.60×10^-41^ | [1] |
| *rs7664572* | 4 | 9808709 | *SLC2A9* | 4.47×10^-17^ | [1] |
| *rs12500086* | 4 | 9809859 | *SLC2A9* | 4.50×10^-41^ | [1] |
| *rs1519095* | 4 | 9810931 | *SLC2A9* | 2.71×10^-17^ | [1] |
| *rs2280333* | 4 | 9811133 | *SLC2A9* | 8.88×10^-62^ | [1] |
| *rs1401438* | 4 | 9814456 | *SLC2A9* | 2.28×10^-43^ | [1] |
| *rs13148356* | 4 | 9817070 | *SLC2A9* | 2.77×10^-61^ | [1] |
| *rs1519094* | 4 | 9817286 | *SLC2A9* | 1.05×10^-18^ | [1] |
| *rs13141706* | 4 | 9818345 | *SLC2A9* | 1.31×10^-61^ | [1] |
| *rs6855095* | 4 | 9819977 | *SLC2A9* | 1.99×10^-63^ | [1] |
| *rs1107912* | 4 | 9820179 | *SLC2A9* | 1.02×10^-63^ | [1] |
| *rs4697892* | 4 | 9823837 | *SLC2A9* | 8.08×10^-82^ | [1] |
| *rs1980220* | 4 | 9824636 | *SLC2A9* | 1.27×10^-91^ | [1] |
| *rs4697893* | 4 | 9826183 | *SLC2A9* | 3.52×10^-26^ | [1] |
| *rs6822889* | 4 | 9826757 | *SLC2A9* | 4.58×10^-67^ | [1] |
| *rs4621429* | 4 | 9826870 | *SLC2A9* | 1.80×10^-92^ | [1] |
| *rs4697895* | 4 | 9828484 | *SLC2A9* | 9.61×10^-92^ | [1] |
| *rs1401440* | 4 | 9832512 | *SLC2A9* | 6.32×10^-67^ | [1] |
| *rs1464258* | 4 | 9834107 | *SLC2A9* | 2.76×10^-67^ | [1] |
| *rs16889260* | 4 | 9834896 | *SLC2A9* | 3.53×10^-98^ | [1] |
| *rs16889264* | 4 | 9834999 | *SLC2A9* | 1.45×10^-13^ | [1] |
| *rs12505312* | 4 | 9837090 | *SLC2A9* | 1.31×10^-91^ | [1] |
| *rs10939552* | 4 | 9838548 | *SLC2A9* | 2.04×10^-91^ | [1] |
| *rs1914874* | 4 | 9839490 | *SLC2A9* | 1.85×10^-8^ | [6] |
| *rs10031303* | 4 | 9841690 | *SLC2A9* | 7.00×10^-66^ | [1] |
| *rs950310* | 4 | 9842850 | *SLC2A9* | 6.28×10^-98^ | [1] |
| *rs10939558* | 4 | 9844162 | *SLC2A9* | 9.04×10^-60^ | [1] |
| *rs7683831* | 4 | 9845079 | *SLC2A9* | 3.44×10^-61^ | [1] |
| *rs13141635* | 4 | 9845985 | *SLC2A9* | 1.71×10^-63^ | [1] |
| *rs1976792* | 4 | 9846309 | *SLC2A9* | 4.21×10^-82^ | [1] |
| *rs13119059* | 4 | 9847860 | *SLC2A9* | 2.99×10^-63^ | [1] |
| *rs11721988* | 4 | 9848899 | *SLC2A9* | 5.97×10^-92^ | [1] |
| *rs2176644* | 4 | 9849528 | *SLC2A9* | 1.71×10^-91^ | [1] |
| *rs6812007* | 4 | 9850220 | *SLC2A9* | 4.38×10^-61^ | [1] |
| *rs4697898* | 4 | 9851827 | *SLC2A9* | 1.12×10^-10^ | [8] |
| *rs4697899* | 4 | 9851875 | *SLC2A9* | 3.66×10^-10^ | [8] |
| *rs6831796* | 4 | 9852898 | *SLC2A9* | 7.38×10^-89^ | [1] |
| *rs6826806* | 4 | 9856469 | *SLC2A9* | 3.16×10^-88^ | [1] |
| *rs4507358* | 4 | 9857956 | *SLC2A9* | 9.81×10^-11^ | [8] |
| *rs4697900* | 4 | 9859976 | *SLC2A9* | 1.86×10^-44^ | [1] |
| *rs9684729* | 4 | 9862036 | *SLC2A9* | 4.50×10^-62^ | [1] |
| *rs4697903* | 4 | 9865426 | *SLC2A9* | 9.99×10^-89^ | [1] |
| *rs2292917* | 4 | 9867277 | *SLC2A9* | 1.98×10^-63^ | [1] |
| *rs2139243* | 4 | 9867502 | *SLC2A9* | 2.02×10^-10^ | [6] |
| *rs883041* | 4 | 9868346 | *SLC2A9* | 2.02×10^-89^ | [1] |
| *rs13105954* | 4 | 9868417 | *SLC2A9* | 2.64×10^-58^ | [1] |
| *rs939134* | 4 | 9868593 | *SLC2A9* | 1.05×10^-9^ | [6] |
| *rs884573* | 4 | 9869734 | *SLC2A9* | 2.04×10^-75^ | [1] |
| *rs1048252* | 4 | 9870535 | *SLC2A9* | 1.92×10^-61^ | [1] |
| *rs1568318* | 4 | 9871541 | *SLC2A9* | 7.99×10^-11^ | [6] |
| *rs16889842* | 4 | 9872013 | *SLC2A9* | 4.04×10^-13^ | [1] |
| *rs2867394* | 4 | 9872475 | *SLC2A9* | 1.27×10^-99^ | [1] |
| *rs13148571* | 4 | 9873855 | *SLC2A9* | 6.32×10^-71^ | [1] |
| *rs12647883* | 4 | 9878464 | *SLC2A9* | 6.90×10^-13^ | [1] |
| *rs1519098* | 4 | 9881158 | *SLC2A9* | 3.57×10^-63^ | [1] |
| *rs6824636* | 4 | 9881851 | *SLC2A9* | 2.83×10^-71^ | [1] |
| *rs6449090* | 4 | 9884536 | *SLC2A9* | 1.11×10^-60^ | [1] |
| *rs10939599* | 4 | 9886860 | *SLC2A9* | 1.08×10^-95^ | [1] |
| *rs10939600* | 4 | 9886931 | *SLC2A9* | 5.87×10^-60^ | [1] |
| *rs9993652* | 4 | 9888849 | *SLC2A9* | 3.87×10^-54^ | [1] |
| *rs6818572* | 4 | 9889448 | *SLC2A9* | 1.19×10^-91^ | [1] |
| *rs1107710* | 4 | 9890708 | *SLC2A9* | 1.57×10^-96^ | [1] |
| *rs938563* | 4 | 9890998 | *SLC2A9* | 3.54×10^-9^ | [6] |
| *rs938562* | 4 | 9891031 | *SLC2A9* | 6.46×10^-97^ | [1] |
| *rs10939602* | 4 | 9892102 | *SLC2A9* | 3.15×10^-55^ | [1] |
| *rs4697692* | 4 | 9893197 | *SLC2A9* | 7.14×10^-10^ | [6] |
| *rs12644047* | 4 | 9893403 | *SLC2A9* | 2.64×10^-102^ | [1] |
| *rs7669444* | 4 | 9893577 | *SLC2A9* | 3.52×10^-61^ | [1] |
| *rs4697693* | 4 | 9895860 | *SLC2A9* | 1.14×10^-92^ | [1] |
| *rs10939605* | 4 | 9896293 | *SLC2A9* | 2.29×10^-94^ | [1] |
| *rs4697694* | 4 | 9896642 | *SLC2A9* | 2.03×10^-93^ | [1] |
| *rs6449097* | 4 | 9896734 | *SLC2A9* | 1.06×10^-69^ | [1] |
| *rs13115121* | 4 | 9897342 | *SLC2A9* | 2.85×10^-69^ | [1] |
| *rs13129868* | 4 | 9897372 | *SLC2A9* | 1.67×10^-69^ | [1] |
| *rs12505366* | 4 | 9897520 | *SLC2A9* | 4.85×10^-13^ | [1] |
| *rs13122026* | 4 | 9898207 | *SLC2A9* | 1.96×10^-69^ | [1] |
| *rs6449100* | 4 | 9901563 | *SLC2A9* | 1.20×10^-63^ | [1] |
| *rs4697910* | 4 | 9901874 | *SLC2A9* | 5.95×10^-94^ | [1] |
| *rs11732681* | 4 | 9902876 | *SLC2A9* | 2.07×10^-72^ | [1] |
| *rs11737685* | 4 | 9903121 | *SLC2A9* | 4.88×10^-8^ | [6] |
| *rs6829755* | 4 | 9903518 | *SLC2A9* | 3.56×10^-8^ | [1] |
| *rs7694136* | 4 | 9908791 | *SLC2A9* | 1.15×10^-69^ | [1] |
| *rs2280202* | 4 | 9909295 | *SLC2A9* | 1.62×10^-64^ | [1] |
| *rs2280205* | 4 | 9909923 | *SLC2A9* | 5.08×10^-23^ | [9] |
| *rs11734893* | 4 | 9910441 | *SLC2A9* | 6.45×10^-70^ | [1] |
| *rs13103429* | 4 | 9910635 | *SLC2A9* | 6.39×10^-70^ | [1] |
| *rs13108825* | 4 | 9910663 | *SLC2A9* | 3.45×10^-61^ | [1] |
| *rs11722228* | 4 | 9915741 | *SLC2A9* | 3.28×10^-261^ | [1] |
| *rs4697695* | 4 | 9915850 | *SLC2A9* | 1.44×10^-30^ | [6] |
| *rs10516194* | 4 | 9916209 | *SLC2A9* | 1.25×10^-73^ | [1] |
| *rs10805346* | 4 | 9920347 | *SLC2A9* | 0.00×10^0^ | [1] |
| *rs874432* | 4 | 9920606 | *SLC2A9* | 1.15×10^-61^ | [6] |
| *rs6823877* | 4 | 9921931 | *SLC2A9* | 1.69×10^-221^ | [1] |
| *rs3733591* | 4 | 9922130 | *SLC2A9* | 1.27×10^-17^ | [10] |
| *rs16890979* | 4 | 9922167 | *SLC2A9* | 4.05×10^-116^ | [11] |
| *rs938564* | 4 | 9922573 | *SLC2A9* | 0.00×10^0^ | [1] |
| *rs734553* | 4 | 9923004 | *SLC2A9* | 0.00×10^0^ | [1] |
| *rs6832439* | 4 | 9924319 | *SLC2A9* | 5.64×10^-11^ | [12] |
| *rs938553* | 4 | 9925526 | *RP13-560N11.1* | 7.94×10^-40^ | [1] |
| *rs938554* | 4 | 9925692 | *RP13-560N11.1* | 0.00×10^0^ | [1] |
| *rs938555* | 4 | 9926051 | *SLC2A9* | 0.00×10^0^ | [1] |
| *rs10939614* | 4 | 9926613 | *SLC2A9* | 6.06×10^-270^ | [1] |
| *rs13129697* | 4 | 9926967 | *SLC2A9* | 3.84×10^-193^ | [8] |
| *rs6838021* | 4 | 9927620 | *SLC2A9* | 0.00×10^0^ | [1] |
| *rs881971* | 4 | 9930962 | *SLC2A9* | 2.62×10^-257^ | [1] |
| *rs737267* | 4 | 9934744 | *SLC2A9* | 6.96×10^-20^ | [2] |
| *rs6855911* | 4 | 9935910 | *SLC2A9* | 2.36×10^-62^ | [6] |
| *rs7670751* | 4 | 9938773 | *SLC2A9* | 0.00×10^0^ | [1] |
| *rs4447863* | 4 | 9938969 | *SLC2A9* | 0.00×10^0^ | [1] |
| *rs938558* | 4 | 9939205 | *SLC2A9* | 0.00×10^0^ | [1] |
| *rs4511996* | 4 | 9939818 | *SLC2A9* | 5.65×10^-53^ | [1] |
| *rs5028843* | 4 | 9940806 | *SLC2A9* | 0.00×10^0^ | [1] |
| *rs4697913* | 4 | 9941262 | *SLC2A9* | 0.00×10^0^ | [1] |
| *rs7675964* | 4 | 9941434 | *SLC2A9* | 0.00×10^0^ | [1] |
| *rs4697698* | 4 | 9942577 | *SLC2A9* | 1.15×10^-19^ | [6] |
| *rs7669296* | 4 | 9942642 | *SLC2A9* | 9.21×10^-9^ | [1] |
| *rs149454410* | 4 | 9943624 | *SLC2A9* | 2.00×10^-16^ | [4] |
| *rs4292327* | 4 | 9943700 | *SLC2A9* | 8.13×10^-55^ | [1] |
| *rs12498742* | 4 | 9944052 | *SLC2A9* | 0.00×10^0^ | [1] |
| *rs6449144* | 4 | 9944650 | *SLC2A9* | 8.08×10^-271^ | [1] |
| *rs4235346* | 4 | 9945296 | *SLC2A9* | 3.08×10^-18^ | [6] |
| *rs4697700* | 4 | 9945792 | *SLC2A9* | 7.01×10^-60^ | [6] |
| *rs4697701* | 4 | 9946095 | *SLC2A9* | 0.00×10^0^ | [1] |
| *rs16891234* | 4 | 9946163 | *SLC2A9* | 4.07×10^-42^ | [1] |
| *rs4475146* | 4 | 9946656 | *SLC2A9* | 0.00×10^0^ | [1] |
| *rs2018643* | 4 | 9947121 | *SLC2A9* | 2.45×10^-24^ | [6] |
| *rs1122141* | 4 | 9947278 | *SLC2A9* | 1.43×10^-245^ | [1] |
| *rs4621431* | 4 | 9947590 | *SLC2A9* | 5.22×10^-249^ | [1] |
| *rs4339211* | 4 | 9947658 | *SLC2A9* | 2.22×10^-228^ | [1] |
| *rs7694997* | 4 | 9947811 | *SLC2A9* | 7.91×10^-250^ | [1] |
| *rs7686538* | 4 | 9948077 | *SLC2A9* | 1.39×10^-228^ | [1] |
| *rs4580649* | 4 | 9948461 | *SLC2A9* | 9.24×10^-250^ | [1] |
| *rs998676* | 4 | 9948564 | *SLC2A9* | 3.25×10^-287^ | [1] |
| *rs998675* | 4 | 9948829 | *SLC2A9* | 6.36×10^-20^ | [6] |
| *rs12498150* | 4 | 9950537 | *SLC2A9* | 2.78×10^-250^ | [1] |
| *rs12498956* | 4 | 9950705 | *SLC2A9* | 2.17×10^-24^ | [6] |
| *rs13328050* | 4 | 9951120 | *SLC2A9* | 8.87×10^-24^ | [6] |
| *rs1079128* | 4 | 9951221 | *SLC2A9* | 1.31×10^-250^ | [1] |
| *rs9993410* | 4 | 9951264 | *SLC2A9* | 1.13×10^-250^ | [1] |
| *rs11723439* | 4 | 9951819 | *SLC2A9* | 0.00×10^0^ | [1] |
| *rs4235347* | 4 | 9951956 | *SLC2A9* | 2.58×10^-242^ | [1] |
| *rs4697914* | 4 | 9952266 | *SLC2A9* | 9.37×10^-36^ | [8] |
| *rs4455410* | 4 | 9953297 | *SLC2A9* | 4.71×10^-23^ | [6] |
| *rs4560411* | 4 | 9953361 | *SLC2A9* | 8.02×10^-222^ | [1] |
| *rs4447861* | 4 | 9953940 | *SLC2A9* | 6.85×10^-194^ | [1] |
| *rs4459990* | 4 | 9954005 | *SLC2A9* | 8.81×10^-222^ | [1] |
| *rs9994266* | 4 | 9954450 | *SLC2A9* | 5.58×10^-22^ | [6] |
| *rs7376948* | 4 | 9954708 | *SLC2A9* | 5.43×10^-242^ | [1] |
| *rs7375587* | 4 | 9954758 | *SLC2A9* | 3.07×10^-242^ | [1] |
| *rs7378305* | 4 | 9954893 | *SLC2A9* | 2.14×10^-242^ | [1] |
| *rs7375599* | 4 | 9954918 | *SLC2A9* | 6.95×10^-19^ | [6] |
| *rs7378340* | 4 | 9955198 | *SLC2A9* | 2.76×10^-23^ | [6] |
| *rs4519796* | 4 | 9955936 | *SLC2A9* | 1.08×10^-230^ | [1] |
| *rs4311316* | 4 | 9955971 | *SLC2A9* | 2.17×10^-23^ | [6] |
| *rs4481233* | 4 | 9956079 | *SLC2A9* | 1.32×10^-58^ | [6] |
| *rs4314284* | 4 | 9956096 | *SLC2A9* | 2.87×10^-23^ | [6] |
| *rs4312757* | 4 | 9956145 | *SLC2A9* | 1.06×10^-247^ | [1] |
| *rs6814664* | 4 | 9956228 | *SLC2A9* | 3.01×10^-242^ | [1] |
| *rs6449155* | 4 | 9956547 | *SLC2A9* | 3.28×10^-242^ | [1] |
| *rs6449156* | 4 | 9956712 | *SLC2A9* | 3.29×10^-256^ | [1] |
| *rs17245436* | 4 | 9958169 | *SLC2A9* | 2.09×10^-243^ | [1] |
| *rs17185835* | 4 | 9958180 | *SLC2A9* | 1.37×10^-243^ | [1] |
| *rs17185870* | 4 | 9958214 | *SLC2A9* | 2.25×10^-243^ | [1] |
| *rs11724510* | 4 | 9958583 | *SLC2A9* | 7.40×10^-244^ | [1] |
| *rs6815001* | 4 | 9958662 | *SLC2A9* | 3.73×10^-223^ | [1] |
| *rs6849717* | 4 | 9958719 | *SLC2A9* | 0.00×10^0^ | [1] |
| *rs6849729* | 4 | 9958732 | *SLC2A9* | 4.58×10^-244^ | [1] |
| *rs6843873* | 4 | 9958788 | *SLC2A9* | 7.99×10^-244^ | [1] |
| *rs6850143* | 4 | 9958924 | *SLC2A9* | 2.72×10^-211^ | [1] |
| *rs6844316* | 4 | 9958977 | *SLC2A9* | 2.96×10^-244^ | [1] |
| *rs6834893* | 4 | 9959123 | *SLC2A9* | 8.58×10^-244^ | [1] |
| *rs10001964* | 4 | 9959275 | *SLC2A9* | 1.53×10^-197^ | [1] |
| *rs4515163* | 4 | 9959603 | *SLC2A9* | 1.05×10^-244^ | [1] |
| *rs6449157* | 4 | 9960442 | *SLC2A9* | 9.35×10^-245^ | [1] |
| *rs6449159* | 4 | 9960498 | *SLC2A9* | 4.16×10^-244^ | [1] |
| *rs7672947* | 4 | 9961368 | *SLC2A9* | 7.03×10^-244^ | [1] |
| *rs17245723* | 4 | 9962218 | *SLC2A9* | 2.56×10^-258^ | [1] |
| *rs11942223* | 4 | 9962765 | *SLC2A9* | 0.00×10^0^ | [1] |
| *rs6823361* | 4 | 9963127 | *SLC2A9* | 3.92×10^-243^ | [1] |
| *rs6836706* | 4 | 9964251 | *SLC2A9* | 4.08×10^-243^ | [1] |
| *rs6850684* | 4 | 9964380 | *SLC2A9* | 5.04×10^-243^ | [1] |
| *rs10018204* | 4 | 9964570 | *SLC2A9* | 6.37×10^-243^ | [1] |
| *rs6839490* | 4 | 9965000 | *SLC2A9* | 9.08×10^-15^ | [6] |
| *rs6856127* | 4 | 9965443 | *SLC2A9* | 3.34×10^-223^ | [1] |
| *rs6840802* | 4 | 9965633 | *SLC2A9* | 1.68×10^-22^ | [6] |
| *rs6449171* | 4 | 9965998 | *SLC2A9* | 7.24×10^-23^ | [6] |
| *rs6449172* | 4 | 9966036 | *SLC2A9* | 3.84×10^-243^ | [1] |
| *rs6449173* | 4 | 9966105 | *SLC2A9* | 0.00×10^0^ | [1] |
| *rs6847019* | 4 | 9966249 | *SLC2A9* | 2.62×10^-53^ | [1] |
| *rs7442295* | 4 | 9966380 | *SLC2A9* | 8.65×10^-65^ | [6] |
| *rs6449174* | 4 | 9966422 | *SLC2A9* | 2.35×10^-23^ | [6] |
| *rs9998811* | 4 | 9966477 | *SLC2A9* | 0.00×10^0^ | [1] |
| *rs7658170* | 4 | 9966593 | *SLC2A9* | 1.62×10^-23^ | [6] |
| *rs6449175* | 4 | 9966610 | *SLC2A9* | 2.23×10^-54^ | [1] |
| *rs7663097* | 4 | 9966791 | *SLC2A9* | 1.35×10^-249^ | [1] |
| *rs7676733* | 4 | 9966956 | *SLC2A9* | 2.02×10^-243^ | [1] |
| *rs10017674* | 4 | 9967053 | *SLC2A9* | 3.77×10^-206^ | [1] |
| *rs7435196* | 4 | 9967556 | *SLC2A9* | 1.07×10^-243^ | [1] |
| *rs6449176* | 4 | 9967843 | *SLC2A9* | 9.35×10^-244^ | [1] |
| *rs6449178* | 4 | 9968684 | *SLC2A9* | 9.11×10^-23^ | [6] |
| *rs6449179* | 4 | 9969117 | *SLC2A9* | 2.45×10^-243^ | [1] |
| *rs7677710* | 4 | 9969517 | *SLC2A9* | 1.25×10^-243^ | [1] |
| *rs7683283* | 4 | 9969974 | *SLC2A9* | 4.40×10^-243^ | [1] |
| *rs7376960* | 4 | 9970570 | *SLC2A9* | 0.00×10^0^ | [1] |
| *rs6449183* | 4 | 9970691 | *SLC2A9* | 1.55×10^-229^ | [1] |
| *rs4292328* | 4 | 9970962 | *SLC2A9* | 0.00×10^0^ | [1] |
| *rs4473653* | 4 | 9971058 | *SLC2A9* | 3.97×10^-242^ | [1] |
| *rs7439210* | 4 | 9971749 | *SLC2A9* | 0.00×10^0^ | [1] |
| *rs13132625* | 4 | 9972163 | *SLC2A9* | 1.64×10^-18^ | [1] |
| *rs13103690* | 4 | 9972778 | *SLC2A9* | 2.99×10^-265^ | [1] |
| *rs13103879* | 4 | 9972879 | *SLC2A9* | 8.61×10^-244^ | [1] |
| *rs6852441* | 4 | 9973744 | *SLC2A9* | 1.72×10^-242^ | [1] |
| *rs6449201* | 4 | 9973894 | *SLC2A9* | 1.74×10^-242^ | [1] |
| *rs6449202* | 4 | 9974043 | *SLC2A9* | 2.70×10^-222^ | [1] |
| *rs1071988* | 4 | 9974638 | *SLC2A9* | 0.00×10^0^ | [1] |
| *rs4505821* | 4 | 9978094 | *SLC2A9* | 7.90×10^-55^ | [1] |
| *rs62295971* | 4 | 9978142 | *SLC2A9* | 1.11×10^-10^ | [10] |
| *rs16868246* | 4 | 9978305 | *SLC2A9* | 0.00×10^0^ | [1] |
| *rs13103497* | 4 | 9979262 | *SLC2A9* | 7.11×10^-213^ | [1] |
| *rs13144899* | 4 | 9979302 | *SLC2A9* | 5.12×10^-19^ | [1] |
| *rs13125476* | 4 | 9979614 | *SLC2A9* | 7.74×10^-19^ | [1] |
| *rs11723970* | 4 | 9980462 | *SLC2A9* | 9.59×10^-290^ | [1] |
| *rs11722229* | 4 | 9980697 | *SLC2A9* | 0.00×10^0^ | [1] |
| *rs882223* | 4 | 9981625 | *SLC2A9* | 0.00×10^0^ | [1] |
| *rs4364264* | 4 | 9981683 | *SLC2A9* | 9.42×10^-18^ | [1] |
| *rs13131257* | 4 | 9981889 | *SLC2A9* | 9.17×10^-11^ | [12] |
| *rs13145758* | 4 | 9981997 | *SLC2A9* | 0.00×10^0^ | [1] |
| *rs13125029* | 4 | 9982029 | *SLC2A9* | 2.83×10^-194^ | [1] |
| *rs13125209* | 4 | 9982044 | *SLC2A9* | 0.00×10^0^ | [1] |
| *rs13115193* | 4 | 9982191 | *SLC2A9* | 2.35×10^-265^ | [1] |
| *rs13125646* | 4 | 9982330 | *SLC2A9* | 0.00×10^0^ | [1] |
| *rs10003001* | 4 | 9984475 | *SLC2A9* | 3.98×10^-55^ | [1] |
| *rs10033612* | 4 | 9985006 | *SLC2A9* | 2.96×10^-55^ | [1] |
| *rs11723591* | 4 | 9985398 | *SLC2A9* | 2.59×10^-265^ | [1] |
| *rs7660895* | 4 | 9985445 | *SLC2A9* | 1.47×10^-9^ | [12] |
| *rs7680126* | 4 | 9985596 | *SLC2A9* | 1.70×10^-33^ | [6] |
| *rs17246501* | 4 | 9985710 | *SLC2A9* | 1.67×10^-18^ | [6] |
| *rs9992406* | 4 | 9986288 | *SLC2A9* | 7.73×10^-55^ | [1] |
| *rs6849736* | 4 | 9986783 | *SLC2A9* | 3.12×10^-17^ | [1] |
| *rs6836200* | 4 | 9986915 | *SLC2A9* | 1.70×10^-16^ | [1] |
| *rs3733590* | 4 | 9987226 | *SLC2A9* | 1.50×10^-13^ | [13] |
| *rs4385059* | 4 | 9989233 | *SLC2A9* | 2.07×10^-56^ | [6] |
| *rs4502681* | 4 | 9990172 | *SLC2A9* | 5.58×10^-20^ | [1] |
| *rs17187075* | 4 | 9990328 | *SLC2A9* | 8.78×10^-250^ | [1] |
| *rs10011206* | 4 | 9991955 | *SLC2A9* | 2.82×10^-55^ | [1] |
| *rs7678012* | 4 | 9993772 | *SLC2A9* | 2.40×10^-250^ | [1] |
| *rs7663032* | 4 | 9993838 | *SLC2A9* | 1.41×10^-9^ | [14] |
| *rs6449213* | 4 | 9994215 | *SLC2A9* | 2.20×10^-104^ | [15] |
| *rs3775948* | 4 | 9995182 | *SLC2A9* | 8.20×10^-64^ | [16] |
| *rs12499857* | 4 | 9995376 | *SLC2A9* | 6.34×10^-243^ | [1] |
| *rs3796842* | 4 | 9995851 | *SLC2A9* | 6.82×10^-253^ | [1] |
| *rs9998739* | 4 | 9996509 | *SLC2A9* | 1.60×10^-55^ | [1] |
| *rs13111638* | 4 | 9996890 | *SLC2A9* | 7.21×10^-57^ | [6] |
| *rs4547795* | 4 | 9997060 | *SLC2A9* | 4.87×10^-55^ | [1] |
| *rs4529048* | 4 | 9997112 | *SLC2A9* | 1.17×10^-14^ | [17] |
| *rs3733588* | 4 | 9997303 | *SLC2A9* | 1.91×10-66 | [6] |
| *rs3733587* | 4 | 9997434 | *SLC2A9* | 8.42×10-56 | [1] |
| *rs7669607* | 4 | 9997801 | *SLC2A9* | 9.18×10-63 | [6] |
| *rs16891971* | 4 | 9998376 | *SLC2A9* | 1.48×10-15 | [1] |
| *rs10939650* | 4 | 9998440 | *SLC2A9* | 4.22×10-112 | [11] |
| *rs13113918* | 4 | 9998493 | *SLC2A9* | 0.00×100 | [1] |
| *rs10008035* | 4 | 9999335 | *SLC2A9* | 5.68×10^-56^ | [1] |
| *rs7696536* | 4 | 10000236 | *SLC2A9* | 4.33×10^-56^ | [1] |
| *rs7678287* | 4 | 10000501 | *SLC2A9* | 1.57×10^-42^ | [18] |
| *rs1014290* | 4 | 10001861 | *SLC2A9* | 2.30×10^-64^ | [16] |
| *rs7696895* | 4 | 10002425 | *SLC2A9* | 1.13×10^-55^ | [1] |
| *rs9991278* | 4 | 10002665 | *SLC2A9* | 4.62×10^-58^ | [6] |
| *rs4622999* | 4 | 10003395 | *SLC2A9* | 1.38×10^-234^ | [1] |
| *rs7657096* | 4 | 10004000 | *SLC2A9* | 9.25×10^-16^ | [1] |
| *rs17247314* | 4 | 10004743 | *SLC2A9* | 2.28×10^-233^ | [1] |
| *rs10023068* | 4 | 10004832 | *SLC2A9* | 5.21×10^-44^ | [6] |
| *rs6853437* | 4 | 10005435 | *SLC2A9* | 2.32×10^-44^ | [6] |
| *rs10022499* | 4 | 10006537 | *SLC2A9* | 0.00×10^0^ | [1] |
| *rs9291640* | 4 | 10007086 | *SLC2A9* | 5.92×10^-44^ | [6] |
| *rs9291642* | 4 | 10007275 | *SLC2A9* | 0.00×10^0^ | [1] |
| *rs4543113* | 4 | 10008305 | *RP11-448G15.1* | 3.73×10^-202^ | [1] |
| *rs6845554* | 4 | 10013173 | *SLC2A9* | 5.95×10^-217^ | [1] |
| *rs3756236* | 4 | 10013463 | *SLC2A9* | 5.46×10^-243^ | [1] |
| *rs6827754* | 4 | 10018153 | *SLC2A9* | 6.50×10^-22^ | [6] |
| *rs13128385* | 4 | 10019563 | *SLC2A9* | 1.24×10^-13^ | [1] |
| *rs13133766* | 4 | 10019732 | *SLC2A9* | 1.50×10^-242^ | [1] |
| *rs3775942* | 4 | 10020307 | *SLC2A9* | 8.39×10^-9^ | [1] |
| *rs2240720* | 4 | 10020480 | *SLC2A9* | 1.84×10^-242^ | [1] |
| *rs2240721* | 4 | 10020564 | *SLC2A9* | 2.75×10^-242^ | [1] |
| *rs2240723* | 4 | 10021151 | *SLC2A9* | 1.46×10^-8^ | [1] |
| *rs2240724* | 4 | 10021290 | *SLC2A9* | 4.64×10^-239^ | [1] |
| *rs6849273* | 4 | 10021595 | *SLC2A9* | 1.88×10^-215^ | [1] |
| *rs2276961* | 4 | 10022981 | *SLC2A9* | 3.12×10^-41^ | [11] |
| *rs12509955* | 4 | 10024303 | *SLC2A9* | 2.14×10^-54^ | [6] |
| *rs3775940* | 4 | 10025163 | *SLC2A9* | 7.11×10^-22^ | [6] |
| *rs6826764* | 4 | 10030794 | *SLC2A9* | 9.15×10^-43^ | [6] |
| *rs6856396* | 4 | 10031163 | *SLC2A9* | 3.18×10^-9^ | [14] |
| *rs12506455* | 4 | 10031569 | *SLC2A9* | 3.43×10^-239^ | [1] |
| *rs10939663* | 4 | 10032516 | *SLC2A9* | 3.08×10^-191^ | [1] |
| *rs12506122* | 4 | 10033538 | *SLC2A9* | 5.82×10^-214^ | [1] |
| *rs13146686* | 4 | 10034933 | *SLC2A9* | 1.76×10^-19^ | [6] |
| *rs11722930* | 4 | 10035454 | *SLC2A9* | 5.85×10^-233^ | [1] |
| *rs16892419* | 4 | 10035702 | *SLC2A9* | 4.15×10^-8^ | [1] |
| *rs16892420* | 4 | 10035715 | *SLC2A9* | 1.88×10^-8^ | [1] |
| *rs10006397* | 4 | 10036140 | *SLC2A9* | 0.00×10^0^ | [1] |
| *rs11727199* | 4 | 10036190 | *SLC2A9* | 1.18×10^-232^ | [1] |
| *rs3733585* | 4 | 10036339 | *SLC2A9* | 1.38×10^-238^ | [1] |
| *rs3822250* | 4 | 10036630 | *SLC2A9* | 9.54×10^-9^ | [1] |
| *rs11731110* | 4 | 10037346 | *SLC2A9* | 1.59×10^-238^ | [1] |
| *rs10939665* | 4 | 10037628 | *SLC2A9* | 6.44×10^-238^ | [1] |
| *rs10012779* | 4 | 10038112 | *SLC2A9* | 1.52×10^-104^ | [1] |
| *rs13139055* | 4 | 10038924 | *SLC2A9* | 3.31×10^-212^ | [1] |
| *rs13115776* | 4 | 10040189 | *SLC2A9* | 2.90×10^-212^ | [1] |
| *rs16892474* | 4 | 10040220 | *SLC2A9* | 4.86×10^-8^ | [1] |
| *rs12508991* | 4 | 10041104 | *SLC2A9* | 2.07×10^-222^ | [1] |
| *rs10029311* | 4 | 10041134 | *SLC2A9* | 7.87×10^-104^ | [1] |
| *rs7679916* | 4 | 10042160 | *SLC2A9* | 6.00×10^-231^ | [1] |
| *rs7349721* | 4 | 10042562 | *SLC2A9* | 0.00×10^0^ | [1] |
| *rs13101785* | 4 | 10042915 | *SLC2A9* | 1.69×10^-234^ | [1] |
| *rs13137343* | 4 | 10043028 | *SLC2A9* | 6.65×10^-235^ | [1] |
| *rs13110307* | 4 | 10044364 | *SLC2A9* | 1.41×10^-211^ | [1] |
| *rs13129453* | 4 | 10044784 | *SLC2A9* | 9.24×10^-212^ | [1] |
| *rs4529049* | 4 | 10045389 | *SLC2A9* | 2.71×10^-212^ | [1] |
| *rs4637402* | 4 | 10045430 | *SLC2A9* | 8.70×10^-70^ | [8] |
| *rs10939669* | 4 | 10045827 | *SLC2A9* | 1.56×10^-236^ | [1] |
| *rs11734375* | 4 | 10046298 | *SLC2A9* | 1.09×10^-16^ | [19] |
| *rs4608811* | 4 | 10049675 | *SLC2A9* | 2.30×10^-42^ | [6] |
| *rs733175* | 4 | 10050141 | *SLC2A9* | 1.03×10^-15^ | [10] |
| *rs6829727* | 4 | 10051672 | *SLC2A9* | 3.03×10^-22^ | [6] |
| *rs13120348* | 4 | 10053155 | *SLC2A9* | 7.90×10^-23^ | [6] |
| *rs7671266* | 4 | 10056376 | *SLC2A9* | 1.16×10^-52^ | [6] |
| *rs10516198* | 4 | 10059448 | *5' of SLC2A9* | 4.83×10^-35^ | [1] |
| *rs714873* | 4 | 10059618 | *5' of SLC2A9* | 1.02×10^-264^ | [1] |
| *rs6834555* | 4 | 10062326 | *5' of SLC2A9* | 4.30×10^-14^ | [10] |
| *rs6820756* | 4 | 10062849 | *5' of SLC2A9* | 4.91×10^-26^ | [6] |
| *rs12506004* | 4 | 10066870 | *3' of RP11-448G15.3* | 7.06×10^-141^ | [1] |
| *rs16868313* | 4 | 10068064 | *3' of RP11-448G15.3* | 6.25×10^-11^ | [6] |
| *rs3775938* | 4 | 10069531 | *3' of RP11-448G15.3* | 5.98×10^-15^ | [1] |
| *rs7667452* | 4 | 10072912 | *RP11-448G15.3* | 1.60×10^-14^ | [1] |
| *rs4320137* | 4 | 10072969 | *RP11-448G15.3* | 7.39×10^-40^ | [6] |
| *rs4461524* | 4 | 10074170 | *RP11-448G15.3* | 4.14×10^-54^ | [1] |
| *rs11731597* | 4 | 10075485 | *3' of WDR1* | 4.63×10^-11^ | [6] |
| *rs9926* | 4 | 10076860 | *WDR1* | 1.85×10^-119^ | [1] |
| *rs3775935* | 4 | 10077638 | *WDR1* | 1.65×10^-15^ | [1] |
| *rs2241469* | 4 | 10080462 | *WDR1* | 8.86×10^-16^ | [1] |
| *rs10516200* | 4 | 10082156 | *WDR1* | 1.67×10^-11^ | [6] |
| *rs3756230* | 4 | 10083829 | *WDR1* | 1.70×10^-140^ | [1] |
| *rs2241470* | 4 | 10084570 | *WDR1* | 4.83×10^-114^ | [1] |
| *rs2241473* | 4 | 10085949 | *WDR1* | 2.20×10^-120^ | [1] |
| *rs2241475* | 4 | 10086188 | *WDR1* | 3.55×10^-12^ | [6] |
| *rs3756227* | 4 | 10087995 | *WDR1* | 1.28×10^-128^ | [1] |
| *rs2241480* | 4 | 10089763 | *WDR1* | 2.83×10^-11^ | [6] |
| *rs734122* | 4 | 10089865 | *WDR1* | 2.62×10^-11^ | [6] |
| *rs3796822* | 4 | 10093651 | *WDR1* | 9.64×10^-15^ | [1] |
| *rs3822242* | 4 | 10094904 | *WDR1* | 1.84×10^-169^ | [1] |
| *rs3822239* | 4 | 10095539 | *WDR1* | 6.21×10^-16^ | [1] |
| *rs11727087* | 4 | 10096020 | *WDR1* | 5.00×10^-21^ | [6] |
| *rs3796818* | 4 | 10097976 | *WDR1* | 3.31×10^-140^ | [1] |
| *rs11726271* | 4 | 10098192 | *WDR1* | 3.35×10^-172^ | [1] |
| *rs2241482* | 4 | 10099814 | *WDR1* | 2.83×10^-16^ | [1] |
| *rs2241483* | 4 | 10099831 | *WDR1* | 8.87×10^-148^ | [1] |
| *rs2241486* | 4 | 10101083 | *WDR1* | 1.10×10^-137^ | [1] |
| *rs2241488* | 4 | 10101131 | *WDR1* | 9.14×10^-129^ | [1] |
| *rs6830786* | 4 | 10101443 | *WDR1* | 3.57×10^-139^ | [1] |
| *rs4697917* | 4 | 10101960 | *WDR1* | 3.70×10^-16^ | [1] |
| *rs16868326* | 4 | 10102593 | *WDR1* | 9.56×10^-137^ | [1] |
| *rs12499240* | 4 | 10103890 | *WDR1* | 3.41×10^-15^ | [1] |
| *rs717615* | 4 | 10104670 | *WDR1* | 1.01×10^-16^ | [6] |
| *rs717614* | 4 | 10104788 | *WDR1* | 6.01×10^-15^ | [20] |
| *rs3756224* | 4 | 10105739 | *WDR1* | 9.45×10^-10^ | [21] |
| *rs3756223* | 4 | 10105797 | *WDR1* | 5.48×10^-9^ | [6] |
| *rs12509714* | 4 | 10107091 | *WDR1* | 8.25×10^-28^ | [6] |
| *rs4697922* | 4 | 10110605 | *WDR1* | 4.58×10^-16^ | [1] |
| *rs4459989* | 4 | 10112602 | *WDR1* | 1.49×10^-31^ | [1] |
| *rs2241468* | 4 | 10113905 | *WDR1* | 1.03×10^-137^ | [1] |
| *rs4604059* | 4 | 10115065 | *WDR1* | 3.04×10^-18^ | [6] |
| *rs4393994* | 4 | 10115121 | *WDR1* | 3.49×10^-15^ | [1] |
| *rs12498927* | 4 | 10115523 | *WDR1* | 1.24×10^-15^ | [6] |
| *rs10939710* | 4 | 10116801 | *WDR1* | 5.43×10^-34^ | [1] |
| *rs35782983* | 4 | 10117728 | *WDR1* | 2.07×10^-94^ | [11] |
| *rs3822236* | 4 | 10119961 | *5' of WDR1* | 6.53×10^-231^ | [1] |
| *rs12374320* | 4 | 10120609 | *5' of WDR1* | 3.15×10^-34^ | [1] |
| *rs4697708* | 4 | 10121189 | *5' of WDR1* | 1.71×10^-26^ | [6] |
| *rs3756215* | 4 | 10121260 | *5' of WDR1* | 2.88×10^-102^ | [1] |
| *rs4697710* | 4 | 10122649 | *5' of WDR1* | 2.21×10^-231^ | [1] |
| *rs6825888* | 4 | 10122734 | *5' of WDR1* | 3.83×10^-9^ | [6] |
| *rs4235354* | 4 | 10122942 | *5' of WDR1* | 2.79×10^-25^ | [1] |
| *rs4235355* | 4 | 10123078 | *5' of WDR1* | 3.48×10^-199^ | [1] |
| *rs4235356* | 4 | 10123106 | *5' of WDR1* | 2.85×10^-231^ | [1] |
| *rs4311315* | 4 | 10123191 | *5' of WDR1* | 2.52×10^-28^ | [1] |
| *rs12506893* | 4 | 10123665 | *5' of WDR1* | 1.69×10^-119^ | [1] |
| *rs10516201* | 4 | 10123941 | *5' of WDR1* | 8.17×10^-9^ | [6] |
| *rs4697924* | 4 | 10124239 | *5' of WDR1* | 4.21×10^-17^ | [6] |
| *rs4697926* | 4 | 10124567 | *5' of WDR1* | 2.92×10^-17^ | [6] |
| *rs4444830* | 4 | 10124819 | *5' of WDR1* | 1.37×10^-11^ | [6] |
| *rs4456954* | 4 | 10124838 | *5' of WDR1* | 3.04×10^-86^ | [1] |
| *rs715979* | 4 | 10125242 | *5' of WDR1* | 2.57×10^-114^ | [1] |
| *rs3886038* | 4 | 10125255 | *5' of WDR1* | 1.18×10^-8^ | [6] |
| *rs7699512* | 4 | 10125808 | *5' of WDR1* | 1.42×10^-169^ | [1] |
| *rs7699671* | 4 | 10125874 | *5' of WDR1* | 3.16×10^-200^ | [1] |
| *rs11722989* | 4 | 10126139 | *5' of WDR1* | 2.64×10^-16^ | [6] |
| *rs11723016* | 4 | 10126189 | *5' of WDR1* | 1.12×10^-153^ | [1] |
| *rs6449286* | 4 | 10126699 | *5' of WDR1* | 4.10×10^-119^ | [1] |
| *rs4619888* | 4 | 10126978 | *5' of WDR1* | 6.89×10^-126^ | [1] |
| *rs4467562* | 4 | 10127141 | *5' of WDR1* | 1.44×10^-122^ | [1] |
| *rs10001106* | 4 | 10127441 | *5' of WDR1* | 8.06×10^-137^ | [1] |
| *rs11722643* | 4 | 10127484 | *5' of WDR1* | 1.39×10^-9^ | [1] |
| *rs17250843* | 4 | 10127978 | *5' of WDR1* | 5.19×10^-24^ | [1] |
| *rs715259* | 4 | 10128071 | *5' of WDR1* | 1.04×10^-9^ | [1] |
| *rs715260* | 4 | 10128146 | *5' of WDR1* | 3.23×10^-127^ | [1] |
| *rs7667775* | 4 | 10128536 | *5' of WDR1* | 4.29×10^-103^ | [1] |
| *rs10155145* | 4 | 10128800 | *5' of WDR1* | 1.84×10^-10^ | [1] |
| *rs12502342* | 4 | 10129916 | *5' of WDR1* | 5.22×10^-10^ | [1] |
| *rs12502368* | 4 | 10129953 | *5' of WDR1* | 9.02×10^-10^ | [1] |
| *rs12502556* | 4 | 10130505 | *5' of WDR1* | 3.02×10^-141^ | [1] |
| *rs10009493* | 4 | 10132047 | *5' of WDR1* | 0.00×10^0^ | [1] |
| *rs12501597* | 4 | 10132137 | *5' of WDR1* | 2.22×10^-127^ | [1] |
| *rs6449289* | 4 | 10132543 | *5' of WDR1* | 7.69×10^-120^ | [1] |
| *rs881641* | 4 | 10133747 | *5' of WDR1* | 1.90×10^-136^ | [1] |
| *rs881642* | 4 | 10133873 | *5' of WDR1* | 1.09×10^-126^ | [1] |
| *rs881643* | 4 | 10134117 | *5' of WDR1* | 1.06×10^-9^ | [6] |
| *rs17197769* | 4 | 10134336 | *5' of WDR1* | 4.67×10^-136^ | [1] |
| *rs1109472* | 4 | 10134448 | *5' of WDR1* | 1.63×10^-9^ | [6] |
| *rs11938608* | 4 | 10135849 | *5' of WDR1* | 5.65×10^-122^ | [1] |
| *rs4358401* | 4 | 10136807 | *5' of WDR1* | 7.44×10^-40^ | [1] |
| *rs873984* | 4 | 10137125 | *5' of WDR1* | 1.26×10^-44^ | [1] |
| *rs4399989* | 4 | 10137388 | *5' of WDR1* | 1.65×10^-40^ | [1] |
| *rs4315785* | 4 | 10137607 | *5' of WDR1* | 1.29×10^-40^ | [1] |
| *rs4235357* | 4 | 10137756 | *5' of WDR1* | 2.59×10^-40^ | [1] |
| *rs12507725* | 4 | 10137790 | *5' of WDR1* | 1.04×10^-134^ | [1] |
| *rs17198113* | 4 | 10138470 | *5' of WDR1* | 1.11×10^-8^ | [6] |
| *rs11732828* | 4 | 10138746 | *5' of WDR1* | 2.67×10^-41^ | [1] |
| *rs10939722* | 4 | 10139047 | *5' of WDR1* | 5.11×10^-40^ | [1] |
| *rs10939723* | 4 | 10139105 | *5' of WDR1* | 3.14×10^-24^ | [6] |
| *rs17198197* | 4 | 10139157 | *5' of WDR1* | 5.21×10^-24^ | [1] |
| *rs6449300* | 4 | 10139551 | *5' of WDR1* | 5.46×10^-135^ | [1] |
| *rs6850516* | 4 | 10139978 | *5' of WDR1* | 4.22×10^-38^ | [1] |
| *rs10025456* | 4 | 10140259 | *5' of WDR1* | 1.32×10^-29^ | [1] |
| *rs10022911* | 4 | 10140551 | *5' of WDR1* | 0.00×10^0^ | [1] |
| *rs10020887* | 4 | 10140751 | *5' of WDR1* | 0.00×10^0^ | [1] |
| *rs10012288* | 4 | 10140953 | *5' of WDR1* | 1.57×10^-41^ | [1] |
| *rs4533775* | 4 | 10141404 | *5' of WDR1* | 1.58×10^-24^ | [1] |
| *rs17198547* | 4 | 10141419 | *5' of WDR1* | 4.75×10^-38^ | [6] |
| *rs10805356* | 4 | 10141602 | *5' of WDR1* | 1.21×10^-40^ | [1] |
| *rs4473652* | 4 | 10141617 | *5' of WDR1* | 2.10×10^-24^ | [1] |
| *rs10028503* | 4 | 10141927 | *5' of WDR1* | 9.76×10^-9^ | [6] |
| *rs10015494* | 4 | 10141990 | *5' of WDR1* | 8.25×10^-38^ | [1] |
| *rs10028937* | 4 | 10142366 | *5' of WDR1* | 1.10×10^-8^ | [6] |
| *rs10015872* | 4 | 10142410 | *5' of WDR1* | 1.09×10^-8^ | [6] |
| *rs17251963* | 4 | 10142561 | *5' of WDR1* | 2.08×10^-39^ | [6] |
| *rs10031453* | 4 | 10142659 | *5' of WDR1* | 6.94×10^-38^ | [1] |
| *rs4697713* | 4 | 10143582 | *5' of WDR1* | 6.36×10^-38^ | [1] |
| *rs4697930* | 4 | 10143594 | *5' of WDR1* | 3.27×10^-40^ | [1] |
| *rs4697714* | 4 | 10143786 | *5' of WDR1* | 1.16×10^-38^ | [6] |
| *rs4292329* | 4 | 10144459 | *5' of WDR1* | 3.87×10^-24^ | [1] |
| *rs4697931* | 4 | 10145296 | *5' of WDR1* | 9.08×10^-9^ | [6] |
| *rs4574408* | 4 | 10145568 | *5' of WDR1* | 3.39×10^-8^ | [6] |
| *rs4640669* | 4 | 10145733 | *5' of WDR1* | 5.99×10^-38^ | [6] |
| *rs4484300* | 4 | 10145794 | *5' of WDR1* | 8.29×10^-9^ | [6] |
| *rs4401449* | 4 | 10145998 | *5' of WDR1* | 3.14×10^-40^ | [1] |
| *rs10939730* | 4 | 10146049 | *5' of WDR1* | 2.85×10^-37^ | [1] |
| *rs4697933* | 4 | 10146493 | *5' of WDR1* | 0.00×10^0^ | [1] |
| *rs4697715* | 4 | 10146654 | *5' of WDR1* | 2.78×10^-26^ | [1] |
| *rs6855657* | 4 | 10148148 | *5' of WDR1* | 8.09×10^-39^ | [1] |
| *rs10939732* | 4 | 10148390 | *5' of WDR1* | 1.99×10^-36^ | [1] |
| *rs12507586* | 4 | 10148575 | *5' of WDR1* | 1.31×10^-135^ | [1] |
| *rs4168* | 4 | 10148609 | *5' of WDR1* | 1.13×10^-36^ | [1] |
| *rs11735668* | 4 | 10148655 | *5' of WDR1* | 3.95×10^-33^ | [6] |
| *rs12508413* | 4 | 10148671 | *5' of WDR1* | 1.05×10^-135^ | [1] |
| *rs6813334* | 4 | 10148753 | *5' of WDR1* | 7.73×10^-136^ | [1] |
| *rs6813385* | 4 | 10148828 | *5' of WDR1* | 2.06×10^-27^ | [1] |
| *rs6846402* | 4 | 10148881 | *5' of WDR1* | 1.24×10^-35^ | [1] |
| *rs4697934* | 4 | 10149064 | *5' of WDR1* | 2.08×10^-8^ | [6] |
| *rs4697936* | 4 | 10149595 | *5' of WDR1* | 1.69×10^-49^ | [1] |
| *rs4697937* | 4 | 10149669 | *5' of WDR1* | 1.85×10^-8^ | [6] |
| *rs6826693* | 4 | 10150739 | *5' of WDR1* | 9.76×10^-32^ | [1] |
| *rs6840883* | 4 | 10150864 | *5' of WDR1* | 2.81×10^-36^ | [1] |
| *rs11721682* | 4 | 10151147 | *5' of WDR1* | 4.04×10^-31^ | [6] |
| *rs6827946* | 4 | 10151335 | *5' of WDR1* | 0.00×10^0^ | [1] |
| *rs6827496* | 4 | 10151656 | *5' of WDR1* | 1.89×10^-139^ | [1] |
| *rs6847379* | 4 | 10151704 | *5' of WDR1* | 8.52×10^-38^ | [1] |
| *rs4697940* | 4 | 10152329 | *5' of WDR1* | 1.21×10^-33^ | [1] |
| *rs731069* | 4 | 10152431 | *5' of WDR1* | 3.03×10^-34^ | [1] |
| *rs731070* | 4 | 10152582 | *5' of WDR1* | 9.38×10^-35^ | [1] |
| *rs747357* | 4 | 10152878 | *5' of WDR1* | 4.81×10^-26^ | [1] |
| *rs747356* | 4 | 10153051 | *5' of WDR1* | 3.17×10^-35^ | [1] |
| *rs737601* | 4 | 10153233 | *5' of WDR1* | 2.78×10^-13^ | [1] |
| *rs6814532* | 4 | 10154554 | *5' of WDR1* | 1.00×10^-9^ | [1] |
| *rs11736560* | 4 | 10154872 | *5' of WDR1* | 1.17×10^-13^ | [1] |
| *rs6851524* | 4 | 10155041 | *5' of WDR1* | 9.47×10^-25^ | [1] |
| *rs10939741* | 4 | 10155346 | *5' of WDR1* | 2.01×10^-13^ | [1] |
| *rs11932627* | 4 | 10156177 | *5' of WDR1* | 5.59×10^-11^ | [1] |
| *rs12513376* | 4 | 10156222 | *5' of WDR1* | 1.12×10^-116^ | [1] |
| *rs11929718* | 4 | 10156321 | *5' of WDR1* | 1.74×10^-12^ | [6] |
| *rs12498256* | 4 | 10156544 | *5' of WDR1* | 7.00×10^-12^ | [1] |
| *rs6823778* | 4 | 10158163 | *5' of WDR1* | 3.79×10^-15^ | [10] |
| *rs6834055* | 4 | 10158511 | *5' of WDR1* | 0.00×10^0^ | [1] |
| *rs2241464* | 4 | 10158961 | *5' of WDR1* | 2.31×10^-37^ | [1] |
| *rs2241465* | 4 | 10159020 | *5' of WDR1* | 2.97×10^-151^ | [1] |
| *rs4697941* | 4 | 10160862 | *5' of WDR1* | 1.49×10^-9^ | [6] |
| *rs17450260* | 4 | 10163789 | *5' of WDR1* | 4.45×10^-126^ | [1] |
| *rs6816215* | 4 | 10164027 | *5' of WDR1* | 1.14×10^-69^ | [1] |
| *rs17450372* | 4 | 10164344 | *5' of WDR1* | 1.01×10^-124^ | [1] |
| *rs1009144* | 4 | 10164931 | *5' of WDR1* | 1.20×10^-132^ | [1] |
| *rs17450434* | 4 | 10164961 | *5' of WDR1* | 1.16×10^-132^ | [1] |
| *rs16894270* | 4 | 10165779 | *5' of WDR1* | 6.30×10^-49^ | [1] |
| *rs11729371* | 4 | 10165957 | *5' of WDR1* | 3.12×10^-18^ | [6] |
| *rs11724641* | 4 | 10166994 | *5' of WDR1* | 9.12×10^-123^ | [1] |
| *rs7659717* | 4 | 10167217 | *5' of WDR1* | 2.04×10^-139^ | [1] |
| *rs17385112* | 4 | 10167532 | *5' of WDR1* | 1.87×10^-220^ | [1] |
| *rs17385294* | 4 | 10168447 | *5' of WDR1* | 1.36×10^-132^ | [1] |
| *rs1001216* | 4 | 10168849 | *5' of WDR1* | 1.93×10^-219^ | [1] |
| *rs1001217* | 4 | 10168999 | *5' of WDR1* | 1.13×10^-24^ | [1] |
| *rs11734209* | 4 | 10170371 | *5' of WDR1* | 5.66×10^-36^ | [1] |
| *rs874079* | 4 | 10171105 | *5' of WDR1* | 1.84×10^-19^ | [1] |
| *rs7686718* | 4 | 10171487 | *5' of WDR1* | 4.83×10^-14^ | [1] |
| *rs4697948* | 4 | 10171567 | *5' of WDR1* | 3.34×10^-25^ | [1] |
| *rs4697950* | 4 | 10171644 | *5' of WDR1* | 5.00×10^-13^ | [10] |
| *rs2080076* | 4 | 10171945 | *5' of WDR1* | 9.01×10^-20^ | [1] |
| *rs2098234* | 4 | 10172113 | *5' of WDR1* | 2.40×10^-17^ | [1] |
| *rs6835689* | 4 | 10172479 | *5' of WDR1* | 2.93×10^-25^ | [1] |
| *rs55672974* | 4 | 10173696 | *WDR1-* | 1.85×10^-8^ | [6] |
| *rs10034405* | 4 | 10173696 | *5' of WDR1* | 1.45×10^-19^ | [8] |
| *rs4697718* | 4 | 10174139 | *5' of WDR1* | 1.60×10^-22^ | [8] |
| *rs4697719* | 4 | 10174158 | *5' of WDR1* | 5.30×10^-9^ | [6] |
| *rs4697721* | 4 | 10174217 | *5' of WDR1* | 2.62×10^-141^ | [1] |
| *rs4697954* | 4 | 10174529 | *5' of WDR1* | 4.69×10^-23^ | [8] |
| *rs10017447* | 4 | 10175536 | *5' of WDR1* | 3.08×10^-13^ | [10] |
| *rs10024152* | 4 | 10175689 | *5' of WDR1* | 1.37×10^-23^ | [8] |
| *rs2903827* | 4 | 10175872 | *5' of WDR1* | 0.00×10^0^ | [1] |
| *rs2868414* | 4 | 10177120 | *5' of WDR1* | 9.61×10^-139^ | [1] |
| *rs4697724* | 4 | 10177818 | *5' of WDR1* | 6.66×10^-13^ | [10] |
| *rs17385872* | 4 | 10178168 | *5' of WDR1* | 1.38×10^-10^ | [6] |
| *rs16894579* | 4 | 10178217 | *5' of WDR1* | 2.12×10^-139^ | [1] |
| *rs10033955* | 4 | 10178556 | *5' of WDR1* | 1.67×10^-127^ | [1] |
| *rs11737347* | 4 | 10178922 | *5' of WDR1* | 2.05×10^-138^ | [1] |
| *rs7657551* | 4 | 10179309 | *5' of WDR1* | 1.13×10^-138^ | [1] |
| *rs6449342* | 4 | 10179769 | *5' of WDR1* | 7.89×10^-139^ | [1] |
| *rs4273473* | 4 | 10180622 | *5' of WDR1* | 9.05×10^-9^ | [6] |
| *rs11724112* | 4 | 10180643 | *5' of WDR1* | 2.58×10^-263^ | [1] |
| *rs6811287* | 4 | 10180823 | *5' of WDR1* | 6.13×10^-263^ | [1] |
| *rs723663* | 4 | 10181374 | *5' of WDR1* | 3.12×10^-9^ | [6] |
| *rs12500891* | 4 | 10181387 | *5' of WDR1* | 2.84×10^-10^ | [6] |
| *rs4697956* | 4 | 10181672 | *5' of WDR1* | 1.62×10^-138^ | [1] |
| *rs4697957* | 4 | 10182254 | *5' of WDR1* | 0.00×10^0^ | [1] |
| *rs4697958* | 4 | 10182556 | *5' of WDR1* | 1.28×10^-138^ | [1] |
| *rs887735* | 4 | 10182845 | *5' of WDR1* | 5.43×10^-10^ | [6] |
| *rs887734* | 4 | 10182913 | *5' of WDR1* | 4.00×10^-13^ | [10] |
| *rs887733* | 4 | 10183108 | *5' of WDR1* | 4.08×10^-142^ | [1] |
| *rs887732* | 4 | 10183117 | *5' of WDR1* | 0.00×10^0^ | [1] |
| *rs887731* | 4 | 10183186 | *5' of WDR1* | 1.49×10^-138^ | [1] |
| *rs887729* | 4 | 10183611 | *5' of WDR1* | 3.39×10^-10^ | [6] |
| *rs887728* | 4 | 10183798 | *5' of WDR1* | 1.18×10^-138^ | [1] |
| *rs887727* | 4 | 10183819 | *5' of WDR1* | 1.77×10^-143^ | [1] |
| *rs887725* | 4 | 10183895 | *5' of WDR1* | 1.96×10^-22^ | [1] |
| *rs10025980* | 4 | 10185799 | *5' of WDR1* | 1.54×10^-13^ | [10] |
| *rs11723976* | 4 | 10186251 | *5' of WDR1* | 1.88×10^-14^ | [10] |
| *rs11724092* | 4 | 10186604 | *5' of WDR1* | 1.68×10^-14^ | [10] |
| *rs11722345* | 4 | 10186799 | *5' of WDR1* | 3.68×10^-138^ | [1] |
| *rs4697960* | 4 | 10187263 | *5' of WDR1* | 2.97×10^-143^ | [1] |
| *rs4697961* | 4 | 10187373 | *5' of WDR1* | 4.39×10^-13^ | [10] |
| *rs4697726* | 4 | 10187395 | *5' of WDR1* | 1.61×10^-13^ | [10] |
| *rs56391253* | 4 | 10187580 | *5' of WDR1* | 2.04×10^-13^ | [10] |
| *rs4697727* | 4 | 10187922 | *5' of WDR1* | 2.40×10^-9^ | [6] |
| *rs956312* | 4 | 10188326 | *5' of WDR1* | 7.97×10^-9^ | [20] |
| *rs956311* | 4 | 10188381 | *5' of WDR1* | 6.43×10^-9^ | [6] |
| *rs4697962* | 4 | 10188832 | *5' of WDR1* | 5.57×10^-14^ | [10] |
| *rs11721530* | 4 | 10189162 | *5' of WDR1* | 1.36×10^-9^ | [6] |
| *rs62285986* | 4 | 10189213 | *5' of WDR1* | 1.57×10^-13^ | [10] |
| *rs4697963* | 4 | 10189483 | *5' of WDR1* | 8.29×10^-14^ | [10] |
| *rs4697964* | 4 | 10189812 | *5' of WDR1* | 3.41×10^-138^ | [1] |
| *rs4697965* | 4 | 10190058 | *5' of WDR1* | 2.35×10^-9^ | [6] |
| *rs4697966* | 4 | 10190074 | *5' of WDR1* | 4.13×10^-9^ | [6] |
| *rs28496435* | 4 | 10190318 | *5' of WDR1* | 5.97×10^-14^ | [10] |
| *rs11726987* | 4 | 10190792 | *5' of WDR1* | 8.64×10^-14^ | [10] |
| *rs10000104* | 4 | 10191766 | *5' of WDR1* | 6.59×10^-14^ | [10] |
| *rs2215691* | 4 | 10192108 | *5' of WDR1* | 6.58×10^-14^ | [10] |
| *rs6449351* | 4 | 10192744 | *5' of WDR1* | 8.00×10^-14^ | [10] |
| *rs2159865* | 4 | 10193287 | *5' of WDR1* | 4.84×10^-14^ | [10] |
| *rs10489080* | 4 | 10193427 | *5' of WDR1* | 6.81×10^-10^ | [6] |
| *rs2159864* | 4 | 10193604 | *5' of WDR1* | 1.36×10^-133^ | [1] |
| *rs6832085* | 4 | 10194270 | *5' of WDR1* | 1.26×10^-13^ | [10] |
| *rs6826383* | 4 | 10194504 | *5' of WDR1* | 3.39×10^-137^ | [1] |
| *rs4697728* | 4 | 10196638 | *5' of WDR1* | 1.27×10^-133^ | [1] |
| *rs917825* | 4 | 10196761 | *5' of WDR1* | 3.65×10^-9^ | [6] |
| *rs1860903* | 4 | 10196853 | *5' of WDR1* | 5.92×10^-141^ | [1] |
| *rs929575* | 4 | 10196886 | *5' of WDR1* | 0.00×10^0^ | [1] |
| *rs917823* | 4 | 10197225 | *5' of WDR1* | 2.16×10^-125^ | [1] |
| *rs66769576* | 4 | 10197663 | *5' of WDR1* | 1.76×10^-13^ | [10] |
| *rs6836606* | 4 | 10198086 | *5' of WDR1* | 7.10×10^-14^ | [10] |
| *rs4697968* | 4 | 10198628 | *5' of WDR1* | 4.44×10^-9^ | [6] |
| *rs4697969* | 4 | 10198981 | *5' of WDR1* | 1.18×10^-23^ | [8] |
| *rs11726996* | 4 | 10199139 | *5' of WDR1* | 1.32×10^-13^ | [10] |
| *rs2080075* | 4 | 10199448 | *5' of WDR1* | 6.97×10^-120^ | [1] |
| *rs55878266* | 4 | 10199948 | *5' of WDR1* | 5.07×10^-14^ | [10] |
| *rs55775442* | 4 | 10200204 | *5' of WDR1* | 1.22×10^-13^ | [10] |
| *rs4697729* | 4 | 10200401 | *5' of WDR1* | 2.09×10^-120^ | [1] |
| *rs4697730* | 4 | 10200496 | *5' of WDR1* | 2.58×10^-9^ | [6] |
| *rs4697731* | 4 | 10200718 | *5' of WDR1* | 2.11×10^-13^ | [10] |
| *rs4697971* | 4 | 10200860 | *5' of WDR1* | 4.35×10^-118^ | [1] |
| *rs4697972* | 4 | 10201503 | *5' of WDR1* | 2.37×10^-14^ | [10] |
| *rs1990469* | 4 | 10201652 | *5' of WDR1* | 4.78×10^-13^ | [10] |
| *rs4697732* | 4 | 10201873 | *5' of WDR1* | 8.15×10^-121^ | [1] |
| *rs2868416* | 4 | 10202574 | *5' of WDR1* | 1.61×10^-122^ | [1] |
| *rs2868420* | 4 | 10202997 | *5' of WDR1* | 5.67×10^-14^ | [10] |
| *rs4697973* | 4 | 10203152 | *5' of WDR1* | 4.78×10^-14^ | [10] |
| *rs10489079* | 4 | 10203963 | *5' of WDR1* | 1.79×10^-135^ | [1] |
| *rs6858209* | 4 | 10204157 | *5' of WDR1* | 3.48×10^-127^ | [1] |
| *rs9283699* | 4 | 10204189 | *5' of WDR1* | 1.50×10^-127^ | [1] |
| *rs10030776* | 4 | 10204475 | *5' of WDR1* | 2.47×10^-127^ | [1] |
| *rs10030782* | 4 | 10204496 | *5' of WDR1* | 3.92×10^-128^ | [1] |
| *rs9990501* | 4 | 10204593 | *5' of WDR1* | 1.46×10^-12^ | [10] |
| *rs10939766* | 4 | 10204970 | *5' of WDR1* | 3.52×10^-9^ | [6] |
| *rs16894893* | 4 | 10205595 | *5' of WDR1* | 6.01×10^-37^ | [1] |
| *rs4697974* | 4 | 10205718 | *5' of WDR1* | 6.35×10^-14^ | [10] |
| *rs231* | 4 | 10205848 | *5' of WDR1* | 1.83×10^-132^ | [1] |
| *rs17455117* | 4 | 10206204 | *5' of WDR1* | 1.16×10^-84^ | [1] |
| *rs6449355* | 4 | 10206731 | *5' of WDR1* | 1.33×10^-134^ | [1] |
| *rs17389602* | 4 | 10206926 | *5' of WDR1* | 0.00×10^0^ | [1] |
| *rs7690319* | 4 | 10207061 | *5' of WDR1* | 1.37×10^-13^ | [10] |
| *rs10026434* | 4 | 10208128 | *5' of WDR1* | 8.27×10^-13^ | [10] |
| *rs11734623* | 4 | 10208303 | *5' of WDR1* | 5.09×10^-14^ | [10] |
| *rs6826185* | 4 | 10208656 | *5' of WDR1* | 1.13×10^-13^ | [10] |
| *rs6812780* | 4 | 10208725 | *5' of WDR1* | 3.56×10^-8^ | [6] |
| *rs6826450* | 4 | 10208778 | *5' of WDR1* | 2.34×10^-9^ | [6] |
| *rs6845818* | 4 | 10208794 | *5' of WDR1* | 5.38×10^-9^ | [6] |
| *rs6855489* | 4 | 10208888 | *5' of WDR1* | 2.35×10^-9^ | [6] |
| *rs11947517* | 4 | 10209251 | *5' of WDR1* | 2.70×10^-9^ | [6] |
| *rs4697977* | 4 | 10210653 | *5' of WDR1* | 9.30×10^-131^ | [1] |
| *rs2080072* | 4 | 10235622 | *5' of WDR1* | 7.01×10^-262^ | [1] |
| *rs11734783* | 4 | 10240663 | *5' of WDR1* | 2.82×10^-48^ | [6] |
| *rs917821* | 4 | 10241132 | *5' of WDR1* | 2.22×10^-13^ | [1] |
| *rs11727366* | 4 | 10244841 | *5' of WDR1* | 3.87×10^-148^ | [1] |
| *rs10023177* | 4 | 10244955 | *5' of WDR1* | 3.04×10^-12^ | [6] |
| *rs1978274* | 4 | 10245087 | *5' of WDR1* | 0.00×10^0^ | [1] |
| *rs7675945* | 4 | 10245340 | *5' of WDR1* | 1.37×10^-147^ | [1] |
| *rs6853056* | 4 | 10245898 | *5' of WDR1* | 6.07×10^-12^ | [6] |
| *rs10010656* | 4 | 10246327 | *5' of WDR1* | 9.50×10^-148^ | [1] |
| *rs11732042* | 4 | 10246448 | *5' of WDR1* | 4.62×10^-30^ | [1] |
| *rs4697983* | 4 | 10247248 | *5' of WDR1* | 1.17×10^-67^ | [1] |
| *rs10939801* | 4 | 10247512 | *5' of WDR1* | 3.24×10^-8^ | [6] |
| *rs11730940* | 4 | 10247893 | *5' of WDR1* | 6.14×10^-134^ | [1] |
| *rs10025702* | 4 | 10248174 | *5' of WDR1* | 4.38×10^-11^ | [6] |
| *rs2024282* | 4 | 10249688 | *5' of WDR1* | 3.31×10^-113^ | [1] |
| *rs2024281* | 4 | 10249751 | *5' of WDR1* | 2.14×10^-134^ | [1] |
| *rs6449395* | 4 | 10249877 | *5' of WDR1* | 2.42×10^-9^ | [1] |
| *rs7661555* | 4 | 10250026 | *5' of WDR1* | 9.09×10^-10^ | [6] |
| *rs17392044* | 4 | 10250340 | *5' of WDR1* | 4.26×10^-258^ | [1] |
| *rs1017124* | 4 | 10250399 | *5' of WDR1* | 1.22×10^-11^ | [6] |
| *rs12509424* | 4 | 10250503 | *5' of WDR1* | 6.60×10^-136^ | [1] |
| *rs1860896* | 4 | 10250777 | *5' of WDR1* | 6.60×10^-127^ | [1] |
| *rs1860895* | 4 | 10250779 | *5' of WDR1* | 2.76×10^-13^ | [10] |
| *rs16895216* | 4 | 10250983 | *5' of WDR1* | 5.28×10^-21^ | [1] |
| *rs11735543* | 4 | 10251652 | *5' of WDR1* | 0.00×10^0^ | [1] |
| *rs11735623* | 4 | 10251925 | *5' of WDR1* | 3.01×10^-13^ | [10] |
| *rs10029208* | 4 | 10252005 | *5' of WDR1* | 1.21×10^-150^ | [1] |
| *rs6838644* | 4 | 10252871 | *5' of WDR1* | 8.98×10^-125^ | [1] |
| *rs4522862* | 4 | 10253015 | *5' of WDR1* | 8.78×10^-151^ | [1] |
| *rs4406017* | 4 | 10253169 | *5' of WDR1* | 1.92×10-9 | [1] |
| *rs11724760* | 4 | 10254162 | *5' of WDR1* | 1.83×10^-13^ | [10] |
| *rs4697984* | 4 | 10254550 | *5' of WDR1* | 1.24×10^-150^ | [1] |
| *rs12513165* | 4 | 10256577 | *5' of WDR1* | 5.27×10^-137^ | [1] |
| *rs2192101* | 4 | 10258057 | *5' of WDR1* | 2.50×10^-150^ | [1] |
| *rs2192100* | 4 | 10258373 | *5' of WDR1* | 3.19×10^-9^ | [1] |
| *rs4697986* | 4 | 10258968 | *5' of WDR1* | 1.81×10^-138^ | [1] |
| *rs4697736* | 4 | 10259834 | *5' of WDR1* | 7.30×10^-10^ | [1] |
| *rs17406107* | 4 | 10263380 | *5' of WDR1* | 0.00×10^0^ | [1] |
| *rs4697995* | 4 | 10264276 | *5' of WDR1* | 1.16×10^-8^ | [1] |
| *rs929577* | 4 | 10264888 | *5' of WDR1* | 8.78×10^-10^ | [6] |
| *rs759031* | 4 | 10265433 | *5' of WDR1* | 1.96×10^-136^ | [1] |
| *rs17472370* | 4 | 10270131 | *5' of WDR1* | 6.52×10^-67^ | [1] |
| *rs10489076* | 4 | 10270848 | *5' of WDR1* | 6.40×10^-42^ | [1] |
| *rs13134726* | 4 | 10271119 | *5' of WDR1* | 1.49×10^-8^ | [1] |
| *rs12505222* | 4 | 10271136 | *5' of WDR1* | 1.64×10^-41^ | [1] |
| *rs7676442* | 4 | 10272591 | *5' of WDR1* | 8.88×10^-46^ | [1] |
| *rs10489074* | 4 | 10272605 | *5' of WDR1* | 1.02×10^-35^ | [1] |
| *rs10489073* | 4 | 10272788 | *5' of WDR1* | 1.20×10^-8^ | [6] |
| *rs17407324* | 4 | 10273043 | *5' of WDR1* | 1.91×10^-8^ | [6] |
| *rs10489072* | 4 | 10273244 | *5' of WDR1* | 3.79×10^-44^ | [1] |
| *rs10939814* | 4 | 10273329 | *5' of WDR1* | 9.58×10^-45^ | [1] |
| *rs10489071* | 4 | 10273549 | *5' of WDR1* | 1.83×10^-42^ | [1] |
| *rs2192095* | 4 | 10274173 | *5' of WDR1* | 1.49×10^-44^ | [1] |
| *rs4697998* | 4 | 10274626 | *5' of WDR1* | 5.53×10^-41^ | [1] |
| *rs4697999* | 4 | 10274934 | *5' of WDR1* | 3.01×10^-38^ | [1] |
| *rs17407555* | 4 | 10274994 | *5' of WDR1* | 3.78×10^-36^ | [1] |
| *rs1860911* | 4 | 10275057 | *5' of WDR1* | 4.10×10^-136^ | [1] |
| *rs1860910* | 4 | 10275470 | *5' of WDR1* | 3.25×10^-136^ | [1] |
| *rs10805364* | 4 | 10275518 | *5' of WDR1* | 1.67×10^-9^ | [12] |
| *rs6823180* | 4 | 10275831 | *5' of WDR1* | 2.36×10^-36^ | [1] |
| *rs6833142* | 4 | 10275982 | *5' of WDR1* | 1.36×10^-35^ | [1] |
| *rs10489070* | 4 | 10276352 | *5' of WDR1* | 4.18×10^-11^ | [22] |
| *rs12510549* | 4 | 10276467 | *5' of WDR1* | 5.24×10^-47^ | [6] |
| *rs4698000* | 4 | 10277467 | *5' of WDR1* | 5.81×10^-38^ | [1] |
| *rs6836916* | 4 | 10277792 | *5' of WDR1* | 3.40×10^-38^ | [1] |
| *rs11736814* | 4 | 10277869 | *5' of WDR1* | 5.01×10^-12^ | [1] |
| *rs16895836* | 4 | 10278349 | *5' of WDR1* | 4.51×10^-40^ | [1] |
| *rs7435841* | 4 | 10278552 | *5' of WDR1* | 4.51×10^-38^ | [1] |
| *rs10489069* | 4 | 10278668 | *5' of WDR1* | 1.46×10^-135^ | [1] |
| *rs10032742* | 4 | 10278893 | *5' of WDR1* | 1.51×10^-37^ | [1] |
| *rs4698001* | 4 | 10279413 | *5' of WDR1* | 1.98×10^-39^ | [1] |
| *rs1860909* | 4 | 10279557 | *5' of WDR1* | 5.16×10^-9^ | [1] |
| *rs17474174* | 4 | 10281411 | *3' of ZNF518B* | 3.34×10^-138^ | [1] |
| *rs2159868* | 4 | 10283560 | *3' of ZNF518B* | 5.56×10^-10^ | [1] |
| *rs4698005* | 4 | 10283627 | *3' of ZNF518B* | 7.21×10^-10^ | [1] |
| *rs4697740* | 4 | 10284565 | *3' of ZNF518B* | 4.90×10^-9^ | [1] |
| *rs16895984* | 4 | 10284727 | *3' of ZNF518B* | 1.69×10^-25^ | [6] |
| *rs4698009* | 4 | 10284993 | *3' of ZNF518B* | 1.25×10^-37^ | [6] |
| *rs4698014* | 4 | 10286301 | *3' of ZNF518B* | 1.67×10^-9^ | [12] |
| *rs17409460* | 4 | 10286427 | *3' of ZNF518B* | 6.25×10^-138^ | [1] |
| *rs7685241* | 4 | 10286665 | *3' of ZNF518B* | 5.12×10^-133^ | [1] |
| *rs12640013* | 4 | 10286687 | *3' of ZNF518B* | 9.55×10^-9^ | [1] |
| *rs10939818* | 4 | 10286962 | *3' of ZNF518B* | 4.92×10^-13^ | [10] |
| *rs17475334* | 4 | 10287170 | *3' of ZNF518B* | 7.16×10^-138^ | [1] |
| *rs11932349* | 4 | 10287251 | *3' of ZNF518B* | 1.68×10^-10^ | [6] |
| *rs10489068* | 4 | 10287268 | *3' of ZNF518B* | 1.42×10^-8^ | [6] |
| *rs11945358* | 4 | 10287559 | *3' of ZNF518B* | 1.14×10^-13^ | [10] |
| *rs17475461* | 4 | 10287677 | *3' of ZNF518B* | 3.89×10^-30^ | [1] |
| *rs7670709* | 4 | 10288932 | *3' of ZNF518B* | 8.53×10^-14^ | [10] |
| *rs11937220* | 4 | 10289874 | *3' of ZNF518B* | 1.11×10^-9^ | [6] |
| *rs757628* | 4 | 10290297 | *3' of ZNF518B* | 2.22×10^-13^ | [10] |
| *rs7692559* | 4 | 10290726 | *3' of ZNF518B* | 3.03×10^-30^ | [1] |
| *rs6449438* | 4 | 10291063 | *3' of ZNF518B* | 2.92×10^-31^ | [1] |
| *rs2024280* | 4 | 10291813 | *3' of ZNF518B* | 2.34×10^-46^ | [6] |
| *rs2192094* | 4 | 10291968 | *3' of ZNF518B* | 1.92×10^-138^ | [1] |
| *rs11730631* | 4 | 10292968 | *3' of ZNF518B* | 3.19×10^-47^ | [6] |
| *rs11731652* | 4 | 10292984 | *3' of ZNF518B* | 0.00×10^0^ | [1] |
| *rs917827* | 4 | 10295500 | *3' of ZNF518B* | 7.54×10^-132^ | [1] |
| *rs6839820* | 4 | 10296114 | *3' of ZNF518B* | 4.73×10^-14^ | [10] |
| *rs11728055* | 4 | 10296298 | *3' of ZNF518B* | 4.36×10^-17^ | [6] |
| *rs1860907* | 4 | 10296699 | *3' of ZNF518B* | 7.25×10^-134^ | [1] |
| *rs6856707* | 4 | 10297330 | *3' of ZNF518B* | 2.21×10^-13^ | [10] |
| *rs17410735* | 4 | 10297447 | *3' of ZNF518B* | 8.67×10^-138^ | [1] |
| *rs4698017* | 4 | 10298094 | *3' of ZNF518B* | 3.71×10^-13^ | [10] |
| *rs4697744* | 4 | 10298147 | *3' of ZNF518B* | 1.20×10^-13^ | [10] |
| *rs11732729* | 4 | 10300316 | *3' of ZNF518B* | 5.93×10^-24^ | [1] |
| *rs2080077* | 4 | 10300382 | *3' of ZNF518B* | 9.39×10^-147^ | [1] |
| *rs2098236* | 4 | 10300472 | *3' of ZNF518B* | 1.28×10^-146^ | [1] |
| *rs6834574* | 4 | 10300814 | *3' of ZNF518B* | 8.22×10^-138^ | [1] |
| *rs10939829* | 4 | 10300819 | *3' of ZNF518B* | 4.49×10^-109^ | [1] |
| *rs7683755* | 4 | 10301757 | *3' of ZNF518B* | 1.45×10^-137^ | [1] |
| *rs10014800* | 4 | 10302493 | *3' of ZNF518B* | 3.18×10^-13^ | [10] |
| *rs2868937* | 4 | 10303081 | *3' of ZNF518B* | 0.00×10^0^ | [1] |
| *rs4698023* | 4 | 10304726 | *3' of ZNF518B* | 0.00×10^0^ | [1] |
| *rs4698025* | 4 | 10305157 | *3' of ZNF518B* | 4.68×10^-141^ | [1] |
| *rs7689060* | 4 | 10305463 | *3' of ZNF518B* | 2.16×10^-35^ | [6] |
| *rs1468692* | 4 | 10305775 | *3' of ZNF518B* | 6.03×10^-29^ | [1] |
| *rs991458* | 4 | 10308756 | *3' of ZNF518B* | 6.35×10^-9^ | [1] |
| *rs9991653* | 4 | 10310495 | *3' of ZNF518B* | 1.10×10^-35^ | [1] |
| *rs7436833* | 4 | 10311074 | *3' of ZNF518B* | 2.46×10^-32^ | [1] |
| *rs6449449* | 4 | 10311707 | *3' of ZNF518B* | 1.16×10^-131^ | [1] |
| *rs6449450* | 4 | 10311887 | *3' of ZNF518B* | 9.33×10^-14^ | [10] |
| *rs12511337* | 4 | 10311972 | *3' of ZNF518B* | 5.46×10^-41^ | [6] |
| *rs6449451* | 4 | 10312073 | *3' of ZNF518B* | 1.50×10^-8^ | [6] |
| *rs6449452* | 4 | 10312112 | *3' of ZNF518B* | 9.44×10^-121^ | [1] |
| *rs4698028* | 4 | 10312216 | *3' of ZNF518B* | 2.45×10^-8^ | [6] |
| *rs4698029* | 4 | 10312798 | *3' of ZNF518B* | 1.33×10^-40^ | [6] |
| *rs2192093* | 4 | 10313106 | *3' of ZNF518B* | 1.04×10^-8^ | [6] |
| *rs6810699* | 4 | 10314025 | *3' of ZNF518B* | 1.59×10^-8^ | [6] |
| *rs727995* | 4 | 10314177 | *3' of ZNF518B* | 1.93×10^-31^ | [1] |
| *rs727996* | 4 | 10314188 | *3' of ZNF518B* | 2.75×10^-12^ | [1] |
| *rs714436* | 4 | 10314667 | *3' of ZNF518B* | 2.48×10^-8^ | [12] |
| *rs2868939* | 4 | 10314921 | *3' of ZNF518B* | 3.78×10^-25^ | [1] |
| *rs17477561* | 4 | 10315096 | *3' of ZNF518B* | 2.68×10^-8^ | [6] |
| *rs4698031* | 4 | 10315921 | *3' of ZNF518B* | 0.00×10^0^ | [1] |
| *rs6449453* | 4 | 10316834 | *3' of ZNF518B* | 3.15×10^-130^ | [1] |
| *rs17418478* | 4 | 10316851 | *3' of ZNF518B* | 0.00×10^0^ | [1] |
| *rs7666514* | 4 | 10316879 | *3' of ZNF518B* | 1.66×10^-119^ | [1] |
| *rs17418533* | 4 | 10316941 | *3' of ZNF518B* | 0.00×10^0^ | [1] |
| *rs6449454* | 4 | 10317381 | *3' of ZNF518B* | 2.36×10^-126^ | [1] |
| *rs11722185* | 4 | 10317482 | *3' of ZNF518B* | 0.00×10^0^ | [1] |
| *rs1860905* | 4 | 10318836 | *3' of ZNF518B* | 5.03×10^-142^ | [1] |
| *rs11737588* | 4 | 10319007 | *3' of ZNF518B* | 2.10×10^-31^ | [1] |
| *rs4698033* | 4 | 10319571 | *3' of ZNF518B* | 4.81×10^-142^ | [1] |
| *rs4697748* | 4 | 10319699 | *3' of ZNF518B* | 2.84×10^-142^ | [1] |
| *rs12506560* | 4 | 10322032 | *3' of ZNF518B* | 1.77×10^-10^ | [1] |
| *rs12506625* | 4 | 10322185 | *3' of ZNF518B* | 2.38×10^-10^ | [1] |
| *rs993173* | 4 | 10323935 | *3' of ZNF518B* | 1.34×10^-165^ | [1] |
| *rs9291683* | 4 | 10324160 | *3' of ZNF518B* | 2.74×10^-8^ | [10] |
| *rs17478453* | 4 | 10324312 | *3' of ZNF518B* | 2.84×10^-9^ | [6] |
| *rs993172* | 4 | 10324361 | *3' of ZNF518B* | 2.94×10^-13^ | [6] |
| *rs1558489* | 4 | 10325489 | *3' of ZNF518B* | 4.75×10^-141^ | [1] |
| *rs1558488* | 4 | 10327414 | *3' of ZNF518B* | 1.25×10^-38^ | [1] |
| *rs4698036* | 4 | 10331294 | *3' of ZNF518B* | 2.89×10^-9^ | [12] |
| *rs11729318* | 4 | 10336919 | *3' of ZNF518B* | 0.00×10^0^ | [1] |
| *rs17419612* | 4 | 10337263 | *3' of ZNF518B* | 1.43×10^-124^ | [1] |
| *rs1964268* | 4 | 10337921 | *3' of ZNF518B* | 1.21×10^-142^ | [1] |
| *rs13142790* | 4 | 10339040 | *3' of ZNF518B* | 2.19×10^-124^ | [1] |
| *rs759024* | 4 | 10339095 | *3' of ZNF518B* | 4.59×10^-145^ | [1] |
| *rs759022* | 4 | 10339426 | *3' of ZNF518B* | 4.49×10^-11^ | [1] |
| *rs10489067* | 4 | 10340486 | *3' of ZNF518B* | 7.41×10^-11^ | [1] |
| *rs2007103* | 4 | 10341255 | *3' of ZNF518B* | 1.04×10^-145^ | [1] |
| *rs4306950* | 4 | 10341419 | *3' of ZNF518B* | 6.36×10^-11^ | [6] |
| *rs10938761* | 4 | 10342723 | *3' of ZNF518B* | 5.21×10^-11^ | [1] |
| *rs984723* | 4 | 10344624 | *3' of ZNF518B* | 1.44×10^-10^ | [6] |
| *rs2052165* | 4 | 10345172 | *3' of ZNF518B* | 7.27×10^-31^ | [1] |
| *rs1035052* | 4 | 10345339 | *3' of ZNF518B* | 7.94×10^-12^ | [1] |
| *rs17420080* | 4 | 10345548 | *3' of ZNF518B* | 3.53×10^-8^ | [12] |
| *rs13109847* | 4 | 10346245 | *3' of ZNF518B* | 2.45×10^-8^ | [6] |
| *rs17479487* | 4 | 10346550 | *3' of ZNF518B* | 2.42×10^-127^ | [1] |
| *rs2192084* | 4 | 10347051 | *3' of ZNF518B* | 1.73×10^-135^ | [1] |
| *rs16897170* | 4 | 10347414 | *3' of ZNF518B* | 8.21×10^-11^ | [1] |
| *rs2192083* | 4 | 10347764 | *3' of ZNF518B* | 1.69×10^-10^ | [1] |
| *rs6849037* | 4 | 10348431 | *3' of ZNF518B* | 3.89×10^-150^ | [1] |
| *rs4698037* | 4 | 10348895 | *3' of ZNF518B* | 1.08×10^-11^ | [6] |
| *rs1544599* | 4 | 10349168 | *3' of ZNF518B* | 1.38×10^-13^ | [10] |
| *rs17420450* | 4 | 10350765 | *3' of ZNF518B* | 1.09×10^-86^ | [1] |
| *rs13145430* | 4 | 10350948 | *3' of ZNF518B* | 3.23×10^-8^ | [6] |
| *rs17420513* | 4 | 10350975 | *3' of ZNF518B* | 2.23×10^-137^ | [1] |
| *rs17420562* | 4 | 10351595 | *3' of ZNF518B* | 2.28×10^-135^ | [1] |
| *rs4697750* | 4 | 10351706 | *3' of ZNF518B* | 2.12×10^-149^ | [1] |
| *rs6853659* | 4 | 10351970 | *3' of ZNF518B* | 2.57×10^-138^ | [1] |
| *rs4697751* | 4 | 10352506 | *3' of ZNF518B* | 4.05×10^-141^ | [1] |
| *rs4698040* | 4 | 10352550 | *3' of ZNF518B* | 0.00×10^0^ | [1] |
| *rs4697752* | 4 | 10353381 | *3' of ZNF518B* | 5.51×10^-150^ | [1] |
| *rs2098235* | 4 | 10354069 | *3' of ZNF518B* | 2.26×10^-11^ | [1] |
| *rs10938768* | 4 | 10354341 | *3' of ZNF518B* | 1.36×10^-138^ | [1] |
| *rs6838846* | 4 | 10355529 | *3' of ZNF518B* | 3.97×10^-102^ | [1] |
| *rs11931317* | 4 | 10356348 | *3' of ZNF518B* | 3.72×10^-150^ | [1] |
| *rs1860904* | 4 | 10357448 | *3' of ZNF518B* | 1.57×10^-11^ | [6] |
| *rs7680825* | 4 | 10358790 | *3' of ZNF518B* | 5.10×10^-150^ | [1] |
| *rs7681212* | 4 | 10358964 | *3' of ZNF518B* | 4.96×10^-31^ | [6] |
| *rs13114042* | 4 | 10359104 | *3' of ZNF518B* | 6.58×10^-10^ | [1] |
| *rs7661209* | 4 | 10359607 | *3' of ZNF518B* | 1.28×10^-125^ | [1] |
| *rs11727261* | 4 | 10363482 | *3' of ZNF518B* | 8.33×10^-10^ | [1] |
| *rs4235361* | 4 | 10365093 | *3' of ZNF518B* | 8.38×10^-10^ | [1] |
| *rs4697753* | 4 | 10366453 | *3' of ZNF518B* | 5.50×10^-70^ | [1] |
| *rs4698041* | 4 | 10368235 | *3' of ZNF518B* | 5.21×10^-150^ | [1] |
| *rs9291406* | 4 | 10369564 | *3' of ZNF518B* | 6.34×10^-10^ | [1] |
| *rs13122923* | 4 | 10369776 | *3' of ZNF518B* | 1.30×10^-9^ | [1] |
| *rs6857135* | 4 | 10370585 | *3' of ZNF518B* | 9.93×10^-151^ | [1] |
| *rs6813712* | 4 | 10370663 | *3' of ZNF518B* | 1.05×10^-150^ | [1] |
| *rs6813919* | 4 | 10370791 | *3' of ZNF518B* | 1.24×10^-9^ | [1] |
| *rs6849583* | 4 | 10372790 | *3' of ZNF518B* | 1.06×10^-150^ | [1] |
| *rs13125564* | 4 | 10372914 | *3' of ZNF518B* | 1.50×10^-9^ | [1] |
| *rs6851536* | 4 | 10372966 | *3' of ZNF518B* | 8.71×10^-151^ | [1] |
| *rs11943393* | 4 | 10374005 | *3' of ZNF518B* | 2.08×10^-150^ | [1] |
| *rs10938772* | 4 | 10375328 | *3' of ZNF518B* | 0.00×10^0^ | [1] |
| *rs11732092* | 4 | 10377405 | *3' of ZNF518B* | 1.31×10^-13^ | [10] |
| *rs4698043* | 4 | 10378602 | *3' of ZNF518B* | 2.07×10^-151^ | [1] |
| *rs7677806* | 4 | 10383005 | *3' of ZNF518B* | 1.18×10^-11^ | [6] |
| *rs59420943* | 4 | 10384278 | *3' of ZNF518B* | 8.02×10^-15^ | [10] |
| *rs4302456* | 4 | 10386674 | *3' of ZNF518B* | 1.92×10^-137^ | [1] |
| *rs4302457* | 4 | 10386973 | *3' of ZNF518B* | 1.57×10^-9^ | [6] |
| *rs9990427* | 4 | 10388313 | *3' of ZNF518B* | 3.63×10^-14^ | [10] |
| *rs9990701* | 4 | 10388610 | *3' of ZNF518B* | 3.55×10^-13^ | [10] |
| *rs11724536* | 4 | 10390289 | *3' of ZNF518B* | 1.47×10^-9^ | [1] |
| *rs4607209* | 4 | 10400156 | *3' of ZNF518B* | 4.76×10^-20^ | [1] |
| *rs10017305* | 4 | 10401223 | *3' of ZNF518B* | 0.00×10^0^ | [1] |
| *rs11943276* | 4 | 10403545 | *3' of ZNF518B* | 3.45×10^-29^ | [6] |
| *rs7654258* | 4 | 10404512 | *3' of ZNF518B* | 1.09×10^-19^ | [1] |
| *rs7677318* | 4 | 10406291 | *3' of ZNF518B* | 1.88×10^-9^ | [1] |
| *rs4463062* | 4 | 10406994 | *3' of ZNF518B* | 6.02×10^-9^ | [6] |
| *rs6819959* | 4 | 10407534 | *3' of ZNF518B* | 4.74×10^-8^ | [6] |
| *rs4643800* | 4 | 10407572 | *3' of ZNF518B* | 5.93×10^-278^ | [1] |
| *rs11728025* | 4 | 10408221 | *3' of ZNF518B* | 1.57×10^-247^ | [1] |
| *rs7697246* | 4 | 10408757 | *3' of ZNF518B* | 2.79×10^-131^ | [1] |
| *rs4698049* | 4 | 10409770 | *3' of ZNF518B* | 2.69×10^-131^ | [1] |
| *rs7656072* | 4 | 10409841 | *3' of ZNF518B* | 9.26×10^-31^ | [1] |
| *rs4698050* | 4 | 10410748 | *3' of ZNF518B* | 2.05×10^-8^ | [12] |
| *rs11721485* | 4 | 10412554 | *3' of ZNF518B* | 4.10×10^-10^ | [1] |
| *rs4610325* | 4 | 10413168 | *3' of ZNF518B* | 5.54×10^-131^ | [1] |
| *rs7661365* | 4 | 10413908 | *3' of ZNF518B* | 5.70×10^-9^ | [1] |
| *rs11736389* | 4 | 10416360 | *3' of ZNF518B* | 0.00×10^0^ | [1] |
| *rs13125855* | 4 | 10418078 | *3' of ZNF518B* | 6.86×10^-57^ | [1] |
| *rs6858510* | 4 | 10432508 | *3' of ZNF518B* | 2.88×10^-16^ | [1] |
| *rs7691990* | 4 | 10434459 | *3' of ZNF518B* | 1.46×10^-43^ | [1] |
| *rs10003864* | 4 | 10437391 | *3' of ZNF518B* | 7.08×10^-27^ | [1] |
| *rs4422413* | 4 | 10443384 | *ZNF518B* | 6.76×10^-36^ | [1] |
| *rs10938799* | 4 | 10443425 | *ZNF518B* | 1.97×10^-54^ | [1] |
| *rs3217* | 4 | 10444650 | *ZNF518B* | 4.20×10^-36^ | [1] |
| *rs10016022* | 4 | 10446906 | *ZNF518B* | 1.96×10^-8^ | [11] |
| *rs66538112* | 4 | 10447168 | *ZNF518B* | 1.27×10^-8^ | [11] |
| *rs10016702* | 4 | 10447640 | *ZNF518B* | 1.38×10^-12^ | [1] |
| *rs7674156* | 4 | 10452763 | *ZNF518B* | 3.29×10^-35^ | [1] |
| *rs12019277* | 4 | 10454994 | *ZNF518B* | 2.91×10^-35^ | [1] |
| *rs9996284* | 4 | 10469446 | *5' of ZNF518B* | 3.80×10^-35^ | [1] |
| *rs10029818* | 4 | 10472670 | *5' of ZNF518B* | 3.23×10^-36^ | [1] |
| *rs11732503* | 4 | 10482614 | *3' of CLNK* | 2.52×10^-51^ | [1] |
| *rs11737650* | 4 | 10485014 | *3' of CLNK* | 2.83×10^-44^ | [1] |
| *rs11733306* | 4 | 10488402 | *CLNK* | 2.98×10^-44^ | [1] |
| *rs11737601* | 4 | 10488568 | *CLNK* | 1.45×10^-60^ | [1] |
| *rs4541501* | 4 | 10488621 | *CLNK* | 4.45×10^-42^ | [1] |
| *rs13111270* | 4 | 10489007 | *CLNK* | 2.50×10^-36^ | [1] |
| *rs13130674* | 4 | 10489600 | *CLNK* | 4.55×10^-44^ | [1] |
| *rs7692088* | 4 | 10491040 | *CLNK* | 1.70×10^-48^ | [1] |
| *rs9790491* | 4 | 10493115 | *CLNK* | 1.33×10^-44^ | [1] |
| *rs13115661* | 4 | 10493961 | *CLNK* | 1.13×10^-25^ | [1] |
| *rs3749558* | 4 | 10494003 | *CLNK* | 2.83×10^-44^ | [1] |
| *rs7667644* | 4 | 10495072 | *CLNK* | 1.56×10^-44^ | [1] |
| *rs16869060* | 4 | 10495790 | *CLNK* | 2.05×10^-50^ | [1] |
| *rs13142053* | 4 | 10496241 | *CLNK* | 5.50×10^-34^ | [1] |
| *rs10033825* | 4 | 10496792 | *CLNK* | 1.63×10^-35^ | [1] |
| *rs7698826* | 4 | 10497423 | *CLNK* | 1.36×10^-46^ | [1] |
| *rs6819820* | 4 | 10498830 | *CLNK* | 1.54×10^-23^ | [1] |
| *rs12504795* | 4 | 10499344 | *CLNK* | 6.37×10^-57^ | [1] |
| *rs17467273* | 4 | 10500431 | *CLNK* | 3.28×10^-35^ | [1] |
| *rs13109939* | 4 | 10501625 | *CLNK* | 4.95×10^-45^ | [1] |
| *rs10488946* | 4 | 10502650 | *CLNK* | 6.89×10^-14^ | [1] |
| *rs6833095* | 4 | 10506599 | *CLNK* | 8.04×10^-35^ | [1] |
| *rs11734599* | 4 | 10507490 | *CLNK* | 2.11×10^-45^ | [1] |
| *rs887112* | 4 | 10507984 | *CLNK* | 4.57×10^-14^ | [1] |
| *rs13141385* | 4 | 10508404 | *CLNK* | 1.60×10^-35^ | [1] |
| *rs2868941* | 4 | 10509699 | *CLNK* | 3.19×10^-58^ | [1] |
| *rs11929850* | 4 | 10510050 | *CLNK* | 2.75×10^-14^ | [1] |
| *rs13108998* | 4 | 10510869 | *CLNK* | 9.03×10^-32^ | [1] |
| *rs13109005* | 4 | 10510881 | *CLNK* | 3.84×10^-19^ | [1] |
| *rs1004327* | 4 | 10511483 | *CLNK* | 6.88×10^-42^ | [1] |
| *rs12508358* | 4 | 10512238 | *CLNK* | 2.74×10^-42^ | [1] |
| *rs13125670* | 4 | 10513072 | *CLNK* | 5.47×10^-56^ | [1] |
| *rs12499142* | 4 | 10514238 | *CLNK* | 2.59×10^-37^ | [1] |
| *rs2868942* | 4 | 10514568 | *CLNK* | 1.92×10^-41^ | [1] |
| *rs16869379* | 4 | 10514681 | *CLNK* | 1.61×10^-51^ | [1] |
| *rs10034180* | 4 | 10518525 | *CLNK* | 2.15×10^-33^ | [1] |
| *rs16869430* | 4 | 10519426 | *CLNK* | 6.36×10^-55^ | [1] |
| *rs2041215* | 4 | 10519982 | *CLNK* | 3.20×10^-34^ | [1] |
| *rs7665423* | 4 | 10520600 | *CLNK* | 1.39×10^-12^ | [1] |
| *rs13125086* | 4 | 10520693 | *CLNK* | 8.07×10^-24^ | [1] |
| *rs16869474* | 4 | 10520972 | *CLNK* | 3.77×10^-55^ | [1] |
| *rs2012249* | 4 | 10521158 | *CLNK* | 2.03×10^-27^ | [1] |
| *rs2012237* | 4 | 10521221 | *CLNK* | 1.56×10^-12^ | [1] |
| *rs10001632* | 4 | 10521871 | *CLNK* | 5.58×10^-18^ | [1] |
| *rs2041216* | 4 | 10523090 | *CLNK* | 2.29×10^-34^ | [1] |
| *rs997219* | 4 | 10524671 | *CLNK* | 4.02×10^-34^ | [1] |
| *rs2108878* | 4 | 10527342 | *CLNK* | 5.48×10^-67^ | [1] |
| *rs12641877* | 4 | 10528226 | *CLNK* | 1.59×10^-66^ | [1] |
| *rs2286463* | 4 | 10532258 | *CLNK* | 3.04×10^-47^ | [1] |
| *rs2286465* | 4 | 10532634 | *CLNK* | 3.65×10^-56^ | [1] |
| *rs10030521* | 4 | 10536417 | *CLNK* | 1.70×10^-17^ | [1] |
| *rs10488948* | 4 | 10536544 | *CLNK* | 8.02×10^-41^ | [1] |
| *rs2108879* | 4 | 10538144 | *CLNK* | 3.70×10^-16^ | [1] |
| *rs7688620* | 4 | 10543318 | *CLNK* | 9.96×10^-18^ | [1] |
| *rs6856070* | 4 | 10545768 | *CLNK* | 2.07×10^-17^ | [1] |
| *rs11939512* | 4 | 10546359 | *CLNK* | 1.17×10^-17^ | [1] |
| *rs10938845* | 4 | 10551500 | *CLNK* | 6.20×10^-25^ | [1] |
| *rs1974584* | 4 | 10557861 | *CLNK* | 2.60×10^-22^ | [1] |
| *rs1558201* | 4 | 10557918 | *CLNK* | 8.09×10^-26^ | [1] |
| *rs4698068* | 4 | 10558992 | *CLNK* | 3.36×10^-9^ | [1] |
| *rs4698069* | 4 | 10559041 | *CLNK* | 3.85×10^-14^ | [1] |
| *rs9994202* | 4 | 10559396 | *CLNK* | 3.09×10^-13^ | [1] |
| *rs12643937* | 4 | 10577980 | *CLNK* | 3.95×10^-11^ | [1] |
| *rs12640119* | 4 | 10586384 | *CLNK* | 1.11×10^-10^ | [1] |
| *rs10488949* | 4 | 10591448 | *CLNK* | 8.29×10^-11^ | [1] |
| *rs2531178* | 4 | 10601134 | *CLNK* | 2.72×10^-16^ | [1] |
| *rs2531185* | 4 | 10605821 | *CLNK* | 2.26×10^-9^ | [1] |
| *rs6836007* | 4 | 10647584 | *CLNK* | 5.00×10^-10^ | [1] |
| *rs4593124* | 4 | 10648278 | *CLNK* | 5.97×10^-11^ | [1] |
| *rs12650571* | 4 | 10649456 | *CLNK* | 5.19×10^-10^ | [1] |
| *rs7696539* | 4 | 10649778 | *CLNK* | 4.79×10^-10^ | [1] |
| *rs10516207* | 4 | 10650693 | *CLNK* | 4.07×10^-9^ | [1] |
| *rs13107947* | 4 | 10651617 | *CLNK* | 2.10×10^-12^ | [1] |
| *rs13139842* | 4 | 10651701 | *CLNK* | 6.81×10^-12^ | [1] |
| *rs6822578* | 4 | 10652262 | *CLNK* | 2.55×10^-11^ | [1] |
| *rs4449408* | 4 | 10652413 | *CLNK* | 7.15×10^-12^ | [1] |
| *rs12233843* | 4 | 10655218 | *CLNK* | 1.12×10^-10^ | [1] |
| *rs10938919* | 4 | 10655503 | *CLNK* | 6.92×10^-13^ | [1] |
| *rs9683460* | 4 | 10656446 | *CLNK* | 6.78×10^-11^ | [1] |
| *rs6830367* | 4 | 10671323 | *CLNK* | 2.35×10^-11^ | [1] |
| *rs141646361* | 4 | 77676155 | *SHROOM3* | 2.00×10^-16^ | [4] |
| *rs17013187* | 4 | 88733531 | *3' of IBSP* | 2.85×10^-12^ | [21] |
| *rs4466013* | 4 | 88760413 | *MEPE* | 1.60×10^-16^ | [1] |
| *rs17013282* | 4 | 88765873 | *MEPE* | 4.95×10^-17^ | [21] |
| *rs6854498* | 4 | 88776760 | *3' of MEPE* | 1.11×10^-16^ | [1] |
| *rs6854361* | 4 | 88776864 | *3' of MEPE* | 5.56×10^-17^ | [1] |
| *rs2169612* | 4 | 88817414 | *3' of MEPE* | 4.84×10^-10^ | [1] |
| *rs12651696* | 4 | 88821873 | *3' of MEPE* | 2.49×10^-9^ | [1] |
| *rs17013544* | 4 | 88823643 | *3' of MEPE* | 1.15×10^-10^ | [1] |
| *rs17013545* | 4 | 88826327 | *3' of MEPE* | 3.73×10^-9^ | [1] |
| *rs716202* | 4 | 88836002 | *5' of SPP1* | 1.13×10^-8^ | [1] |
| *rs17842205* | 4 | 88849552 | *5' of SPP1* | 1.45×10^-30^ | [1] |
| *rs10516796* | 4 | 88856733 | *5' of SPP1* | 2.35×10^-8^ | [1] |
| *rs4693920* | 4 | 88864775 | *5' of SPP1* | 6.69×10^-31^ | [1] |
| *rs2728123* | 4 | 88870795 | *5' of SPP1* | 1.17×10^-31^ | [1] |
| *rs17013584* | 4 | 88872963 | *5' of SPP1* | 8.00×10^-30^ | [1] |
| *rs2728119* | 4 | 88877531 | *5' of SPP1* | 2.29×10^-40^ | [1] |
| *rs4490426* | 4 | 88880522 | *5' of SPP1* | 9.01×10^-15^ | [1] |
| *rs10516798* | 4 | 88883537 | *5' of SPP1* | 1.64×10^-14^ | [1] |
| *rs11730059* | 4 | 88887327 | *5' of SPP1* | 1.00×10^-46^ | [1] |
| *rs12641001* | 4 | 88888940 | *5' of SPP1* | 8.14×10^-14^ | [1] |
| *rs10516800* | 4 | 88891590 | *5' of SPP1* | 1.19×10^-13^ | [1] |
| *rs6813526* | 4 | 88894235 | *5' of SPP1* | 2.99×10^-15^ | [1] |
| *rs2853749* | 4 | 88897814 | *SPP1* | 1.21×10^-15^ | [1] |
| *rs7685225* | 4 | 88906458 | *3' of SPP1* | 1.76×10^-12^ | [1] |
| *rs6818927* | 4 | 88907257 | *3' of SPP1* | 7.90×10^-13^ | [1] |
| *rs4128340* | 4 | 88908136 | *3' of SPP1* | 5.29×10^-13^ | [1] |
| *rs6532041* | 4 | 88909033 | *3' of SPP1* | 4.66×10^-12^ | [1] |
| *rs6832511* | 4 | 88909088 | *3' of SPP1* | 3.92×10^-13^ | [1] |
| *rs6838095* | 4 | 88909436 | *3' of SPP1* | 4.40×10-^13^ | [1] |
| *rs12509864* | 4 | 88909766 | *3' of SPP1* | 5.44×10^-11^ | [1] |
| *rs11938988* | 4 | 88910113 | *3' of SPP1* | 2.01×10^-13^ | [1] |
| *rs2728116* | 4 | 88931050 | *PKD2* | 4.67×10^-8^ | [1] |
| *rs2725234* | 4 | 88932272 | *PKD2* | 2.20×10^-43^ | [1] |
| *rs12503776* | 4 | 88932464 | *PKD2* | 1.92×10^-10^ | [1] |
| *rs2728113* | 4 | 88939736 | *PKD2* | 3.65×10^-8^ | [6] |
| *rs2725227* | 4 | 88944511 | *PKD2* | 7.44×10^-12^ | [1] |
| *rs2467052* | 4 | 88949175 | *PKD2* | 2.27×10^-11^ | [1] |
| *rs13149278* | 4 | 88951390 | *PKD2* | 5.76×10^-11^ | [1] |
| *rs2725225* | 4 | 88952076 | *PKD2* | 1.01×10^-11^ | [1] |
| *rs2728110* | 4 | 88952883 | *PKD2* | 5.53×10^-12^ | [1] |
| *rs17786456* | 4 | 88957562 | *PKD2* | 9.57×10^-10^ | [1] |
| *rs2728109* | 4 | 88957723 | *PKD2* | 1.66×10^-8^ | [6] |
| *rs2725220* | 4 | 88959922 | *PKD2* | 9.40×10^-27^ | [10] |
| *rs7696304* | 4 | 88959998 | *PKD2* | 6.13×10^-9^ | [1] |
| *rs2725217* | 4 | 88960258 | *PKD2* | 1.56×10^-61^ | [1] |
| *rs2725215* | 4 | 88961571 | *PKD2* | 3.28×10^-9^ | [6] |
| *rs2725212* | 4 | 88968713 | *PKD2* | 3.45×10^-53^ | [1] |
| *rs2725211* | 4 | 88970375 | *PKD2* | 3.18×10^-41^ | [21] |
| *rs2728106* | 4 | 88972051 | *PKD2* | 7.11×10^-51^ | [1] |
| *rs2728104* | 4 | 88973006 | *PKD2* | 9.01×10^-57^ | [1] |
| *rs2725210* | 4 | 88973427 | *PKD2* | 2.34×10^-47^ | [1] |
| *rs2728099* | 4 | 88975738 | *PKD2* | 6.03×10^-10^ | [6] |
| *rs2725207* | 4 | 88979529 | *PKD2* | 1.66×10^-47^ | [1] |
| *rs2728133* | 4 | 88981690 | *PKD2* | 3.10×10^-46^ | [1] |
| *rs2728132* | 4 | 88982461 | *PKD2* | 1.33×10^-46^ | [1] |
| *rs2725205* | 4 | 88985711 | *PKD2* | 4.85×10^-46^ | [1] |
| *rs11938025* | 4 | 88987276 | *PKD2* | 7.74×10^-10^ | [1] |
| *rs4336187* | 4 | 88987916 | *PKD2* | 1.44×10^-46^ | [1] |
| *rs2725203* | 4 | 88995795 | *PKD2* | 8.23×10^-47^ | [1] |
| *rs2728121* | 4 | 88997102 | *PKD2* | 6.95×10^-44^ | [1] |
| *rs10965* | 4 | 88998083 | *PKD2* | 1.54×10^-9^ | [1] |
| *rs2728126* | 4 | 88999222 | *3' of PKD2* | 3.46×10^-49^ | [1] |
| *rs2725201* | 4 | 88999306 | *3' of PKD2* | 2.95×10^-8^ | [10] |
| *rs2728125* | 4 | 89001893 | *3' of PKD2* | 1.00×10^-10^ | [6] |
| *rs199897813* | 4 | 89015728 | *ABCG2* | 2.00×10^-16^ | [4] |
| *rs2231164* | 4 | 89015857 | *ABCG2* | 1.98×10^-12^ | [10] |
| *rs2231156* | 4 | 89020427 | *ABCG2* | 7.33×10^-61^ | [1] |
| *rs4148157* | 4 | 89020934 | *ABCG2* | 6.64×10^-30^ | [10] |
| *rs4693924* | 4 | 89023224 | *ABCG2* | 8.51×10^-63^ | [1] |
| *rs2231148* | 4 | 89028478 | *ABCG2* | 2.27×10^-12^ | [1] |
| *rs2054576* | 4 | 89028775 | *ABCG2* | 5.08×10^-13^ | [23] |
| *rs12505410* | 4 | 89030841 | *ABCG2* | 3.21×10^-29^ | [1] |
| *rs2622621* | 4 | 89030920 | *ABCG2* | 7.41×10^-55^ | [1] |
| *rs13120400* | 4 | 89033527 | *ABCG2* | 3.19×10^-33^ | [1] |
| *rs1481012* | 4 | 89039082 | *ABCG2* | 1.13×10^-21^ | [6] |
| *rs2199936* | 4 | 89045331 | *ABCG2* | 2.32×10^-25^ | [8] |
| *rs2231142* | 4 | 89052323 | *ABCG2* | 1.00×10^-18^ | [16] |
| *rs4148155* | 4 | 89054667 | *ABCG2* | 1.50×10^-25^ | [3] |
| *rs4148152* | 4 | 89060909 | *ABCG2* | 2.95×10^-18^ | [3] |
| *rs13137622* | 4 | 89062513 | *ABCG2* | 8.49×10^-27^ | [1] |
| *rs3114018* | 4 | 89064581 | *ABCG2* | 2.93×10^-12^ | [8] |
| *rs3109823* | 4 | 89064602 | *ABCG2* | 9.30×10^-12^ | [10] |
| *rs6857600* | 4 | 89066075 | *ABCG2* | 8.83×10^-12^ | [1] |
| *rs2622626* | 4 | 89066715 | *ABCG2* | 7.71×10^-54^ | [1] |
| *rs6532049* | 4 | 89067526 | *ABCG2* | 8.40×10^-11^ | [1] |
| *rs17731799* | 4 | 89068455 | *ABCG2* | 1.77×10^-14^ | [10] |
| *rs2622624* | 4 | 89069406 | *ABCG2* | 7.78×10^-62^ | [1] |
| *rs2622604* | 4 | 89078924 | *ABCG2* | 7.07×10^-11^ | [10] |
| *rs2622605* | 4 | 89079386 | *ABCG2* | 3.92×10^-64^ | [1] |
| *rs3114020* | 4 | 89083666 | *ABCG2* | 2.33×10^-13^ | [10] |
| *rs2622609* | 4 | 89088475 | *ABCG2* | 1.03×10^-9^ | [6] |
| *rs10011796* | 4 | 89090877 | *ABCG2* | 2.43×10^-51^ | [1] |
| *rs10009618* | 4 | 89094008 | *ABCG2* | 1.98×10^-51^ | [1] |
| *rs2622629* | 4 | 89094064 | *ABCG2* | 2.19×10^-15^ | [1] |
| *rs1481014* | 4 | 89097151 | *ABCG2* | 4.62×10^-8^ | [1] |
| *rs1481017* | 4 | 89097477 | *ABCG2* | 2.67×10^-9^ | [6] |
| *rs11724427* | 4 | 89103172 | *ABCG2* | 8.89×10^-24^ | [1] |
| *rs6821227* | 4 | 89104688 | *ABCG2* | 3.86×10^-19^ | [1] |
| *rs6821239* | 4 | 89104751 | *ABCG2* | 1.96×10^-17^ | [1] |
| *rs9784454* | 4 | 89107490 | *ABCG2* | 4.42×10^-16^ | [1] |
| *rs6532053* | 4 | 89109259 | *ABCG2* | 1.26×10^-20^ | [1] |
| *rs2127863* | 4 | 89111468 | *ABCG2* | 3.38×10^-19^ | [1] |
| *rs4560364* | 4 | 89116178 | *ABCG2* | 6.95×10^-21^ | [1] |
| *rs13108900* | 4 | 89116702 | *ABCG2* | 9.95×10^-21^ | [1] |
| *rs10856870* | 4 | 89119659 | *ABCG2* | 3.00×10^-16^ | [1] |
| *rs4693930* | 4 | 89122833 | *ABCG2* | 4.94×10^-22^ | [1] |
| *rs10023457* | 4 | 89134704 | *ABCG2* | 3.06×10^-19^ | [1] |
| *rs6819328* | 4 | 89137318 | *ABCG2* | 9.65×10^-16^ | [1] |
| *rs2869736* | 4 | 89138377 | *ABCG2* | 1.72×10^-11^ | [1] |
| *rs4693935* | 4 | 89139275 | *ABCG2* | 1.08×10^-15^ | [1] |
| *rs2904185* | 4 | 89139832 | *ABCG2* | 3.91×10^-15^ | [1] |
| *rs4491984* | 4 | 89140356 | *ABCG2* | 3.96×10^-18^ | [1] |
| *rs1904903* | 4 | 89145274 | *ABCG2* | 2.86×10^-20^ | [1] |
| *rs9307048* | 4 | 89149570 | *ABCG2* | 5.64×10^-19^ | [1] |
| *rs10032109* | 4 | 89152063 | *ABCG2* | 1.04×10^-14^ | [1] |
| *rs4693941* | 4 | 89156899 | *5' of ABCG2* | 1.01×10^-19^ | [1] |
| *rs1114568* | 4 | 89159819 | *5' of ABCG2* | 3.09×10^-14^ | [1] |
| *rs13120254* | 4 | 89160561 | *5' of ABCG2* | 4.54×10^-21^ | [1] |
| *rs13120819* | 4 | 89160677 | *5' of ABCG2* | 5.28×10^-20^ | [1] |
| *rs6532058* | 4 | 89161902 | *5' of ABCG2* | 1.51×10^-8^ | [1] |
| *rs997630* | 4 | 89163853 | *5' of ABCG2* | 1.31×10^-18^ | [1] |
| *rs4693942* | 4 | 89165192 | *5' of ABCG2* | 3.90×10^-19^ | [1] |
| *rs6532061* | 4 | 89165613 | *5' of ABCG2* | 8.13×10^-17^ | [1] |
| *rs17013965* | 4 | 89170730 | *3' of PPM1K* | 3.43×10^-11^ | [1] |
| *rs11729997* | 4 | 89171059 | *3' of PPM1K* | 5.03×10^-13^ | [1] |
| *rs6853928* | 4 | 89172568 | *3' of PPM1K* | 8.31×10^-14^ | [1] |
| *rs4693946* | 4 | 89219705 | *RP11-10L7.1* | 1.99×10^-9^ | [1] |
| *rs17013995* | 4 | 89227094 | *RP11-10L7.1* | 1.47×10^-9^ | [1] |
| *rs7676986* | 4 | 89227673 | *RP11-10L7.1* | 1.56×10^-9^ | [1] |
| *rs1545207* | 4 | 89239492 | *RP11-10L7.1* | 3.75×10^-8^ | [1] |
| *rs17014018* | 4 | 89248035 | *RP11-10L7.1* | 9.69×10^-14^ | [1] |
| *rs4693211* | 4 | 89249061 | *RP11-10L7.1* | 1.67×10^-13^ | [1] |
| *rs4693950* | 4 | 89249444 | *RP11-10L7.1* | 3.74×10^-8^ | [1] |
| *rs12512051* | 4 | 89252068 | *RP11-10L7.1* | 2.13×10^-12^ | [1] |
| *rs151134704* | 4 | 103537592 | *NFKB1* | 2.00×10^-16^ | [4] |
| *rs75869162* | 5 | 16617922 | *CTC-461F20.1* | 1.57×10^-8^ | [24] |
| *rs151305324* | 5 | 72419433 | *TMEM171* | 2.00×10^-16^ | [4] |
| *rs17632159* | 5 | 72431482 | *RP11-232L2.2* | 3.52×10^-11^ | [1] |
| *rs575416* | 5 | 72437534 | *RP11-232L2.2* | 3.63×10^-9^ | [1] |
| *rs622704* | 5 | 72439029 | *RP11-232L2.2* | 9.15×10^-9^ | [1] |
| *rs636266* | 5 | 72439774 | *RP11-232L2.2* | 6.88×10^-9^ | [1] |
| *rs484573* | 5 | 72441107 | *RP11-232L2.2* | 9.80×10^-9^ | [1] |
| *rs587189* | 5 | 72441476 | *RP11-232L2.2* | 9.03×10^-9^ | [1] |
| *rs527511* | 5 | 72446334 | *RP11-232L2.2* | 5.55×10^-9^ | [1] |
| *rs522152* | 5 | 72452355 | *5' of AC116345.1* | 9.63×10^-9^ | [1] |
| *rs814174* | 6 | 7025868 | *3' of snoU13* | 1.06×10^-9^ | [1] |
| *rs3863225* | 6 | 7032317 | *3' of snoU13* | 8.64×10^-11^ | [1] |
| *rs9505008* | 6 | 7033860 | *3' of snoU13* | 7.75×10^-11^ | [1] |
| *rs13212734* | 6 | 7037637 | *3' of snoU13* | 1.06×10^-10^ | [1] |
| *rs10458103* | 6 | 7037738 | *3' of snoU13* | 6.87×10^-11^ | [1] |
| *rs13202703* | 6 | 7038265 | *3' of snoU13* | 8.09×10^-11^ | [1] |
| *rs13207230* | 6 | 7041700 | *5' of snoU13* | 3.35×10^-11^ | [1] |
| *rs17584570* | 6 | 7044167 | *5' of snoU13* | 7.72×10^-12^ | [1] |
| *rs1286005* | 6 | 7046452 | *5' of snoU13* | 1.87×10^-13^ | [1] |
| *rs6929119* | 6 | 7046594 | *5' of snoU13* | 1.58×10^-12^ | [1] |
| *rs9328398* | 6 | 7046763 | *5' of snoU13* | 1.01×10^-11^ | [1] |
| *rs1286003* | 6 | 7046998 | *5' of snoU13* | 1.80×10^-9^ | [1] |
| *rs560131* | 6 | 7047411 | *5' of snoU13* | 1.19×10^-11^ | [1] |
| *rs7743312* | 6 | 7049929 | *5' of snoU13* | 4.73×10^-9^ | [1] |
| *rs7769136* | 6 | 7052225 | *5' of snoU13* | 1.46×10^-8^ | [1] |
| *rs592377* | 6 | 7060902 | *5' of snoU13* | 5.23×10^-10^ | [1] |
| *rs2148088* | 6 | 7061912 | *5' of snoU13* | 1.55×10^-9^ | [1] |
| *rs6936076* | 6 | 7063271 | *5' of snoU13* | 3.16×10^-9^ | [1] |
| *rs2326878* | 6 | 7075094 | *5' of RREB1* | 7.85×10^-17^ | [1] |
| *rs611555* | 6 | 7075822 | *5' of RREB1* | 2.37×10^-17^ | [1] |
| *rs6912908* | 6 | 7080522 | *5' of RREB1* | 2.37×10^-17^ | [1] |
| *rs501510* | 6 | 7081840 | *5' of RREB1* | 3.40×10^-17^ | [1] |
| *rs9405325* | 6 | 7082633 | *5' of RREB1* | 9.78×10^-18^ | [1] |
| *rs13213992* | 6 | 7083218 | *5' of RREB1* | 1.66×10^-17^ | [1] |
| *rs4959424* | 6 | 7084857 | *5' of RREB1* | 1.08×10^-16^ | [1] |
| *rs675209* | 6 | 7102084 | *5' of RREB1* | 1.25×10^-23^ | [1] |
| *rs2842895* | 6 | 7106316 | *5' of RREB1* | 8.66×10^-17^ | [1] |
| *rs1285874* | 6 | 7115533 | *RREB1* | 4.84×10^-13^ | [1] |
| *rs1285875* | 6 | 7115927 | *RREB1* | 7.79×10^-15^ | [1] |
| *rs11755724* | 6 | 7118990 | *RREB1* | 6.03×10^-19^ | [1] |
| *rs6933716* | 6 | 7125709 | *RREB1* | 6.31×10^-14^ | [1] |
| *rs687467* | 6 | 7128076 | *RREB1* | 1.05×10^-16^ | [1] |
| *rs622404* | 6 | 7130596 | *RREB1* | 1.47×10^-17^ | [1] |
| *rs630258* | 6 | 7134401 | *RREB1* | 4.05×10^-17^ | [1] |
| *rs1285879* | 6 | 7137363 | *RREB1* | 9.22×10^-17^ | [1] |
| *rs665723* | 6 | 7139064 | *RREB1* | 3.20×10^-17^ | [1] |
| *rs4585612* | 6 | 7153152 | *RREB1* | 1.93×10^-16^ | [1] |
| *rs1334577* | 6 | 7211751 | *RREB1* | 2.11×10^-18^ | [1] |
| *rs17762454* | 6 | 7213200 | *RREB1* | 8.07×10^-17^ | [1] |
| *rs143231463* | 6 | 7226810 | *RREB1* | 2.00×10^-16^ | [4] |
| *rs141635364* | 6 | 7231115 | *RREB1* | 2.00×10^-16^ | [4] |
| *rs301395* | 6 | 25486626 | *LRRC16A* | 7.76×10^-17^ | [1] |
| *rs4712944* | 6 | 25488265 | *LRRC16A* | 1.24×10^-16^ | [1] |
| *rs12528639* | 6 | 25491807 | *LRRC16A* | 1.93×10^-15^ | [1] |
| *rs1034050* | 6 | 25492364 | *LRRC16A* | 1.34×10^-17^ | [1] |
| *rs12526321* | 6 | 25494503 | *LRRC16A* | 1.51×10^-10^ | [1] |
| *rs17252870* | 6 | 25494666 | *LRRC16A* | 4.42×10^-11^ | [1] |
| *rs301393* | 6 | 25500709 | *LRRC16A* | 1.47×10^-10^ | [1] |
| *rs17253044* | 6 | 25503735 | *LRRC16A* | 6.87×10^-11^ | [1] |
| *rs407934* | 6 | 25504562 | *LRRC16A* | 6.45×10^-9^ | [1] |
| *rs1747568* | 6 | 25506072 | *LRRC16A* | 3.44×10^-8^ | [1] |
| *rs572657* | 6 | 25513147 | *LRRC16A* | 4.57×10^-8^ | [1] |
| *rs1408280* | 6 | 25513240 | *LRRC16A* | 4.66×10^-8^ | [1] |
| *rs1830798* | 6 | 25513276 | *LRRC16A* | 4.61×10^-8^ | [1] |
| *rs301390* | 6 | 25514216 | *LRRC16A* | 3.61×10^-8^ | [1] |
| *rs301383* | 6 | 25515234 | *LRRC16A* | 4.46×10^-8^ | [1] |
| *rs301382* | 6 | 25515450 | *LRRC16A* | 4.34×10^-8^ | [1] |
| *rs301381* | 6 | 25515576 | *LRRC16A* | 4.05×10^-8^ | [1] |
| *rs150551* | 6 | 25533930 | *LRRC16A* | 2.31×10^-8^ | [1] |
| *rs969297* | 6 | 25543639 | *LRRC16A* | 4.86×10^-8^ | [1] |
| *rs2064128* | 6 | 25550139 | *LRRC16A* | 1.65×10^-10^ | [1] |
| *rs169946* | 6 | 25552143 | *LRRC16A* | 4.39×10^-8^ | [1] |
| *rs3804133* | 6 | 25552868 | *LRRC16A* | 3.10×10^-10^ | [1] |
| *rs214055* | 6 | 25560264 | *LRRC16A* | 2.33×10^-10^ | [1] |
| *rs3788994* | 6 | 25587836 | *LRRC16A* | 6.27×10^-11^ | [1] |
| *rs13212936* | 6 | 25588815 | *LRRC16A* | 2.37×10^-8^ | [1] |
| *rs1028318* | 6 | 25590224 | *LRRC16A* | 9.47×10^-9^ | [1] |
| *rs2077393* | 6 | 25607365 | *LRRC16A* | 1.32×10^-8^ | [8] |
| *rs742132* | 6 | 25607571 | *LRRC16A* | 8.50×10^-9^ | [3] |
| *rs6903765* | 6 | 25609738 | *LRRC16A* | 1.92×10^-8^ | [1] |
| *rs6908390* | 6 | 25609756 | *LRRC16A* | 2.75×10^-8^ | [1] |
| *rs3804105* | 6 | 25612683 | *LRRC16A* | 2.42×10^-8^ | [1] |
| *rs10946785* | 6 | 25617068 | *LRRC16A* | 2.68×10^-8^ | [1] |
| *rs1997672* | 6 | 25617544 | *LRRC16A* | 3.19×10^-8^ | [1] |
| *rs7761700* | 6 | 25618191 | *LRRC16A* | 2.79×10^-8^ | [1] |
| *rs2281070* | 6 | 25622116 | *3' of LRRC16A* | 1.60×10^-8^ | [1] |
| *rs12183240* | 6 | 25624416 | *3' of LRRC16A* | 2.07×10^-8^ | [1] |
| *rs12201186* | 6 | 25625595 | *3' of LRRC16A* | 2.65×10^-8^ | [1] |
| *rs10223506* | 6 | 25627216 | *3' of LRRC16A* | 2.41×10^-8^ | [1] |
| *rs9467538* | 6 | 25628688 | *3' of LRRC16A* | 2.62×10^-8^ | [1] |
| *rs2223323* | 6 | 25628852 | *3' of LRRC16A* | 2.71×10^-8^ | [1] |
| *rs2205933* | 6 | 25628924 | *3' of LRRC16A* | 2.72×10^-8^ | [1] |
| *rs1980449* | 6 | 25629658 | *3' of LRRC16A* | 5.87×10^-10^ | [1] |
| *rs1980450* | 6 | 25629747 | *3' of LRRC16A* | 5.00×10^-8^ | [1] |
| *rs1980451* | 6 | 25630091 | *3' of LRRC16A* | 2.77×10^-8^ | [1] |
| *rs1980452* | 6 | 25630136 | *3' of LRRC16A* | 3.18×10^-8^ | [1] |
| *rs9467541* | 6 | 25630549 | *3' of LRRC16A* | 3.11×10^-8^ | [1] |
| *rs2072844* | 6 | 25632041 | *3' of LRRC16A* | 4.19×10^-8^ | [1] |
| *rs4140641* | 6 | 25635400 | *3' of LRRC16A* | 2.10×10^-10^ | [1] |
| *rs11969981* | 6 | 25650817 | *5' of SCGN* | 1.02×10^-9^ | [1] |
| *rs12528182* | 6 | 25651197 | *5' of SCGN* | 4.51×10^-8^ | [1] |
| *rs12524760* | 6 | 25651263 | *5' of SCGN* | 4.70×10^-8^ | [1] |
| *rs4140640* | 6 | 25651650 | *5' of SCGN* | 4.76×10^-8^ | [1] |
| *rs2294344* | 6 | 25652225 | *5' of SCGN* | 1.47×10^-9^ | [1] |
| *rs2072846* | 6 | 25655104 | *SCGN* | 4.96×10^-8^ | [1] |
| *rs10484591* | 6 | 25656661 | *SCGN* | 3.24×10^-8^ | [1] |
| *rs4712950* | 6 | 25657590 | *SCGN* | 2.68×10^-10^ | [1] |
| *rs2205936* | 6 | 25685492 | *SCGN* | 6.22×10^-27^ | [1] |
| *rs9366627* | 6 | 25686405 | *SCGN* | 1.19×10^-11^ | [1] |
| *rs9379778* | 6 | 25687579 | *SCGN* | 1.55×10^-9^ | [1] |
| *rs4711093* | 6 | 25687895 | *SCGN* | 1.68×10^-9^ | [1] |
| *rs2294346* | 6 | 25689487 | *SCGN* | 5.63×10^-28^ | [1] |
| *rs17492659* | 6 | 25691362 | *SCGN* | 7.98×10^-15^ | [1] |
| *rs4419666* | 6 | 25693274 | *SCGN* | 2.66×10^-22^ | [1] |
| *rs4409177* | 6 | 25694491 | *SCGN* | 1.99×10^-21^ | [1] |
| *rs3922681* | 6 | 25698481 | *SCGN* | 3.10×10^-10^ | [1] |
| *rs9467570* | 6 | 25699283 | *SCGN* | 9.52×10^-22^ | [1] |
| *rs11794* | 6 | 25701718 | *SCGN* | 1.19×10^-9^ | [1] |
| *rs6908713* | 6 | 25703611 | *3' of SCGN* | 9.35×10^-10^ | [1] |
| *rs9467573* | 6 | 25704190 | *3' of SCGN* | 4.53×10^-23^ | [1] |
| *rs9467574* | 6 | 25705718 | *3' of SCGN* | 3.49×10^-23^ | [1] |
| *rs4711094* | 6 | 25706174 | *3' of SCGN* | 1.37×10^-8^ | [1] |
| *rs4395714* | 6 | 25708714 | *3' of SCGN* | 2.06×10^-14^ | [1] |
| *rs6456688* | 6 | 25708960 | *3' of SCGN* | 4.01×10^-21^ | [1] |
| *rs6456693* | 6 | 25710087 | *3' of SCGN* | 1.74×10^-23^ | [1] |
| *rs6912391* | 6 | 25710763 | *3' of SCGN* | 6.95×10^-25^ | [1] |
| *rs6456694* | 6 | 25712894 | *3' of SCGN* | 1.13×10^-8^ | [1] |
| *rs4236036* | 6 | 25712985 | *3' of SCGN* | 9.32×10^-15^ | [1] |
| *rs9379782* | 6 | 25715023 | *3' of HIST1H2AA* | 5.79×10^-10^ | [1] |
| *rs4132072* | 6 | 25717843 | *3' of HIST1H2AA* | 6.59×10^-10^ | [1] |
| *rs9461204* | 6 | 25718466 | *3' of HIST1H2AA* | 5.96×10^-23^ | [1] |
| *rs7450798* | 6 | 25719280 | *3' of HIST1H2AA* | 5.94×10^-10^ | [1] |
| *rs9348692* | 6 | 25720812 | *3' of HIST1H2AA* | 4.48×10^-10^ | [1] |
| *rs9379783* | 6 | 25725207 | *3' of HIST1H2AA* | 4.88×10^-10^ | [1] |
| *rs9379784* | 6 | 25725506 | *3' of HIST1H2AA* | 4.81×10^-10^ | [1] |
| *rs12215823* | 6 | 25726074 | *3' of HIST1H2AA* | 1.17×10^-28^ | [1] |
| *rs9379785* | 6 | 25726213 | *3' of HIST1H2AA* | 4.63×10^-10^ | [1] |
| *rs9358871* | 6 | 25726675 | *HIST1H2AA* | 4.71×10^-10^ | [1] |
| *rs4711095* | 6 | 25726774 | *HIST1H2AA* | 4.78×10^-10^ | [1] |
| *rs4711096* | 6 | 25727054 | *5' of HIST1H2BA* | 4.84×10^-10^ | [1] |
| *rs4712959* | 6 | 25727057 | *5' of HIST1H2BA* | 2.29×10^-10^ | [1] |
| *rs4712960* | 6 | 25727265 | *HIST1H2BA* | 5.30×10^-10^ | [1] |
| *rs4712961* | 6 | 25727334 | *HIST1H2BA* | 5.33×10^-10^ | [1] |
| *rs9358872* | 6 | 25727517 | *HIST1H2BA* | 6.11×10^-10^ | [1] |
| *rs17320558* | 6 | 25727527 | *HIST1H2BA* | 5.84×10^-15^ | [1] |
| *rs17267614* | 6 | 25730027 | *3' of HIST1H2BA* | 1.16×10^-9^ | [1] |
| *rs9379786* | 6 | 25730255 | *3' of HIST1H2BA* | 5.63×10^-10^ | [1] |
| *rs9358873* | 6 | 25731783 | *3' of HIST1H2BA* | 4.25×10^-10^ | [1] |
| *rs4711097* | 6 | 25732314 | *3' of HIST1H2BA* | 3.57×10^-10^ | [1] |
| *rs4464787* | 6 | 25733558 | *3' of HIST1H2BA* | 1.53×10^-9^ | [1] |
| *rs9461210* | 6 | 25734073 | *3' of HIST1H2BA* | 2.06×10^-23^ | [1] |
| *rs4360128* | 6 | 25734558 | *3' of HIST1H2BA* | 4.58×10^-10^ | [1] |
| *rs3923725* | 6 | 25734920 | *3' of HIST1H2BA* | 4.54×10^-10^ | [1] |
| *rs3922699* | 6 | 25734969 | *3' of HIST1H2BA* | 4.88×10^-10^ | [1] |
| *rs6924794* | 6 | 25735874 | *3' of HIST1H2BA* | 5.10×10^-10^ | [1] |
| *rs3922842* | 6 | 25736564 | *3' of HIST1H2BA* | 7.31×10^-10^ | [1] |
| *rs9358875* | 6 | 25738008 | *3' of HIST1H2BA* | 1.23×10^-8^ | [1] |
| *rs9358876* | 6 | 25738956 | *3' of HIST1H2BA* | 1.32×10^-8^ | [1] |
| *rs9393661* | 6 | 25739080 | *3' of HIST1H2BA* | 1.03×10^-9^ | [1] |
| *rs9356986* | 6 | 25739119 | *3' of HIST1H2BA* | 1.03×10^-9^ | [1] |
| *rs9356987* | 6 | 25739187 | *3' of HIST1H2BA* | 1.04×10^-9^ | [1] |
| *rs9358877* | 6 | 25739228 | *3' of HIST1H2BA* | 9.05×10^-10^ | [1] |
| *rs12663099* | 6 | 25739518 | *3' of HIST1H2BA* | 9.04×10^-10^ | [1] |
| *rs9393662* | 6 | 25739670 | *3' of HIST1H2BA* | 8.77×10^-10^ | [1] |
| *rs9358878* | 6 | 25740273 | *3' of HIST1H2BA* | 9.41×10^-10^ | [1] |
| *rs4712963* | 6 | 25740502 | *3' of HIST1H2BA* | 5.48×10^-10^ | [1] |
| *rs9366629* | 6 | 25741216 | *3' of HIST1H2BA* | 1.03×10^-9^ | [1] |
| *rs6456697* | 6 | 25741434 | *5' of SLC17A4* | 9.85×10^-10^ | [1] |
| *rs4711103* | 6 | 25742224 | *5' of SLC17A4* | 6.75×10^-10^ | [1] |
| *rs9467591* | 6 | 25742699 | *5' of SLC17A4* | 2.08×10^-19^ | [1] |
| *rs9358880* | 6 | 25743161 | *5' of SLC17A4* | 8.92×10^-10^ | [1] |
| *rs9393665* | 6 | 25744284 | *5' of SLC17A4* | 8.57×10^-10^ | [1] |
| *rs1937131* | 6 | 25744831 | *5' of SLC17A4* | 8.50×10^-10^ | [1] |
| *rs7754296* | 6 | 25744878 | *5' of SLC17A4* | 1.15×10^-9^ | [1] |
| *rs7754733* | 6 | 25745049 | *5' of SLC17A4* | 9.30×10^-10^ | [1] |
| *rs7754814* | 6 | 25745243 | *5' of SLC17A4* | 9.59×10^-10^ | [1] |
| *rs7775354* | 6 | 25745266 | *5' of SLC17A4* | 1.43×10^-9^ | [1] |
| *rs9379789* | 6 | 25745579 | *5' of SLC17A4* | 9.83×10^-10^ | [1] |
| *rs6941933* | 6 | 25745702 | *5' of SLC17A4* | 1.02×10^-9^ | [1] |
| *rs6923367* | 6 | 25745852 | *5' of SLC17A4* | 3.77×10^-8^ | [1] |
| *rs2154219* | 6 | 25749957 | *5' of SLC17A4* | 3.40×10^-8^ | [1] |
| *rs10498728* | 6 | 25751320 | *5' of SLC17A4* | 1.41×10^-23^ | [1] |
| *rs12207270* | 6 | 25751721 | *5' of SLC17A4* | 7.92×10^-24^ | [1] |
| *rs9348694* | 6 | 25753640 | *5' of SLC17A4* | 8.37×10^-10^ | [1] |
| *rs1892256* | 6 | 25754271 | *5' of SLC17A4* | 9.15×10^-24^ | [1] |
| *rs13194155* | 6 | 25755508 | *SLC17A4* | 6.01×10^-21^ | [1] |
| *rs16890999* | 6 | 25755583 | *SLC17A4* | 2.16×10^-23^ | [1] |
| *rs9358886* | 6 | 25758253 | *SLC17A4* | 1.01×10^-9^ | [1] |
| *rs17268697* | 6 | 25758448 | *SLC17A4* | 1.58×10^-16^ | [1] |
| *rs2000351* | 6 | 25759783 | *SLC17A4* | 1.63×10^-12^ | [1] |
| *rs7770037* | 6 | 25760591 | *SLC17A4* | 1.68×10^-12^ | [1] |
| *rs4236040* | 6 | 25761209 | *SLC17A4* | 6.29×10^-11^ | [1] |
| *rs4712969* | 6 | 25764192 | *SLC17A4* | 5.59×10^-11^ | [1] |
| *rs2186087* | 6 | 25764842 | *SLC17A4* | 3.48×10^-12^ | [1] |
| *rs1317816* | 6 | 25765390 | *SLC17A4* | 8.44×10^-12^ | [1] |
| *rs6937800* | 6 | 25765943 | *SLC17A4* | 4.67×10^-11^ | [1] |
| *rs6902211* | 6 | 25767437 | *SLC17A4* | 5.46×10^-11^ | [1] |
| *rs3778272* | 6 | 25767661 | *SLC17A4* | 1.21×10^-10^ | [1] |
| *rs2275904* | 6 | 25768085 | *SLC17A4* | 1.36×10^-9^ | [1] |
| *rs2275905* | 6 | 25768106 | *SLC17A4* | 3.96×10^-12^ | [1] |
| *rs1892248* | 6 | 25768914 | *SLC17A4* | 9.31×10^-13^ | [1] |
| *rs1892250* | 6 | 25769024 | *SLC17A4* | 6.57×10^-11^ | [1] |
| *rs1892251* | 6 | 25769349 | *SLC17A4* | 2.09×10^-8^ | [1] |
| *rs2328892* | 6 | 25769510 | *SLC17A4* | 4.75×10^-12^ | [1] |
| *rs1937127* | 6 | 25769872 | *SLC17A4* | 4.59×10^-12^ | [1] |
| *rs2328893* | 6 | 25770239 | *SLC17A4* | 1.56×10^-24^ | [1] |
| *rs4712970* | 6 | 25770707 | *SLC17A4* | 6.95×10^-12^ | [1] |
| *rs4712971* | 6 | 25771900 | *SLC17A4* | 9.77×10^-11^ | [1] |
| *rs4712972* | 6 | 25772047 | *SLC17A4* | 9.74×10^-10^ | [1] |
| *rs1892252* | 6 | 25772639 | *SLC17A4* | 9.68×10^-10^ | [1] |
| *rs2275906* | 6 | 25773809 | *SLC17A4* | 1.65×10^-16^ | [1] |
| *rs6456701* | 6 | 25774130 | *SLC17A4* | 3.33×10^-10^ | [1] |
| *rs17269374* | 6 | 25775242 | *SLC17A4* | 3.01×10^-12^ | [1] |
| *rs1954598* | 6 | 25776060 | *SLC17A4* | 2.03×10^-10^ | [1] |
| *rs2154218* | 6 | 25776290 | *SLC17A4* | 2.77×10^-13^ | [1] |
| *rs11754288* | 6 | 25776949 | *SLC17A4* | 1.40×10^-11^ | [11] |
| *rs9356988* | 6 | 25777481 | *SLC17A4* | 3.70×10^-12^ | [1] |
| *rs3949215* | 6 | 25777497 | *SLC17A4* | 2.25×10^-10^ | [1] |
| *rs12201071* | 6 | 25778055 | *SLC17A4* | 2.48×10^-24^ | [1] |
| *rs1317510* | 6 | 25778924 | *SLC17A4* | 1.20×10^-13^ | [1] |
| *rs1141034* | 6 | 25780332 | *SLC17A4* | 3.88×10^-13^ | [1] |
| *rs6910549* | 6 | 25780811 | *SLC17A4* | 6.67×10^-10^ | [1] |
| *rs7749149* | 6 | 25781139 | *SLC17A4* | 1.95×10^-14^ | [1] |
| *rs10946798* | 6 | 25781853 | *3' of SLC17A4* | 1.74×10^-28^ | [1] |
| *rs1892253* | 6 | 25782314 | *3' of SLC17A1* | 6.47×10^-10^ | [1] |
| *rs3923* | 6 | 25783315 | *SLC17A1* | 1.00×10^-26^ | [1] |
| *rs3757131* | 6 | 25783909 | *SLC17A1* | 3.26×10^-49^ | [1] |
| *rs12662869* | 6 | 25784481 | *SLC17A1* | 1.76×10^-14^ | [1] |
| *rs13197601* | 6 | 25785935 | *SLC17A1* | 3.08×10^-49^ | [1] |
| *rs13213957* | 6 | 25786226 | *SLC17A1* | 2.24×10^-9^ | [1] |
| *rs3799344* | 6 | 25786993 | *SLC17A1* | 1.43×10^-47^ | [1] |
| *rs2096386* | 6 | 25787817 | *SLC17A1* | 2.46×10^-26^ | [1] |
| *rs9393670* | 6 | 25789061 | *SLC17A1* | 1.91×10^-28^ | [1] |
| *rs9393671* | 6 | 25789189 | *SLC17A1* | 2.73×10^-13^ | [1] |
| *rs10214468* | 6 | 25789390 | *SLC17A1* | 3.46×10^-11^ | [1] |
| *rs13200921* | 6 | 25790378 | *SLC17A1* | 2.76×10^-10^ | [1] |
| *rs3799346* | 6 | 25791354 | *SLC17A1* | 4.26×10^-31^ | [1] |
| *rs1165157* | 6 | 25792250 | *SLC17A1* | 1.66×10^-13^ | [1] |
| *rs942377* | 6 | 25792292 | *SLC17A1* | 3.92×10^-31^ | [1] |
| *rs1165156* | 6 | 25792978 | *SLC17A1* | 1.91×10^-13^ | [1] |
| *rs12209856* | 6 | 25793673 | *SLC17A1* | 2.36×10^-13^ | [1] |
| *rs2762353* | 6 | 25794431 | *SLC17A1* | 4.23×10^-56^ | [1] |
| *rs1165155* | 6 | 25795577 | *SLC17A1* | 2.94×10^-13^ | [1] |
| *rs1165215* | 6 | 25798932 | *SLC17A1* | 3.42×10^-57^ | [1] |
| *rs1165211* | 6 | 25800922 | *SLC17A1* | 1.64×10^-13^ | [1] |
| *rs1165209* | 6 | 25801319 | *SLC17A1* | 1.98×10^-57^ | [1] |
| *rs1324082* | 6 | 25801971 | *SLC17A1* | 3.52×10^-31^ | [1] |
| *rs12191655* | 6 | 25803095 | *SLC17A1* | 3.59×10^-10^ | [1] |
| *rs1165208* | 6 | 25803904 | *SLC17A1* | 1.94×10^-13^ | [1] |
| *rs9467604* | 6 | 25805002 | *SLC17A1* | 2.76×10^-25^ | [1] |
| *rs9461216* | 6 | 25808743 | *SLC17A1* | 4.27×10^-10^ | [1] |
| *rs9467606* | 6 | 25809218 | *SLC17A1* | 1.84×10^-13^ | [1] |
| *rs9467609* | 6 | 25809751 | *SLC17A1* | 1.22×10^-11^ | [1] |
| *rs1359231* | 6 | 25809798 | *SLC17A1* | 1.52×10^-17^ | [1] |
| *rs1575535* | 6 | 25809909 | *SLC17A1* | 1.98×10^-13^ | [1] |
| *rs1575534* | 6 | 25811404 | *SLC17A1* | 5.93×10^-31^ | [1] |
| *rs10498730* | 6 | 25812069 | *SLC17A1* | 1.30×10^-10^ | [1] |
| *rs942378* | 6 | 25812459 | *SLC17A1* | 4.40×10^-31^ | [1] |
| *rs9467613* | 6 | 25812641 | *SLC17A1* | 6.22×10^-10^ | [1] |
| *rs1165196* | 6 | 25813150 | *SLC17A1* | 5.00×10^-25^ | [2] |
| *rs7753366* | 6 | 25817518 | *SLC17A1* | 7.38×10^-31^ | [1] |
| *rs1165153* | 6 | 25817789 | *SLC17A1* | 1.16×10^-57^ | [1] |
| *rs1185567* | 6 | 25818588 | *SLC17A1* | 2.39×10^-57^ | [1] |
| *rs1183200* | 6 | 25818646 | *SLC17A1* | 3.31×10^-57^ | [1] |
| *rs12182983* | 6 | 25818755 | *SLC17A1* | 1.01×10^-31^ | [1] |
| *rs1165152* | 6 | 25818766 | *SLC17A1* | 2.96×10^-57^ | [1] |
| *rs6913879* | 6 | 25820428 | *SLC17A1* | 1.79×10^-31^ | [1] |
| *rs17270561* | 6 | 25820439 | *SLC17A1* | 1.60×10^-9^ | [1] |
| *rs6939997* | 6 | 25821224 | *SLC17A1* | 4.16×10^-10^ | [1] |
| *rs6940698* | 6 | 25821580 | *SLC17A1* | 7.83×10^-10^ | [1] |
| *rs1165151* | 6 | 25821616 | *SLC17A1* | 7.04×10^-70^ | [1] |
| *rs1185976* | 6 | 25822363 | *SLC17A1* | 1.67×10^-12^ | [1] |
| *rs3799352* | 6 | 25822620 | *SLC17A1* | 9.69×10^-60^ | [1] |
| *rs13201341* | 6 | 25822661 | *SLC17A1* | 2.47×10^-9^ | [1] |
| *rs1183201* | 6 | 25823444 | *SLC17A1* | 3.04×10^-14^ | [8] |
| *rs12211184* | 6 | 25823774 | *SLC17A1* | 1.94×10^-8^ | [1] |
| *rs1165181* | 6 | 25825390 | *SLC17A1* | 4.61×10^-12^ | [1] |
| *rs6456703* | 6 | 25826119 | *SLC17A1* | 5.46×10^-31^ | [1] |
| *rs1408268* | 6 | 25826986 | *SLC17A1* | 4.26×10^-31^ | [1] |
| *rs1165178* | 6 | 25827516 | *SLC17A1* | 2.46×10^-59^ | [1] |
| *rs765285* | 6 | 25828242 | *SLC17A1* | 5.92×10^-52^ | [1] |
| *rs13199775* | 6 | 25828782 | *SLC17A1* | 2.00×10^-10^ | [1] |
| *rs12200962* | 6 | 25828986 | *SLC17A1* | 2.92×10^-8^ | [1] |
| *rs13200784* | 6 | 25829633 | *SLC17A1* | 1.35×10^-30^ | [1] |
| *rs1165177* | 6 | 25829659 | *SLC17A1* | 4.52×10^-55^ | [1] |
| *rs1165176* | 6 | 25830298 | *SLC17A1* | 3.76×10^-57^ | [1] |
| *rs2070642* | 6 | 25831212 | *SLC17A1* | 3.81×10^-12^ | [1] |
| *rs1185569* | 6 | 25831603 | *SLC17A1* | 1.13×10^-50^ | [1] |
| *rs1185568* | 6 | 25834428 | *SLC17A3* | 4.07×10^-57^ | [1] |
| *rs1184803* | 6 | 25834658 | *SLC17A3* | 3.53×10^-50^ | [1] |
| *rs1185978* | 6 | 25835895 | *SLC17A3* | 5.53×10^-47^ | [1] |
| *rs9461219* | 6 | 25836927 | *SLC17A3* | 5.33×10^-10^ | [1] |
| *rs1165182* | 6 | 25837829 | *SLC17A3* | 1.74×10^-58^ | [1] |
| *rs6905614* | 6 | 25840485 | *SLC17A3* | 2.12×10^-56^ | [1] |
| *rs1408273* | 6 | 25840946 | *SLC17A3* | 1.41×10^-50^ | [1] |
| *rs1324088* | 6 | 25841122 | *SLC17A3* | 9.72×10^-10^ | [1] |
| *rs1324087* | 6 | 25841408 | *SLC17A3* | 6.49×10^-10^ | [1] |
| *rs4712976* | 6 | 25842203 | *SLC17A3* | 4.76×10^-12^ | [1] |
| *rs9393672* | 6 | 25842605 | *SLC17A3* | 4.27×10^-14^ | [8] |
| *rs3887266* | 6 | 25843746 | *SLC17A3* | 1.33×10^-18^ | [1] |
| *rs1165148* | 6 | 25844710 | *SLC17A3* | 2.03×10^-29^ | [1] |
| *rs942379* | 6 | 25849620 | *SLC17A3* | 7.99×10^-14^ | [8] |
| *rs1165189* | 6 | 25849779 | *SLC17A3* | 2.11×10^-29^ | [1] |
| *rs9467621* | 6 | 25851338 | *SLC17A3* | 5.18×10^-10^ | [1] |
| *rs1165187* | 6 | 25851369 | *SLC17A3* | 1.71×10^-29^ | [1] |
| *rs9467622* | 6 | 25854644 | *SLC17A3* | 5.13×10^-10^ | [1] |
| *rs1780969* | 6 | 25858432 | *SLC17A3* | 7.56×10^-24^ | [1] |
| *rs1179087* | 6 | 25858704 | *SLC17A3* | 2.66×10^-27^ | [1] |
| *rs1177441* | 6 | 25859413 | *SLC17A3* | 1.14×10^-9^ | [1] |
| *rs1165168* | 6 | 25859508 | *SLC17A3* | 7.15×10^-10^ | [1] |
| *rs1182814* | 6 | 25859554 | *SLC17A3* | 4.44×10^-29^ | [1] |
| *rs1408271* | 6 | 25859621 | *SLC17A3* | 1.12×10^-10^ | [1] |
| *rs1165167* | 6 | 25860688 | *SLC17A3* | 3.18×10^-29^ | [1] |
| *rs1165165* | 6 | 25862466 | *SLC17A3* | 1.32×10^-27^ | [1] |
| *rs1165164* | 6 | 25863481 | *SLC17A3* | 9.10×10^-28^ | [1] |
| *rs1165162* | 6 | 25863605 | *SLC17A3* | 1.76×10^-29^ | [1] |
| *rs17271121* | 6 | 25863650 | *SLC17A3* | 1.13×10^-17^ | [1] |
| *rs1165161* | 6 | 25864362 | *SLC17A3* | 1.09×10^-29^ | [1] |
| *rs1165160* | 6 | 25864456 | *SLC17A3* | 6.12×10^-53^ | [1] |
| *rs1165159* | 6 | 25864625 | *SLC17A3* | 3.37×10^-28^ | [1] |
| *rs13211947* | 6 | 25864818 | *SLC17A3* | 3.34×10^-9^ | [1] |
| *rs1184498* | 6 | 25864882 | *SLC17A3* | 4.65×10^-10^ | [1] |
| *rs1165158* | 6 | 25864898 | *SLC17A3* | 3.84×10^-29^ | [1] |
| *rs1165207* | 6 | 25865266 | *SLC17A3* | 1.28×10^-56^ | [1] |
| *rs1165206* | 6 | 25867566 | *SLC17A3* | 3.25×10^-25^ | [1] |
| *rs1184804* | 6 | 25868226 | *SLC17A3* | 6.48×10^-50^ | [1] |
| *rs548987* | 6 | 25869371 | *SLC17A3* | 2.51×10^-9^ | [1] |
| *rs523383* | 6 | 25869848 | *SLC17A3* | 5.75×10^-10^ | [1] |
| *rs1165205* | 6 | 25870542 | *SLC17A3* | 1.60×10^-13^ | [3] |
| *rs555460* | 6 | 25870655 | *SLC17A3* | 1.01×10^-9^ | [1] |
| *rs556339* | 6 | 25870745 | *SLC17A3* | 1.15×10^-29^ | [1] |
| *rs972087* | 6 | 25872579 | *SLC17A3* | 6.59×10^-10^ | [1] |
| *rs501220* | 6 | 25873025 | *SLC17A3* | 1.95×10^-9^ | [1] |
| *rs9467626* | 6 | 25873746 | *SLC17A3* | 5.32×10^-10^ | [1] |
| *rs13198474* | 6 | 25874423 | *SLC17A3* | 1.81×10^-9^ | [1] |
| *rs1165201* | 6 | 25874823 | *SLC17A3* | 6.87×10^-17^ | [1] |
| *rs12664474* | 6 | 25876089 | *SLC17A3* | 1.95×10^-20^ | [1] |
| *rs603089* | 6 | 25877970 | *SLC17A3* | 6.24×10^-18^ | [1] |
| *rs11964886* | 6 | 25878379 | *SLC17A3* | 1.72×10^-16^ | [1] |
| *rs11969868* | 6 | 25878506 | *SLC17A3* | 4.71×10^-16^ | [1] |
| *rs629835* | 6 | 25879330 | *SLC17A3* | 8.27×10^-17^ | [1] |
| *rs645279* | 6 | 25880494 | *SLC17A3* | 8.87×10^-16^ | [1] |
| *rs531750* | 6 | 25882642 | *5' of SLC17A3* | 3.15×10^-16^ | [1] |
| *rs599444* | 6 | 25883714 | *5' of SLC17A3* | 6.61×10^-16^ | [1] |
| *rs629444* | 6 | 25885814 | *5' of SLC17A3* | 7.17×10^-10^ | [1] |
| *rs537581* | 6 | 25886497 | *5' of SLC17A3* | 9.33×10^-15^ | [1] |
| *rs483906* | 6 | 25888094 | *5' of SLC17A3* | 1.08×10^-14^ | [1] |
| *rs9379800* | 6 | 25889099 | *5' of SLC17A3* | 2.70×10^-19^ | [1] |
| *rs518700* | 6 | 25889553 | *5' of SLC17A3* | 1.17×10^-12^ | [1] |
| *rs9467632* | 6 | 25889706 | *5' of SLC17A3* | 4.91×10^-9^ | [1] |
| *rs6910741* | 6 | 25895575 | *5' of SLC17A3* | 3.95×10^-13^ | [1] |
| *rs13220488* | 6 | 25896907 | *5' of SLC17A3* | 1.52×10^-8^ | [1] |
| *rs428469* | 6 | 25898624 | *3' of SLC17A2* | 1.34×10^-11^ | [1] |
| *rs16891142* | 6 | 25899139 | *3' of SLC17A2* | 2.51×10^-13^ | [1] |
| *rs13207673* | 6 | 25901133 | *3' of SLC17A2* | 3.54×10^-8^ | [1] |
| *rs9379801* | 6 | 25901711 | *3' of SLC17A2* | 1.50×10^-24^ | [1] |
| *rs9356991* | 6 | 25901758 | *3' of SLC17A2* | 1.17×10^-17^ | [1] |
| *rs6910138* | 6 | 25904352 | *3' of SLC17A2* | 2.16×10^-17^ | [1] |
| *rs7748167* | 6 | 25904652 | *3' of SLC17A2* | 4.19×10^-8^ | [1] |
| *rs9461222* | 6 | 25905111 | *3' of SLC17A2* | 2.10×10^-8^ | [1] |
| *rs9467635* | 6 | 25909166 | *3' of SLC17A2* | 2.25×10^-8^ | [1] |
| *rs3799371* | 6 | 25912816 | *3' of SLC17A2* | 2.38×10^-17^ | [1] |
| *rs6932113* | 6 | 25913098 | *SLC17A2* | 1.24×10^-16^ | [1] |
| *rs6938233* | 6 | 25914077 | *SLC17A2* | 3.75×10^-17^ | [1] |
| *rs2071301* | 6 | 25914263 | *SLC17A2* | 1.96×10^-17^ | [1] |
| *rs1865760* | 6 | 25916979 | *SLC17A2* | 2.24×10^-17^ | [1] |
| *rs9295675* | 6 | 25918473 | *SLC17A2* | 2.39×10^-8^ | [1] |
| *rs3752421* | 6 | 25918688 | *SLC17A2* | 1.72×10^-17^ | [1] |
| *rs17526722* | 6 | 25918855 | *SLC17A2* | 4.52×10^-8^ | [1] |
| *rs13195279* | 6 | 25919431 | *SLC17A2* | 3.36×10^-8^ | [1] |
| *rs9467636* | 6 | 25919549 | *SLC17A2* | 1.76×10^-17^ | [1] |
| *rs7749342* | 6 | 25920265 | *SLC17A2* | 9.10×10^-29^ | [1] |
| *rs9358893* | 6 | 25921761 | *SLC17A2* | 1.83×10^-17^ | [1] |
| *rs3799372* | 6 | 25922311 | *SLC17A2* | 1.87×10^-17^ | [1] |
| *rs1540273* | 6 | 25924158 | *SLC17A2* | 1.85×10^-17^ | [1] |
| *rs2071297* | 6 | 25924513 | *SLC17A2* | 2.32×10^-17^ | [1] |
| *rs7770139* | 6 | 25925823 | *SLC17A2* | 1.79×10^-16^ | [1] |
| *rs3734523* | 6 | 25925987 | *SLC17A2* | 3.18×10^-8^ | [1] |
| *rs10484431* | 6 | 25926674 | *SLC17A2* | 8.64×10^-13^ | [1] |
| *rs17586553* | 6 | 25928775 | *SLC17A2* | 2.50×10^-8^ | [1] |
| *rs3799373* | 6 | 25929173 | *SLC17A2* | 3.04×10^-17^ | [1] |
| *rs199741* | 6 | 25931577 | *5' of SLC17A2* | 2.84×10^-10^ | [1] |
| *rs199737* | 6 | 25933538 | *5' of SLC17A2* | 3.09×10^-19^ | [1] |
| *rs442601* | 6 | 25934524 | *5' of SLC17A2* | 3.61×10^-18^ | [1] |
| *rs9461224* | 6 | 25936402 | *5' of SLC17A2* | 8.93×10^-18^ | [1] |
| *rs199736* | 6 | 25936787 | *5' of SLC17A2* | 2.83×10^-18^ | [1] |
| *rs9393676* | 6 | 25936944 | *5' of SLC17A2* | 2.06×10^-31^ | [1] |
| *rs9295678* | 6 | 25937033 | *5' of SLC17A2* | 2.13×10^-31^ | [1] |
| *rs1436307* | 6 | 25939723 | *5' of SLC17A2* | 1.06×10^-17^ | [1] |
| *rs199734* | 6 | 25940393 | *5' of SLC17A2* | 1.94×10^-19^ | [1] |
| *rs9379806* | 6 | 25940958 | *5' of SLC17A2* | 6.13×10^-18^ | [1] |
| *rs9358896* | 6 | 25943197 | *5' of SLC17A2* | 1.69×10^-17^ | [1] |
| *rs9295680* | 6 | 25944103 | *5' of SLC17A2* | 1.03×10^-18^ | [1] |
| *rs9295681* | 6 | 25944117 | *5' of SLC17A2* | 5.00×10^-20^ | [1] |
| *rs2051541* | 6 | 25945211 | *5' of SLC17A2* | 7.44×10^-31^ | [1] |
| *rs1436306* | 6 | 25948421 | *5' of TRIM38* | 6.17×10^-20^ | [1] |
| *rs1541987* | 6 | 25950397 | *5' of TRIM38* | 3.85×10^-20^ | [1] |
| *rs199726* | 6 | 25953360 | *5' of TRIM38* | 2.16×10^-18^ | [1] |
| *rs2876693* | 6 | 25954434 | *5' of TRIM38* | 1.52×10^-17^ | [1] |
| *rs9467646* | 6 | 25957408 | *5' of TRIM38* | 1.04×10^-17^ | [1] |
| *rs9467647* | 6 | 25957642 | *5' of TRIM38* | 5.69×10^-20^ | [1] |
| *rs10946800* | 6 | 25957773 | *5' of TRIM38* | 6.36×10^-20^ | [1] |
| *rs12195653* | 6 | 25958621 | *5' of TRIM38* | 7.51×10^-18^ | [1] |
| *rs9467652* | 6 | 25959127 | *5' of TRIM38* | 6.56×10^-19^ | [1] |
| *rs199739* | 6 | 25960509 | *5' of TRIM38* | 9.81×10^-20^ | [1] |
| *rs129129* | 6 | 25961029 | *5' of TRIM38* | 3.85×10^-18^ | [1] |
| *rs12210098* | 6 | 25963966 | *TRIM38* | 2.73×10^-13^ | [1] |
| *rs1436310* | 6 | 25969958 | *TRIM38* | 8.79×10^-20^ | [1] |
| *rs1436309* | 6 | 25970109 | *TRIM38* | 8.26×10^-20^ | [1] |
| *rs115810* | 6 | 25975883 | *TRIM38* | 6.39×10^-20^ | [1] |
| *rs1130000* | 6 | 25985396 | *3' of TRIM38* | 4.92×10^-16^ | [1] |
| *rs2032450* | 6 | 25993114 | *3' of TRIM38* | 5.80×10^-16^ | [1] |
| *rs9467656* | 6 | 25993559 | *3' of TRIM38* | 4.48×10^-16^ | [1] |
| *rs2013063* | 6 | 25994098 | *3' of TRIM38* | 3.56×10^-16^ | [1] |
| *rs12216125* | 6 | 25997458 | *3' of TRIM38* | 1.32×10^-11^ | [1] |
| *rs199753* | 6 | 26001888 | *3' of HIST1H1A* | 1.27×10^-14^ | [1] |
| *rs9393681* | 6 | 26008260 | *3' of HIST1H1A* | 1.36×10^-15^ | [1] |
| *rs9467658* | 6 | 26010881 | *3' of HIST1H1A* | 2.25×10^-17^ | [1] |
| *rs199752* | 6 | 26012875 | *3' of HIST1H1A* | 3.28×10^-16^ | [1] |
| *rs199751* | 6 | 26015583 | *3' of HIST1H1A* | 2.89×10^-17^ | [1] |
| *rs199750* | 6 | 26016462 | *3' of HIST1H1A* | 2.86×10^-17^ | [1] |
| *rs9461230* | 6 | 26019240 | *5' of HIST1H1A* | 4.65×10^-16^ | [1] |
| *rs2157050* | 6 | 26020431 | *5' of HIST1H3A* | 4.36×10^-12^ | [1] |
| *rs9467664* | 6 | 26021813 | *5' of HIST1H4A* | 1.60×10^-16^ | [1] |
| *rs9379818* | 6 | 26023206 | *3' of HIST1H4A* | 1.13×10^-13^ | [1] |
| *rs9358901* | 6 | 26024436 | *3' of HIST1H4A* | 2.91×10^-15^ | [1] |
| *rs2032449* | 6 | 26026599 | *3' of HIST1H4B* | 7.20×10^-11^ | [1] |
| *rs3752419* | 6 | 26027433 | *HIST1H4B* | 1.48×10^-11^ | [1] |
| *rs1540276* | 6 | 26028819 | *5' of HIST1H4B* | 2.11×10^-17^ | [1] |
| *rs2213284* | 6 | 26031868 | *HIST1H3B* | 1.09×10^-16^ | [1] |
| *rs2230655* | 6 | 26033506 | *HIST1H2AB* | 5.76×10^-17^ | [1] |
| *rs4401650* | 6 | 26035208 | *5' of HIST1H2AB* | 1.05×10^-15^ | [1] |
| *rs1540275* | 6 | 26036476 | *5' of HIST1H2AB* | 6.01×10^-17^ | [1] |
| *rs7753826* | 6 | 26042239 | *3' of HIST1H2BB* | 2.10×10^-9^ | [1] |
| *rs2032447* | 6 | 26044369 | *5' of HIST1H2BB* | 2.26×10^-13^ | [1] |
| *rs7756117* | 6 | 26046565 | *3' of HIST1H3C* | 9.17×10^-16^ | [1] |
| *rs7772312* | 6 | 26049616 | *3' of HIST1H3C* | 2.10×10^-10^ | [1] |
| *rs9379820* | 6 | 26049924 | *3' of HIST1H3C* | 1.85×10^-10^ | [1] |
| *rs10425* | 6 | 26056549 | *HIST1H1C* | 2.62×10^-18^ | [1] |
| *rs807214* | 6 | 26061769 | *5' of HIST1H1C* | 6.24×10^-21^ | [1] |
| *rs9358903* | 6 | 26061949 | *5' of HIST1H1C* | 3.44×10^-13^ | [1] |
| *rs807212* | 6 | 26065621 | *5' of HIST1H1C* | 3.18×10^-18^ | [1] |
| *rs9295684* | 6 | 26069669 | *5' of HIST1H1C* | 6.23×10^-13^ | [1] |
| *rs9295685* | 6 | 26071725 | *5' of HIST1H1C* | 2.43×10^-8^ | [1] |
| *rs9295687* | 6 | 26082710 | *5' of HFE* | 5.83×10^-11^ | [1] |
| *rs4529296* | 6 | 26083135 | *5' of HFE* | 6.07×10^-11^ | [1] |
| *rs9379825* | 6 | 26083871 | *5' of HFE* | 5.30×10^-11^ | [1] |
| *rs9467672* | 6 | 26083904 | *5' of HFE* | 5.25×10^-11^ | [1] |
| *rs9295688* | 6 | 26084217 | *5' of HFE* | 5.15×10^-11^ | [1] |
| *rs2006736* | 6 | 26086017 | *5' of HFE* | 5.74×10^-11^ | [1] |
| *rs1800702* | 6 | 26086463 | *5' of HFE* | 5.30×10^-11^ | [1] |
| *rs2794720* | 6 | 26087202 | *5' of HFE* | 1.62×10^-10^ | [1] |
| *rs2794719* | 6 | 26088890 | *HFE* | 5.41×10^-11^ | [1] |
| *rs2071303* | 6 | 26091336 | *HFE* | 9.80×10^-17^ | [1] |
| *rs1572982* | 6 | 26094367 | *HFE* | 4.26×10^-9^ | [1] |
| *rs12346* | 6 | 26097046 | *HFE* | 1.24×10^-16^ | [1] |
| *rs6918586* | 6 | 26097384 | *HFE* | 2.12×10^-9^ | [1] |
| *rs1150660* | 6 | 26101439 | *5' of HIST1H4C* | 2.50×10^-16^ | [1] |
| *rs198857* | 6 | 26102417 | *5' of HIST1H4C* | 5.71×10^-9^ | [1] |
| *rs198856* | 6 | 26102708 | *5' of HIST1H4C* | 6.72×10^-10^ | [1] |
| *rs198854* | 6 | 26104057 | *5' of HIST1H4C* | 1.13×10^-9^ | [1] |
| *rs198853* | 6 | 26104096 | *5' of HIST1H4C* | 8.16×10^-21^ | [1] |
| *rs198852* | 6 | 26104448 | *HIST1H4C* | 4.44×10^-15^ | [1] |
| *rs198848* | 6 | 26106325 | *3' of HIST1H1T* | 6.26×10^-15^ | [1] |
| *rs198845* | 6 | 26107790 | *HIST1H1T* | 2.09×10^-14^ | [1] |
| *rs198844* | 6 | 26108282 | *HIST1H1T* | 8.72×10^-9^ | [1] |
| *rs198838* | 6 | 26113340 | *3' of HIST1H2BC* | 1.11×10^-8^ | [1] |
| *rs198837* | 6 | 26113398 | *3' of HIST1H2BC* | 6.69×10^-9^ | [1] |
| *rs198836* | 6 | 26113616 | *3' of HIST1H2BC* | 1.59×10^-9^ | [1] |
| *rs198834* | 6 | 26114372 | *3' of HIST1H2BC* | 8.75×10^-14^ | [1] |
| *rs13161* | 6 | 26114702 | *3' of HIST1H2BC* | 1.47×10^-9^ | [1] |
| *rs198828* | 6 | 26119459 | *HIST1H2BC* | 8.59×10^-9^ | [1] |
| *rs198827* | 6 | 26121002 | *HIST1H2BC* | 4.11×10^-9^ | [1] |
| *rs198826* | 6 | 26121153 | *HIST1H2BC* | 1.94×10^-10^ | [1] |
| *rs198825* | 6 | 26122502 | *HIST1H2BC* | 2.11×10^-9^ | [1] |
| *rs198821* | 6 | 26123629 | *HIST1H2BC* | 9.23×10^-10^ | [1] |
| *rs198820* | 6 | 26124243 | *5' of HIST1H2BC* | 9.21×10^-11^ | [1] |
| *rs198815* | 6 | 26127271 | *HIST1H2AC* | 9.82×10^-12^ | [1] |
| *rs198814* | 6 | 26127759 | *HIST1H2AC* | 7.78×10^-9^ | [1] |
| *rs198809* | 6 | 26128766 | *HIST1H2AC* | 8.81×10^-9^ | [1] |
| *rs138551969* | 6 | 43748486 | *VEGFA* | 2.00×10^-16^ | [4] |
| *rs729761* | 6 | 43804571 | *3' of RP11-344J7.2* | 7.98×10^-16^ | [1] |
| *rs2396083* | 6 | 43804808 | *3' of RP11-344J7.2* | 1.23×10^-11^ | [1] |
| *rs744103* | 6 | 43805362 | *3' of RP11-344J7.2* | 8.31×10^-12^ | [1] |
| *rs881858* | 6 | 43806609 | *3' of RP11-344J7.2* | 1.50×10^-11^ | [1] |
| *rs9472135* | 6 | 43809802 | *3' of RP11-344J7.2* | 6.80×10^-11^ | [1] |
| *rs9369425* | 6 | 43810974 | *3' of RP11-344J7.2* | 1.09×10^-9^ | [1] |
| *rs9369427* | 6 | 43811430 | *3' of RP11-344J7.2* | 1.62×10^-9^ | [1] |
| *rs9472138* | 6 | 43811762 | *3' of RP11-344J7.2* | 1.50×10^-9^ | [1] |
| *rs10456526* | 6 | 43814625 | *3' of RP11-344J7.2* | 3.74×10^-9^ | [1] |
| *rs6937438* | 6 | 43815364 | *3' of RP11-344J7.2* | 4.09×10^-8^ | [1] |
| *rs9321453* | 6 | 134773554 | *RP11-557H15.3* | 1.00×10^-9^ | [25] |
| *rs2240466* | 7 | 72856269 | *BAZ1B* | 2.98×10^-9^ | [1] |
| *rs1178979* | 7 | 72856430 | *BAZ1B* | 9.80×10^-12^ | [1] |
| *rs1178977* | 7 | 72857049 | *BAZ1B* | 1.19×10^-12^ | [1] |
| *rs714052* | 7 | 72864869 | *BAZ1B* | 3.68×10^-9^ | [1] |
| *rs2074755* | 7 | 72877166 | *BAZ1B* | 1.61×10^-9^ | [1] |
| *rs12056034* | 7 | 72878645 | *BAZ1B* | 2.32×10^-9^ | [1] |
| *rs6976930* | 7 | 72885810 | *BAZ1B* | 3.14×10^-11^ | [1] |
| *rs200548390* | 7 | 72892517 | *BAZ1B* | 2.00×10^-16^ | [4] |
| *rs17145713* | 7 | 72904810 | *BAZ1B* | 2.16×10^-11^ | [1] |
| *rs13244268* | 7 | 72911843 | *BAZ1B* | 2.27×10^-9^ | [1] |
| *rs7811265* | 7 | 72934510 | *BAZ1B* | 2.17×10^-10^ | [1] |
| *rs11983997* | 7 | 72939244 | *5' of BAZ1B* | 1.76×10^-11^ | [1] |
| *rs17145732* | 7 | 72970268 | *BCL7B* | 1.25×10^-8^ | [1] |
| *rs13233571* | 7 | 72971231 | *BCL7B* | 1.03×10^-9^ | [1] |
| *rs12539316* | 7 | 72977898 | *5' of BCL7B* | 5.95×10^-9^ | [1] |
| *rs17145738* | 7 | 72982874 | *3' of TBL2* | 2.59×10^-9^ | [1] |
| *rs13232120* | 7 | 72983310 | *3' of TBL2* | 2.38×10^-9^ | [1] |
| *rs14415* | 7 | 72984780 | *TBL2* | 5.40×10^-9^ | [1] |
| *rs2286276* | 7 | 72987354 | *TBL2* | 7.24×10^-9^ | [1] |
| *rs11974409* | 7 | 72989390 | *TBL2* | 4.22×10^-11^ | [1] |
| *rs1051921* | 7 | 73007943 | *MLXIPL* | 5.06×10^-11^ | [1] |
| *rs17145750* | 7 | 73026378 | *MLXIPL* | 1.37×10^-9^ | [1] |
| *rs7798357* | 7 | 73032265 | *MLXIPL* | 1.27×10^-8^ | [1] |
| *rs7785479* | 7 | 73032835 | *MLXIPL* | 4.94×10^-8^ | [1] |
| *rs7800944* | 7 | 73035857 | *MLXIPL* | 1.30×10^-8^ | [26] |
| *rs799166* | 7 | 73051932 | *5' of MLXIPL* | 4.02×10^-8^ | [1] |
| *rs10480300* | 7 | 151406005 | *PRKAG2* | 4.09×10^-9^ | [1] |
| *rs17786744* | 8 | 23777006 | *5' of STC1* | 1.40×10^-8^ | [1] |
[truncated: 70,579 more chars]
